# SmI_2_-Catalyzed Intermolecular Coupling of Cyclopropyl Ketones and Alkynes: A Link between Ketone Conformation and Reactivity

**DOI:** 10.1021/jacs.1c01356

**Published:** 2021-02-25

**Authors:** Soumitra Agasti, Nicholas A. Beattie, Joseph J. W. McDouall, David J. Procter

**Affiliations:** Department of Chemistry, The University of Manchester, Oxford Road, Manchester M13 9PL, U.K.

## Abstract

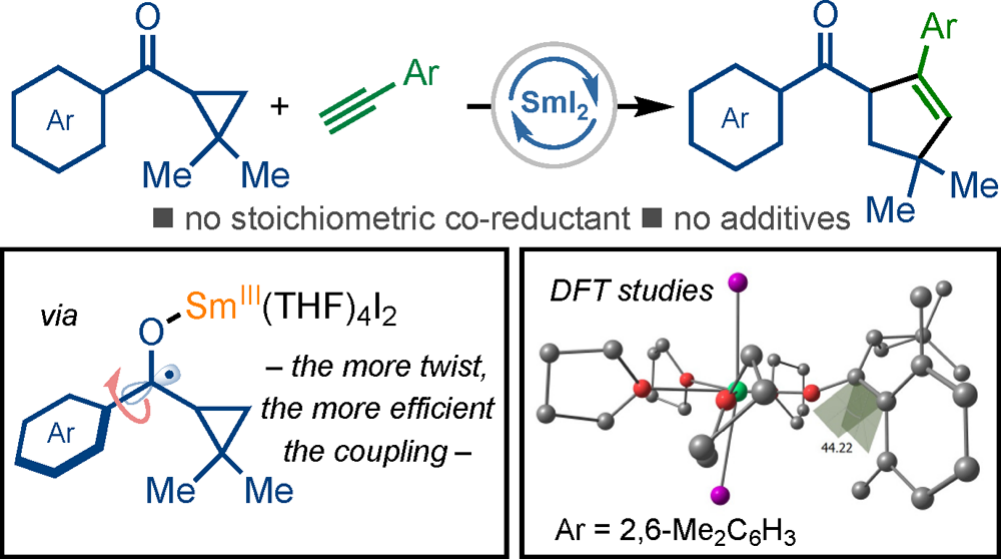

The archetypal single electron transfer reductant, samarium(II) diiodide (SmI_2_, Kagan’s reagent), remains one of the most important reducing agents and mediators of radical chemistry after four decades of widespread use in synthesis. While the chemistry of SmI_2_ is very often unique, and thus the reagent is indispensable, it is almost invariably used in superstoichiometric amounts, thus raising issues of cost and waste. Of the few reports of the use of catalytic SmI_2_, all require the use of superstoichiometric amounts of a metal coreductant to regenerate Sm(II). Here, we describe a SmI_2_-catalyzed intermolecular radical coupling of aryl cyclopropyl ketones and alkynes. The process shows broad substrate scope and delivers a library of decorated cyclopentenes with loadings of SmI_2_ as low as 15 mol %. The radical relay strategy negates the need for a superstoichiometric coreductant and additives to regenerate SmI_2_. Crucially, our study uncovers an intriguing link between ketone conformation and efficient cross-coupling and thus provides an insight into the mechanism of radical relays involving SmI_2_. The study lays further groundwork for the future use of the classical reagent SmI_2_ in contemporary radical catalysis.

## Introduction

**Scheme 1:**
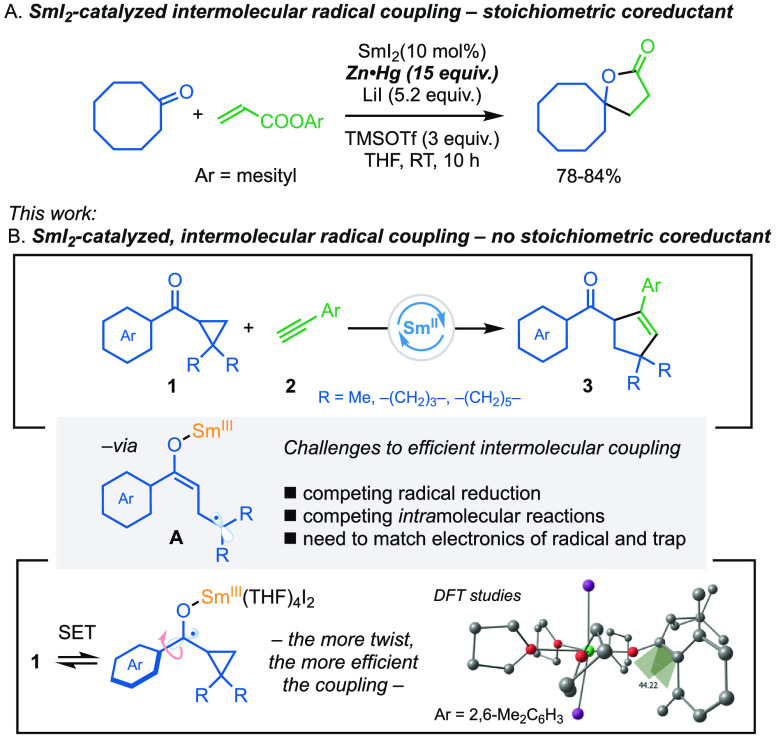
SmI_2_-Catalyzed Intermolecular Radical Couplings (A) Using a stoichiometric coreductant to regenerate Sm(II). (B) This work. Using a radical relay to regenerate Sm(II). The crucial link between conformation and the efficiency of the coupling. TMS = trimethylsilyl.

The archetypal single electron transfer (SET)^1^ reductant, samarium(II) diiodide (SmI_2_, Kagan’s reagent),^2^ remains one of the most important reducing agents and mediators of radical chemistry after four decades of widespread use in synthesis.^3^ Intramolecular processes using the commercially available reagent are particularly popular, and SmI_2_-mediated radical cyclizations feature in the total synthesis of numerous high profile and complex natural products.^4^ Intermolecular processes using SmI_2_ are inherently more challenging as intermolecular radical C–C bond formation must outrun the competing reduction of radicals to carbanions. While the chemistry of SmI_2_ is very often unique, and thus the reagent is indispensable,^2−4^ it is almost invariably used in superstoichiometric amounts, thus raising issues of cost and waste. Of the few reports of the use of catalytic SmI_2_, all require the use of superstoichiometric amounts of a metal coreductant to regenerate Sm(II).^5^ For example, Corey described one of the very few SmI_2_-catalyzed intermolecular coupling processes:^5b^ Unfortunately, the catalytic system requires 15 equiv of Zn/Hg amalgam (Scheme 1A).

We recently reported a radical-relay approach to catalysis with SmI_2_ that negates the need for coreductants and additives: cyclopropyl ketones underwent catalytic radical cyclization to give complex bicyclic ketones.^6^ We envisaged that unprecedented and more-challenging, intermolecular couplings might be possible using catalytic SmI_2_, as the reduction of radical intermediates **A** would be less-problematic at lower concentrations of the reagent.

Herein, we disclose an efficient method for the construction of decorated cyclopentenes using an intermolecular radical coupling of aryl cyclopropyl ketones and alkynes catalyzed by SmI_2_. Prior to this study, the only previous intermolecular radical coupling of cyclopropyl ketones and alkynes was an enantioselective process utilizing a noncommercial, chiral-at-rhodium complex and requiring imidazolyl cyclopropyl ketones capable of two-point binding to the metal.^8f^ Crucially, our studies uncover an intriguing link between ketone conformation and efficient coupling and thus provide an insight into the mechanism of radical relays^7,8^ involving SmI_2_ (Scheme 1B).

## Results and Discussion

Optimization studies began with the SmI_2_-mediated coupling of readily available cyclopropyl phenyl ketones **1a**–**c** and phenylacetylene **2a** (Table 1). While the use of 25 mol % of SmI_2_ with phenyl ketone **1a** resulted in low conversion and a 35% yield of **3a** (entry 1), byproduct **4** was also observed in the product mixture (9%). In an attempt to block competing intramolecular radical addition, the use of 2-methylphenyl ketone **1b** was investigated; under identical conditions, **1b** gave **3b** in 99% isolated yield after 45 min (entry 2). Lowering the reaction temperature (entry 3) or the catalytic loading of SmI_2_ to 20 mol % (entry 4) and 15 mol % (entry 5) led to lower conversion. However, switching to 2,6-dimethylphenyl ketone **1c** and using 15 mol % SmI_2_ gave **3c** in 87% yield (entry 6). The use of the corresponding cyclohexyl cyclopropyl ketone **1d** resulted in no product formation, and starting materials were recovered unchanged (entry 7) (vide infra). This is likely due to reversible reduction of the carbonyl and/or reversible fragmentation.

**Table 1:**
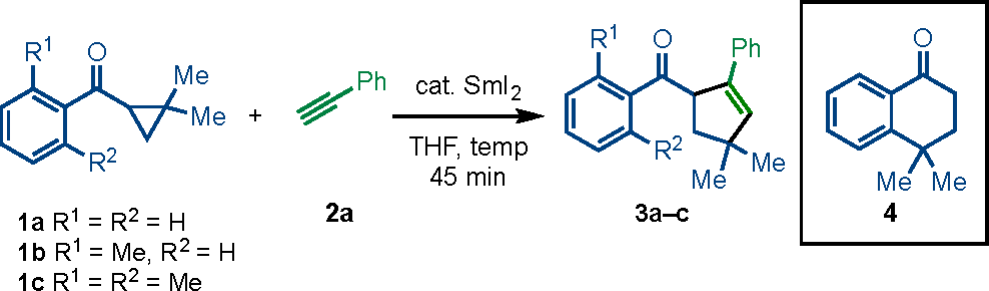
Screening of Catalytic Conditions^a^

entry	ketone	temp. (°C)	SmI_2_ loading	Conversion	Yield of **3**^a^
1	**1a**	55	25 mol %	40%	35%^b^
2	**1b**	55	25 mol %	100%	99%^b^
3	**1b**	RT	25 mol %	85%	82%^c^
4	**1b**	55	20 mol %	85%	79%
5	**1b**	55	15 mol %	63%	50%
6	**1c**	55	15 mol %	89%	87%
7	**1d**^d^	55	25 mol %	0%	0%

a Reaction conditions: **1a**–**c** (1 equiv), **2a** (5 equiv), SmI_2_ (0.1 M in THF), in THF (0.5 mL/0.1 mmol of substrate) under nitrogen. ^a^NMR yield using nitromethane as internal standard.

b Isolated yield given.

c Reaction time, 16 h.

d Cyclohexyl 2,2-dimethylcyclopropylketone **1d** was used. THF = tetrahydrofuran.

**Figure 1:**
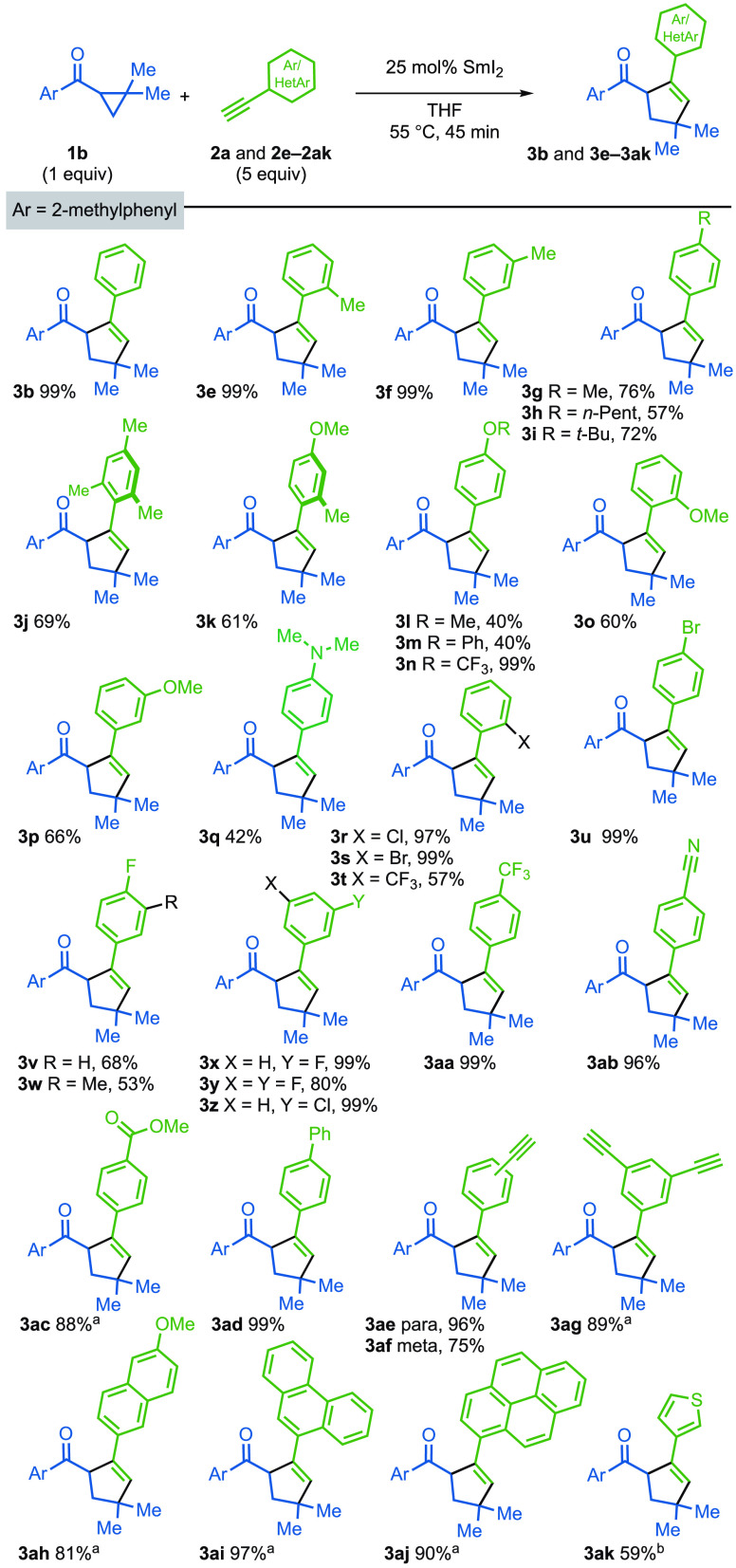
Scope with respect to the aryl alkyne. Reaction conditions: **1b** (1 equiv), **2** (typically 5 equiv), 25 mol % SmI_2_ (0.1 M in THF), in THF (0.5 mL/0.1 mmol of substrate) under nitrogen. Isolated yields; ^a^ using 2.5 equiv of **2**; ^b^ using 40 mol % SmI_2_.

The scope of the reaction with regard to the aryl alkyne was explored using 2-methylphenyl ketone **1b** (Figure 1). The presence of electron-releasing alkyl, alkoxy, amino, and trifluoromethoxy groups on the aryl substituent of the alkyne was tolerated (**3e**-**3q**). In line with the intermolecular addition of a nucleophilic radical (cf. **A** in Scheme 1B) to the alkyne, aryl alkynes bearing electron-withdrawing groups (e.g., bromo, fluoro, trifluoromethyl, phenyl, nitrile, and carbomethoxy) generally gave higher yields of **3** (**3r**–**3ad**). Diynes and a triyne underwent monocoupling to give **3ae**-**3ag** in high yield. Naphthyl (**3ah**), phenanthrenyl (**3ai**), and pyrenyl (**3aj**) motifs were tolerated, as was the important heteroaromatic, thiophene (**3ak**). Crucially, functional groups that are typically reduced by SmI_2_ (e.g., carbomethoxy, nitrile and bromo) are unreactive under the catalytic conditions. The alkyl substituted alkyne, prop-2-yn-1-ylcyclopentane, was unreactive, as was phenyl propiolate. Attempted coupling with benzofuran was unsuccessful, and starting materials were recovered.^6^ For ineffective coupling partners, it appears that trapping of the radical formed upon reversible fragmentation of the cyclopropyl ring is inefficient and starting ketone is recovered. See the Supporting Information for further details and a table of unsuccessful coupling partners.

**Figure 2:**
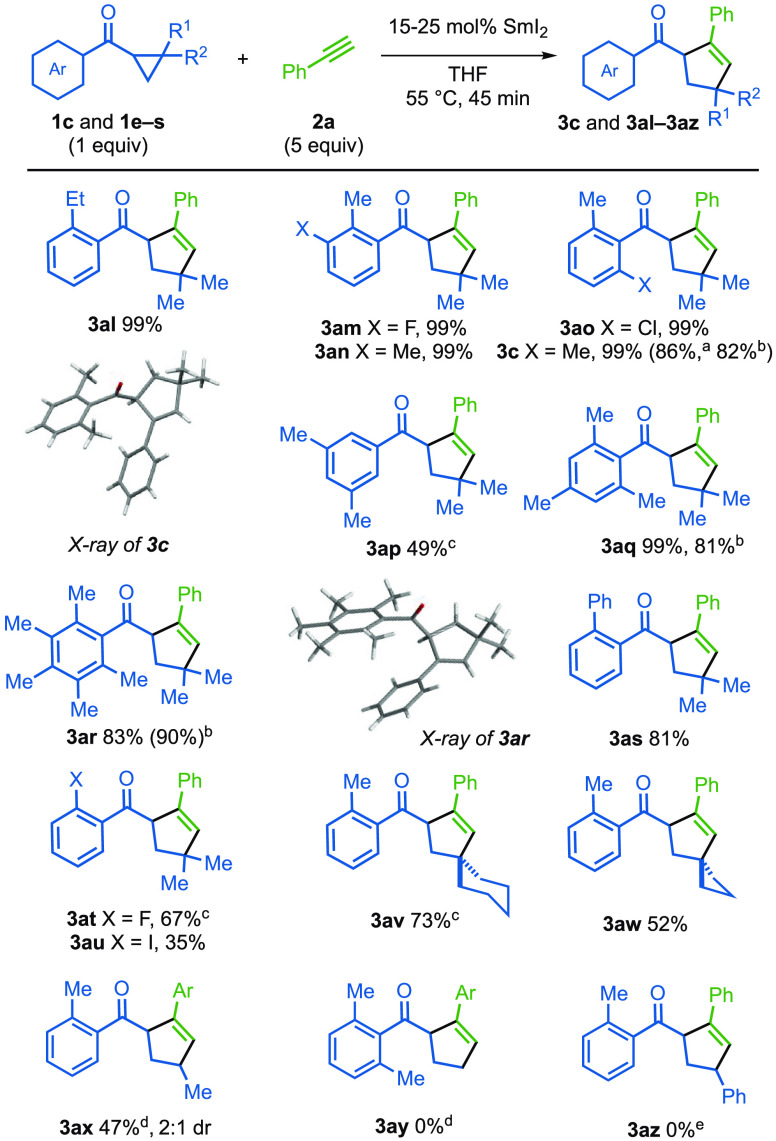
Scope with respect to the aryl cyclopropyl ketone. Reaction conditions: **1** (1 equiv), **2** (typically 5 equiv), 25 mol % SmI_2_ (0.1 M in THF), in THF (0.5 mL/0.1 mmol of substrate) under nitrogen. Isolated yields. ^a^ Using 1.01 g of ketone partner. ^b^ Using 15 mol % of SmI_2_. ^c^ Using 40 mol % SmI_2_. ^d^ 4-Ethynylbenzonitrile was used as the alkyne partner. ^e^ Starting ketone was recovered.

We next varied the aryl cyclopropyl ketone partner **1** (Figure 2). As noted during optimization studies, the presence of an *ortho*-methyl substituent on the aryl ring had a marked, beneficial effect on the efficiency of the catalytic radical coupling. In addition, ethyl (**3al**), fluoro (**3am, 3at**), chloro (**3ao**), phenyl (**3as**), and iodo (**3au**) substituents on the aryl ring of the ketone were compatible with the catalytic coupling. Ortho substitution was again seen to have a clear, beneficial impact on the efficiency of coupling; compare the yield of **3c** with that of **3ap**. The use of conveniently prepared spirocyclic cyclopropylketones gave spirocycles **3av** and **3aw** in 73% and 52%, respectively.

Finally, the importance of gem-dialkyl substitution on the cyclopropane ring in **1** was probed; monomethyl substrate **1q** gave **3ax** in moderate yield and as a 2:1 mixture of diastereoisomers, while the use of the simple, unsubstituted cyclopropyl ketone failed to deliver **3ay**. Cyclopropyl ketone **1s**, bearing a phenyl substituent on the cyclopropane ring, failed to deliver **3az** and starting ketone was recovered; although the radical anion intermediate derived from **1s** is likely to undergo facile ring-opening,^9a^ the benzylic radical from cyclopropane fragmentation appears to be insufficiently reactive to be trapped by the alkyne. Pleasingly, **3c** was prepared on a 1 g scale in 86% while **3ar** and **3c** were prepared on a 1 mmol scale in 90% yield and 82% yield, respectively, with a reduced 15 mol % loading of SmI_2_.

**Scheme 2:**
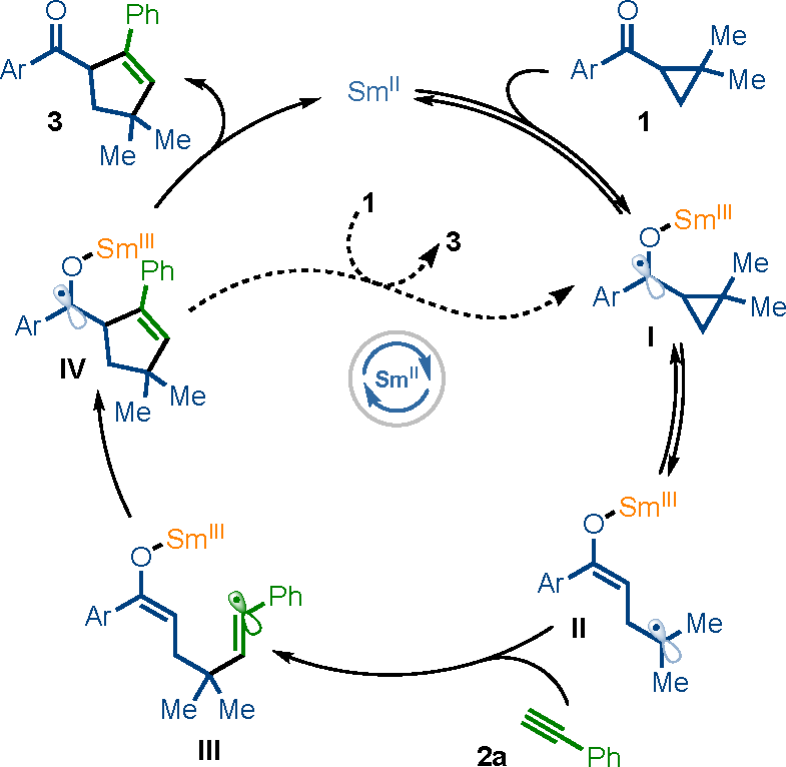
Proposed Radical Relay for the Intermolecular Radical Coupling

In line with our previous mechanistic studies,^6^ we propose a radical-relay mechanism for the SmI_2_-catalyzed, intermolecular radical coupling (Scheme 2): Note that exposure of **1b** and **2a** to various Lewis acids (e.g., SmI_3_, Yb(OTf)_3_, La(OTf)_3_) gave no trace of **3b**, thus ruling out a Lewis acid-mediated coupling.^10^ Reversible SET from SmI_2_ to ketone **1** gives ketyl radical **I**(11) which fragments^9b^ to give enolate/radical **II**. Intermolecular coupling with **2a** then generates radical **III** which rebounds by addition to the Sm(III)-enolate moiety, generating new ketyl radical **IV**. Back electron transfer to Sm(III) regenerates the SmI_2_ catalyst and liberates product **3**. It is also possible that ketyl radical **IV** directly reduces starting ketone **1**.^12^ Calculations (vide infra) suggest that product ketyl radical **IV** has a similar reducing ability to the starting ketyl radical **I**. Thus, rather than a case of “reductant upconversion”,^12b^ in which a more reducing radical is formed from a less-reducing radical and an electron-transfer chain process results, we believe it is the instability of the starting ketyl radical **I** and the formation of a more stable product ketyl radical **IV** that is key to the catalytic process.

**Figure 3:**
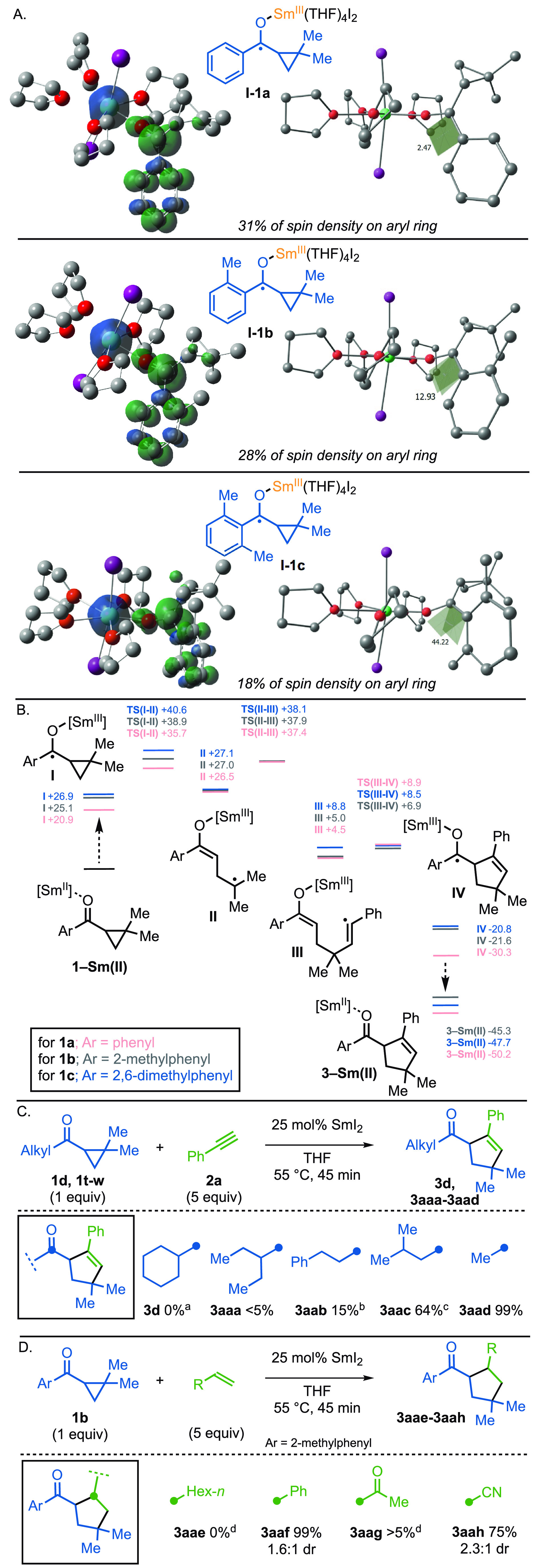
Computational studies. (A) Probing the importance of conformation on ketyl radical stability. (B) Mechanism of the catalytic radical coupling and the influence of the aryl substituent. Level of theory; PBE0/Def2-TZVP/PCM(THF)/D3(B-J)//Def2-SVP. (C) Scope with respect to alkyl cyclopropyl ketones. Reaction conditions: **1** (1 equiv), **2a** (5 equiv), 25 mol % SmI_2_ (0.1 M in THF), in THF (0.5 mL/0.1 mmol of substrate) under nitrogen. Isolated yields. ^a^ Starting ketone was recovered. ^b^ Starting ketone was recovered in 81% yield. ^c^ Starting ketone was recovered in 22% yield. (D) Survey of alkene partners. Reaction conditions: **1** (1 equiv), alkene (5 equiv), 25 mol % SmI_2_ (0.1 M in THF), in THF (0.5 mL/0.1 mmol of substrate) under nitrogen. Isolated yields. ^d^ Starting ketone was recovered.

Computational studies (PBE0/Def2-TZVP/PCM(THF)/D3(B-J)//Def2-SVP) have been used to probe the mechanism of the catalytic, intermolecular, radical coupling and, in particular, the crucial impact that ortho-substitution in the aryl ketones **1** has on the efficiency of coupling (Figure 3). First, we examined the conformation of the samarium ketyl radicals derived from ketones **1a**–**c** (cf. **I** in Scheme 2) and the distribution of spin density in the radicals (Figure 3A). The main impact of the ortho-methyl substituents in ketones **1b** and **1c** is that the aryl rings are twisted out of the plane of the ketone carbonyl. This can be seen for ketyl radicals **I-1b** and **I-1c**, in which the aryl rings are 13° and 44°, respectively, out of plane. This renders the ketyl radicals **I-1b** and **I-1c** less stable, with less spin density in the aromatic ring and more of the spin density localized on what was the ketone carbonyl carbon (31% spin density on the aryl ring **I-1a**, 28% in **I-1b** and 18% in **I-1c**) (Figure 3A).

Crucially, the destabilization of the ketyl radical **I** appears to lower the barrier for ring-opening of the cyclopropyl ring to give radicals **II**; the computed barrier for ring opening of **I-1a** is 14.8 kcal mol^–1^, while those for **I-1b** and **I-1c** are 13.8 and 13.7 kcal mol^–1^, respectively (Figure 3B). It is interesting to note that the **TS(I–II)** is also destabilized, as ortho substituents are introduced to the aryl ring, but to a slighter lesser extent, while the stability of the radical product of ring-opening **II** is largely unaffected by the nature of the aryl ring as the spin density is now remote from the aryl substituent. It is important to note that destabilization of ketyl radical **I** makes SET from Sm(II) to the ketone a higher energy process (20.9 kcal mol^–1^ for **1a**; 25.1 kcal mol^–1^ for **1b**, and; 26.9 kcal mol^–1^ for **1c**); however, this energy cost is repaid in full at the end of the relay during the favorable reduction of Sm(III) by the ketyl radical.

Building on our proposal that aryl cyclopropyl ketones are more effective substrates when their aryl rings are twisted out of the plane, thus rendering the ketyl radicals formed upon reduction less stable, we revisited the use of alkyl cyclopropyl ketones in the reaction. As discussed previously, cyclohexyl cyclopropyl ketone **1d** was unreactive and the use of other bulky alkyl cyclopropyl ketones resulted in low yields or the return of only starting material. However, *i*-butyl and methyl cyclopropyl ketones underwent smooth coupling, to give **3aac** and **3aad**, respectively (Figure 3C). Attempts to switch alkyne partners for alkenes showed that the use of simple alkenes was not effective (e.g., oct-1-ene), although coupling was seen with the activated acceptors, styrene and acrylonitrile, to give **3aaf** and **3aah**, respectively (Figure 3D).

**Figure 4:**
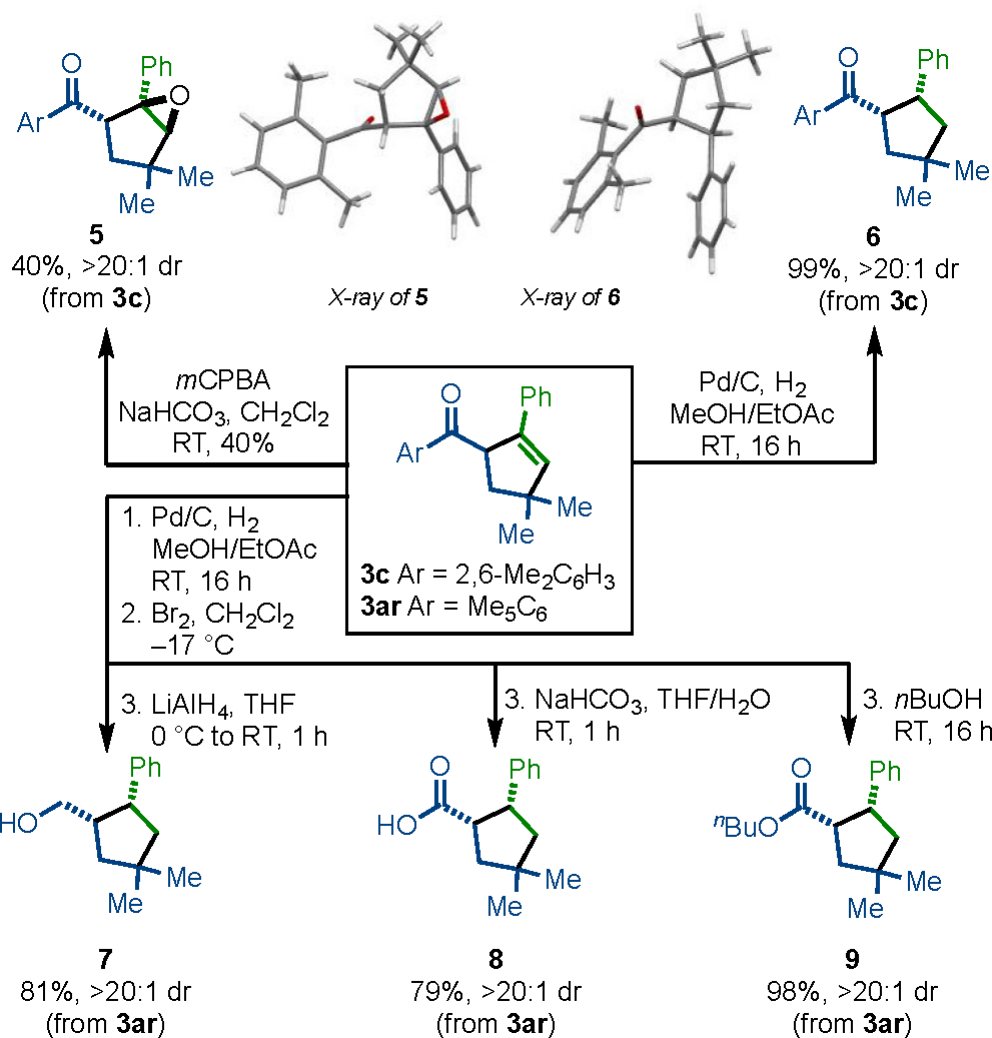
Selective manipulation of the products of SmI_2_-catalyzed intermolecular coupling.

The products of the SmI_2_-catalyzed cross-coupling are versatile building blocks for synthesis. For example, cyclopentene **3c** can be selectively oxidized and reduced to give epoxide **5** and ketone **6**, respectively (Figure 4). Furthermore, the product of cross-coupling **3ar**, bearing the pentamethylphenyl ketone motif, can be efficiently converted to the corresponding alcohol **7**, acid **8**, and ester **9**.^13^

## Conclusion

In summary, SmI_2_ catalyzes the radical cross-coupling of aryl cyclopropyl ketones and alkynes. The process shows broad substrate scope and delivers a library of decorated cyclopentenes with loadings down to 15 mol % of SmI_2_. We invoke the operation of a radical relay mechanism that negates the need for a superstoichiometric coreductant and additives to regenerate Sm(II). Crucially, our study uncovers an intriguing link between ketone conformation and efficient cross-coupling and thus provides an insight into the mechanism of radical relays involving SmI_2_. The study lays further groundwork for the future use of the classical SET reagent SmI_2_ in contemporary radical catalysis.

## Experimental Section

### Preparation of SmI_2_

An oven-dried round-bottom flask, equipped with a stirrer bar, was flushed with a strong flow of N_2_ for 30 min. The flask was then loaded with samarium metal (∼40 mesh, 1.4 equiv), washed diiodoethane (1 equiv), and the flask was flushed for another 30 min with N_2_. Freshly distilled and degassed THF (0.1 M) was added, and the mixture was stirred overnight at room temperature. Finally, the mixture was allowed to settle for at least 1 h and titrated prior to use.^14^

### General Procedure for the Catalytic Intermolecular Coupling

To an oven–dried microwave reaction vial containing a stirrer bar, was added ketone **1** (0.1 mmol, 1 equiv), and the vial was flushed with N_2_. After 15 min, THF (0.5 mL) and alkyne **2** (0.5 mmol, 5 equiv) were introduced by syringe. The vial was placed in a preheated oil bath at 55 °C, followed by the addition of freshly prepared SmI_2_ (typically 25 mol %, 0.1 M, 0.250 mL). The reaction was stirred vigorously (400 rpm) for 45 min. The reaction mixture was cooled to room temperature and filtered through a silica gel pad (100–200 mesh size), washing with CH_2_Cl_2_ (15 mL). Solvent was removed *in vacuo*, and the desired compound **3** was obtained without further purification. In a few cases, the product **3** was purified by column chromatography on silica gel (100- 200 mesh size) with hexane/ethyl acetate as eluent.

## Supporting Information Available

Experiments, experimental procedures, characterization data, CCDC numbers for X-ray crystal structures, details of computational studies, and spectra for all new compounds (PDF)

## Accession Codes

CCDC 2039352–2039355 contain the supplementary crystallographic data for this paper. These data can be obtained free of charge via www.ccdc.cam.ac.uk/data_request/cif, or by emailing data_request@ccdc.cam.ac.uk, or by contacting The Cambridge Crystallographic Data Centre, 12 Union Road, Cambridge CB2 1EZ, UK; fax: +44 1223 336033.
