# Supplementary material for: SmI2-Catalyzed Intermolecular Coupling of Cyclopropyl Ketones and Alkynes: A Link between Ketone Conformation and Reactivity
Source: J Am Chem Soc. 2021 Feb 25;143(9):3655–61. doi: 10.1021/jacs.1c01356 (PMC8028054; doi:10.1021/jacs.1c01356)

Supporting Information

SmI_2-catalyzed intermolecular coupling of cyclopropyl ketones and alkynes; a link between ketone conformation and reactivity

Soumitra Agasti, Nicholas A. Beattie, Joseph J. W. McDouall, and David J. Procter*

Table of Contents

1. General Information……………………………………………………….S2
2. Preparation of SmI_2...S2
3. Starting Material Preparation……………………………………………..S3
    3a. Synthetic Procedures……………………………………………....S3
    3b. Characterization Data……………………………………………....S7
4. Catalytic Intermolecular Couplings………………………………………S16
    4a. General Procedure …………………………………………………S16
    4b. Characterization Data of Coupling products…………….............S16
5. Mechanistic Study…………………………………………………………S47
6. Reaction Colour *vs* Conversion Study…………………………………..S49
7. Large Scale Reactions……………………………………………………S51
8. Product Manipulation……………………………………………………… S52
9. Unsuccessful Radical Acceptor Substrates…………………………......S57
10. Computational Study…………………………………………………….S58
11. References ……………………………………………………………….S82
12. X-ray Structures………………………………………………………….S83
13. NMR Spectra …………………………………………………………….S87

1. General Information:

All experiments were performed under a nitrogen atmosphere unless stated otherwise. All solvents were purchased at the highest commercial grade and used as received or after distillation from sodium/benzophenone under nitrogen (THF). Diiodoethane was washed with diethyl ether and sodium thiosulfate before use. All other chemicals were purchased at the highest commercial grade and used as received. ^{1}H NMR spectra were recorded on NMR spectrometers at 400 MHz and 500 MHz and ^{13}C NMR at 101 MHz and 126 MHz. ^{1}H NMR chemical shifts (δH) and ^{13}C NMR chemical shifts (δC) are quoted in parts per million (ppm) downfield from trimethylsilane (TMS) and coupling constants (*J*) are quoted in Hertz (Hz). Abbreviations for NMR data are s (singlet), d (doublet), t (triplet), q (quartet), quin (quintet), sxt (sextet). Infrared (IR) spectra were recorded on a FTIR spectrometer and mass spectra were obtained using positive or negative electrospray ionization (ESI), atmospheric pressure chemical ionization (APCI), electron impact ionization (EI) or chemical ionization (CI) techniques. ^{1}H NMR and ^{13}C NMR spectra were assigned with the aid of COSY, HSQC, HMBC, DEPT 135 and nOe NMR techniques and stereochemistry assigned with the aid of X-ray crystallography. Chromatography was carried out using silica gel 60 Angstrom (Å), 100–200 mesh. Thin layer chromatography (TLC) was performed on aluminium sheets pre-coated with silica gel, 0.20 mm (Macherey-Nagel, Polygram@ Sil G/UV254). TLC plates were visualized by UV absorption or potassium permanganate solution and heating.

2. Preparation of SmI_2:

An oven-dried round bottom flask, equipped with a stirrer bar, was flushed with a strong flow of N_2 for 30 min. Subsequently, the flask was loaded with samarium metal (~40 mesh, 1.4 eq.) and washed diiodoethane (1 eq.). It was then flushed for another 30 min, before freshly distilled and degassed THF (0.1 M) was added followed by continuous stirring overnight at room temperature. Finally, the mixture was allowed to settle for at least one hour and titrated prior to use.[1]

3. Starting Material Preparation:[2]

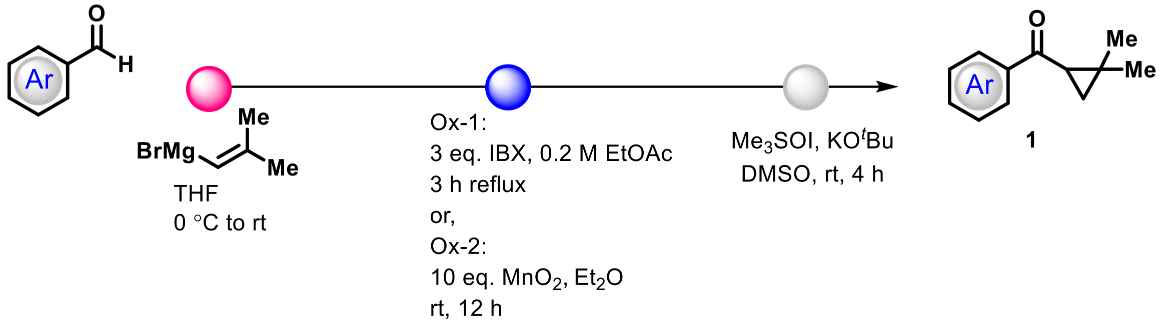

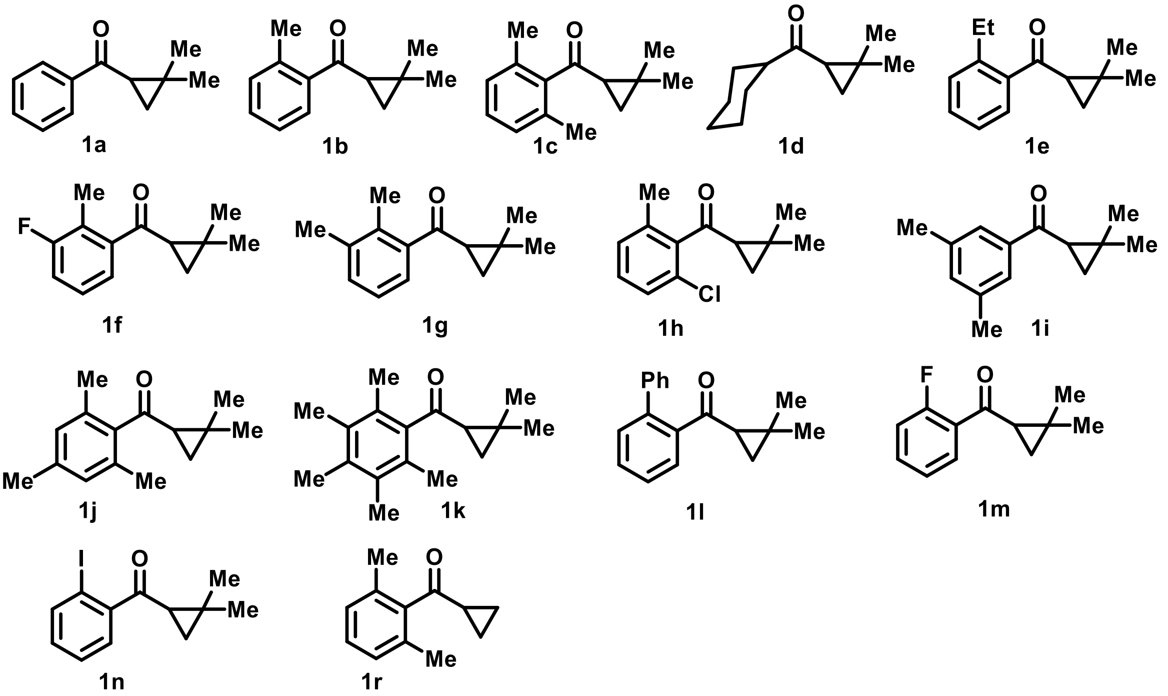

3a. Synthetic Procedures:

The corresponding aldehyde (7.5 mmol, 1 eq.) was placed in a round bottom flask under N_2. The flask was cooled to 0 ºC and dry THF (15 mL) was added. 2-Methyl-1-propenylmagnesium bromide solution (8.25 mmol, 1.1 eq.) was added dropwise at 0 ºC and the reaction mixture was stirred at room temperature for 2 h. Subsequently, the reaction mixture was quenched with water and extracted with EtOAc (3 × 15 mL). The organic layers were collected, dried over $MgSO_4$ and concentrated in vacuo. The desired compound was isolated by column chromatography on silica gel using petroleum ether and EtOAc as eluent.

Ox-1:[3] To a solution of the corresponding alcohol in EtOAc (0.2 M) was added IBX (3 eq.) in one portion and the solution heated under reflux for 3 h. Subsequently, the reaction was cooled to room temperature and filtered through a celite pad. Volatiles were removed in vacuo and the product was purified by column chromatography on silica gel using petroleum ether and EtOAc as eluent.

Ox-2: In an oven dried round bottom flask, to the corresponding alcohol in Et_2O (6 mL/mmol) was added MnO_2 (10 eq.) and the reaction mixture was stirred vigorously for 12 h at room temperature. The reaction mixture was filtered, and the excess solvent was removed in vacuo. The product was purified by column chromatography on silica gel using petroleum ether and EtOAc as eluent.

To potassium *tert*-butoxide (1.2 eq.) and trimethylsulfoxonium iodide (1.2 eq.) under N_2 in a round bottom flask was added DMSO (3.5 mL/mmol) and the reaction mixture stirred for 15 min at room temperature until the solution became clear. Additionally, ketone derivative (1 eq.) was dissolved in DMSO (2 mL/mmol) and the solution added into the reaction mixture in one portion. The reaction mixture was allowed to stir at room temperature for 4 h before quenching by the addition of water. The aqueous layer was extracted with Et_2O (3 × 15 mL) and the organic layer was dried over $MgSO_4$. The combined organic layer was concentrated in vacuo and the crude product was isolated by column chromatography on silica gel using petroleum ether and EtOAc as eluent.

Synthesis of **1o** and **1p:**

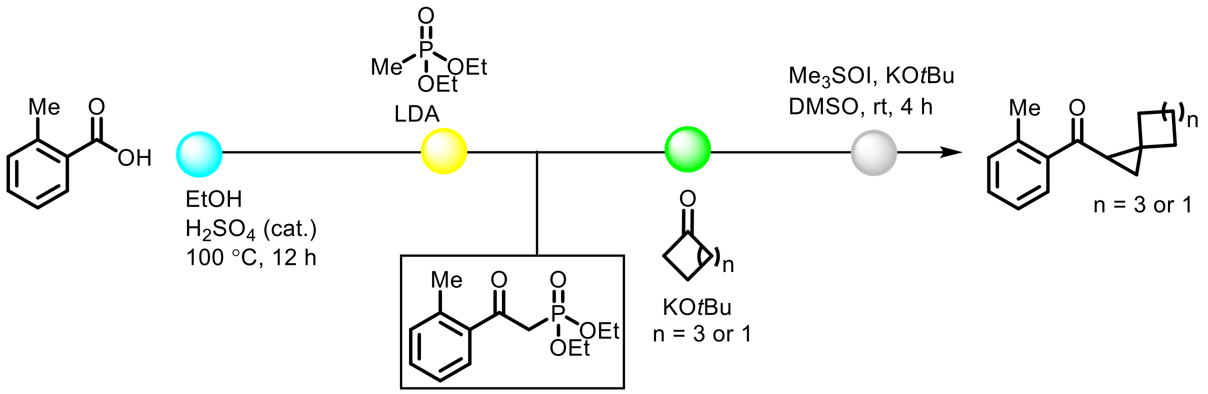

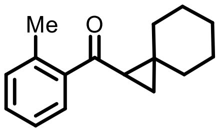

1o

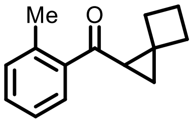

1p

To a solution of 2-methylbenzoic acid (3 g, 22 mmol) in EtOH was added a cat. amount of H_2SO_4 and the solution heated under reflux for 12 h. Subsequently, the reaction mixture was cooled to room temperature and neutralized with saturated aqueous Na_2CO_3. The mixture was then extracted with CH_2Cl_2 (3 × 15 mL). Organic layers were dried over $MgSO_4$ and concentrated under reduced pressure. Ethyl 2-methylbenzoate was isolated by column chromatography on silica gel using petroleum ether and EtOAc as eluent (quantitative yield).

An oven dried round bottom flask was charged with ethyl 2-methylbenzoate (16.8 mmol, 1.4 eq.) and methyl diethylphosphonate (12 mmol, 1 eq.) and the flask was placed under N_2. The reaction mixture was then cooled to 0 ºC and dry THF (15 mL) was added. After 30 min, LDA solution (24 mmol, 2 eq.) was added dropwise at 0 ºC and the reaction mixture was stirred at the same temperature for another 30 min. Subsequently, the reaction mixture was quenched with saturated aqueous NH_4Cl and extracted with Et_2O (3 × 40 mL). The combined organic layers were dried over $MgSO_4$ and concentrated under reduced pressure. The desired phosphonate ester was isolated by column chromatography on silica gel using petroleum ether and EtOAc as eluent.

To potassium *tert*-butoxide (2 mmol, 1 eq.) in a round bottom flask under N_2 was added dry THF (2 mL/mmol). Phosphonate ester (2 mmol, 1 eq.) was added dropwise and the reaction mixture was stirred at room temperature for 2 h. After this time, ketone (1 eq.) was added dropwise to the reaction mixture. The reaction flask was then equipped with a condenser and heated under reflux for 16 h. The reaction mixture was then filtered and diluted with Et_2O followed by brine. The aqueous layers were extracted with Et_2O (3 × 10 mL). The combined organic layer was dried over $MgSO_4$ and concentrated in vacuo to give the crude product. The cyclopropyl ketone was isolated by column chromatography on silica gel using petroleum ether and EtOAc as eluent.

Synthesis of **1q:**

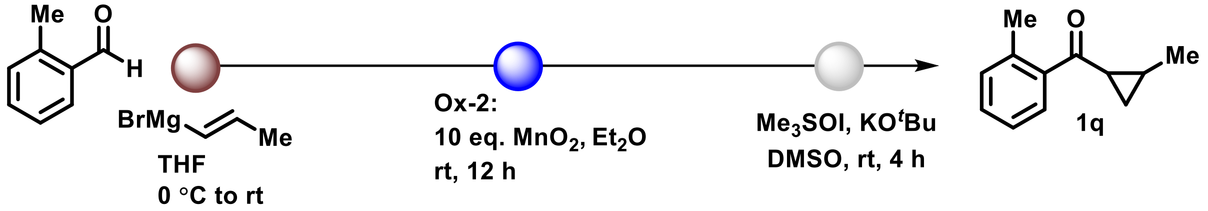

To 2-methylbenzaldehyde (7.5 mmol, 1 eq.) in a round bottom flask under N_2 at 0 ºC was added dry THF (15 mL). 1-Propenylmagnesium bromide solution (8.25 mmol, 1.1 eq.) was added dropwise at 0 ºC and the reaction mixture was stirred at room temperature for 2 h. Subsequently, the reaction mixture was quenched with water and extracted with EtOAc (3 × 15 mL). The organic layers were collected, dried over $MgSO_4$ and concentrated in vacuo. The desired compound was isolated by column chromatography on silica gel using petroleum ether and EtOAc as eluent.

Synthesis of **1s:**

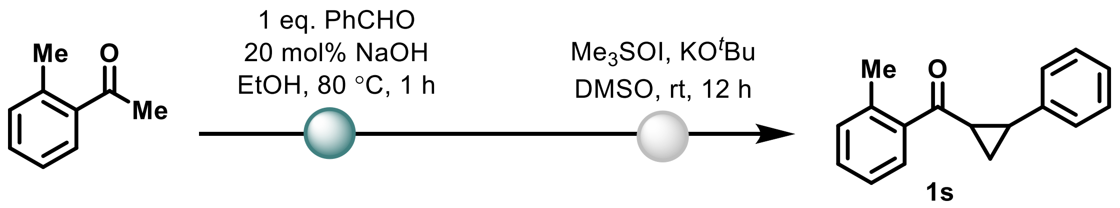

To a solution of 2-methylacetophenone (10 mmol, 1 eq.) in EtOH was added NaOH (20 mol%), benzaldehyde (10 mmol, 1 eq.) and the solution heated under reflux for 1 h. Subsequently, the reaction mixture was cooled to room temperature and neutralized with 1 (M) HCl. The mixture was then extracted with CH_2Cl_2 (3 × 30 mL). Organic layers were dried over $MgSO_4$ and concentrated under reduced pressure. The desired chalcone derivative was isolated by column chromatography on silica gel using petroleum ether and EtOAc as eluent.

Synthesis of **1t-v:**

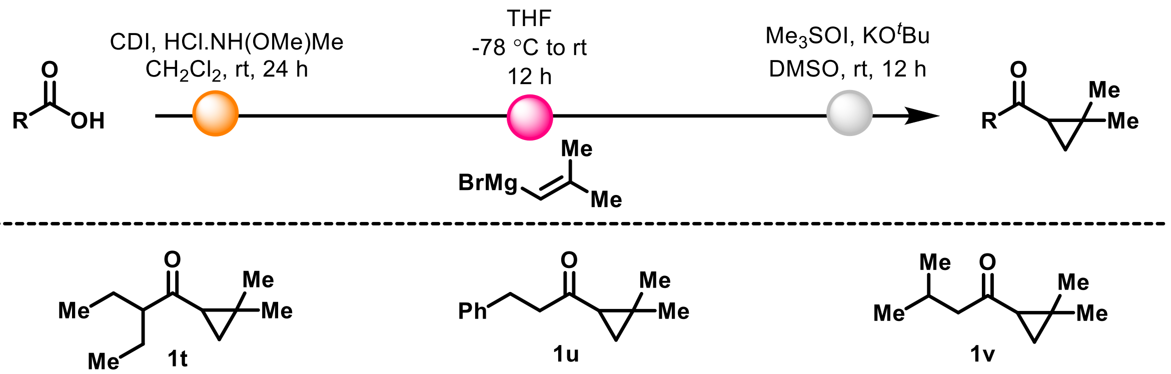

To a solution of carboxylic acid (20 mmol, 1eq.) in CH_2Cl_2 (60 mL) was added carbonyldiimidazole (23 mmol, 1.15 eq.) portion wise and the solution stirred at room temperature for 1 h. After this time, N_2 was bubbled through the solution for 30 min. Then, *N*,*O*-dimethylhydroxylamine hydrochloride (26 mmol, 1.3 eq.) was added and the reaction mixture was stirred at room temperature for 24 h. Subsequently, the reaction mixture was quenched with saturated aqueous NH_4Cl and extracted with CH_2Cl_2 (3 × 70 mL). The combined organic layer was dried over $MgSO_4$ and concentrated in vacuo to afford the product.

The corresponding amide (1 eq.) was placed in a round bottom flask under N_2. The flask was cooled to -78 ºC and dry THF (5 mL/mmol) was added. 2-Methyl-1-propenylmagnesium bromide solution (1.2 eq.) was added dropwise at -78 ºC and the reaction mixture was stirred at -78 ºC for 15 min. The reaction mixture was then stirred at room temperature for 12 h. Subsequently, the reaction mixture was quenched with saturated aqueous NH_4Cl and extracted with CH_2Cl_2 (3 × 30 mL). The organic layers were collected, dried over $MgSO_4$ and concentrated in vacuo. The desired compound

was isolated by column chromatography on silica gel using petroleum ether and EtOAc as eluent.

Synthesis of **1w:**

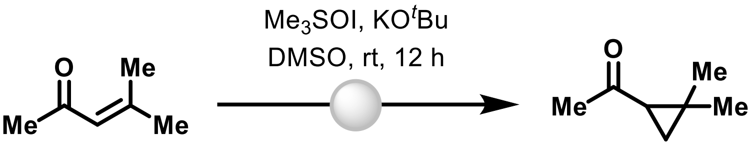

1w

Title compound **1w** was prepared from commercially available mesityl oxide according to the procedure described in page S4. The reaction was performed for 12 h.

3b. Characterization Data:

(2,2-Dimethylcyclopropyl)(phenyl)methanone (**1a**)[2]

Yield: 40% over 3 steps (using Ox-2)

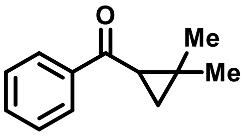

^{1}H NMR (500 MHz, Chloroform-*d*) δ: 7.97 – 7.92 (m, 2H, Ar*H*), 7.58 – 7.51 (m, 1H, Ar*H*), 7.57 – 7.43 (m, 2H, Ar*H*), 2.48 (dd, *J* = 7.4, 5.7 Hz, 1H, C(O)C*H*), 1.52 (dd, *J* = 5.4, 4.3 Hz, 1H, CHC*H*$_2$), 1.36 (s, 3H, C(C*H*$_3$)$_2$), 1.09 (s, 3H, C(C*H*$_3$)$_2$), 0.95 (dd, *J* = 7.5, 4.1 Hz, 1H, CHC*H*$_2$).

^{13}C NMR (126 MHz, Chloroform-*d*) δ: 198.8 (*C*=O), 139.3 (Ar*C*^{q}), 132.6 (Ar*C*H), 128.7 (2 × Ar*C*H), 128.2 (2 × Ar*C*H), 33.1 (C(O)*C*H), 27.3 (C(*C*H$_3$)$_2$), 27.2 (C(*C*H$_3$)$_2$), 22.2 (CH*C*H$_2$), 18.7 (CH*C*(CH$_3$)$_2$).

(2,2-Dimethylcyclopropyl)(o-tolyl)methanone (**1b**)

Yield: 48% over 3 steps (using Ox-2)

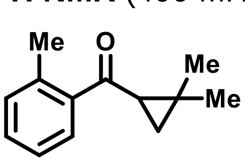

^{1}H NMR (400 MHz, Chloroform-*d*) δ: 7.58 (dd, *J* = 7.7, 1.4 Hz, 1H, Ar*H*), 7.34 (td, *J* = 7.5, 1.5 Hz, 1H, Ar*H*), 7.29 – 7.21 (m, 2H, Ar*H*), 2.49 (s, 3H, CC*H*$_3$), 2.27 (dd, *J* = 7.4, 5.6 Hz, 1H, C(O)C*H*), 1.51 (dd, *J* = 5.6, 4.0 Hz, 1H, CHC*H*$_2$), 1.33 (s, 3H, C(C*H*$_3$)$_2$), 1.18 (s, 3H, C(C*H*$_3$)$_2$), 0.95 (dd, *J* = 7.5, 4.0 Hz, 1H, CHC*H*$_2$).

^{13}C NMR (101 MHz, Chloroform-*d*) δ: 203 (*C*=O), 140.7 (Ar*C*^{q}), 137.2 (Ar*C*^{q}), 131.7 (Ar*C*H), 130.8 (Ar*C*H), 128.6 (Ar*C*H), 125.9 (Ar*C*H), 36.2 (C(O)*C*H), 28 (CH*C*(CH$_3$)$_2$), 27.3 (C(*C*H$_3$)$_2$), 23 (CH*C*H$_2$), 20.8 (C(*C*H$_3$)$_2$), 18.6 (*o*-C*C*H$_3$).

IR (neat, cm^{-1}): 2947, 2927, 1669, 1456, 1433, 1382, 1214, 1115, 1087, 1036, 995, 912, 827, 741, 638.

HRMS (ESI$^+$): calculated for $C_{13}H_{16}ONa$ (M + Na$^+$): 211.1093 Found: 211.1089

(2,2-Dimethylcyclopropyl)(2,6-dimethylphenyl)methanone (**1c**)

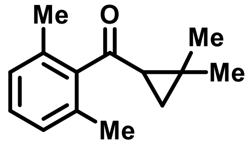

Yield: 45% over 3 steps (using Ox-2)

^{1}H NMR (400 MHz, Chloroform-*d*) δ 7.15 (t, *J* = 7.6 Hz, 1H, Ar*H*), 7.02 (d, *J* = 7.6 Hz, 2H, Ar*H*), 2.27 (s, 6H, 2 × CCH_3), 2.12 – 2.03 (m, 1H, C(O)C*H*), 1.50 (dd, *J* = 5.5, 3.7 Hz, 1H, CHCH_2), 1.36 (s, 3H, $C(CH_3)_2$), 1.23 (s, 3H, $C(CH_3)_2$), 1.09 (dd, *J* = 7.8, 3.7 Hz, 1H, CHCH_2).

^{13}C NMR (101 MHz, Chloroform-*d*) δ: 208.7 (*C*=O), 144.1 (ArC^q), 133.1 (2 × ArC^q), 128.5 (Ar*C*H), 127.9 (2 × Ar*C*H), 37.1 (C(O)*C*H), 29.8 ($CHC(CH_3)_2$), 27.5 ($C(CH_3)_2$), 26.1 ($CHCH_2$), 19.4 ($C(CH_3)_2$), 18.4 (2 × CCH_3).

IR (neat, cm^{-1}): 2949, 2925, 1674, 1460, 1427, 1378, 1207, 1115, 1094, 1037, 995, 812, 768, 715.

HRMS (ESI$^+$): calculated for $C_{14}H_{18}ONa$ (M + Na^+): 225.1250 Found: 225.1245

Cyclohexyl(2,2-dimethylcyclopropyl)methanone (**1d**)

Yield: 29% over 3 steps (using Ox-1)

^{1}H NMR (400 MHz, Chloroform-*d*) δ 2.41 (tt, *J* = 11.3, 3.3 Hz, 1H, cyclohexyl C*H*), 1.97 – 1.62 (m, 7H, cyclohexyl C*H* + C(O)C*H*), 1.45 – 1.20 (m, 5H, cyclohexyl C*H* + CHCH_2), 1.19 (s, 3H, $C(CH_3)_2$), 1.01 (s, 3H, $C(CH_3)_2$), 0.76 (dd, *J* = 7.5, 3.9 Hz, 1H, CHCH_2).

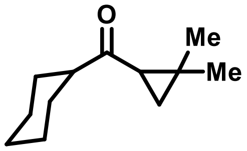

^{13}C NMR (101 MHz, Chloroform-*d*) δ 211.7 (*C*=O), 52.3 (cyclohexyl *C*H), 34.3 (C(O)*C*H), 28.6 (CH_2), 28.1 (CH_2), 27.3 ($CHC(CH_3)_2$), 26.6 ($CHC(CH_3)_2$), 26.2 (cyclohexyl CH_2), 26.2 (cyclohexyl CH_2), 25.7 (cyclohexyl CH_2), 22.6 (cyclohexyl CH_2), 18.6 ($C(CH_3)_2$).

HRMS (ESI$^+$): calculated for $C_{12}H_{21}O$ (M + H^+): 181.1587 Found: 181.1583

(2,2-Dimethylcyclopropyl)(2-ethylphenyl)methanone (**1e**)

Yield: 51% over 3 steps (using Ox-1)

^{1}H NMR (400 MHz, Chloroform-*d*) δ: 7.53 (dd, *J* = 8.1, 1.3 Hz, 1H, Ar*H*), 7.42 – 7.36 (m, 1H, Ar*H*), 7.32 – 7.27 (m, 2H, Ar*H*), 2.95 – 2.85 (m, 1H, CH_3CH_2), 2.85 – 2.76 (m, 1H, CH_3CH_2), 2.28 (dd, *J* = 7.5, 5.6 Hz, 1H, C(O)C*H*), 1.53 (dd, *J* = 5.6, 4.0 Hz, 1H, CHCH_2), 1.34 (s, 3H, $C(CH_3)_2$), 1.25 (t, *J* = 7.5 Hz, 3H, CH_3CH_2), 1.22 (s, 3H, $C(CH_3)_2$), 0.99 (dd, *J* = 7.5, 4.0 Hz, 1H, CHCH_2).

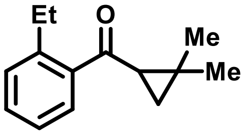

^{13}C NMR (101 MHz, Chloroform-*d*) δ: 203.5 (*C*=O), 143.2 (ArC^q), 140.9 (ArC^q), 130.8 (Ar*C*H), 130.1 (Ar*C*H), 128.3 (Ar*C*H), 125.8 (Ar*C*H), 36.4 (C(O)*C*H), 28.3 ($C(CH_3)_2$), 27.3 ($C(CH_3)_2$), 26.6 (CH_3CH_2), 23.5 ($CHCH_2$), 18.5 ($CHC(CH_3)_2$), 16.4 (CH_3CH_2).

HRMS (ESI$^+$): calculated for $C_{14}H_{18}ONa$ (M + Na^+): 225.1250 Found: 225.1246

(2,2-Dimethylcyclopropyl)(3-fluoro-2-methylphenyl)methanone (**1f**)

Yield: 34% over 3 steps (using Ox-1)

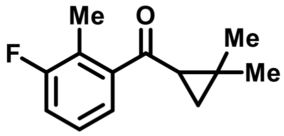

^{1}H NMR (400 MHz, Chloroform-*d*) δ 7.38 (d, *J* = 7.6 Hz, 1H, Ar*H*), 7.33 – 7.27 (m, 1H, Ar*H*), 7.17 (t, *J* = 8.8 Hz, 1H, Ar*H*), 2.43 (d, *J* = 2.4 Hz, 3H, CCH_3), 2.30 (dd, *J* = 7.5, 5.6 Hz, 1H, C(O)C*H*), 1.58 (dd, *J* = 5.6, 4.1 Hz, 1H, CHCH_2), 1.39 (s, 3H, C$(CH_3)_2$), 1.25 (s, 3H, C$(CH_3)_2$), 1.05 (dd, *J* = 7.5, 4.0 Hz, 1H, CHCH_2).

^{13}C NMR (101 MHz, Chloroform-d) δ: 202.2 (d, *J* = 3.03 Hz, *C*=O), 161.7 (d, *J* = 246.44 Hz, ArC^q–F), 143.2 (d, *J* = 4.04 Hz, ArC^q), 126.9 (d, *J* = 8.08 Hz, Ar*C*H), 124.1 (d, *J* = 17.17 Hz, ArC^q), 123.8 (d, *J* = 4.04 Hz, Ar*C*H), 117.4 (d, *J* = 24.24 Hz, Ar*C*H), 36.5 (C(O)*C*H), 28.7 (CH*C*$(CH_3)_2$), 27.2 (C$(CH_3)_2$), 23.6 (CHCH_2), 18.5 (C$(CH_3)_2$), 11.6 (d, *J* = 6.06 Hz, CCH_3).

^{19}F NMR (376 MHz, Chloroform-*d*) δ: –115.7 (s).

HRMS (ESI$^+$): calculated for $C_{13}H_{15}OFNa$ (M + Na^+): 229.0999 Found: 229.0996

(2,2-Dimethylcyclopropyl)(2,3-dimethylphenyl)methanone (**1g**)

Yield: 33% over 3 steps (using Ox-2)

^{1}H NMR (400 MHz, Chloroform-*d*) δ: 7.32 (d, *J* = 7.6 Hz, 1H, Ar*H*), 7.23 (d, *J* = 7.5 Hz, 1H, Ar*H*), 7.16 (t, *J* = 7.5 Hz, 1H, Ar*H*), 2.34 (s, 3H, CCH_3), 2.31 (s, 3H, CCH_3), 2.24 (dd, *J* = 7.4, 5.8 Hz, 1H, C(O)C*H*), 1.51 (dd, *J* = 5.6, 4.0 Hz, 1H, CHCH_2), 1.31 (s, 3H, C$(CH_3)_2$), 1.21 (s, 3H, C$(CH_3)_2$), 0.97 (dd, *J* = 7.5, 4.0 Hz, 1H, CHCH_2)

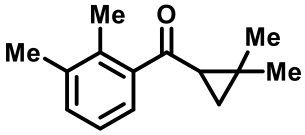

^{13}C NMR (101 MHz, Chloroform-d) δ: 204.5 (*C*=O), 142.3 (ArC^q), 138.2 (ArC^q), 134.7 (ArC^q), 132 (Ar*C*H), 125.6 (Ar*C*H), 125.5 (Ar*C*H), 36.8 (C(O)*C*H), 28.4 (CH*C*$(CH_3)_2$), 27.3 (C$(CH_3)_2$), 23.7 (CHCH_2), 20.5 (C$(CH_3)_2$), 18.5 (*m*-CCH_3), 16.5 (*o*-CCH_3).

IR (neat, cm^{-1}): 2946, 1668, 1456, 1430, 1382, 1269, 1231, 1114, 1091, 989, 839, 770, 753, 718.

HRMS (ESI$^+$): calculated for $C_{14}H_{19}O$ (M + H^+): 203.1430 Found: 203.1429

(2-Chloro-6-methylphenyl)(2,2-dimethylcyclopropyl)methanone (**1h**)

Yield: 41% over 3 steps (using Ox-1)

^{1}H NMR (500 MHz, Chloroform-*d*) δ: 7.22 – 7.15 (m, 2H, Ar*H*), 7.09 (d, *J* = 7.3 Hz, 1H, Ar*H*), 2.26 (d, *J* = 1.5 Hz, 3H, CCH_3), 2.11 (dd, *J* = 7.8, 5.7 Hz, 1H, C(O)C*H*), 1.52 (t, *J* = 4.7 Hz, 1H, CHCH_2), 1.35 (s, 3H, C$(CH_3)_2$), 1.24 (s, 3H, C$(CH_3)_2$), 1.12 (dd, *J* = 7.8, 3.6 Hz, 1H, CHCH_2).

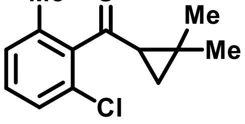

^{13}C NMR (101 MHz, Chloroform-*d*) δ: 204.5 (*C*=O), 142.5 (ArC^q), 136 (ArC^q), 129.7(ArC^q–Cl), 129.6 (Ar*C*H), 129 (Ar*C*H), 127.1 (Ar*C*H), 37.2 (C(O)*C*H), 30.6 (CH*C*$(CH_3)_2$), 27.4 (C$(CH_3)_2$), 26.3 (CHCH_2), 19.4 (C$(CH_3)_2$), 18.4 (CCH_3).

HRMS (ESI$^+$): calculated for $C_{13}H_{15}OClNa$ (M + Na^+): 245.0704 Found: 245.0700

(2,2-Dimethylcyclopropyl)(3,5-dimethylphenyl)methanone (**1i**)

Yield: 35% over 3 steps (using Ox-2)

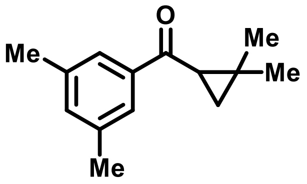

^{1}H NMR (400 MHz, Chloroform-*d*) δ: 7.54 (s, 2H, Ar*H*), 7.20 – 7.17 (m, 1H, Ar*H*), 2.46 (dd, *J* = 7.5, 5.6 Hz, 1H, C(O)C*H*), 2.38 (s, 6H, 2 × CCH_3), 1.50 (dd, *J* = 5.6, 4.0 Hz, 1H, CHCH_2), 1.36 (s, 3H, C(CH_3)$_2$), 1.08 (s, 3H, C(CH_3)$_2$), 0.93 (dd, *J* = 7.5, 4.0 Hz, 1H, CHCH_2).

^{13}C NMR (101 MHz, Chloroform-*d*) δ: 199.2 (*C*=O), 139.4 (ArC^q), 138.3 (Ar*C*H), 134.3 (2 × ArC^q), 126.0 (2 × Ar*C*H), 33.1 (C(O)*C*H), 27.3 (C(*C*H_3)$_2$), 27.1 (C(*C*H_3)$_2$), 22.2 (CH*C*H_2), 21.5 (CH*C*(CH$_3$)$_2$), 18.7 (2 × C*C*H_3).

HRMS (ESI$^+$): calculated for $C_{14}H_{18}ONa$ (M + Na$^+$): 225.12 Found: 225.1243

(2,2-Dimethylcyclopropyl)(mesityl)methanone (**1j**)

Yield: 37% over 3 steps (using Ox-1)

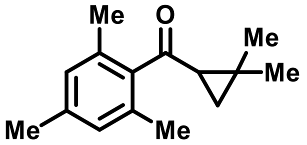

^{1}H NMR (400 MHz, Chloroform-*d*) δ: 6.84 (s, 2H, Ar*H*), 2.28 (s, 3H, CCH_3), 2.24 (s, 6H, 2 × CCH_3), 2.08 (dd, *J* = 7.7, 5.5 Hz, 1H, C(O)C*H*), 1.48 (dd, *J* = 5.5, 3.7 Hz, 1H, CHCH_2), 1.34 (s, 3H, C(CH_3)$_2$), 1.22 (s, 3H, C(CH_3)$_2$), 1.06 (dd, *J* = 7.7, 3.7 Hz, 1H, CHCH_2).

^{13}C NMR (101 MHz, Chloroform-*d*) δ: 208.7 (*C*=O), 141.4 (ArC^q), 138.3 (ArC^q), 133.3 (2 × ArC^q), 128.7 (2 × Ar*C*H), 37.3 (C(O)*C*H), 29.6 (CH*C*H_2), 27.5 (C(*C*H_3)$_2$), 26.0 (C(*C*H_3)$_2$), 21.3 (CH*C*(CH$_3$)$_2$), 19.4 (2 × C*C*H_3), 18.4 (C*C*H_3).

IR (neat, cm^{-1}): 2948, 2921, 1673, 1610, 1430, 1376, 1215, 1155, 1115, 1084, 1037, 994, 895, 849, 814, 753.

HRMS (ESI$^+$): calculated for $C_{15}H_{20}ONa$ (M + Na$^+$): 239.1406 Found: 239.1399

(2,2-Dimethylcyclopropyl)(2,3,4,5,6-pentamethylphenyl)methanone (**1k**)

Yield: 53% over 3 steps (using Ox-2)

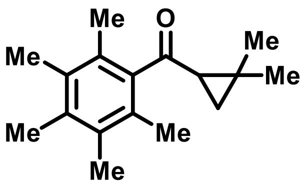

^{1}H NMR (400 MHz, Chloroform-*d*) δ: 2.24 (s, 3H, CCH_3), 2.20 (s, 6H, 2 × CCH_3), 2.15 (s, 6H, 2 × CCH_3), 2.08 (dd, *J* = 7.8, 5.5 Hz, 1H, C(O)C*H*), 1.48 (dd, *J* = 5.5, 3.6 Hz, 1H, CHCH_2), 1.39 (s, 3H, C(CH_3)$_2$), 1.19 (s, 3H, C(CH_3)$_2$), 1.09 (dd, *J* = 7.8, 3.6 Hz, 1H, CHCH_2).

^{13}C NMR (101 MHz, Chloroform-*d*) δ: 210.4 (*C*=O), 142.9 (ArC^q), 135.3 (ArC^q), 133.7 (2 × ArC^q), 128.0 (2 × ArC^q), 37.6 (C(O)*C*H), 29.6 (CH*C*H_2), 27.6 (2 × C(*C*H_3)$_2$), 26.3 (CH*C*(CH$_3$)$_2$), 18.4 (2 × C*C*H_3), 16.9 (C*C*H_3), 16.2 (2 × C*C*H_3).

IR (neat, cm^{-1}): 2943, 1674, 1453, 1435, 1376, 1134, 1114, 1096, 1038, 996, 959, 947, 886, 805, 697.

HRMS (ESI$^+$): calculated for $C_{17}H_{25}O$ (M + H$^+$): 245.1900 Found: 245.1896

[1,1'-Biphenyl]-2-yl(2,2-dimethylcyclopropyl)methanone (**1l**)

Yield: 35% over 3 steps (using Ox-1)

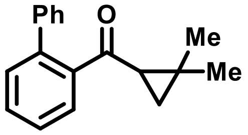

^{1}H NMR (400 MHz, Chloroform-*d*) δ: 7.55 – 7.47 (m, 2H, Ar*H*), 7.47 – 7.38 (m, 4H, Ar*H*), 7.38 – 7.33 (m, 3H, Ar*H*), 1.65 (dd, *J* = 7.7, 5.4 Hz, 1H, C(O)C*H*), 1.26 (dd, *J* = 5.5, 3.6 Hz, 1H, CHCH_2), 1.14 (s, 3H, C(CH_3)$_2$), 0.77 (dd, *J* = 7.8, 3.6 Hz, 1H, CHCH_2), 0.63 (s, 3H, C(CH_3)$_2$).

^{13}C NMR (101 MHz, Chloroform-*d*) δ: 205.3 (*C*=O), 142.5 (ArC^q), 141.2 (ArC^q), 140.7 (ArC^q), 130.6 (Ar*C*H), 130.5 (Ar*C*H), 129.5 (2 × Ar*C*H), 128.7 (2 × Ar*C*H), 128.2 (Ar*C*H), 127.7 (Ar*C*H), 127.6 (Ar*C*H), 37.4 (C(O)*C*H), 29.6 (CH*C*H_2), 26.8 (C(*C*H_3)$_2$), 26.7 (C(*C*H_3)$_2$), 17.6 (CH*C*(CH_3)$_2$).

HRMS (ESI$^+$): calculated for $C_{18}H_{18}ONa$ (M + Na$^+$): 273.1250 Found: 273.1241

(2,2-Dimethylcyclopropyl)(2-fluorophenyl)methanone (**1m**)

Yield: 43% over 3 steps (using Ox-1)

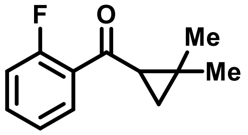

^{1}H NMR (400 MHz, Chloroform-*d*) δ: 7.72 (td, *J* = 7.6, 1.9 Hz, 1H, Ar*H*), 7.48 (dddd, *J* = 8.3, 7.1, 5.0, 1.9 Hz, 1H, Ar*H*), 7.21 (td, *J* = 7.5, 1.1 Hz, 1H, Ar*H*), 7.12 (ddd, *J* = 10.9, 8.3, 1.1 Hz, 1H, Ar*H*), 2.48 (ddd, *J* = 7.3, 5.7, 3.9 Hz, 1H, C(O)C*H*), 1.54 (ddd, *J* = 5.6, 4.1, 1.3 Hz, 1H, CHCH_2), 1.33 (s, 3H, C(CH_3)$_2$), 1.14 (s, 3H, C(CH_3)$_2$), 0.96 (dd, *J* = 7.3, 4.0 Hz, 1H, CHCH_2).

^{13}C NMR (101 MHz, Chloroform-*d*) δ: 197.4 (d, *J* = 3.03 Hz, *C*=O), 161.7 (d, *J* = 254.52 Hz, ArC^q–F), 133.8 (d, *J* = 8.08 Hz, Ar*C*H), 130.4 (d, *J* = 2.02 Hz, Ar*C*H), 128.5 (d, *J* = 13.13 Hz, ArC^q), 124.4 (d, *J* = 4.04 Hz, Ar*C*H), 116.7 (d, *J* = 23.23 Hz, Ar*C*H), 37.3 (d, *J* = 7.07 Hz, C(O)*C*H), 28.6 (CH*C*(CH_3)$_2$), 27.4 (C(*C*H_3)$_2$), 23.0 (CH*C*H_2), 18.6 (C(*C*H_3)$_2$).

^{19}F NMR (376 MHz, Chloroform-*d*) δ: –112.3 (s).

HRMS (ESI$^+$): calculated for $C_{12}H_{13}OFNa$ (M + Na$^+$): 215.0843 Found: 215.0839

(2,2-Dimethylcyclopropyl)(2-iodophenyl)methanone (**1n**)

Yield: 57% over 3 steps (using Ox-1)

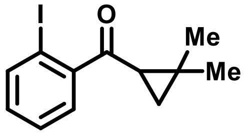

^{1}H NMR (400 MHz, Chloroform-*d*) δ: 7.91 (dd, *J* = 7.7, 0.9 Hz, 1H, Ar*H*), 7.46 – 7.35 (m, 2H, Ar*H*), 7.10 (ddd, *J* = 7.9, 6.2, 2.9 Hz, 1H, Ar*H*), 2.25 (dd, *J* = 7.5, 5.5 Hz, 1H, C(O)C*H*), 1.55 (dd, *J* = 5.5, 4.0 Hz, 1H, CHCH_2), 1.31 (s, 3H, C(CH_3)$_2$), 1.26 (s, 3H, C(CH_3)$_2$), 1.05 (dd, *J* = 7.5, 3.9 Hz, 1H, CHCH_2).

^{13}C NMR (101 MHz, Chloroform-*d*) δ: 202.8 (*C*=O), 146.2 (Ar*C^q*), 140.6 (Ar*C*H), 131.6 (Ar*C*H), 128.7 (Ar*C*H), 128.3 (Ar*C*H), 91.4 (Ar*C^q*–I), 36.0 (C(O)*C*H), 30.0 (CH*C*H$_2$), 27.3 (C(*C*H$_3$)$_2$), 25.1 (C(*C*H$_3$)$_2$), 18.6 (CH*C*(CH$_3$)$_2$).

HRMS (ESI$^+$): calculated for $C_{12}H_{13}OINa$ (M + Na$^+$): 322.9903 Found: 322.9894

Spiro[2.5]octan-1-yl(o-tolyl)methanone (**1o**)

Yield: 30% over 2 steps

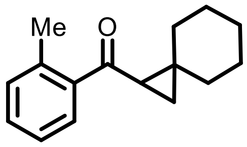

^{1}H NMR (400 MHz, Chloroform-*d*) δ: 7.71 (dd, *J* = 7.6, 1.5 Hz, 1H, Ar*H*), 7.34 (td, *J* = 7.5, 1.5 Hz, 1H, Ar*H*), 7.31 – 7.19 (m, 2H, Ar*H*), 2.49 (s, 3H, CC*H$_3$*), 2.28 (dd, *J* = 7.4, 5.5 Hz, 1H, C(O)C*H*), 1.68 – 1.54 (m, 6H, cyclohexyl C*H*), 1.47 (ddd, *J* = 14.7, 7.6, 5.3 Hz, 5H, cyclohexyl C*H* + CHC*H$_2$*), 0.94 (dd, *J* = 7.4, 3.9 Hz, 1H, CHC*H$_2$*).

^{13}C NMR (101 MHz, Chloroform-*d*) δ 202.6 (*C*=O), 140.6 (Ar*C^q*), 137.3 (Ar*C^q*), 131.8 (Ar*C*H), 130.8 (Ar*C*H), 128.8 (Ar*C*H), 125.8 (Ar*C*H), 38 (cyclohexyl *C*H$_2$), 36 (CH$_2$*C^q*), 35.4 (C(O)*C*H), 28.4 (cyclohexyl *C*H$_2$), 26.4 (cyclohexyl *C*H$_2$), 26.2 (cyclohexyl *C*H$_2$), 26.1 (cyclohexyl *C*H$_2$), 22.3 (CH*C*H$_2$), 21 (C*C*H$_3$).

IR (neat, cm^{-1}): 2922, 2850, 1666, 1445, 1392, 1213, 1026, 977, 738.

HRMS (ESI$^+$): calculated for $C_{16}H_{21}O$ (M + H$^+$): 229.1587 Found: 229.1585

Spiro[2.3]hexan-1-yl(o-tolyl)methanone (**1p**)

Yield: 28% over 2 steps

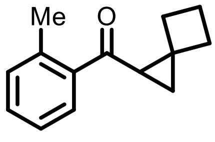

^{1}H NMR (500 MHz, Chloroform-*d*) δ 7.65 (dd, *J* = 7.6, 1.5 Hz, 1H, Ar*H*), 7.36 (td, *J* = 7.5, 1.5 Hz, 1H, Ar*H*), 7.28 (td, *J* = 7.6, 1.3 Hz, 1H, Ar*H*), 7.24 (d, *J* = 7.6 Hz, 1H, Ar*H*), 2.50 (s, 3H, CC*H$_3$*), 2.42 (dd, *J* = 7.9, 5.4 Hz, 1H, C(O)C*H*), 2.39 – 2.29 (m, 1H, cyclobutyl C*H*), 2.29 – 2.16 (m, 3H, cyclobutyl C*H*), 2.15 – 2.08 (m, 1H, cyclobutyl C*H*), 2.01 (dtt, *J* = 11.3, 9.5, 5.5 Hz, 1H cyclobutyl C*H*), 1.63 – 1.54 (m, 1H, CHC*H$_2$*), 1.19 (dd, *J* = 7.9, 4.1 Hz, 1H, CHC*H$_2$*).

^{13}C NMR (126 MHz, Chloroform-*d*) δ: 203 (*C*=O), 140.7 (Ar*C^q*), 137 (Ar*C^q*), 131.7 (Ar*C*H), 130.8 (Ar*C*H), 128.3 (Ar*C*H), 125.9 (Ar*C*H), 35.8 (CH$_2$*C^q*), 34.2 (C(O)*C*H), 31.1 (cyclobutyl *C*H$_2$), 27.9 (cyclobutyl *C*H$_2$), 23.5 (CH*C*H$_2$), 20.8 (C*C*H$_3$) , 16.9 (cyclobutyl *C*H$_2$).

IR (neat, cm^{-1}): 2928, 1665, 1382, 1315, 1216, 1194, 1105, 1047, 1021, 961, 829, 735, 649.

HRMS (ESI$^+$): calculated for $C_{14}H_{17}O$ (M + H$^+$): 201.1274 Found: 201.1274

(2,2-Dimethylcyclopropyl)(o-tolyl)methanone (**1q**)

Yield: 42% over 3 steps (using Ox-2)

^{1}H NMR (400 MHz, Chloroform-*d*) δ 7.66 (dd, *J* = 7.6, 1.4 Hz, 1H, Ar*H*), 7.37 (td, *J* = 7.4, 1.5 Hz, 1H, Ar*H*), 7.31 – 7.22 (m, 2H, Ar*H*), 2.49 (s, 3H, CCH_3), 2.17 (dt, *J* = 8.1, 4.3 Hz, 1H, C(O)C*H*), 1.62 (dq, *J* = 9.8, 3.8 Hz, 1H, $CHCH_3$), 1.51 (ddd, *J* = 8.4, 4.6, 3.5 Hz, 1H, $CHCH_2$), 1.23 (d, *J* = 6.0 Hz, 3H, $CHCH_3$), 0.90 (ddd, *J* = 7.6, 6.4, 3.4 Hz, 1H, $CHCH_2$).

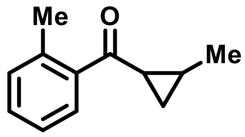

^{13}C NMR (101 MHz, Chloroform-*d*) δ: 204.9 (*C*=O), 140.2 (ArC^q), 136.9 (ArC^q), 131.6 (Ar*C*H), 130.8 (Ar*C*H), 128.4 (Ar*C*H), 125.8 (Ar*C*H), 30.3 (C(O)*C*H), 21.8 ($CHCH_3$), 20.9 ($CHCH_3$), 20.5 ($CHCH_2$), 18.4 (CCH_3).

HRMS (ESI$^+$): calculated for $C_{12}H_{14}ONa$ (M + Na^+): 197.0937 Found: 197.0934

Cyclopropyl(2,6-dimethylphenyl)methanone (**1r**)

Title compound was prepared from vinylmagnesium bromide and 2,6-dimethylbenzaldehyde.

Yield: 39% over 3 steps (using Ox-2)

^{1}H NMR (400 MHz, Chloroform-*d*) δ 7.16 (dd, *J* = 8.1, 7.1 Hz, 1H, Ar*H*), 7.03 (d, *J* = 7.6 Hz, 2H, Ar*H*), 2.29 (s, 6H, CCH_3), 2.19 (tt, *J* = 7.7, 4.6 Hz, 1H, C(O)C*H*), 1.29 (dt, *J* = 4.5, 3.4 Hz, 2H, CH_2), 1.07 (dq, *J* = 7.4, 3.6 Hz, 2H, CH_2).

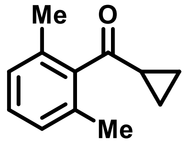

^{13}C NMR (101 MHz, Chloroform-*d*) δ 210.6 (*C*=O), 142.9 (ArC^q), 133.1 (2 × ArC^q), 128.72 (Ar*C*H), 127.9 (2 × Ar*C*H), 23.2 (C(O)*C*H), 19.7 (2 × CCH_3), 12.7 (2 × CH_2).

HRMS (ESI$^+$): calculated for $C_{12}H_{15}O$ (M + H^+): 175.1117 Found: 175.1114

(2-Phenylcyclopropyl)(o-tolyl)methanone (**1s**)

Yield: 58% over 2 steps

^{1}H NMR (400 MHz, Chloroform-*d*) δ: 7.67 (dd, *J* = 8.0, 1.5 Hz, 1H, Ar*H*), 7.41 – 7.29 (m, 3H, Ar*H*), 7.28 – 7.20 (m, 3H, Ar*H*), 7.19 – 7.14 (m, 2H, Ar*H*), 2.79 – 2.64 (m, 2H, C(O)C*H and* C*H*Ph), 2.49 (s, 3H, CCH_3), 1.93 (ddd, *J* = 8.4, 5.9, 4.1 Hz, 1H, $CHCH_2$), 1.60 – 1.49 (m, 1H, $CHCH_2$).

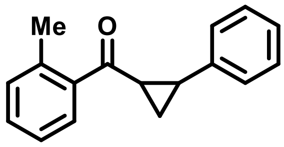

^{13}C NMR (101 MHz, Chloroform-*d*) δ: 202.9 (*C*=O), 140.6 (ArC^q), 139.5 (ArC^q), 137.4 (ArC^q), 131.8 (Ar*C*H), 131.2 (Ar*C*H), 128.8 (2 × Ar*C*H), 128.6 (Ar*C*H), 126.8 (Ar*C*H), 126.3 (2 × Ar*C*H), 125.9 (Ar*C*H), 32.9 (C(O)*C*H), 30.5 (*C*HPh), 21.0 (CCH_3), 19.8 ($CHCH_2$).

IR (neat, cm^{-1}): 1666, 1454, 1392, 1337, 1215, 1026, 981, 739, 725, 695, 648.

HRMS (ESI$^+$): calculated for $C_{17}H_{16}ONa$ (M + H^+): 259.1093 Found: 259.1095

1-(2,2-Dimethylcyclopropyl)-2-ethylbutan-1-one (**1t**)

Yield: 47% over 3 steps

^{1}H NMR (400 MHz, Chloroform-*d*) δ: 2.43 – 2.35 (m, 1H, C(O)C*H*$(C_2H_5)_2$), 1.85 (dd, *J* = 7.6, 5.6 Hz, 1H, C(O)C*H*), 1.72 – 1.59 (m, 2H, 2 × C*H*$_2$CH$_3$), 1.53 – 1.44 (m, 2H, 2 × C*H*$_2$CH$_3$), 1.25 (dd, *J* = 5.6, 3.7 Hz, 1H, CHC*H*$_2$), 1.19 (s, 3H, C(C*H*$_3$)$_2$), 1.10 (s, 3H, C(C*H*$_3$)$_2$), 0.87 (td, *J* = 7.5, 6.0 Hz, 6H, 2 × CH$_2$C*H*$_3$), 0.82 (dd, *J* = 7.6, 3.8 Hz, 1H, CHC*H*$_2$).

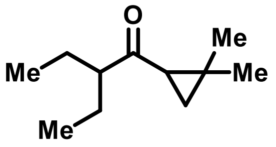

^{13}C NMR (101 MHz, Chloroform-*d*) δ: 212.6 (C=O), 57.5 (COCH$(C_2H_5)_2$), 34.5 (COCH), 27.5 (C(CH$_3$)$_2$), 27.2 (*C*(CH$_3$)$_2$), 24.5 (*C*H$_2$), 24.2 (*C*H$_2$), 23.5 (*C*H$_2$), 18.5 (C(*C*H$_3$)$_2$), 12.2 (CH$_2$*C*H$_3$), 12.1 (CH$_2$*C*H$_3$).

IR (neat, cm^{-1}): 2961, 2929, 2874, 1687, 1459, 1392, 1377, 1117, 1096, 1022.

1-(2,2-Dimethylcyclopropyl)-3-phenylpropan-1-one (**1u**)

Yield: 38% over 3 steps

^{1}H NMR (400 MHz, Chloroform-*d*) δ: 7.32 – 7.25 (m, 2H, Ar*H*), 7.22 – 7.16 (m, 3H, Ar*H*), 2.95 – 2.90 (m, 2H, C(O)C*H*$_2$), 2.87 – 2.82 (m, 2H, C(O)CH$_2$C*H*$_2$), 1.83 (dd, *J* = 7.6, 5.6 Hz, 1H, C(O)C*H*), 1.27 (dd, *J* = 5.6, 3.8 Hz, 1H, CHC*H*$_2$), 1.17 (s, 3H, C(C*H*$_3$)$_2$), 1.04 (s, 3H, C(C*H*$_3$)$_2$), 0.82 (dd, *J* = 7.6, 3.9 Hz, 1H, CHC*H*$_2$).

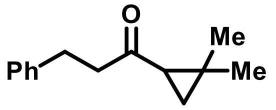

^{13}C NMR (101 MHz, Chloroform-*d*) δ: 208.0 (*C*=O), 141.5 (Ar*C*^{q}), 128.6 (2 × Ar*C*H), 128.5 (2 × Ar*C*H), 126.2 (ArCH), 46.6 (CO*C*H$_2$), 35.4 (CO*C*H), 30.2 (COCH$_2$*C*H$_2$), 27.5 (C(*C*H$_3$)$_2$), 27.0 (*C*(CH$_3$)$_2$), 23.4 (C(O)CH*C*H$_2$), 18.4 (C(*C*H$_3$)$_2$).

IR (neat, cm^{-1}): 1692, 1453, 1434, 1393, 1375, 1112, 1057, 1030, 1009, 819, 747, 698.

HRMS (ESI$^+$): calculated for $C_{14}H_{19}O$ (M + H$^+$): 203.1430 Found: 203.1436

1-(2,2-Dimethylcyclopropyl)-3-methylbutan-1-one (**1v**)

Yield: 54% over 3 steps

^{1}H NMR (400 MHz, Chloroform-*d*) δ: 2.44 – 2.29 (m, 2H, C(O)C*H*$_2$), 2.21 – 2.11 (m, 1H, C*H*(CH$_3$)$_2$), 1.82 (dd, *J* = 7.6, 5.5 Hz, 1H, C(O)C*H*), 1.24 (dd, *J* = 5.6, 3.9 Hz, 1H, CHC*H*$_2$), 1.19 (s, 3H, C(CH$_3$)$_2$), 1.08 (s, 3H, C(CH$_3$)$_2$), 0.93 (d, *J* = 6.6 Hz, 3H, CH(C*H*$_3$)$_2$), 0.91 (d, *J* = 6.6 Hz, 3H, CH(C*H*$_3$)$_2$), 0.79 (dd, *J* = 7.6, 3.9 Hz, 1H, CHC*H*$_2$).

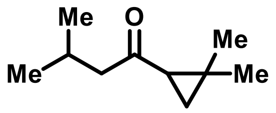

^{13}C NMR (101 MHz, Chloroform-*d*) δ: 209.1 (*C*=O), 54.5 (C(O)*C*H$_2$), 35.6 (C(O)*C*H), 27.5 (C(*C*H$_3$)$_2$), 26.8 (*C*(CH$_3$)$_2$), 25.3 (*C*H(CH$_3$)$_2$), 23.2 (C(O)CH*C*H$_2$), 23.0 (CH(*C*H$_3$)$_2$), 22.8 (CH(*C*H$_3$)$_2$), 18.4 (C(*C*H$_3$)$_2$).

IR (neat, cm^{-1}): 2954, 2871, 1691, 1465, 1434, 1392, 1376, 1147, 1095, 1032, 826.

HRMS (ESI$^+$): calculated for $C_{10}H_{19}O$ (M + H$^+$): 155.1430 Found: 155.1431

1-(2,2-Dimethylcyclopropyl)ethan-1-one (**1w**)[2]

Yield: quantitative

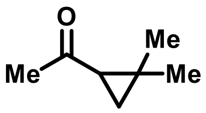

^{1}H NMR (400 MHz, Chloroform-*d*) δ: 2.20 (s, 3H, COC*H*$_3$), 1.83 (dd, *J* = 7.6, 5.5 Hz, 1H, C(O)C*H*), 1.20 (dd, *J* = 5.7, 4.1 Hz, 1H CHC*H*$_2$), 1.17 (s, 3H, C(C*H*$_3$)$_2$), 1.06 (s, 3H, C(C*H*$_3$)$_2$), 0.78 (dd, *J* = 7.6, 4.0 Hz, 1H CHC*H*$_2$). **^{13}C NMR** (101 MHz, Chloroform-*d*) δ: 206.8 (*C*=O), 35.8 (COC*H*), 32.4 (CO*C*H$_3$), 27.4 (C(*C*H$_3$)$_2$), 26.8 (*C*(CH$_3$)$_2$), 23.3 (CH*C*H$_2$), 18.2 (C(*C*H$_3$)$_2$).

IR (neat, cm^{-1}): 1693, 1391, 1376, 1351, 1176, 1093, 971, 945, 826.

4. Catalytic Intermolecular Couplings

4a. General procedure A for the Intermolecular Coupling Reactions:

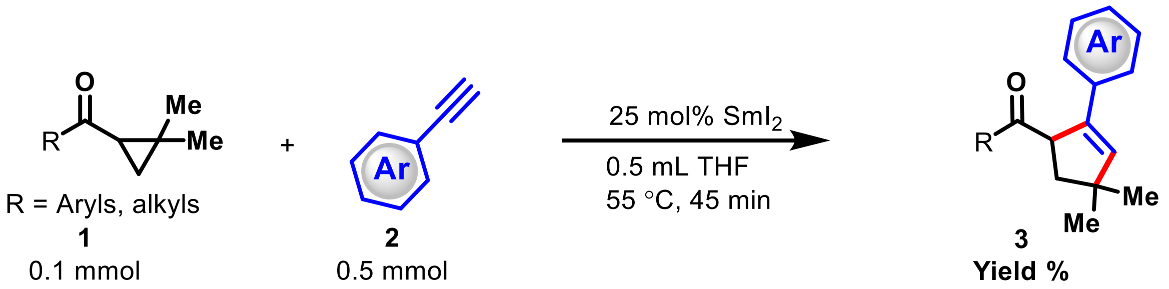

To a clean, oven–dried microwave reaction vial containing a magnetic stir–bar, ketone **1** (0.1 mmol, 1 eq.) was added and the vial flushed with N_2. After 15 min, THF (0.5 mL) and alkyne **2** (0.25 or 0.5 mmol) were introduced by syringe. The vial was placed in a preheated oil bath at 55 °C, followed by the addition of freshly prepared SmI_2 (typically 25 mol%, 0.1 M, 0.250 mL). The reaction was stirred (at 400 rpm) vigorously for 45 min. The reaction mixture was cooled to room temperature and filtered through a silica gel pad (100-200 mesh size) using CH_2Cl_2 (15 mL). Excess solvent was removed in vacuo and the desired compound was obtained without further purification. In a few cases, the final product was purified by column chromatography using silica gel (100-200 mesh size) and hexane/ethyl acetate as eluent.

4b. Characterization Data of the Coupling Products:

rac-(4,4-Dimethyl-2-phenylcyclopent-2-en-1-yl)(phenyl)methanone (**3a**)

Prepared by following the **general procedure A** from 0.1 mmol (**1a**), 0.5 mmol (**2a**), and SmI_2 (25 mol%). The reaction mixture was filtered through a silica gel pad and concentrated in vacuo. The crude product was purified by column chromatography (silica gel 100-200 mesh size; hexane/ethyl acetate) to afford **3a** (9.6 mg, 0.035 mmol, 35% yield) as a brown solid.

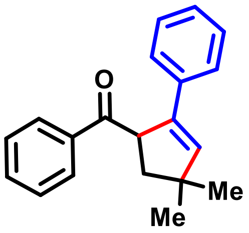

^{1}H NMR (400 MHz, Chloroform-*d*) δ: 8.07 (d, J = 7.9 Hz, 2H, Ar*H*), 7.61 (t, J = 7.3 Hz, 1H, Ar*H*), 7.51 (t, J = 7.5 Hz, 2H, Ar*H*), 7.30 (d, J = 7.9 Hz, 2H, Ar*H*), 7.24 (t, J = 7.4 Hz, 2H, Ar*H*), 7.17 (t, J = 7.1 Hz, 1H, Ar*H*), 6.26 (s, 1H, C*H*C(CH_3)$_2$), 5.04 (dd, J = 10.2, 5.4 Hz, 1H, C(O)C*H*), 2.46 – 2.37 (m, 1H, CHCH_2), 1.97 (dd, J = 13.0, 5.4 Hz, 1H, CHCH_2), 1.23 (s, 3H, CH_2C(CH_3)$_2$), 1.20 (s, 3H, CH_2C(CH_3)$_2$).

^{13}C NMR (101 MHz, Chloroform-*d*) δ: 201.7 (*C*=O), 140.3 (C(CH_3)$_2$CH), 138.5 (ArC^q), 136.7 (ArC^q), 135.8 (CHC^q–Ar), 133.3 (Ar*C*H), 128.9 (4 × Ar*C*H), 128.6 (2 × Ar*C*H), 127.3 (Ar*C*H), 126.0 (2 × Ar*C*H), 53.6 (C(O)*C*H), 45.9 (CH*C*H_2), 44.9 (CH*C*(CH_3)$_2$), 29.6 (CH_2C(*C*H_3)$_2$), 29.0 (CH_2C(*C*H_3)$_2$).

IR (neat, cm^{-1}): 2952, 2927, 1680, 1595, 1446, 1326, 1205, 1178, 1011, 767, 749, 704, 690.

HRMS (ESI$^+$): calculated for $C_{20}H_{21}O$ (M + H^+): 277.1587 Found: 277.1585

rac-(4,4-Dimethyl-2-phenylcyclopent-2-en-1-yl)(o-tolyl)methanone (**3b**)

Prepared by following the **general procedure A** from 0.1 mmol (**1b**), 0.5 mmol (**2a**), and SmI_2 (25 mol%). The reaction mixture was filtered through a silica gel pad and concentrated in vacuo to afford the final product (28.8 mg, 0.099 mmol, 99% yield) as a viscous liquid.

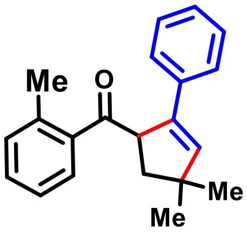

^{1}H NMR (400 MHz, Chloroform-*d*) δ 7.78 (dd, *J* = 7.7, 1.3 Hz, 1H, Ar*H*), 7.42 (td, *J* = 7.5, 1.4 Hz, 1H, Ar*H*), 7.33 (ddt, *J* = 8.4, 3.4, 1.7 Hz, 3H, Ar*H*), 7.30 – 7.24 (m, 3H, Ar*H*), 7.22 – 7.17 (m, 1H, Ar*H*), 6.24 (d, *J* = 1.5 Hz, 1H, C*H*C(CH_3)$_2$), 4.97 (ddd, *J* = 9.9, 5.3, 1.6 Hz, 1H, C(O)C*H*), 2.40 (s, 3H, CC*H$_3$*), 2.34 (dd, *J* = 13.0, 9.9 Hz, 1H, CHC*H$_2$*), 2.01 (dd, *J* = 13.0, 5.3 Hz, 1H, CHC*H$_2$*), 1.24 (s, 3H, CH$_2$C(C*H$_3$*)$_2$), 1.23 (s, 3H, CH$_2$C(C*H$_3$*)$_2$).

^{13}C NMR (101 MHz, Chloroform-*d*) δ: 205.9 (*C*=O), 140.4 (C(CH_3)$_2$*C*H), 139.0 (Ar*C^q*), 138.9 (Ar*C^q*), 138.2 (Ar*C^q*), 135.8 (CH*C^q*–Ar), 132.2 (Ar*C*H), 131.4 (Ar*C*H), 128.5 (2 × Ar*C*H), 128.5 (Ar*C*H), 127.3 (Ar*C*H), 126.1 (2 × Ar*C*H), 125.8 (Ar*C*H), 56.3 (C(O)*C*H), 45.8 (CH*C*(CH_3)$_2$), 44.6 (CH*C*H$_2$), 29.6 (CH$_2$C(*C*H$_3$)$_2$), 29.1 (CH$_2$C(*C*H$_3$)$_2$), 21.3 (C*C*H$_3$).

IR (neat, cm^{-1}): 2952, 2926, 1682, 1454, 1446, 1320, 1205, 1195, 917, 854, 764, 731, 691.

HRMS (ESI$^+$): calculated for $C_{21}H_{23}O$ (M + H$^+$): 291.1743 Found: 291.1739

rac-(4,4-Dimethyl-2-phenylcyclopent-2-en-1-yl)(2,6-dimethylphenyl)methanone (**3c**)

Prepared by following the **general procedure A** from 0.1 mmol (**1c**), 0.5 mmol (**2a**), and SmI_2 (25 mol%). The reaction mixture was filtered through a silica gel pad and concentrated in vacuo to afford the final product (30.3 mg, 0.099 mmol, 99% yield) as a light-yellow solid.

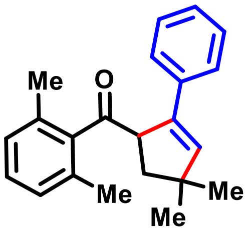

^{1}H NMR (400 MHz, Chloroform-*d*) δ: 7.20 – 7.14 (m, 2H, Ar*H*), 7.13 – 7.04 (m, 4H, Ar*H*), 6.87 (d, *J* = 7.6 Hz, 2H, Ar*H*), 5.96 (d, *J* = 1.8 Hz, 1H, C*H*C(CH_3)$_2$), 4.73 (ddd, *J* = 9.4, 3.9, 1.8 Hz, 1H, C(O)C*H*), 2.40 (dd, *J* = 13.1, 3.9 Hz, 1H, CHC*H$_2$*), 2.11 (dd, *J* = 13.1, 9.5 Hz, 1H, CHC*H$_2$*), 2.04 (s, 6H, 2 × CC*H$_3$*), 1.31 (s, 3H, CH$_2$C(C*H$_3$*)$_2$), 1.19 (s, 3H, CH$_2$C(C*H$_3$*)$_2$).

^{13}C NMR (101 MHz, Chloroform-*d*) δ: 210.7 (*C*=O), 142.4 (Ar*C^q*), 141.7 (C(CH_3)$_2$*C*H), 139.1 (Ar*C^q*), 136.1 (CH*C^q*–Ar), 133.9 (2 × Ar*C^q*–CH_3), 129 (Ar*C*H), 128.1 (4 × Ar*C*H), 127.3 (Ar*C*H), 126.5 (2 × Ar*C*H), 59.4 (C(O)*C*H), 45.3 (CH*C*(CH_3)$_2$), 42.4 (CH*C*H$_2$), 30.2 (CH$_2$C(*C*H$_3$)$_2$), 29.3 (CH$_2$C(*C*H$_3$)$_2$), 20.1 (2 × C*C*H$_3$).

IR (neat, cm^{-1}): 2956, 2927, 1687, 1460, 1312, 1198, 917, 872, 776, 756, 693.

HRMS (ESI$^+$): calculated for $C_{22}H_{24}ONa$ (M + Na$^+$): 327.1719 Found: 327.1716

rac-(4,4-Dimethyl-2-(o-tolyl)cyclopent-2-en-1-yl)(o-tolyl)methanone (**3e**)

Prepared by following the **general procedure A** from 0.1 mmol (**1b**), 0.5 mmol (**2e**), and SmI_2 (25 mol%). The reaction mixture was filtered through a silica gel pad and concentrated in vacuo to afford the final product (30.1 mg, 0.099 mmol, 99% yield) as a colourless oil.

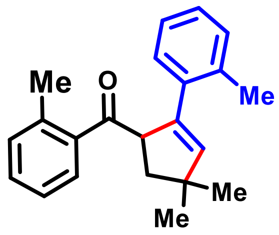

^{1}H NMR (400 MHz, Chloroform-*d*) δ: 7.46 (dd, *J* = 7.7, 1.3 Hz, 1H, Ar*H*), 7.30 – 7.25 (m, 1H, Ar*H*), 7.17 (d, *J* = 7.6 Hz, 1H, Ar*H*), 7.15 – 6.98 (m, 5H, Ar*H*), 5.71 (d, *J* = 1.9 Hz, 1H, C*H*C(CH$_3$)$_2$), 4.95 (ddd, *J* = 8.8, 6.6, 1.9 Hz, 1H, C(O)C*H*), 2.35 (s, 3H, C(O)CCC*H$_3$*), 2.27 – 2.21 (m, 1H, CHC*H$_2$*), 2.19 (s, 3H, CC*H$_3$*), 2.09 (dd, *J* = 12.7, 6.7 Hz, 1H, CHC*H$_2$*), 1.29 (s, 3H, CH$_2$C(C*H$_3$*)$_2$), 1.20 (s, 3H, CH$_2$C(C*H$_3$*)$_2$).

^{13}C NMR (101 MHz, Chloroform-*d*) δ: 206.4 (*C*=O), 143.4 (C(CH$_3$)$_2$*C*H), 139.3 (2 × ArC^q), 137.9 (ArC^q), 136.8 (ArC^q), 135.8 (CHC^q–Ar), 131.7 (Ar*C*H), 130.9 (Ar*C*H), 130.4 (Ar*C*H), 128.5 (Ar*C*H), 128 (Ar*C*H), 127 (Ar*C*H), 125.5 (Ar*C*H), 125.4 (Ar*C*H), 59.0 (C(O)*C*H), 45.9 (CH*C*(CH$_3$)$_2$), 44.3 (CH*C*H$_2$), 29.3 (CH$_2$C(*C*H$_3$)$_2$), 29.1 (CH$_2$C(*C*H$_3$)$_2$), 20.9 (C(O)CC*C*H$_3$), 20.7 (C*C*H$_3$).

IR (neat, cm^{-1}): 2954, 2927, 1711, 1682, 1455, 1253, 1209, 756, 733.

HRMS (ESI$^+$): calculated for $C_{22}H_{25}O$ (M + H$^+$): 305.1900 Found: 305.1895

rac-(4,4-Dimethyl-2-(m-tolyl)cyclopent-2-en-1-yl)(o-tolyl)methanone (**3f**)

Prepared by following the **general procedure A** from 0.1 mmol (**1b**), 0.5 mmol (**2f**), and SmI_2 (25 mol%). The reaction mixture was filtered through a silica gel pad and concentrated in vacuo to afford the final product (30.1 mg, 0.099 mmol, 99% yield) as a viscous liquid.

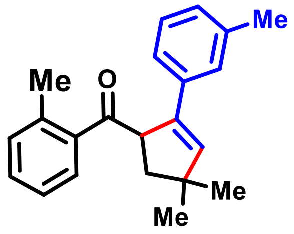

^{1}H NMR (400 MHz, Chloroform-*d*) δ 7.74 (dd, *J* = 7.7, 1.4 Hz, 1H, Ar*H*), 7.39 (td, *J* = 7.5, 1.4 Hz, 1H, Ar*H*), 7.33 – 7.23 (m, 2H, Ar*H*), 7.14 – 7.04 (m, 3H, Ar*H*), 6.98 (d, *J* = 7.3 Hz, 1H, Ar*H*), 6.18 (d, *J* = 1.6 Hz, 1H, C*H*C(CH$_3$)$_2$), 4.93 (ddd, *J* = 9.8, 5.5, 1.6 Hz, 1H, C(O)C*H*), 2.36 (s, 3H, C(O)CCC*H$_3$*), 2.30 (dd, *J* = 13.0, 9.8 Hz, 1H, CHC*H$_2$*), 2.25 (s, 3H, CC*H$_3$*), 1.98 (dd, *J* = 12.9, 5.5 Hz, 1H, CHC*H$_2$*), 1.21 (s, 3H, CH$_2$C(C*H$_3$*)$_2$), 1.19 (s, 3H, CH$_2$C(C*H$_3$*)$_2$).

^{13}C NMR (101 MHz, Chloroform-*d*) δ: 206.2 (*C*=O), 140.2 (C(CH$_3$)$_2$*C*H), 139.1 (ArC^q), 138.9 (ArC^q), 138.5 (ArC^q), 138.0 (ArC^q), 135.8 (CHC^q–Ar), 132.2 (Ar*C*H), 131.4 (Ar*C*H), 128.4 (Ar*C*H), 128.4 (Ar*C*H), 128.1 (Ar*C*H), 126.9 (Ar*C*H), 125.7 (Ar*C*H), 123.2 (Ar*C*H), 56.4 (C(O)*C*H), 45.7 (CH*C*(CH$_3$)$_2$), 44.6 (CH*C*H$_2$), 29.6 (CH$_2$C(*C*H$_3$)$_2$), 29.1 (CH$_2$C(*C*H$_3$)$_2$), 21.6 (C(O)CC*C*H$_3$), 21.1 (C*C*H$_3$).

IR (neat, cm^{-1}): 2952, 2926, 1683, 1600, 1454, 1319, 1207, 1194, 1012, 779, 731, 695.

HRMS (ESI$^+$): calculated for $C_{22}H_{25}O$ (M + H$^+$): 305.1900 Found: 305.1895

rac-(4,4-Dimethyl-2-(p-tolyl)cyclopent-2-en-1-yl)(o-tolyl)methanone (**3g**)

Prepared by following the **general procedure A** from 0.1 mmol (**1b**), 0.5 mmol (**2g**), and SmI_2 (25 mol%). The reaction mixture was filtered through a silica gel pad and concentrated in vacuo. The crude product was purified by column chromatography (silica gel 100-200 mesh size; hexane/ethyl acetate) to afford **3g** (23.2 mg, 0.076 mmol, 76% yield) as a yellow liquid.

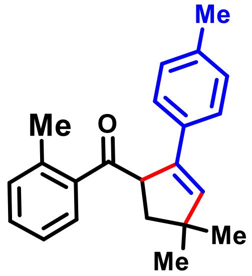

^{1}H NMR (400 MHz, Chloroform-*d*) δ: 7.77 (d, *J* = 7.6 Hz, 1H, Ar*H*), 7.40 (t, *J* = 7.5 Hz, 1H, Ar*H*), 7.34 – 7.27 (m, 2H, Ar*H*), 7.20 (d, *J* = 7.8 Hz, 2H, Ar*H*), 7.05 (d, *J* = 7.8 Hz, 2H, Ar*H*), 6.17 (s, 1H, C*H*C(CH_3)$_2$), 4.92 (dd, *J* = 10.0, 5.4 Hz, 1H, C(O)C*H*), 2.40 (s, 3H, *o*-CC*H$_3$*), 2.30 (s, 3H, *p*-CC*H$_3$*), 2.30 (dd, *J* = 13.0, 9.8 Hz, 1H, CHC*H*$_2$),1.98 (dd, *J* = 12.9, 5.4 Hz, 1H, CHC*H*$_2$), 1.22 (s, 3H, CH_2C(C*H*$_3$)$_2$), 1.20 (s, 3H, CH_2C(C*H*$_3$)$_2$).

^{13}C NMR (101 MHz, Chloroform-*d*) δ: 206.0 (*C*=O), 139.4 (C(CH_3)$_2$*C*H), 139.0 (Ar*C*^{q}), 138.8 (Ar*C*^{q}), 138.3 (Ar*C*^{q}), 137.0 (Ar*C*^{q}), 133.0 (CH*C*^{q}–Ar), 132.2 (Ar*C*H), 131.4 (Ar*C*H), 129.2 (2 × Ar*C*H), 128.5 (Ar*C*H), 126.0 (2 × Ar*C*H), 125.7 (Ar*C*H), 56.4 (C(O)*C*H), 45.7 (CH*C*(CH_3)$_2$), 44.6 (CH*C*H$_2$), 29.6 (CH_2C(*C*H$_3$)$_2$), 29.2 (CH_2C(*C*H$_3$)$_2$), 21.3 (*p*-C*C*H$_3$)., 21.3 (*o*-C*C*H$_3$).

IR (neat, cm^{-1}): 2951, 2925, 2862, 1682, 1512, 1454, 1320, 1207, 1194, 811, 753, 731.

HRMS (ESI$^+$): calculated for $C_{22}H_{25}O$ (M + H$^+$): 305.1900 Found: 305.1890

rac-(4,4-Dimethyl-2-(4-pentylphenyl)cyclopent-2-en-1-yl)(o-tolyl)methanone (**3h**)

Prepared by following the **general procedure A** from 0.1 mmol (**1b**), 0.5 mmol (**2h**), and SmI_2 (25 mol%). The reaction mixture was filtered through a silica gel pad and concentrated in vacuo. The crude product was purified by column chromatography (silica gel 100-200 mesh size; hexane/ethyl acetate) to afford **3h** (20.6 mg, 0.057 mmol, 57% yield) as a viscous liquid.

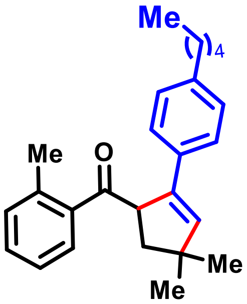

^{1}H NMR (400 MHz, Chloroform-*d*) δ: 7.75 (dd, *J* = 7.7, 1.4 Hz, 1H , Ar*H*), 7.39 (td, *J* = 7.5, 1.4 Hz, 1H, Ar*H*), 7.29 (td, *J* = 7.7, 1.4 Hz, 1H, Ar*H*), 7.25 (d, *J* = 6.3 Hz, 1H, Ar*H*), 7.23 – 7.18 (m, 2H, Ar*H*), 7.07 – 7.01 (m, 2H, Ar*H*), 6.17 (d, *J* = 1.6 Hz, 1H, C*H*C(CH_3)$_2$), 4.91 (ddd, *J* = 9.9, 5.3, 1.6 Hz, 1H, C(O)C*H*), 2.54 (t, *J* = 7.7 Hz, 2H, C*H*$_2$CH$_2$CH$_2$CH$_2$CH$_3$), 2.38 (s, 3H, CC*H*$_3$), 2.34 – 2.21 (m, 1H, CHC*H*$_2$), 1.97 (dd, *J* = 13.0, 5.3 Hz, 1H, CHC*H*$_2$), 1.59 – 1.44 (m, 2H, CH$_2$C*H*$_2$CH$_2$CH$_2$CH$_3$), 1.33 – 1.23 (m, 4H, CH$_2$CH$_2$C*H*$_2$C*H*$_2$CH$_3$), 1.20 (s, 3H, CH_2C(C*H*$_3$)$_2$), 1.19 (s, 3H, CH_2C(C*H*$_3$)$_2$), 0.87 (t, *J* = 6.9 Hz, 3H, CH$_2$C*H*$_3$).

^{13}C NMR (101 MHz, Chloroform-*d*) δ: 206.1 (*C*=O), 142.1 (C(CH_3)$_2$*C*H), 139.4 (Ar*C*^{q}–pentyl), 138.9 (Ar*C*^{q}), 138.8 (Ar*C*^{q}), 138.3 (Ar*C*^{q}), 133.1 (CH*C*^{q}–Ar), 132.2 (Ar*C*H), 131.4 (Ar*C*H), 128.6 (2 × Ar*C*H), 128.5 (Ar*C*H), 126.0 (2 × Ar*C*H), 125.7

(Ar*C*H), 56.4 (C(O)*C*H), 45.7 (CH*C*(CH_3)$_2$), 44.5 (CH*C*H_2), 35.7 (*C*$H_2C_4H_9$), 31.6 (CH_2*C*$H_2C_3H_7$), 31.3 (C_2H_4*C*$H_2C_2H_5$), 29.6 (CH_2C(*C*H_3)$_2$), 29.1 (CH_2C(*C*H_3)$_2$), 22.7 (C_3H_6*C*H_2CH_3), 21.3 (C*C*H_3), 14.2 (*C*H_3).

IR (neat, cm^{-1}): 2954, 2927, 2858, 1681, 1455, 1212, 997, 732.

HRMS (ESI$^+$): calculated for $C_{26}H_{31}O$ (M - H$^+$): 359.2380 Found: 359.2393

rac-(2-(4-(*tert*-Butyl)phenyl)-4,4-dimethylcyclopent-2-en-1-yl)(o-tolyl)methanone (**3i**)

Prepared by following the **general procedure A** from 0.1 mmol (**1b**), 0.5 mmol (**2i**), and SmI_2 (25 mol%). The reaction mixture was filtered through a silica gel pad and concentrated in vacuo. The crude product was purified by column chromatography (silica gel 100-200 mesh size; hexane/ethyl acetate) to afford **3i** (25 mg, 0.072 mmol, 72% yield) as a colourless oil.

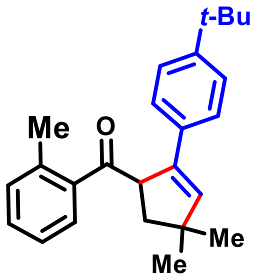

^{1}H NMR (400 MHz, Chloroform-*d*) δ 7.76 (d, *J* = 7.6 Hz, 1H, Ar*H*), 7.39 (t, *J* = 7.5 Hz, 1H, Ar*H*), 7.33 – 7.19 (m, 6H, Ar*H*), 6.17 (s, 1H, C*H*C(CH_3)$_2$), 4.91 (dd, *J* = 10.1, 5.3 Hz, 1H, C(O)C*H*), 2.39 (s, 3H, CC*H$_3$*), 2.28 (dd, *J* = 12.9, 10.0 Hz, 1H, CHC*H$_2$*), 1.95 (dd, *J* = 13.0, 5.3 Hz, 1H, CHC*H$_2$*), 1.27 (s, 9H, *t*-Bu), 1.20 (s, 3H, CH_2C(C*H$_3$*)$_2$), 1.18 (s, 3H, CH_2C(C*H$_3$*)$_2$).

^{13}C NMR (101 MHz, Chloroform-*d*) δ: 206.0 (*C*=O), 150.3 (Ar*C^q*–*t*-Bu), 139.6 (C(CH_3)$_2$*C*H), 139.0 (Ar*C^q*), 138.7 (Ar*C^q*), 138.3 (Ar*C^q*), 132.9 (CH*C^q*–Ar), 132.2 (Ar*C*H), 131.4 (Ar*C*H), 128.5 (Ar*C*H), 125.8 (2 × Ar*C*H), 125.7 (Ar*C*H), 125.5 (2 × Ar*C*H), 56.36 (C(O)C*H*), 45.7 (CH*C*(CH_3)$_2$), 44.5 (CH*C*H_2), 34.7 (*C*(CH_3)$_3$), 31.5 (3 × C(*C*H_3)$_3$), 29.6 (CH_2C(*C*H_3)$_2$), 29.1 (CH_2C(*C*H_3)$_2$), 21.3 (C*C*H_3).

IR (neat, cm^{-1}): 2955, 2929, 2864, 1682, 1457, 1362, 1267, 1195, 831, 755, 732

HRMS (ESI$^+$): calculated for $C_{25}H_{31}O$ (M + H$^+$): 347.2369 Found: 347.2364

rac-(2-Mesityl-4,4-dimethylcyclopent-2-en-1-yl)(o-tolyl)methanone (**3j**)

Prepared by following the **general procedure A** from 0.1 mmol (**1b**), 0.5 mmol (**2j**), and SmI_2 (25 mol%). The reaction mixture was filtered through a silica gel pad and concentrated in vacuo. The crude product was purified by column chromatography (silica gel 100-200 mesh size; hexane/ethyl acetate) to afford **3j** (23 mg, 0.069 mmol, 69% yield) as a viscous liquid.

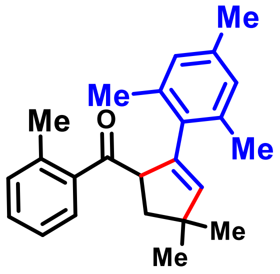

^{1}H NMR (400 MHz, Chloroform-*d*) δ: 7.23 – 7.15 (m, 2H, Ar*H*), 7.08 – 7.00 (m, 2H, Ar*H*), 6.71 (s, 2H, Ar*H*), 5.50 (d, *J* = 2.3 Hz, 1H, C*H*C(CH_3)$_2$), 4.88 (td, *J* = 7.8, 2.3 Hz, 1H, C(O)C*H*), 2.29 (dd, *J* = 12.6, 7.3 Hz, 1H, CHC*H$_2$*), 2.19 (s, 9H, 3 × C*H$_3$*), 2.16 (s, 3H, CC*H$_3$*), 2.11 (dd, *J* = 12.6, 8.2 Hz, 1H, CHC*H$_2$*), 1.33 (s, 3H, CH_2C(C*H$_3$*)$_2$), 1.21 (s, 3H, CH_2C(C*H$_3$*)$_2$).

^{13}C NMR (101 MHz, Chloroform-*d*) δ: 206.6 (*C*=O), 143.3 (C(CH_3)$_2$*C*H), 139.7 (Ar*C^q*), 138.5 (Ar*C^q*), 137.4 (Ar*C^q*), 136.3 (2 × Ar*C^q*), 133.5 (CH*C^q*–Ar), 131.4 (Ar*C*H), 130.6

(Ar*C*H), 128.2 (Ar*C*H), 127.8 (Ar*C*H), 125.3 (Ar*C*H), 58.5 (C(O)*C*H), 45.4 (CH*C*$(CH_3)_2$), 43.9 (CH*C*H_2), 29.2 (CH_2C(*C*$H_3)_2$), 28.8 (CH_2C(*C*$H_3)_2$), 21.1 (*p*-*C*H_3), 20.6 (*o*-*C*H_3), 20.5 (*o*-*C*H_3).

IR (neat, cm^{-1}): 2951, 2924, 2861, 1682, 1455, 1210, 1033, 914, 850, 752, 728, 665.

HRMS (ESI$^+$): calculated for $C_{24}H_{29}O$ (M + H$^+$): 333.2213 Found: 333.2204

rac-(2-(4-Methoxy-2-methylphenyl)-4,4-dimethylcyclopent-2-en-1-yl)(o-tolyl)methanone (**3k**)

Prepared by following the **general procedure A** from 0.1 mmol (**1b**), 0.5 mmol (**2k**), and SmI_2 (25 mol%). The reaction mixture was filtered through a silica gel pad and concentrated in vacuo. The crude product was purified by column chromatography (silica gel 100-200 mesh size; hexane/ethyl acetate) to afford **3k** (20.4 mg, 0.061 mmol, 61% yield) as a white solid.

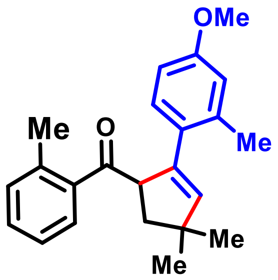

^{1}H NMR (400 MHz, Chloroform-*d*) δ: 7.46 (dd, *J* = 7.7, 1.4 Hz, 1H, Ar*H*), 7.29 (dd, *J* = 7.5, 1.4 Hz, 1H, Ar*H*), 7.23 – 7.10 (m, 2H, Ar*H*), 7.01 (d, *J* = 8.4 Hz, 1H, Ar*H*), 6.65 (d, *J* = 2.7 Hz, 1H, Ar*H*), 6.57 (dd, *J* = 8.5, 2.8 Hz, 1H, Ar*H*), 5.65 (d, *J* = 1.9 Hz, 1H, C*H*C$(CH_3)_2$), 4.90 (ddd, *J* = 8.8, 6.6, 1.9 Hz, 1H, C(O)C*H*), 3.74 (s, 3H, OC*H_3*), 2.33 (s, 3H, CC*H_3*), 2.21 (s, 3H, CC*H_3*), 2.18 (d, *J* = 9.1 Hz, 1H, CHC*H_2*), 2.06 (dd, *J* = 12.7, 6.6 Hz, 1H, CHC*H_2*), 1.27 (s, 3H, CH_2C(C*H_3*$)_2$), 1.19 (s, 3H, CH_2C(C*H_3*$)_2$).

^{13}C NMR (101 MHz, Chloroform-*d*) δ 206.7 (*C*=O), 158.4 (Ar*C^q*–OCH_3), 142.9 (C$(CH_3)_2$*C*H), 139.3 (Ar*C^q*), 138.8 (Ar*C^q*), 137.9 (Ar*C^q*), 137.4 (Ar*C^q*), 131.7 (Ar*C*H), 130.9 (Ar*C*H), 129.6 (Ar*C*H), 129.4 (CH*C^q*–Ar), 128.0 (Ar*C*H), 125.5 (Ar*C*H), 116.0 (Ar*C*H), 110.8 (Ar*C*H), 59.1 (C(O)*C*H), 55.3 (O*C*H_3), 45.8 (CH*C*$(CH_3)_2$), 44.2 (CH*C*H_2), 29.4 (CH_2C(*C*$H_3)_2$), 29.2 (CH_2C(*C*$H_3)_2$), 21.3 (C*C*H_3), 20.8 (C*C*H_3).

IR (neat, cm^{-1}): 2951, 2932, 1682, 1604, 1499, 1453, 1309, 1239, 1222, 1196, 1165, 1106, 1041, 1002, 941, 858, 848, 815, 757, 733, 662.

HRMS (ESI$^+$): calculated for $C_{23}H_{27}O_2$ (M + H$^+$): 335.2006 Found: 335.1998

rac-(2-(4-Methoxyphenyl)-4,4-dimethylcyclopent-2-en-1-yl)(o-tolyl)methanone (**3l**)

Prepared by following the **general procedure A** from 0.1 mmol (**1b**), 0.5 mmol (**2l**), and SmI_2 (25 mol%). The reaction mixture was filtered through a silica gel pad and concentrated in vacuo. The crude product was purified by column chromatography (silica gel 100-200 mesh size; hexane/ethyl acetate) to afford **3g** (12.8 mg, 0.04 mmol, 40% yield) as a colourless liquid.

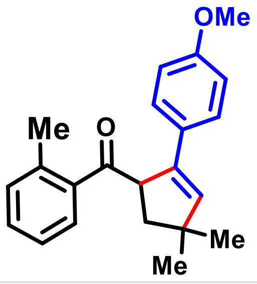

^{1}H NMR (400 MHz, Chloroform-*d*) δ 7.74 (d, *J* = 7.6 Hz, 1H, Ar*H*), 7.38 (t, *J* = 7.5 Hz, 1H, Ar*H*), 7.32 – 7.19 (m, 4H, Ar*H*), 6.79 – 6.71

(m, 2H, Ar*H*), 6.08 (s, 1H, C*H*C(CH_3)$_2$), 4.88 (dd, *J* = 10.1, 5.2 Hz, 1H, C(O)C*H*), 3.76 (s, 3H, OCH_3), 2.38 (s, 3H, CCH_3), 2.29 (dd, *J* = 12.9, 10.1 Hz, 1H, CHCH_2), 1.96 (dd, *J* = 13.0, 5.1 Hz, 1H, CHCH_2), 1.19 (s, 3H, CH_2C(CH_3)$_2$), 1.19 (s, 3H, CH_2C(CH_3)$_2$).

^{13}C NMR (101 MHz, Chloroform-*d*) δ: 206.1 (*C*=O), 158.9 (ArC^q–OCH_3), 139 (ArC^q), 138.5 (C(CH_3)$_2$*C*H), 138.3 (ArC^q), 138.3 (ArC^q), 132.2 (Ar*C*H), 131.4 (Ar*C*H), 128.6 (CHC^q–Ar), 128.5 (Ar*C*H), 127.3 (2 × Ar*C*H), 125.8 (Ar*C*H), 114 (2 × Ar*C*H), 56.5 (C(O)*C*H), 55.5 (OCH_3), 45.7 (CH*C*(CH_3)$_2$), 44.6 (CH*C*H_2), 29.7 (CH_2C(*C*H_3)$_2$), 29.2 (CH_2C(*C*H_3)$_2$), 21.3 (C*C*H_3).

IR (neat, cm^{-1}): 2953, 2927, 1681, 1605, 1510, 1456, 1293, 1248, 1209, 1176, 1031, 828, 752, 732.

HRMS (ESI$^+$): calculated for $C_{22}H_{25}O_2$ (M + H$^+$): 321.1849 Found: 321.1835

rac-(4,4-Dimethyl-2-(4-phenoxyphenyl)cyclopent-2-en-1-yl)(o-tolyl)methanone (**3m**)

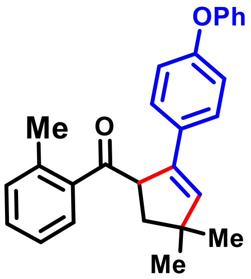

Prepared by following the **general procedure A** from 0.1 mmol (**1b**), 0.5 mmol (**2m**), and SmI_2 (25 mol%). The reaction mixture was filtered through a silica gel pad and concentrated in vacuo. The crude product was purified by column chromatography (silica gel 100-200 mesh size; hexane/ethyl acetate) to afford **3m** (15.3 mg, 0.04 mmol, 40% yield) as a yellow solid.

^{1}H NMR (500 MHz, Chloroform-*d*) δ: 7.75 (d, *J* = 7.7 Hz, 1H, Ar*H*), 7.39 (t, *J* = 7.5 Hz, 1H, Ar*H*), 7.34 – 7.23 (m, 6H, Ar*H*), 7.08 (t, *J* = 7.4 Hz, 1H, Ar*H*), 6.97 (d, *J* = 8.0 Hz, 2H, Ar*H*), 6.89 (d, *J* = 8.4 Hz, 2H, Ar*H*), 6.15 (s, 1H, C*H*C(CH_3)$_2$), 4.91 (dd, *J* = 10.1, 5.1 Hz, 1H, C(O)C*H*), 2.40 (s, 3H, CCH_3), 2.31 (dd, *J* = 13.0, 10.0 Hz, 1H, CHCH_2), 1.99 (dd, *J* = 13.0, 5.1 Hz, 1H, CHCH_2), 1.22 (s, 3H, CH_2C(CH_3)$_2$), 1.21 (s, 3H, CH_2C(CH_3)$_2$).

^{13}C NMR (101 MHz, Chloroform-*d*) δ: 205.9 (*C*=O), 157.5 (ArC^q of OPh), 156.4 (ArC^q–OPh), 139.6 (C(CH_3)$_2$*C*H), 139.0 (ArC^q), 138.2 (ArC^q), 138.2 (ArC^q), 132.2 (Ar*C*H), 131.5 (Ar*C*H), 131.2 (CHC^q–Ar), 129.9 (2 × Ar*C*H), 128.5 (Ar*C*H), 127.5 (2 × Ar*C*H), 125.8 (Ar*C*H), 123.3 (Ar*C*H), 119.1 (2 × Ar*C*H), 118.9 (2 × Ar*C*H), 56.5 (C(O)*C*H), 45.8 (CH*C*(CH_3)$_2$), 44.5 (CH*C*H_2), 29.7 (CH_2C(*C*H_3)$_2$), 29.1 (CH_2C(*C*H_3)$_2$), 21.3 (C*C*H_3).

IR (neat, cm^{-1}): 2953, 2923, 1681, 1587, 1505, 1488, 1455, 1233, 1166, 870, 836, 749, 732, 691.

HRMS (ESI$^+$): calculated for $C_{27}H_{27}O_2$ (M + H$^+$): 383.2006 Found: 383.1999

rac-(4,4-Dimethyl-2-(4-(trifluoromethoxy)phenyl)cyclopent-2-en-1-yl)(o tolyl)methanone (**3n**)

Prepared by following the **general procedure A** from 0.1 mmol (**1b**), 0.5 mmol (**2n**), and SmI_2 (25 mol%). The reaction mixture was filtered through a silica gel pad and concentrated in vacuo to afford the final product (37.1 mg, 0.099 mmol, 99% yield) as a viscous liquid.

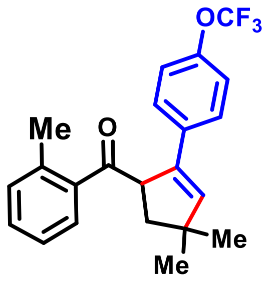

¹H NMR (400 MHz, Chloroform-*d*) δ: 7.71 (dd, *J* = 7.7, 1.4 Hz, 1H, Ar*H*), 7.37 (td, *J* = 7.5, 1.4 Hz, 1H, Ar*H*), 7.31 – 7.20 (m, 4H, Ar*H*), 7.05 (d, *J* = 8.3 Hz, 2H, Ar*H*), 6.16 (d, *J* = 1.5 Hz, 1H, C*H*C(CH₃)₂), 4.89 (ddd, *J* = 10.1, 5.3, 1.6 Hz, 1H, C(O)C*H*), 2.35 (s, 3H, CC*H₃*), 2.29 (dd, *J* = 13.0, 10.0 Hz, 1H, CHC*H₂*), 1.96 (dd, *J* = 13.0, 5.2 Hz, 1H, CHC*H₂*), 1.18 (s, 3H, CH₂C(C*H₃*)₂), 1.17 (s, 3H, CH₂C(C*H₃*)₂).

¹³C NMR (101 MHz, Chloroform-*d*) δ: 205.5 (*C*=O), 148.3 (q, *J* = 2 Hz, Ar*C*^{q}–OCF₃), 141.4 (C(CH₃)₂*C*H), 139.1 (Ar*C*^{q}), 137.9 (Ar*C*^{q}), 137.7 (Ar*C*^{q}), 134.8 (CH*C*^{q}–Ar), 132.3 (Ar*C*H), 131.7 (Ar*C*H), 128.6 (Ar*C*H), 127.4 (2 × Ar*C*H), 125.9 (Ar*C*H), 121.1 (2 × Ar*C*H), 120.7 (q, *J* = 258.0 Hz, O*C*F₃), 56.3 (C(O)*C*H), 45.9 (CH*C*(CH₃)₂), 44.5 (CH*C*H₂), 29.5 (CH₂C(*C*H₃)₂), 29.0 (CH₂C(*C*H₃)₂), 21.3 (C*C*H₃).

¹⁹F NMR (376 MHz, Chloroform-*d*) δ: –57.9 (s).

IR (neat, cm⁻¹): 2958, 2929, 1682, 1508, 1252, 1206, 1158, 1108, 1035, 1016, 918, 843, 804, 733, 646.

HRMS (ESI⁺): calculated for $C_{22}H_{20}O_2F_3$ (M - H⁺): 373.1421 Found: 373.1431

rac-(2-(2-Methoxyphenyl)-4,4-dimethylcyclopent-2-en-1-yl)(o-tolyl)methanone (**3o**)

Prepared by following the **general procedure A** from 0.1 mmol (**1b**), 0.5 mmol (**2o**), and SmI_2 (25 mol%). The reaction mixture was filtered through a silica gel pad and concentrated in vacuo. The crude product was purified by column chromatography (silica gel 100-200 mesh size; hexane/ethyl acetate) to afford **3o** (19.2 mg, 0.06 mmol, 60% yield) as a white solid.

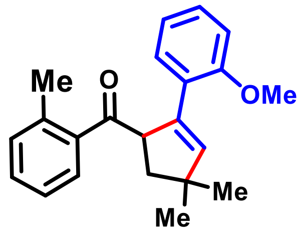

¹H NMR (500 MHz, Chloroform-*d*) δ: 7.70 (dd, *J* = 7.7, 1.4 Hz, 1H, Ar*H*), 7.32 (ddd, *J* = 9.0, 7.5, 1.6 Hz, 2H, Ar*H*), 7.27 – 7.22 (m, 1H, Ar*H*), 7.20 – 7.09 (m, 2H, Ar*H*), 6.86 (td, *J* = 7.5, 1.1 Hz, 1H, Ar*H*), 6.70 (dd, *J* = 8.2, 1.0 Hz, 1H, Ar*H*), 6.08 (d, *J* = 1.9 Hz, 1H, C*H*C(CH₃)₂), 5.24 (ddd, *J* = 9.2, 7.6, 2.0 Hz, 1H, C(O)C*H*), 3.51 (s, 3H, OC*H₃*), 2.27 (s, 3H, CC*H₃*), 2.19 (dd, *J* = 12.5, 8.8 Hz, 1H, CHC*H₂*), 1.95 (dd, *J* = 12.5, 7.6 Hz, 1H, CHC*H₂*), 1.24 (s, 3H, CH₂C(C*H₃*)₂), 1.20 (s, 3H, CH₂C(C*H₃*)₂).

¹³C NMR (101 MHz, Chloroform-*d*) δ: 206.0 (*C*=O), 156.4 (Ar*C*^{q}–OCH₃), 142.5 (C(CH₃)₂*C*H), 138.8 (Ar*C*^{q}), 138.5 (Ar*C*^{q}), 138.1 (CH*C*^{q}–Ar), 131.8 (Ar*C*H), 130.9 (Ar*C*H), 129.6 (Ar*C*H), 128.4 (2 × Ar*CH*), 125.5 (Ar*C*^{q}C(OCH₃)), 125.5 (Ar*C*H), 120.8 (Ar*C*H), 110.6 (Ar*C*H), 57.4 (C(O)*C*H), 54.8 (O*C*H₃), 44.9 (CH*C*(CH₃)₂), 44.6 (CH*C*H₂), 29.1 (CH₂C(*C*H₃)₂), 28.9 (CH₂C(*C*H₃)₂), 21.0 (C*C*H₃).

IR (neat, cm^{-1}): 2941, 2924, 1685, 1488, 1463, 1453, 1436, 1312, 1262, 1235, 1210, 1197, 1181, 1122, 1099, 1023, 866, 773, 751, 727, 664.

HRMS (ESI$^+$): calculated for $C_{22}H_{25}O_2$ (M + H$^+$): 321.1849 Found: 321.1847

rac-(2-(3-Methoxyphenyl)-4,4-dimethylcyclopent-2-en-1-yl)(o-tolyl)methanone (**3p**)

Prepared by following the **general procedure A** from 0.1 mmol (**1b**), 0.5 mmol (**2p**), and SmI_2 (25 mol%). The reaction mixture was filtered through a silica gel pad and concentrated in vacuo. The crude product was purified by column chromatography (silica gel 100-200 mesh size; hexane/ethyl acetate) to afford **3p** (21.2 mg, 0.066 mmol, 66% yield) as a viscous liquid.

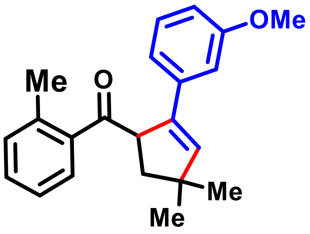

^{1}H NMR (400 MHz, Chloroform-*d*) δ 7.75 (d, *J* = 7.7 Hz, 1H, Ar*H*), 7.38 (t, *J* = 7.5 Hz, 1H, Ar*H*), 7.33 – 7.23 (m, 2H, Ar*H*), 7.14 (t, *J* = 7.9 Hz, 1H, Ar*H*), 6.86 (d, *J* = 8.3 Hz, 2H, Ar*H*), 6.77 – 6.71 (m, 1H, Ar*H*), 6.20 (s, 1H, C*H*C(CH_3)$_2$), 4.93 (dd, *J* = 10.0, 5.5 Hz, 1H, C(O)C*H*), 3.71 (d, *J* = 1.4 Hz, 3H, OC*H$_3$*), 2.37 (s, 3H, CC*H$_3$*), 2.34 – 2.26 (m, 1H, CHC*H$_2$*), 1.98 (dd, *J* = 13.0, 5.4 Hz, 1H, CHC*H$_2$*), 1.22 (s, 3H, CH_2C(C*H$_3$*)$_2$), 1.20 (s, 3H, CH_2C(C*H$_3$*)$_2$).

^{13}C NMR (101 MHz, Chloroform-*d*) δ: 206.0 (*C*=O), 159.7 (Ar*C^q*–OCH_3), 140.7 (C(CH_3)$_2$*C*H), 139.0 (Ar*C^q*), 139.0 (Ar*C^q*), 138.3 (Ar*C^q*), 137.3 (CH*C^q*–Ar), 132.2 (Ar*C*H), 131.4 (Ar*C*H), 129.5 (Ar*C*H), 128.5 (Ar*C*H), 125.8 (Ar*C*H), 118.7 (Ar*C*H), 113.2 (Ar*C*H), 111.5 (Ar*C*H), 56.4 (C(O)*C*H), 55.3 (O*C*H_3), 45.8 (CH*C*(CH_3)$_2$), 44.5 (CH*C*H_2), 29.5 (CH_2C(*C*H_3)$_2$), 29.1 (CH_2C(*C*H_3)$_2$), 21.2 (C*C*H_3).

IR (neat, cm^{-1}): 2952, 2927, 1683, 1598, 1576, 1454, 1287, 1227, 1208, 1195, 1162, 1037, 850, 774, 731, 689.

HRMS (ESI$^+$): calculated for $C_{22}H_{25}O_2$ (M + H$^+$): 321.1849 Found: 321.1841

rac-(2-(4-(Dimethylamino)phenyl)-4,4-dimethylcyclopent-2-en-1-yl)(o-tolyl)methanone (**3q**)

Prepared by following the **general procedure A** from 0.1 mmol (**1b**), 0.5 mmol (**2q**), and SmI_2 (25 mol%). The reaction mixture was filtered through a silica gel pad and concentrated in vacuo. The crude product was purified by column chromatography (silica gel 100-200 mesh size; hexane/ethyl acetate) to afford **3q** (14.1 mg, 0.042 mmol, 42% yield) as a brown viscous liquid.

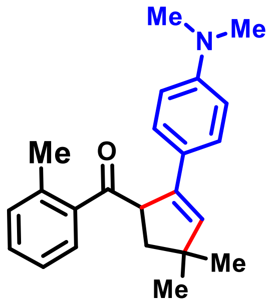

^{1}H NMR (400 MHz, Chloroform-*d*) δ: 7.74 (d, *J* = 7.7 Hz, 1H, Ar*H*), 7.38 (td, *J* = 7.4, 1.3 Hz, 1H, Ar*H*), 7.24 – 7.30 (m, 2H, Ar*H*), 7.18 (d, *J* = 8.6 Hz, 2H, Ar*H*), 6.60 (d, *J* = 8.5 Hz, 2H, Ar*H*), 6.02 (d, *J* = 1.4 Hz, 1H, C*H*C(CH_3)$_2$), 4.86 (ddd, *J* = 10.0, 5.2, 1.5 Hz, 1H, C(O)C*H*), 2.90 (s, 2 × 3H, N(C*H$_3$*)$_2$), 2.40 (s, 3H, CC*H$_3$*), 2.27 (dd, *J* = 13.0,

10.0 Hz, 1H, CHCH_2), 1.94 (dd, J = 12.9, 5.2 Hz, 1H, CHCH_2), 1.18 (s, 3H, $CH_2C(CH_3)_2$), 1.17 (s, 3H, $CH_2C(CH_3)_2$).

^{13}C NMR (101 MHz, Chloroform-*d*) δ: 206.4 (*C*=O), 149.9 (ArC^q–N(CH_3)$_2$), 138.9 (ArC^q), 138.6 (ArC^q), 138.5 (CHC^q–Ar), 136.5 (C(CH_3)$_2$*C*H), 132.1 (Ar*C*H), 131.3 (Ar*C*H), 128.5 (Ar*C*H), 127.0 (2 × Ar*C*H), 125.7 (Ar*C*H), 124.3 (ArC^q), 112.6 (2 × Ar*C*H), 56.6 (C(O)*C*H), 45.7 (CH*C*(CH_3)$_2$), 44.6 (CH*C*H_2), 40.8 (N(*C*H_3)$_2$), 29.9 (CH_2C(*C*H_3)$_2$), 29.3 (CH_2C(*C*H_3)$_2$), 21.3 (C*C*H_3).

IR (neat, cm^{-1}): 2954, 2927, 1678, 1606, 1520, 1325, 1164, 1117.

HRMS (ESI$^+$): calculated for $C_{23}H_{28}ON$ (M + H$^+$): 334.2165 Found: 334.2160

rac- (2-(2-Chlorophenyl)-4,4-dimethylcyclopent-2-en-1-yl)(o-tolyl)methanone (**3r**)

Prepared by following the **general procedure A** from 0.1 mmol (**1b**), 0.5 mmol (**2r**), and SmI_2 (25 mol%). The reaction mixture was filtered through a silica gel pad and concentrated in vacuo. The crude product was purified by column chromatography (silica gel 100-200 mesh size; hexane/ethyl acetate) to afford **3r** (31.5 mg, 0.097 mmol, 97% yield) as a colourless oil.

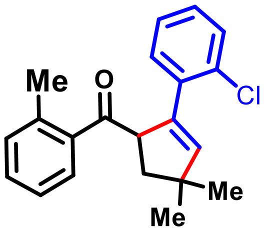

^{1}H NMR (400 MHz, Chloroform-*d*) δ 7.58 (dd, J = 7.8, 1.4 Hz, 1H, Ar*H*), 7.33 – 7.24 (m, 2H, Ar*H*), 7.23 (dd, J = 7.3, 2.0 Hz, 1H, Ar*H*), 7.21 – 7.16 (m, 1H, Ar*H*), 7.15 – 7.05 (m, 3H, Ar*H*), 5.88 (d, J = 1.9 Hz, 1H, C*H*C(CH_3)$_2$), 5.25 (ddd, J = 8.9, 6.9, 1.9 Hz, 1H, C(O)C*H*), 2.28 (s, 3H, CCH_3), 2.22 (dd, J = 12.6, 9.0 Hz, 1H, CHCH_2), 2.04 (dd, J = 12.6, 6.9 Hz, 1H, CHCH_2), 1.25 (s, 3H, $CH_2C(CH_3)_2$), 1.22 (s, 3H, $CH_2C(CH_3)_2$).

^{13}C NMR (101 MHz, Chloroform-*d*) δ: 205.7 (*C*=O), 144.9 (C(CH_3)$_2$*C*H), 138.6 (ArC^q), 138.5 (ArC^q), 138.1 (ArC^q–Cl), 136.5 (CHC^q–Ar), 132.2 (ArC^q–Cl), 131.7 (Ar*C*H), 131.3 (Ar*C*H), 131.1 (Ar*C*H), 129.6 (Ar*C*H), 128.4 (Ar*C*H), 128.4 (Ar*C*H), 126.7 (Ar*C*H), 125.6 (Ar*C*H), 57.8 (C(O)*C*H), 45.3 (CH*C*(CH_3)$_2$), 44.26 (CH*C*H_2), 28.9 (CH_2C(*C*H_3)$_2$), 28.9 (CH_2C(*C*H_3)$_2$), 21.0 (C*C*H_3).

IR (neat, cm^{-1}): 2953, 2927, 2863, 1682, 1467, 1432, 1315, 1287, 1210, 1196, 1064, 1034, 916, 858, 751, 730, 705, 663.

HRMS (ESI$^+$): calculated for $C_{21}H_{20}ClO$ (M - H$^+$): 323.1208 Found: 323.1216

rac- (2-(2-Bromophenyl)-4,4-dimethylcyclopent-2-en-1-yl)(o-tolyl)methanone (**3s**)

Prepared by following the **general procedure A** from 0.1 mmol (**1b**), 0.5 mmol (**2s**), and SmI_2 (25 mol%). The reaction mixture was filtered through a silica gel pad and concentrated in vacuo. The crude product was purified by column chromatography (silica gel 100-200 mesh size; hexane/ethyl acetate) to afford **3s** (36.6 mg, 0.099 mmol, 99% yield) as a viscous liquid.

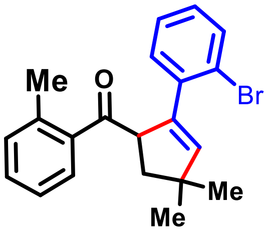

^{1}H NMR (400 MHz, Chloroform-*d*) δ 7.52 (dd, *J* = 7.7, 1.4 Hz, 1H, Ar*H*), 7.41 (dd, *J* = 8.0, 1.3 Hz, 1H, Ar*H*), 7.30 – 7.22 (m, 2H, Ar*H*), 7.14 (dddd, *J* = 10.8, 9.0, 6.0, 3.9 Hz, 3H, Ar*H*), 7.00 (td, *J* = 7.7, 1.8 Hz, 1H, Ar*H*), 5.77 (d, *J* = 1.9 Hz, 1H, C*H*C(CH_3)$_2$), 5.24 (ddd, *J* = 8.8, 6.6, 1.9 Hz, 1H, C(O)C*H*), 2.27 (s, 3H, CCH_3), 2.20 (dd, *J* = 12.6, 9.0 Hz, 1H, CHCH_2), 2.06 (dd, *J* = 12.7, 6.6 Hz, 1H, CHCH_2), 1.24 (s, 3H, CH_2C(CH_3)$_2$), 1.21 (s, 3H, CH_2C(CH_3)$_2$).

^{13}C NMR (101 MHz, Chloroform-*d*) δ: 205.8 (*C*=O), 144.7 (C(CH_3)$_2$*C*H), 139.8 (Ar*C*^{q}), 138.8 (Ar*C*^{q}), 138.6 (Ar*C*^{q}), 138.0 (CH*C*^{q}–Ar), 132.6 (Ar*C*H), 131.8 (Ar*C*H), 131.7 (Ar*C*H), 131.0 (Ar*C*H), 128.6 (Ar*C*H), 128.4 (Ar*C*H), 127.2 (Ar*C*H), 125.6 (Ar*C*H), 122.2 (Ar*C*^{q}–Br), 57.8 (C(O)*C*H), 45.4 (CH*C*(CH_3)$_2$), 44.1 (CH*C*H$_2$), 28.9 (CH_2C(*C*H$_3$)$_2$), 28.9 (CH_2C(*C*H$_3$)$_2$), 21.0 (C*C*H$_3$).

IR (neat, cm^{-1}): 2953, 2926, 1681, 1464, 1211, 1196, 1022, 750, 732.

HRMS (ESI$^+$): calculated for $C_{21}H_{22}BrO$ (M + H$^+$): 369.0849 Found: 369.0840

rac-(4,4-Dimethyl-2-(2-(trifluoromethyl)phenyl)cyclopent-2-en-1-yl)(o-tolyl)methanone (**3t**)

Prepared by following the **general procedure A** from 0.1 mmol (**1b**), 0.5 mmol (**2t**), and SmI_2 (25 mol%). The reaction mixture was filtered through a silica gel pad and concentrated in vacuo. The crude product was purified by column chromatography (silica gel 100-200 mesh size; hexane/ethyl acetate) to afford **3t** (20.5 mg, 0.057 mmol, 57% yield) as a viscous liquid.

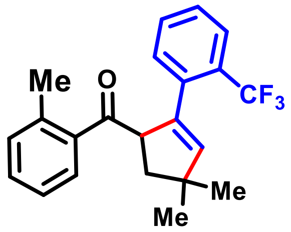

^{1}H NMR (400 MHz, Chloroform-*d*) δ 7.57 (d, *J* = 7.8 Hz, 1H, Ar*H*), 7.47 – 7.38 (m, 3H, Ar*H*), 7.32 – 7.26 (m, 2H, Ar*H*), 7.15 (d, *J* = 7.0 Hz, 2H, Ar*H*), 5.73 (s, 1H, C*H*C(CH_3)$_2$), 4.94 (dd, *J* = 8.9, 6.8 Hz, 1H, C(O)C*H*), 2.33 (s, 3H, CCH_3), 2.21 (dd, *J* = 12.4, 9.3 Hz, 1H, CHCH_2), 2.03 (dd, *J* = 12.7, 6.6 Hz, 1H, CHCH_2), 1.26 (s, 3H, CH_2C(CH_3)$_2$), 1.20 (s, 3H, CH_2C(CH_3)$_2$).

^{13}C NMR (101 MHz, Chloroform-*d*) δ: 205.9 (*C*=O), 145.2 (C(CH_3)$_2$*C*H), 138.7 (Ar*C*^{q}), 138.1 (Ar*C*^{q}), 137.2 (Ar*C*^{q}), 137.2 (CH*C*^{q}–Ar), 132.4 (Ar*C*H), 131.8 (Ar*C*H), 131.4 (Ar*C*H), 131.2 (Ar*C*H), 128.4 (Ar*C*H), 127.2 (Ar*C*H), 125.8 (q, *J* = 5.6 Hz, Ar*C*H), 124.9 (q, *J* = 239.9 Hz, Ar*C*F$_3$), 125.7 (Ar*C*H), 59.5 (C(O)*C*H), 45.5 (CH*C*(CH_3)$_2$), 44.7 (CH*C*H$_2$), 28.9 (CH_2C(*C*H$_3$)$_2$), 28.4 (CH_2C(*C*H$_3$)$_2$), 21.1 (C*C*H$_3$) "Ar*C*^{q}CF$_3$ not observed."

^{19}F NMR (376 MHz, Chloroform-*d*) δ: –58.4 (s).

IR (neat, cm^{-}1): 2955, 2928, 1680, 1448, 1313, 1257, 1212, 1163, 1121, 1106, 1065, 1033, 998, 916, 767, 730, 662.

HRMS (ESI$^+$): calculated for $C_{22}H_{22}F_3O$ (M + H$^+$): 359.1617 Found: 359.1605

rac-(2-(4-Bromophenyl)-4,4-dimethylcyclopent-2-en-1-yl)(o-tolyl)methanone (**3u**)

Prepared by following the **general procedure A** from 0.1 mmol (**1b**), 0.5 mmol (**2u**), and SmI_2 (25 mol%). The reaction mixture was filtered through a silica gel pad and concentrated in vacuo to afford the final product (36.6 mg, 0.099 mmol, 99% yield) as a yellow liquid.

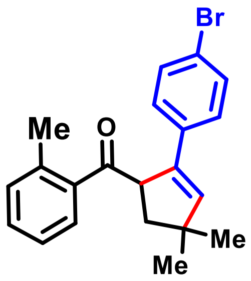

^{1}H NMR (400 MHz, Chloroform-*d*) δ 7.74 (dd, *J* = 7.7, 1.4 Hz, 1H, Ar*H*), 7.40 (td, *J* = 7.5, 1.4 Hz, 1H, Ar*H*), 7.37 – 7.33 (m, 2H, Ar*H*), 7.31 (d, *J* = 7.6 Hz, 1H, Ar*H*), 7.28 – 7.24 (m, 1H, Ar*H*), 7.15 (d, *J* = 8.5 Hz, 2H, Ar*H*), 6.20 (d, *J* = 1.5 Hz, 1H, C*H*C(CH_3)$_2$), 4.89 (ddd, *J* = 10.0, 5.3, 1.6 Hz, 1H, C(O)C*H*), 2.39 (s, 3H, CC*H$_3$*), 2.31 (dd, *J* = 13.0, 10.0 Hz, 1H, CHC*H*$_2$), 1.96 (dd, *J* = 13.0, 5.3 Hz, 1H, CHC*H*$_2$), 1.19 (s, 2 × 3H, CH$_2$C(C*H$_3$*)$_2$).

^{13}C NMR (101 MHz, Chloroform-*d*) δ 205.5 (*C*=O), 141.2 (C(CH_3)$_2$CH), 139.2 (Ar*C^q*), 138.0 (Ar*C^q*), 137.8 (Ar*C^q*), 134.9 (CH*C^q*–Ar), 132.4 (Ar*C*H), 131.7 (Ar*C*H), 131.6 (2 × Ar*CH*), 128.6 (Ar*C*H), 127.7 (2 × Ar*CH*), 125.8 (Ar*C*H), 121.1 (Ar*C^q*–Br), 56.2 (C(O)*C*H), 45.9 (CH*C*(CH_3)$_2$), 44.6 (CH*C*H$_2$), 29.5 (CH$_2$C(*C*H$_3$)$_2$), 29.0 (CH$_2$C(*C*H$_3$)$_2$), 21.4 (C*C*H$_3$).

IR (neat, cm^{-1}): 2953, 2927, 1680, 1486, 1455, 1319, 1206, 1195, 1072, 1006, 816, 731.

HRMS (ESI$^+$): calculated for $C_{21}H_{22}OBr$ (M + H$^+$): 369.0849 Found: 369.0845

rac-(2-(4-Fluorophenyl)-4,4-dimethylcyclopent-2-en-1-yl)(o-tolyl)methanone (**3v**)

Prepared by following the **general procedure A** from 0.1 mmol (**1b**), 0.5 mmol (**2v**), and SmI_2 (25 mol%). The reaction mixture was filtered through a silica gel pad and concentrated in vacuo. The crude product was purified by column chromatography (silica gel 100-200 mesh size; hexane/ethyl acetate) to afford **3v** (21 mg, 0.068 mmol, 68% yield) as a viscous liquid.

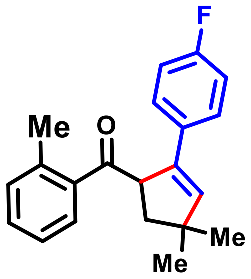

^{1}H NMR (400 MHz, Chloroform-*d*) δ: 7.70 (d, *J* = 7.7 Hz, 1H, Ar*H*), 7.36 (t, *J* = 7.4 Hz, 1H, Ar*H*), 7.30 – 7.17 (m, 4H, Ar*H*), 6.92 – 6.83 (m, 2H, Ar*H*), 6.09 (s, 1H, C*H*C(CH_3)$_2$), 4.86 (dd, *J* = 10.0, 5.2 Hz, 1H, C(O)C*H*), 2.34 (s, 3H, CC*H$_3$*), 2.27 (dd, *J* = 12.9, 10.1 Hz, 1H, CHC*H*$_2$), 1.94 (dd, *J* = 13.0, 5.2 Hz, 1H, CHC*H*$_2$), 1.16 (s, 3H, CH$_2$C(C*H$_3$*)$_2$), 1.16 (s, 3H, CH$_2$C(C*H$_3$*)$_2$).

^{13}C NMR (101 MHz, Chloroform-*d*) δ: 205.7 (*C*=O), 162.1 (d, *J* = 247.5 Hz, Ar*C^q*–F), 140.2 (d, *J* = 2.02 Hz, C(CH_3)$_2$CH), 139.0 (Ar*C^q*), 138.1 (Ar*C^q*), 137.9 (CH*C^q*–Ar), 132.3 (Ar*C*H), 132.1 (d, *J* = 3.03 Hz, Ar*C^q*), 131.6 (Ar*C*H), 128.5 (Ar*C*H), 127.7 (d, *J* = 10.1 Hz, 2 × Ar*C*H), 125.8 (Ar*C*H), 115.4 (d, *J* = 22.22 Hz, 2 × Ar*C*H), 56.4 (C(O)*C*H), 45.8 (CH*C*(CH_3)$_2$), 44.58 (CH*C*H$_2$), 29.6 (CH$_2$C(*C*H$_3$)$_2$), 29.1 (CH$_2$C(*C*H$_3$)$_2$), 21.3 (C*C*H$_3$).

^{19}F NMR (376 MHz, Chloroform-*d*) δ: –115.2 (s).

IR (neat, cm^{-1}): 2954, 2927, 1681, 1600, 1508, 1455, 1226, 1195, 1158, 832, 806, 731.

HRMS (ESI$^+$): calculated for $C_{21}H_{22}FO$ (M + H$^+$): 309.1649 Found: 309.1641

rac-(2-(4-Fluoro-3-methylphenyl)-4,4-dimethylcyclopent-2-en-1-yl)(o-tolyl)methanone (**3w**)

Prepared by following the **general procedure A** from 0.1 mmol (**1b**), 0.5 mmol (**2w**), and SmI_2 (25 mol%). The reaction mixture was filtered through a silica gel pad and concentrated in vacuo. The crude product was purified by column chromatography (silica gel 100-200 mesh size; hexane/ethyl acetate) to afford **3w** (17.1 mg, 0.053 mmol, 53% yield) as a colourless oil.

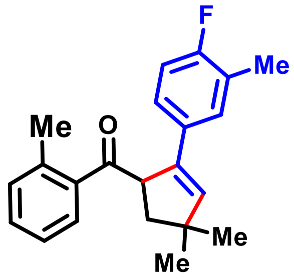

^{1}H NMR (400 MHz, Chloroform-*d*) δ: 7.73 (dd, *J* = 7.6, 1.4 Hz, 1H, Ar*H*) 7.39 (td, *J* = 7.5, 1.4 Hz, 1H, Ar*H*), 7.29 (t, *J* = 7.6 Hz, 1H, Ar*H*), 7.24 (s, 1H, Ar*H*), 7.12 (dd, *J* = 7.4, 2.4 Hz, 1H, Ar*H*), 7.03 (ddd, *J* = 7.8, 4.9, 2.4 Hz, 1H, Ar*H*), 6.84 (t, *J* = 9.0 Hz, 1H, Ar*H*), 6.10 (d, *J* = 1.6 Hz, 1H, C*H*C(CH_3)$_2$), 4.89 (ddd, *J* = 9.9, 5.4, 1.6 Hz, 1H, C(O)C*H*), 2.36 (s, 3H, CCH_3), 2.29 (dd, *J* = 13.0, 9.9 Hz, 1H, CHCH_2), 2.17 (d, *J* = 1.9 Hz, 3H, C(F)CCH_3), 1.98 (dd, *J* = 12.9, 5.3 Hz, 1H, CHCH_2), 1.20 (s, 3H, CH_2C(CH_3)$_2$), 1.19 (s, 3H, CH_2C(CH_3)$_2$).

^{13}C NMR (101 MHz, Chloroform-*d*) δ: 206.0 (*C*=O), 160.8 (d, J^1 = 246.4 Hz, ArC^q–F), 139.9 (d, J^6 = 2.0 Hz, (C(CH_3)$_2$*C*H)), 138.9 (ArC^q), 138.3 (ArC^q), 138.2 (CHC^q–Ar), 132.2 (Ar*C*H), 131.8 (d, J^4 = 3.0 Hz, (ArC^q)), 131.5 (Ar*C*H), 129.3 (d, J^3 = 5.1 Hz, Ar*C*H), 128.4 (Ar*C*H), 125.8 (Ar*C*H), 125.0 (d, J^3 = 8.1 Hz, Ar*C*H), 124.7 (d, J^2 = 18.2 Hz, ArC^q–CH_3), 115.0 (d, J^2 = 23.2 Hz, Ar*C*H), 56.5 (C(O)*C*H), 45.8 (CH*C*(CH_3)$_2$), 44.6 (CH*C*H_2), 29.6 (CH_2C(*C*H_3)$_2$), 29.1 (CH_2C(*C*H_3)$_2$), 21.2 (C*C*H_3), 14.8 (d, J^3 = 3.0 Hz, C^q(F)C^q*C*H_3)

^{19}F NMR (376 MHz, Chloroform-*d*) δ: –119.5 (s).

IR (neat, cm^{-1}): 2953, 2926, 1684, 1501, 1232, 1208, 1118, 815, 755, 731.

HRMS (ESI$^+$): calculated for $C_{22}H_{22}FO$ (M - H$^+$): 321.1660 Found: 321.1667

rac-(2-(3-Fluorophenyl)-4,4-dimethylcyclopent-2-en-1-yl)(o-tolyl)methanone (**3x**)

Prepared by following the **general procedure A** from 0.1 mmol (**1b**), 0.5 mmol (**2x**), and SmI_2 (25 mol%). The reaction mixture was filtered through a silica gel pad and concentrated in vacuo to afford the final product (30.5 mg, 0.099 mmol, 99% yield) as a colourless oil.

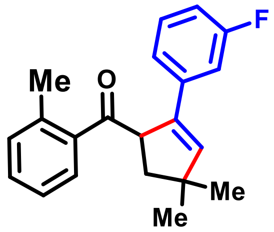

^{1}H NMR (400 MHz, Chloroform-*d*) δ: 7.73 (d, *J* = 7.7 Hz, 1H, Ar*H*), 7.39 (t, *J* = 7.4 Hz, 1H, Ar*H*), 7.33 – 7.22 (m, 2H, Ar*H*), 7.23 – 7.12 (m, 1H, Ar*H*), 7.06 – 6.95 (m, 2H, Ar*H*), 6.86 (td, *J* = 8.5, 2.5 Hz, 1H, Ar*H*), 6.24 – 6.17 (m, 1H, C*H*C(CH_3)$_2$), 4.89 (dd, *J* = 10.1, 5.2 Hz, 1H, C(O)C*H*), 2.38 (s, 3H, CCH_3), 2.31 (dd, *J* = 13.1, 10.0 Hz, 1H, CHCH_2), 1.96 (dd, *J* = 13.0, 5.3 Hz, 1H, CHCH_2), 1.20 (s, 3H, CH_2C(CH_3)$_2$), 1.19 (s, 3H, CH_2C(CH_3)$_2$).

^{13}C NMR (101 MHz, Chloroform-*d*) δ: 205.4 (*C*=O), 163.1 (d, J^1 = 245.4 Hz, ArC^q–F),141.7 (C(CH$_3$)$_2$*C*H), 139.1 (ArC^q), 138.3 (d, J^3 = 8.1 Hz, ArC^q), 138.0 (d, J^4 = 3.0 Hz, CHC^q–Ar), 138.0 (ArC^q), 132.3 (Ar*C*H), 131.6 (Ar*C*H), 130.0 (d, J^3 = 8.1 Hz, Ar*C*H), 128.6 (Ar*C*H), 125.8 (Ar*C*H), 121.8 (d, J^4 = 2.0 Hz, Ar*C*H), 114.1 (d, J^2 = 21.2 Hz, Ar*C*H), 112.9 (d, J^2 = 22.2 Hz, Ar*C*H), 56.2 (C(O)*C*H), 45.8 (CHC^q(CH$_3$)$_2$), 44.5 (CH*C*H$_2$), 29.5 (CH$_2$C(*C*H$_3$)$_2$), 29.0 (CH$_2$C(*C*H$_3$)$_2$), 21.3 (C*C*H$_3$).

IR (neat, cm^{-1}): 2954, 2927, 1682, 1610, 1581, 1444, 1206, 1194, 1154, 855, 778, 754, 730, 686, 665.

^{19}F NMR (376 MHz, Chloroform-*d*) δ: –113.7 (s).

HRMS (ESI$^+$): calculated for $C_{21}H_{22}OF$ (M + H$^+$): 309.1649 Found: 309.1642

rac- (2-(3,5-Difluorophenyl)-4,4-dimethylcyclopent-2-en-1-yl)(o-tolyl)methanone (**3y**)

Prepared by following the **general procedure A** from 0.1 mmol (**1b**), 0.5 mmol (**2y**), and SmI$_2$ (25 mol%). The reaction mixture was filtered through a silica gel pad and concentrated in vacuo. The crude product was purified by column chromatography (silica gel 100-200 mesh size; hexane/ethyl acetate) to afford **3y** (26 mg, 0.08 mmol, 80% yield) as a viscous liquid.

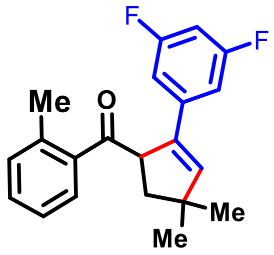

^{1}H NMR (400 MHz, Chloroform-*d*) δ 7.75 (dd, *J* = 7.8, 1.4 Hz, 1H, Ar*H*), 7.41 (td, *J* = 7.5, 1.4 Hz, 1H, Ar*H*), 7.35 – 7.26 (m, 2H, Ar*H*), 6.83 – 6.74 (m, 2H, Ar*H*), 6.62 (tt, *J* = 8.9, 2.3 Hz, 1H, Ar*H*), 6.24 (d, *J* = 1.5 Hz, 1H, C*H*C(CH$_3$)$_2$), 4.86 (ddd, *J* = 10.1, 5.2, 1.5 Hz, 1H, C(O)C*H*), 2.41 (s, 3H, CC*H$_3$*), 2.39 – 2.29 (m, 1H, CHC*H$_2$*), 2.06 – 1.92 (m, 1H, CHC*H$_2$*), 1.20 (s, 3H, CH$_2$C(C*H$_3$*)$_2$), 1.19 (s, 3H, CH$_2$C(C*H$_3$*)$_2$).

^{13}C NMR (101 MHz, Chloroform-*d*) δ 205.0 (*C*=O), 163.3 (d, *J* = 248 Hz, ArC^q–F), 163.1 (d, *J* = 248 Hz, ArC^q–F), 143.0 (Ar*C*H), 139.5 (t, *J* = 10 Hz, ArC^q), 139.2 (ArC^q), 137.4 (t, *J* = 3 Hz, CHC^q–Ar),137.6 (ArC^q), 132.4 (Ar*C*H), 131.8 (Ar*C*H), 128.7 (Ar*C*H), 125.9 (Ar*C*H), 108.9 (d, *J* = 25.3 Hz, Ar*C*H), 108.9 (d, *J* = 12 Hz, Ar*C*H), 102.5 (t, *J* = 26 Hz, Ar*C*H), 56.0 (C(O)*C*H), 45.8 (CHC^q(CH$_3$)$_2$), 44.5 (CH*C*H$_2$), 29.3 (CH$_2$C(*C*H$_3$)$_2$), 28.9 (CH$_2$C(*C*H$_3$)$_2$), 21.4 (C*C*H$_3$).

^{19}F NMR (376 MHz, Chloroform-*d*) δ: –110.4 (s).

IR (neat, cm^{-1}): 2955, 2928, 1681, 1620, 1589, 1438, 1341, 1318, 1207, 1195, 1115, 985, 848, 765, 729, 662.

HRMS (ESI$^+$): calculated for $C_{21}H_{21}OF_2$ (M + H$^+$): 327.1555 Found: 327.1550

rac-(2-(3-Chlorophenyl)-4,4-dimethylcyclopent-2-en-1-yl)(o-tolyl)methanone (**3z**)

Prepared by following the **general procedure A** from 0.1 mmol (**1b**), 0.5 mmol (**2z**), and SmI_2 (25 mol%). The reaction mixture was filtered through a silica gel pad and concentrated in vacuo. The crude product was purified by column chromatography (silica gel 100-200 mesh size; hexane/ethyl acetate) to afford **3z** (32.1 mg, 0.099 mmol, 99% yield) as a colourless oil.

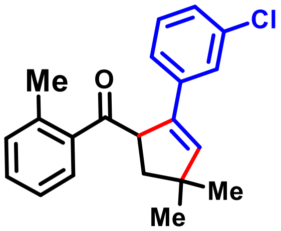

^{1}H NMR (400 MHz, Chloroform-*d*) δ 7.74 (d, J = 7.7 Hz, 1H, Ar*H*), 7.40 (t, J = 7.5 Hz, 1H, Ar*H*), 7.34 – 7.24 (m, 3H, Ar*H*), 7.14 (d, J = 4.2 Hz, 3H, Ar*H*), 6.22 (s, 1H, C*H*C(CH_3)$_2$), 4.90 (dd, J = 10.0, 5.3 Hz, 1H, C(O)C*H*), 2.39 (s, 3H, CCH_3), 2.32 (dd, J = 13.0, 9.9 Hz, 1H, CHCH_2), 1.98 (dd, J = 13.1, 5.4 Hz, 1H, CHCH_2), 1.20 (s, 3H, CH_2C(CH_3)$_2$), 1.20 (s, 3H, CH_2C(CH_3)$_2$).

^{13}C NMR (101 MHz, Chloroform-*d*) δ: 205.5 (*C*=O), 141.8 (C(CH_3)$_2$*C*H), 139.0 (ArC^q), 138.0 (ArC^q), 137.9 (ArC^q), 137.8 (ArC^q), 134.5 (CHC^q–Ar), 132.3 (Ar*C*H), 131.6 (Ar*C*H), 129.7 (Ar*C*H), 128.5 (Ar*C*H), 127.3 (Ar*C*H), 126.3 (Ar*C*H), 125.8 (Ar*C*H), 124.2 (Ar*C*H), 56.2 (C(O)*C*H), 45.8 (CH*C*(CH_3)$_2$), 44.5 (CHCH_2), 29.4 (CH_2C(CH_3)$_2$), 29.0 (CH_2C(CH_3)$_2$), 21.2 (CCH_3).

IR (neat, cm^{-}1): 2954, 2926, 1681, 1593, 1455, 1207, 1195, 858, 780, 753, 732, 686, 664.

HRMS (ESI$^+$): calculated for $C_{21}H_{20}ClO$ (M - H$^+$): 323.1208 Found: 323.1216

rac-(4,4-Dimethyl-2-(4-(trifluoromethyl)phenyl)cyclopent-2-en-1-yl)(o-tolyl)methanone (**3aa**)

Prepared by following the **general procedure A** from 0.1 mmol (**1b**), 0.5 mmol (**2aa**), and SmI_2 (25 mol%). The reaction mixture was filtered through a silica gel pad and concentrated in vacuo to afford the final product (35.4 mg, 0.099 mmol, 99% yield) as a colourless oil.

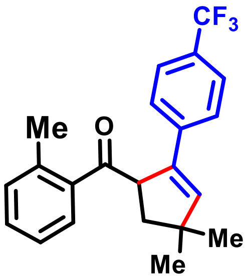

^{1}H NMR (400 MHz, Chloroform-*d*) δ: 7.75 (d, J = 7.7 Hz, 1H, Ar*H*), 7.47 (d, J = 8.2 Hz, 2H, Ar*H*), 7.42 – 7.35 (m, 3H, Ar*H*), 7.32 – 7.22 (m, 2H, Ar*H*), 6.29 (d, J = 1.5 Hz, 1H, C*H*C(CH_3)$_2$), 4.94 (ddd, J = 10.1, 5.4, 1.5 Hz, 1H, C(O)C*H*), 2.37 (s, 3H, CCH_3), 2.32 (dd, J = 13.1, 10.0 Hz, 1H, CHCH_2), 1.97 (dd, J = 13.0, 5.4 Hz, 1H, CHCH_2), 1.20 (s, 2 × 3H, CH_2C(CH_3)$_2$).

^{13}C NMR (101 MHz, Chloroform-*d*) δ: 205.2 (*C*=O), 142.9 (C(CH_3)$_2$*C*H), 139.5 (ArC^q), 139.3 (ArC^q), 137.9 (ArC^q), 137.7 (CHC^q–Ar), 132.4 (Ar*C*H), 131.8 (Ar*C*H), 129.1 (q, J^2 = 33.3 Hz, ArC^q–CF_3), 128.7 (Ar*C*H), 126.3 (2 × Ar*C*H), 125.9 (Ar*C*H), 125.5 (q, J^3 = 4.0 Hz, 2 × Ar*C*H), 124.4 (q, J^1 = 272.7 Hz, ArCF_3), 56.1 (C(O)*C*H), 45.9 (CH*C*(CH_3)$_2$), 44.5 (CHCH_2), 29.4 (CH_2C(CH_3)$_2$), 28.9 (CH_2C(CH_3)$_2$), 21.4 (CCH_3).

^{19}F NMR (376 MHz, Chloroform-*d*) δ: –62.5 (s).

IR (neat, cm^{-}1): 2957, 2929, 1682, 1615, 1456, 1322, 1208, 1195, 1162, 1110, 1067, 1014, 917, 83, 757, 731.

HRMS (ESI⁺): calculated for $C_{22}H_{20}F_3O$ (M - H^+): 357.1472 Found: 357.1474

rac-4-(3,3-Dimethyl-5-(2-methylbenzoyl)cyclopent-1-en-1-yl)benzonitrile (**3ab**)

Prepared by following the **general procedure A** from 0.1 mmol (**1b**), 0.5 mmol (**2ab**), and SmI_2 (25 mol%). The reaction mixture was filtered through a silica gel pad and concentrated in vacuo. The crude product was purified by column chromatography (silica gel 100-200 mesh size; hexane/ethyl acetate) to afford **3ab** (30.3 mg, 0.096 mmol, 96% yield) as a viscous liquid.

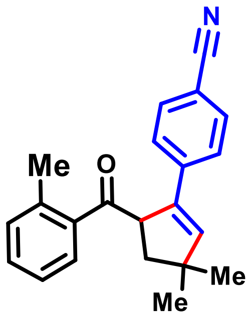

^{1}H NMR (400 MHz, Chloroform-*d*) δ: 7.76 (dd, *J* = 7.7, 1.4 Hz, 1H, Ar*H*), 7.53 – 7.49 (m, 2H, Ar*H*), 7.42 (td, *J* = 7.5, 1.4 Hz, 1H, Ar*H*), 7.38 – 7.27 (m, 4H, Ar*H*), 6.35 (d, *J* = 1.5 Hz, 1H, C*H*C(CH_3)$_2$), 4.94 (ddd, *J* = 10.0, 5.3, 1.5 Hz, 1H, C(O)C*H*), 2.39 (s, 3H, CCH_3), 2.34 (dd, *J* = 13.1, 10.1 Hz, 1H, CHCH_2), 1.97 (dd, *J* = 13.1, 5.3 Hz, 1H, CHCH_2), 1.21 (s, 3H, CH_2C(CH_3)$_2$), 1.20 (s, 3H, CH_2C(CH_3)$_2$).

^{13}C NMR (101 MHz, Chloroform-*d*) δ: 204.8 (*C*=O), 144.2 (C(CH_3)$_2$*C*H), 140.6 (Ar*C*^{q}), 139.3 (Ar*C*^{q}), 137.7 (Ar*C*^{q}), 137.3 (CH*C*^{q}–Ar), 132.5 (Ar*C*H), 132.3 (2 × Ar*CH*), 131.9 (Ar*C*H), 128.7 (Ar*C*H), 126.6 (2 × Ar*CH*), 125.9 (Ar*C*H), 119.2 (*C*N), 110.4 (ArC^q–CN), 55.8 (C(O)*C*H), 46.0 (CH*C*(CH_3)$_2$), 44.4 (CH*C*H_2), 29.3 (CH_2C(*C*H_3)$_2$), 28.8 (CH_2C(*C*H_3)$_2$), 21.5 (C*C*H_3).

IR (neat, cm^{-1}): 2955, 2926, 2224, 1679, 1603, 1455, 1320, 1207, 1196, 836, 753, 731.

HRMS (ESI⁺): calculated for $C_{22}H_{20}ON$ (M - H^+): 314.1550 Found: 314.1554

rac-Methyl 4-(3,3-dimethyl-5-(2-methylbenzoyl)cyclopent-1-en-1-yl)benzoate (**3ac**)

Prepared by following the **general procedure A** from 0.1 mmol (**1b**), 0.25 mmol (**2ac**), and SmI_2 (25 mol%). The reaction mixture was filtered through a silica gel pad and concentrated in vacuo. The crude product was purified by column chromatography (silica gel 100-200 mesh size; hexane/ethyl acetate) to afford **3ac** (30.7 mg, 0.088 mmol, 88% yield) as a colourless oil.

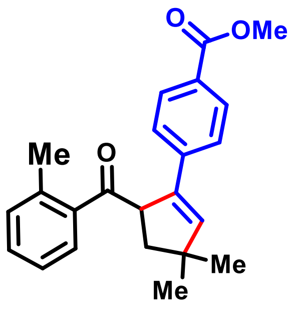

^{1}H NMR (500 MHz, Chloroform-*d*) δ: 7.91 (d, *J* = 8.4 Hz, 2H, Ar*H*), 7.77 (dd, *J* = 7.8, 1.3 Hz, 1H, Ar*H*), 7.41 (td, *J* = 7.5, 1.3 Hz, 1H, Ar*H*), 7.36 – 7.29 (m, 3H, Ar*H*), 7.27 (d, *J* = 3.4 Hz, 1H, Ar*H*), 6.34 (s, 1H, C*H*C(CH_3)$_2$), 4.96 (ddd, *J* = 10.1, 5.3, 1.5 Hz, 1H, C(O)C*H*), 3.89 (s, 3H, C(O)OCH_3), 2.38 (s, 3H, CCH_3), 2.34 (dd, *J* = 13.0, 10.0 Hz, 1H, CHCH_2), 2.00 (dd, *J* = 13.0, 5.3 Hz, 1H, CHCH_2), 1.23 (s, 3H, CH_2C(CH_3)$_2$), 1.22 (s, 3H, CH_2C(CH_3)$_2$).

^{13}C NMR (126 MHz, Chloroform-*d*) δ: 205.3 (*C*=O), 167.1 (*C*(O)OCH_3), 143.0 (Ar*C*^{q}),143.0 (Ar*C*^{q}),140.5 (Ar*C*^{q}), 139.2 (Ar*C*^{q}), 138.4 (CH*C*^{q}–Ar), 137.8 (C(CH_3)$_2$*C*H),

132.4 (Ar*C*H), 131.7 (Ar*C*H), 129.9 (Ar*C*H), 128.7 (Ar*C*H), 128.6 (Ar*C*H), 126.0 (2 × Ar*C*H), 125.9 (Ar*C*H), 56.1 (C(O)*C*H), 52.2 (C(O)OCH_3) , 46.0 (CH*C*(CH_3)$_2$), 44.6 (CH*C*H_2), 29.4 (CH_2C(*C*H_3)$_2$), 29.0 (CH_2C(*C*H_3)$_2$), 21.4 (*C*CH_3).

rac-(2-([1,1'-Biphenyl]-4-yl)-4,4-dimethylcyclopent-2-en-1-yl)(o-tolyl)methanone (**3ad**)

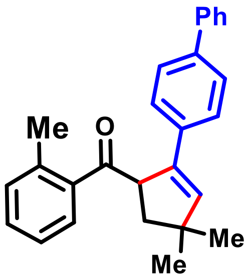

Prepared by following the **general procedure A** from 0.1 mmol (**1b**), 0.5 mmol (**2ad**), and SmI_2 (25 mol%). The reaction mixture was filtered through a silica gel pad and concentrated in vacuo. The crude product was purified by column chromatography (silica gel 100-200 mesh size; hexane/ethyl acetate) to afford **3ad** (36.3 mg, 0.099 mmol, 99% yield) as a viscous liquid.

^{1}H NMR (500 MHz, Chloroform-*d*) δ 7.77 (d, *J* = 7.6 Hz, 1H, Ar*H*), 7.57 – 7.52 (m, 2H, Ar*H*), 7.49 – 7.45 (m, 2H, Ar*H*), 7.43 – 7.34 (m, 5H, Ar*H*), 7.32 – 7.28 (m, 2H, Ar*H*), 7.24 (d, *J* = 2.6 Hz, 1H, Ar*H*), 6.25 (s, 1H, C*H*C(CH_3)$_2$), 4.96 (dd, *J* = 10.1, 5.4 Hz, 1H, C(O)C*H*), 2.39 (s, 3H, CCH_3), 2.32 (dd, *J* = 12.9, 10.0 Hz, 1H, CHCH_2), 1.99 (dd, *J* = 13.1, 5.3 Hz, 1H, CHCH_2), 1.22 (s, 3H, CH_2C(CH_3)$_2$), 1.21 (s, 3H, CH_2C(CH_3)$_2$).

^{13}C NMR (101 MHz, Chloroform-*d*) δ: 205.8 (*C*=O), 141.0 (ArC^q), 140.5 (C(CH_3)$_2$*C*H), 140.0 (ArC^q), 139.1 (ArC^q), 138.5 (ArC^q), 138.1 (ArC^q), 134.9 (CHC^q–Ar), 132.3 (Ar*C*H), 131.5 (Ar*C*H), 128.9 (2 × Ar*C*H), 128.6 (Ar*C*H), 127.4 (Ar*C*H), 127.2 (2 × Ar*C*H), 127.1 (2 × Ar*C*H), 126.5 (2 × Ar*C*H), 125.8 (Ar*C*H), 56.3 (C(O)*C*H), 45.8 (CH*C*(CH_3)$_2$), 44.6 (CH*C*H_2), 29.6 (CH_2C(*C*H_3)$_2$), 29.1 (CH_2C(*C*H_3)$_2$), 21.4 (*C*CH_3).

IR (neat, cm^{-1}): 2957, 2927, 1681, 1599, 1486, 1455, 1285, 1214, 1075, 1006, 837, 750, 696, 665.

HRMS (ESI$^+$): calculated for $C_{27}H_{27}O$ (M + H$^+$): 367.2056 Found: 367.2057

rac-(2-(4-Ethynylphenyl)-4,4-dimethylcyclopent-2-en-1-yl)(o-tolyl)methanone (**3ae**)

Prepared by following the **general procedure A** from 0.1 mmol (**1b**), 0.5 mmol (**2ae**), and SmI_2 (25 mol%). The reaction mixture was filtered through a silica gel pad and concentrated in vacuo. The crude product was purified by column chromatography (silica gel 100-200 mesh size; hexane/ethyl acetate) to afford **3ae** (30.2 mg, 0.096 mmol, 96% yield) as a brown solid.

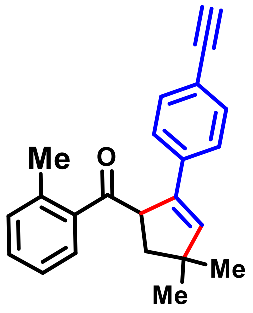

^{1}H NMR (400 MHz, Chloroform-*d*) δ: 7.75 (dd, *J* = 7.7, 1.4 Hz, 1H, Ar*H*), 7.43 – 7.33 (m, 3H, Ar*H*), 7.32 – 7.22 (m, 4H, Ar*H*), 6.24 (d, *J* = 1.6 Hz, 1H, C*H*C(CH_3)$_2$), 4.91 (ddd, *J* = 10.0, 5.3, 1.6 Hz, 1H, C(O)C*H*), 3.07 (s, 1H, alkyne C*H*), 2.38 (s, 3H, CCH_3), 2.32 (dd, *J* = 13.0, 10.0 Hz, 1H, CHCH_2), 1.98 (dd, *J* = 13.0, 5.3 Hz, 1H, CHCH_2), 1.21 (s, 3H, CH_2C(CH_3)$_2$), 1.20 (s, 3H, CH_2C(CH_3)$_2$).

^{13}C NMR (101 MHz, Chloroform-*d*) δ: 205.5 (*C*=O), 141.7 (C(CH$_3$)$_2$*C*H), 139.1 (Ar*C*^{q}), 138.4 (Ar*C*^{q}), 137.9 (Ar*C*^{q}), 136.4 (CH*C*^{q}–Ar), 132.3 (2 × Ar*C*H), 131.6 (Ar*C*H), 128.6 (Ar*C*H), 126.0 (2 × Ar*C*H), 125.8 (Ar*C*H), 120.7 (Ar*C*^{q}–alkyne), 84.0 (alkyne*C*CH), 77.7 (alkyneC*C*H), 56.1 (C(O)*C*H), 45.9 (CH*C*(CH$_3$)$_2$), 44.6 (CH*C*H$_2$), 29.5 (CH$_2$C(*C*H$_3$)$_2$), 29.0 (CH$_2$C(*C*H$_3$)$_2$), 21.4 (C*C*H$_3$) "1 × Ar*C*H not observed".

IR (neat, cm^{-1}): 3291, 2956, 2925, 2864, 1680, 1600, 1455, 1381, 1320, 1285, 1210, 1109, 1014, 916, 836, 753, 733, 664.

HRMS (ESI$^+$): calculated for $C_{23}H_{23}O$ (M + H$^+$): 315.1743 Found: 315.1737

rac-(2-(3-Ethynylphenyl)-4,4-dimethylcyclopent-2-en-1-yl)(o-tolyl)methanone (**3af**)

Prepared by following the **general procedure A** from 0.1 mmol (**1b**), 0.5 mmol (**2af**), and SmI_2 (25 mol%). The reaction mixture was filtered through a silica gel pad and concentrated in vacuo. The crude product was purified by column chromatography (silica gel 100-200 mesh size; hexane/ethyl acetate) to afford **3af** (23.5 mg, 0.075 mmol, 75% yield) as a viscous liquid.

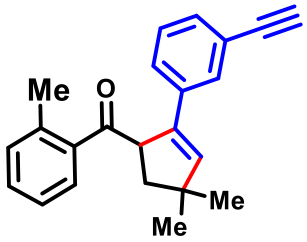

^{1}H NMR (400 MHz, Chloroform-*d*) δ 7.74 (dd, *J* = 7.8, 1.4 Hz, 1H, Ar*H*), 7.44 – 7.36 (m, 2H, Ar*H*), 7.30 (dddd, *J* = 7.6, 4.5, 3.1, 1.3 Hz, 2H, Ar*H*), 7.27 – 7.25 (m, 1H, Ar*H*), 7.24 (d, *J* = 1.7 Hz, 1H, Ar*H*), 7.18 (t, *J* = 7.7 Hz, 1H, Ar*H*), 6.22 (d, *J* = 1.6 Hz, 1H, C*H*C(CH$_3$)$_2$), 4.90 (ddd, *J* = 10.0, 5.3, 1.6 Hz, 1H, C(O)C*H*), 3.01 (s, 1H , alkyne C*H*), 2.38 (s, 3H, CC*H$_3$*), 2.32 (dd, *J* = 13.0, 10.0 Hz, 1H, CHC*H*$_2$), 1.98 (dd, *J* = 13.0, 5.3 Hz, 1H, CHC*H*$_2$), 1.20 (s, 3H, CH$_2$C(C*H$_3$*)$_2$), 1.19 (s, 3H, CH$_2$C(C*H$_3$*)$_2$).

^{13}C NMR (101 MHz, Chloroform-*d*) δ: 205.6 (*C*=O), 141.4 (C(CH$_3$)$_2$*C*H), 139.0 (Ar*C*^{q}), 138.1 (Ar*C*^{q}), 138.1 (Ar*C*^{q}), 136.1 (CH*C*–Ar), 132.3 (Ar*C*H), 131.5 (Ar*C*H), 130.9 (Ar*C*H), 129.9 (Ar*C*H), 128.5 (Ar*C*H), 128.5 (Ar*C*H), 126.5 (Ar*C*H), 125.8 (Ar*C*H), 122.3 (Ar*C*^{q}–alkyne), 83.8 (alkyne*C*CH), 77.2 (alkyneC*C*H), 56.3 (C(O)*C*H), 45.8 (CH*C*(CH$_3$)$_2$), 44.5 (CH*C*H$_2$), 29.5 (CH$_2$C(*C*H$_3$)$_2$), 29.0 (CH$_2$C(*C*H$_3$)$_2$), 21.2 (C*C*H$_3$).

IR (neat, cm^{-1}): 3291, 2953, 2927, 2863, 1681,1 597, 1571, 1478, 1455, 1318, 1286, 1206, 1195, 1009, 894, 860, 792, 752, 731, 688, 665, 649, 625.

HRMS (ESI$^+$): calculated for $C_{23}H_{23}O$ (M + H$^+$): 315.1743 Found: 315.1737

rac-(2-(3,5-Diethynylphenyl)-4,4-dimethylcyclopent-2-en-1-yl)(o-tolyl)methanone (**3ag**)

Prepared by following the **general procedure A** from 0.1 mmol (**1b**), 0.25 mmol (**2ag**), and SmI_2 (25 mol%). The reaction mixture was filtered through a silica gel pad and concentrated in vacuo. The crude product was purified by column chromatography (silica gel 100-200 mesh size; hexane/ethyl acetate) to afford **3ag** (30.12 mg, 0.089 mmol, 89% yield) as a brown viscous liquid.

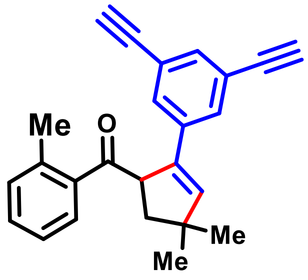

^{1}H NMR (400 MHz, Chloroform-*d*) δ: 7.73 (dd, *J* = 7.8, 1.3 Hz, 1H, Ar*H*), 7.41 (d, *J* = 1.4 Hz, 1H, Ar*H*), 7.40 – 7.36 (m, 3H, Ar*H*), 7.33 – 7.22 (m, 2H, Ar*H*), 6.24 (d, *J* = 1.4 Hz, 1H, C*H*C(CH_3)$_2$), 4.87 (ddd, *J* = 10.0, 5.3, 1.5 Hz, 1H, C(O)C*H*), 3.02 (s, 2H, two alkyne C*H*), 2.39 (s, 3H, CC*H*$_3$), 2.32 (dd, *J* = 13.0, 10.0 Hz, 1H, CHC*H*$_2$), 1.98 (dd, *J* = 13.0, 5.3 Hz, 1H, CHC*H*$_2$), 1.19 (s, 2 × 3H, CH_2C(C*H*$_3$)$_2$).

^{13}C NMR (101 MHz, Chloroform-*d*) δ: 205.3 (*C*=O), 142.3 (C(CH_3)$_2$*C*H), 139.0 (Ar*C*^{q}), 137.9 (Ar*C*^{q}), 137.4 (Ar*C*^{q}), 136.6 (CH*C*^{q}–Ar), 134.1 (Ar*C*H), 132.3 (Ar*C*H), 131.6 (Ar*C*H), 130.1 (2 × Ar*C*H), 128.5 (Ar*C*H), 125.8 (Ar*C*H), 122.7 (2 × Ar*C*^{q}–alkyne), 82.8 (2 × alkyne*C*CH), 77.9 (2 × alkyneC*C*H), 56.2 (C(O)*C*H), 45.8 (CH*C*(CH_3)$_2$), 44.5 (CH*C*H$_2$), 29.4 (CH_2C(*C*H$_3$)$_2$), 28.9 (CH_2C(*C*H$_3$)$_2$), 21.2 (C*C*H$_3$).

IR (neat, cm^{-1}): 3291, 2956, 2928, 1683, 1583, 1456, 1210, 664.

HRMS (ESI$^+$): calculated for $C_{25}H_{23}O$ (M + H$^+$): 339.1743 Found: 339.1731

rac-(2-(6-Methoxynaphthalen-2-yl)-4,4-dimethylcyclopent-2-en-1-yl)(o tolyl)methanone (**3ah**)

Prepared by following the **general procedure A** from 0.1 mmol (**1b**), 0.25 mmol (**2ah**), and SmI_2 (25 mol%). The reaction mixture was filtered through a silica gel pad and concentrated in vacuo. The crude product was purified by column chromatography (silica gel 100-200 mesh size; hexane/ethyl acetate) to afford **3ah** (30 mg, 0.081 mmol, 81% yield) as a brown solid.

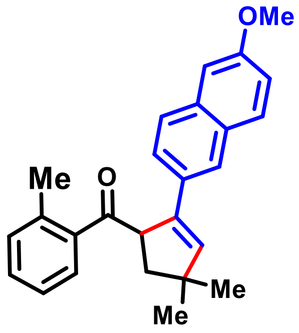

^{1}H NMR (400 MHz, Chloroform-*d*) δ: 7.80 (dd, *J* = 7.7, 1.5 Hz, 1H, Ar*H*), 7.63 (d, *J* = 8.6 Hz, 1H, Ar*H*), 7.57 (dd, *J* = 8.6, 1.8 Hz, 1H, Ar*H*), 7.51 – 7.47 (m, 1H, Ar*H*), 7.45 – 7.39 (m, 2H, Ar*H*), 7.35 – 7.30 (m, 1H, Ar*H*), 7.26 (s, 1H, Ar*H*), 7.06 (d, *J* = 7.4 Hz, 2H, Ar*H*), 6.30 (d, *J* = 1.5 Hz, 1H, C*H*C(CH_3)$_2$), 5.03 (ddd, *J* = 9.9, 5.3, 1.5 Hz, 1H, C(O)C*H*), 3.89 (s, 3H, OC*H*$_3$), 2.40 – 2.35 (4H, CC*H*$_3$ and CHC*H*$_2$), 2.05 (dd, *J* = 12.9, 5.3 Hz, 1H, CHC*H*$_2$), 1.26 (s, 3H, CH_2C(C*H*$_3$)$_2$), 1.23 (s, 3H, CH_2C(C*H*$_3$)$_2$).

^{13}C NMR (101 MHz, Chloroform-*d*) δ: 206.3 (*C*=O), 157.8 (Ar*C*^{q}–OCH$_3$), 140.1 (C(CH_3)$_2$*C*H), 139.1 (Ar*C*^{q}), 138.9 (Ar*C*^{q}), 138.5 (Ar*C*^{q}), 133.9 (Ar*C*^{q}), 132.2 (Ar*C*H), 131.5 (Ar*C*H), 131.1 (CH*C*^{q}–Ar), 129.7 (Ar*C*H), 129.0 (Ar*C*^{q}), 128.5 (Ar*C*H), 127.0 (Ar*C*H), 125.8 (Ar*C*H), 125.1 (Ar*C*H), 124.6 (Ar*C*H), 119.0 (Ar*C*H), 105.9 (Ar*C*H), 56.6 (O*C*H$_3$), 55.5 (C(O)*C*H), 45.9 (CH*C*(CH_3)$_2$), 44.7 (CH*C*H$_2$), 29.7 (CH_2C(*C*H$_3$)$_2$), 29.2 (CH_2C(*C*H$_3$)$_2$), 21.2 (C*C*H$_3$).

IR (neat, cm^{-1}): 2952, 2928, 2862, 1682, 1627, 1600, 1483, 1456, 1388, 1343, 1260, 1208, 1164, 1121, 1030, 911, 850, 805, 753, 733, 657.

HRMS (ESI$^+$): calculated for $C_{26}H_{25}O_2$ (M - H$^+$): 369.1860 Found: 369.1860

rac-(4,4-Dimethyl-2-(phenanthren-9-yl)cyclopent-2-en-1-yl)(o-tolyl)methanone (**3ai**)

Prepared by following the **general procedure A** from 0.1 mmol (**1b**), 0.25 mmol (**2ai**), and SmI_2 (25 mol%). The reaction mixture was filtered through a silica gel pad and concentrated in vacuo. The crude product was purified by column chromatography (silica gel 100-200 mesh size; hexane/ethyl acetate) to afford **3ai** (37.9 mg, 0.097 mmol, 97% yield) as a white solid.

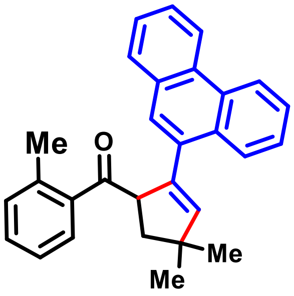

^{1}H NMR (500 MHz, Chloroform-*d*) δ: 8.65 (dd, *J* = 7.2, 2.0 Hz, 1H, Ar*H*), 8.59 (d, *J* = 8.2 Hz, 1H, Ar*H*), 8.25 – 8.17 (m, 1H, Ar*H*), 7.74 (d, *J* = 7.8 Hz, 1H, Ar*H*), 7.65 – 7.56 (m, 4H, Ar*H*), 7.53 (t, *J* = 7.4 Hz, 1H, Ar*H*), 7.32 (d, *J* = 7.7 Hz, 1H, Ar*H*), 7.08 (t, *J* = 7.4 Hz, 1H, Ar*H*), 6.92 (t, *J* = 7.8 Hz, 2H, Ar*H*), 5.96 (d, *J* = 1.9 Hz, 1H, C*H*C(CH$_3$)$_2$), 5.16 (ddd, *J* = 8.6, 6.2, 1.8 Hz, 1H, C(O)C*H*), 2.35 (dd, *J* = 12.9, 8.8 Hz, 1H, CHC*H$_2$*), 2.29 (dd, *J* = 12.9, 6.2 Hz, 1H, CHC*H$_2$*), 2.12 (s, 3H, CC*H$_3$*), 1.42 (s, 3H, CH$_2$C(C*H$_3$*)$_2$), 1.33 (s, 3H, CH$_2$C(C*H$_3$*)$_2$).

^{13}C NMR (101 MHz, Chloroform-*d*) δ: 206.0 (*C*=O), 144.8 (C(CH$_3$)$_2$*C*H), 139.0 (Ar*C^q*), 138.3 (Ar*C^q*), 137.7 (Ar*C^q*), 133.8 (CH*C^q*–Ar), 131.5 (Ar*C*H), 131.0 (Ar*C^q*), 130.7 (Ar*C*H), 130.6 (Ar*C^q*), 129.9 (Ar*C^q*), 128.7 (Ar*C*H), 128.0 (Ar*C*H), 126.7 (Ar*C*H), 126.6 (Ar*C*H), 126.6 (Ar*C*H), 126.5 (Ar*C*H), 126.5 (Ar*C*H), 126.4 (Ar*C*H), 125.2 (Ar*C*H), 123.0 (Ar*C*H), 122.5 (Ar*C*H), 59.6 (C(O)*C*H), 46.1 (CH*C*(CH$_3$)$_2$), 43.9 (CH*C*H$_2$), 29.4 (CH$_2$C(*C*H$_3$)$_2$), 29.4 (CH$_2$C(*C*H$_3$)$_2$), 20.8 (C*C*H$_3$) “1 × Ar*C^q* not observed”.

IR (neat, cm^{-1}): 2957, 2926, 1675, 1448, 1211, 1025, 907, 886, 863, 856, 760, 740, 722, 693, 665.

HRMS (ESI$^+$): calculated for $C_{29}H_{27}O$ (M + H$^+$): 391.2056 Found: 391.2050

rac-(4,4-Dimethyl-2-(pyren-1-yl)cyclopent-2-en-1-yl)(o-tolyl)methanone (**3aj**)

Prepared by following the **general procedure A** from 0.1 mmol (**1b**), 0.25 mmol (**2aj**), and SmI_2 (25 mol%). The reaction mixture was filtered through a silica gel pad and concentrated in vacuo. The crude product was purified by column chromatography (silica gel 100-200 mesh size; hexane/ethyl acetate) to afford **3aj** (37.3 mg, 0.090 mmol, 90% yield) as a brown solid.

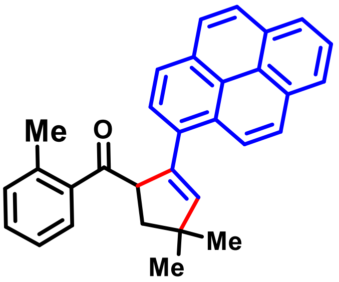

^{1}H NMR (400 MHz, Chloroform-*d*) δ: 8.51 (d, *J* = 9.2 Hz, 1H, Ar*H*), 8.30 – 8.13 (m, 2H, Ar*H*), 8.09 (d, *J* = 9.3 Hz, 1H, Ar*H*), 8.05 – 7.95 (m, 4H, Ar*H*), 7.83 (d, *J* = 7.9 Hz, 1H, Ar*H*), 7.41 (dd, *J* = 7.8, 1.4 Hz, 1H, Ar*H*), 7.12 (td, *J* = 7.5, 1.4 Hz, 1H, Ar*H*), 7.03 – 6.92 (m, 2H,

Ar*H*), 6.03 (d, *J* = 1.8 Hz, 1H, C*H*C(CH_3)$_2$), 5.27 (ddd, *J* = 8.6, 6.4, 1.9 Hz, 1H, C(O)C*H*), 2.41 (dd, *J* = 12.8, 9.0 Hz, 1H, CHC*H*$_2$), 2.30 (dd, *J* = 12.8, 6.4 Hz, 1H, CHC*H*$_2$), 2.06 (s, 3H, CC*H*$_3$), 1.46 (s, 3H, CH_2C(C*H*$_3$)$_2$), 1.35 (s, 3H, CH_2C(C*H*$_3$)$_2$).

^{13}C NMR (101 MHz, Chloroform-*d*) δ: 206.2 (*C*=O), 145.6 (C(CH_3)$_2$*C*H), 139.0 (Ar*C*^{q}), 138.5 (Ar*C*^{q}), 137.9 (Ar*C*^{q}), 133.1 (CH*C*^{q}–Ar), 131.6 (Ar*C*H), 131.6 (Ar*C*^{q}), 131.2 (Ar*C*^{q}), 130.8 (Ar*C*H), 130.4 (Ar*C*^{q}), 129.0 (Ar*C*^{q}), 128.1 (Ar*C*H), 127.6 (Ar*C*H), 127.5 (Ar*C*H), 127.3 (Ar*C*H), 126.1 (Ar*C*H), 126.0 (Ar*C*H), 125.4 (Ar*C*H), 125.4 (Ar*C*H), 125.1 (Ar*C*H), 125.1 (Ar*C*^{q}), 125.0 (Ar*C*^{q}), 124.9 (Ar*C*H), 124.5 (Ar*C*H), 60.1 (C(O)*C*H), 46.5 (CH*C*(CH_3)$_2$), 44.4 (CH*C*H_2), 29.5 (CH_2C(*C*H_3)$_2$), 29.3 (CH_2C(*C*H_3)$_2$), 20.8 (C*C*H_3).

IR (neat, cm^{-1}): 2950, 2924, 1675, 1453, 1207, 1188, 841, 825, 790, 773, 757, 732, 718, 681, 627.

HRMS (ESI$^+$): calculated for $C_{31}H_{25}O$ (M - H^+): 413.1911 Found: 413.1909

rac-(4,4-Dimethyl-2-(thiophen-3-yl)cyclopent-2-en-1-yl)(o-tolyl)methanone (**3ak**)

Prepared by following the **general procedure A** from 0.1 mmol (**1b**), 0.5 mmol (**2ak**), and SmI_2 (40 mol%). The reaction mixture was filtered through a silica gel pad and concentrated in vacuo. The crude product was purified by column chromatography (silica gel 100-200 mesh size; hexane/ethyl acetate) to afford **3ak** (17.5 mg, 0.059 mmol, 59% yield) as a yellow oil.

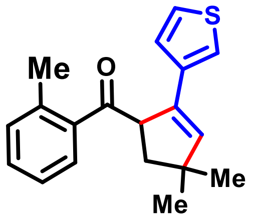

^{1}H NMR (400 MHz, Chloroform-*d*) δ: 7.72 (dd, *J* = 7.7, 1.4 Hz, 1H, Ar*H*), 7.39 (td, *J* = 7.5, 1.4 Hz, 1H, Ar*H*), 7.32 – 7.27 (m, 2H, Ar*H*), 7.22 (dd, *J* = 5.0, 2.8 Hz, 1H, Ar*H*), 7.18 (dd, *J* = 5.1, 1.4 Hz, 1H, Ar*H*), 6.83 (dd, *J* = 2.8, 1.4 Hz, 1H, Ar*H*), 6.07 (d, *J* = 1.6 Hz, 1H, C*H*C(CH_3)$_2$), 4.81 (ddd, *J* = 10.0, 5.0, 1.6 Hz, 1H, C(O)C*H*), 2.41 (s, 3H, CC*H*$_3$), 2.27 (dd, *J* = 13.1, 10.0 Hz, 1H, CHC*H*$_2$), 1.96 (dd, *J* = 13.1, 5.0 Hz, 1H, CHC*H*$_2$), 1.20 (s, 3H, CH_2C(C*H*$_3$)$_2$), 1.18 (s, 3H, CH_2C(C*H*$_3$)$_2$).

^{13}C NMR (101 MHz, Chloroform-*d*) δ: 206.1 (*C*=O), 139.9 (C(CH_3)$_2$*C*H), 138.9 (Ar*C*^{q}), 138.3 (Ar*C*^{q}), 137.9 (Ar*C*^{q}), 134.2 (CH*C*^{q}–Ar), 132.3 (Ar*C*H), 131.5 (Ar*C*H), 128.5 (Ar*C*H), 126.4 (Ar*C*H), 125.8 (2 × Ar*C*H), 120.7 (Ar*C*H), 57.3 (C(O)*C*H), 45.9 (CH*C*(CH_3)$_2$), 44.5 (CH*C*H_2), 29.8 (CH_2C(*C*H_3)$_2$), 29.2 (CH_2C(*C*H_3)$_2$), 21.3 (C*C*H_3).

HRMS (ESI$^+$): calculated for $C_{19}H_{19}OS$ (M - H^+): 295.1162 Found: 295.1163

rac-(4,4-Dimethyl-2-phenylcyclopent-2-en-1-yl)(2-ethylphenyl)methanone (**3al**)

Prepared by following the **general procedure A** from 0.1 mmol (**1e**), 0.5 mmol (**2a**), and SmI_2 (25 mol%). The reaction mixture was filtered through a silica gel pad and concentrated in vacuo to afford the final product (30.1 mg, 0.099 mmol, 99% yield) as a viscous liquid.

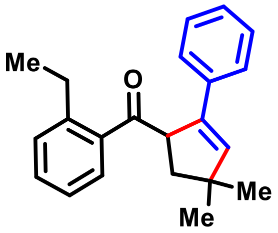

^{1}H NMR (400 MHz, Chloroform-*d*) δ: 7.75 – 7.70 (m, 1H, Ar*H*), 7.43 (td, *J* = 7.8, 7.4, 1.4 Hz, 1H, Ar*H*), 7.30 (d, *J* = 7.5 Hz, 4H, Ar*H*), 7.26 – 7.20 (m, 2H, Ar*H*), 7.19 – 7.14 (m, 1H, Ar*H*), 6.20 (d, *J* = 1.6 Hz, 1H, C*H*C(CH_3)$_2$), 4.94 (ddd, *J* = 9.9, 5.3, 1.6 Hz, 1H, C(O)C*H*), 2.70 (q, *J* = 7.4 Hz, 2H, CC*H*$_2$CH$_3$), 2.30 (dd, *J* = 13.0, 9.9 Hz, 1H, CHC*H*$_2$), 2.00 (dd, *J* = 13.0, 5.3 Hz, 1H, CHC*H*$_2$), 1.22 (s, 3H, CH$_2$C(C*H*$_3$)$_2$), 1.20 (s, 3H, CH$_2$C(C*H*$_3$)$_2$), 1.16 (t, *J* = 7.5 Hz, 3H CCH$_2$C*H*$_3$).

^{13}C NMR (101 MHz, Chloroform-*d*) δ: 206.0 (*C*=O), 145.1 (Ar*C*^{q}–C$_2$H$_5$), 140.4 (C(CH$_3$)$_2$*C*H), 139.0 (Ar*C*^{q}), 138.1 (Ar*C*^{q}), 135.8 (CH*C*^{q}–Ar), 131.6 (Ar*C*H), 130.8 (Ar*C*H), 128.5 (2 × Ar*C*H), 128.4 (Ar*C*H), 127.3 (Ar*C*H), 126.2 (2 × Ar*C*H), 125.7 (Ar*C*H), 56.6 (C(O)*C*H), 45.8 (CH*C*(CH$_3$)$_2$), 44.5 (CH*C*H$_2$), 29.6 (CH$_2$C(*C*H$_3$)$_2$), 29.1 (CH$_2$C(*C*H$_3$)$_2$), 27.1 (C*C*H$_2$CH$_3$), 16.2 (CCH$_2$*C*H$_3$).

IR (neat, cm^{-1}): 2953, 2930, 2864, 1682, 1445, 1320, 1203, 1008, 918, 753, 691.

HRMS (ESI$^+$): calculated for $C_{22}H_{25}O$ (M + H$^+$): 305.1900 Found: 305.1895

rac-(4,4-Dimethyl-2-phenylcyclopent-2-en-1-yl)(3-fluoro-2-methylphenyl)methanone (**3am**)

Prepared by following the **general procedure A** from 0.1 mmol (**1f**), 0.5 mmol (**2a**), and SmI_2 (25 mol%). The reaction mixture was filtered through a silica gel pad and concentrated in vacuo to afford the final product (30.5 mg, 0.099 mmol, 99% yield) as a colourless oil.

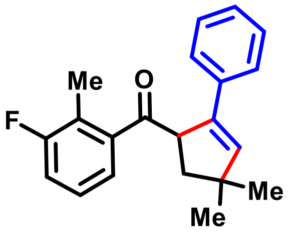

^{1}H NMR (400 MHz, Chloroform-*d*) δ: 7.52 (d, *J* = 7.7 Hz, 1H, Ar*H*), 7.35 – 7.27 (m, 5H, Ar*H*), 7.25 – 7.16 (m, 2H, Ar*H*), 6.23 (d, *J* = 1.6 Hz, 1H, C*H*C(CH$_3$)$_2$), 4.93 (ddd, *J* = 9.9, 5.3, 1.6 Hz, 1H, C(O)C*H*), 2.33 (dd, *J* = 13.0, 9.9 Hz, 1H, CHC*H*$_2$), 2.26 (d, *J* = 2.4 Hz, 3H, CC*H*$_3$), 2.03 (dd, *J* = 13.0, 5.4 Hz, 1H, CHC*H*$_2$), 1.26 (s, 3H, CH$_2$C(C*H*$_3$)$_2$), 1.24 (s, 3H, CH$_2$C(C*H*$_3$)$_2$).

^{13}C NMR (101 MHz, Chloroform-*d*) δ: 205.4 (d, J^4 = 3.0, *C*=O), 161.9 (d, J^1 = 246.4, (Ar*C*^{q}–F)), 141.0 (d, J^3 = 3.0, Ar*C*^{q}–C(O)), 140.6 (C(CH$_3$)$_2$*C*H), 138.7 (Ar*C*^{q}), 135.7 (CH*C*^{q}–Ar), 128.6 (2 × Ar*C*H), 127.4 (Ar*C*H), 126.7 (d, J^3 = 8.1, Ar*C*H) 126.1 (2 × Ar*C*H), 125.6 (d, J^2 = 17.2, (Ar*C*^{q}–CH$_3$)), 123.6 (d, J^4 = 3.0, (Ar*C*H)), 118.1 (d, J^2 = 24.2, (Ar*C*H)), 56.9 (C(O)*C*H), 45.8 (CH*C*(CH$_3$)$_2$), 44.3 (CH*C*H$_2$), 29.6 (CH$_2$C(*C*H$_3$)$_2$), 29.1 (CH$_2$C(*C*H$_3$)$_2$), 11.6 (d, J^3 = 6.1, C*C*H$_3$).

^{19}F NMR (376 MHz, Chloroform-*d*) δ: –114.8 (s).

IR (neat, cm⁻¹): 2954, 2930, 1687, 1456, 1242, 1196, 1000, 763, 732, 693.

HRMS (ESI⁺): calculated for $C_{21}H_{22}FO$ (M + H⁺): 309.1649 Found: 309.1634

rac-(4,4-Dimethyl-2-phenylcyclopent-2-en-1-yl)(2,3-dimethylphenyl)methanone (**3an**)

Prepared by following the **general procedure A** from 0.1 mmol (**1g**), 0.5 mmol (**2a**), and SmI_2 (25 mol%). The reaction mixture was filtered through a silica gel pad and concentrated in vacuo. The crude product was purified by column chromatography (silica gel 100-200 mesh size; hexane/ethyl acetate) to afford **3an** (30.1 mg, 0.099 mmol, 99% yield) as a viscous liquid.

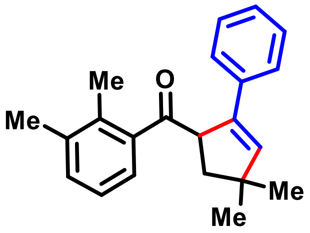

¹H NMR (400 MHz, Chloroform-*d*) δ: 7.47 (d, *J* = 7.7 Hz, 1H, Ar*H*), 7.29 (d, *J* = 8.2 Hz, 2H, Ar*H*), 7.25 – 7.19 (m, 3H, Ar*H*), 7.16 (t, *J* = 7.4 Hz, 2H, Ar*H*), 6.17 (s, 1H, C*H*C(CH_3)$_2$), 4.88 (dd, *J* = 9.9, 5.4 Hz, 1H, C(O)C*H*), 2.29 (s, 3H, *m*-CC*H*$_3$), 2.27 – 2.23 (m, 1H, CHC*H*$_2$), 2.17 (s, 3H, *o*-CC*H*$_3$), 2.00 (dd, *J* = 13.0, 5.4 Hz, 1H, CHC*H*$_2$), 1.22 (s, 3H, CH_2C(C*H*$_3$)$_2$), 1.18 (s, 3H, CH_2C(C*H*$_3$)$_2$).

¹³C NMR (101 MHz, Chloroform-*d*) δ: 207.4 (*C*=O), 140.5 (C(CH_3)$_2$*C*H), 140.1 (Ar*C*^{q}), 139.0 (Ar*C*^{q}), 138.6 (Ar*C*^{q}), 136.3 (CH*C*^{q}–Ar), 135.9 (Ar*C*^{q}), 132.6 (Ar*C*H), 128.5 (2 × Ar*C*H), 127.3 (Ar*C*H), 126.2 (2 × Ar*C*H), 125.4 (Ar*C*H), 125.2 (Ar*C*H), 57.3 (C(O)*C*H), 45.7 (CH*C*(CH_3)$_2$), 44.3 (CH*C*H$_2$), 29.6 (CH_2C(*C*H$_3$)$_2$), 29.1 (CH_2C(*C*H$_3$)$_2$), 20.7 (*m*-C*C*H$_3$), 16.5 (*o*-C*C*H$_3$).

IR (neat, cm⁻¹): 2951, 2925, 2862, 1685, 1445, 1319, 1229, 1100, 761, 727, 692.

HRMS (ESI⁺): calculated for $C_{22}H_{25}O$ (M + H⁺): 305.1900 Found: 305.1894

rac-(2-Chloro-6-methylphenyl)(4,4-dimethyl-2-phenylcyclopent-2-en-1-yl)methanone (**3ao**)

Prepared by following the **general procedure A** from 0.1 mmol (**1h**), 0.5 mmol (**2a**), and SmI_2 (25 mol%). The reaction mixture was filtered through a silica gel pad and concentrated in vacuo to afford the final product (32.2 mg, 0.099 mmol, 99% yield) as a yellow solid.

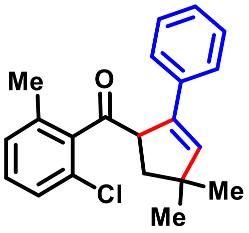

¹H NMR (400 MHz, Chloroform-*d*) δ 7.25 – 7.21 (m, 2H, Ar*H*), 7.17 – 7.05 (m, 5H, Ar*H*), 6.85 (d, *J* = 7.5 Hz, 1H, Ar*H*), 5.97 (d, *J* = 1.8 Hz, 1H, C*H*C(CH_3)$_2$), 4.97 (ddd, *J* = 9.5, 3.8, 1.8 Hz, 1H, C(O)C*H*), 2.44 (dd, *J* = 13.3, 3.8 Hz, 1H, CHC*H*$_2$), 2.20 – 2.07 (m, 1H, CHC*H*$_2$), 1.83 (s, 3H, CC*H*$_3$), 1.29 (s, 3H, CH_2C(C*H*$_3$)$_2$), 1.20 (s, 3H, CH_2C(C*H*$_3$)$_2$).

¹³C NMR (101 MHz, Chloroform-*d*) δ: 206.6 (*C*=O), 141.8 (C(CH_3)$_2$*C*H), 140.7 (Ar*C*^{q}), 138.6 (Ar*C*^{q}), 137.5 (Ar*C*^{q}), 136.1 (CH*C*^{q}–Ar), 130.1 (Ar*C*H), 130.0 (Ar*C*^{q}–Cl), 129.2 (Ar*C*H), 128.1 (2 × Ar*C*H), 127.3 (Ar*C*H), 127.1 (Ar*C*H), 126.5 (2 × Ar*C*H), 59.0 (C(O)*C*H), 45.2 (CH*C*(CH_3)$_2$), 42.0 (CH*C*H$_2$), 30.2 (CH_2C(*C*H$_3$)$_2$), 29.2 (CH_2C(*C*H$_3$)$_2$), 19.7 (C*C*H$_3$).

IR (neat, cm^{-1}): 2953, 2928, 1697, 1445, 1195, 773, 751, 694.

HRMS (ESI$^+$): calculated for $C_{21}H_{22}OCl$ (M + H$^+$): 325.1354 Found: 325.1364

rac-(4,4-Dimethyl-2-phenylcyclopent-2-en-1-yl)(3,5-dimethylphenyl)methanone (**3ap**)

Prepared by following the **general procedure A** from 0.1 mmol (**1i**), 0.5 mmol (**2a**), and SmI_2 (40 mol%). The reaction mixture was filtered through a silica gel pad and concentrated in vacuo. The crude product was purified by column chromatography (silica gel 100-200 mesh size; hexane/ethyl acetate) to afford **3ap** (14.7 mg, 0.049 mmol, 49% yield) as a colourless oil.

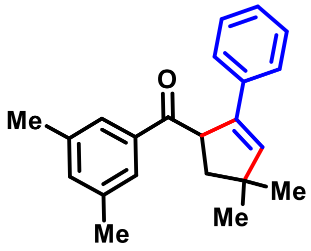

^{1}H NMR (400 MHz, Chloroform-*d*) δ: 7.65 (d, *J* = 1.5 Hz, 2H, Ar*H*), 7.30 – 7.26 (m, 2H, Ar*H*), 7.25 – 7.20 (m, 3H, Ar*H*), 7.18 – 7.15 (m, 1H, Ar*H*), 6.24 (d, *J* = 1.4 Hz, 1H, C*H*C(CH_3)$_2$), 5.01 (ddd, *J* = 10.2, 5.3, 1.5 Hz, 1H, C(O)C*H*), 2.40 (s, 6H, 2 × CC*H*$_3$), 2.38 (dd, *J* = 13.0, 10.2 Hz, 1H, CHC*H*$_2$), 1.93 (dd, *J* = 13.0, 5.3 Hz, 1H, CHC*H*$_2$), 1.22 (s, 3H, CH_2C(C*H*$_3$)$_2$), 1.18 (s, 3H, CH_2C(C*H*$_3$)$_2$).

^{13}C NMR (101 MHz, Chloroform-*d*) δ: 202.1 (*C*=O), 140.2 (C(CH_3)$_2$*C*H), 138.6 (Ar*C*^{q}), 138.5 (2 × Ar*C*^{q}), 136.8 (Ar*C*^{q}), 135.8 (CH*C*^{q}–Ar), 134.9 (Ar*C*H), 128.5 (2 × Ar*C*H), 127.2 (Ar*C*H), 126.7 (2 × Ar*C*H), 126.0 (2 × Ar*C*H), 53.5 (C(O)*C*H), 45.9 (CH*C*(CH_3)$_2$), 45.0 (CH*C*H$_2$), 29.6 (CH_2C(*C*H$_3$)$_2$), 29.1 (CH_2C(*C*H$_3$)$_2$), 21.5 (2 × C*C*H$_3$).

IR (neat, cm^{-1}): 2954, 2924, 1677, 1599, 1446, 1294, 1177, 1157, 859, 754, 692, 678.

HRMS (ESI$^+$): calculated for $C_{22}H_{25}O$ (M + H$^+$): 305.1900 Found: 305.1892

rac-(4,4-Dimethyl-2-phenylcyclopent-2-en-1-yl)(mesityl)methanone (**3aq**)

Prepared by following the **general procedure A** from 0.1 mmol (**1j**), 0.5 mmol (**2a**), and SmI_2 (25 mol%). The reaction mixture was filtered through a silica gel pad and concentrated in vacuo to afford the final product (31.5 mg, 0.099 mmol, 99% yield) as a yellow solid.

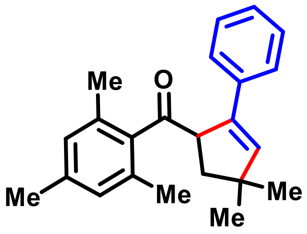

An 81% yield was obtained when 15 mol% catalyst was used.

^{1}H NMR (400 MHz, Chloroform-*d*) δ: 7.22 – 7.17 (m, 2H, Ar*H*), 7.16 – 7.07 (m, 3H, Ar*H*), 6.71 (s, 2H, Ar*H*), 5.97 (d, *J* = 1.8 Hz, 1H, C*H*C(CH_3)$_2$), 4.73 (ddd, *J* = 9.4, 4.2, 1.8 Hz, 1H, C(O)C*H*), 2.35 (dd, *J* = 13.1, 4.2 Hz, 1H, CHC*H*$_2$), 2.24 (s, 3H, CC*H*$_3$), 2.12 (dd, *J* = 13.1, 9.4 Hz, 1H, CHC*H*$_2$), 2.01 (s, 2 × 3H, CC*H*$_3$), 1.32 (s, 3H, CH_2C(C*H*$_3$)$_2$), 1.19 (s, 3H, CH_2C(C*H*$_3$)$_2$).

^{13}C NMR (101 MHz, Chloroform-*d*) δ: 211.0 (*C*=O), 141.5 (C(CH_3)$_2$*C*H), 139.7 (Ar*C*^{q}), 139.3 (Ar*C*^{q}), 138.9 (Ar*C*^{q}), 136.2 (CH*C*^{q}–Ar), 134.1 (2 × Ar*C*^{q}), 129.0 (2 × Ar*C*H), 128.1 (2 × Ar*C*H), 127.2 (Ar*C*H), 126.6 (2 × Ar*C*H), 59.4 (C(O)*C*H), 45.3 (CH*C*(CH_3)$_2$), 42.8 (CH*C*H$_2$), 30.2 (CH_2C(*C*H$_3$)$_2$), 29.3 (CH_2C(*C*H$_3$)$_2$), 21.2 (C*C*H$_3$), 20.1 (2 × C*C*H$_3$).

IR (neat, cm^{-1}): 2953, 2924, 1688, 1609, 1444, 1152, 914, 848, 755, 694.

HRMS (ESI$^+$): calculated for $C_{23}H_{25}O$ (M - H$^+$): 317.1911 Found: 317.1911

rac-(4,4-Dimethyl-2-phenylcyclopent-2-en-1-yl)(2,3,4,5,6 pentamethylphenyl)methanone (**3ar**)

Prepared by following the **general procedure A** from 0.1 mmol (**1k**), 0.5 mmol (**2a**), and SmI_2 (25 mol%). The reaction mixture was filtered through a silica gel pad and concentrated in vacuo. The crude product was purified by column chromatography (silica gel 100-200 mesh size; hexane/ethyl acetate) to afford **3ar** (28.8 mg, 0.083 mmol, 83% yield) as a white solid.

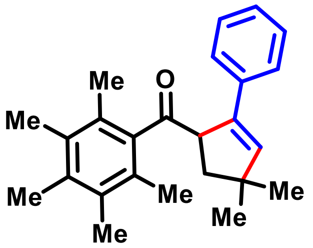

^{1}H NMR (400 MHz, Chloroform-*d*) δ: 7.12 – 6.99 (m, 5H, Ar*H*), 5.89 (d, *J* = 1.9 Hz, 1H, C*H*C(CH_3)$_2$), 4.60 (ddd, *J* = 9.5, 3.7, 1.9 Hz, 1H, C(O)C*H*), 2.50 (dd, *J* = 13.2, 3.5 Hz, 1H, CHC*H*$_2$), 2.24 – 2.19 (m, 1H, CHC*H*$_2$), 2.15 (s, 3H, C*H*$_3$), 2.13 – 1.74 (s, 4 × 3H, C*H*$_3$), 1.30 (s, 3H, CH_2C(C*H*$_3$)$_2$), 1.18 (s, 3H, CH_2C(C*H*$_3$)$_2$).

^{13}C NMR (101 MHz, Chloroform-*d*) δ: 211.6 (*C*=O), 141.7 (C(CH_3)$_2$*C*H), 140.9 (Ar*C*^{q}), 138.9 (2 × Ar*C*^{q}), 136.3 (Ar*C*^{q}), 135.6 (CH*C*^{q}–Ar), 132.9 (2 × Ar*C*^{q}), 127.7 (2 × Ar*C*H), 126.8 (Ar*C*H), 126.6 (2 × Ar*C*H), 60.5 (C(O)*C*H), 45.0 (CH*C*(CH_3)$_2$), 41.7 (CH*C*H$_2$), 30.4 (CH_2C(*C*H$_3$)$_2$), 29.2 (CH_2C(*C*H$_3$)$_2$), 16.8 (2 × C*C*H$_3$), 16.1 (3 × C*C*H$_3$) "1 × Ar*C*^{q} not observed".

IR (neat, cm^{-1}): 2954, 2927, 2863, 1690, 1444, 1381, 1129, 1115, 898, 775, 753, 692.

HRMS (ESI$^+$): calculated for $C_{25}H_{29}O$ (M - H$^+$): 345.2224 Found: 345.2230

rac-[1,1'-Biphenyl]-2-yl(4,4-dimethyl-2-phenylcyclopent-2-en-1-yl)methanone (**3as**)

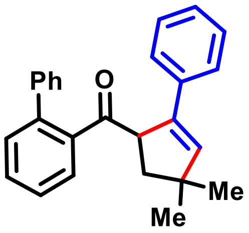

Prepared by following the **general procedure A** from 0.1 mmol (**1l**), 0.5 mmol (**2a**), and SmI_2 (25 mol%). The reaction mixture was filtered through a silica gel pad and concentrated in vacuo. The crude product was purified by column chromatography (silica gel 100-200 mesh size; hexane/ethyl acetate) to afford **3as** (28.5 mg, 0.081 mmol, 81% yield) as a viscous liquid.

^{1}H NMR (400 MHz, Chloroform-*d*) δ 7.50 – 7.34 (m, 7H, Ar*H*), 7.32 – 7.29 (m, 2H, Ar*H*), 7.14 (dd, *J* = 5.1, 1.9 Hz, 3H, Ar*H*), 7.03 (dd, *J* = 6.7, 3.0 Hz, 2H, Ar*H*), 5.89 (d, *J* = 1.6 Hz, 1H, C*H*C(CH_3)$_2$), 4.16 (ddd, *J* = 9.8, 4.7, 1.7 Hz, 1H, C(O)C*H*), 1.86 (dd, *J* = 13.1, 4.7 Hz, 1H, CHC*H*$_2$), 1.60 (dd, *J* = 13.1, 9.7 Hz, 1H, CHC*H*$_2$), 1.12 (s, 3H, CH_2C(C*H*$_3$)$_2$), 0.99 (s, 3H, CH_2C(C*H*$_3$)$_2$).

^{13}C NMR (101 MHz, Chloroform-*d*) δ: 208.9 (*C*=O), 141.1 (Ar*C*^{q}), 141.1 (Ar*C*^{q}), 140.5 (Ar*C*^{q}), 140.5 (C(CH_3)$_2$*C*H), 139.4 (Ar*C*^{q}), 136 (CH*C*^{q}–Ar), 130.5 (Ar*C*H), 130.5 (Ar*C*H), 129.3 (2 × Ar*C*H), 128.9 (Ar*C*H), 128.9 (2 × Ar*C*H), 128.2 (2 × Ar*C*H), 128 (Ar*C*H), 127.5 (Ar*C*H), 127.1 (Ar*C*H), 126.5 (2 × Ar*C*H), 57.7 (C(O)*C*H), 45.1 (CH*C*(CH_3)$_2$), 44 (CH*C*H$_2$), 29.4 (CH_2C(*C*H$_3$)$_2$), 28.8 (CH_2C(*C*H$_3$)$_2$).

IR (neat, cm^{-1}): 2954, 2927, 1686, 1448, 1202, 776, 746, 697.

HRMS (ESI$^+$): calculated for $C_{26}H_{24}ONa$ (M + Na$^+$): 375.1719 Found: 375.1719

rac-(4,4-Dimethyl-2-phenylcyclopent-2-en-1-yl)(2-fluorophenyl)methanone (**3at**)

Prepared by following the **general procedure A** from 0.1 mmol (**1m**), 0.5 mmol (**2a**), and SmI_2 (40 mol%). The reaction mixture was filtered through a silica gel pad and concentrated in vacuo. The crude product was purified by column chromatography (silica gel 100-200 mesh size; hexane/ethyl acetate) to afford **3at** (19.7 mg, 0.067 mmol, 67% yield) as a viscous liquid.

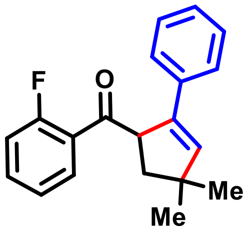

^{1}H NMR (400 MHz, Chloroform-*d*) δ: 7.81 (td, *J* = 7.6, 1.9 Hz, 1H, Ar*H*), 7.57 – 7.50 (m, 1H, Ar*H*), 7.32 – 7.28 (m, 2H, Ar*H*), 7.25 – 7.14 (m, 5H, Ar*H*), 6.20 (d, *J* = 1.3 Hz, 1H, C*H*C(CH_3)$_2$), 4.94 (ddt, *J* = 10.1, 4.8, 1.5 Hz, 1H, C(O)C*H*), 2.46 – 2.35 (m, 1H, CHC*H*$_2$), 1.95 (ddd, *J* = 13.2, 4.9, 2.1 Hz, 1H, CHC*H*$_2$), 1.19 (s, 3H, CH$_2$C(C*H*$_3$)$_2$), 1.16 (s, 3H, CH$_2$C(C*H*$_3$)$_2$).

^{13}C NMR (101 MHz, Chloroform-*d*) δ: 200.7 (d, J^3 = 4.0, *C*=O), 161.8 (d, J^1 = 254.5, ArC^q–F), 140.4 (C(CH_3)$_2$CH), 138.6 (ArC^q), 135.9 (CHC^q–Ar), 134.6 (d, J^3 = 9.1, Ar*C*H), 131.5 (d, J^3 = 3.0, Ar*C*H), 128.5 (2 × Ar*C*H), 127.3 (ArCH), 126.1 (d, J^2 = 13.1, ArC^q), 126.1 (2 × ArCH), 124.8 (d, J^4 = 3.0, ArCH), 116.9 (d, J^2 = 24.2, ArCH), 57.9 (d, J^4 = 6.1, C(O)CH) 45.6 (CH*C*(CH_3)$_2$), 44.0 (d, J^5 = 2.0, CH*C*H$_2$), 29.6 (CH$_2$C(*C*H$_3$)$_2$), 28.9 (CH$_2$C(*C*H$_3$)$_2$).

^{19}F NMR (376 MHz, Chloroform-*d*) δ: –109.9 (s).

IR (neat, cm^{-1}): 2953, 2928, 1681, 1607, 1478, 1449, 1324, 1267, 1195, 1101, 765, 748, 690.

HRMS (ESI$^+$): calculated for $C_{20}H_{20}OF$ (M + H$^+$): 295.1493 Found: 295.1487

rac-(4,4-Dimethyl-2-phenylcyclopent-2-en-1-yl)(2-iodophenyl)methanone (**3au**)

Prepared by following the **general procedure A** from 0.1 mmol (**1n**), 0.5 mmol (**2a**), and SmI_2 (25 mol%). The reaction mixture was filtered through a silica gel pad and concentrated in vacuo. The crude product was purified by column chromatography (silica gel 100-200 mesh size; hexane/ethyl acetate) to afford **3au** (14.1 mg, 0.035 mmol, 35% yield) as a viscous liquid.

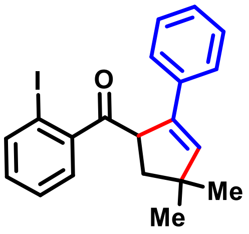

^{1}H NMR (400 MHz, Chloroform-*d*) δ: 7.93 (dd, *J* = 7.9, 1.0 Hz, 1H, Ar*H*), 7.40 – 7.29 (m, 4H, Ar*H*), 7.25 – 7.14 (m, 3H, Ar*H*), 7.13 – 7.07 (m, 1H, Ar*H*), 6.15 (d, *J* = 1.6 Hz, 1H, C*H*C(CH_3)$_2$), 4.90 (ddd, *J* = 9.9, 5.0, 1.6 Hz, 1H, C(O)C*H*), 2.25 (dd, *J* = 13.2, 9.8 Hz, 1H, CHC*H*$_2$), 2.12 (dd, *J* = 13.2, 5.0 Hz, 1H, CHC*H*$_2$), 1.21 (s, 3H, CH$_2$C(C*H*$_3$)$_2$), 1.19 (s, 3H, CH$_2$C(C*H*$_3$)$_2$).

^{13}C NMR (101 MHz, Chloroform-*d*) δ: 205.1 (*C*=O), 144.0 (ArC^q), 141.2 (C(CH_3)$_2$*C*H), 141.1 (Ar*C*H), 138.4 (ArC^q), 135.7 (CHC^q–Ar), 131.9 (Ar*C*H), 128.8 (Ar*C*H), 128.5 (2

× Ar*C*H), 127.9 (Ar*C*H), 127.4 (Ar*C*H), 126.4 (2 × Ar*C*H), 92.8 (ArC^q–I), 57.0 (C(O)*C*H), 45.7 (CH*C*(CH_3)$_2$), 43.5 (CH*C*H_2), 29.7 (CH_2C(*C*H_3)$_2$), 29.1 (CH_2C(*C*H_3)$_2$).

IR (neat, cm^{-1}): 2954, 2925, 1686, 1207, 738, 696.

HRMS (ESI$^+$): calculated for $C_{20}H_{20}IO$ (M + H$^+$): 403.0553 Found: 403.0551

rac-(3-Phenylspiro[4.5]dec-3-en-2-yl)(o-tolyl)methanone (**3av**)

Prepared by following the **general procedure A** from 0.1 mmol (**1o**), 0.5 mmol (**2a**), and SmI_2 (25 mol%). The reaction mixture was filtered through a silica gel pad and concentrated in vacuo. The crude product was purified by column chromatography (silica gel 100-200 mesh size; hexane/ethyl acetate) to afford **3av** (13 mg, 0.04 mmol, 40% yield) as a viscous liquid.

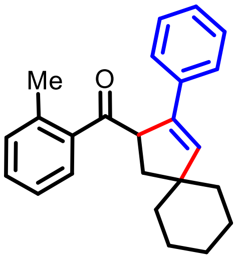

An 73% yield was obtained when 40 mol% catalyst was used.

^{1}H NMR (500 MHz, Chloroform-*d*) δ 7.76 (dd, *J* = 7.7, 1.4 Hz, 1H, Ar*H*), 7.39 (td, *J* = 7.5, 1.4 Hz, 1H, Ar*H*), 7.30 (ddt, *J* = 8.2, 3.3, 1.8 Hz, 3H, Ar*H*), 7.25 – 7.20 (m, 3H, Ar*H*), 7.19 – 7.15 (m, 1H, Ar*H*), 6.37 (s, 1H, C*H*C–Ph), 4.89 (ddd, *J* = 10.2, 5.1, 1.5 Hz, 1H, C(O)C*H*), 2.37 (s, 3H, *o*-CC*H$_3$*), 2.28 (dd, *J* = 13.1, 10.2 Hz, 1H, CHC*H$_2$*), 1.96 (dd, *J* = 13.1, 5.1 Hz, 1H, CHC*H$_2$*), 1.71 – 1.36 (m, 10H, cyclohexyl C*H$_2$*).

^{13}C NMR (126 MHz, Chloroform-*d*) δ 206.1 (*C*=O), 139.2 (ArC^q), 139.0 (C(Ph)*C*H), 138.3 (ArC^q), 135.9 (ArC^q), 132.2 (Ar*C*H), 131.4 (Ar*C*H), 128.5 (2 × Ar*C*H), 128.5 (Ar*C*H), 128.0 (Ar*C*H), 126.1 (2 × Ar*C*H), 125.8 (Ar*C*H), 55.6 (C(O)*C*H), 50.0 (CH_2C^q), 38.5 (CH*C*H_2), 37.9 (cyclohexyl *C*H_2), 29.9 (cyclohexyl *C*H_2), 26.1 (cyclohexyl *C*H_2), 23.7 (cyclohexyl *C*H_2), 23.5 (cyclohexyl *C*H_2), 21.2 (C*C*H_3).

IR (neat, cm^{-1}): 2921, 2850, 1684, 761, 731, 692.

HRMS (ESI$^+$): calculated for $C_{24}H_{26}ONa$ (M + Na$^+$): 353.1876 Found: 353.1873

rac-((7-Phenylspiro[3.4]oct-7-en-6-yl)(o-tolyl)methanone (**3aw**)

Prepared by following the **general procedure A** from 0.1 mmol (**1p**), 0.5 mmol (**2a**), and SmI_2 (25 mol%). The reaction mixture was filtered through a silica gel pad and concentrated in vacuo. The crude product was purified by column chromatography (silica gel 100-200 mesh size; hexane/ethyl acetate) to afford **3aw** (16 mg, 0.052 mmol, 52% yield) as a viscous liquid.

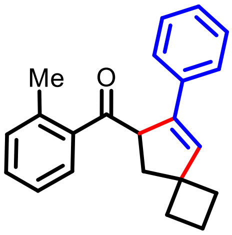

1H NMR (400 MHz, Chloroform-*d*) δ 7.73 (d, *J* = 7.6 Hz, 1H, Ar*H*), 7.43 – 7.34 (m, 1H, Ar*H*), 7.33 – 7.28 (m, 3H, Ar*H*), 7.27 – 7.20 (m, 3H, Ar*H*), 7.19 – 7.14 (m, 1H, Ar*H*), 6.54 (d, *J* = 1.4 Hz, 1H, C*H*C–Ph), 4.86 – 4.78 (m, 1H, C(O)C*H*), 2.54 (dd, *J* = 13.2, 9.6 Hz, 1H, CHC*H$_2$*), 2.36 (s, 3H, *o*-CC*H$_3$*), 2.28 (dd, *J* = 13.2, 4.3 Hz, 1H, CHC*H$_2$*), 2.21 (dtd, *J* = 9.3, 7.0, 6.3, 3.4 Hz, 2H, cyclobutyl C*H$_2$*), 2.03 (ttd, *J* = 12.5, 7.6, 6.4, 2.6 Hz, 2H, cyclobutyl C*H$_2$*), 1.89 (dtq, *J* = 9.0, 6.7, 4.8 Hz, 2H, cyclobutyl C*H$_2$*).

^{13}C NMR (101 MHz, Chloroform-*d*) δ: 205.6 (*C*=O), 139.9 (Ar*C*^{q}), 138.8 (Ar*C*^{q}), 138.3 (Ar*C*^{q}), 138.2 (C(Ph)*C*H), 135.7 (CH*C*–Ph), 132.2 (Ar*C*H), 131.4 (Ar*C*H), 128.6 (2 × Ar*C*H), 128.4 (Ar*C*H), 127.4 (Ar*C*H), 126.1 (2 × Ar*C*H), 125.7 (Ar*C*H), 56.3 (C(O)*C*H), 52.7 (CH_2*C*^{q}), 44.4 (CH*C*H_2), 35.3 (cyclobutyl *C*H_2), 35 (cyclobutyl *C*H_2), 21.2 (C*C*H_3), 16.8 (cyclobutyl *C*H_2).

IR (neat, cm^{-1}): 2925, 1681, 1446, 1209, 1194, 757, 730, 692.

HRMS (ESI$^+$): calculated for $C_{22}H_{22}ONa$ (M + Na$^+$): 325.1563 Found: 325.1563

rac- 4-(3-Methyl-5-(2-methylbenzoyl)cyclopent-1-en-1-yl)benzonitrile (**3ax**)

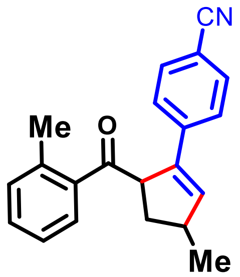

Prepared by following the **general procedure A** from 0.1 mmol (**1q**), 0.5 mmol (**2ab**), and SmI_2 (25 mol%). The reaction mixture was filtered through a silica gel pad and concentrated in vacuo. The crude product was purified by column chromatography (silica gel 100-200 mesh size; hexane/ethyl acetate) to afford **3ax** (14 mg, 0.047 mmol, 47% yield) as a yellow solid and as a 2:1 mixture of diastereomers.

^{1}H NMR (400 MHz, Chloroform-*d*) δ: 7.76 (ddd, *J* = 13.2, 7.8, 1.4 Hz, 1H, Ar*H*), 7.59 – 7.48 (m, 2H, Ar*H*), 7.42 (tdd, *J* = 7.6, 4.2, 1.4 Hz, 1H, Ar*H*), 7.35 (dq, *J* = 8.3, 1.9 Hz, 2H, Ar*H*), 7.33 – 7.24 (m, 2H, Ar*H*), 6.60 – 6.32 (m, 1H, C*H*CH(CH_3)), 4.87 (ddt, *J* = 9.7, 6.0, 1.8 Hz, 1H, C(O)C*H*), 3.18 – 2.96 (m, 1H, CH_2C*H*(CH_3)), 2.75 (ddd, *J* = 13.1, 9.9, 8.4 Hz, 1H, CHC*H*2 major diastereomer), 2.44 – 2.39 (m, 1H, CHC*H*2 minor diastereomer), 2.37 (s, 3H, CC*H*3), 2.09 – 1.93 (m, 1H, CHC*H*2, minor diastereomer,), 1.70 (ddd, *J* = 13.1, 6.2, 5.4 Hz, 1H CHC*H*2, major diastereomer), 1.17 (d, *J* = 7.0 Hz, 3H CH_2CHC*H*3).

^{13}C NMR (101 MHz, Chloroform-*d*) δ: 205.1 (*C*=O major diastereomer), 204.6 (*C*=O minor diastereomer), 140.6 (Ar*C*^{q}), 140.3 {(*C*HCH(CH_3) minor diastereomer}, 140.3 (Ar*C*^{q}), 139.9 (Ar*C*^{q}), 139.8 {(*C*HCH(CH_3) major diastereomer}, 139.7 (Ar*C*^{q}), 139.3 (Ar*C*^{q}), 139.1 (Ar*C*^{q}), 137.5 (CH*C*^{q}–Ar major diastereomer), 137.4 (CH*C*^{q}–Ar, minor diastereomer), 132.5 (Ar*C*H), 132.4 (2 × Ar*C*H minor diastereomer), 132.4 (2 × Ar*C*H major diastereomer), 131.9 (Ar*C*H major diastereomer), 131.8 (Ar*C*H minor diastereomer), 128.7 (Ar*C*H major diastereomer), 128.6 (Ar*C*H minor diastereomer), 126.6 (2 × Ar*C*H major diastereomer), 126.5 (2 × Ar*C*H minor diastereomer), 126.0 (Ar*C*H major diastereomer), 125.9 (Ar*C*H minor diastereomer), 119.2 (Ar*C*N major diastereomer), 119.2 (Ar*C*N minor diastereomer), 110.6 (Ar*C*^{q}–CN minor diastereomer), 110.5 (Ar*C*^{q}–CN major diastereomer), 56.2 (C(O)*C*H minor diastereomer), 56.1 (C(O)*C*H major diastereomer), 40.5 {(CH*C*H(CH_3) major diastereomer}, 40.2 {(CH*C*H(CH_3) minor diastereomer}, 38.5 (CH*C*H2 minor diastereomer), 38.0 (CH*C*H2 major diastereomer), 21.5 (CH_2CH*C*H3 major diastereomer), 21.4 (CH_2CH*C*H3 minor diastereomer), 21.0 (C*C*H3 major diastereomer), 20.7 (C*C*H3 minor diastereomer).

IR (neat, cm^{-1}): 2958, 2926, 2225, 1679, 1603, 1454, 1325, 1286, 1209, 1179, 829, 753, 735.

HRMS (ESI$^+$): calculated for $C_{21}H_{18}NO$ (M - H$^+$): 300.1394 Found: 300.1400

rac- 1-(4,4-Dimethyl-2-phenylcyclopent-2-en-1-yl)-3-phenylpropan-1-one (**3aab**)

Prepared by following the **general procedure A** from 0.2 mmol (**1u**), 1.0 mmol (**2a**), and SmI_2 (25 mol%). The reaction mixture was filtered through a silica gel pad and concentrated in vacuo. The crude product was purified by column chromatography (silica gel 100-200 mesh size; hexane/CH_2Cl_2) to afford **3aab** (9 mg, 0.015 mmol, 15% yield) as a viscous liquid.

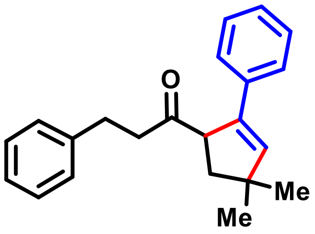

^{1}H NMR (400 MHz, Chloroform-*d*) δ: 7.29 – 7.12 (m, 8H, Ar*H*), 7.09 – 7.02 (m, 2H, Ar*H*), 6.10 (d, *J* = 1.7 Hz, 1H, C=C*H*C(CH_3)$_2$), 4.14 (ddd, *J* = 9.5, 6.5, 1.8 Hz, 1H, C(O)C*H*), 2.81 (ddt, *J* = 13.5, 9.7, 6.3 Hz, 2H, COCH_2), 2.69 (ddd, *J* = 15.6, 8.8, 5.6 Hz, 1H, COCH$_2$CH_2), 2.64 – 2.47 (m, 1H, COCH$_2$CH_2), 2.10 (dd, *J* = 13.2, 9.5 Hz, 1H, COCHCH_2), 1.69 (dd, *J* = 13.2, 6.6 Hz, 1H, COCHCH_2), 1.15 (s, 3H, CHC(CH_3)$_2$), 1.12 (s, 3H, CHC(CH_3)$_2$).

^{13}C NMR (101 MHz, Chloroform-*d*) δ: 212.0 (*C*=O), 141.4 (ArC^q), 141.1 (C=*C*HC(CH$_3$)$_2$), 138.4 (ArC^q), 135.8 (HC=C^q–Ar), 128.8 (2 × Ar*C*H), 128.6 (2 × Ar*C*H and Ar*C*H), 127.6 (Ar*C*H), 126.2 (Ar*C*H), 125.9 (2 × Ar*C*H), 59.4 (C(O)*C*H), 45.6 (CH$_2$*C*(CH$_3$)$_2$), 42.7 (CO*C*H$_2$), 41.0 (COCH*C*H$_2$), 30.1 (COCH$_2$*C*H$_2$), 29.2 (CH$_2$C(*C*H$_3$)$_2$), 29.1 (CH$_2$C(*C*H$_3$)$_2$) "1 × Ar*C*H not observed".

IR (neat, cm^{-1}): 3026, 2953, 2928, 2864, 1705, 1495, 1452, 1360, 1090, 1073, 760, 695.

HRMS (ESI$^+$): calculated for $C_{22}H_{24}ONa$ (M + Na$^+$): 327.1719 Found: 327.1716

rac- 1-(4,4-Dimethyl-2-phenylcyclopent-2-en-1-yl)-3-methylbutan-1-one (**3aac**)

Prepared by following the **general procedure A** from 0.1 mmol (**1v**), 0.5 mmol (**2a**), and SmI_2 (25 mol%). The reaction mixture was filtered through a silica gel pad and concentrated in vacuo. The crude product was purified by column chromatography (silica gel 100-200 mesh size; hexane/CH_2Cl_2) to afford **3aac** (16 mg, 0.064 mmol, 64% yield) as a viscous liquid.

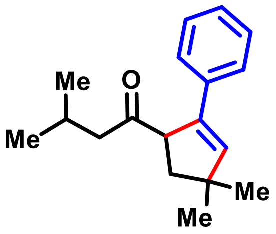

^{1}H NMR (400 MHz, Chloroform-*d*) δ: 7.36 – 7.08 (m, 5H, Ar*H*), 6.15 – 6.02 (m, 1H, C=C*H*C(CH$_3$)$_2$), 4.20 – 4.09 (m, 1H, C(O)C*H*), 2.58 – 2.32 (m, 1H, COCH_2), 2.25 – 2.02 (m, 3H, COCH_2, COCHCH_2, and COCH$_2$C*H*), 1.84 (dd, *J* = 13.1, 6.5 Hz, 1H, CO CHCH_2), 1.23 (s, 3H, CH$_2$C(CH_3)$_2$), 1.15 (s, 3H, CH$_2$C(CH_3)$_2$), 0.84 (d, *J* = 6.2 Hz, 3H CHC(CH_3)$_2$), 0.74 (d, *J* = 6.2 Hz, 3H, CHC(CH_3)$_2$).

^{13}C NMR (101 MHz, Chloroform-*d*) δ: 212.4 (*C*=O), 141.0 (C=*C*HC(CH$_3$)$_2$), 138.6 (ArC^q), 136.0 (HC=C^q–Ar), 128.7 (2 × Ar*C*H), 127.5 (Ar*C*H), 126.0 (2 × Ar*C*H), 59.5 (C(O)*C*H), 48.5 (C(O)*C*H$_2$), 45.5 (CH$_2$*C*(CH$_3$)$_2$), 42.8 (COCH*C*H$_2$), 29.3 (CH$_2$C(*C*H$_3$)$_2$), 29.2 (CH$_2$C(*C*H$_3$)$_2$), 23.9 (CH$_2$*C*H(CH$_3$)$_2$), 22.8 (CH(*C*H$_3$)$_2$), 22.6 (CH(*C*H$_3$)$_2$).

IR (neat, cm^{-1}): 2953, 2926, 2867, 1703, 1464, 1361, 1063, 760, 693.

HRMS (ESI$^+$): calculated for $C_{18}H_{24}ONa$ (M + Na$^+$): 279.1719 Found: 279.1713

rac- 1-(4,4-Dimethyl-2-phenylcyclopent-2-en-1-yl)ethan-1-one (**3aad**)

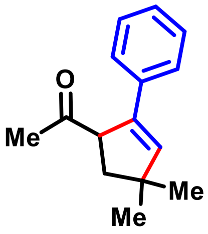

Prepared by following the **general procedure A** from 0.1 mmol (**1w**), 0.5 mmol (**2a**), and SmI_2 (25 mol%). The reaction mixture was filtered through a silica gel pad and concentrated in vacuo to afford the final product **3aad** (21.2 mg, 0.099 mmol, 99% yield) as a colourless liquid.

^{1}H NMR (400 MHz, Chloroform-*d*) δ: 7.36 – 7.27 (m, 4H, Ar*H*), 7.25 – 7.19 (m, 1H, Ar*H*), 6.13 (d, *J* = 1.7 Hz, 1H, C=C*H*C(CH_3)$_2$), 4.13 (ddd, *J* = 8.9, 6.6, 1.8 Hz, 1H, C(O)C*H*), 2.20 (dd, *J* = 13.2, 9.4 Hz, 1H CHCH_2), 2.03 (s, 3H, COCH_3), 1.87 (dd, *J* = 13.2, 6.7 Hz, 1H CHCH_2), 1.24 (s, 3H, CH_2C(CH_3)$_2$), 1.15 (s, 3H, CH_2C(CH_3)$_2$).

^{13}C NMR (101 MHz, Chloroform-*d*) δ: 211.4 (*C*=O), 141.1 (C=*C*HC(CH_3)$_2$), 138.6 (ArC^q), 135.8 (HC=C^q–Ar), 128.8 (2 × Ar*C*H), 127.6 (Ar*C*H), 125.9 (2 × Ar*C*H), 59.9 (C(O)*C*H), 45.6 (CH_2*C*(CH_3)$_2$), 43.0 (COCH*C*H_2), 29.2 (CH_2C(*C*H_3)$_2$), 29.2 (CH_2C(*C*H_3)$_2$), 26.7 (CO*C*H_3).

IR (neat, cm^{-1}): 2953, 2926, 1704, 1446, 1355, 1162, 1116, 860, 765, 748, 694.

HRMS (ESI$^+$): calculated for $C_{15}H_{19}O$ (M + H$^+$): 215.1430 Found: 215.1433

rac- (4,4-Dimethyl-2-phenylcyclopentyl)(o-tolyl)methanone (**3aaf**)

Prepared by following the **general procedure A** from 0.1 mmol (**1b**), 0.5 mmol styrene, and SmI_2 (25 mol%). The reaction mixture was filtered through a silica gel pad and concentrated in vacuo to afford the final product **3aaf** (29 mg, 0.099 mmol, 99% yield) as a sticky liquid and as a 1.6:1 mixture of diastereomers.

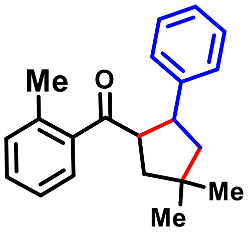

^{1}H NMR (500 MHz, Chloroform-*d*) δ 7.41 (d, *J* = 7.7 Hz, 1H, Ar*H* minor diastereomer), 7.31 – 7.24 (m, 8H, Ar*H* major and minor diastereomers), 7.22 (t, *J* = 7.4 Hz, 1H, Ar*H* major diastereomer), 7.19 – 7.10 (m, 4H, Ar*H* major and minor diastereomers), 7.09 – 7.01 (m, 2H, Ar*H* major and minor diastereomers), 6.98 (d, *J* = 7.5 Hz, 1H, Ar*H* minor diastereomer), 6.95 (d, *J* = 6.6 Hz, 1H, Ar*H* major diastereomer), 4.33 (td, *J* = 9.7, 7.6 Hz, 1H, COC*H* minor diastereomer), 3.91 – 3.78 (m, 2H, COC*H* and COCHC*H*Ph major diastereomer), 3.72 (td, *J* = 10.2, 7.5 Hz, 1H, COCHC*H*Ph minor diastereomer), 2.37 (s, 3H, CC*H$_3$* major diastereomer), 2.25 (dd, *J* = 13.0, 9.1 Hz, 1H, CH_2 minor diastereomer), 2.10 – 1.99 (m, 3H, CH_2 major diastereomer and CH_2 minor diastereomer), 1.98 – 1.93 (m, 1H, CH_2 minor diastereomer), 1.90 (s, 3H, CCH_3 minor diastereomer), 1.87 – 1.78 (m, 2H, CH_2 major diastereomer), 1.77 – 1.73 (m, 1H, CH_2 minor diastereomer), 1.30 (s, 3H , C(CH_3)$_2$ minor diastereomer), 1.19 (s, 3H, C(CH_3)$_2$ major diastereomer), 1.18 (s, 3H C(CH_3)$_2$ minor diastereomer), 1.16 (s, 3H C(CH_3)$_2$ major diastereomer).

^{13}C NMR (126 MHz, Chloroform-*d*) δ: 206.7, 206.1, 144.2, 142.8, 139.2, 139.2, 138.7, 137.8, 131.7, 131.7, 130.9, 130.8, 128.6, 128.6, 128.5, 128.1, 128.1, 127.5, 126.4, 126.2, 125.6, 125.3, 58.3, 53.3, 49.7, 48.7, 48.1, 47.8, 46.4, 44.1, 39.0, 38.6, 30.8, 29.9, 29.8, 29.0, 21.0, 20.7.

rac- 4,4-Dimethyl-2-(2-methylbenzoyl)cyclopentane-1-carbonitrile (**3aah**)

Prepared by following the **general procedure A** from 0.1 mmol (**1b**), 0.5 mmol acrylonitrile, and SmI_2 (25 mol%). The reaction mixture was filtered through a silica gel pad and concentrated in vacuo. The crude product (d.r. 2.3:1) was purified by column chromatography (silica gel 100-200 mesh size; hexane/ethyl acetate) to afford the final compound (major diastereomer: 13.5 mg, 0.056 mmol, 56% yield. minor diastereomer: 5 mg, 0.019 mmol, 19% yield) as a sticky colourless liquid.

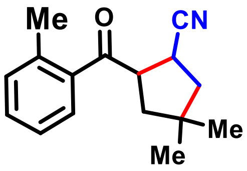

Major diastereomer: **^{1}H NMR** (400 MHz, Chloroform-*d*) δ: 7.61 (dd, *J* = 7.7, 1.3 Hz, 1H, Ar*H*), 7.41 (td, *J* = 7.5, 1.4 Hz, 1H, Ar*H*), 7.33 – 7.26 (m, 2H, Ar*H*), 4.09 (dt, *J* = 9.7, 8.3 Hz, 1H, C(O)C*H*), 3.66 (q, *J* = 8.4 Hz, 1H, C*H*–CN), 2.49 (s, 3H, *o*-CCH_3), 2.08 – 1.95 (m, 2H, (CN)CHCH_2), 1.90 (dd, *J* = 12.9, 8.3 Hz, 1H, CHCH_2), 1.52 (dd, *J* = 13.0, 8.6 Hz, 1H, CHCH_2), 1.17 (s, 3H, C(CH_3)$_2$), 1.01 (s, 3H, C(CH_3)$_2$).

^{13}C NMR (101 MHz, Chloroform-*d*) δ: 202.2 (*C*=O), 139.0 (ArC^q), 136.7 (ArC^q), 132.4 (Ar*C*H), 132.1 (Ar*C*H), 128.8 (Ar*C*H), 126.1 (Ar*C*H), 122.9 (*C*N), 54.2 (C(O)*C*H), 45.6 (*C*H_2), 45.3 (*C*H_2), 40.6 (*C*(CH_3)$_2$), 28.9 (C(*C*H_3)$_2$), 28.7 (*C*H(CN)), 28.4 (C(*C*H_3)$_2$), 21.5 (*o*-C*C*H_3).

HRMS (ESI$^+$): calculated for $C_{16}H_{19}ONNa$ (M + Na^+): 264.1359 Found: 264.1350.

Minor diastereomer: **^{1}H NMR** (400 MHz, Chloroform-*d*) δ: 7.58 – 7.53 (m, 1H, Ar*H*), 7.40 (td, *J* = 7.5, 1.4 Hz, 1H, Ar*H*), 7.31 – 7.26 (m, 2H, Ar*H*), 4.01 (dt, *J* = 9.5, 8.1 Hz, 1H, C(O)C*H*), 3.22 (q, *J* = 7.8 Hz, 1H, C*H*–CN), 2.55 (s, 3H, *o*-CCH_3), 2.10 – 2.02 (m, 2H, (CN)CHCH_2 and CHCH_2), 1.97 – 1.91 (m, 1H, (CN)CHCH_2), 1.85 (dd, *J* = 13.3, 7.9 Hz, 1H, CHCH_2), 1.22 (s, 3H, C(CH_3)$_2$), 1.07 (s, 3H, C(CH_3)$_2$).

^{13}C NMR (101 MHz, $CDCl_3$) δ: 202.4 (*C*=O), 139.5 (ArC^q), 137.3 (ArC^q), 132.5 (Ar*C*H), 131.9 (Ar*C*H), 128.4 (Ar*C*H), 125.9 (Ar*C*H), 120.9 (*C*N), 50.8 (C(O)*C*H), 45.5 (*C*H_2), 43.5 (*C*H_2), 39.1 (*C*(CH_3)$_2$), 31.7 (*C*H(CN)), 29.9 (C(*C*H_3)$_2$), 29.6 (C(*C*H_3)$_2$), 21.4 (*o*-C*C*H_3).

HRMS (ESI$^+$): calculated for $C_{18}H_{24}ONa$ (M + Na^+): 264.1359 Found: 264.1355.

4,4-Dimethyl-3,4-dihydronaphthalen-1(2H)-one (**4**)[4]

^{1}H NMR (500 MHz, Chloroform-d) δ: 8.02 (d, *J* = 7.8 Hz, 1H, Ar*H*), 7.53 (t, *J* = 7.6 Hz, 1H, Ar*H*), 7.42 (d, *J* = 7.9 Hz, 1H, Ar*H*), 7.30 (t, J = 7.5 Hz, 1H, Ar*H*), 2.73 (t, *J* = 6.7 Hz, 2H, COCH_2), 2.03 (t, *J* = 6.8 Hz, 2H, C(CH_3)$_2$CH_2), 1.40 (s, 6H, 2 × CH_3).

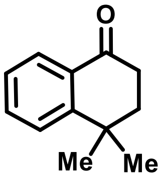

4

^{13}C NMR (126 MHz, Chloroform-*d*) δ: 198.7 (*C*=O), 152.5 (ArC^q–C(O)), 134.1 (Ar*C*H), 131.4 (ArC^q–C(CH_3)$_2$), 127.5 (Ar*C*H), 126.5 (Ar*C*H), 126.1 (Ar*C*H), 37.3 (CO*C*H_2), 35.4 (C(CH_3)$_2$*C*H_2), 34.1 (CH_2*C*(CH_3)$_2$), 30.0 (2 × *C*H_3).

5. Mechanistic Study

Control experiments with Lewis acids

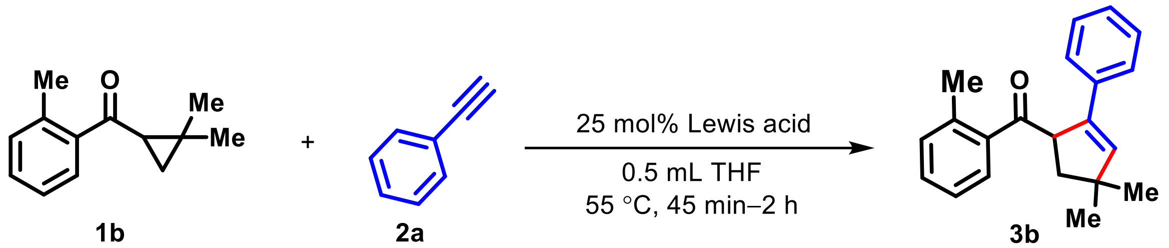

1b 0.1 mmol; **2a** 0.5 mmol; **3b**

SmI_3, $Yb(OTf)_3$, $La(OTf)_3$, $Fe(OTf)_3$, $Sm(OTf)_3$, $Al(OTf)_3$, $Gd(OTf)_3$ → **3b** not detected

To a clean, oven–dried microwave reaction vial containing a magnetic stir–bar, ketone derivative **1b** (0.1 mmol, 1 eq.) and Lewis acid (0.025 mmol, 25 mol%) were added and the vial flushed with nitrogen. After 15 min, THF (0.5 mL) and alkyne **2a** (0.5 mmol, 5 eq.) were introduced by syringe. The tube was placed in a preheated oil bath at 55 °C. The reaction was stirred (at 400 rpm) vigorously for 45 min. The reaction was cooled to room temperature and filtered through a silica gel pad (100-200 mesh size) using CH_2Cl_2 (15 mL). Excess solvent was removed in vacuo and the reaction mixture was carefully analysed by ^{1}H NMR using nitromethane as an internal standard.

Reactivity comparison between <u>(2,2-dimethylcyclopropyl)(*o*-tolyl)methanone</u> and <u>(2,2-dimethylcyclopropyl)(2,6-dimethylphenyl)methanone</u>

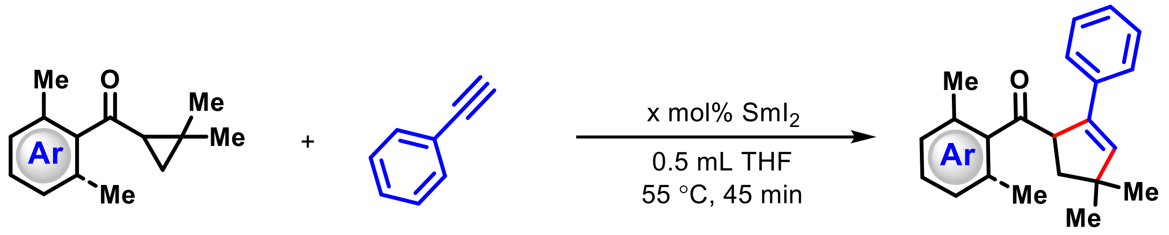

1b or **1c** 0.1 mmol; **2a** 0.5 mmol; **3b** or **3c** Yield %

SmI_2	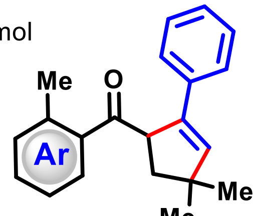 **3b**	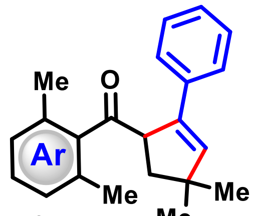 **3c**
25 mol%	99%	99%
20 mol%	79%	95%
15 mol%	50%	87%

To an oven–dried microwave reaction vial containing a magnetic stir–bar, ketone derivative **1b** or **1c** (0.1 mmol, 1 eq.) was added and the vial flushed with nitrogen. After 15 min, THF (0.5 mL) and alkyne **2a** (0.5 mmol, 5 eq.) were introduced by

syringe. The vial was placed in a preheated oil bath at 55 °C, followed by the addition of freshly prepared SmI_2 (15-25 mol%, 0.1 M). The reaction was stirred (at 400 rpm) vigorously for 45 min. The reaction was cooled to room temperature and filtered through a silica gel pad (100-200 mesh size) using CH_2Cl_2 (15 mL). Excess solvent was removed in vacuo and the reaction mixture was carefully analysed by ^1H NMR using nitromethane as an internal standard.

6. Reaction Colour *vs* Conversion Study:

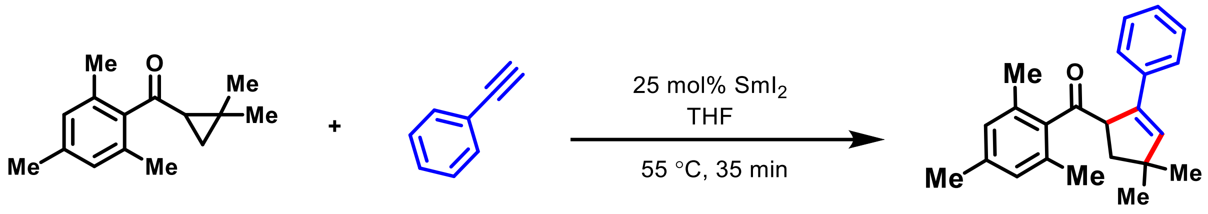

1j **2a** **3aq**

To a clean, oven–dried reaction vial containing a magnetic stir–bar, ketone **1j** (1 mmol, 1 eq.) was added and the vial flushed with N_2. After 15 min, THF (5 mL) and alkyne **2a** (5 mmol) were introduced by syringe. The vial was placed in a preheated oil bath at 55 °C, followed by the addition of freshly prepared SmI_2 (25 mol%, 0.1 M, 2.5 mL). The reaction was stirred (at 400 rpm) vigorously for 35 min. The reaction mixture was monitored by ^{1}H NMR to detect the amount of product **3aq** and starting material **1j**. The reaction was over after 7 min (with 99% product formation) and the mixture was still blue. The blue colour associated with SmI_2 was lost after 30 min.

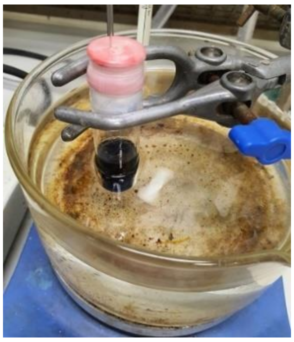
0 min

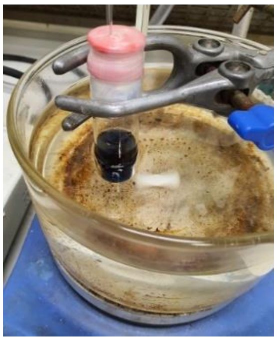
1 min
(50% NMR yield of **3aq**)

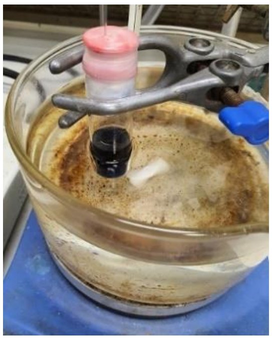
2 min
(70% NMR yield of **3aq**)

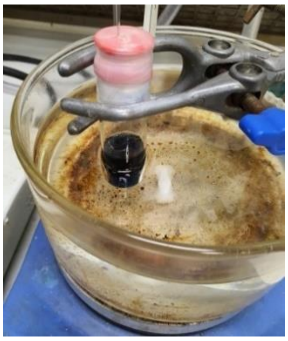
3 min

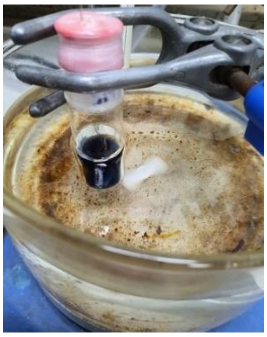
4 min
(86% yield of **3aq**)

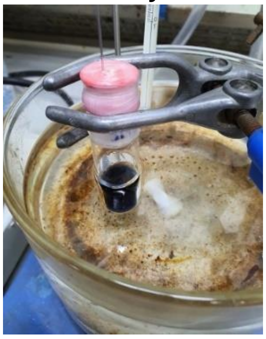
5 min

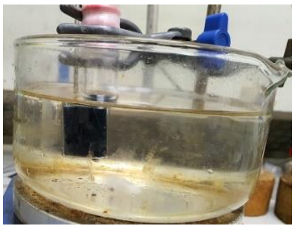
6 min
(95% yield of **3aq**)

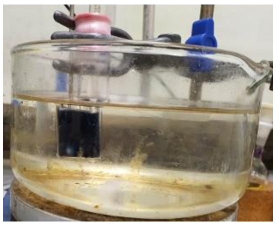
7 min
(99% yield of **3aq**)

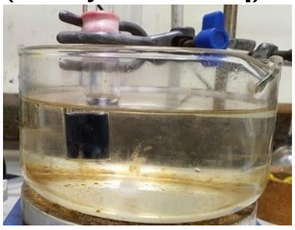
9 min

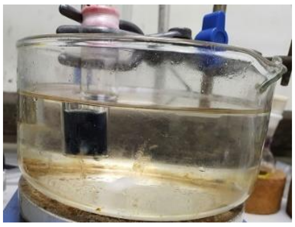
12 min

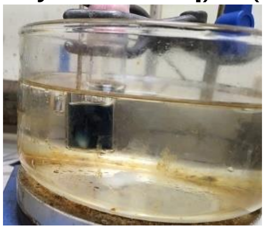
15 min

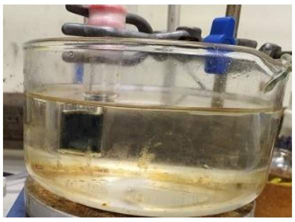
18 min

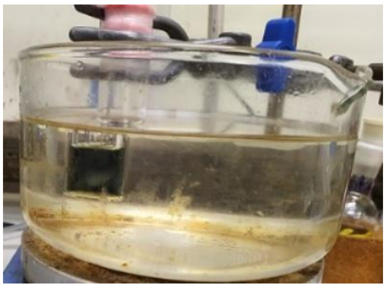
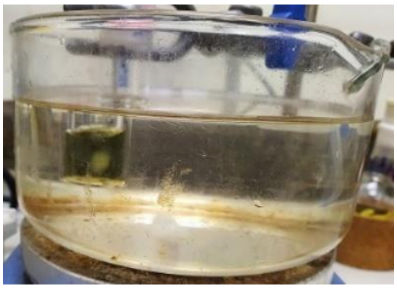
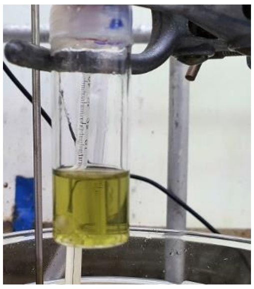

7. Large Scale Reaction

General Procedure B: To an oven–dried round-bottom flask containing a magnetic stir–bar, ketone derivative **1c** or **1k** (1 eq.) was added and the flask flushed with nitrogen. After 15 min, THF (5 mL/mmol) and alkyne **2a** (5 eq.) were introduced by syringe. The vial was placed in a preheated oil bath at 55 °C, followed by the addition of freshly prepared SmI_2 (15 or 25 mol%, 0.1 M). The reaction was stirred (at 400 rpm) vigorously for 35 min. After completion the reaction mixture was diluted with CH_2Cl_2 followed by saturated aqueous sodium thiosulphate solution. The mixture was poured into a saturated aqueous solution of sodium potassium tartrate and the aqueous layer was extracted three times with CH_2Cl_2. The combined organic layer was dried over $MgSO_4$ and concentrated in vacuo to give the crude product. Finally, the crude product was purified by column chromatography (silica gel 100-200 mesh size; hexane/ethyl acetate) to afford the desired product.

Gram-scale reaction

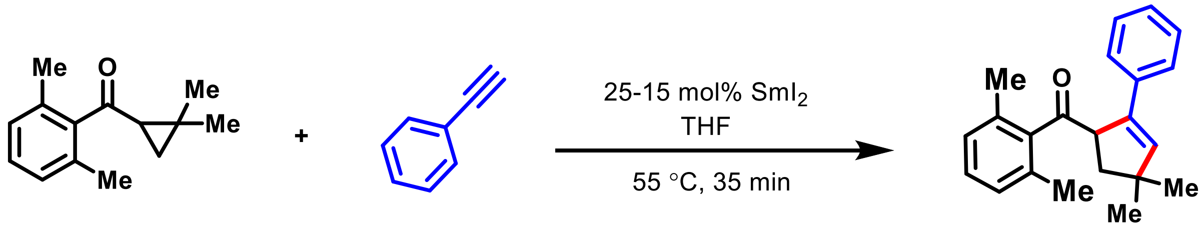

Prepared by following the **general procedure B** from **1c** (1.01 g, 5 mmol), **2a** (2.74 mL, 25 mmol), and SmI_2 (25 mol%). The crude product was purified by column chromatography (silica gel 100-200 mesh size; hexane/ethyl acetate) to afford **3c** (1.3 g, 4.27 mmol, 86% yield) as a light-yellow solid.

An 82% yield was obtained when the reaction was performed in 1 mmol scale with 15 mol% catalyst loading.

mmol-scale reaction

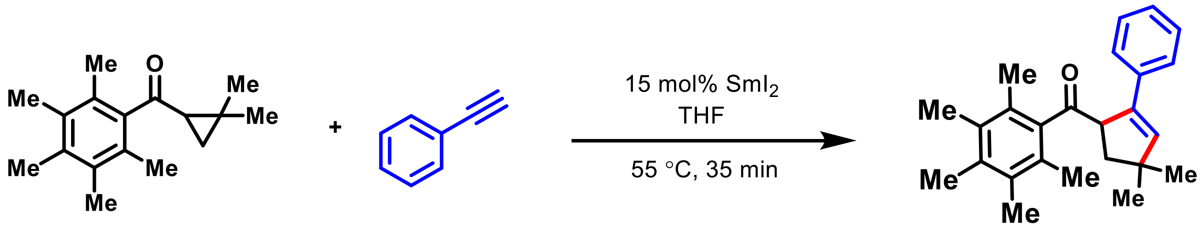

Prepared by following the **general procedure B** from **1k** (300 mg, 1.23 mmol), **2a** (677 µL, 6.15 mmol), and SmI_2 (15 mol%). The crude product was purified by column chromatography (silica gel 100-200 mesh size; hexane/ethyl acetate) to afford **3ar** (384 mg, 1.11 mmol, 90% yield) as a white solid.

8. Product Manipulation

Epoxidation:

rac-(4,4-Dimethyl-1-phenyl-6-oxabicyclo[3.1.0]hexan-2-yl)(2,6-dimethylphenyl)methanone (**5**)

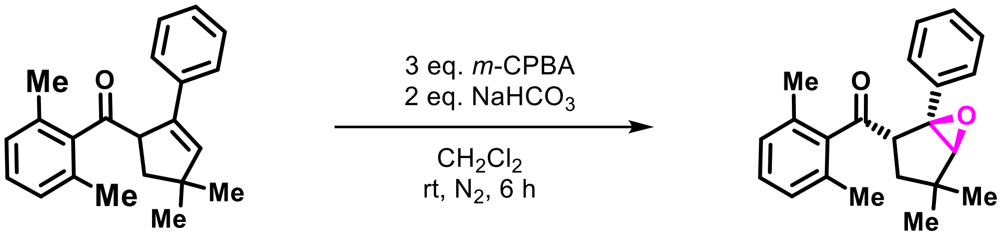

To an oven–dried reaction vial containing a magnetic stir–bar was added ketone **3c** (0.2 mmol, 61 mg) and $NaHCO_3$ (0.8 mmol, 67 mg) and the vial cooled in an ice-bath. After 10 min, CH_2Cl_2 (2 mL) was introduced by syringe. *m*-CPBA (0.4 mmol, 70 mg) was then added in portions and the mixture was vigorous stirred at room temperature for 6 h. After completion, Me_2S (30 µL, 0.4 mmol) was added and the mixture stirred for 10 min. The reaction mixture was diluted with saturated aqueous $NaHCO_3$ and extracted with CH_2Cl_2 (3 × 10 mL). The combined organic layer was dried over $MgSO_4$ and concentrated in vacuo. Finally, the crude product was purified by column chromatography (silica gel 100-200 mesh size; hexane/ethyl acetate) to afford **5** (25 mg, 0.08 mmol, 40% yield) as a white solid and as an >20:1 mixture of diastereomers.

^{1}H NMR (400 MHz, Chloroform-*d*) δ 7.56 – 7.52 (m, 2H, Ar*H*), 7.29 (dd, *J* = 5.0, 2.0 Hz, 3H, Ar*H*), 7.11 (t, *J* = 7.6 Hz, 1H, Ar*H*), 6.92 (d, *J* = 7.6 Hz, 2H, Ar*H*), 4.16 – 4.05 (m, 1H , C(O)C*H*), 3.54 (s, 1H, $C(CH_3)_2CH$), 2.02 (s, 6H, 2 × CCH_3), 1.79 – 1.76 (m, 2H, $CHCH_2$), 1.39 (s, 3H, $CH_2C(CH_3)_2$), 1.21 (s, 3H, $CH_2C(CH_3)_2$).

^{13}C NMR (101 MHz, Chloroform-*d*) δ 209.2 (*C*=O), 141.1 (ArC^q), 135.8 (ArC^q), 134.4 (2 × ArC^q), 129.4 (Ar*C*H), 129.4 (2 × Ar*C*H), 128.6 (2 × Ar*C*H), 128.2 (2 × Ar*C*H), 72.4 ($CH_{epoxide\ ring}$), 71.2 ($C_{epoxide\ ring}$), 57.3 (C(O)*C*H), 39.8 ($CH_2C(CH_3)_2$), 39.6 ($CHCH_2$), 25.5 ($CH_2C(CH_3)_2$), 24.6 ($CH_2C(CH_3)_2$), 20.2 (2 × CCH_3) "1 × Ar*C*H not observed".

IR (neat, cm^{-1}): 2956, 2925, 1686, 1461, 1448, 1421, 1332, 1200, 1004, 913, 903, 851, 767, 750, 697, 690, 655.

HRMS (ESI$^+$): calculated for $C_{22}H_{25}O_2$ (M + H$^+$): 321.1849 Found: 321.1846

Hydrogenation:

General Procedure B: In an oven-dried round bottom flask, to the ketone (1 eq.) was added Pd/C (10 mol%) and the reaction mixture was flushed with H_2 gas for 5 min. Then, MeOH and EtOAc (7:3) were introduced by syringe. The mixture was vigorous stirred under a H_2 atmosphere for 16 h. After completion, the reaction was filtered through a celite pad. Volatiles were removed in vacuo and the product was purified by column chromatography on silica gel using petroleum ether and EtOAc as eluent.

rac-(4,4-Dimethyl-2-phenylcyclopentyl)(2,6-dimethylphenyl)methanone (**6**)

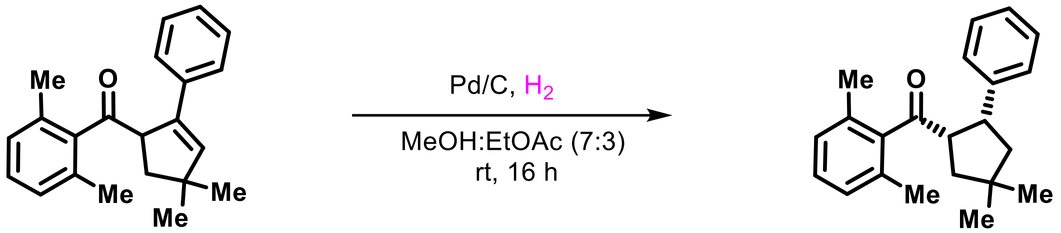

Title compound **6** was prepared from **3c** (91.3 mg, 0.3 mmol) and Pd/C (10 mol%) in 2 mL of solvent mixture according to **general procedure B**. The crude product was purified by column chromatography (silica gel 100-200 mesh size; hexane/ethyl acetate) to afford **6** (92 mg, 0.3 mmol, quan. yield) as a light-yellow solid and as an >20:1 mixture of diastereomers.

^{1}H NMR (400 MHz, Chloroform-*d*) δ 7.25 (d, *J* = 4.1 Hz, 3H, Ar*H*), 7.17 (dt, *J* = 13.1, 6.9 Hz, 2H, Ar*H*), 7.10 – 7.00 (m, 1H, Ar*H*), 6.86 (d, *J* = 7.6 Hz, 2H, Ar*H*), 3.99 (td, *J* = 8.4, 6.3 Hz, 1H, C(O)C*H*), 3.82 – 3.50 (m, 1H, CHC*H*(Ph)), 2.40 (t, *J* = 12.1 Hz, 1H, C*H*$_2$), 2.10 (dd, *J* = 13.3, 6.3 Hz, 1H, C*H*$_2$), 2.02 – 1.86 (m, 2H, C*H*$_2$), 1.84 (s, 6H, 2 × CC*H*$_3$), 1.31 (s, 3H, CH$_2$C(C*H*$_3$)$_2$)), 1.15 (s, 3H, CH$_2$C(C*H*$_3$)$_2$).

^{13}C NMR (101 MHz, Chloroform-*d*) δ 212.1 (*C*=O), 142.3 (Ar*C*^{q}), 141.6 (Ar*C*^{q}), 134.7 (2 × Ar*C*^{q}), 129.3 (2 × Ar*C*H), 128.9 (Ar*C*H), 128.4 (2 × Ar*C*H), 128.1 (2 × Ar*C*H), 126.5 (Ar*C*H), 56.3 (C(O)*C*H), 48.6 (CH*C*H(Ph), 46.9 (CH*C*H$_2$), 45.9 (CH*C*H$_2$), 38.1 (CH$_2$*C*(CH$_3$)$_2$), 30.4 (CH$_2$C(*C*H$_3$)$_2$), 30.2 (CH$_2$C(*C*H$_3$)$_2$), 20.0 (2 × C*C*H$_3$).

HRMS (ESI$^+$): calculated for $C_{22}H_{27}O$ (M + H$^+$): 307.2056 Found: 307.2047

rac-(4,4-Dimethyl-2-phenylcyclopentyl)(2,3,4,5,6-pentamethylphenyl)methanone (**3ar'**)

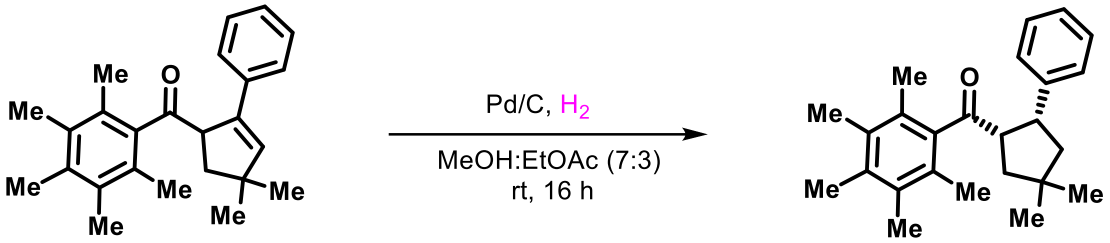

Title compound **3ar'** was prepared from **3ar** (300 mg, 0.87 mmol) and Pd/C (10 mol%) in 9 mL of solvent mixture according to **general procedure B**. The crude product was purified by column chromatography (silica gel 100-200 mesh size; hexane/ethyl acetate) to afford **3ar'** (303 mg, 0.87 mmol, quan. yield) as a white solid and as an >20:1 mixture of diastereomers.

^{1}H NMR (400 MHz, Chloroform-*d*) δ 7.34 – 7.27 (m, 2H, Ar*H*), 7.26 – 7.11 (m, 3H, Ar*H*), 3.84 (td, *J* = 8.3, 6.5 Hz, 1H, C(O)C*H*), 3.63 (dt, *J* = 11.6, 7.6 Hz, 1H, CHC*H*(Ph)), 2.37 (t, *J* = 12.0 Hz, 1H, C*H*$_2$), 2.26 – 2.20 (m, 1H, C*H*$_2$), 2.19 (s, 3H, C*H*$_3$), 2.09 (s,

6H, 2 × CH_3), 1.93 – 1.78 (m, 8H, 2 × CH_3 and CH_2), 1.29 (s, 3H, $CH_2C(CH_3)_2$), 1.12 (s, 3H, $CH_2C(CH_3)_2$).

^{13}C NMR (101 MHz, Chloroform-*d*) δ: 212.7 (*C*=O), 141.9 (Ar*Cq*), 141.1 (Ar*Cq*), 135.7 (Ar*Cq*), 133.1 (Ar*Cq*), 129.6 (2 × Ar*C*H), 128 (2 × Ar*C*H), 126.4 (Ar*C*H), 57.5 (C(O)*C*H), 48.6 (CH*C*H(Ph), 47.2 (CH*C*H$_2$), 45.3 (CH*C*H$_2$), 37.8 ($CH_2C(CH_3)_2$), 30.6 ($CH_2C(CH_3)_2$), 30.3 ($CH_2C(CH_3)_2$), 17 (2 × C*C*H$_3$), 16.2 (3 × C*C*H$_3$).

HRMS (ESI$^+$): calculated for $C_{25}H_{33}O$ (M + H$^+$): 349.2526 Found: 349.2518

Cleavage of the pentamethylphenyl ketone group:[5]

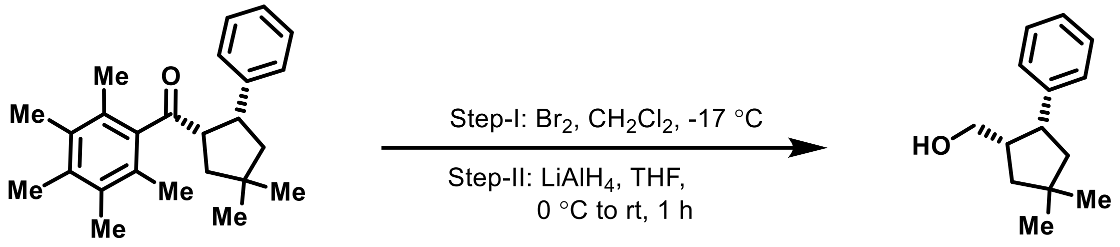

To an oven–dried reaction vial containing a magnetic stir–bar, ketone **3ar’** (40 mg, 0.12 mmol) was added and the vial flushed with nitrogen for 10 min. CH_2Cl_2 (1 mL) was then introduced by syringe and the reaction mixture was placed in a -17 °C bath. Br_2 (2 eq.) was added dropwise and the reaction mixture was kept at the same temperature for 15 min. After completion the reaction was warmed to room temperature and the volatilities were removed in vacuo. The solid residue was dissolved in THF (2 mL) and the reaction was placed in an ice-bath. $LiAlH_4$ (5 eq.) was added at 0 °C and the reaction was stirred vigorously for 1 h at room temperature. After this time, the reaction mixture was diluted with Et_2O (2 mL) and H_2O (1 mL). The organic layer was washed with saturated aqueous NaOH (1 mL) and the aqueous layer was extracted with Et_2O (3 × 3 mL). The combined organic layer was dried over $MgSO_4$ and concentrated in vacuo. Finally, the crude product was purified by column chromatography (silica gel 100-200 mesh size; hexane/ethyl acetate) to afford **7** (20 mg, 0.097 mmol, 81% yield) as a colourless liquid and as an >20:1 mixture of diastereomers.

^{1}H NMR (400 MHz, Chloroform-*d*) δ 7.34 – 7.26 (m, 3H, Ar*H*), 7.25 – 7.17 (m, 2H, Ar*H*), 3.54 (ddd, *J* = 11.3, 9.4, 7.4 Hz, 1H, CH$_2$C*H*(Ph)), 3.32 (dd, *J* = 11.2, 7.4 Hz, 1H, C*H*$_2$OH), 3.22 (dd, *J* = 11.2, 6.5 Hz, 1H, C*H*$_2$OH), 2.69 – 2.51 (m, 1H, C*H*CH$_2$OH), 1.85 (d, *J* = 11.7 Hz, 1H, C*H*$_2$), 1.81 (dd, *J* = 7.5, 1.5 Hz, 1H, C*H*$_2$), 1.71 (ddd, *J* = 13.0, 7.7, 1.5 Hz, 1H, C*H*$_2$), 1.38 (dd, *J* = 12.9, 8.1 Hz, 1H, C*H*$_2$), 1.18 (s, 3H, $CH_2C(CH_3)_2$), 1.10 (s, 3H, $CH_2C(CH_3)_2$).

^{13}C NMR (126 MHz, Chloroform-*d*) δ: 142.4 (Ar*Cq*), 128.7 (2 × Ar*C*H), 128.5 (2 × Ar*C*H), 126.6 (Ar*C*H), 65.0 (*C*H$_2$OH), 46.6 (*C*H$_2$), 45.9 (*C*HCH$_2$OH), 45.4 {CH$_2$*C*H(Ph)}, 44.6 (*C*H$_2$), 38.2 ($CH_2C(CH_3)_2$), 30.5 ($CH_2C(CH_3)_2$), 29.1 ($CH_2C(CH_3)_2$).

IR (neat, cm^{-1}): 2950, 2925, 1028, 754, 698.

HRMS (ESI$^+$): calculated for $C_{14}H_{20}ONa$ (M + Na$^+$): 227.1406 Found: 227.1398

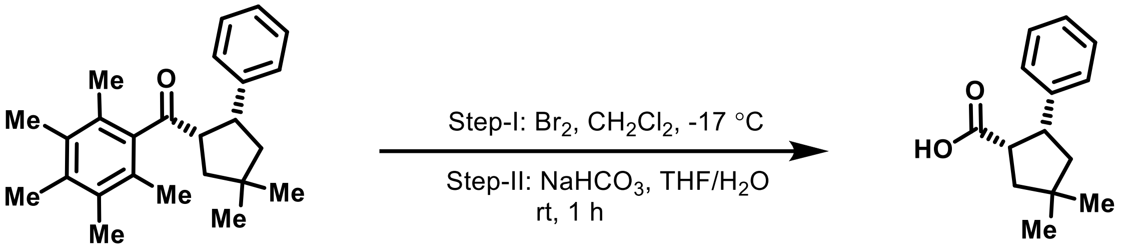

To an oven-dried reaction vial equipped with a stirrer bar was added ketone **3ar'** (50 mg, 0.14 mmol) and the vial flushed with N_2 for 10 min. The vial was cooled to -17 ºC and CH_2Cl_2 (1 mL) was added. Subsequently, Br_2 (2.0 eq.) was added dropwise and the resulting solution was stirred at -17 ºC for 15 min. After completion, the reaction mixture was warmed to room temperature and the volatiles were removed in vacuo. To a stirred solution of solid residue in THF (1 mL), aqueous $NaHCO_3$ (1 mL) was added dropwise. Following this, the resulting mixture was stirred at room temperature for 1 h and subsequently diluted with 1 M aqueous HCl and CH_2Cl_2 (2 mL). The aqueous layer was extracted with CH_2Cl_2 (3 × 4 mL). The organic layers were collected, dried over $MgSO_4$ and concentrated in vacuo. The desired compound was isolated by column chromatography (silica gel 100-200 mesh size; hexane/ethyl acetate) to afford **8** (24 mg, 0.11 mmol, 79% yield) as a white solid and as a >20:1 mixture of diastereomers.

^{1}H NMR (400 MHz, Chloroform-*d*) δ: 7.25 – 7.11 (m, 5H, Ar*H*), 3.65 (ddd, *J* = 11.8, 9.8, 6.9 Hz, 1H, CH_2C*H*(Ph)), 3.30 – 3.22 (m, 1H, C*H*CO_2H), 2.04 (t, *J* = 12.2 Hz, 1H, C*H*$_2$), 1.96 (dd, *J* = 13.3, 7.4 Hz, 1H, C*H*$_2$), 1.77 (dddd, *J* = 19.7, 13.3, 7.5, 1.5 Hz, 2H, C*H*$_2$), 1.22 (s, 3H, $CH_2C(CH_3)_2$), 1.09 (s, 3H, $CH_2C(CH_3)_2$).

^{13}C NMR (101 MHz, Chloroform-*d*) δ: 180.4 {*C*(O)OH}, 141.4 (Ar*C*^{q}), 128.2 (2 × Ar*C*H), 128.2 (2 × Ar*C*H), 126.7 (Ar*C*H), 49.5 (*C*HCO_2H), 47.6 {CH_2*C*H(Ph)}, 46.5 (*C*H$_2$), 43.7 (*C*H$_2$), 38.7 (CH_2*C*$(CH_3)_2$), 29.8 ($CH_2C(CH_3)_2$), 29.3 ($CH_2C(CH_3)_2$).

IR (neat, cm^{-1}): 2953, 2926, 2862, 1687, 1438, 1242, 964, 745, 696.

HRMS (ESI$^+$): calculated for $C_{14}H_{19}O_2$ (M + H$^+$): 219.1380 Found: 219.1374

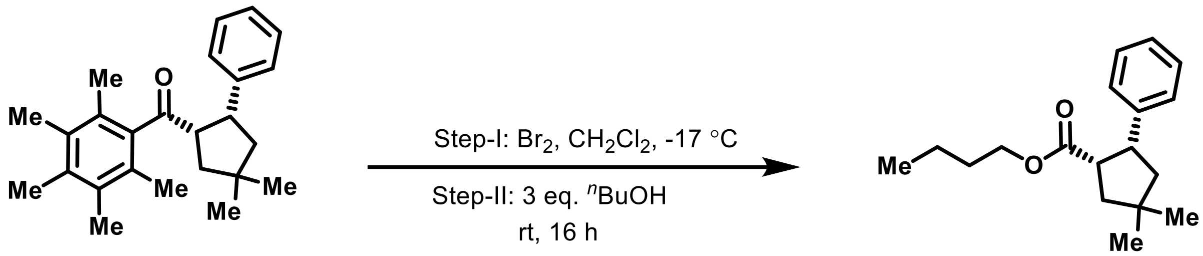

To an oven-dried reaction vial equipped with a stirrer bar was added ketone **3ar'** (50 mg, 0.14 mmol) and the vial flushed with N_2 for 10 min. The vial was cooled to -17 ºC

and CH_2Cl_2 (1 mL) was added. Following this, Br_2 (2.0 eq.) was added dropwise and the resulting solution was stirred at -17 ºC for 15 min. *n*-Butanol (3.0 eq.) was then added and the resulting solution was stirred at room temperature for 16 h. After this time, the reaction mixture was diluted with Et_2O (3 mL) and H_2O (2 mL). The aqueous layer was extracted with Et_2O (3 × 4 mL) and the combined organic layers were washed with saturated aqueous $NaHCO_3$, saturated aqueous $Na_2S_2O_3$ and brine solution. The organic layer was dried over $MgSO_4$ and concentrated in vacuo. Finally, the crude product was purified by column chromatography (silica gel 100-200 mesh size; hexane/ethyl acetate) to afford **9** (38 mg, 0.138 mmol, 98% yield) as a colourless liquid and as a >20:1 mixture of diastereomers.

^{1}H NMR (400 MHz, Chloroform-*d*) δ 7.25 – 7.13 (m, 5H, Ar*H*), 3.73 – 3.64 (m, 1H, OCH_2), 3.64 – 3.58 (m, 1H, OCH_2), 3.43 (dt, *J* = 10.8, 6.4 Hz, 1H, CH_2C*H*(Ph)), 3.31 (dt, *J* = 10.1, 7.9 Hz, 1H, C*H*$CO_2{}^n$Bu), 2.22 – 2.14 (m, 1H, CH_2), 2.10 – 1.95 (m, 3H, CH_2 and OCH_2CH_2), 1.84 – 1.70 (m, 2H, CH_2), 1.24 (s, 3H, $CH_2C(CH_3)_2$), 1.19 – 1.11 (m, 2H, CH_3CH_2), 1.09 (s, 3H, $CH_2C(CH_3)_2$), 0.78 (t, *J* = 7.1 Hz, 3H, C$H_3$$CH_2$).

^{13}C NMR (101 MHz, *Chloroform-d*) δ: 175.0 (*C*=O), 142.0 (ArC^q), 128.4 (2 × Ar*C*H), 128.1 (2 × Ar*C*H), 126.5 (Ar*C*H), 64.0 (OCH_2), 49.7 (*C*HC=O), 47.6 {CH_2*C*H(Ph)}, 46.9 (*C*H_2), 43.7 (*C*H_2), 38.8 ($CH_2C(CH_3)_2$), 30.5 (OCH_2CH_2), 29.7 ($CH_2C(CH_3)_2$), 28.9 ($CH_2C(CH_3)_2$), 19.2 (CH_3CH_2), 13.9 (CH_3CH_2).

IR (neat, cm^{-1}): 2954, 2931, 2867, 1727, 1455, 1177, 1154, 697.

HRMS (ESI$^+$): calculated for $C_{18}H_{27}O_2$ (M + H^+): 275.2006 Found: 275.2004

9. Unsuccessful Radical Acceptor Substrates

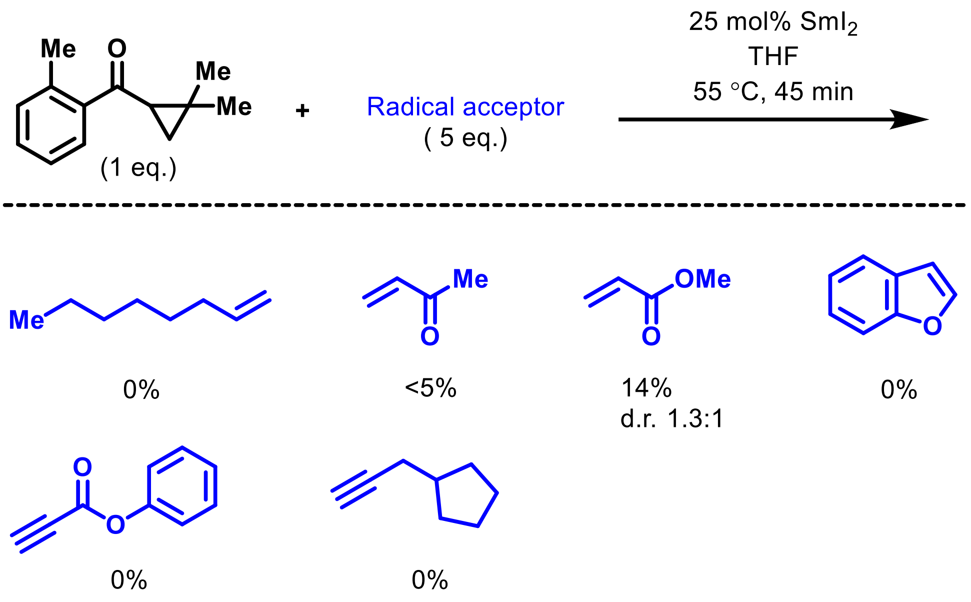

10. Computational Study:

10a. Computational Details

In this study, geometry optimisation calculations were performed using the PBE0 functional[6] and def2SVP basis set[7] using the Gaussian 09, Revision D.01.[8] Further single point energy calculations using the def2TZVP[7] basis set and corrections for solvation (PCM in THF ($\varepsilon = 7.6$)[9] and dispersion (Grimme's D3 model with Becke-Johnson damping)[10] were then run on the optimised geometries. Complexes containing Sm(II) were treated as possessing a septet spin whilst Sm(III) complexes were treated as possessing a quintet spin. The Sm^{3+} ion possesses a $4f^7$ electronic configuration giving a sextet spin ground state. Ligation of the radical anion to Sm^{3+} can possess either septet spin, arising from the spins coupling in parallel, or quintet spin, where the spins couple in an antiparallel combination. Our calculations suggest that despite the septet state (Sm(II)) being lower in energy, the reactivity takes place *via* the quintet spin state (Sm(III)). Care must be taken with computations on this f element system, as there are several close energy solutions to the Kohn-Sham equations. In all cases, stability analysis was carried out to confirm a minimum in the variational parameters had been obtained.

10b. Benchmarking – Cyclopropane Ring Opening

We have carried out computations on the simple ring-opening reaction of cyclopropylcarbinyl radical, which has been well studied both experimentally and theoretically. The experimentally determined activation energy is found to be $E_a = 7.05$ kcal mol^{-1} (M. Newcomb, A. G. Glenn, *J. Am. Chem. Soc.* 1989, **111**, 275). In the studies of Radom, the activation energy is adjusted to remove zero point energies and all thermal corrections yielding $E_a = 7.46$ kcal mol^{-1}, to which a large number of computational techniques are compared (see Table 2 – D.M. Smith, A. Nicolaides, B.T. Golding and L. Radom, *J. Am. Chem. Soc.* 1998, **120**, 10223). The PBE0/Def2-TZVP//Def2-SVP method used here produces $E_a = 10.38$ kcal mol^{-1}, somewhat overestimating the activation energy but performing in line with the findings of Newcomb and Glen that suggest errors relative to experiment of 2 - 5 kcal mol^{-1}. It should be borne in mind that this reaction is, experimentally, conducted in the gas phase. If, nevertheless, the solvation correction, PCM(THF), is included in the computations, E_a changes by –0.3 kcal mol^{-1} and inclusion of the D3(B-J) dispersion correction changes E_a by 0.04 kcal mol^{-1}. This illustrates that the reasonable agreement obtained is not due to a skewing of the computational description by these additional terms.

10c. Breakdown of Energy Contributions

Table S.1: *Breakdown of energy contributions detailing the evolution of relative energies as the successive corrections to the initial SCF energy are included. Energies in kcal mol^{-1}:*

ΔE: SCF energy computed with the PBE0 functional
ΔH: Enthalpy at 0K
ΔG: Free energy at 298.15 K and 1 atm
ΔG_{Solv}: Free energy corrected for THF solvent
ΔG_{Disp}: Free energy corrected for dispersion effects
$\Delta G_{FinalSVP}$: Free energy corrected for THF solvent and dispersion effects with def2SVP basis set
$\Delta G_{FinalTZVP}$: Free energy corrected for THF solvent and dispersion effects with def2TZVP basis set. This is the data used in the main article

Label	ΔE	ΔH	ΔG	ΔG_{Solv}	ΔG_{Disp}	$\Delta G_{FinalSVP}$	$\Delta G_{FinalTZVP}$
**1a**	0.0	0.0	0.0	0.0	0.0	0.0	0.0
**Ia**	+20.2	+19.5	+21.5	+25.0	+17.3	+20.9	+20.0
**TS(I-II)a**	+36.7	+35.0	+37.8	+41.3	+34.2	+37.7	+35.7
**IIa**	+28.1	+26.5	+28.8	+32.6	+24.7	+28.5	+26.5
**TS(II-III)a**	+30.0	+29.3	+43.8	+49.7	+26.9	+38.2	+37.5
**IIIa**	-8.7	-7.1	+8.5	+15.0	-8.8	+3.1	+4.5
**TS(III-IV)a**	-4.2	-2.5	+14.3	+19.9	-3.8	+7.2	+8.9
**IVa**	-43.0	-38.9	-20.4	-15.2	-40.9	-30.3	-26.0
**3a**	-60.3	-56.1	-41.0	-39.2	-57.4	-50.2	-45.2
**1b**	0.0	0.0	0.0	0.0	0.0	0.0	0.0
**Ib**	+21.5	+21.7	+25.4	+29.3	+21.3	+25.2	+25.1
**TS(I-II)b**	+38.3	+37.1	+40.8	+44.5	+36.5	+40.2	+38.9
**IIb**	+26.3	+25.0	+27.6	+31.9	+24.4	+28.6	+27.0
**TS(II-III)b**	+27.9	+27.6	+43.1	+49.2	+26.5	+38.1	+37.9
**IIIb**	-8.0	-6.6	+7.7	+13.4	-8.2	+3.0	+5.0
**TS(III-IV)b**	-5.5	-4.1	+12.6	+17.7	-6.1	+5.1	+6.9
**IVb**	-39.7	-35.5	-16.6	-11.0	-37.1	-26.0	-21.6
**3b**	-61.3	-57.0	-41.2	-39.7	-57.4	-50.5	-45.3
**1c**	0.0	0.0	0.0	0.0	0.0	0.0	0.0
**Ic**	+24.5	+24.0	+26.3	+30.9	+22.4	+27.0	+26.6
**TS(I-II)c**	+39.7	+38.2	+41.4	+45.5	+37.7	+41.9	+40.6
**IIc**	+26.4	+24.9	+27.4	+31.8	+24.5	+28.9	+27.1
**TS(II-III)c**	+28.7	+28.0	+43.6	+50.0	+26.3	+38.1	+38.1
**IIIc**	-9.8	-8.3	+7.2	+13.9	-9.4	+2.8	+4.6
**TS(III-IV)c**	-4.6	-3.2	+13.3	+19.1	-4.9	+6.3	+8.5
**IVc**	-37.4	-33.9	-16.4	-10.7	-36.3	-25.2	-20.8
**3c**	-60.5	-57.0	-44.0	-42.2	-59.5	-52.2	-47.7

10d. Cartesian Coordinates (Å) and computed energies (in Hartrees) for all optimised structures. All structures can be visualized via the All_geometries.xyz filed provided.

1a

SCF Energy = -2721.644493
Free Enthalpy (0K) = -2720.937477
Free Energy (298K) = -2721.029282
Free Energy (TZVP,PCM(THF),GD3BJ) = -2722.755047

C	5.409846000	-3.128732000	-1.206325000
C	6.079793000	-2.060167000	-0.608423000
C	5.360650000	-0.988825000	-0.087935000
C	3.957060000	-0.968710000	-0.163649000
C	3.295524000	-2.044521000	-0.778374000
C	4.016940000	-3.116672000	-1.290889000
C	3.145309000	0.161172000	0.358761000
C	3.815839000	1.203746000	1.167062000
C	3.030135000	2.320199000	1.806758000
C	3.910876000	2.656597000	0.656267000
C	5.223040000	3.355246000	0.922589000
O	1.941254000	0.228445000	0.101897000
Sm	-0.583121000	0.014036000	0.013650000
O	0.209147000	-1.171377000	2.211889000
C	0.815654000	-0.525256000	3.335660000
C	1.370643000	-1.649239000	4.198617000
C	0.408288000	-2.791742000	3.890683000
C	0.156527000	-2.586418000	2.406410000
I	-1.101928000	2.728590000	1.676574000
I	-0.503049000	-2.764477000	-1.663656000
O	-2.750907000	0.630514000	-1.379614000
C	-3.838786000	1.424022000	-0.891051000
C	-4.752519000	1.642393000	-2.087548000
C	-4.512174000	0.376215000	-2.903322000
C	-3.021338000	0.165489000	-2.704476000
O	0.122961000	1.349569000	-2.123433000
C	-0.305035000	2.683872000	-2.408807000
C	0.541212000	3.133718000	-3.589357000
C	0.778644000	1.816953000	-4.324384000
C	0.961079000	0.846329000	-3.169110000
O	-2.687585000	-1.050108000	1.225264000
C	-3.068608000	-0.724184000	2.564498000
C	-4.493715000	-1.230532000	2.708542000
C	-4.472919000	-2.460263000	1.805477000
C	-3.609663000	-1.980596000	0.649486000
H	-4.349736000	0.865303000	-0.086900000
H	-3.436123000	2.348026000	-0.449278000
H	-2.436259000	0.765273000	-3.426342000
H	-2.688145000	-0.880491000	-2.782110000
H	-4.436258000	2.530891000	-2.658577000
H	-5.801873000	1.790968000	-1.794716000
H	-4.789531000	0.470816000	-3.963100000
H	-5.079823000	-0.469787000	-2.481765000
H	-1.379221000	2.665529000	-2.665024000
H	-0.186465000	3.292295000	-1.498954000
H	2.006556000	0.831455000	-2.813820000
H	0.659636000	-0.189371000	-3.388953000
H	1.497539000	3.555481000	-3.240059000
H	0.039776000	3.898098000	-4.200228000
H	1.645733000	1.837257000	-5.000070000
H	-0.106657000	1.543560000	-4.922036000
H	-2.389772000	-1.240010000	3.267596000
H	-2.949376000	0.360923000	2.708144000
H	-4.214601000	-1.457629000	-0.112656000
H	-3.037947000	-2.771329000	0.140362000
H	-5.208072000	-0.482864000	2.326006000
H	-4.762854000	-1.448728000	3.752075000
H	-5.469901000	-2.790253000	1.479794000
H	-3.991383000	-3.306884000	2.322036000
H	1.585450000	0.171602000	2.973763000
H	0.046866000	0.068282000	3.859500000
H	-0.821280000	-2.948204000	2.056105000
H	0.933097000	-3.070832000	1.788631000
H	1.412961000	-1.380917000	5.263968000
H	2.390677000	-1.915300000	3.876705000
H	-0.526399000	-2.671431000	4.463102000
H	0.816388000	-3.787235000	4.116775000
H	5.902055000	-0.155609000	0.363421000
H	2.204771000	-2.040799000	-0.859909000
H	7.170841000	-2.061119000	-0.549413000
H	3.480124000	-3.943647000	-1.761982000
H	5.977089000	-3.971046000	-1.610706000
H	4.708923000	0.866229000	1.696527000
C	3.268717000	3.018360000	-0.660998000
H	3.342599000	2.624667000	2.809584000
H	1.947493000	2.327144000	1.638205000
H	3.037933000	4.095699000	-0.675357000
H	3.946520000	2.807228000	-1.504619000
H	2.329270000	2.472547000	-0.818974000
H	5.965368000	3.122570000	0.141622000
H	5.078296000	4.447921000	0.929495000
H	5.647990000	3.068405000	1.896198000

Ia

SCF Energy = -2721.612286
Free Enthalpy (0K) = -2720.906353
Free Energy (298K) = -2720.995059
Free Energy (TZVP,PCM(THF),GD3BJ) = -2722.723117

C	5.331384000	-2.914850000	-1.339423000
C	5.858628000	-1.944969000	-0.477738000
C	5.061448000	-0.923772000	0.018065000
C	3.682039000	-0.834197000	-0.325390000
C	3.169350000	-1.823474000	-1.210159000
C	3.980619000	-2.835658000	-1.700921000
C	2.843617000	0.197869000	0.198516000
C	3.398958000	1.221394000	1.128382000
C	2.772537000	2.577886000	1.241721000
C	4.141578000	2.461619000	0.638051000
C	5.319920000	2.931460000	1.457642000
O	1.556354000	0.231588000	-0.088026000
Sm	-0.583024000	-0.002326000	-0.003701000
O	0.275998000	-1.722707000	1.597736000
C	1.118023000	-1.376248000	2.712027000
C	2.123796000	-2.507276000	2.809522000
C	1.293575000	-3.703272000	2.355108000
C	0.466240000	-3.101897000	1.231751000
I	-0.965620000	1.927781000	2.375524000
I	-0.772320000	-1.983835000	-2.362666000
O	-2.897939000	0.900449000	-0.801963000
C	-3.919221000	1.472434000	0.030021000
C	-4.856412000	2.188815000	-0.924580000

C	-4.779996000	1.297430000	-2.158665000
C	-3.309274000	0.918319000	-2.180465000
O	-0.320160000	1.847159000	-1.678270000
C	-0.630437000	3.227633000	-1.442905000
C	0.466783000	4.001277000	-2.149808000
C	0.749693000	3.110023000	-3.354939000
C	0.620705000	1.715033000	-2.764821000
O	-2.510829000	-1.408218000	1.002334000
C	-2.684470000	-1.612413000	2.413942000
C	-4.093656000	-2.153009000	2.563298000
C	-4.236351000	-2.983397000	1.292947000
C	-3.523212000	-2.116833000	0.268217000
H	-4.438385000	0.659711000	0.568620000
H	-3.437418000	2.119065000	0.776521000
H	-2.702323000	1.666599000	-2.716741000
H	-3.097986000	-0.066873000	-2.620580000
H	-4.476519000	3.198296000	-1.152178000
H	-5.871186000	2.296506000	-0.515518000
H	-5.097488000	1.793827000	-3.086812000
H	-5.409668000	0.402305000	-2.026013000
H	-1.623306000	3.451124000	-1.874766000
H	-0.676848000	3.393796000	-0.356172000
H	1.566556000	1.341833000	-2.342980000
H	0.235179000	0.960990000	-3.466238000
H	1.357221000	4.074848000	-1.505621000
H	0.156183000	5.021371000	-2.417326000
H	1.739586000	3.279268000	-3.801934000
H	-0.005928000	3.273599000	-4.140535000
H	-1.934339000	-2.341703000	2.763960000
H	-2.503917000	-0.655064000	2.924358000
H	-4.207710000	-1.379996000	-0.185150000
H	-3.039232000	-2.676415000	-0.544900000
H	-4.825203000	-1.328286000	2.573229000
H	-4.225961000	-2.731095000	3.489039000
H	-5.278545000	-3.191815000	1.011728000
H	-3.720622000	-3.950727000	1.407408000
H	1.561063000	-0.390386000	2.518210000
H	0.486893000	-1.300376000	3.614843000
H	-0.523134000	-3.565702000	1.100172000
H	0.997978000	-3.140331000	0.267875000
H	2.533926000	-2.616542000	3.823657000
H	2.959395000	-2.333800000	2.113110000
H	0.645271000	-4.058518000	3.173318000
H	1.900513000	-4.551971000	2.009725000
H	5.499800000	-0.174944000	0.681067000
H	2.123024000	-1.767721000	-1.517152000
H	6.913697000	-1.987394000	-0.192396000
H	3.554055000	-3.575430000	-2.384475000
H	5.965779000	-3.714419000	-1.728977000
H	3.815787000	0.809531000	2.059309000
C	4.299560000	2.668485000	-0.848501000
H	2.664150000	3.021035000	2.235063000
H	1.927138000	2.771435000	0.577073000
H	4.415069000	3.740012000	-1.083898000
H	5.188231000	2.140274000	-1.230713000
H	3.430706000	2.287005000	-1.403728000
H	6.247384000	2.416722000	1.154921000
H	5.484751000	4.014404000	1.328482000
H	5.164770000	2.744362000	2.531303000

TS(I-II)a

SCF Energy = -2721.586019

Free Enthalpy (0K) = -2720.88176

Free Energy (298K) = -2720.969082

Free Energy (TZVP,PCM(THF),GD3BJ) = -2722.698132

C	3.719409000	-1.446914000	-0.800234000
O	2.774178000	-0.435535000	-1.177936000
C	3.257074000	0.303435000	-2.310666000
C	4.440776000	-0.491035000	-2.835406000
C	4.992772000	-1.109271000	-1.556258000
Sm	0.469788000	-0.027911000	-0.038965000
O	-0.340815000	0.669738000	2.232374000
C	-0.110762000	1.924762000	2.892329000
C	-1.233953000	2.061013000	3.909877000
C	-1.572762000	0.605911000	4.218662000
C	-1.435399000	-0.035287000	2.851923000
O	-1.647557000	0.031821000	-0.329838000
C	-2.958921000	0.012637000	-0.578296000
C	-3.574036000	1.006011000	-1.336019000
C	-2.951684000	2.290939000	-1.759681000
C	-3.770988000	2.909664000	-0.687233000
C	-3.175625000	3.137797000	0.658427000
C	-3.742600000	-1.111517000	-0.047640000
C	-5.146162000	-1.048163000	0.095068000
C	-5.869754000	-2.133522000	0.575496000
C	-5.220827000	-3.315022000	0.943031000
C	-3.832090000	-3.387371000	0.826960000
C	-3.104632000	-2.304366000	0.343334000
H	-5.671960000	-0.122637000	-0.149009000
C	-5.090480000	3.520388000	-1.016300000
O	0.237530000	-0.745791000	-2.446981000
C	-0.692555000	-0.075111000	-3.323901000
C	-0.676033000	-0.880159000	-4.612126000
C	-0.377049000	-2.285068000	-4.100017000
C	0.638863000	-2.002754000	-3.009477000
I	0.961063000	2.914119000	-0.858230000
I	0.752585000	-2.964137000	0.892064000
O	2.486876000	0.341127000	1.629658000
C	3.547410000	1.284992000	1.451651000
C	4.024471000	1.596362000	2.855594000
C	3.905649000	0.229636000	3.522571000
C	2.650094000	-0.352224000	2.879690000
H	4.355582000	0.825099000	0.853142000
H	3.145882000	2.143751000	0.895661000
H	1.745655000	-0.176292000	3.482759000
H	2.712461000	-1.433036000	2.683017000
H	3.353664000	2.329628000	3.332328000
H	5.043030000	2.009797000	2.876081000
H	3.828500000	0.277406000	4.618217000
H	4.785749000	-0.387445000	3.280445000
H	0.878117000	1.883302000	3.380119000
H	-0.088461000	2.722907000	2.134977000
H	-2.339214000	0.100688000	2.237328000
H	-1.185227000	-1.105930000	2.868108000
H	-2.101880000	2.565851000	3.458257000
H	-0.927661000	2.641268000	4.792065000
H	-2.577370000	0.469473000	4.643225000
H	-0.844387000	0.175823000	4.926047000
H	2.430551000	0.415409000	-3.026014000
H	3.549076000	1.312870000	-1.976878000
H	3.822971000	-1.435469000	0.293410000
H	3.316200000	-2.433550000	-1.081734000
H	5.164035000	0.141535000	-3.369520000
H	4.108139000	-1.280436000	-3.529118000

H	5.591717000	-0.368204000	-1.001743000
H	5.624700000	-1.992509000	-1.726656000
H	-1.683517000	-0.080704000	-2.846151000
H	-0.362668000	0.968558000	-3.437680000
H	1.656894000	-1.900416000	-3.426987000
H	0.660980000	-2.754475000	-2.207076000
H	0.127729000	-0.535919000	-5.283678000
H	-1.626814000	-0.803745000	-5.158263000
H	0.011442000	-2.965857000	-4.870957000
H	-1.282487000	-2.738974000	-3.666644000
H	-2.019220000	-2.379471000	0.260940000
H	-6.955419000	-2.051942000	0.678619000
H	-3.301673000	-4.299000000	1.115702000
H	-5.792069000	-4.164820000	1.324864000
H	-4.593841000	0.819559000	-1.676239000
H	-3.192964000	2.615949000	-2.782744000
H	-1.869390000	2.333825000	-1.584737000
H	-3.938129000	3.099947000	1.454895000
H	-2.404942000	2.384307000	0.876408000
H	-2.683783000	4.129574000	0.721662000
H	-5.810382000	3.433524000	-0.185118000
H	-4.987634000	4.603624000	-1.235300000
H	-5.545111000	3.063084000	-1.909768000

IIa

SCF Energy = -2721.599734
Free Enthalpy (0K) = -2720.895332
Free Energy (298K) = -2720.98333
Free Energy (TZVP,PCM(THF),GD3BJ) = -2722.712828

C	4.571774000	-3.949381000	-0.994890000
C	5.190682000	-3.192518000	0.001351000
C	4.679828000	-1.946839000	0.358140000
C	3.540755000	-1.425074000	-0.276231000
C	2.922990000	-2.199992000	-1.268718000
C	3.435702000	-3.445288000	-1.627511000
C	2.958679000	-0.101591000	0.091623000
C	3.733614000	0.889597000	0.603969000
C	3.246144000	2.237345000	1.030262000
C	4.063927000	3.394027000	0.532655000
C	4.026967000	4.678903000	1.286729000
O	1.658237000	0.033876000	-0.121614000
Sm	-0.474940000	-0.012911000	-0.019891000
O	0.158934000	-2.132034000	1.154686000
C	1.075025000	-2.136530000	2.266476000
C	1.684606000	-3.526480000	2.270724000
C	0.542479000	-4.375925000	1.724163000
C	-0.038893000	-3.464504000	0.657373000
I	-0.579991000	1.444564000	2.707000000
I	-0.859648000	-1.473370000	-2.724714000
O	-2.694418000	1.239935000	-0.574661000
C	-3.629230000	1.741199000	0.391460000
C	-4.513139000	2.707664000	-0.375100000
C	-4.568891000	2.049605000	-1.749258000
C	-3.151347000	1.524526000	-1.908435000
O	-0.043139000	2.140959000	-1.231545000
C	-0.384790000	3.464771000	-0.794279000
C	0.635217000	4.380343000	-1.449457000
C	0.946029000	3.635883000	-2.743867000
C	0.948461000	2.192248000	-2.277094000
O	-2.573688000	-1.344474000	0.768177000
C	-2.803455000	-1.733818000	2.130817000
C	-4.262741000	-2.145340000	2.181872000
C	-4.452981000	-2.761743000	0.800309000
C	-3.627136000	-1.833850000	-0.076261000
H	-4.215528000	0.896643000	0.795511000
H	-3.063725000	2.188047000	1.221540000
H	-2.482030000	2.280074000	-2.352754000
H	-3.070082000	0.608822000	-2.511925000
H	-4.036274000	3.699507000	-0.438195000
H	-5.498531000	2.839195000	0.094367000
H	-4.853306000	2.735849000	-2.559695000
H	-5.292866000	1.218225000	-1.747470000
H	-1.409411000	3.695564000	-1.134845000
H	-0.370057000	3.488503000	0.305850000
H	1.918370000	1.894387000	-1.848422000
H	0.666003000	1.462160000	-3.049420000
H	1.539058000	4.454859000	-0.824648000
H	0.245394000	5.396539000	-1.604856000
H	1.903855000	3.926914000	-3.198259000
H	0.152170000	3.804647000	-3.490083000
H	-2.139917000	-2.580197000	2.379244000
H	-2.538684000	-0.886450000	2.779888000
H	-4.221573000	-0.974032000	-0.429443000
H	-3.176556000	-2.316436000	-0.955729000
H	-4.910348000	-1.261781000	2.306057000
H	-4.476292000	-2.836780000	3.009484000
H	-5.502633000	-2.817853000	0.478215000
H	-4.041466000	-3.784045000	0.777488000
H	1.804388000	-1.328162000	2.121290000
H	0.502381000	-1.925581000	3.185804000
H	-1.113914000	-3.611675000	0.476255000
H	0.490549000	-3.568856000	-0.303237000
H	2.018827000	-3.830995000	3.272803000
H	2.551469000	-3.565448000	1.592402000
H	-0.202021000	-4.579910000	2.511947000
H	0.871022000	-5.340073000	1.310891000
H	5.157208000	-1.379283000	1.160161000
H	2.028532000	-1.814659000	-1.763440000
H	6.074633000	-3.580648000	0.514400000
H	2.940684000	-4.023492000	-2.412476000
H	4.972266000	-4.927689000	-1.272625000
H	4.803513000	0.694225000	0.714201000
C	4.579490000	3.414786000	-0.865628000
H	3.213654000	2.287763000	2.136344000
H	2.182070000	2.336392000	0.729401000
H	4.929859000	5.286191000	1.105228000
H	3.164241000	5.315500000	0.991500000
H	3.932739000	4.516080000	2.372285000
H	5.563815000	3.911147000	-0.929807000
H	4.675964000	2.400293000	-1.281449000
H	3.913708000	3.985518000	-1.549190000

TS(II-III)a

SCF Energy = -3029.396641
Free Enthalpy (0K) = -3028.580318
Free Energy (298K) = -3028.679292
Free Energy (TZVP,PCM(THF),GD3BJ) = -3030.755756

C	-6.526195000	0.405632000	-0.932261000
C	-5.678917000	0.283664000	0.189472000
C	-5.241294000	-1.004041000	0.567591000
C	-5.647644000	-2.123578000	-0.150423000
C	-6.489441000	-1.991158000	-1.257200000
C	-6.923470000	-0.721035000	-1.643445000
C	-5.306821000	1.422892000	0.949514000

C	-4.958234000	2.402521000	1.602516000
C	-2.929802000	3.582275000	1.017701000
C	-2.264574000	3.510505000	2.353068000
C	-2.320154000	2.774240000	-0.097002000
C	-1.048490000	3.414032000	-0.576732000
C	0.171412000	2.820287000	-0.663763000
C	1.373557000	3.620475000	-1.041469000
C	1.522327000	4.959633000	-0.646902000
C	2.656354000	5.688148000	-0.999274000
C	3.670341000	5.091994000	-1.749969000
C	3.538624000	3.759141000	-2.139731000
C	2.405673000	3.030078000	-1.784502000
O	0.380345000	1.531471000	-0.446362000
Sm	0.815363000	-0.513964000	0.006424000
O	0.214078000	-3.003047000	-0.453644000
C	-0.280203000	-3.927073000	0.525764000
C	-0.901825000	-5.054093000	-0.275805000
C	0.024993000	-5.114014000	-1.484986000
C	0.313534000	-3.642729000	-1.738821000
O	2.280106000	-2.123336000	1.431348000
C	3.222271000	-3.074314000	0.913478000
C	4.197296000	-3.323458000	2.048223000
C	3.284523000	-3.216260000	3.263536000
C	2.364267000	-2.072792000	2.867813000
O	-0.938915000	-0.706926000	-1.763482000
C	-2.284371000	-1.156511000	-1.525285000
C	-3.144694000	-0.388910000	-2.512234000
C	-2.185003000	-0.184133000	-3.678629000
C	-0.881609000	0.100687000	-2.956436000
O	2.343490000	0.780866000	1.548978000
C	1.810559000	1.799255000	2.418924000
C	3.014732000	2.605037000	2.876530000
C	3.960561000	2.475216000	1.687163000
C	3.726213000	1.038409000	1.258725000
I	-1.218671000	-0.693709000	2.340027000
I	2.953247000	-0.895300000	-2.219651000
C	-3.590359000	4.871491000	0.648745000
H	0.564141000	-4.289867000	1.138896000
H	-0.975274000	-3.389443000	1.185694000
H	-0.431405000	-3.186002000	-2.410602000
H	1.311620000	-3.440920000	-2.154028000
H	-1.926053000	-4.786816000	-0.583849000
H	-0.954680000	-5.995034000	0.290489000
H	-0.417905000	-5.609571000	-2.360693000
H	0.951932000	-5.653020000	-1.229529000
H	-2.328213000	-2.245363000	-1.706238000
H	-2.537914000	-0.969885000	-0.472125000
H	-0.800100000	1.157886000	-2.657074000
H	0.022271000	-0.180355000	-3.516349000
H	-3.438420000	0.580220000	-2.081118000
H	-4.065209000	-0.930366000	-2.770573000
H	-2.475608000	0.637029000	-4.349209000
H	-2.103041000	-1.102482000	-4.283643000
H	2.779132000	-1.092683000	3.152844000
H	1.349028000	-2.148032000	3.284047000
H	2.685088000	-3.999520000	0.636821000
H	3.676491000	-2.654970000	0.004819000
H	2.711467000	-4.148499000	3.396004000
H	4.708928000	-4.292501000	1.959517000
H	4.967392000	-2.535131000	2.070824000
H	1.096185000	2.405194000	1.840435000
H	1.261130000	1.304282000	3.233258000
H	4.346986000	0.331235000	1.839768000
H	3.900623000	0.857075000	0.187703000
H	3.462876000	2.158363000	3.779478000
H	2.749548000	3.645495000	3.111681000
H	5.013136000	2.670682000	1.937379000
H	3.665298000	3.163619000	0.879790000
H	0.746460000	5.427194000	-0.035919000
H	2.318274000	1.983889000	-2.084961000
H	2.753825000	6.727744000	-0.675238000
H	4.324351000	3.276556000	-2.727032000
H	4.561252000	5.662762000	-2.024128000
H	-1.101941000	4.470420000	-0.853682000
H	-2.110522000	1.750209000	0.248744000
H	-3.055004000	2.703034000	-0.921924000
H	-2.894476000	3.937432000	3.151632000
H	-2.008169000	2.473730000	2.621654000
H	-1.320036000	4.092142000	2.353193000
H	-4.218069000	5.262706000	1.466670000
H	-2.841474000	5.660704000	0.431373000
H	-4.220476000	4.766419000	-0.248458000
H	-5.007574000	3.147816000	2.379024000
H	-4.579912000	-1.105627000	1.431184000
H	-5.306650000	-3.114237000	0.162057000
H	-6.811485000	-2.874895000	-1.813489000
H	-7.584913000	-0.608438000	-2.506364000
H	-6.872979000	1.397663000	-1.229118000
H	3.817379000	-3.015859000	4.204098000

IIIa

SCF Energy = -3029.458298

Free Enthalpy (0K) = -3028.638302

Free Energy (298K) = -3028.735588

Free Energy (TZVP,PCM(THF),GD3BJ) = -3030.808275

C	6.472962000	0.456999000	0.913828000
C	5.519920000	0.393563000	-0.150860000
C	5.228075000	-0.893062000	-0.704809000
C	5.855481000	-2.027248000	-0.218017000
C	6.785901000	-1.943383000	0.827424000
C	7.086033000	-0.691993000	1.383206000
C	4.886066000	1.527081000	-0.617227000
C	4.442662000	2.601100000	-1.210710000
C	3.172275000	3.388813000	-0.917177000
C	2.349592000	3.454907000	-2.209230000
C	2.361152000	2.699720000	0.203065000
C	1.092455000	3.394792000	0.570813000
C	-0.136444000	2.818229000	0.650587000
C	-1.338937000	3.642743000	0.970584000
C	-1.467294000	4.966365000	0.520676000
C	-2.602801000	5.716784000	0.818602000
C	-3.637550000	5.158359000	1.569909000
C	-3.525044000	3.841426000	2.015940000
C	-2.391082000	3.090070000	1.714354000
O	-0.354736000	1.524977000	0.473095000
Sm	-0.809829000	-0.513214000	0.011398000
O	-0.298332000	-3.024636000	0.497472000
C	0.217319000	-3.952352000	-0.467618000
C	0.743810000	-5.119293000	0.346205000
C	-0.237829000	-5.148209000	1.512665000
C	-0.465990000	-3.667947000	1.772274000
O	-2.292981000	-2.101317000	-1.414601000
C	-3.245928000	-3.037963000	-0.891385000
C	-4.235302000	-3.265047000	-2.018115000

C	-3.328940000	-3.176576000	-3.240085000
C	-2.379971000	-2.054196000	-2.850893000
O	0.983579000	-0.751880000	1.728509000
C	2.271386000	-1.364461000	1.541343000
C	3.186338000	-0.695527000	2.552262000
C	2.218174000	-0.341803000	3.675704000
C	0.992313000	0.093481000	2.896280000
O	-2.304387000	0.802984000	-1.546556000
C	-1.748702000	1.796974000	-2.429868000
C	-2.939060000	2.599081000	-2.927865000
C	-3.904124000	2.508415000	-1.750279000
C	-3.686962000	1.082775000	-1.277076000
I	1.229869000	-0.742843000	-2.312467000
I	-2.966445000	-0.817933000	2.235501000
C	3.601781000	4.803066000	-0.503896000
H	-0.602446000	-4.265828000	-1.138272000
H	0.971738000	-3.433334000	-1.075246000
H	0.278804000	-3.253896000	2.472625000
H	-1.465173000	-3.420775000	2.158230000
H	1.765238000	-4.909788000	0.703870000
H	0.775379000	-6.054539000	-0.230974000
H	0.141975000	-5.674378000	2.400054000
H	-1.177946000	-5.638304000	1.210700000
H	2.174264000	-2.447138000	1.733823000
H	2.582366000	-1.222923000	0.496395000
H	1.060865000	1.143115000	2.568365000
H	0.039510000	-0.049559000	3.426186000
H	3.625766000	0.216297000	2.119887000
H	4.015514000	-1.348327000	2.859373000
H	2.588875000	0.449291000	4.342975000
H	1.993019000	-1.227321000	4.293178000
H	-2.771338000	-1.065237000	-3.137960000
H	-1.367962000	-2.155585000	-3.269613000
H	-2.722858000	-3.974224000	-0.624923000
H	-3.683124000	-2.614092000	0.023607000
H	-2.778208000	-4.121478000	-3.377292000
H	-4.766579000	-4.223180000	-1.926408000
H	-4.988594000	-2.460505000	-2.033817000
H	-1.040152000	2.412459000	-1.854036000
H	-1.188472000	1.278755000	-3.221982000
H	-4.309000000	0.364145000	-1.842445000
H	-3.873831000	0.935769000	-0.202900000
H	-3.376268000	2.131361000	-3.825549000
H	-2.662222000	3.630830000	-3.186639000
H	-4.951327000	2.703192000	-2.022690000
H	-3.617413000	3.218178000	-0.958589000
H	-0.673841000	5.404294000	-0.089764000
H	-2.317693000	2.056077000	2.057823000
H	-2.684528000	6.743437000	0.451802000
H	-4.326925000	3.389223000	2.605562000
H	-4.529386000	5.746265000	1.801570000
H	1.148123000	4.457362000	0.820537000
H	2.136245000	1.668628000	-0.111000000
H	3.026602000	2.624498000	1.086017000
H	2.925880000	3.924751000	-3.022532000
H	2.049535000	2.446915000	-2.534399000
H	1.436785000	4.049981000	-2.049080000
H	4.247742000	5.256915000	-1.272350000
H	2.730293000	5.463229000	-0.376324000
H	4.164713000	4.786917000	0.442625000
H	5.047880000	3.019787000	-2.035894000
H	4.497614000	-0.960955000	-1.514363000
H	5.621884000	-2.999096000	-0.661362000
H	7.278027000	-2.844077000	1.201490000
H	7.815634000	-0.618575000	2.194155000
H	6.711585000	1.430467000	1.347586000
H	-3.864166000	-2.963797000	-4.176551000

TS(III-IV)a

SCF Energy = -3029.451123
Free Enthalpy (0K) = -3028.630901
Free Energy (298K) = -3028.726278
Free Energy (TZVP,PCM(THF),GD3BJ) = -3030.801229

C	3.326248000	4.799872000	-0.153793000
C	3.769247000	4.159857000	-1.313180000
C	3.373724000	2.854647000	-1.592026000
C	2.525414000	2.150281000	-0.717199000
C	2.081138000	2.811188000	0.439956000
C	2.479878000	4.116401000	0.719011000
C	2.080647000	0.760226000	-0.985352000
C	2.837991000	-0.090895000	-1.754911000
C	2.404901000	-1.455685000	-2.182152000
C	3.579022000	-2.422919000	-2.413185000
C	4.555444000	-2.257320000	-1.265669000
C	4.460495000	-1.288222000	-0.376655000
C	5.018012000	-0.755254000	0.815214000
O	0.954978000	0.376517000	-0.410029000
Sm	-1.112855000	-0.006506000	0.028551000
O	-1.225936000	2.010731000	-1.461620000
C	-0.642208000	2.049262000	-2.776877000
C	-0.243812000	3.498862000	-2.991393000
C	-1.297778000	4.242721000	-2.178074000
C	-1.443610000	3.341583000	-0.965205000
I	-1.680439000	-1.778671000	-2.440387000
I	-1.131313000	1.673708000	2.614961000
O	-2.797458000	-1.541046000	1.304970000
C	-3.831954000	-2.329139000	0.695107000
C	-4.225526000	-3.354061000	1.743219000
C	-4.011184000	-2.574672000	3.035389000
C	-2.751481000	-1.786028000	2.721294000
O	0.062699000	-1.891192000	1.168748000
C	-0.033305000	-3.296886000	0.892507000
C	1.192665000	-3.925104000	1.543377000
C	1.508915000	-2.941256000	2.664849000
C	1.217295000	-1.617795000	1.989289000
O	-3.542335000	0.862351000	-0.237392000
C	-4.170647000	1.071336000	-1.510576000
C	-5.656391000	1.055573000	-1.213375000
C	-5.698309000	1.754427000	0.141069000
C	-4.442745000	1.226547000	0.823468000
C	3.067114000	-3.864853000	-2.471800000
H	-4.679958000	-1.668598000	0.441222000
H	-3.441033000	-2.753958000	-0.240427000
H	-1.840042000	-2.365117000	2.947047000
H	-2.676461000	-0.819408000	3.239888000
H	-3.554054000	-4.227407000	1.701416000
H	-5.254356000	-3.717647000	1.608823000
H	-3.895483000	-3.210948000	3.924420000
H	-4.858758000	-1.894108000	3.219476000
H	-0.971472000	-3.671714000	1.336421000
H	-0.088096000	-3.444361000	-0.196966000
H	2.046488000	-1.290666000	1.341298000
H	0.958311000	-0.797175000	2.673457000

H	2.030177000	-3.959059000	0.828830000
H	1.001004000	-4.950722000	1.889986000
H	2.544086000	-3.002590000	3.027432000
H	0.837026000	-3.095501000	3.525629000
H	-3.854820000	2.048346000	-1.917254000
H	-3.829051000	0.280727000	-2.194122000
H	-4.645557000	0.325217000	1.423931000
H	-3.947159000	1.958948000	1.477422000
H	-6.019609000	0.017905000	-1.132559000
H	-6.248427000	1.560427000	-1.990103000
H	-6.607779000	1.543753000	0.721764000
H	-5.636096000	2.846442000	0.007128000
H	0.200633000	1.344761000	-2.807776000
H	-1.402244000	1.712668000	-3.502498000
H	-2.435673000	3.374924000	-0.491184000
H	-0.686221000	3.563214000	-0.195933000
H	-0.233150000	3.774844000	-4.055514000
H	0.759394000	3.684396000	-2.576911000
H	-2.247445000	4.304723000	-2.735440000
H	-0.998773000	5.263761000	-1.902020000
H	3.709545000	2.383345000	-2.518457000
H	1.419509000	2.285763000	1.131314000
H	4.422443000	4.686840000	-2.013897000
H	2.125555000	4.599887000	1.633328000
H	3.636191000	5.825181000	0.063290000
H	3.706303000	0.341523000	-2.255745000
C	4.308512000	-2.101730000	-3.726342000
H	1.798563000	-1.404085000	-3.106546000
H	1.729938000	-1.865426000	-1.414746000
H	5.184865000	-2.755935000	-3.859644000
H	3.640260000	-2.252529000	-4.589565000
H	4.667273000	-1.061701000	-3.747140000
H	3.891593000	-4.575252000	-2.645059000
H	2.568368000	-4.147026000	-1.531542000
H	2.338247000	-3.986615000	-3.288933000
C	5.485228000	0.578923000	0.878393000
C	5.994952000	1.097557000	2.061405000
C	6.051930000	0.313852000	3.217040000
C	5.592314000	-1.004439000	3.175307000
C	5.078313000	-1.533219000	1.997458000
H	5.426254000	1.204436000	-0.014467000
H	6.345526000	2.132484000	2.085955000
H	6.448434000	0.729933000	4.146205000
H	5.639213000	-1.627649000	4.072582000
H	4.727408000	-2.567628000	1.962917000
H	5.410736000	-2.955910000	-1.240229000

IVa

SCF Energy = -3029.513049

Free Enthalpy (0K) = -3028.688917

Free Energy (298K) = -3028.781669

Free Energy (TZVP,PCM(THF),GD3BJ) = -3030.856835

C	2.580605000	2.451922000	-1.208637000
C	3.211665000	1.083471000	-0.886108000
C	3.593144000	1.265876000	0.584124000
C	3.589166000	2.569865000	0.907162000
C	3.216437000	3.472482000	-0.243176000
C	2.323765000	-0.086780000	-1.237700000
C	2.738592000	-1.139146000	-2.118323000
C	4.029830000	-1.208714000	-2.719902000
C	4.384318000	-2.257109000	-3.556218000
C	3.481715000	-3.286702000	-3.844935000
C	2.204040000	-3.236420000	-3.275215000
C	1.838371000	-2.196333000	-2.435408000
H	3.912772000	2.968402000	1.873162000
H	1.505929000	2.375902000	-0.985035000
O	1.104763000	-0.097798000	-0.732800000
Sm	-0.924650000	-0.047407000	-0.005483000
O	-0.071676000	-2.067315000	1.168092000
C	1.204520000	-2.019957000	1.842061000
C	1.730788000	-3.439580000	1.780237000
C	0.444869000	-4.254851000	1.854102000
C	-0.496370000	-3.430003000	0.994137000
I	-0.222886000	1.874854000	2.270536000
I	-2.187897000	-1.994226000	-2.068026000
O	-3.230353000	1.217438000	-0.130030000
C	-3.773373000	2.029665000	0.923532000
C	-4.973513000	2.734163000	0.314012000
C	-5.454571000	1.711394000	-0.708450000
C	-4.142941000	1.160155000	-1.238128000
O	-0.953735000	1.684444000	-1.826694000
C	-1.186166000	3.094446000	-1.694522000
C	-0.668550000	3.715901000	-2.983523000
C	-0.834657000	2.576679000	-3.984767000
C	-0.462143000	1.370948000	-3.144506000
O	-2.693010000	-0.988203000	1.684925000
C	-2.424748000	-1.177235000	3.085097000
C	-3.734863000	-1.654469000	3.688665000
C	-4.361367000	-2.410783000	2.523112000
C	-3.977198000	-1.531241000	1.347385000
H	-4.065756000	1.372199000	1.760510000
H	-2.986273000	2.703178000	1.290597000
H	-3.745855000	1.781424000	-2.060070000
H	-4.191085000	0.122181000	-1.596690000
H	-4.661999000	3.664128000	-0.189173000
H	-5.729439000	2.999970000	1.066734000
H	-6.080583000	2.139489000	-1.504386000
H	-6.038474000	0.916671000	-0.215710000
H	-2.269219000	3.258850000	-1.567786000
H	-0.674786000	3.459496000	-0.790928000
H	0.628618000	1.223887000	-3.091328000
H	-0.928980000	0.426743000	-3.459192000
H	0.395167000	3.979903000	-2.879781000
H	-1.217923000	4.628655000	-3.255238000
H	-0.197016000	2.678738000	-4.874207000
H	-1.880720000	2.503825000	-4.325189000
H	-1.628556000	-1.933071000	3.188366000
H	-2.052209000	-0.228844000	3.498491000
H	-4.696010000	-0.702243000	1.222573000
H	-3.890256000	-2.062710000	0.388463000
H	-4.366064000	-0.797089000	3.974482000
H	-3.581692000	-2.270093000	4.586530000
H	-5.449233000	-2.543153000	2.611422000
H	-3.909004000	-3.410963000	2.421626000
H	1.836046000	-1.284323000	1.330890000
H	1.039324000	-1.680161000	2.878798000
H	-1.555722000	-3.504966000	1.281413000
H	-0.415785000	-3.689386000	-0.073308000
H	2.443461000	-3.651474000	2.588883000
H	2.244832000	-3.610681000	0.821076000
H	0.081184000	-4.308883000	2.893837000
H	0.550408000	-5.283196000	1.479841000
H	4.768113000	-0.426024000	-2.534780000
H	0.829936000	-2.169300000	-2.022426000

H	5.385363000	-2.271002000	-3.996484000
H	1.474355000	-4.020144000	-3.497924000
H	3.767136000	-4.107578000	-4.506690000
H	4.149117000	0.978995000	-1.455110000
C	4.033211000	0.177616000	1.468719000
C	3.839440000	0.261423000	2.859079000
C	4.301103000	-0.739737000	3.708257000
C	4.962079000	-1.855016000	3.189487000
C	5.144730000	-1.961288000	1.811311000
C	4.679646000	-0.961119000	0.958958000
H	3.292445000	1.114245000	3.267696000
H	4.138289000	-0.651717000	4.785770000
H	5.328449000	-2.639278000	3.857014000
H	5.655225000	-2.831472000	1.390566000
H	4.830084000	-1.066626000	-0.117182000
H	2.698613000	2.731771000	-2.268162000
C	4.489590000	4.092110000	-0.840128000
C	2.245496000	4.581298000	0.161267000
H	2.719985000	5.288696000	0.860849000
H	1.920910000	5.159061000	-0.720776000
H	1.356487000	4.163009000	0.657843000
H	5.017273000	4.706702000	-0.093458000
H	5.189885000	3.316359000	-1.186875000
H	4.245201000	4.741698000	-1.696946000

3a

SCF Energy = -3029.540624
Free Enthalpy (0K) = -3028.716374
Free Energy (298K) = -3028.814471
Free Energy (TZVP,PCM(THF),GD3BJ) = -3030.887486

C	3.552178000	-1.908879000	-1.273291000
C	3.679528000	-0.910069000	-0.088269000
C	3.942057000	0.420113000	-0.775906000
C	4.156584000	0.241440000	-2.088138000
C	4.117272000	-1.197358000	-2.532114000
C	2.430872000	-0.987591000	0.798825000
C	2.454490000	-1.957309000	1.921389000
C	3.541597000	-2.816489000	2.168240000
C	3.502450000	-3.720028000	3.224748000
C	2.382014000	-3.779830000	4.053709000
C	1.298008000	-2.933946000	3.818391000
C	1.333292000	-2.032720000	2.762649000
H	4.417583000	1.040759000	-2.785723000
H	2.492193000	-2.165284000	-1.419305000
O	1.388333000	-0.367842000	0.577435000
Sm	-1.085764000	0.096809000	-0.026731000
O	-0.611616000	1.747327000	1.940198000
C	0.485395000	2.657070000	2.019193000
C	0.585092000	3.023899000	3.489648000
C	-0.877374000	2.999490000	3.916704000
C	-1.416714000	1.820694000	3.124510000
I	0.103147000	2.070406000	-2.303690000
I	-2.460583000	-1.867837000	2.164134000
O	-2.888490000	-0.345030000	-1.901655000
C	-3.107971000	0.387039000	-3.104462000
C	-4.159931000	-0.380559000	-3.892321000
C	-4.951059000	-1.054764000	-2.772642000
C	-3.856661000	-1.393521000	-1.778658000
O	-0.515708000	-2.115350000	-1.339027000
C	-0.372220000	-2.198127000	-2.759626000
C	0.246665000	-3.557749000	-3.031587000
C	-0.338763000	-4.390267000	-1.895864000
C	-0.309429000	-3.404092000	-0.740440000
O	-2.827917000	1.452005000	0.355041000
C	-2.521515000	2.797301000	0.752422000
C	-3.817090000	3.572249000	0.631801000
C	-4.833556000	2.500831000	0.982717000
C	-4.238672000	1.267926000	0.315569000
H	-3.496050000	1.374205000	-2.781670000
H	-2.152425000	0.523331000	-3.619464000
H	-3.374683000	-2.353785000	-2.040094000
H	-4.181117000	-1.453575000	-0.728848000
H	-3.681851000	-1.128156000	-4.547030000
H	-4.778476000	0.267769000	-4.528647000
H	-5.520637000	-1.937593000	-3.096752000
H	-5.661429000	-0.341385000	-2.324307000
H	-1.371398000	-2.116857000	-3.221055000
H	0.221535000	-1.341570000	-3.104610000
H	0.669613000	-3.411096000	-0.230199000
H	-1.089141000	-3.566649000	0.017245000
H	1.343666000	-3.507720000	-2.947456000
H	0.003824000	-3.938257000	-4.034135000
H	0.226199000	-5.308285000	-1.681242000
H	-1.376667000	-4.679787000	-2.128474000
H	-2.160732000	2.776791000	1.797730000
H	-1.711485000	3.171130000	0.114490000
H	-4.613711000	1.184388000	-0.732991000
H	-4.493144000	0.329265000	0.833953000
H	-3.964704000	3.925675000	-0.401792000
H	-3.848929000	4.449510000	1.293279000
H	-5.854198000	2.714299000	0.633256000
H	-4.875624000	2.368543000	2.076278000
H	1.377213000	2.160218000	1.622032000
H	0.286776000	3.541742000	1.389886000
H	-2.472477000	1.932488000	2.831509000
H	-1.327232000	0.865426000	3.667827000
H	1.077348000	3.992203000	3.650098000
H	1.161708000	2.257666000	4.032813000
H	-1.377851000	3.933703000	3.613479000
H	-1.022192000	2.877892000	4.999684000
H	4.426062000	-2.791894000	1.529932000
H	0.473695000	-1.384450000	2.580390000
H	4.352162000	-4.383111000	3.402172000
H	0.410268000	-2.974807000	4.453521000
H	2.354204000	-4.489694000	4.884026000
H	4.548717000	-1.145842000	0.545234000
C	4.117965000	1.687688000	-0.049765000
C	3.945920000	2.922112000	-0.697433000
C	4.201714000	4.115946000	-0.030980000
C	4.633048000	4.108878000	1.296514000
C	4.789988000	2.893596000	1.959754000
C	4.521934000	1.696531000	1.297799000
H	3.572640000	2.940553000	-1.723712000
H	4.054785000	5.064647000	-0.553681000
H	4.838340000	5.049533000	1.814326000
H	5.120671000	2.874728000	3.001431000
H	4.660526000	0.754778000	1.833621000
H	4.077594000	-2.856160000	-1.073159000
C	5.543521000	-1.689782000	-2.859501000
C	3.235230000	-1.342652000	-3.786187000
H	3.517197000	-0.597721000	-4.546318000
H	3.332810000	-2.340288000	-4.248276000
H	2.182905000	-1.163717000	-3.541104000
H	6.087554000	-0.950639000	-3.470103000

H	6.134937000	-1.859450000	-1.945665000
H	5.512965000	-2.633129000	-3.428950000

1b

SCF Energy = -2760.876711

Free Enthalpy (0K) = -2760.142314

Free Energy (298K) = -2760.236455

Free Energy (TZVP,PCM(THF),GD3BJ) = -2762.010309

C	-4.807868000	-2.708283000	2.010741000
C	-5.354068000	-2.238272000	0.819176000
C	-4.833815000	-1.123381000	0.149019000
C	-3.710170000	-0.477727000	0.719124000
C	-3.142762000	-0.984388000	1.900448000
C	-3.688440000	-2.081522000	2.553869000
C	-3.011047000	0.705630000	0.129137000
C	-3.802392000	1.773221000	-0.509881000
C	-3.200534000	2.566784000	-1.660713000
C	-3.461430000	3.271370000	-0.383053000
C	-4.672854000	4.174551000	-0.310444000
O	-1.785530000	0.781287000	0.222344000
Sm	0.635884000	-0.009646000	-0.032032000
O	-0.878217000	-1.816874000	-1.214657000
C	-1.599068000	-1.677493000	-2.441310000
C	-2.472903000	-2.920390000	-2.547821000
C	-1.659104000	-3.951403000	-1.772252000
C	-1.105762000	-3.106427000	-0.638020000
I	0.774142000	1.642382000	-2.796619000
I	0.734867000	-1.697898000	2.733343000
O	3.198520000	0.627729000	0.322039000
C	4.128064000	0.872599000	-0.736295000
C	5.375397000	1.412023000	-0.056093000
C	5.343142000	0.671814000	1.277767000
C	3.855213000	0.664763000	1.591934000
O	0.811004000	2.176858000	1.428866000
C	1.432246000	3.383006000	0.973052000
C	1.161516000	4.415107000	2.057583000
C	1.089921000	3.546250000	3.310100000
C	0.380182000	2.308862000	2.787628000
O	2.179637000	-1.925516000	-1.008717000
C	2.171358000	-2.306411000	-2.385515000
C	3.435765000	-3.125924000	-2.581333000
C	3.571662000	-3.803548000	-1.220942000
C	3.141197000	-2.691505000	-0.277656000
H	4.336783000	-0.078676000	-1.259053000
H	3.662487000	1.557569000	-1.460615000
H	3.555449000	1.586098000	2.123745000
H	3.514840000	-0.194602000	2.189954000
H	5.287436000	2.499289000	0.104563000
H	6.288473000	1.233070000	-0.641934000
H	5.939309000	1.151455000	2.067379000
H	5.717903000	-0.357806000	1.154972000
H	2.513532000	3.197866000	0.848757000
H	1.022420000	3.644049000	-0.014768000
H	-0.716978000	2.436712000	2.804172000
H	0.625891000	1.378040000	3.321227000
H	0.194479000	4.913327000	1.882581000
H	1.937413000	5.192971000	2.100879000
H	0.555278000	4.019218000	4.146372000
H	2.102907000	3.289665000	3.661780000
H	1.270325000	-2.913363000	-2.586046000
H	2.114918000	-1.394012000	-2.999478000
H	3.992384000	-2.037890000	-0.015612000
H	2.677144000	-3.033164000	0.660013000
H	4.298027000	-2.465685000	-2.771377000
H	3.359617000	-3.829031000	-3.423254000
H	4.585632000	-4.170613000	-1.006401000
H	2.882196000	-4.660710000	-1.148439000
H	-2.167263000	-0.734638000	-2.421750000
H	-0.876636000	-1.602834000	-3.271929000
H	-0.156813000	-3.468137000	-0.216247000
H	-1.830390000	-3.015037000	0.190077000
H	-2.672511000	-3.205632000	-3.590755000
H	-3.441240000	-2.756238000	-2.048817000
H	-0.842201000	-4.349201000	-2.396961000
H	-2.254297000	-4.802844000	-1.412450000
C	-5.473065000	-0.730259000	-1.157730000
H	-2.243992000	-0.510603000	2.301464000
H	-6.209606000	-2.759466000	0.380099000
H	-3.222971000	-2.450643000	3.470220000
H	-5.248553000	-3.578387000	2.504038000
H	-4.873997000	1.585782000	-0.549731000
C	-2.306849000	3.733642000	0.469094000
H	-3.854149000	2.759090000	-2.516553000
H	-2.161110000	2.319941000	-1.908959000
H	-1.967723000	4.721021000	0.115873000
H	-2.618212000	3.842557000	1.520950000
H	-1.460178000	3.039169000	0.424842000
H	-5.053671000	4.247376000	0.720880000
H	-4.406795000	5.191166000	-0.642603000
H	-5.491674000	3.817239000	-0.953033000
H	-5.976228000	-1.598686000	-1.606692000
H	-6.244941000	0.045207000	-1.020076000
H	-4.749518000	-0.343616000	-1.888362000

Ib

SCF Energy = -2760.843624

Free Enthalpy (0K) = -2760.109049

Free Energy (298K) = -2760.196958

Free Energy (TZVP,PCM(THF),GD3BJ) = -2761.97088

C	-4.941918000	-2.890499000	1.633764000
C	-5.510379000	-2.149977000	0.594485000
C	-4.866810000	-1.069921000	-0.012101000
C	-3.557567000	-0.696693000	0.448617000
C	-2.998384000	-1.471647000	1.506472000
C	-3.668955000	-2.535039000	2.087177000
C	-2.746404000	0.369850000	-0.078468000
C	-3.233433000	1.491865000	-0.938702000
C	-2.556085000	2.824551000	-0.837563000
C	-3.976832000	2.698226000	-0.370313000
C	-5.047401000	3.338928000	-1.222354000
O	-1.465453000	0.402373000	0.253544000
Sm	0.632394000	0.019185000	0.012720000
O	-0.403209000	-2.042074000	-0.976644000
C	-1.378020000	-2.031229000	-2.036836000
C	-2.172769000	-3.312736000	-1.859689000
C	-1.134273000	-4.254355000	-1.259458000
C	-0.379209000	-3.324803000	-0.327368000
I	0.680898000	1.285252000	-2.792244000
I	1.039695000	-1.346375000	2.753862000
O	3.055055000	0.934981000	0.299074000
C	3.961675000	1.223553000	-0.774708000
C	5.023368000	2.118415000	-0.164300000
C	5.124338000	1.550652000	1.246977000
C	3.673469000	1.225032000	1.565720000

O	0.666174000	2.230415000	1.215522000
C	1.046349000	3.494429000	0.650041000
C	0.290248000	4.540064000	1.454267000
C	0.160053000	3.865854000	2.816577000
C	-0.088956000	2.420738000	2.427268000
O	2.421449000	-1.677078000	-0.838218000
C	2.463379000	-2.174658000	-2.185220000
C	3.825263000	-2.829103000	-2.322962000
C	4.041186000	-3.381848000	-0.918679000
C	3.466366000	-2.272922000	-0.053125000
H	4.400025000	0.277887000	-1.141154000
H	3.389680000	1.674790000	-1.597529000
H	3.149107000	2.082595000	2.018458000
H	3.539737000	0.360519000	2.232323000
H	4.677373000	3.164745000	-0.139153000
H	5.970058000	2.090899000	-0.722549000
H	5.565784000	2.245397000	1.975756000
H	5.736879000	0.634167000	1.247515000
H	2.138976000	3.612344000	0.758254000
H	0.803269000	3.488293000	-0.423275000
H	-1.150388000	2.222347000	2.207478000
H	0.255855000	1.679514000	3.162380000
H	-0.705031000	4.715387000	1.016516000
H	0.818556000	5.503702000	1.486101000
H	-0.651100000	4.276844000	3.434075000
H	1.098816000	3.957747000	3.387292000
H	1.650000000	-2.907764000	-2.320039000
H	2.285277000	-1.332736000	-2.870224000
H	4.223416000	-1.501552000	0.169825000
H	3.037387000	-2.612242000	0.900941000
H	4.594131000	-2.077440000	-2.566697000
H	3.844788000	-3.596335000	-3.110091000
H	5.092949000	-3.596075000	-0.680877000
H	3.470515000	-4.314845000	-0.781840000
H	-1.987652000	-1.122386000	-1.942547000
H	-0.839118000	-1.994257000	-2.999003000
H	0.671117000	-3.603669000	-0.158487000
H	-0.877252000	-3.235803000	0.651128000
H	-2.598315000	-3.673336000	-2.807119000
H	-2.998337000	-3.150774000	-1.148597000
H	-0.467965000	-4.651989000	-2.043517000
H	-1.574520000	-5.107296000	-0.723871000
C	-5.565515000	-0.401950000	-1.163250000
H	-2.012865000	-1.191157000	1.880927000
H	-6.501941000	-2.428729000	0.224255000
H	-3.197315000	-3.083225000	2.907635000
H	-5.486643000	-3.726694000	2.078217000
H	-3.541039000	1.216689000	-1.953982000
C	-4.257484000	2.736343000	1.112321000
H	-2.347422000	3.368260000	-1.762741000
H	-1.770395000	2.900229000	-0.083558000
H	-4.343228000	3.776717000	1.468841000
H	-5.202533000	2.221281000	1.350836000
H	-3.464692000	2.237856000	1.689037000
H	-6.036315000	2.883777000	-1.045260000
H	-5.136578000	4.414211000	-0.993402000
H	-4.819961000	3.244267000	-2.295318000
H	-6.527954000	-0.895310000	-1.360702000
H	-5.766189000	0.662594000	-0.977951000
H	-4.972729000	-0.455030000	-2.090493000

TS(I-II)b

SCF Energy = -2760.816892
Free Enthalpy (0K) = -2760.084473
Free Energy (298K) = -2760.172421
Free Energy (TZVP,PCM(THF),GD3BJ) = -2722.698132

C	-5.358904000	-2.437057000	1.818555000
C	-5.903227000	-1.682025000	0.761950000
C	-5.126110000	-0.733547000	0.088433000
C	-3.767546000	-0.504117000	0.446839000
C	-3.236370000	-1.274973000	1.516504000
C	-4.020039000	-2.221116000	2.188990000
C	-2.904239000	0.468028000	-0.248768000
C	-3.435731000	1.449950000	-1.120922000
C	-2.615673000	2.502409000	-1.788892000
C	-2.997290000	3.342052000	-0.618665000
C	-4.072715000	4.387316000	-0.648437000
O	-1.601235000	0.457821000	0.002345000
Sm	0.561303000	-0.013339000	-0.055229000
O	-0.559074000	-2.067225000	-1.125779000
C	-1.450191000	-1.980400000	-2.273076000
C	-2.401533000	-3.168987000	-2.136957000
C	-1.524981000	-4.205814000	-1.419301000
C	-0.756250000	-3.329327000	-0.433401000
I	1.041299000	1.192309000	-2.902778000
I	0.644783000	-1.368850000	2.775839000
O	3.024259000	0.816792000	0.456373000
C	4.086392000	0.961261000	-0.527507000
C	5.279190000	1.550152000	0.230216000
C	5.048507000	1.031091000	1.656856000
C	3.532070000	1.138637000	1.777615000
O	0.546427000	2.264410000	1.109404000
C	1.079515000	3.513265000	0.589733000
C	0.943338000	4.548703000	1.726595000
C	0.512578000	3.711193000	2.947151000
C	-0.224561000	2.543996000	2.298396000
O	2.348131000	-1.871258000	-0.691264000
C	2.443900000	-2.482957000	-2.007442000
C	3.673748000	-3.389451000	-1.950117000
C	3.688732000	-3.808292000	-0.472917000
C	3.283191000	-2.508780000	0.216942000
H	4.311788000	-0.044772000	-0.943700000
H	3.714499000	1.593866000	-1.358609000
H	3.223246000	2.174763000	2.044991000
H	3.070169000	0.437993000	2.502113000
H	5.241903000	2.659746000	0.214839000
H	6.249495000	1.241294000	-0.205838000
H	5.579060000	1.615767000	2.433837000
H	5.372265000	-0.027538000	1.747816000
H	2.124716000	3.309889000	0.280760000
H	0.505795000	3.817286000	-0.310179000
H	-1.266595000	2.819876000	2.014943000
H	-0.255540000	1.612442000	2.897884000
H	0.170440000	5.294898000	1.456044000
H	1.887237000	5.100533000	1.904035000
H	-0.125977000	4.276424000	3.654265000
H	1.390850000	3.334170000	3.511954000
H	1.514409000	-3.063211000	-2.192765000
H	2.500216000	-1.675388000	-2.765653000
H	4.163355000	-1.840369000	0.359594000
H	2.777510000	-2.630984000	1.196257000
H	4.593986000	-2.819609000	-2.199010000
H	3.603667000	-4.240776000	-2.655367000
H	4.670534000	-4.185848000	-0.125736000

H	2.934126000	-4.601275000	-0.284004000
H	-1.960855000	-0.997728000	-2.250230000
H	-0.830297000	-2.031875000	-3.195649000
H	0.241054000	-3.719218000	-0.146408000
H	-1.339434000	-3.147373000	0.494991000
H	-2.785336000	-3.515536000	-3.116471000
H	-3.268719000	-2.893975000	-1.501906000
H	-0.831678000	-4.696717000	-2.135658000
H	-2.107626000	-4.997701000	-0.909394000
C	-5.744738000	0.026658000	-1.047080000
H	-2.213033000	-1.111950000	1.820817000
H	-6.949709000	-1.838630000	0.455695000
H	-3.575799000	-2.791961000	3.020057000
H	-5.973908000	-3.182235000	2.345914000
H	-4.516863000	1.454248000	-1.317130000
C	-2.179147000	3.165444000	0.626104000
H	-3.025816000	2.858393000	-2.752975000
H	-1.541251000	2.252441000	-1.879656000
H	-2.547205000	3.817643000	1.390348000
H	-2.249953000	2.150848000	0.958491000
H	-1.156673000	3.401861000	0.417408000
H	-4.150681000	4.846373000	0.314936000
H	-3.828030000	5.130093000	-1.378719000
H	-5.006570000	3.931103000	-0.902766000
H	-6.763491000	-0.277743000	-1.167008000
H	-5.708202000	1.075047000	-0.836260000
H	-5.203807000	-0.175052000	-1.947973000

IIb

SCF Energy = -2760.836033
Free Enthalpy (0K) = -2760.103805
Free Energy (298K) = -2760.193356
Free Energy (TZVP,PCM(THF),GD3BJ) = -2761.96787

C	-4.587063000	-3.512330000	1.468531000
C	-5.110456000	-2.920294000	0.323464000
C	-4.604694000	-1.718975000	-0.197403000
C	-3.523924000	-1.099088000	0.474709000
C	-2.988312000	-1.723222000	1.613755000
C	-3.511714000	-2.907883000	2.117682000
C	-2.865808000	0.169563000	0.032936000
C	-3.576918000	1.228991000	-0.431090000
C	-2.994095000	2.527216000	-0.894851000
C	-3.700018000	3.753508000	-0.393854000
C	-3.603084000	5.014693000	-1.181910000
O	-1.546300000	0.197234000	0.167539000
Sm	0.569587000	-0.032348000	0.013522000
O	-0.275008000	-2.119452000	-1.083472000
C	-1.197550000	-2.089967000	-2.188748000
C	-1.892420000	-3.442343000	-2.169827000
C	-0.839921000	-4.343044000	-1.532183000
C	-0.251240000	-3.421299000	-0.480567000
I	0.695133000	1.317268000	-2.769359000
I	0.909599000	-1.411670000	2.768589000
O	2.912050000	1.019751000	0.463620000
C	3.850830000	1.413641000	-0.546725000
C	4.774086000	2.401506000	0.139551000
C	4.854152000	1.815825000	1.544782000
C	3.434294000	1.317376000	1.771541000
O	0.376116000	2.193298000	1.164504000
C	0.799244000	3.465311000	0.651628000
C	-0.107565000	4.488548000	1.315166000
C	-0.418910000	3.820920000	2.650823000
C	-0.565216000	2.367428000	2.241903000
O	2.511523000	-1.580720000	-0.780449000
C	2.646754000	-2.058057000	-2.128405000
C	4.046587000	-2.638534000	-2.203853000
C	4.221110000	-3.194490000	-0.794893000
C	3.552643000	-2.121526000	0.047603000
H	4.407416000	0.523678000	-0.892814000
H	3.288799000	1.817465000	-1.400606000
H	2.792297000	2.086516000	2.230781000
H	3.371008000	0.414437000	2.396187000
H	4.317939000	3.404740000	0.159172000
H	5.749085000	2.485765000	-0.361420000
H	5.168884000	2.539131000	2.310559000
H	5.568054000	0.976301000	1.567812000
H	1.856189000	3.622769000	0.929448000
H	0.727066000	3.442398000	-0.446394000
H	-1.574255000	2.141618000	1.861681000
H	-0.316371000	1.642241000	3.030077000
H	-1.030767000	4.617462000	0.728976000
H	0.373367000	5.472409000	1.412276000
H	-1.326321000	4.209796000	3.134567000
H	0.420244000	3.946505000	3.354839000
H	1.881702000	-2.831732000	-2.310763000
H	2.456890000	-1.218345000	-2.813159000
H	4.258647000	-1.312336000	0.301675000
H	3.101852000	-2.484221000	0.982678000
H	4.785620000	-1.844743000	-2.401653000
H	4.145937000	-3.394817000	-2.995580000
H	5.269233000	-3.360828000	-0.507768000
H	3.689113000	-4.154482000	-0.692802000
H	-1.879756000	-1.238929000	-2.054598000
H	-0.616540000	-1.921641000	-3.110757000
H	0.787921000	-3.648405000	-0.201515000
H	-0.863103000	-3.405527000	0.436821000
H	-2.198850000	-3.766092000	-3.174777000
H	-2.789823000	-3.400701000	-1.532872000
H	-0.073082000	-4.632330000	-2.270235000
H	-1.255893000	-5.261988000	-1.095404000
C	-5.220826000	-1.186894000	-1.462816000
H	-2.130014000	-1.260517000	2.105513000
H	-5.938191000	-3.407260000	-0.201127000
H	-3.074409000	-3.356819000	3.013068000
H	-5.011486000	-4.447020000	1.844190000
H	-4.663728000	1.135450000	-0.471762000
C	-4.158539000	3.842016000	1.021377000
H	-2.993117000	2.559023000	-2.002057000
H	-1.916344000	2.541831000	-0.629269000
H	-4.455382000	5.686843000	-0.984929000
H	-2.689511000	5.598350000	-0.934206000
H	-3.560279000	4.819860000	-2.265403000
H	-5.095118000	4.420032000	1.110870000
H	-4.323417000	2.846660000	1.461222000
H	-3.422522000	4.366716000	1.668789000
H	-5.688698000	-2.002949000	-2.033375000
H	-6.010334000	-0.445670000	-1.255800000
H	-4.480745000	-0.684214000	-2.101600000

TS(II-III)b

SCF Energy = -3068.633335
Free Enthalpy (0K) = -3067.789044
Free Energy (298K) = -3067.888581
Free Energy (TZVP,PCM(THF),GD3BJ) = -3070.010974

C	3.889752000	0.613332000	1.133279000
O	2.493481000	0.550701000	1.458794000
C	2.124092000	1.658807000	2.303937000
C	3.425138000	2.382334000	2.620224000
C	4.288594000	2.049813000	1.407544000
Sm	0.771950000	-0.612522000	0.025217000
I	2.789667000	-1.142269000	-2.282446000
O	0.414164000	1.457060000	-0.387073000
C	0.274371000	2.750295000	-0.639783000
C	1.531894000	3.455614000	-1.045982000
C	1.941426000	4.706467000	-0.527155000
C	3.162201000	5.241966000	-0.965552000
C	3.970249000	4.585970000	-1.888959000
C	3.569065000	3.348276000	-2.389426000
C	2.368947000	2.795244000	-1.959434000
C	-0.917691000	3.398664000	-0.588004000
C	-2.227821000	2.816504000	-0.132578000
C	-2.832077000	3.645967000	0.969594000
C	-3.405593000	4.973797000	0.591349000
C	-2.228043000	3.524899000	2.330004000
O	-0.031851000	-3.064954000	-0.376745000
C	-0.620967000	-3.916190000	0.618407000
C	-1.231455000	-5.072013000	-0.152849000
C	-0.269235000	-5.204294000	-1.327354000
C	0.048062000	-3.751768000	-1.638088000
O	2.176847000	-2.311465000	1.398952000
C	2.963660000	-3.382977000	0.858411000
C	3.928764000	-3.765758000	1.965121000
C	3.085360000	-3.512581000	3.209071000
C	2.335959000	-2.247283000	2.827879000
O	-1.015425000	-0.709156000	-1.713287000
C	-2.374075000	-1.101846000	-1.452424000
C	-3.216824000	-0.327483000	-2.450276000
C	-2.255926000	-0.161254000	-3.621726000
C	-0.944136000	0.096490000	-2.905742000
I	-1.173603000	-0.635226000	2.433416000
C	-4.941475000	2.590442000	1.483750000
C	-5.318698000	1.600952000	0.861366000
C	-5.726314000	0.448813000	0.140514000
C	-6.550970000	0.561224000	-0.999092000
C	-6.982563000	-0.574999000	-1.674010000
C	-6.605842000	-1.845782000	-1.233482000
C	-5.787953000	-1.969311000	-0.107862000
C	-5.348379000	-0.840237000	0.574827000
H	0.171384000	-4.260817000	1.306295000
H	-1.339523000	-3.324980000	1.203274000
H	-0.691137000	-3.308688000	-2.326475000
H	1.047957000	-3.585927000	-2.064002000
H	-2.240760000	-4.807438000	-0.508675000
H	-1.318620000	-5.983390000	0.455918000
H	-0.692824000	-5.734259000	-2.192411000
H	0.642211000	-5.742318000	-1.018840000
H	-2.459297000	-2.192125000	-1.604863000
H	-2.610838000	-0.879999000	-0.401852000
H	-0.839775000	1.152926000	-2.608898000
H	-0.049177000	-0.207313000	-3.467836000
H	-3.486951000	0.654325000	-2.033215000
H	-4.150365000	-0.850059000	-2.700171000
H	-2.527627000	0.660030000	-4.300055000
H	-2.198482000	-1.087690000	-4.217128000
H	2.912602000	-1.340415000	3.071932000
H	1.340844000	-2.154506000	3.287302000
H	2.295429000	-4.224414000	0.602277000
H	3.444503000	-3.030797000	-0.065366000
H	2.382769000	-4.345971000	3.373853000
H	4.285461000	-4.801594000	1.872637000
H	4.809742000	-3.103311000	1.952773000
H	1.419588000	2.289541000	1.742146000
H	1.604294000	1.263655000	3.189153000
H	4.441149000	-0.093238000	1.782234000
H	4.016354000	0.300954000	0.086471000
H	3.880846000	1.982603000	3.541032000
H	3.271192000	3.461439000	2.762234000
H	5.366941000	2.158332000	1.592382000
H	4.018843000	2.687795000	0.551206000
C	1.153774000	5.482199000	0.493297000
H	2.069243000	1.813672000	-2.331533000
H	3.487295000	6.203034000	-0.555588000
H	4.190021000	2.805871000	-3.106636000
H	4.914286000	5.035991000	-2.206919000
H	-0.920605000	4.449662000	-0.886834000
H	-2.069379000	1.783918000	0.215142000
H	-2.945154000	2.778367000	-0.975035000
H	-2.881170000	3.950629000	3.110430000
H	-2.008674000	2.476579000	2.585193000
H	-1.271888000	4.083527000	2.387519000
H	-4.042468000	5.391418000	1.388979000
H	-2.605568000	5.721507000	0.414444000
H	-4.004346000	4.913610000	-0.331111000
H	-4.990151000	3.368149000	2.228100000
H	-4.707070000	-0.935598000	1.454082000
H	-5.492874000	-2.960287000	0.247419000
H	-6.953997000	-2.736509000	-1.762146000
H	-7.625432000	-0.469121000	-2.551698000
H	-6.852434000	1.554228000	-1.339047000
H	3.675400000	-3.386988000	4.128195000
H	1.816478000	6.151254000	1.061965000
H	0.382659000	6.114895000	0.023592000
H	0.627427000	4.820180000	1.195364000

IIIb

SCF Energy = -3068.690532
Free Enthalpy (0K) = -3067.843552
Free Energy (298K) = -3067.945027
Free Energy (TZVP,PCM(THF),GD3BJ) = -3070.06338

C	-4.854689000	-1.917875000	-0.324246000
C	-5.505844000	-0.658449000	-0.126620000
C	-6.432076000	-0.553968000	0.959501000
C	-6.678841000	-1.636956000	1.785168000
C	-6.029861000	-2.861905000	1.575813000
C	-5.120395000	-2.985916000	0.515653000
C	-5.247384000	0.414666000	-0.951414000
C	-4.932289000	1.450643000	-1.679269000
C	-3.830368000	2.470540000	-1.395154000
C	-4.515212000	3.809952000	-1.093711000
C	-2.982289000	1.983531000	-0.201560000
C	-1.942889000	2.926814000	0.303819000
C	-0.638414000	2.629509000	0.541126000
C	0.272571000	3.647299000	1.156191000
C	0.398238000	4.977555000	0.693098000
C	1.299596000	5.828251000	1.350271000
C	2.061362000	5.403603000	2.434241000
C	1.943997000	4.088394000	2.879918000
C	1.064282000	3.226720000	2.236403000

C	-0.366086000	5.521579000	-0.483095000
O	-0.097436000	1.436311000	0.332111000
Sm	0.908384000	-0.419981000	-0.042317000
O	2.632335000	-1.673180000	-1.565333000
C	2.338900000	-2.736710000	-2.483786000
C	3.556119000	-2.836240000	-3.385604000
C	4.678608000	-2.437788000	-2.434661000
C	4.024945000	-1.323989000	-1.636383000
O	1.027705000	-2.969566000	0.494206000
C	2.184476000	-3.657742000	0.991911000
C	1.657700000	-4.960964000	1.564590000
C	0.485552000	-5.255440000	0.635240000
C	-0.090170000	-3.868592000	0.401740000
O	2.135208000	1.140171000	-1.589024000
C	1.825086000	1.274628000	-2.979961000
C	1.772474000	2.770864000	-3.213099000
C	2.899859000	3.259874000	-2.308604000
C	2.792055000	2.331483000	-1.104919000
O	-0.550392000	-1.027052000	1.922119000
C	-1.885821000	-0.489683000	2.043409000
C	-2.407043000	-1.017850000	3.366832000
C	-1.129443000	-1.098760000	4.194498000
C	-0.128636000	-1.599571000	3.169308000
I	-1.159289000	-1.272187000	-2.199381000
I	3.256816000	-0.076897000	1.955873000
C	-2.963773000	2.603694000	-2.651130000
H	2.185303000	-3.669151000	-1.912348000
H	1.400451000	-2.499654000	-3.004363000
H	4.117955000	-0.350778000	-2.146357000
H	4.407488000	-1.211182000	-0.611542000
H	3.480305000	-2.115393000	-4.216085000
H	3.675482000	-3.838709000	-3.821025000
H	5.595744000	-2.106384000	-2.942386000
H	4.945279000	-3.281943000	-1.777564000
H	2.625209000	0.800876000	-3.580435000
H	0.880213000	0.747497000	-3.177000000
H	2.170658000	2.755267000	-0.303315000
H	3.763247000	2.046628000	-0.673797000
H	0.801158000	3.166526000	-2.876154000
H	1.905343000	3.040894000	-4.270525000
H	2.802219000	4.315485000	-2.018299000
H	3.872303000	3.139142000	-2.813010000
H	-0.826389000	-3.592833000	1.175786000
H	-0.565892000	-3.733189000	-0.580154000
H	2.880542000	-3.839868000	0.154055000
H	2.691091000	-3.005896000	1.718041000
H	0.846049000	-5.689137000	-0.311903000
H	2.421330000	-5.751756000	1.580371000
H	1.305314000	-4.814082000	2.598598000
H	-1.816516000	0.608392000	2.033392000
H	-2.472996000	-0.814649000	1.172801000
H	-0.160763000	-2.700597000	3.085559000
H	0.909353000	-1.290320000	3.360235000
H	-2.854958000	-2.016759000	3.238766000
H	-3.175676000	-0.361606000	3.798572000
H	-1.204547000	-1.764783000	5.066092000
H	-0.838385000	-0.098909000	4.554895000
H	0.989812000	2.188072000	2.563970000
H	1.408900000	6.854720000	0.987159000
H	2.544761000	3.726509000	3.717655000
H	2.754207000	6.095284000	2.920286000
H	-2.267967000	3.928549000	0.595024000
H	-3.689858000	1.754666000	0.619509000
H	-2.511202000	1.030348000	-0.483704000
H	-5.200868000	4.092368000	-1.908588000
H	-5.102550000	3.756150000	-0.163576000
H	-3.775366000	4.618858000	-0.989977000
H	-3.561491000	2.942960000	-3.512761000
H	-2.162273000	3.340216000	-2.482340000
H	-2.496017000	1.641307000	-2.909066000
H	-5.511984000	1.650384000	-2.597542000
H	-6.941626000	0.397722000	1.124667000
H	-7.391397000	-1.533272000	2.607898000
H	-6.236003000	-3.712844000	2.229118000
H	-4.613377000	-3.939342000	0.342956000
H	-4.136891000	-2.014121000	-1.141903000
H	-0.558513000	4.748725000	-1.240275000
H	-1.351005000	5.917443000	-0.184515000
H	0.184930000	6.351283000	-0.950586000
H	-0.254007000	-5.949510000	1.059505000

TS(III-IV)b

SCF Energy = -3068.686619
Free Enthalpy (0K) = -3067.83958
Free Energy (298K) = -3067.937187
Free Energy (TZVP,PCM(THF),GD3BJ) = -3070.060263

C	3.504566000	4.389470000	0.333020000
C	3.769590000	3.958041000	-0.963675000
C	3.344526000	2.709059000	-1.439118000
C	2.619873000	1.864974000	-0.559806000
C	2.332625000	2.329175000	0.736995000
C	2.773772000	3.566228000	1.188929000
C	2.104182000	0.514108000	-0.913210000
C	2.853880000	-0.389849000	-1.627858000
C	2.356326000	-1.731061000	-2.063424000
C	3.468800000	-2.788745000	-2.163984000
C	4.365860000	-2.642356000	-0.949473000
C	4.323663000	-1.599032000	-0.146127000
C	4.876986000	-0.968397000	0.994281000
O	0.927120000	0.193534000	-0.402256000
Sm	-1.168547000	0.003256000	0.019611000
O	-1.022801000	2.216677000	-1.159454000
C	-0.456426000	2.364293000	-2.474683000
C	-0.416306000	3.862257000	-2.721504000
C	-0.198678000	4.409362000	-1.314587000
C	-1.062156000	3.482116000	-0.479318000
I	-1.863530000	-1.389035000	-2.633236000
I	-1.025317000	1.209220000	2.856684000
O	-3.089765000	-1.458580000	1.003001000
C	-4.213221000	-1.961709000	0.263806000
C	-5.171885000	-2.509255000	1.307768000
C	-4.213732000	-2.982353000	2.394816000
C	-3.156563000	-1.892385000	2.371179000
O	-0.270067000	-2.133221000	0.962606000
C	-0.571150000	-3.464711000	0.529405000
C	0.126144000	-4.363469000	1.532638000
C	1.391891000	-3.566488000	1.832221000
C	0.899519000	-2.126602000	1.810610000
O	-3.483911000	1.185627000	-0.179856000
C	-4.041688000	1.642966000	-1.422821000
C	-5.475401000	2.025760000	-1.101070000
C	-5.347886000	2.518063000	0.336169000
C	-4.350205000	1.529378000	0.912439000
C	2.859914000	-4.192291000	-2.218701000

H	-4.624683000	-1.144164000	-0.344435000
H	-3.861705000	-2.743120000	-0.430396000
H	-2.155443000	-2.225090000	2.677915000
H	-3.430843000	-1.031865000	3.003772000
H	-5.815861000	-3.305000000	0.906990000
H	-5.825887000	-1.711332000	1.696481000
H	-3.776676000	-3.957398000	2.123133000
H	-4.682953000	-3.088804000	3.383261000
H	-1.663605000	-3.576734000	0.498942000
H	-0.185917000	-3.610089000	-0.494354000
H	1.626753000	-1.418491000	1.389319000
H	0.589912000	-1.764422000	2.804303000
H	0.324359000	-5.367110000	1.129654000
H	-0.487921000	-4.478066000	2.441389000
H	2.143357000	-3.722831000	1.042283000
H	1.854856000	-3.831841000	2.793307000
H	-3.460322000	2.512447000	-1.771591000
H	-3.935565000	0.838144000	-2.165306000
H	-4.854127000	0.613959000	1.268506000
H	-3.735220000	1.922364000	1.734687000
H	-6.134407000	1.143218000	-1.149515000
H	-5.873699000	2.779087000	-1.795667000
H	-6.297760000	2.527497000	0.889653000
H	-4.935332000	3.539981000	0.358576000
H	0.551826000	1.921964000	-2.470725000
H	-1.080292000	1.800216000	-3.183397000
H	-2.112519000	3.824698000	-0.436993000
H	-0.700890000	3.341190000	0.549720000
H	-1.375882000	4.215726000	-3.134489000
H	0.378125000	4.143418000	-3.427365000
H	-0.488586000	5.463867000	-1.201616000
H	0.857744000	4.309110000	-1.020957000
C	3.653065000	2.360452000	-2.870400000
H	1.749000000	1.688392000	1.401073000
H	4.317535000	4.615704000	-1.645421000
H	2.544682000	3.883600000	2.209201000
H	3.856526000	5.368960000	0.666811000
H	3.773699000	-0.020252000	-2.080211000
C	4.321732000	-2.571002000	-3.423346000
H	1.842420000	-1.659261000	-3.040511000
H	1.587221000	-2.064993000	-1.350326000
H	5.159215000	-3.285872000	-3.461593000
H	3.715561000	-2.711824000	-4.333009000
H	4.751289000	-1.558222000	-3.450254000
H	3.640884000	-4.964163000	-2.312570000
H	2.278405000	-4.410344000	-1.309214000
H	2.183431000	-4.290151000	-3.082916000
C	4.609153000	-1.438919000	2.303635000
C	5.122160000	-0.783992000	3.416122000
C	5.908811000	0.360540000	3.265890000
C	6.180388000	0.841966000	1.981946000
C	5.674070000	0.195793000	0.863230000
H	4.001758000	-2.337874000	2.427136000
H	4.907862000	-1.171117000	4.415879000
H	6.304568000	0.876599000	4.143687000
H	6.790471000	1.739740000	1.853603000
H	5.878639000	0.586397000	-0.135844000
H	5.129848000	-3.426547000	-0.803741000
H	3.799591000	3.274983000	-3.464083000
H	4.579916000	1.769466000	-2.962190000
H	2.853652000	1.763951000	-3.333559000

IVb

SCF Energy = -3068.741161

Free Enthalpy (0K) = -3067.889689

Free Energy (298K) = -3067.983738

Free Energy (TZVP,PCM(THF),GD3BJ) = -3070.105772

C	-2.745552000	2.098314000	-1.505771000
C	-3.276636000	1.060268000	-0.499060000
C	-3.595019000	-0.118278000	-1.419258000
C	-3.660303000	0.296041000	-2.696372000
C	-3.412638000	1.777003000	-2.857728000
C	-2.378474000	0.790665000	0.686664000
C	-2.797101000	1.193168000	2.017122000
C	-3.617915000	2.343691000	2.169623000
C	-4.015964000	2.813709000	3.412520000
C	-3.618543000	2.142046000	4.569554000
C	-2.845985000	0.986974000	4.443699000
C	-2.432876000	0.488413000	3.206880000
H	-3.972729000	-0.330358000	-3.537138000
H	-1.660111000	1.941543000	-1.600279000
O	-1.187287000	0.261703000	0.467798000
Sm	0.921358000	-0.021307000	0.055022000
O	0.350012000	-2.355706000	0.671504000
C	-0.808761000	-3.003470000	0.099924000
C	-1.095308000	-4.183336000	1.011592000
C	0.290330000	-4.528103000	1.546189000
C	0.899364000	-3.150822000	1.733085000
I	0.417300000	-0.556064000	-2.929559000
I	2.088972000	0.364952000	2.894582000
O	3.137603000	1.176678000	-0.774386000
C	3.753721000	0.989551000	-2.056214000
C	4.725011000	2.145785000	-2.203740000
C	5.188001000	2.342653000	-0.764795000
C	3.910366000	2.091107000	0.021089000
O	0.704180000	2.475359000	-0.094404000
C	0.858122000	3.280882000	-1.272739000
C	0.176642000	4.602301000	-0.953467000
C	0.341537000	4.693842000	0.560143000
C	0.134726000	3.251608000	0.980185000
O	2.971545000	-1.675856000	-0.285304000
C	2.938329000	-2.832650000	-1.136446000
C	4.369573000	-3.339174000	-1.187939000
C	4.890581000	-2.943024000	0.188707000
C	4.238111000	-1.585538000	0.380785000
H	4.280230000	0.018312000	-2.065447000
H	2.963298000	0.946820000	-2.818073000
H	3.323672000	3.015038000	0.159466000
H	4.071521000	1.648915000	1.014671000
H	4.203453000	3.044767000	-2.571630000
H	5.541123000	1.922794000	-2.905897000
H	5.611765000	3.337349000	-0.565666000
H	5.956234000	1.596035000	-0.505146000
H	1.935445000	3.415140000	-1.465883000
H	0.420619000	2.748745000	-2.131048000
H	-0.932630000	2.997764000	1.075850000
H	0.646607000	2.963784000	1.909366000
H	-0.890554000	4.555264000	-1.218851000
H	0.624677000	5.444164000	-1.500616000
H	-0.377067000	5.373046000	1.040070000
H	1.356621000	5.035179000	0.822473000
H	2.261558000	-3.580662000	-0.689515000
H	2.524148000	-2.537171000	-2.111185000
H	4.837364000	-0.788484000	-0.093132000

H	4.058058000	-1.301723000	1.427877000
H	4.936230000	-2.818698000	-1.977416000
H	4.425815000	-4.418025000	-1.391930000
H	5.987097000	-2.895499000	0.253441000
H	4.540141000	-3.653896000	0.955070000
H	-1.621736000	-2.268765000	0.045345000
H	-0.553578000	-3.316342000	-0.925974000
H	1.994856000	-3.119718000	1.654904000
H	0.614129000	-2.705582000	2.700206000
H	-1.584886000	-5.007813000	0.475671000
H	-1.761503000	-3.879008000	1.833761000
H	0.859805000	-5.110355000	0.802610000
H	0.271525000	-5.104373000	2.482346000
H	-3.912866000	2.906345000	1.280515000
C	-1.709799000	-0.823568000	3.159337000
H	-4.630702000	3.715472000	3.478864000
H	-2.569003000	0.428938000	5.343395000
H	-3.919837000	2.500381000	5.556616000
H	-4.242723000	1.405179000	-0.090710000
C	-3.948853000	-1.460620000	-0.938224000
C	-3.824923000	-2.587110000	-1.771385000
C	-4.205751000	-3.850904000	-1.331407000
C	-4.712763000	-4.025946000	-0.041639000
C	-4.830604000	-2.921789000	0.800695000
C	-4.450566000	-1.654210000	0.360606000
H	-3.402985000	-2.465507000	-2.771699000
H	-4.101586000	-4.709923000	-1.999763000
H	-5.012863000	-5.018573000	0.303715000
H	-5.227524000	-3.042850000	1.812030000
H	-4.556451000	-0.800112000	1.032931000
H	-2.921495000	3.136223000	-1.177859000
C	-4.763327000	2.498146000	-2.996464000
C	-2.526176000	2.120238000	-4.053624000
H	-3.020069000	1.852431000	-5.002077000
H	-2.317989000	3.202959000	-4.085526000
H	-1.568548000	1.580549000	-4.005673000
H	-5.302912000	2.155011000	-3.893582000
H	-5.411999000	2.310772000	-2.126684000
H	-4.617370000	3.587157000	-3.089053000
H	-1.808602000	-1.349070000	4.120808000
H	-2.118247000	-1.465671000	2.364389000
H	-0.635969000	-0.692513000	2.959069000

3b

SCF Energy = -3068.775498
Free Enthalpy (0K) = -3067.92397
Free Energy (298K) = -3068.022895
Free Energy (TZVP,PCM(THF),GD3BJ) = -3070.143471

C	3.025916000	-1.523513000	-1.983871000
C	3.555242000	-0.771091000	-0.744165000
C	3.704217000	0.647959000	-1.272989000
C	3.691658000	0.640132000	-2.616266000
C	3.529660000	-0.734397000	-3.211966000
C	2.612717000	-0.923969000	0.443368000
C	3.039028000	-1.782752000	1.580781000
C	3.851991000	-2.898883000	1.308761000
C	4.231675000	-3.780545000	2.311593000
C	3.828832000	-3.529541000	3.622368000
C	3.051391000	-2.411802000	3.907947000
C	2.626718000	-1.524024000	2.911043000
H	3.865955000	1.519907000	-3.241000000
H	1.926818000	-1.481045000	-1.957142000
O	1.517632000	-0.373875000	0.433158000
Sm	-1.022063000	0.026081000	-0.025850000
O	-0.313874000	2.020508000	1.513188000
C	0.687319000	2.980601000	1.150634000
C	0.919631000	3.812484000	2.402231000
C	-0.445683000	3.758349000	3.079477000
C	-0.874108000	2.328847000	2.790713000
I	-0.491262000	1.662549000	-2.736913000
I	-2.043716000	-1.515353000	2.663233000
O	-3.168853000	-1.016192000	-1.219546000
C	-3.890243000	-0.353277000	-2.265020000
C	-5.000726000	-1.312620000	-2.664922000
C	-5.275923000	-2.039829000	-1.352834000
C	-3.874894000	-2.181517000	-0.783174000
O	-0.385683000	-2.316002000	-1.064096000
C	-0.624892000	-2.692425000	-2.424381000
C	-0.182183000	-4.144621000	-2.530325000
C	-0.436441000	-4.657558000	-1.116025000
C	-0.028726000	-3.459105000	-0.277890000
O	-3.131236000	1.629936000	0.348211000
C	-3.160016000	3.011376000	-0.026703000
C	-4.583167000	3.476274000	0.241429000
C	-4.986376000	2.588515000	1.414358000
C	-4.332676000	1.270853000	1.034854000
H	-4.293284000	0.594396000	-1.867762000
H	-3.190585000	-0.100698000	-3.075478000
H	-3.374564000	-3.081027000	-1.188535000
H	-3.825896000	-2.231375000	0.315141000
H	-4.643010000	-2.022973000	-3.428490000
H	-5.877232000	-0.792964000	-3.078107000
H	-5.781694000	-3.008024000	-1.478346000
H	-5.900065000	-1.418648000	-0.689552000
H	-1.700467000	-2.573987000	-2.636346000
H	-0.075459000	-2.008143000	-3.088567000
H	1.061410000	-3.449863000	-0.097703000
H	-0.541126000	-3.383521000	0.693883000
H	0.891856000	-4.203890000	-2.771049000
H	-0.730481000	-4.695343000	-3.308177000
H	0.133382000	-5.563401000	-0.864108000
H	-1.505912000	-4.881118000	-0.970356000
H	-2.432442000	3.561799000	0.595373000
H	-2.842628000	3.101990000	-1.076521000
H	-4.981088000	0.691080000	0.352398000
H	-4.066629000	0.622916000	1.883665000
H	-5.226424000	3.273725000	-0.630715000
H	-4.642071000	4.552926000	0.456795000
H	-6.073606000	2.498126000	1.551285000
H	-4.560387000	2.974170000	2.355442000
H	1.582423000	2.447081000	0.802341000
H	0.308104000	3.586785000	0.309822000
H	-1.962066000	2.179725000	2.738114000
H	-0.480152000	1.623797000	3.542608000
H	1.262285000	4.830735000	2.170654000
H	1.684315000	3.340745000	3.040021000
H	-1.137814000	4.475108000	2.607036000
H	-0.415355000	3.980118000	4.156036000
H	4.169660000	-3.095366000	0.282519000
C	1.781310000	-0.351361000	3.299927000
H	4.843910000	-4.653215000	2.073836000
H	2.751868000	-2.214905000	4.940619000
H	4.127476000	-4.204014000	4.428762000
H	4.551809000	-1.143441000	-0.453361000

C	3.968921000	1.807708000	-0.411382000
C	3.746326000	3.117612000	-0.872781000
C	4.036594000	4.216304000	-0.071453000
C	4.545388000	4.038080000	1.217127000
C	4.755864000	2.746524000	1.695586000
C	4.468897000	1.644341000	0.891142000
H	3.311227000	3.269654000	-1.862893000
H	3.853086000	5.224249000	-0.452409000
H	4.770760000	4.902917000	1.845920000
H	5.151691000	2.591337000	2.702478000
H	4.653953000	0.640405000	1.282697000
H	3.327612000	-2.583363000	-2.005019000
C	4.898281000	-1.250372000	-3.683128000
C	2.533216000	-0.768217000	-4.370011000
H	2.918972000	-0.207568000	-5.236460000
H	2.356280000	-1.805261000	-4.701012000
H	1.572870000	-0.314845000	-4.078738000
H	5.318204000	-0.595162000	-4.462360000
H	5.623967000	-1.289550000	-2.855437000
H	4.806874000	-2.262290000	-4.110740000
H	1.904085000	-0.135045000	4.370736000
H	2.024668000	0.549778000	2.721495000
H	0.714374000	-0.574070000	3.122827000

1c

SCF Energy = -2800.113797

Free Enthalpy (0K) = -2799.351396

Free Energy (298K) = -2799.44527

Free Energy (TZVP,PCM(THF),GD3BJ) = -2801.265121

C	3.326535	-2.604449	0.099897
O	2.289368	-2.035313	-0.705643
C	2.256940	-2.657311	-1.990906
C	3.583439	-3.386084	-2.116963
C	3.827269	-3.814659	-0.673730
Sm	0.655793	-0.023952	-0.011919
O	0.834865	2.414622	1.019520
C	1.397918	3.496459	0.271315
C	1.305855	4.712185	1.180239
C	1.434510	4.079825	2.563407
C	0.656293	2.785533	2.392403
O	-1.836086	0.744723	0.079325
C	-3.055110	0.745372	-0.069508
C	-3.767325	1.819796	-0.784735
C	-3.436350	3.314155	-0.591743
C	-2.371506	3.740061	0.385647
C	-3.878280	-0.368713	0.505787
C	-4.725546	-1.135398	-0.324456
C	-5.422249	-2.208658	0.241283
C	-5.311915	-2.500324	1.596638
C	-4.495502	-1.720766	2.408558
C	-3.759067	-0.653089	1.884817
C	-4.908910	-0.860862	-1.795405
C	-3.050699	2.629684	-1.849181
C	-4.642128	4.226543	-0.620321
O	-0.773270	-2.102048	-0.770851
C	-1.429531	-2.327757	-2.020483
C	-2.236968	-3.604819	-1.835023
C	-1.412548	-4.355026	-0.793640
C	-0.954014	-3.218944	0.104549
I	0.664489	1.005669	-3.065406
I	1.013909	-1.193405	2.993182
O	3.223170	0.752991	0.088660
C	4.088166	0.829086	-1.048314
C	5.350243	1.512292	-0.548862
C	5.412631	1.025819	0.895447
C	3.944363	1.042456	1.289513
H	4.299425	-0.194705	-1.404941
H	3.562495	1.360680	-1.855626
H	3.642543	2.041553	1.651941
H	3.667863	0.303920	2.057635
H	5.235568	2.608471	-0.582244
H	6.236919	1.250991	-1.144294
H	6.032950	1.654328	1.550638
H	5.812991	-0.000476	0.939165
H	2.445082	3.246318	0.030729
H	0.850063	3.591627	-0.678591
H	-0.421118	2.935560	2.582837
H	1.004524	1.957046	3.027716
H	0.325043	5.202029	1.072472
H	2.080999	5.460625	0.961493
H	1.036889	4.706228	3.374808
H	2.491442	3.867382	2.793525
H	1.413726	-3.370278	-2.027703
H	2.086000	-1.879198	-2.751198
H	4.125814	-1.853634	0.228371
H	2.919774	-2.834805	1.096324
H	4.373099	-2.695163	-2.455570
H	3.541478	-4.222884	-2.829030
H	4.877644	-4.051868	-0.451338
H	3.224618	-4.705427	-0.431613
H	-2.038364	-1.447509	-2.271522
H	-0.668495	-2.430776	-2.812596
H	-0.006644	-3.401458	0.632275
H	-1.718602	-2.969357	0.861160
H	-2.370967	-4.157303	-2.776302
H	-3.234008	-3.374922	-1.426998
H	-0.548431	-4.851786	-1.265473
H	-1.983357	-5.119517	-0.247399
C	-2.889469	0.150628	2.808060
H	-6.063936	-2.821127	-0.398410
H	-4.417808	-1.940365	3.476581
H	-5.869521	-3.337733	2.023460
H	-4.832025	1.630628	-0.929887
H	-3.613496	2.840549	-2.762770
H	-1.997396	2.367112	-2.001462
H	-1.998764	4.738225	0.103216
H	-2.782686	3.816100	1.405785
H	-1.523402	3.044746	0.400168
H	-5.124652	4.279789	0.368738
H	-4.336997	5.248014	-0.900101
H	-5.393974	3.890997	-1.350147
H	-5.130990	-1.793388	-2.334214
H	-5.760139	-0.182299	-1.971606
H	-4.030203	-0.399626	-2.265979
H	-3.252425	0.065639	3.841576
H	-1.846754	-0.212116	2.798481
H	-2.862770	1.216156	2.540937

Ic

SCF Energy = -2800.074776

Free Enthalpy (0K) = -2799.31311

Free Energy (298K) = -2799.403307

Free Energy (TZVP,PCM(THF),GD3BJ) = -2801.222787

C	3.839022	1.311802	1.282438

O 3.125505 0.980787 0.079690
C 3.934574 1.261426 -1.073384
C 5.093593 2.096557 -0.558742
C 5.276292 1.540937 0.848840
Sm 0.674045 0.003862 0.029575
I 1.388787 -1.182049 2.792631
O 2.439457 -1.707188 -0.870175
C 3.577896 -2.218178 -0.157110
C 4.115212 -3.357412 -1.007919
C 3.728856 -2.911917 -2.413496
C 2.358055 -2.310470 -2.171019
O 0.803374 2.298400 1.060511
C 1.110442 3.510994 0.356468
C 0.466030 4.625300 1.165095
C 0.499227 4.057494 2.580886
C 0.210135 2.586884 2.339604
O -1.412750 0.439116 0.322064
C -2.691932 0.473462 -0.024597
C -3.620416 -0.570799 0.384890
C -4.679444 -1.008641 -0.474272
C -5.521216 -2.045497 -0.066253
C -5.366179 -2.684204 1.160893
C -4.362176 -2.241421 2.019958
C -3.507563 -1.195837 1.672202
C -4.885464 -0.462256 -1.860393
C -2.576799 -0.680069 2.728125
O -0.358614 -2.146494 -0.726207
C -1.342314 -2.265058 -1.770178
C -2.125726 -3.520978 -1.436819
C -1.067450 -4.385715 -0.760975
C -0.295450 -3.358353 0.047748
I 0.547259 1.095847 -2.847036
C -3.106537 1.656784 -0.838976
C -2.632473 3.021573 -0.438644
C -4.098121 2.703825 -0.348384
C -5.018474 3.333982 -1.367271
C -4.709822 2.550751 1.023026
H 4.284213 0.307011 -1.504303
H 3.308031 1.758926 -1.826726
H 3.395390 2.223746 1.716949
H 3.703339 0.492682 2.003275
H 4.815535 3.162541 -0.519873
H 5.987986 2.007440 -1.191883
H 5.815801 2.216584 1.527960
H 5.828228 0.587176 0.818365
H 2.208001 3.624611 0.316031
H 0.733581 3.420833 -0.673447
H -0.870023 2.375044 2.279909
H 0.654298 1.911520 3.085194
H -0.573281 4.785067 0.840149
H 1.001588 5.579663 1.059578
H -0.232644 4.521257 3.257455
H 1.498727 4.185911 3.027973
H 1.577068 -3.090054 -2.156067
H 2.061545 -1.534094 -2.891557
H 4.319228 -1.408088 -0.052868
H 3.257339 -2.515208 0.851964
H 4.428184 -2.144813 -2.784967
H 3.705235 -3.729465 -3.148164
H 5.195147 -3.509813 -0.868890
H 3.608962 -4.302954 -0.753896
H -1.947112 -1.348603 -1.782854
H -0.813224 -2.352018 -2.735445
H 0.764297 -3.608160 0.204841
H -0.755942 -3.177510 1.032148
H -2.568593 -3.984141 -2.330079
H -2.939095 -3.286743 -0.733766
H -0.413771 -4.864399 -1.509372
H -1.490873 -5.176778 -0.126111
H -6.305305 -2.379485 -0.752577
H -4.255722 -2.693683 3.010500
H -6.028442 -3.503805 1.449165
H -3.131136 1.505125 -1.924895
H -2.279783 3.695220 -1.224953
H -2.073382 3.087854 0.496651
H -5.008364 3.532046 1.428630
H -5.606944 1.911428 0.989482
H -4.007092 2.088557 1.730940
H -5.953680 2.759093 -1.473473
H -5.295833 4.358636 -1.067349
H -4.543931 3.393574 -2.358870
H -5.648721 -1.050147 -2.389719
H -5.216735 0.587056 -1.855618
H -3.962351 -0.498005 -2.461338
H -2.747112 -1.202927 3.676858
H -2.749784 0.393069 2.898314
H -1.520509 -0.788319 2.469050

TS(I-II)c
SCF Energy = -2800.050499
Free Enthalpy (0K) = -2799.290583
Free Energy (298K) = -2799.379368
Free Energy (TZVP,PCM(THF),GD3BJ) = -2801.200393
C -3.150762 -2.536060 -0.794287
O -2.200604 -2.095753 0.185000
C -2.207242 -2.972636 1.316506
C -3.538830 -3.695839 1.243697
C -3.717044 -3.847334 -0.263860
Sm -0.553807 -0.017627 -0.024355
O -0.994075 2.469362 -0.338345
C -1.414736 3.339846 0.720611
C -1.051103 4.731541 0.239991
C -1.325600 4.626834 -1.257519
C -0.914322 3.195123 -1.581329
O 1.508347 0.539572 -0.130611
C 2.791779 0.649319 0.224887
C 3.263842 1.756088 0.926879
C 2.450513 2.949967 1.260605
C 3.275907 3.578864 0.193891
C 2.765422 3.683554 -1.203171
C 3.731331 -0.426783 -0.195530
C 3.825256 -0.782304 -1.566231
C 4.693967 -1.805411 -1.955236
C 5.471429 -2.486995 -1.022389
C 5.389694 -2.132176 0.319072
C 4.538779 -1.107140 0.752494
C 3.049109 -0.047360 -2.618955
C 4.476835 4.377849 0.578281
O 0.570779 -2.240520 0.361783
C 1.407055 -2.612490 1.472293
C 1.924677 -3.997680 1.129573
C 2.006642 -3.938279 -0.392192
C 0.759335 -3.144618 -0.737697
I -0.604123 0.289043 3.042036

I -1.033066 -0.335695 -3.064087
O -3.150163 0.634107 0.153058
C -3.986772 0.356406 1.288019
C -5.330273 0.999306 0.986023
C -5.368054 0.965097 -0.536771
C -3.918871 1.252870 -0.884826
H -4.075272 -0.735049 1.392644
H -3.497483 0.744757 2.192105
H -3.733781 2.341403 -0.879396
H -3.584111 0.843419 -1.849481
H -5.353448 2.040293 1.347082
H -6.164514 0.464962 1.462641
H -6.061612 1.695661 -0.977160
H -5.660017 -0.036019 -0.895123
H -2.504357 3.235582 0.864203
H -0.913660 3.018491 1.644459
H 0.121300 3.132702 -1.944004
H -1.563208 2.706155 -2.322007
H 0.015818 4.932146 0.428063
H -1.639130 5.514737 0.739428
H -0.764491 5.359714 -1.854660
H -2.397297 4.780890 -1.462997
H -1.366356 -3.681390 1.230163
H -2.056774 -2.366940 2.223017
H -3.930574 -1.763290 -0.898548
H -2.643486 -2.623639 -1.765723
H -4.342238 -3.074423 1.672362
H -3.530138 -4.651888 1.786420
H -4.760207 -4.007322 -0.571908
H -3.126861 -4.701538 -0.632866
H 2.225569 -1.884225 1.546087
H 0.805838 -2.563962 2.391434
H -0.129131 -3.799444 -0.817533
H 0.839351 -2.563753 -1.667229
H 1.215622 -4.777343 1.455927
H 2.894568 -4.200418 1.605510
H 2.015800 -4.926578 -0.874035
H 2.912272 -3.394629 -0.702724
C 4.512685 -0.786397 2.223560
H 4.770779 -2.057681 -3.016931
H 5.999356 -2.660160 1.058489
H 6.144873 -3.286285 -1.342304
H 4.281529 1.710194 1.316269
H 2.568135 3.354286 2.276183
H 1.395434 2.806864 1.005665
H 3.590189 3.696928 -1.934691
H 2.117086 2.830101 -1.448097
H 2.177729 4.611471 -1.368043
H 5.263295 4.342882 -0.194004
H 4.221160 5.447877 0.725929
H 4.917308 4.027907 1.525790
H 3.475530 -0.229628 -3.615836
H 1.990931 -0.347994 -2.642184
H 3.054882 1.035765 -2.430176
H 4.802156 -1.666934 2.816605
H 5.226212 0.015937 2.476017
H 3.524300 -0.438245 2.558445

IIc

SCF Energy = -2800.071749
Free Enthalpy (0K) = -2799.311676
Free Energy (298K) = -2799.401594
Free Energy (TZVP,PCM(THF),GD3BJ) = -2801.22189
C 3.658221 -2.018007 0.333167
O 2.597470 -1.633066 -0.555856
C 2.791967 -2.224016 -1.846449
C 4.258442 -2.608270 -1.883080
C 4.492439 -3.031614 -0.436845
Sm 0.576840 -0.066670 0.006457
O 0.366654 2.280378 0.934581
C 0.758558 3.499777 0.289555
C -0.026649 4.602717 0.987886
C -0.283546 4.004741 2.368479
C -0.527519 2.547484 2.026437
O -1.542298 0.209678 0.072420
C -2.861178 0.271083 -0.064037
C -3.519741 1.365878 -0.515990
C -2.873106 2.622045 -1.009962
C -3.517988 3.905111 -0.581133
C -4.039498 4.044664 0.807759
C -3.627186 -0.967727 0.290170
C -4.318538 -1.683177 -0.708024
C -4.992204 -2.861179 -0.351186
C -5.003458 -3.316108 0.962946
C -4.347483 -2.584813 1.949215
C -3.658190 -1.410685 1.633232
C -4.379850 -1.219248 -2.140577
C -3.351510 5.094676 -1.464545
O -0.175606 -2.297385 -0.868258
C -0.937849 -2.485176 -2.073097
C -1.557966 -3.868653 -1.950173
C -0.572362 -4.595864 -1.042505
C -0.178835 -3.494565 -0.076343
I 0.752046 0.968793 -2.889045
I 0.953987 -1.181235 2.876547
O 2.897315 1.118486 0.364005
C 3.801600 1.502185 -0.683090
C 4.773337 2.475287 -0.039265
C 4.851173 1.940420 1.385962
C 3.413040 1.522653 1.642666
H 4.320947 0.600737 -1.054129
H 3.216327 1.919770 -1.514713
H 2.805632 2.363888 2.017585
H 3.300679 0.684873 2.346059
H 4.358772 3.496804 -0.045872
H 5.742810 2.505561 -0.556817
H 5.210054 2.677802 2.118216
H 5.523760 1.068190 1.433954
H 1.848040 3.619135 0.415531
H 0.547798 3.415775 -0.787271
H -1.563928 2.372483 1.690625
H -0.297132 1.836911 2.833601
H -0.980652 4.780782 0.467229
H 0.524076 5.553891 1.014582
H -1.136911 4.462113 2.889206
H 0.603501 4.109473 3.014932
H 2.145814 -3.114288 -1.940995
H 2.490013 -1.486502 -2.605504
H 4.241655 -1.117410 0.585530
H 3.220657 -2.406696 1.264018
H 4.878677 -1.733857 -2.139601
H 4.470495 -3.399075 -2.616819
H 5.549680 -3.016896 -0.135528
H 4.112913 -4.052856 -0.271807

H -1.678086 -1.677514 -2.147569
H -0.250151 -2.401708 -2.930707
H 0.819556 -3.609665 0.369880
H -0.911891 -3.386369 0.740661
H -1.693362 -4.351175 -2.928677
H -2.542179 -3.801248 -1.461687
H 0.303846 -4.947632 -1.612534
H -1.010399 -5.462700 -0.527598
C -3.002506 -0.610384 2.721215
H -5.521402 -3.423877 -1.125493
H -4.379214 -2.919559 2.989959
H -5.537611 -4.233650 1.224129
H -4.610739 1.299774 -0.581080
H -2.829007 2.596747 -2.117278
H -1.807306 2.600445 -0.705994
H -3.863268 5.984716 -1.068294
H -2.285134 5.367835 -1.610434
H -3.742926 4.904402 -2.481388
H -4.570056 4.997781 0.953823
H -4.728111 3.224327 1.074853
H -3.231618 4.002676 1.570074
H -4.482119 -2.073883 -2.824932
H -5.255539 -0.567751 -2.305021
H -3.502023 -0.627463 -2.433890
H -3.258086 -1.004719 3.714125
H -1.907358 -0.619837 2.624080
H -3.321278 0.442238 2.671593

TS(II-III)c

SCF Energy = -3107.867984
Free Enthalpy (0K) = -3106.996163
Free Energy (298K) = -3107.095635
Free Energy (TZVP,PCM(THF),GD3BJ) = -3109.264875

C -3.917396 -0.836598 -1.112924
O -2.586786 -0.393674 -1.411453
C -2.626309 0.702185 -2.346996
C -4.100364 0.933379 -2.655003
C -4.791896 0.360793 -1.421751
Sm -0.535092 -0.747990 0.007150
I -2.307501 -1.978048 2.245759
O -0.904096 1.336359 0.375112
C -1.095921 2.636815 0.547489
C -2.533356 3.077801 0.621357
C -3.104705 3.831141 -0.427146
C -4.464619 4.165444 -0.362269
C -5.246904 3.785500 0.721915
C -4.668189 3.078512 1.772252
C -3.316881 2.721442 1.741643
C -0.107347 3.558686 0.649417
C 1.373600 3.331106 0.490797
C 1.989007 4.371658 -0.408802
C 2.078119 5.762586 0.134818
C 4.337157 3.951996 -0.471079
C 4.812395 2.826187 -0.324936
C 5.325062 1.512228 -0.168660
C 6.097778 1.169265 0.961921
C 6.624052 -0.110433 1.097175
C 6.396318 -1.076322 0.113852
C 5.634228 -0.747969 -1.009579
C 5.102499 0.528531 -1.155532
C 1.758287 4.236482 -1.878692
O 1.072274 -2.766453 0.466242
C 2.019616 -3.300044 -0.473672
C 2.917816 -4.222107 0.333761
C 1.970961 -4.716852 1.421262
C 1.156103 -3.470179 1.715848
O -1.168884 -2.870891 -1.391976
C -1.462333 -4.169566 -0.858012
C -2.166935 -4.914973 -1.977776
C -1.498922 -4.318527 -3.211572
C -1.371489 -2.859003 -2.813830
O 1.149555 -0.228857 1.787892
C 2.581183 -0.228860 1.642663
C 3.112751 0.612078 2.795540
C 2.002951 0.495849 3.835183
C 0.765019 0.506624 2.961711
I 1.274210 -0.048808 -2.381977
H 1.466961 -3.846621 -1.257476
H 2.550742 -2.468117 -0.957945
H 1.656769 -2.822975 2.456334
H 0.135122 -3.661479 2.074998
H 3.752544 -3.653931 0.775974
H 3.349182 -5.026838 -0.278791
H 2.483977 -5.101681 2.314214
H 1.323978 -5.521506 1.034671
H 2.932473 -1.271605 1.699970
H 2.845690 0.168964 0.652069
H 0.477667 1.528436 2.664301
H -0.108840 0.005186 3.401273
H 3.235353 1.658655 2.477199
H 4.090244 0.254808 3.146902
H 2.004126 1.313178 4.570385
H 2.077369 -0.456274 4.386174
H -2.295742 -2.299349 -3.033552
H -0.527353 -2.327856 -3.277394
H -0.514522 -4.665419 -0.585427
H -2.061065 -4.044971 0.055953
H -0.503857 -4.766946 -3.367166
H -2.053815 -6.005325 -1.892856
H -3.245283 -4.686810 -1.974993
H -2.161116 1.564880 -1.853677
H -2.020535 0.436907 -3.226664
H -4.165770 -1.699540 -1.760361
H -3.942569 -1.165422 -0.064653
H -4.403588 0.381464 -3.559792
H -4.321624 1.997508 -2.817927
H -5.841859 0.084216 -1.594849
H -4.755347 1.081961 -0.589591
C -2.298874 4.321468 -1.600465
C -2.707933 2.006011 2.910460
H -4.909766 4.740665 -1.179437
H -5.269835 2.803620 2.643143
H -6.306033 4.053718 0.756993
H -0.434643 4.587819 0.827211
H 1.545356 2.322873 0.080547
H 1.878415 3.379713 1.475609
H 2.454747 4.861596 -2.461284
H 1.858761 3.192433 -2.211223
H 0.734910 4.567144 -2.146081
H 2.735278 6.403852 -0.476995
H 1.086737 6.257934 0.134488
H 2.447106 5.777629 1.172833
H 4.388927 5.004572 -0.701029
H 4.494907 0.775861 -2.027745

H 5.450850 -1.494442 -1.786745
H 6.819889 -2.078309 0.218960
H 7.227199 -0.355617 1.975346
H 6.284483 1.927341 1.725591
H -2.075807 -4.451912 -4.137903
H -3.396997 1.989222 3.766390
H -2.457166 0.964033 2.660993
H -1.775379 2.500592 3.223360
H -2.950800 4.538668 -2.459810
H -1.767703 5.254636 -1.350295
H -1.526251 3.605653 -1.912707

IIIc
SCF Energy = -3107.929363
Free Enthalpy (0K) = -3107.054041
Free Energy (298K) = -3107.153705
Free Energy (TZVP,PCM(THF),GD3BJ) = -3109.318201
C 3.931556 0.014174 1.055931
O 2.555655 0.076810 1.466757
C 2.368551 1.105560 2.454648
C 3.768220 1.566792 2.825322
C 4.537895 1.320748 1.531881
Sm 0.662078 -0.698211 0.021691
I 2.513567 -1.510557 -2.345784
O 0.650458 1.439536 -0.296458
C 0.597298 2.752749 -0.501573
C 1.912497 3.459504 -0.753824
C 2.456512 4.354671 0.201667
C 3.729488 4.898936 -0.017386
C 4.458712 4.598090 -1.160557
C 3.899211 3.769159 -2.124642
C 2.634234 3.202528 -1.941505
C -0.560791 3.463155 -0.523898
C -1.928447 2.953439 -0.198248
C -2.769415 3.859402 0.731165
C -3.022570 5.231024 0.089062
C -4.127187 3.222591 0.986048
C -4.629739 2.136240 0.464834
C -5.335325 0.985862 0.159845
C -6.456195 1.007692 -0.725657
C -7.130500 -0.159856 -1.041499
C -6.728781 -1.388062 -0.499405
C -5.635495 -1.430128 0.376767
C -4.945491 -0.275540 0.707008
C -2.089493 4.043113 2.092505
O -0.473149 -3.008191 -0.468119
C -1.150011 -3.810377 0.511723
C -1.975820 -4.803285 -0.285243
C -1.087525 -5.033867 -1.501787
C -0.528937 -3.643621 -1.757088
O 1.824898 -2.687198 1.282836
C 2.452671 -3.819811 0.664280
C 3.410928 -4.360556 1.708036
C 2.643313 -4.086869 2.995647
C 2.019027 -2.731232 2.707824
O -1.246404 -0.472932 -1.589466
C -2.628258 -0.785096 -1.342676
C -3.410752 -0.016347 -2.393477
C -2.416335 0.055965 -3.546847
C -1.109034 0.278022 -2.810059
I -1.207604 -0.573275 2.481439
H -0.398192 -4.323559 1.136786
H -1.733768 -3.144393 1.162764
H -1.188394 -3.049728 -2.411784
H 0.478206 -3.634700 -2.197340
H -2.936446 -4.353148 -0.584954
H -2.197343 -5.717733 0.283622
H -1.623176 -5.435385 -2.373878
H -0.275594 -5.737710 -1.255624
H -2.765415 -1.874803 -1.447675
H -2.873123 -0.503515 -0.309480
H -0.954874 1.338388 -2.552683
H -0.220006 -0.092136 -3.341998
H -3.644657 0.993733 -2.024511
H -4.362207 -0.505365 -2.645836
H -2.633090 0.861552 -4.262755
H -2.389489 -0.894289 -4.105640
H 2.690063 -1.903705 2.989010
H 1.049408 -2.567792 3.200045
H 1.679938 -4.568045 0.411050
H 2.930035 -3.487400 -0.268380
H 1.863440 -4.851180 3.146638
H 3.651229 -5.421761 1.550269
H 4.356526 -3.794131 1.691387
H 1.776288 1.908927 1.991257
H 1.788185 0.685420 3.288976
H 4.404952 -0.860645 1.538895
H 3.967954 -0.128669 -0.033659
H 4.177209 0.951388 3.643752
H 3.784232 2.616221 3.152318
H 5.626194 1.251865 1.671601
H 4.332735 2.122139 0.804171
C 1.708566 4.816859 1.422091
C 2.043797 2.367571 -3.031220
H 4.146791 5.580270 0.729468
H 4.448606 3.552480 -3.045075
H 5.455362 5.022433 -1.307681
H -0.465076 4.516180 -0.798482
H -1.837840 1.956764 0.261334
H -2.522620 2.821081 -1.124184
H -2.709740 4.659335 2.763382
H -1.910554 3.072579 2.578549
H -1.120261 4.545916 1.969312
H -3.703134 5.834380 0.710765
H -2.087562 5.801049 -0.020965
H -3.481201 5.125157 -0.906753
H -4.761084 3.790644 1.691939
H -4.094719 -0.307716 1.392382
H -5.325223 -2.383292 0.813541
H -7.268892 -2.303528 -0.751461
H -7.988845 -0.118865 -1.717386
H -6.773030 1.963817 -1.147829
H 3.274395 -4.072362 3.895835
H 2.682498 2.374709 -3.925125
H 1.924480 1.325191 -2.709386
H 1.048011 2.747229 -3.312210
H 2.390964 5.313099 2.128184
H 0.923206 5.540658 1.160264
H 1.199828 3.995710 1.948172

TS(III-IV)c
SCF Energy = -3107.921064
Free Enthalpy (0K) = -3107.045971
Free Energy (298K) = -3107.143901

Free Energy (TZVP,PCM(THF),GD3BJ) = -3109.311944

C	4.929652	-1.819393	2.008611
C	5.052801	-1.121730	0.782301
C	5.823075	0.066728	0.774855
C	6.442051	0.520657	1.931365
C	6.311742	-0.182775	3.132565
C	5.553479	-1.355479	3.160488
C	4.384608	-1.562787	-0.389595
C	4.390187	-2.482411	-1.334732
C	3.408138	-2.478220	-2.488762
C	4.157660	-2.055581	-3.762136
C	2.295258	-1.475649	-2.149955
C	2.810482	-0.188757	-1.584031
C	2.054360	0.695293	-0.847232
O	0.856864	0.404648	-0.367455
Sm	-1.219269	-0.018371	0.027856
O	-3.703157	0.820840	-0.100244
C	-4.280978	1.441704	-1.260444
C	-5.758571	1.589375	-0.943602
C	-5.730488	1.805325	0.564719
C	-4.635946	0.843523	0.990835
C	2.606972	2.056584	-0.544533
C	2.920094	2.965723	-1.580396
C	3.495330	4.203629	-1.253652
C	3.738372	4.558465	0.064970
C	3.415639	3.665609	1.085049
C	2.859956	2.416299	0.804010
C	2.819886	-3.873982	-2.708934
O	-1.363394	2.399408	-0.589669
C	-0.778166	2.894368	-1.807440
C	-0.422559	4.338778	-1.511507
C	-1.537391	4.743302	-0.553516
C	-1.698115	3.488809	0.287574
I	-1.666934	-0.929116	-2.864411
I	-1.337099	0.842197	3.006369
O	-2.950758	-1.866484	0.722043
C	-3.897790	-2.475604	-0.171051
C	-4.491342	-3.641468	0.601428
C	-4.430288	-3.134439	2.037365
C	-3.104366	-2.395181	2.049535
O	-0.096138	-2.125690	0.787726
C	-0.236202	-3.449509	0.251122
C	0.838240	-4.291766	0.934484
C	1.149781	-3.490603	2.195244
C	1.027918	-2.070613	1.684121
H	-4.662710	-1.725701	-0.436433
H	-3.378185	-2.761555	-1.096431
H	-2.262791	-3.077759	2.260796
H	-3.048287	-1.562916	2.765092
H	-3.864914	-4.541009	0.484987
H	-5.505515	-3.896030	0.261869
H	-4.471208	-3.932678	2.792190
H	-5.263272	-2.441124	2.239313
H	-1.252795	-3.803217	0.482898
H	-0.130452	-3.409588	-0.844637
H	1.922463	-1.752732	1.122524
H	0.801244	-1.322237	2.456254
H	1.737455	-4.353653	0.302657
H	0.497563	-5.317744	1.134631
H	2.143361	-3.704950	2.613132
H	0.402533	-3.679315	2.983688
H	-3.801184	2.423573	-1.411018
H	-4.062712	0.813564	-2.136242
H	-5.036767	-0.174392	1.138151
H	-4.098669	1.136794	1.904430
H	-6.301966	0.662437	-1.190134
H	-6.227388	2.411652	-1.502798
H	-6.688537	1.598582	1.062876
H	-5.447022	2.844169	0.800865
H	0.079644	2.257683	-2.057353
H	-1.524456	2.809584	-2.616803
H	-2.718462	3.330165	0.667334
H	-1.009054	3.473026	1.146731
H	-0.380793	4.955028	-2.421085
H	0.558192	4.394107	-1.012829
H	-2.464078	4.964355	-1.108930
H	-1.293593	5.622699	0.059237
C	2.617076	2.719889	-3.037204
C	2.585986	1.465204	1.930719
H	3.744447	4.899972	-2.060395
H	3.621996	3.929207	2.126117
H	4.188030	5.526291	0.302232
H	3.713035	0.237897	-2.030370
H	1.693236	-1.285553	-3.057839
H	1.596481	-1.923564	-1.426846
H	5.001762	-2.733703	-3.965853
H	3.485146	-2.081346	-4.634913
H	4.565283	-1.037723	-3.672584
H	3.603801	-4.601914	-2.973539
H	2.312134	-4.241068	-1.803868
H	2.082192	-3.860025	-3.527297
H	5.915701	0.630614	-0.155730
H	7.031247	1.440826	1.899199
H	6.796681	0.182020	4.041035
H	5.450204	-1.916256	4.093386
H	4.344488	-2.741690	2.032773
H	5.178837	-3.254903	-1.355242
H	3.048627	1.820804	2.861735
H	1.506991	1.347346	2.110861
H	2.992575	0.467727	1.709892
H	2.123691	1.758804	-3.216233
H	1.962146	3.519882	-3.423007
H	3.535454	2.759576	-3.644331

IVc

SCF Energy = -3107.973359
Free Enthalpy (0K) = -3107.094797
Free Energy (298K) = -3107.191228
Free Energy (TZVP,PCM(THF),GD3BJ) = -3109.358654

**C**	4.766972	-1.154023	-0.821749
C	4.211365	-1.598383	0.389306
C	4.242278	-2.979071	0.658952
C	4.819739	-3.874731	-0.235186
C	5.376011	-3.415743	-1.430989
C	5.344433	-2.052558	-1.718196
C	3.666797	-0.649004	1.372817
C	3.165756	0.764179	1.041323
C	2.539973	1.182036	2.387134
C	3.280121	0.393598	3.484606
C	3.710005	-0.824105	2.705622
C	2.272960	0.931470	-0.168862
O	1.082448	0.350445	-0.160067
Sm	-1.045624	-0.027130	-0.049961
O	-3.093463	-1.695022	-0.378217

C	-3.198128	-2.981366	0.252223
C	-4.623935	-3.439611	0.001011
C	-4.913606	-2.814850	-1.358507
C	-4.225690	-1.467047	-1.230514
C	4.534847	1.146312	3.957906
C	2.398174	0.067303	4.687859
C	2.738132	1.641192	-1.368391
C	3.371517	2.919666	-1.281377
C	3.807594	3.561053	-2.443793
C	3.639222	2.989900	-3.699852
C	3.020923	1.746514	-3.794053
C	2.570011	1.063831	-2.664209
O	-0.332688	-2.230172	-0.950958
C	0.736080	-2.961264	-0.312431
C	1.225824	-3.944542	-1.360597
C	-0.038340	-4.210117	-2.170923
C	-0.685739	-2.837154	-2.202733
I	-1.001090	-1.189298	2.808519
I	-1.774918	0.945841	-2.882964
O	-3.383529	0.986975	0.674540
C	-4.217110	0.522940	1.746609
C	-5.248338	1.617474	1.960663
C	-5.407684	2.175456	0.551138
C	-3.979827	2.127091	0.037119
O	-0.920475	2.356644	0.691562
C	-1.138248	2.813812	2.031038
C	-0.273412	4.052727	2.164621
C	-0.357446	4.644884	0.761162
C	-0.357130	3.406694	-0.123472
H	4.135294	-1.702661	3.200621
H	1.483021	0.872993	2.375874
H	-4.692174	-0.425905	1.441561
H	-3.582104	0.313495	2.618865
H	-3.417310	3.031613	0.326600
H	-3.887704	2.005319	-1.051741
H	-4.855104	2.392341	2.639090
H	-6.182240	1.234752	2.396671
H	-5.830991	3.189603	0.519115
H	-6.059457	1.521776	-0.051637
H	-2.209035	3.055347	2.161789
H	-0.881318	1.998516	2.723397
H	0.654172	3.096125	-0.425659
H	-0.969005	3.509343	-1.031669
H	0.761815	3.765553	2.403719
H	-0.629721	4.731715	2.952464
H	0.474259	5.322333	0.521202
H	-1.295257	5.211113	0.639210
H	-2.469285	-3.667247	-0.213330
H	-2.931683	-2.871218	1.313109
H	-4.887579	-0.724450	-0.752022
H	-3.868755	-1.041093	-2.179218
H	-5.303352	-3.029804	0.766488
H	-4.722379	-4.534486	0.018325
H	-5.984894	-2.719626	-1.586667
H	-4.451947	-3.410975	-2.162866
H	1.495528	-2.239982	0.016050
H	0.321859	-3.464103	0.577275
H	-1.781724	-2.855320	-2.281582
H	-0.294809	-2.214610	-3.023641
H	1.659661	-4.847709	-0.910028
H	2.001982	-3.481360	-1.989108
H	-0.686838	-4.934344	-1.650239
H	0.158850	-4.599861	-3.179866
C	3.540625	3.660633	0.017974
C	1.978222	-0.300587	-2.849764
H	4.272923	4.547412	-2.355255
H	2.895047	1.276164	-4.773526
H	3.984025	3.509140	-4.597229
H	4.071084	1.363685	0.847216
H	3.793076	-3.350667	1.583436
H	4.833282	-4.942288	0.000787
H	5.829309	-4.118431	-2.134647
H	5.774323	-1.677611	-2.650518
H	4.754585	-0.089742	-1.065750
H	2.583394	2.269701	2.554827
H	2.951801	-0.528247	5.432383
H	2.063804	0.991527	5.187924
H	1.505131	-0.499288	4.386082
H	5.141910	0.519297	4.630370
H	5.173389	1.440009	3.110167
H	4.256179	2.058253	4.511469
H	2.238386	-0.698523	-3.841840
H	2.341848	-1.000624	-2.084697
H	0.881147	-0.265568	-2.776951
H	3.638387	4.740535	-0.167301
H	2.685943	3.503709	0.691804
H	4.440337	3.351042	0.576423

3c

SCF Energy = -3108.010185
Free Enthalpy (0K) = -3107.131666
Free Energy (298K) = -3107.235195
Free Energy (TZVP,PCM(THF),GD3BJ) = -3109.401573

C	4.696707	-1.936516	2.018707
C	4.734073	-1.106742	0.885038
C	5.473531	0.083845	0.960522
C	6.154960	0.429241	2.125399
C	6.108354	-0.403817	3.241574
C	5.375658	-1.590055	3.182548
C	4.039828	-1.496217	-0.353768
C	3.976495	-2.734185	-0.870756
C	3.347866	-2.794815	-2.239872
C	4.457083	-2.925176	-3.295318
C	2.651194	-1.417875	-2.314180
C	3.381474	-0.501017	-1.320472
C	2.448883	0.492398	-0.624196
O	1.355063	0.134626	-0.217730
Sm	-1.266314	0.013560	0.087118
O	-3.533991	1.376178	-0.160930
C	-3.993083	1.908013	-1.404889
C	-5.490967	2.087930	-1.230966
C	-5.579690	2.461106	0.245427
C	-4.522049	1.556671	0.859140
C	2.920589	1.898945	-0.420996
C	3.347112	2.665490	-1.527638
C	3.809168	3.967659	-1.304813
C	3.857363	4.497257	-0.021231
C	3.424980	3.735939	1.060743
C	2.939422	2.435993	0.890458
C	2.355626	-3.945728	-2.393761
O	-0.782027	2.433616	-0.844760
C	-0.408016	2.818788	-2.168623
C	-0.018026	4.285701	-2.069532
C	-0.911138	4.774045	-0.933790

C	-0.892922	3.578984	0.005499
I	-1.480473	-0.992646	-2.974831
I	-1.359359	0.963198	3.166419
O	-3.356239	-1.563635	0.633898
C	-4.307790	-1.993674	-0.338849
C	-4.935393	-3.242573	0.254959
C	-4.954686	-2.892269	1.740274
C	-3.638276	-2.144822	1.912004
O	-0.421154	-2.342644	0.788772
C	-0.776201	-3.586761	0.182226
C	-0.025080	-4.658742	0.967254
C	0.214990	-3.979332	2.313191
C	0.472704	-2.545941	1.884185
H	-5.066257	-1.201967	-0.488884
H	-3.781704	-2.144419	-1.293829
H	-2.809935	-2.825578	2.184051
H	-3.665773	-1.345321	2.668027
H	-4.291906	-4.119344	0.071194
H	-5.928417	-3.460180	-0.163639
H	-5.032806	-3.766089	2.403225
H	-5.806916	-2.229399	1.962517
H	-1.871115	-3.710830	0.244466
H	-0.517107	-3.547639	-0.887246
H	1.514104	-2.402264	1.542471
H	0.243960	-1.791873	2.653021
H	0.938458	-4.887414	0.483375
H	-0.590396	-5.598839	1.042137
H	1.050650	-4.412644	2.881572
H	-0.687195	-4.028468	2.946534
H	-3.497542	2.878288	-1.590327
H	-3.705505	1.212884	-2.209238
H	-4.939173	0.569480	1.123931
H	-4.031721	1.964361	1.756037
H	-6.016275	1.137759	-1.422795
H	-5.909131	2.846551	-1.908216
H	-6.575078	2.304568	0.685580
H	-5.313227	3.521192	0.388294
H	0.403200	2.162466	-2.514848
H	-1.266060	2.660171	-2.844789
H	-1.799215	3.469297	0.619094
H	-0.025727	3.612228	0.687930
H	-0.169429	4.826743	-3.014853
H	1.041472	4.382960	-1.782892
H	-1.933152	4.964584	-1.301338
H	-0.549864	5.694073	-0.452525
C	3.287356	2.171894	-2.951367
C	2.488503	1.658980	2.092449
H	4.125778	4.572903	-2.158985
H	3.463138	4.153985	2.069957
H	4.230319	5.512100	0.138978
H	4.182330	0.075343	-1.810474
H	2.643182	-1.009784	-3.336228
H	1.600468	-1.512933	-2.000806
H	5.036725	-3.850924	-3.151144
H	4.025460	-2.953468	-4.308934
H	5.160532	-2.079427	-3.244466
H	2.864379	-4.921019	-2.328219
H	1.582121	-3.903749	-1.613897
H	1.844161	-3.892951	-3.367615
H	5.522691	0.750173	0.096338
H	6.723460	1.361899	2.159531
H	6.636656	-0.128530	4.157450
H	5.324720	-2.245956	4.055246
H	4.107376	-2.856546	1.986295
H	4.453191	-3.606258	-0.411624
H	2.833483	2.146615	3.014270
H	1.389934	1.591431	2.151694
H	2.875831	0.629691	2.091115
H	2.613419	1.317821	-3.092248
H	2.930693	2.975282	-3.613535
H	4.284492	1.878117	-3.319718

11. Supplementary References:

1. Szostak, M.; Spain, M.; Procter, D. J. *J. Org. Chem.* **2012,** *77*, 3049–3059.

2. Hao, W.; Harenberg, J. H.; Wu, X.; MacMillan, S. N.; Lin, S. *J. Am. Chem. Soc.* **2018**, *140*, 3514−3517.

3. Feuillastre, S.; Pelotier, B.; Piva, O. *Eur. J. Org. Chem.* **2014**, 1753-1759.

4. Chen, L.; Guo, L.-N.; Ma, Z.-Y.; Gu, Y.-R.; Zhang, J.; Duan, X.-H. *J. Org. Chem.* **2019**, *84*, 6475−6482.

5. Akhtar, W. M.; Armstrong R. J.; Frost, J. R.; Stevenson, N. G.; Donohoe, T. J. *J. Am. Chem. Soc.* **2018**, *140*, 11916−11920.

6. Adamo, C.; Barone, V. *J. Chem. Phys.*, **1999**, *110*, 6158-6169.

7. Weigend, F.; Ahlrichs, R. *Phys. Chem. Chem. Phys.* **2005**, *7*, 3297-3305; Gulde, R.; Pollak, P.; Weigend, F. *J. Chem. Theory Comut.* **2012**, *8*, 4062-4068.

8. Gaussian 09, Revision D.01, M. J. Frisch, G. W. Trucks, H. B. Schlegel, G. E. Scuseria, M. A. Robb, J. R. Cheeseman, G. Scalmani, V. Barone, B. Mennucci, G. A. Petersson, H. Nakatsuji, M. Caricato, X. Li, H. P. Hratchian, A. F. Izmaylov, J. Bloino, G. Zheng, J. L. Sonnenberg, M. Hada, M. Ehara, K. Toyota, R. Fukuda, J. Hasegawa, M. Ishida, T. Nakajima, Y. Honda, O. Kitao, H. Nakai, T. Vreven, J. A. Montgomery, Jr., J. E. Peralta, F. Ogliaro, M. Bearpark, J. J. Heyd, E. Brothers, K. N. Kudin, V. N. Staroverov, R. Kobayashi, J. Normand, K. Raghavachari, A. Rendell, J. C. Burant, S. S. Iyengar, J. Tomasi, M. Cossi, N. Rega, J. M. Millam, M. Klene, J. E. Knox, J. B. Cross, V. Bakken, C. Adamo, J. Jaramillo, R. Gomperts, R. E. Stratmann, O. Yazyev, A. J. Austin, R. Cammi, C. Pomelli, J. W. Ochterski, R. L. Martin, K. Morokuma, V. G. Zakrzewski, G. A. Voth, P. Salvador, J. J. Dannenberg, S. Dapprich, A. D. Daniels, Ö. Farkas, J. B. Foresman, J. V. Ortiz, J. Cioslowski, and D. J. Fox, Gaussian, Inc., Wallingford CT, 2009.

9. Tomasi, J.; Mennucci B.; Cammi, R. *Chem. Rev.* **2005**, *105*, 2999-3094.

10. Grimme, S.; Ehrlich, S.; Goerigk, L. *J. Comput. Chem*. **2011**, *32*, 1456.

12. X-ray Structures

*X-ray structure of **3c***

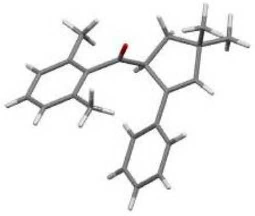

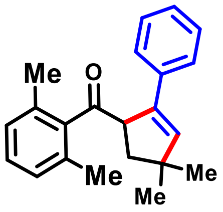

3c

CCDC 2039353

Crystal data and structure refinement of **3c**

Bond precision: C-C = 0.0030 A Wavelength=1.54184
Cell: a=18.5243(8) b=5.8984(2) c=17.0171(8)
alpha=90 beta=111.769(5) gamma=90
Temperature: 100 K

	Calculated	Reported
Volume	1726.76(14)	1726.76(15)
Space group	C c	C 1 c 1
Hall group	C -2yc	C -2yc
Moiety formula	C22 H24 O	C22 H24 O
Sum formula	C22 H24 O	C22 H24 O
Mr	304.41	304.41
Dx,g cm^{-3}	1.171	1.171
Z	4	4
Mu (mm^{-1})	0.532	0.532
F000	656.0	656.0
F000’	657.71	
h, k, lmax	23,7,21	23,7,21
Nref	3589[1796]	3367
Tmin, Tmax	0.916,0.977	0.675,1.000
Tmin’	0.916	

Correction method= # Reported T Limits: Tmin=0.675 Tmax=1.000
AbsCorr = MULTI-SCAN
Data completeness= 1.87/0.94 Theta(max)= 75.611
R(reflections)= 0.0294 (3249) wR2(reflections)= 0.0761(3367)
S = 1.070 Npar= 212

*X-ray structure of **3ar***

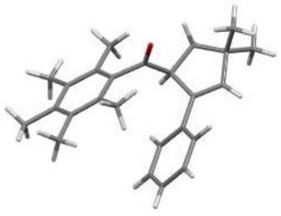

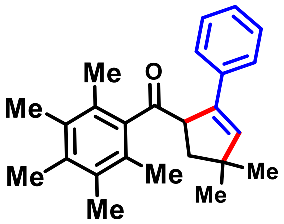

3ar

CCDC 2039352

Crystal data and structure refinement of **3ar**

Bond precision: C-C = 0.0020 A Wavelength=1.54184
Cell: a=15.6991(5) b=5.7074(2) c=21.9561(7)
alpha=90 beta=95.061(3) gamma=90
Temperature: 100 K

	Calculated	Reported
Volume	1959.62(11)	1959.62(11)
Space group	P 21/c	P 1 21/c 1
Hall group	-P 2ybc	-P 2ybc
Moiety formula	C25 H30 O	C25 H30 O
Sum formula	C25 H30 O	C25 H30 O
Mr	346.49	346.49
Dx,g cm^{-3}	1.174	1.174
Z	4	4
Mu (mm^{-1})	0.525	0.525
F000	752.0	752.0
F000’	753.92	
h, k, lmax	19,7,27	19,7,27
Nref	4094	4034
Tmin,Tmax	0.978,0.992	0.726,1.000
Tmin’	0.859	

Correction method= # Reported T Limits: Tmin=0.726 Tmax=1.000
AbsCorr = MULTI-SCAN
Data completeness= 0.985 Theta(max)= 76.145
R(reflections)= 0.0465(3296) wR2(reflections)= 0.1296(4034)
S = 1.082 Npar= 243

*X-ray structure of **5***

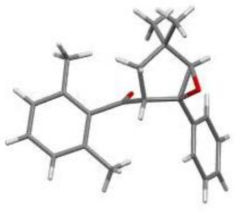

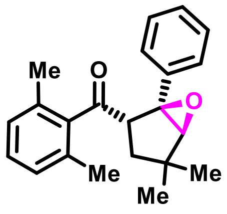

5

CCDC 2039355

Crystal data and structure refinement of **5**

Bond precision: C-C = 0.0019 A Wavelength=1.54184
Cell: a=14.8875(6) b=6.0115(2) c=20.8816(8)
alpha=90 beta=110.560(4) gamma=90
Temperature: 100 K

	Calculated	Reported
Volume	1749.79(12)	1749.79(12)
Space group	P 21/n	P 1 21/n 1
Hall group	-P 2yn	-P 2yn
Moiety formula	C22 H24 O2	C22 H24 O2
Sum formula	C22 H24 O2	C22 H24 O2
Mr	320.41	320.41
Dx,g cm^{-3}	1.216	1.216
Z	4	4
Mu (mm^{-1})	0.595	0.595
F000	688.0	688.0
F000’	689.91	
h, k, lmax	18,7,26	18,7,26
Nref	3656	3519
Tmin,Tmax	0.788,0.942	0.661,1.000
Tmin’	0.788	

Correction method= # Reported T Limits: Tmin=0.661 Tmax=1.000
AbsCorr = MULTI-SCAN
Data completeness= 0.963 Theta(max)= 75.899
R(reflections)= 0.0455(3064) wR2(reflections)= 0.1323(3519)
S = 1.076 Npar= 221

*X-ray structure of **6***

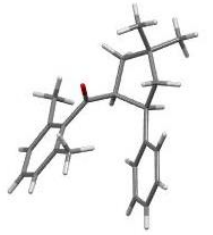

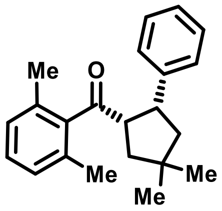

6

CCDC 2039354

Crystal data and structure refinement of **6**

Bond precision: C-C = 0.0030 A Wavelength=1.54184
Cell: a=18.5610(3) b=5.8822(1) c=16.9891(3)
alpha=90 beta=111.282(2) gamma=90
Temperature: 100 K

	Calculated	Reported
Volume	1728.37(6)	1728.37(6)
Space group	C c	C 1 c 1
Hall group	C -2yc	C -2yc
Moiety formula	C22 H26 O	C22 H26 O
Sum formula	C22 H26 O	C22 H26 O
Mr	306.43	306.43
Dx,g cm^{-3}	1.178	1.178
Z	4	4
Mu (mm^{-1})	0.532	0.532
F000	664.0	664.0
F000’	665.71	
h, k, lmax	23,7,21	23,7,21
Nref	3637[1820]	3440
Tmin,Tmax	0.987,0.989	0.756,1.000
Tmin’	0.974	

Correction method= # Reported T Limits: Tmin=0.756 Tmax=1.000
AbsCorr = MULTI-SCAN
Data completeness= 1.89/0.95 Theta(max)= 76.239
R(reflections)= 0.0293(3391) wR2(reflections)= 0.0774(3440)
S = 1.094 Npar= 212

13. NMR Spectra

^{1}H NMR (500 MHz, Chloroform-*d*) (**1a**):

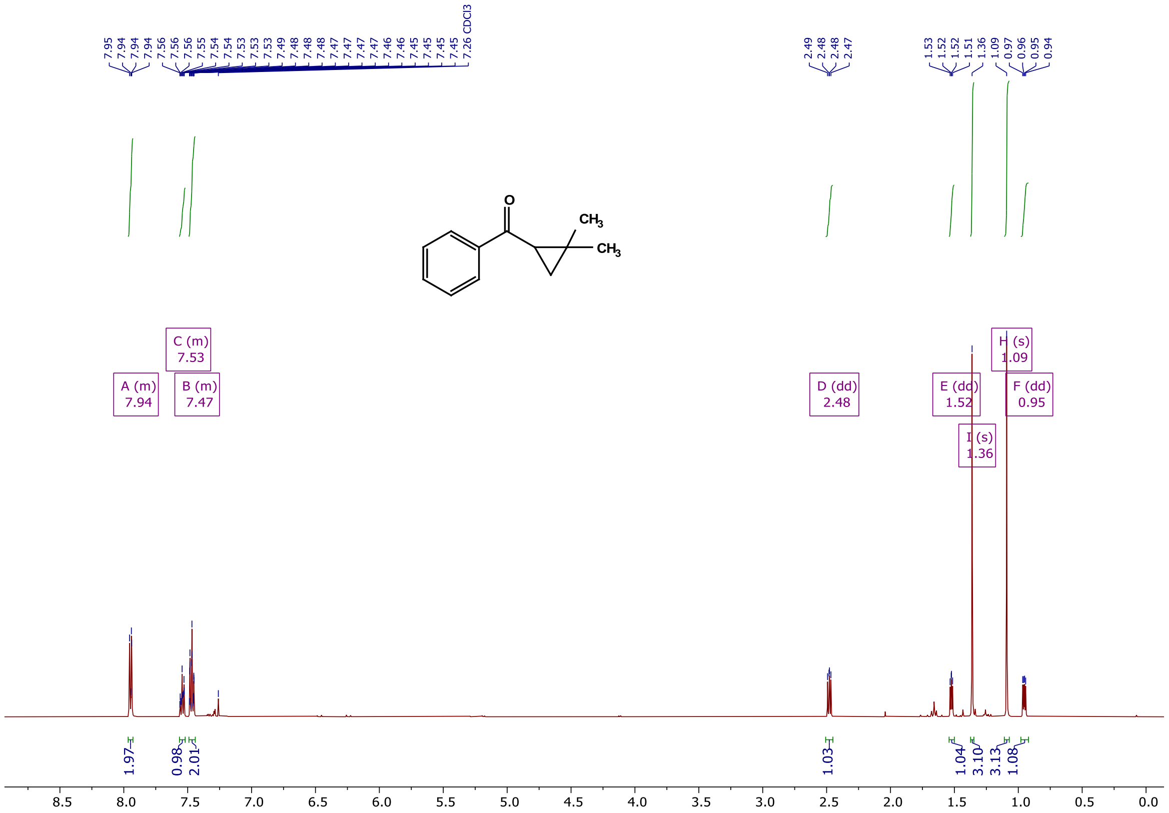

^{13}C NMR (126 MHz, Chloroform-*d*) (**1a**):

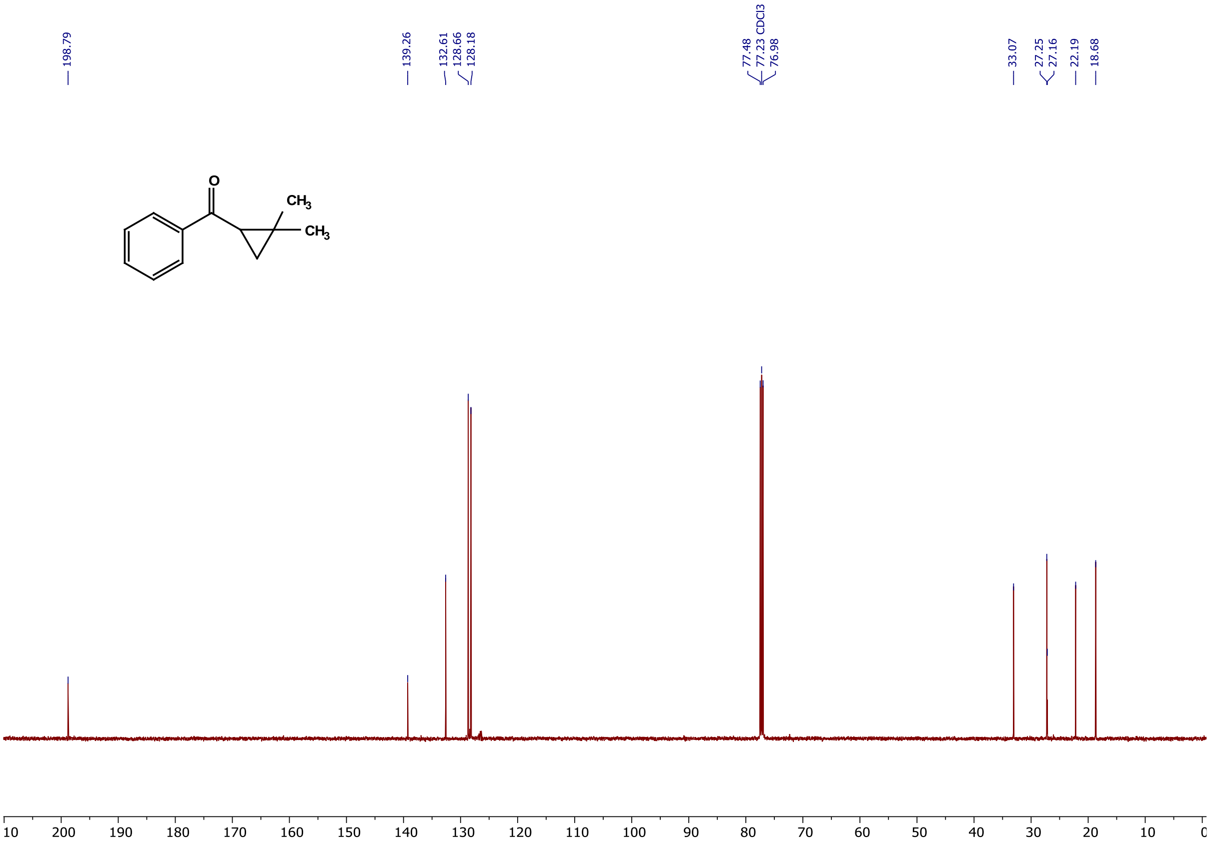

^{1}H NMR (400 MHz, Chloroform-*d*) (**1b**):

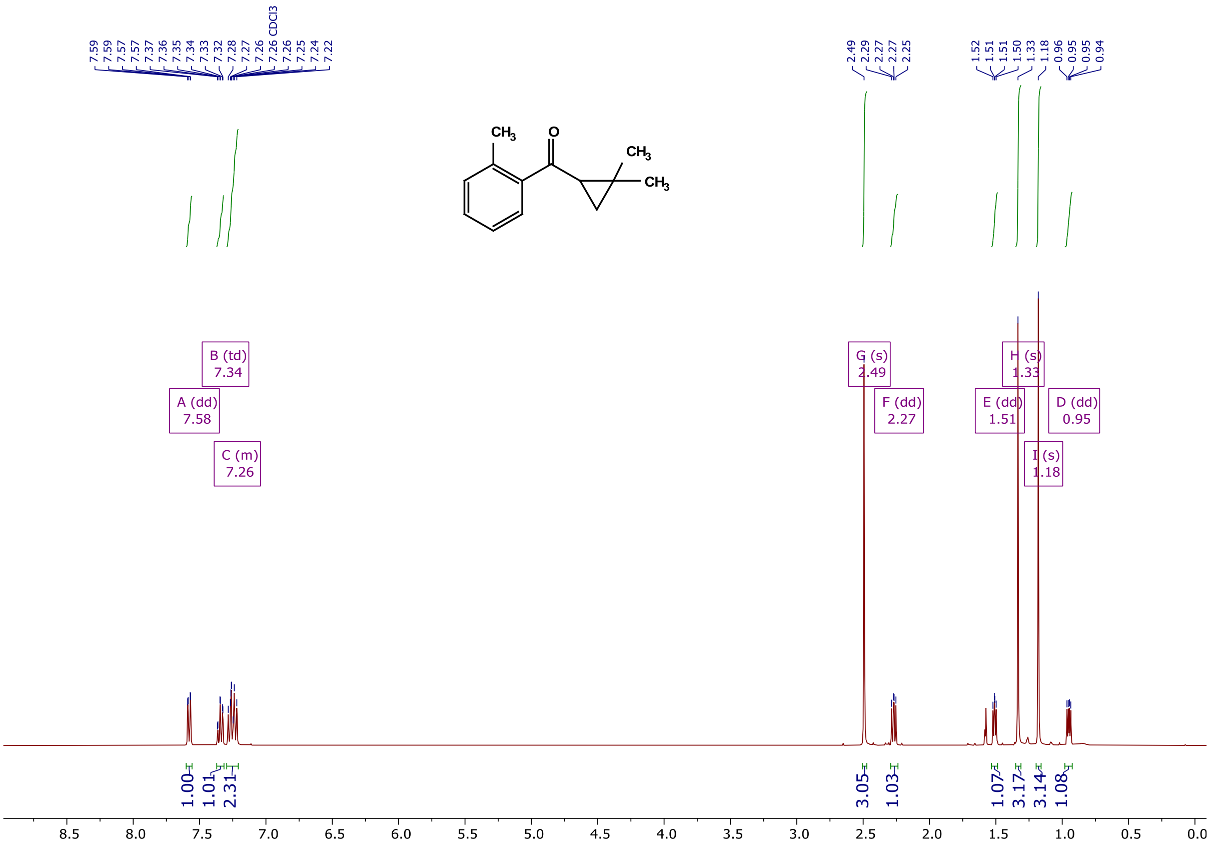

^{13}C NMR (101 MHz, Chloroform-*d*) (**1b**):

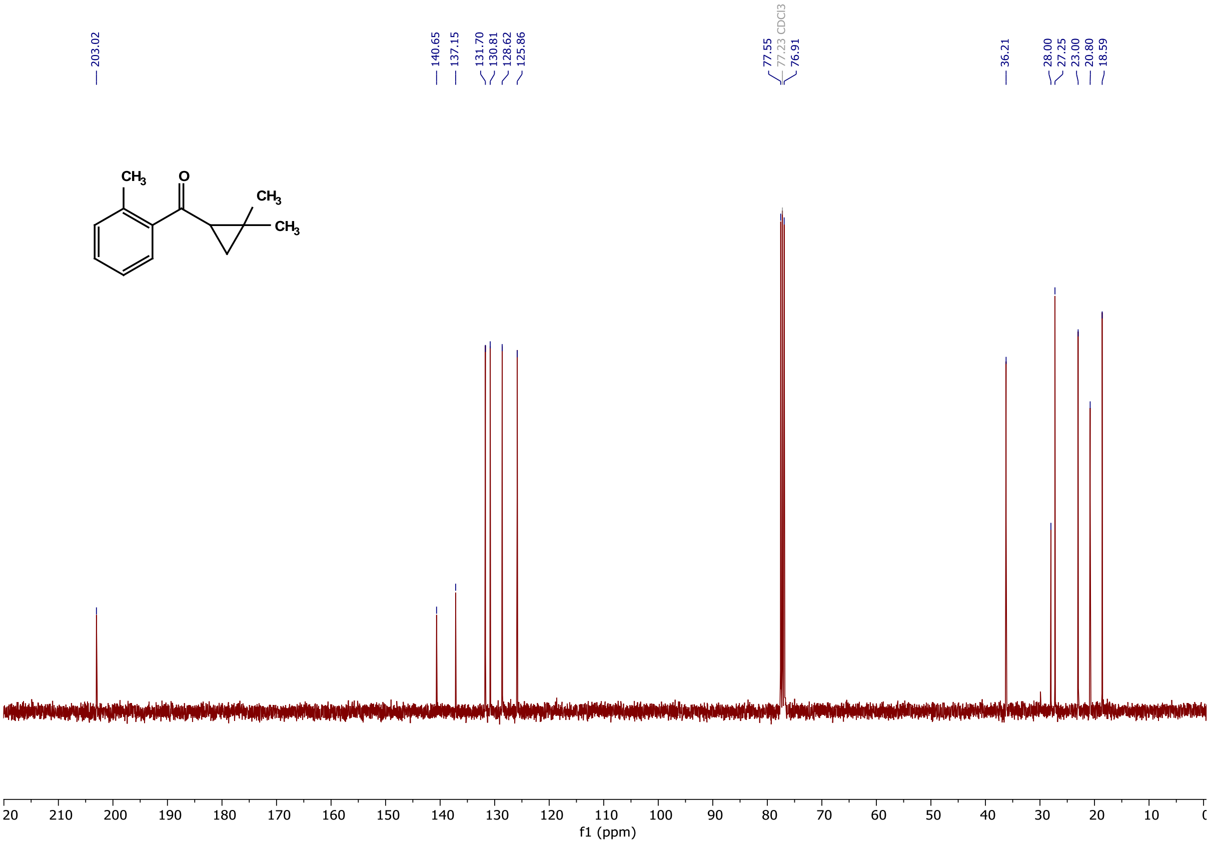

DEPT-135 NMR (101 MHz, Chloroform-*d*) (**1b**):

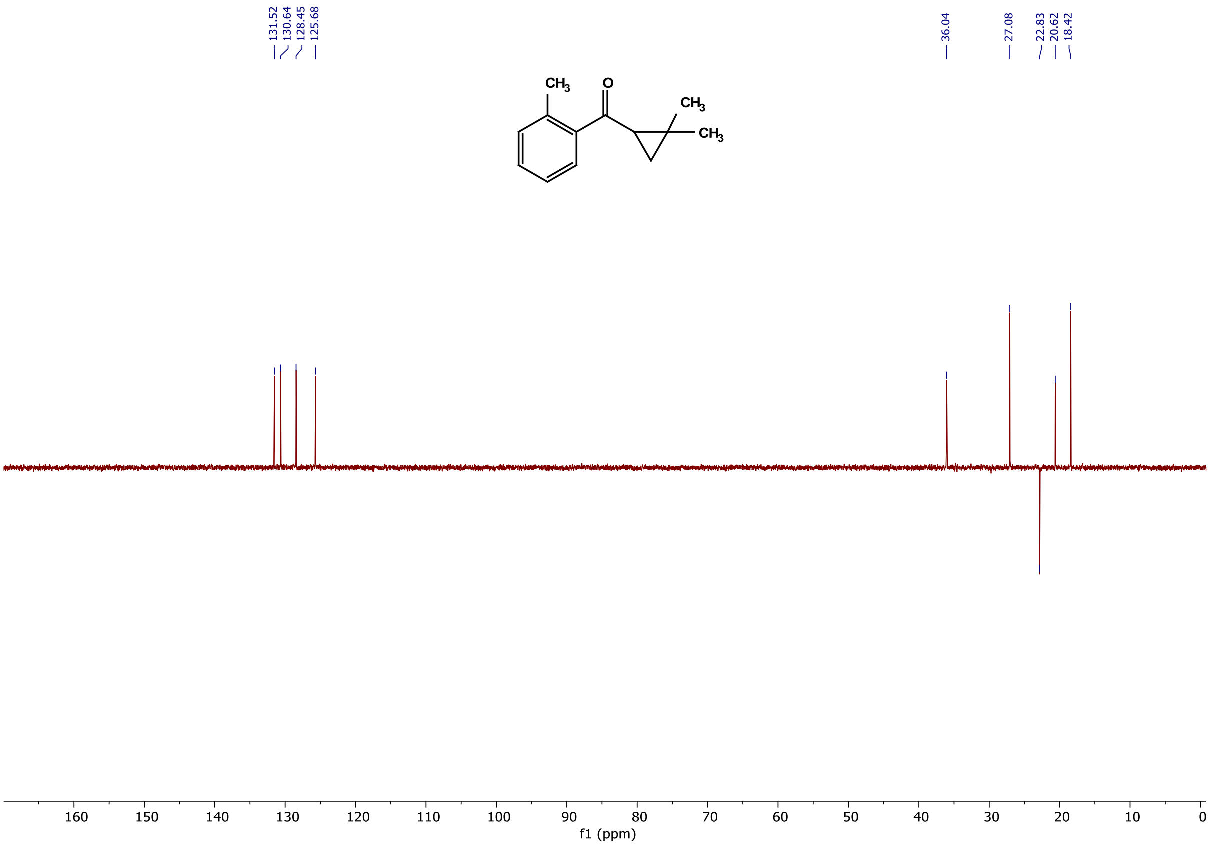

^{1}H NMR (400 MHz, Chloroform-*d*) (**1c**):

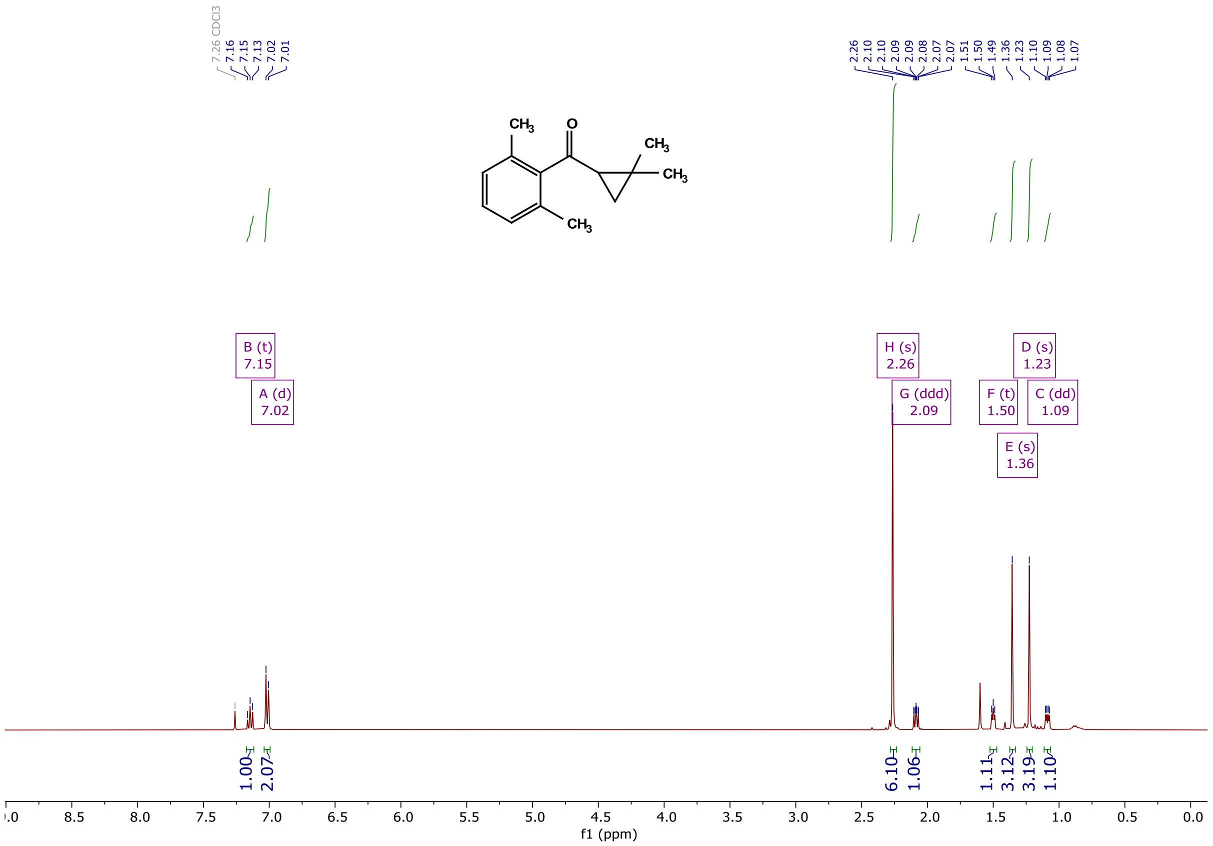

^{13}C NMR (101 MHz, Chloroform-*d*) (**1c**):

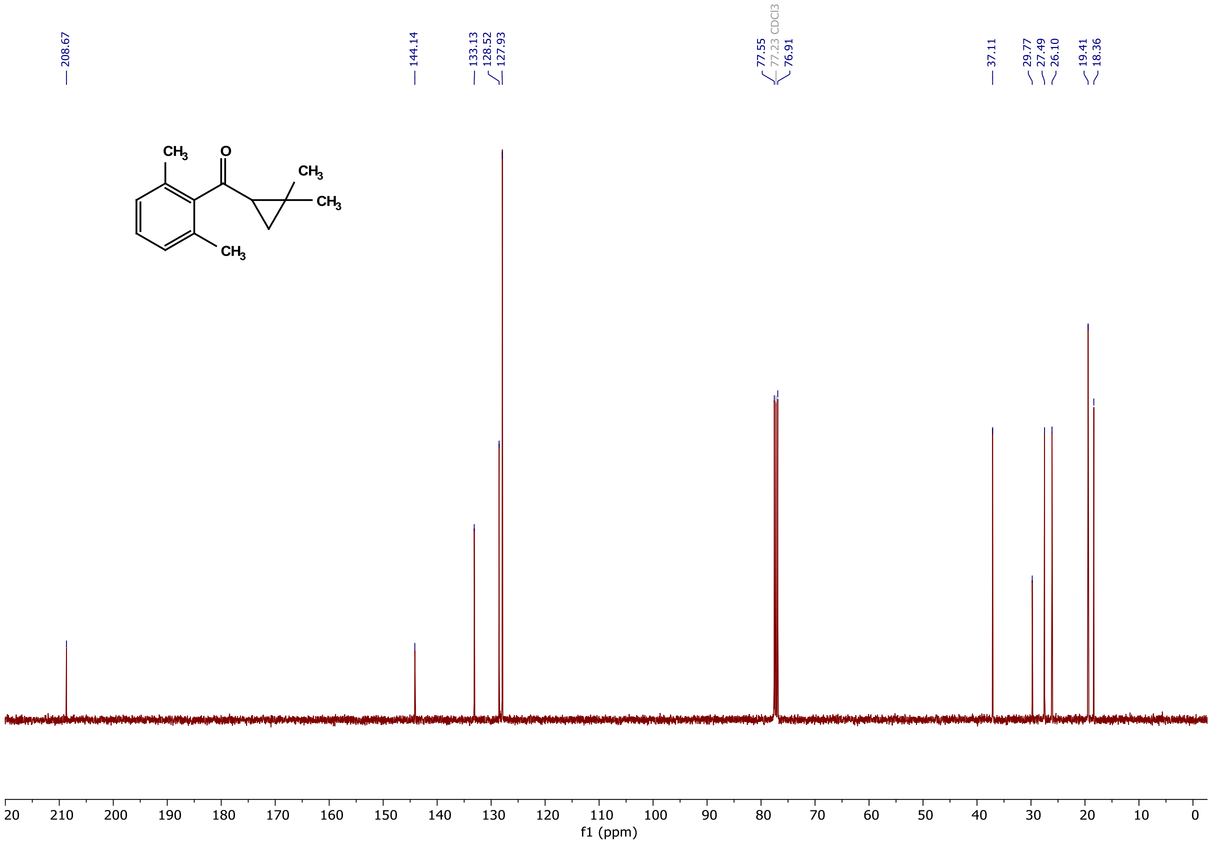

DEPT-135 NMR (101 MHz, Chloroform-*d*) (**1c**):

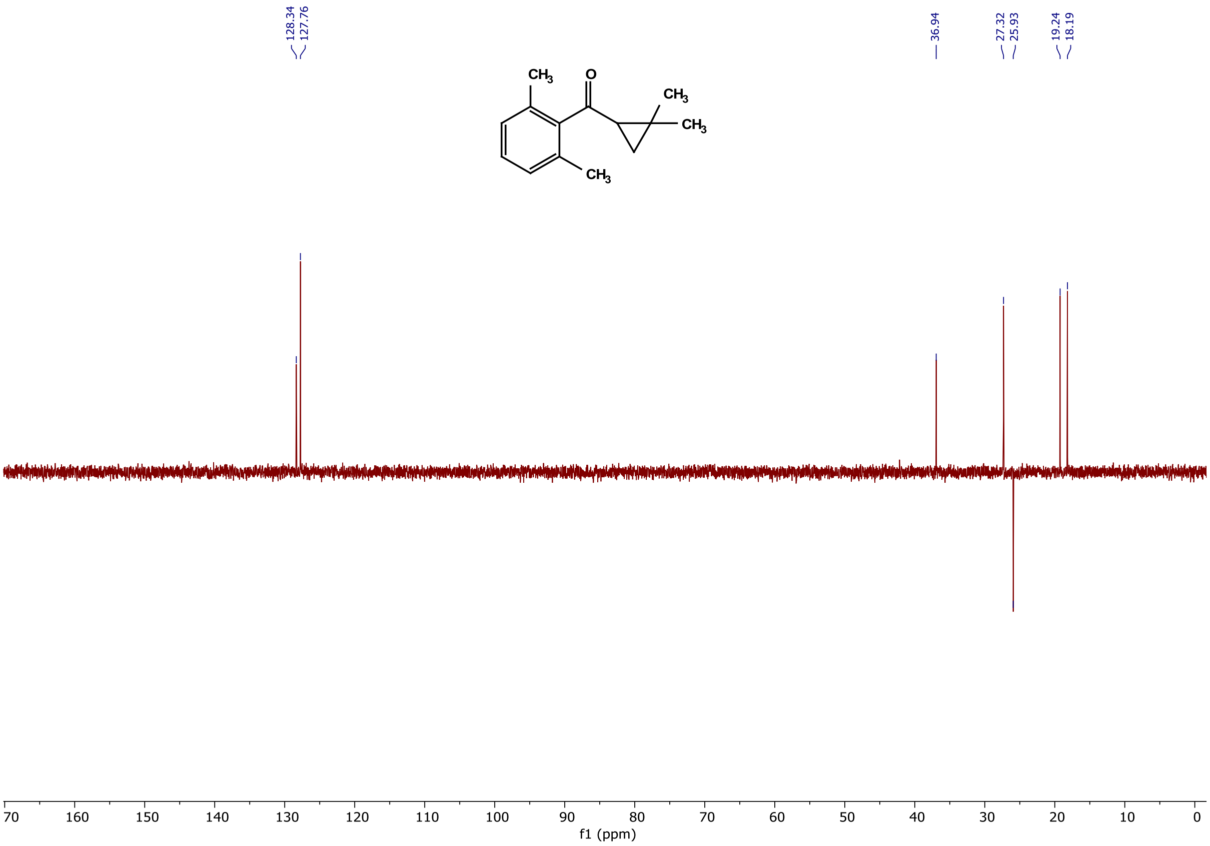

^{1}H NMR (400 MHz, Chloroform-*d*) (**1d**):

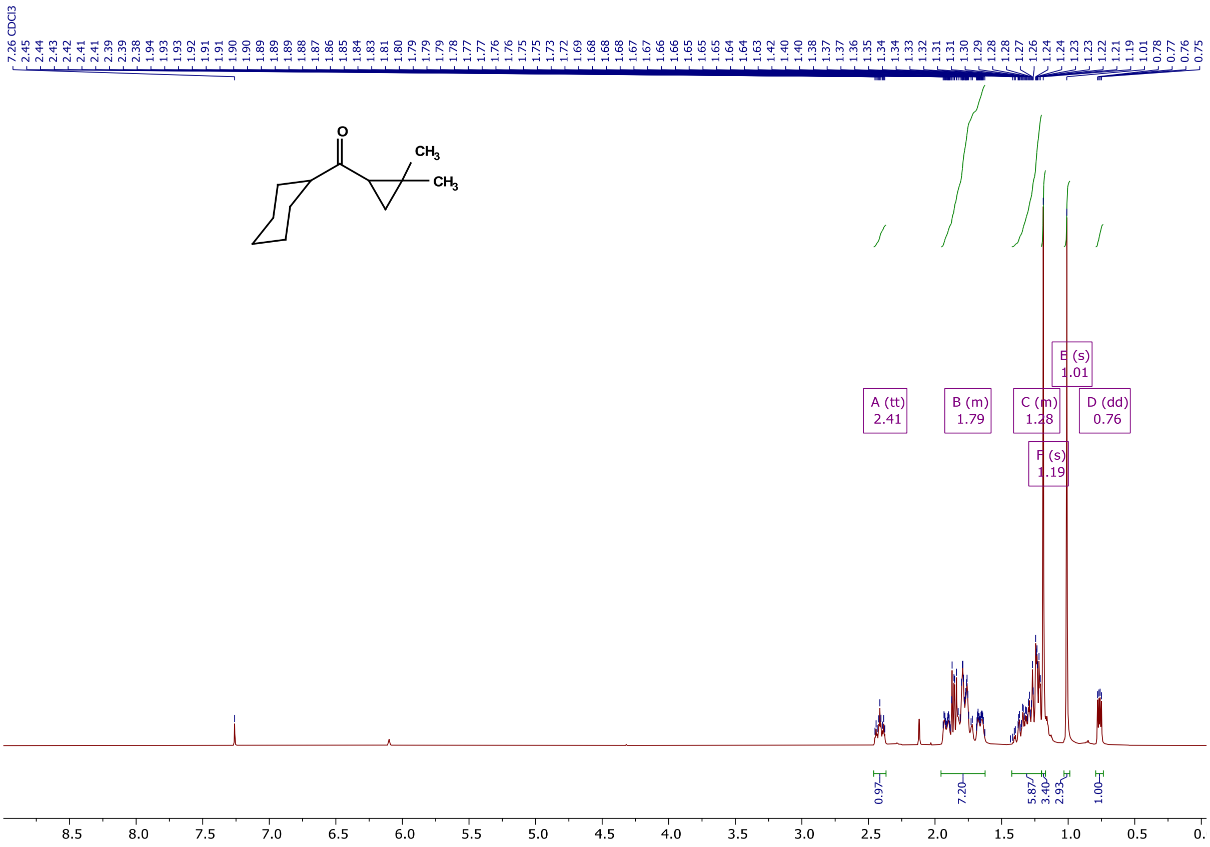

^{13}C NMR (101 MHz, Chloroform-*d*) (**1d**):

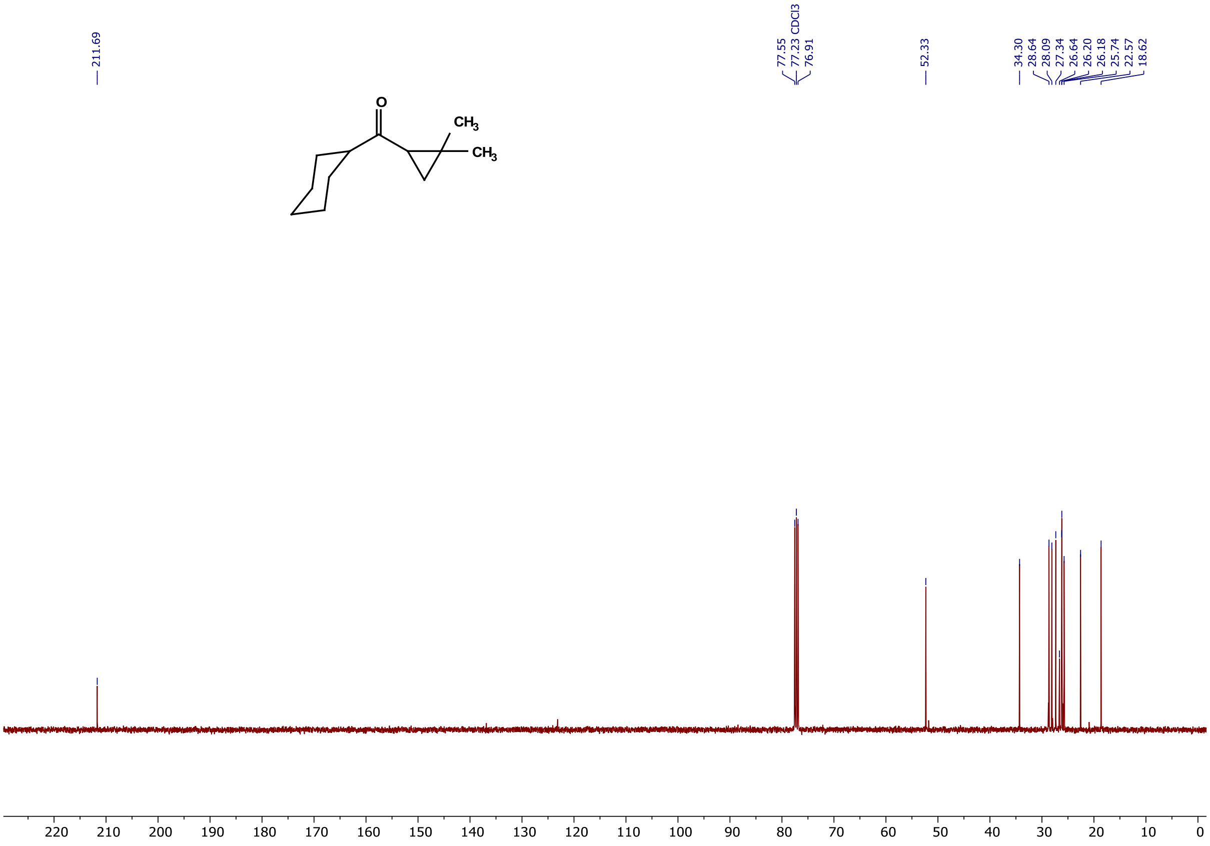

DEPT-135 NMR (101 MHz, Chloroform-*d*) (**1d**):

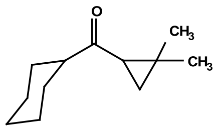

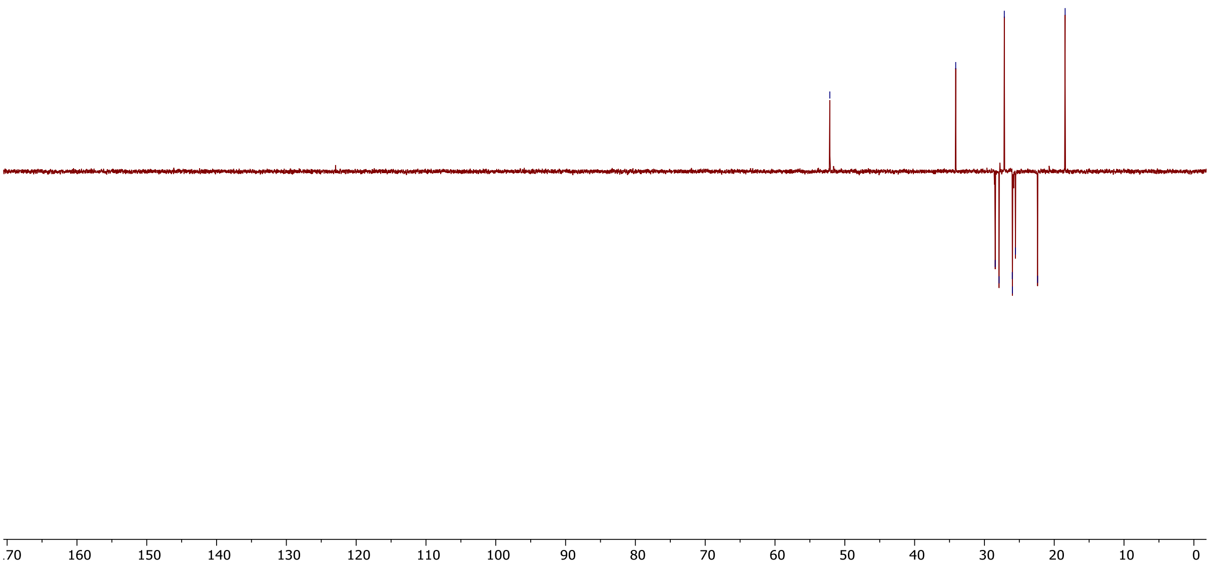

^{1}H NMR (400 MHz, Chloroform-*d*) (**1e**):

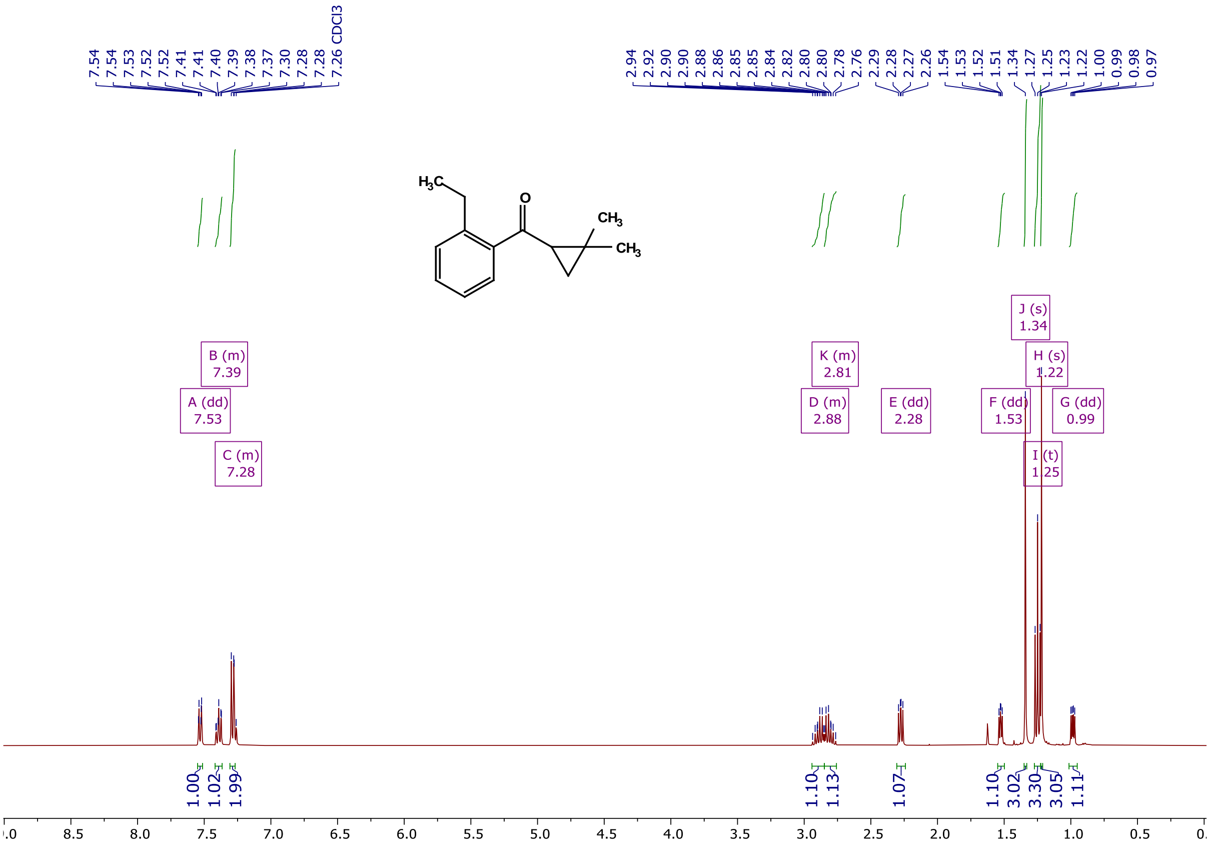

^{13}C NMR (101 MHz, Chloroform-*d*) (**1e**):

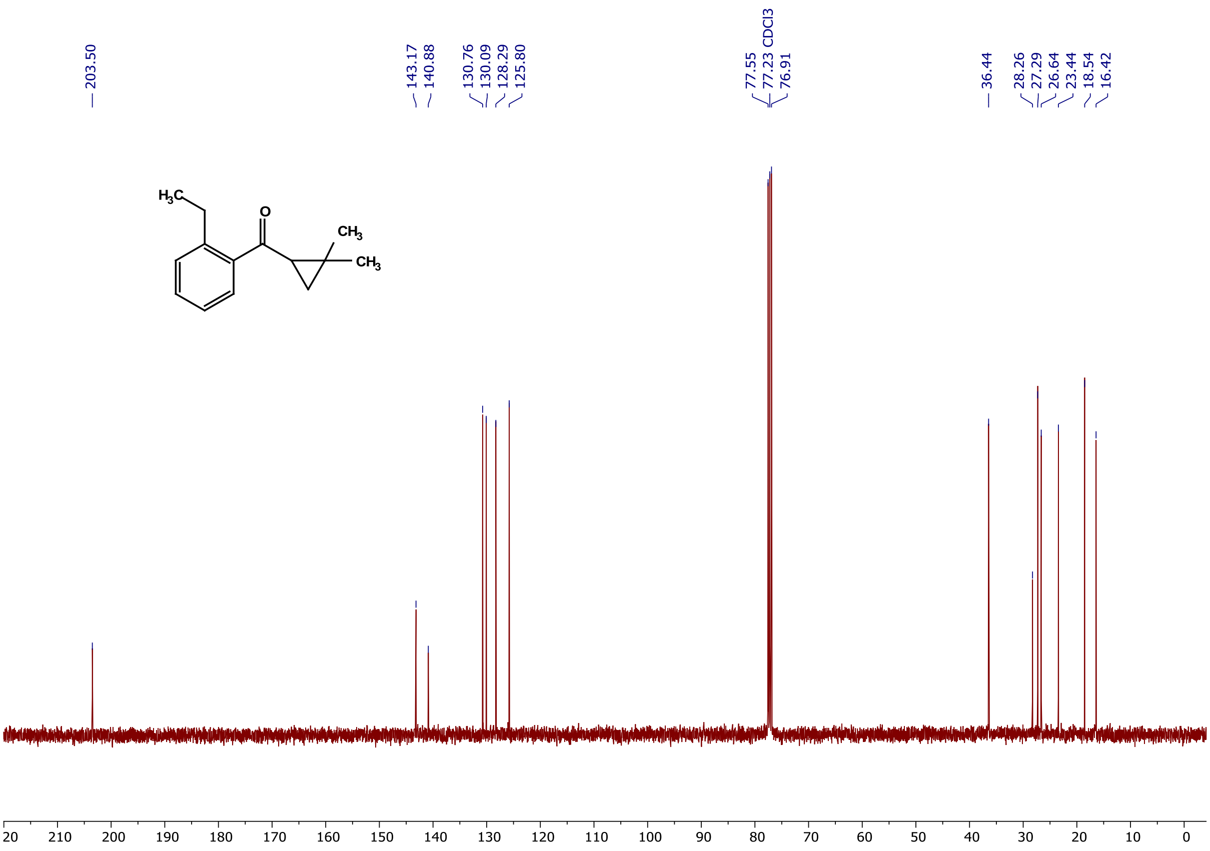

^{1}H NMR (400 MHz, Chloroform-*d*) (**1f**):

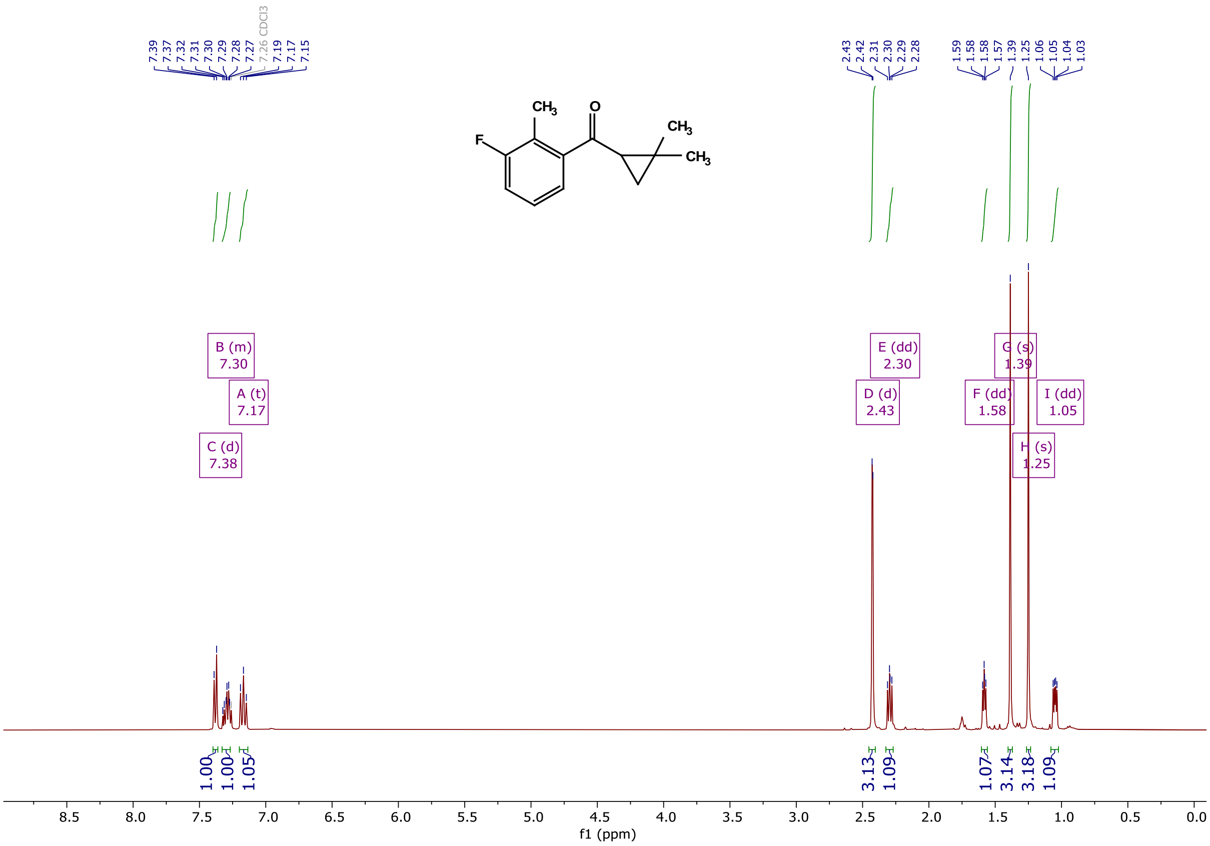

^{13}C NMR (101 MHz, Chloroform-*d*) (**1f**):

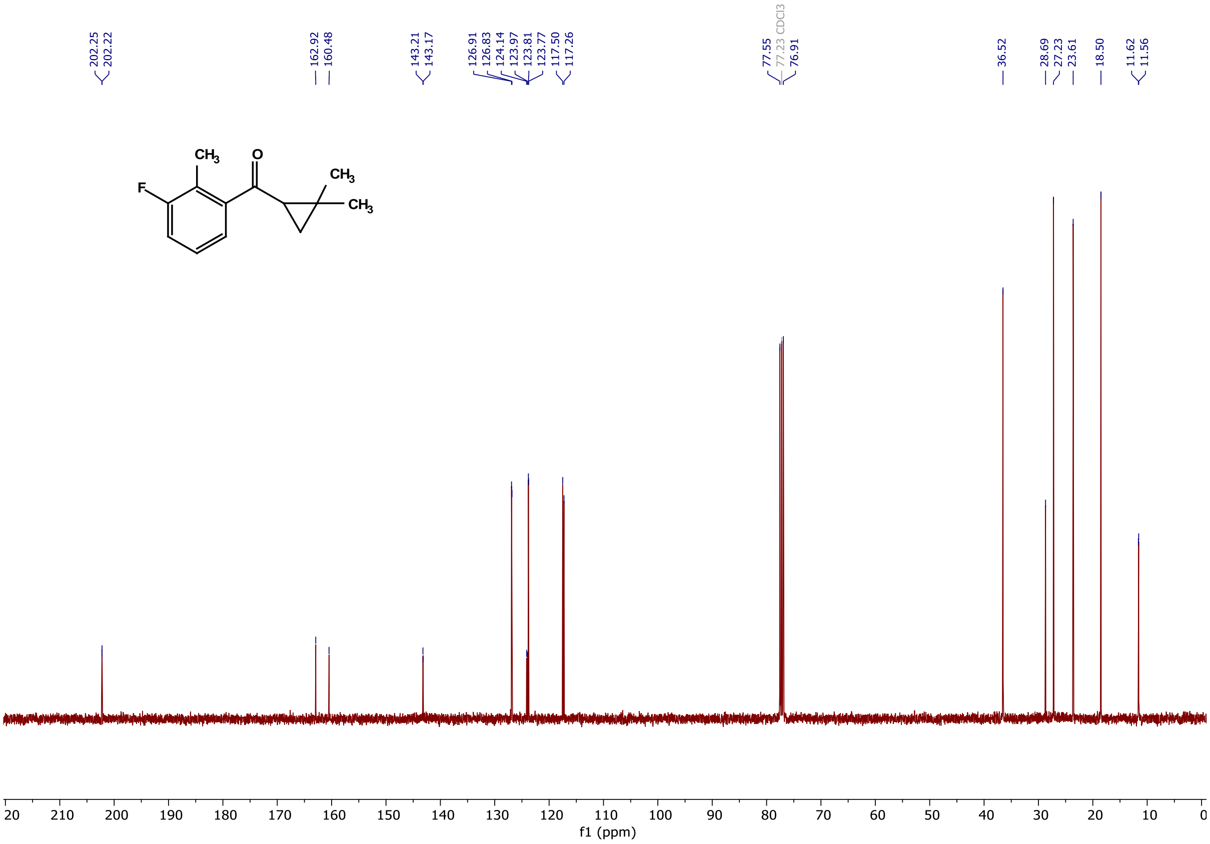

DEPT-135 NMR (101 MHz, Chloroform-*d*) (**1f**):

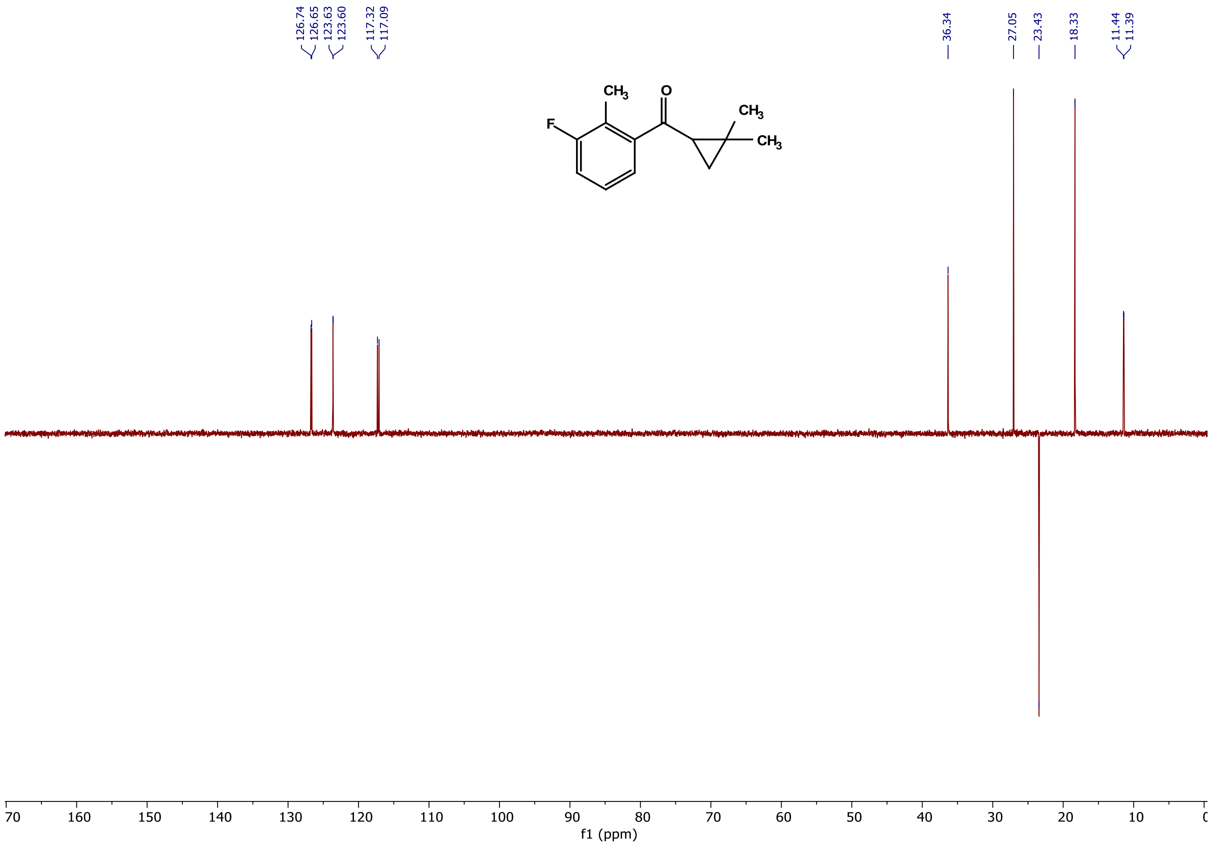

^{19}F NMR (376 MHz, Chloroform-*d*) (**1f**):

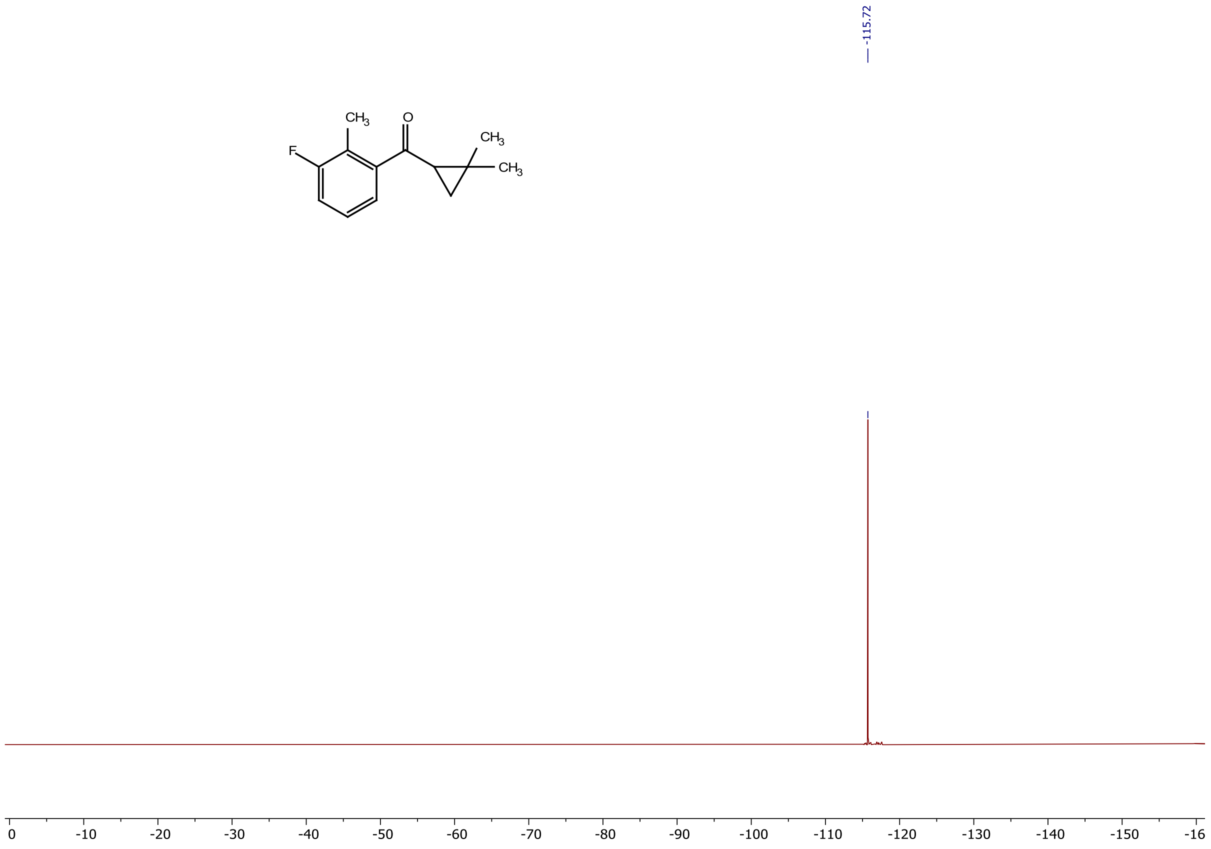

^{1}H NMR (400 MHz, Chloroform-*d*) (**1g**):

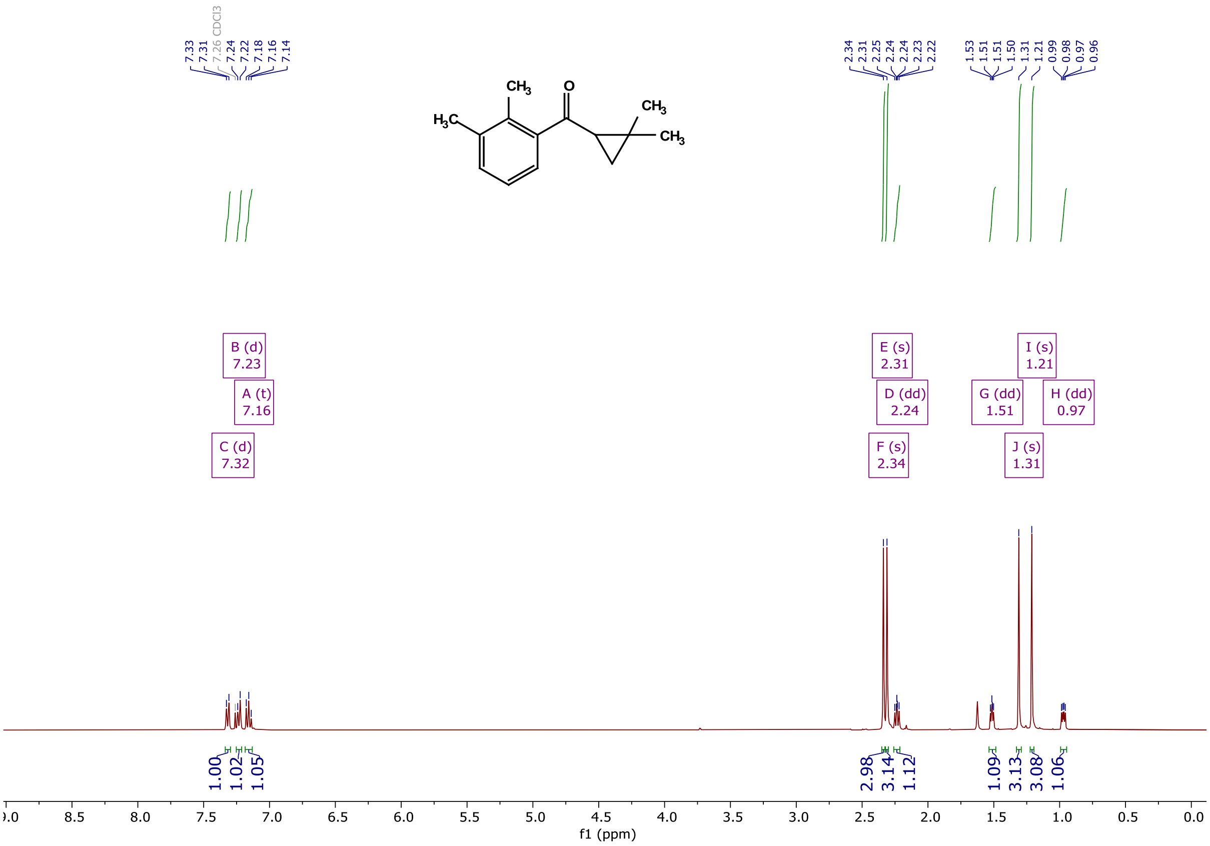

^{13}C NMR (101 MHz, Chloroform-*d*) (**1g**):

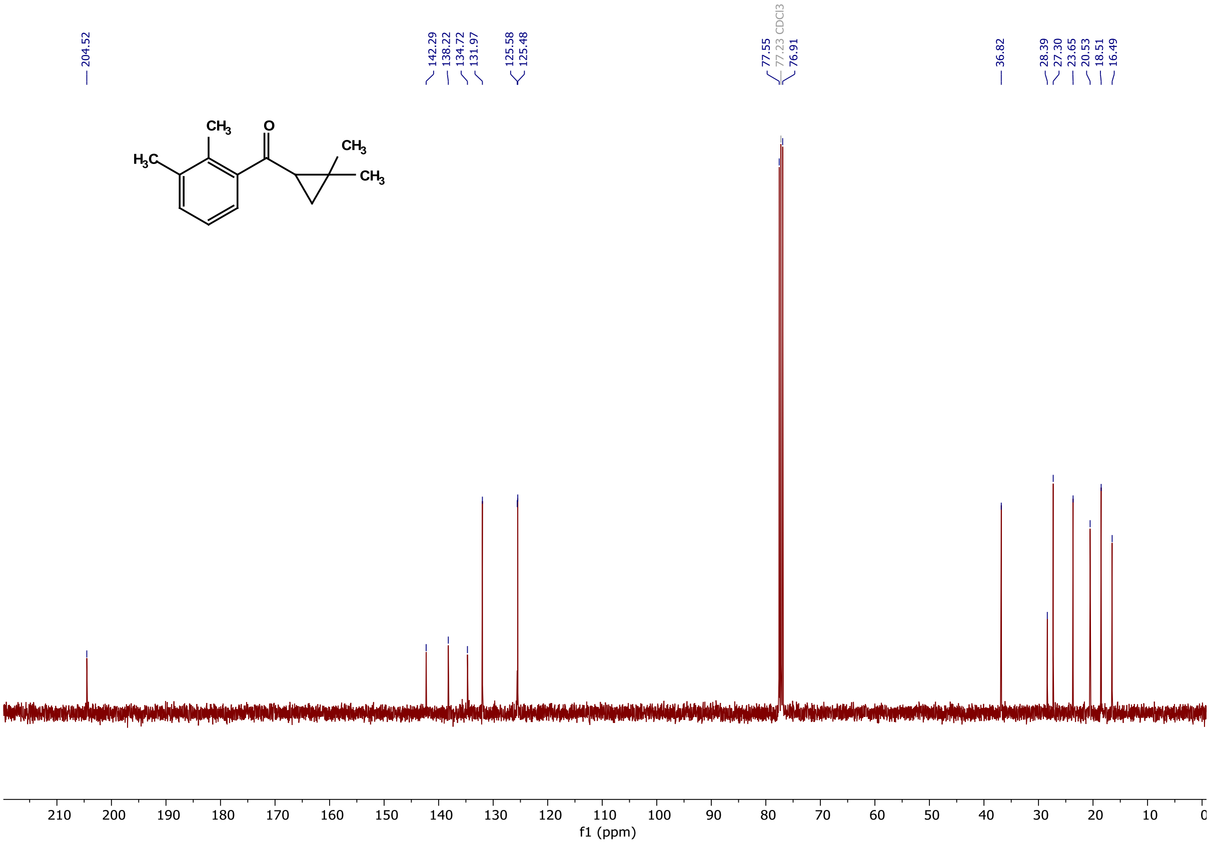

DEPT-135 NMR (101 MHz, Chloroform-*d*) (**1g**):

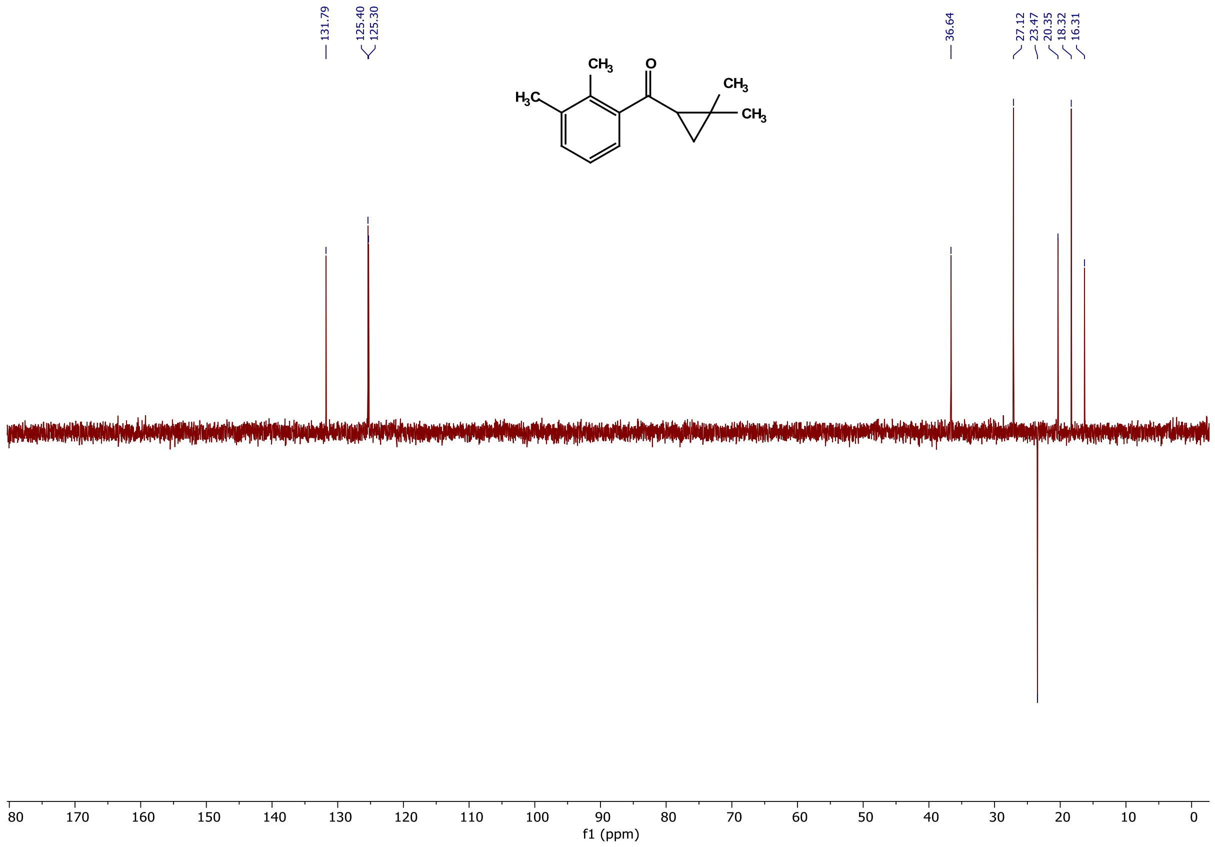

^{1}H NMR (500 MHz, Chloroform-*d*) (**1h**):

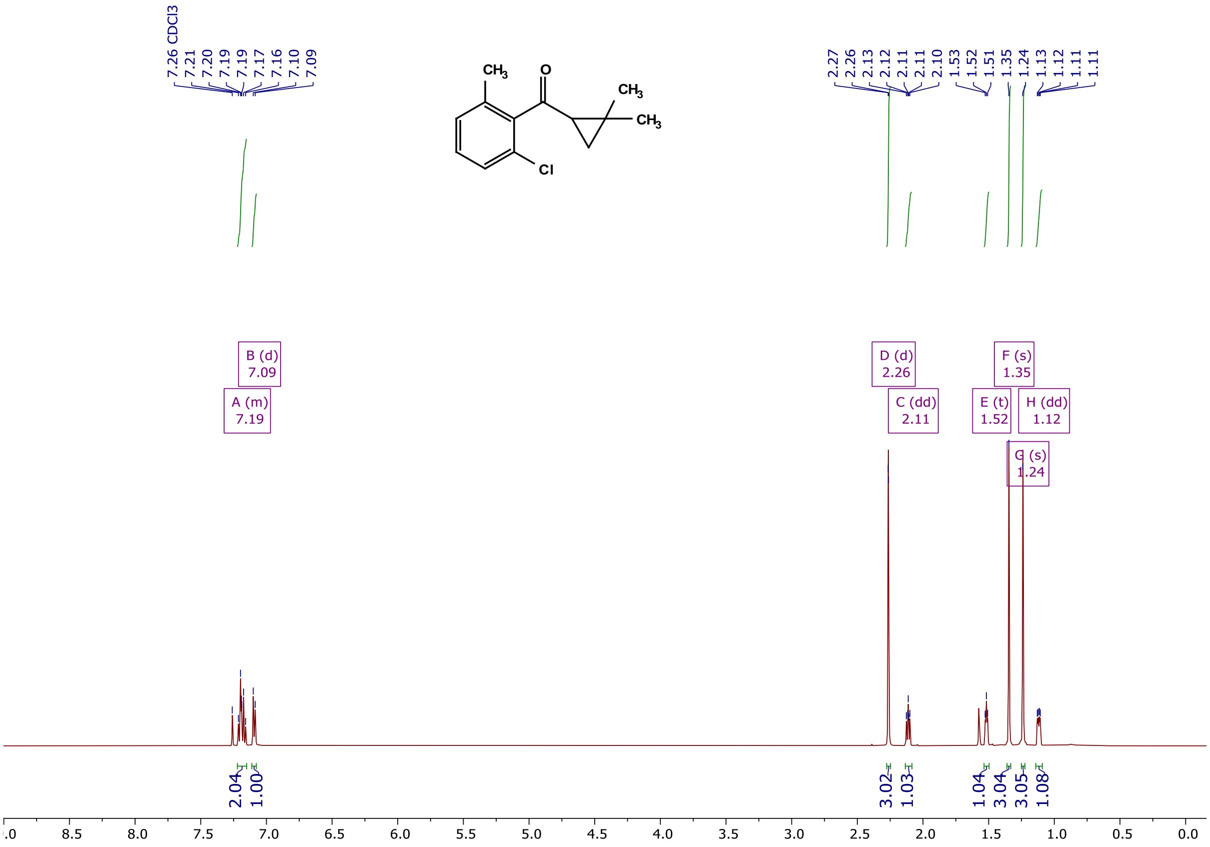

^{13}C NMR (101 MHz, Chloroform-*d*) (**1h**):

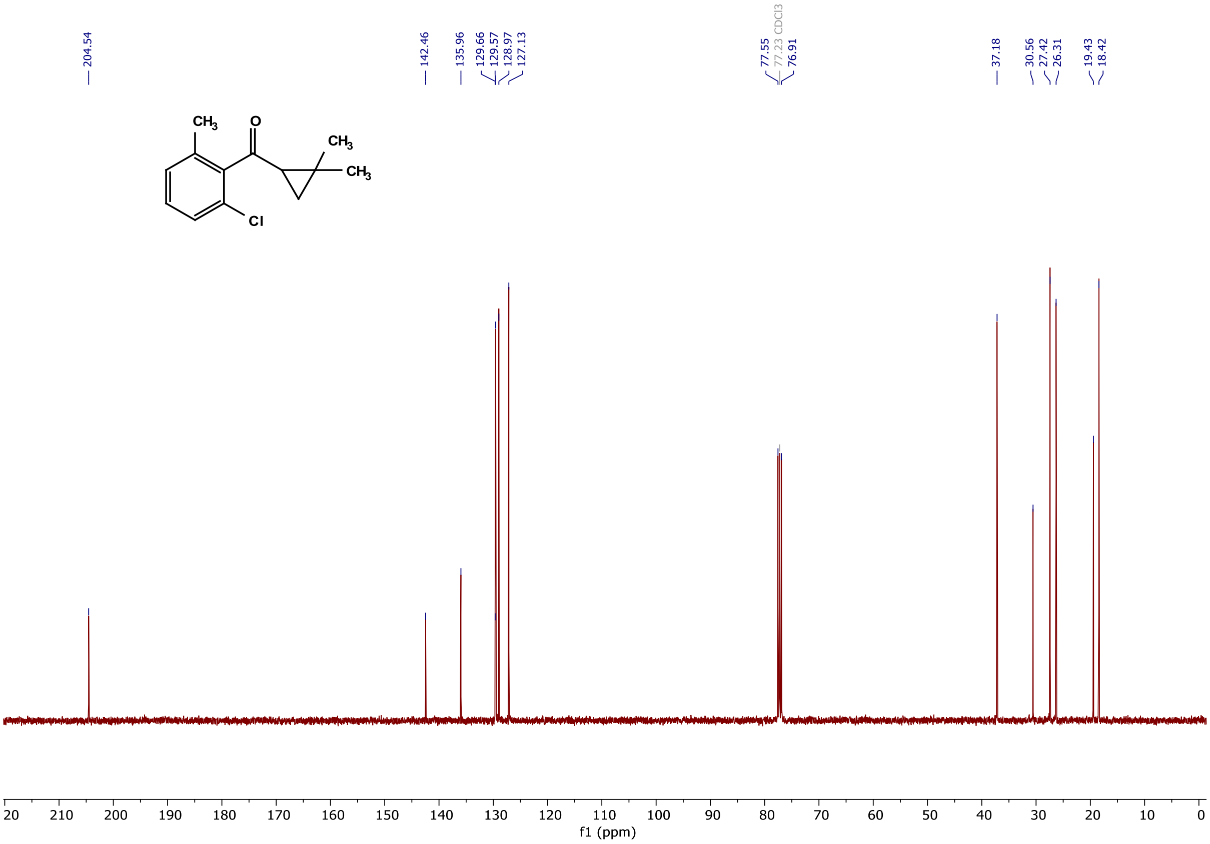

^{1}H NMR (400 MHz, Chloroform-*d*) (**1i**):

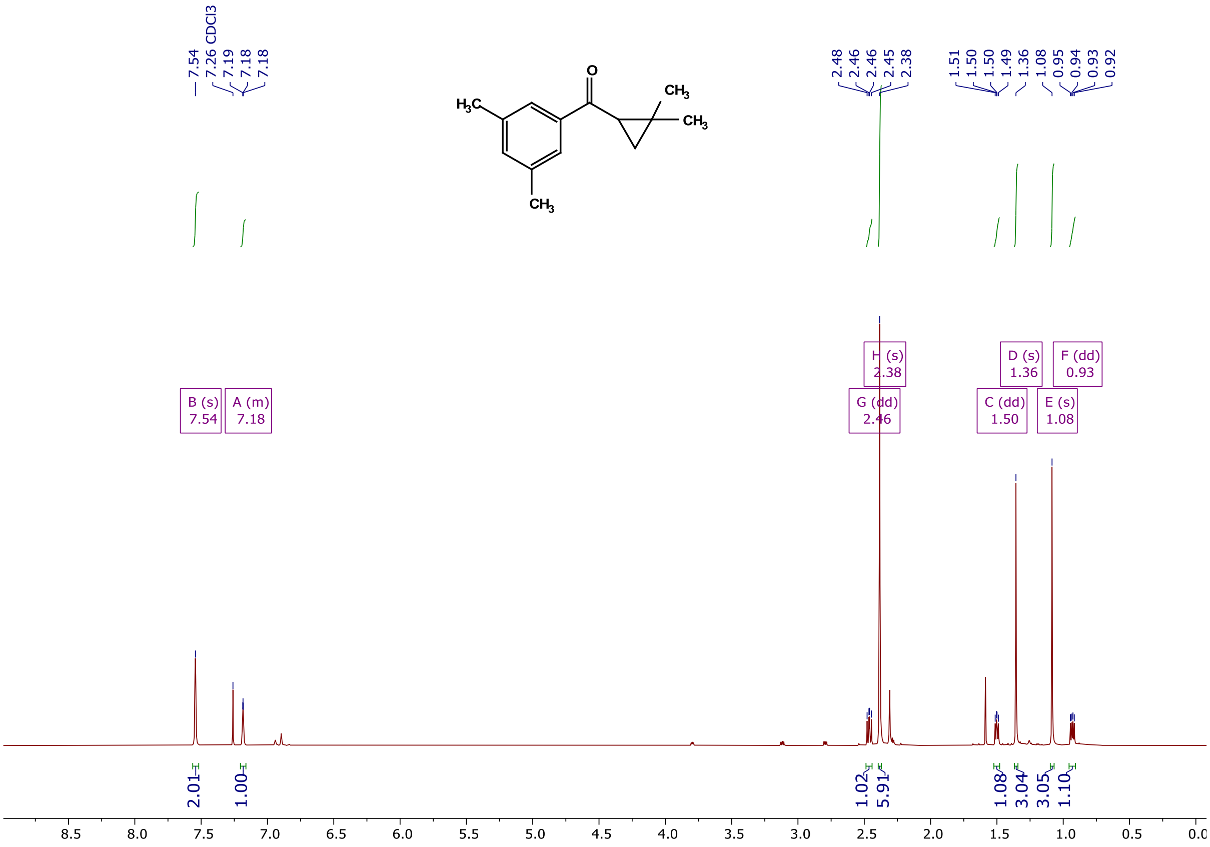

^{13}C NMR (101 MHz, Chloroform-*d*) (**1i**):

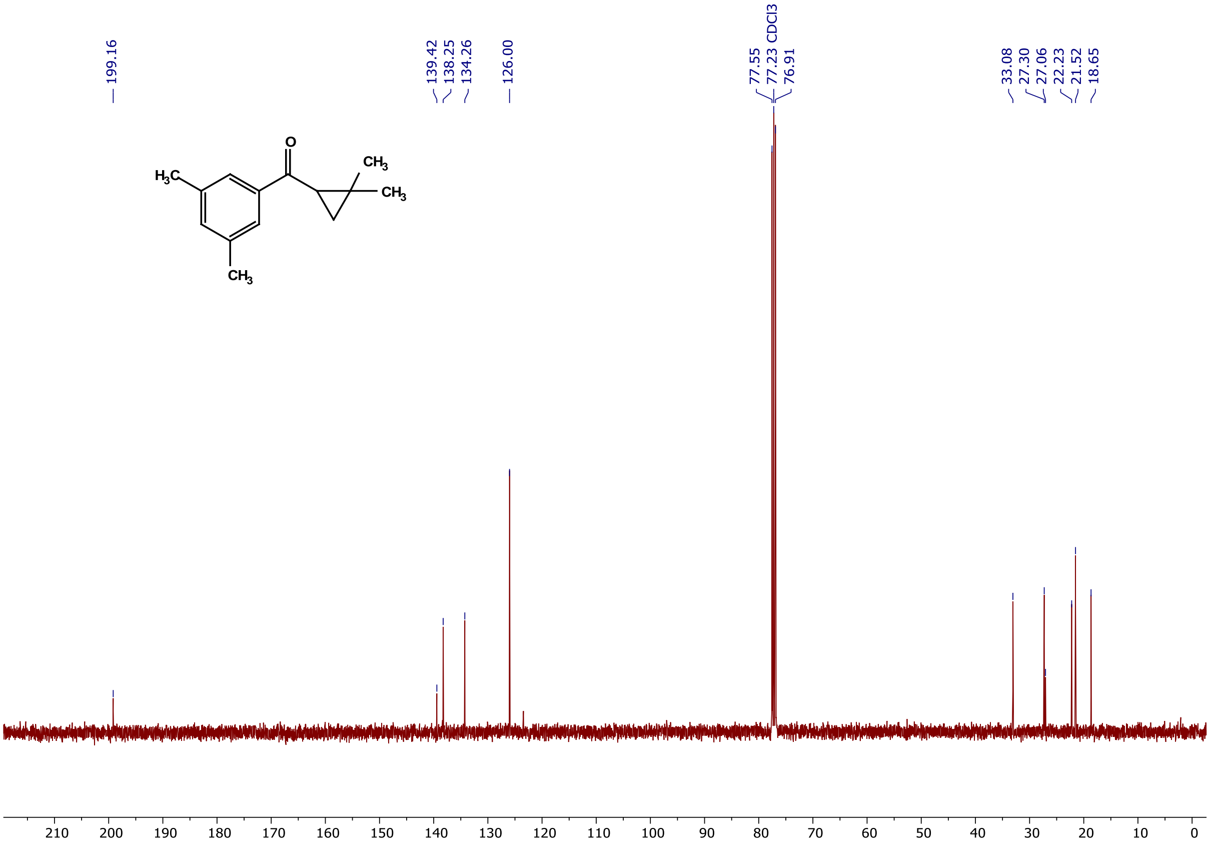

^{1}H NMR (400 MHz, Chloroform-*d*) (**1j**):

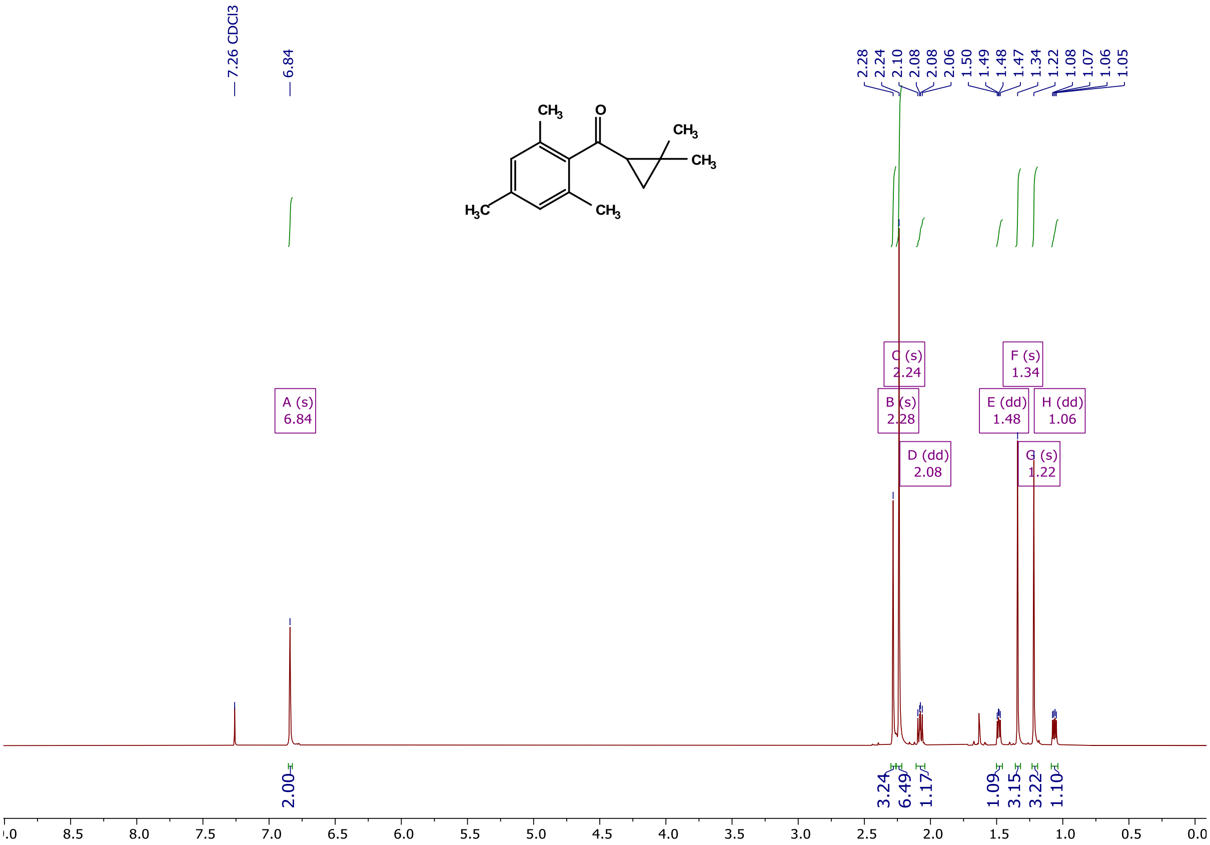

^{13}C NMR (101 MHz, Chloroform-*d*) (**1j**):

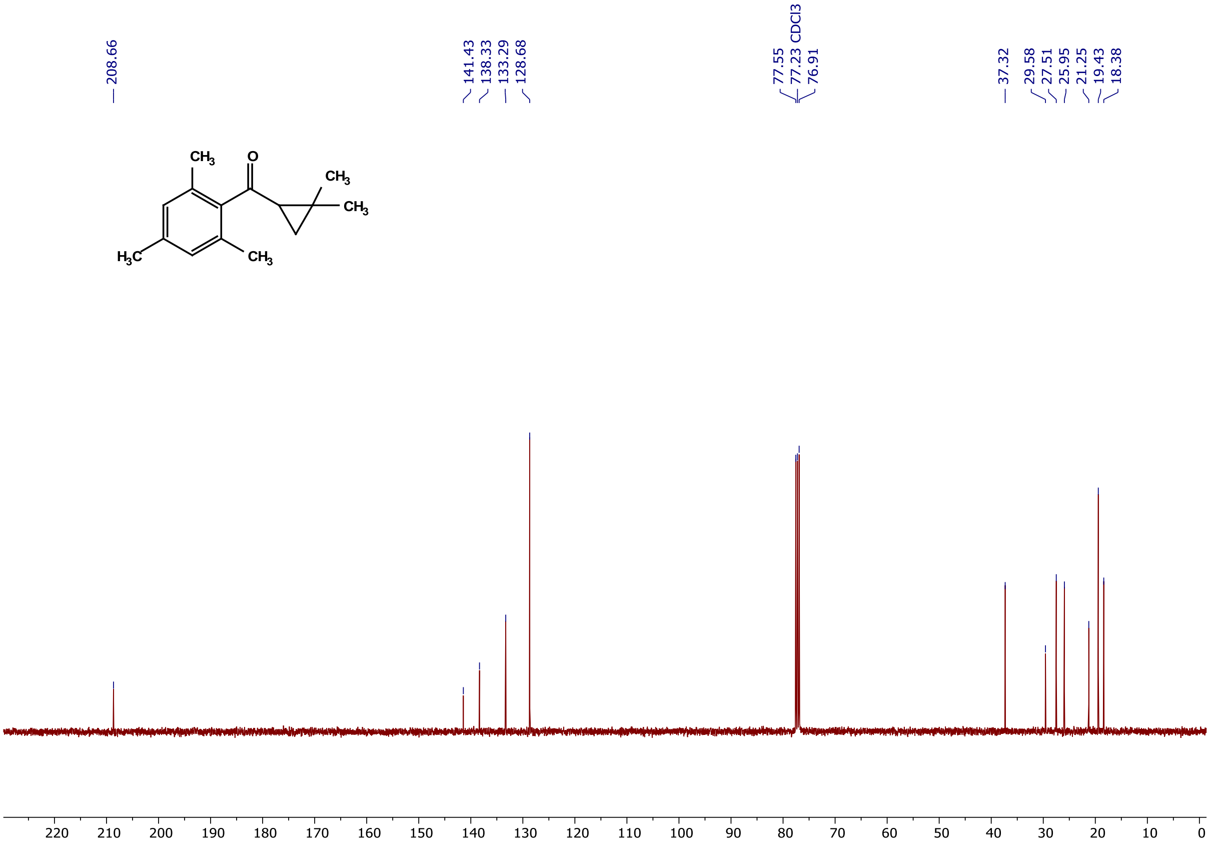

^{1}H NMR (400 MHz, Chloroform-*d*) (**1k**):

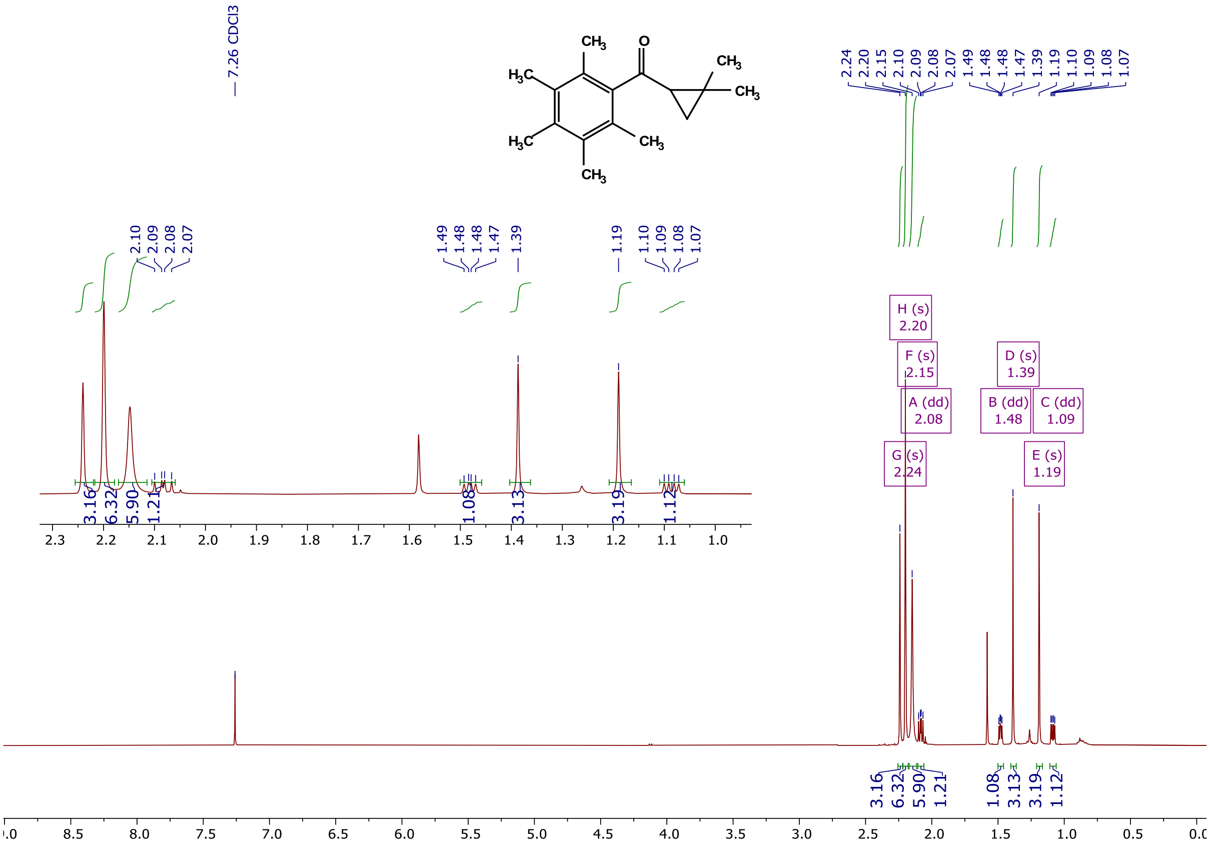

^{13}C NMR (101 MHz, Chloroform-*d*) (**1k**):

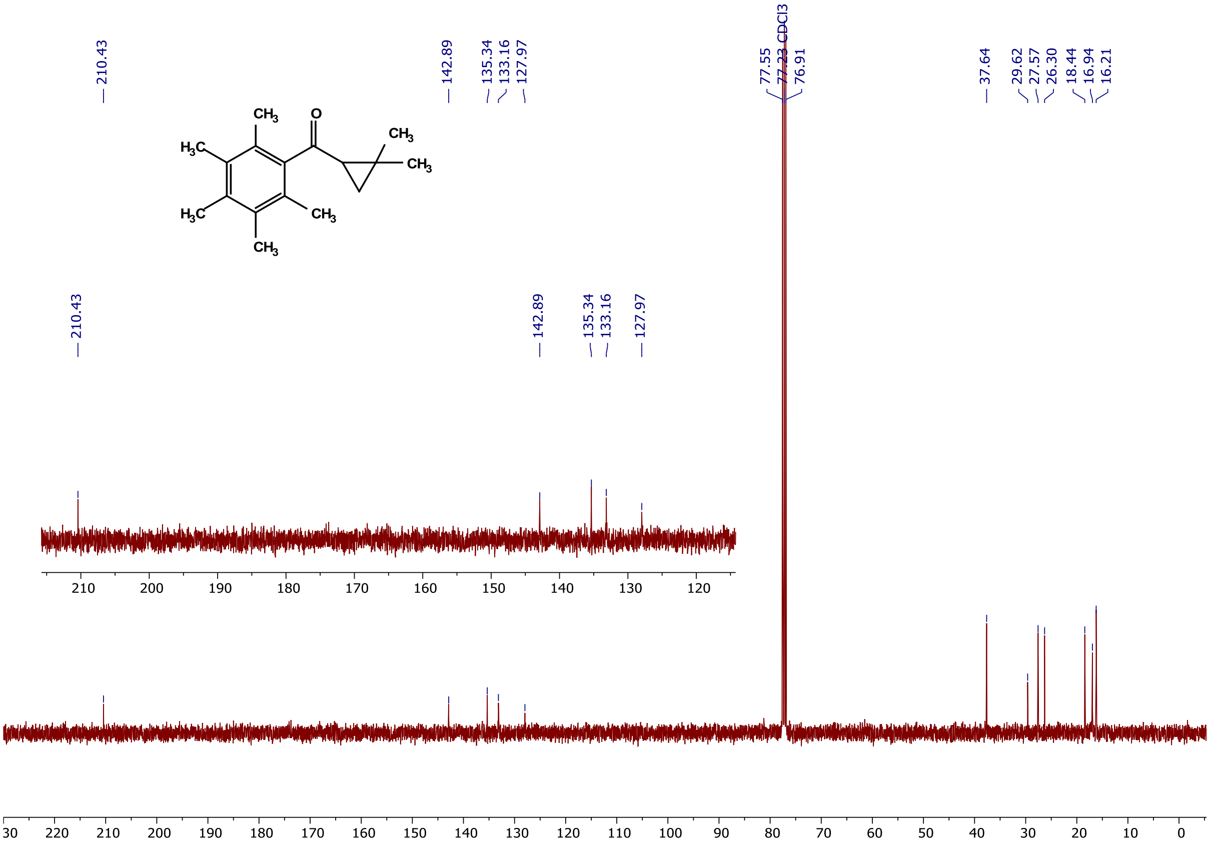

^{1}H NMR (400 MHz, Chloroform-*d*) (**1l**):

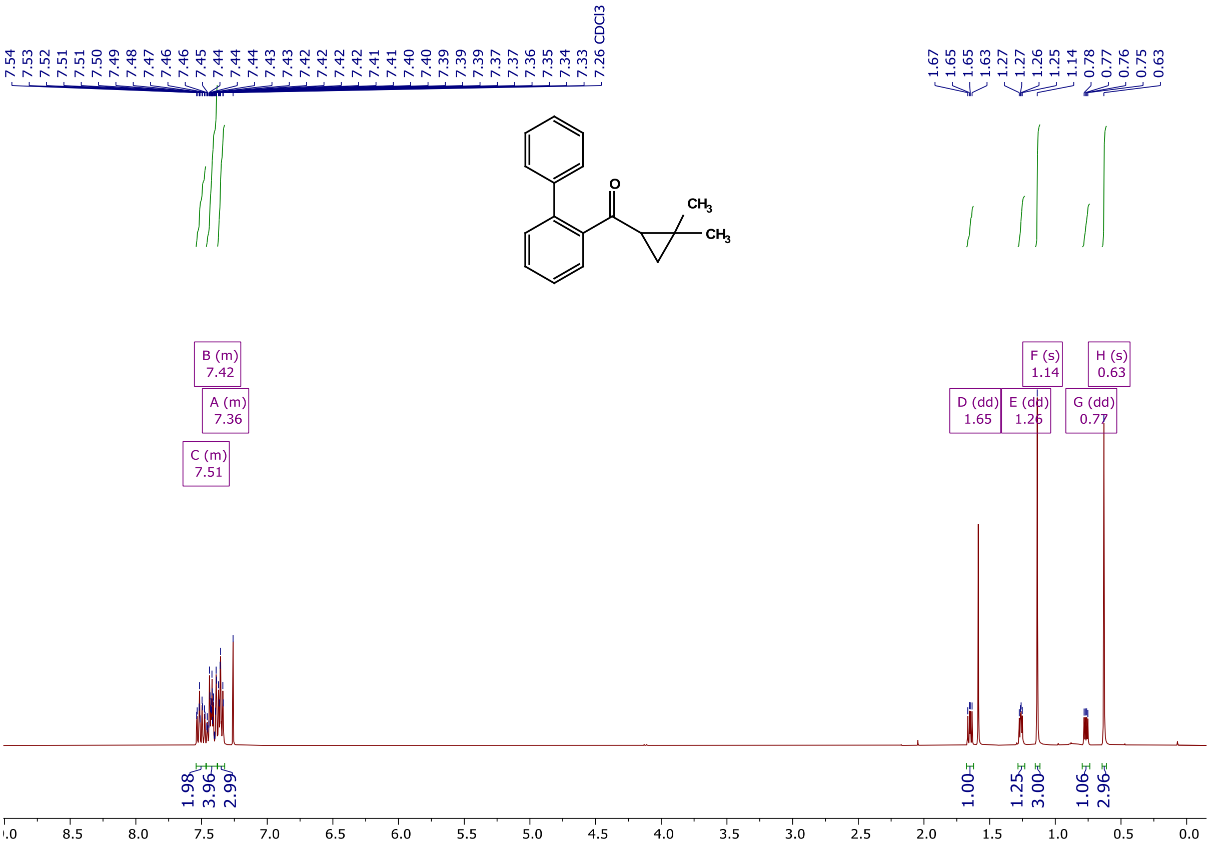

^{13}C NMR (101 MHz, Chloroform-*d*) (**1l**):

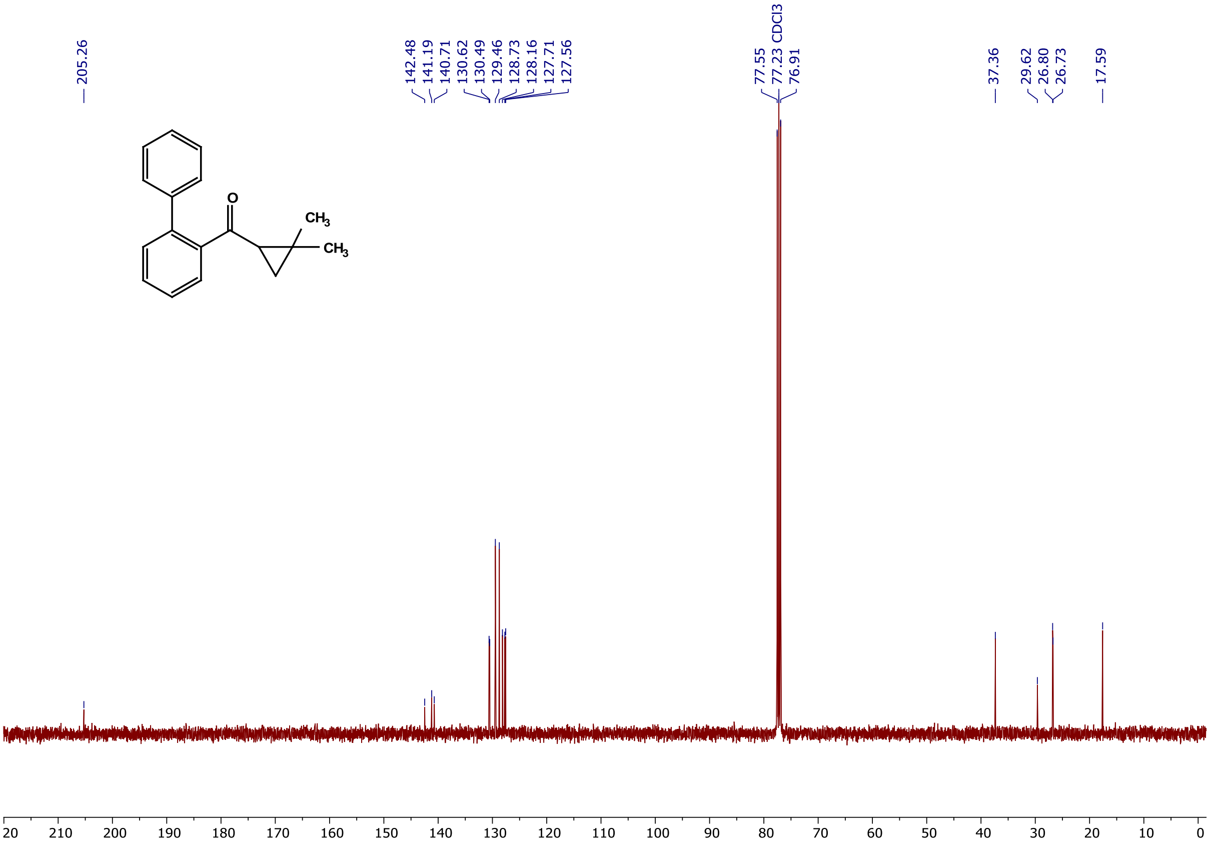

^{1}H NMR (400 MHz, Chloroform-*d*) (**1m**):

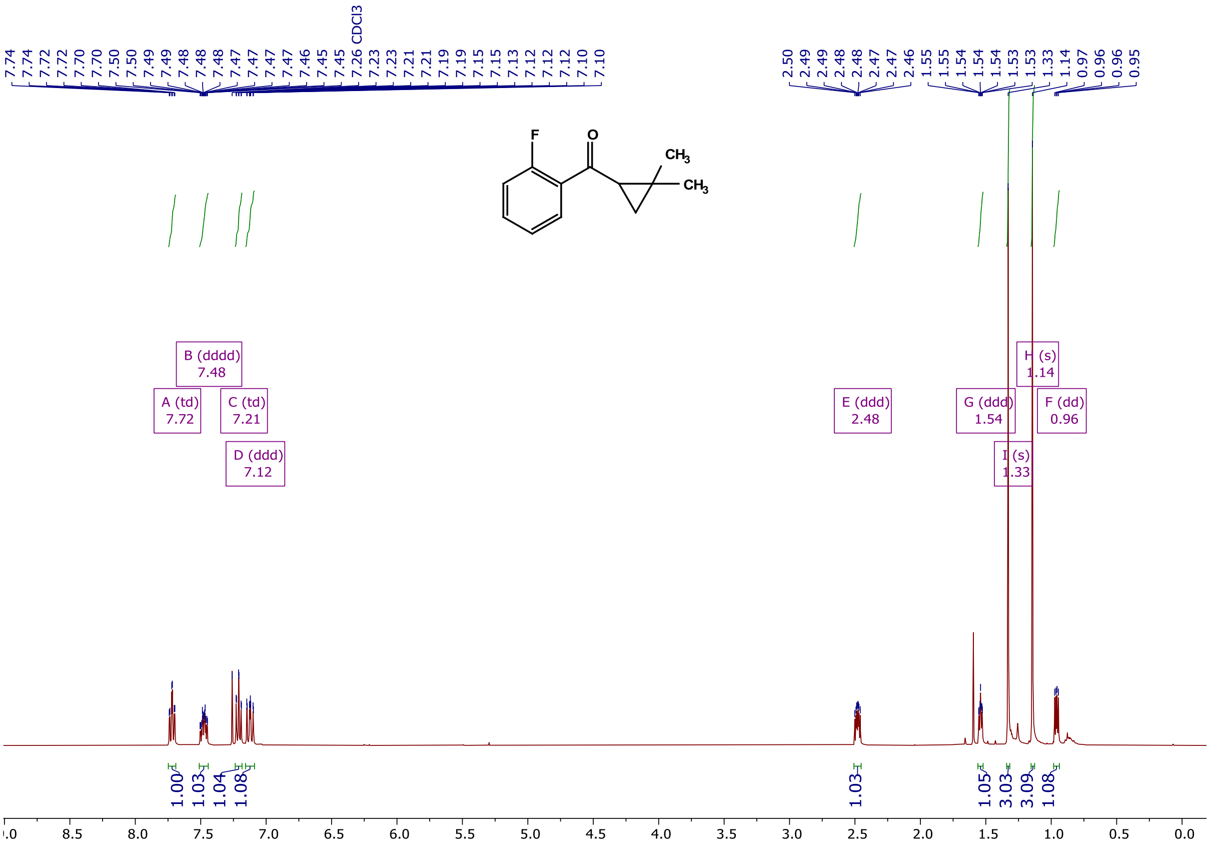

^{13}C NMR (101 MHz, Chloroform-*d*) (**1m**):

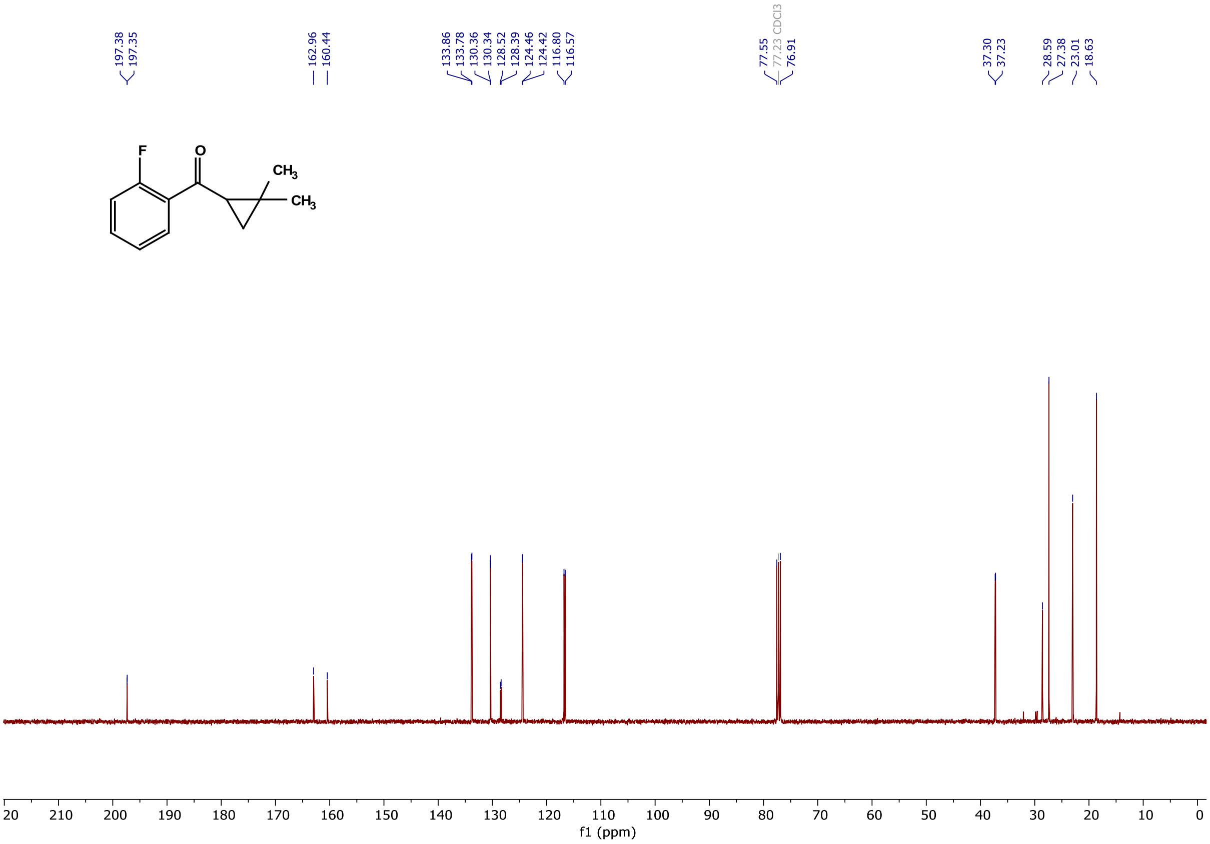

DEPT-135 NMR (101 MHz, Chloroform-*d*) (**1m**):

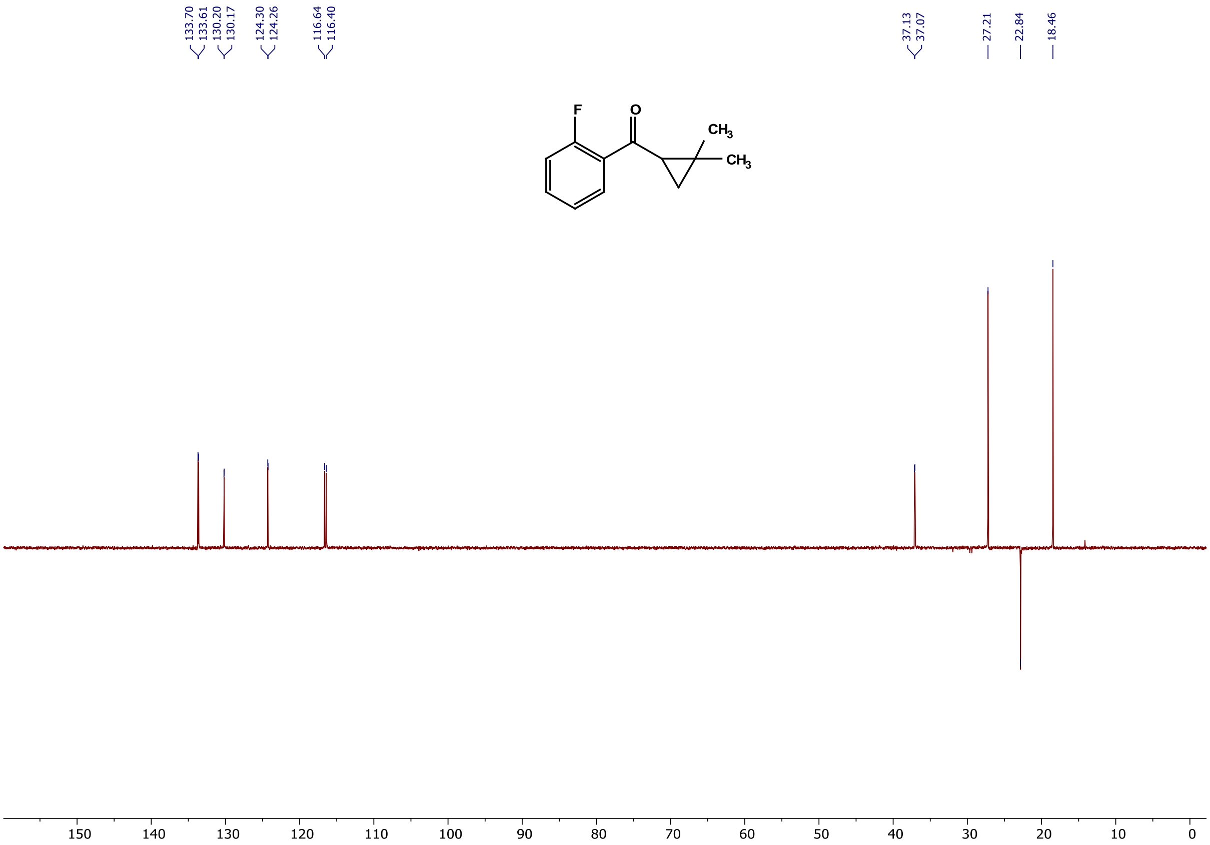

^{19}F NMR (376 MHz, Chloroform-*d*) (**1m**):

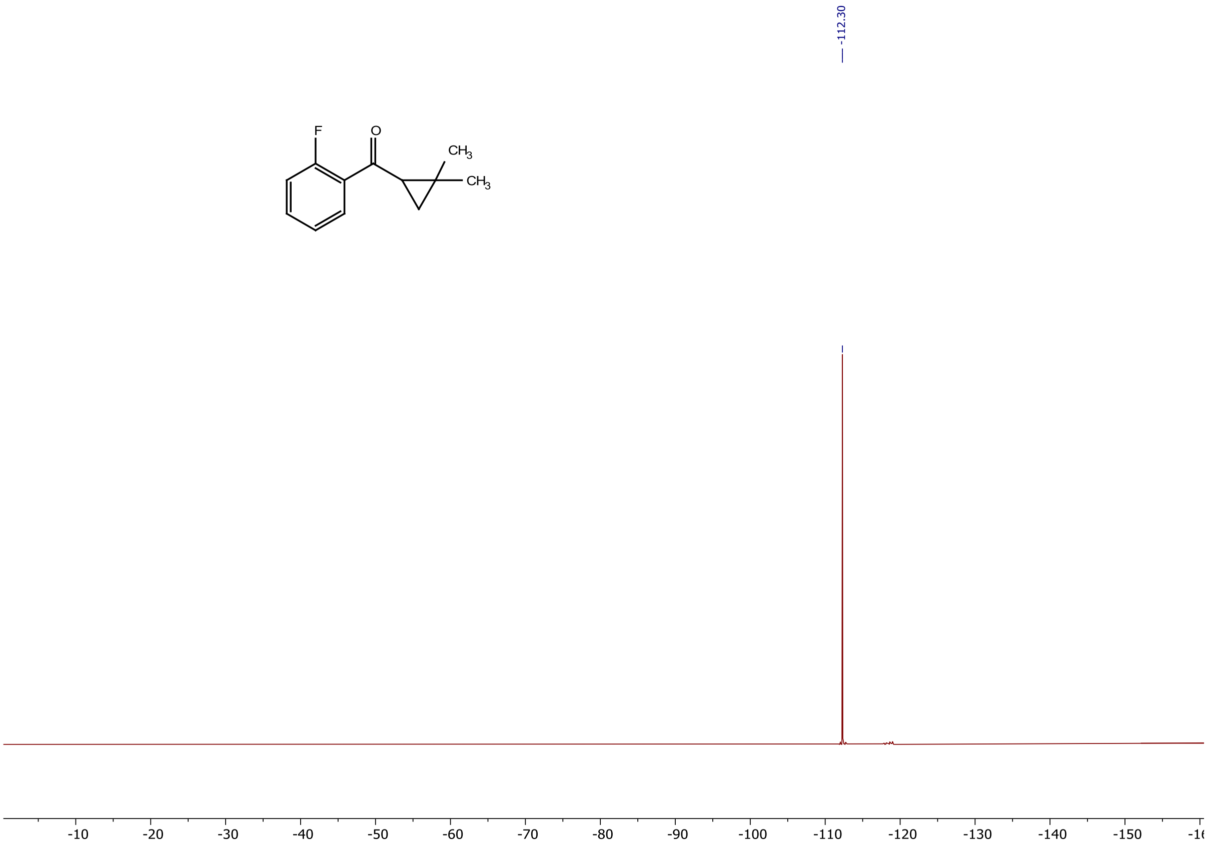

^{1}H NMR (400 MHz, Chloroform-*d*) (**1n**):

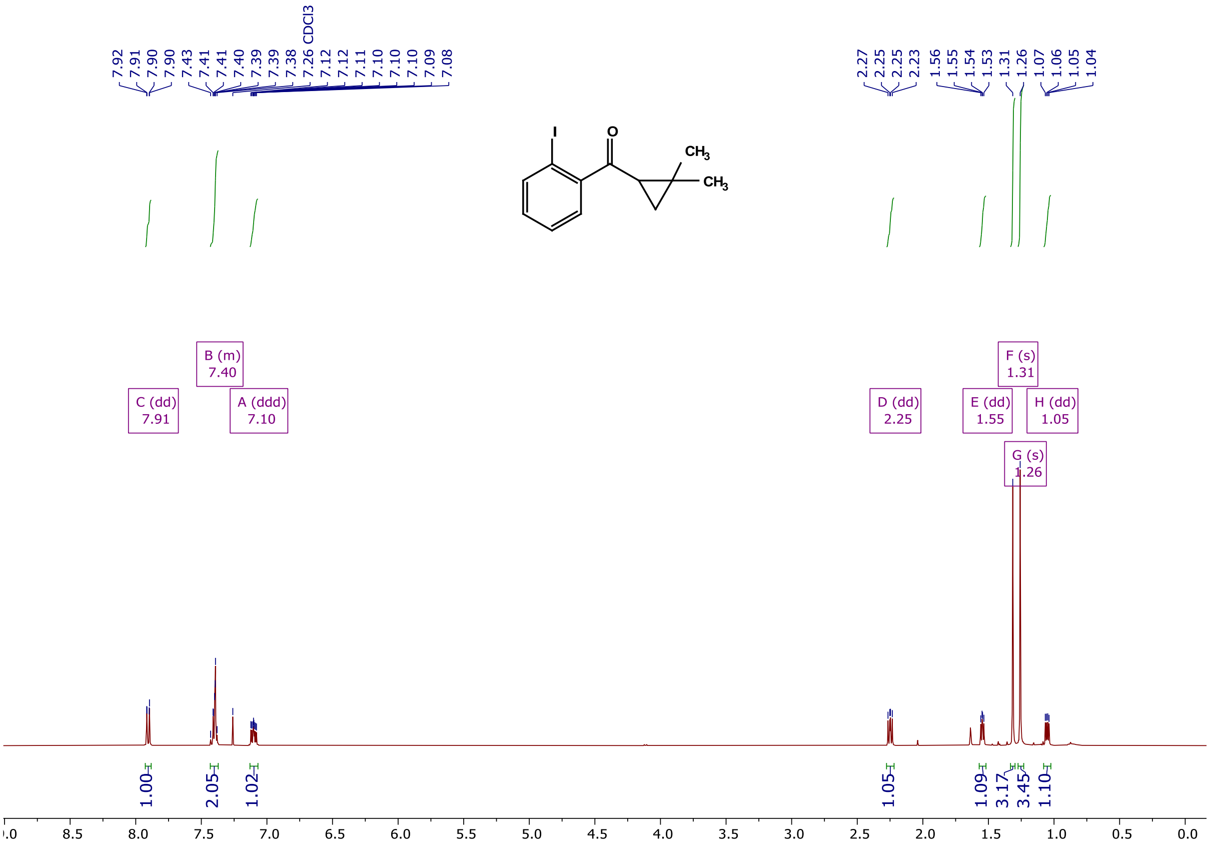

^{13}C NMR (101 MHz, Chloroform-*d*) (**1n**):

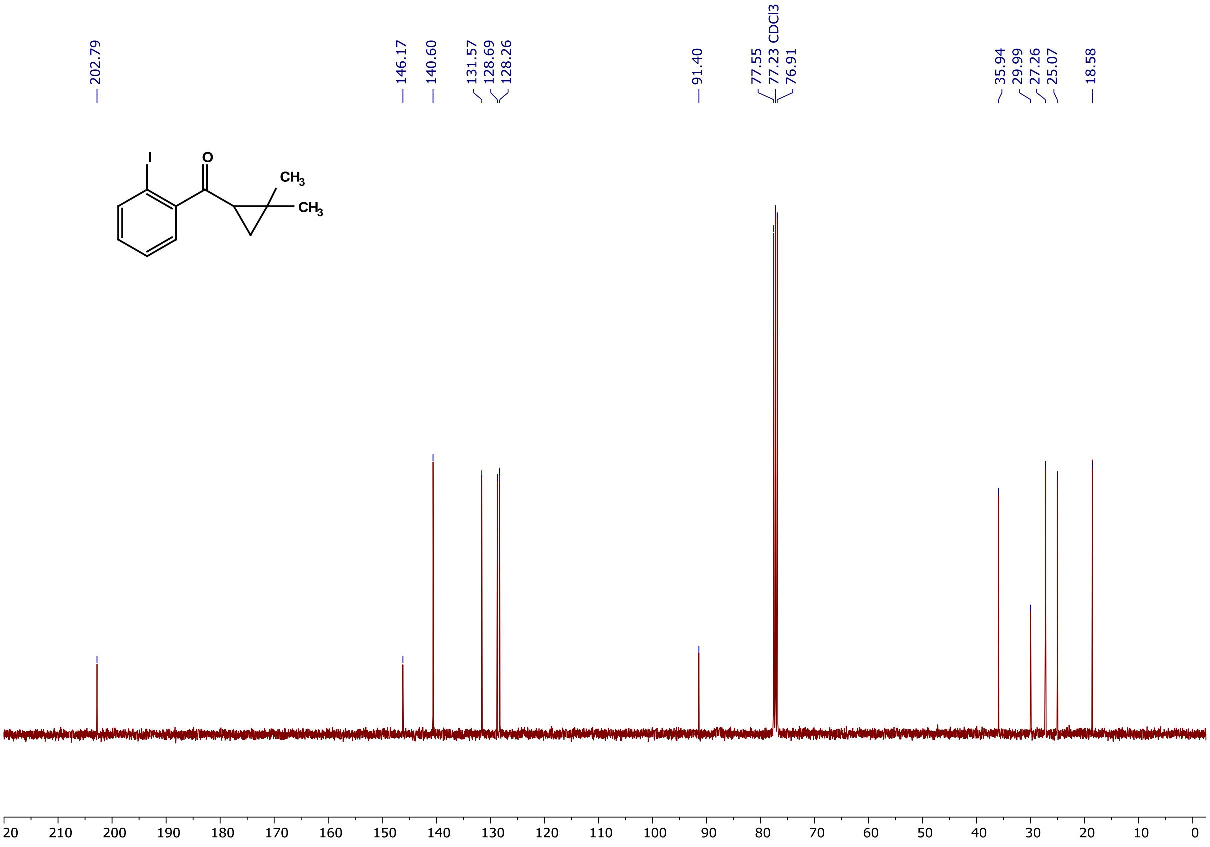

^{1}H NMR (400 MHz, Chloroform-*d*) (**1o**):

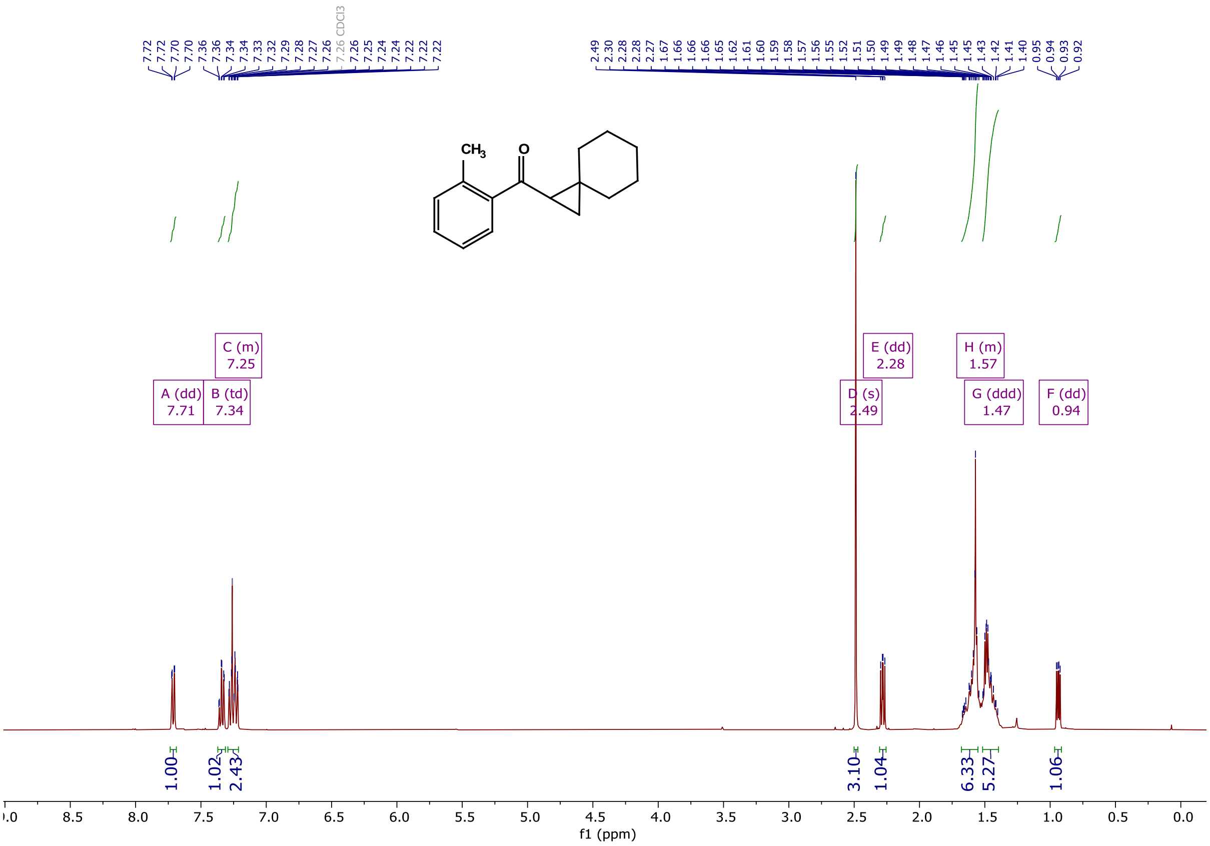

^{13}C NMR (101 MHz, Chloroform-*d*) (**1o**):

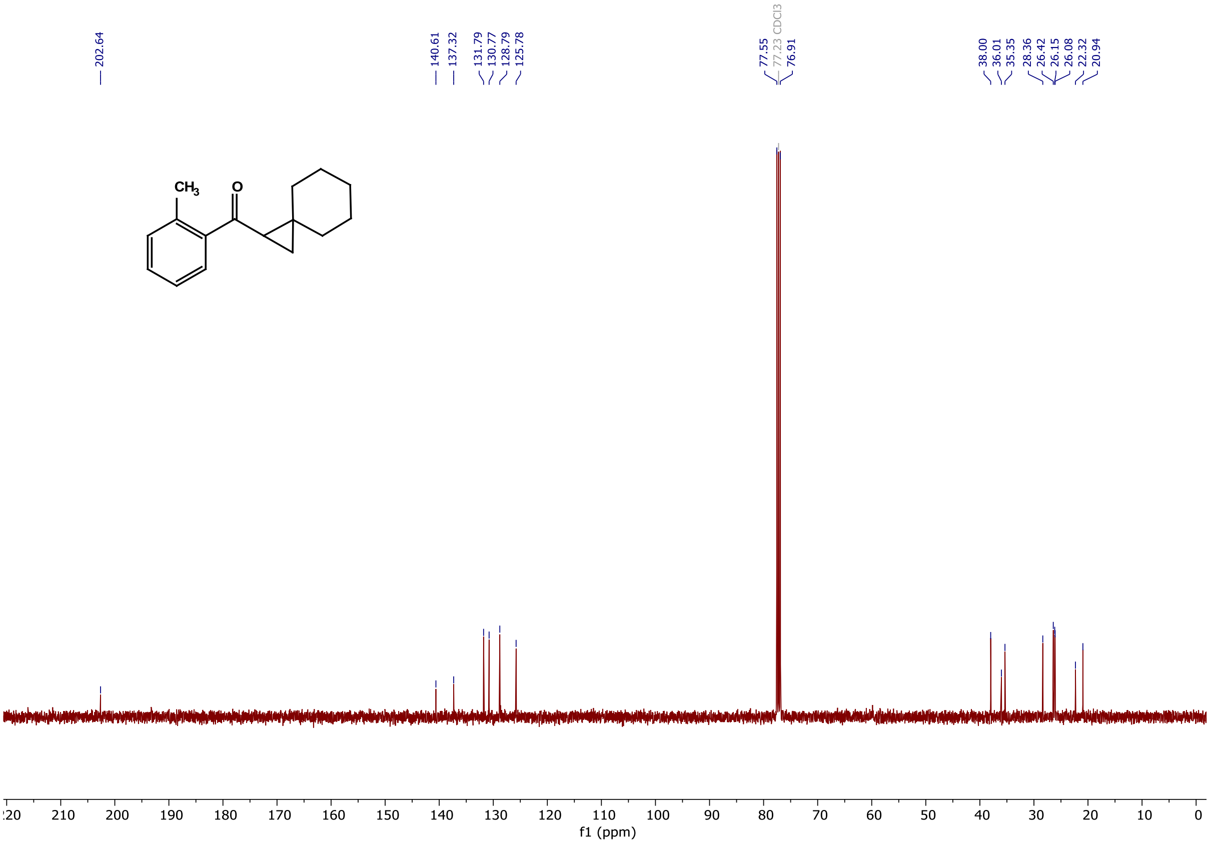

DEPT-135 NMR (101 MHz, Chloroform-*d*) (**1o**):

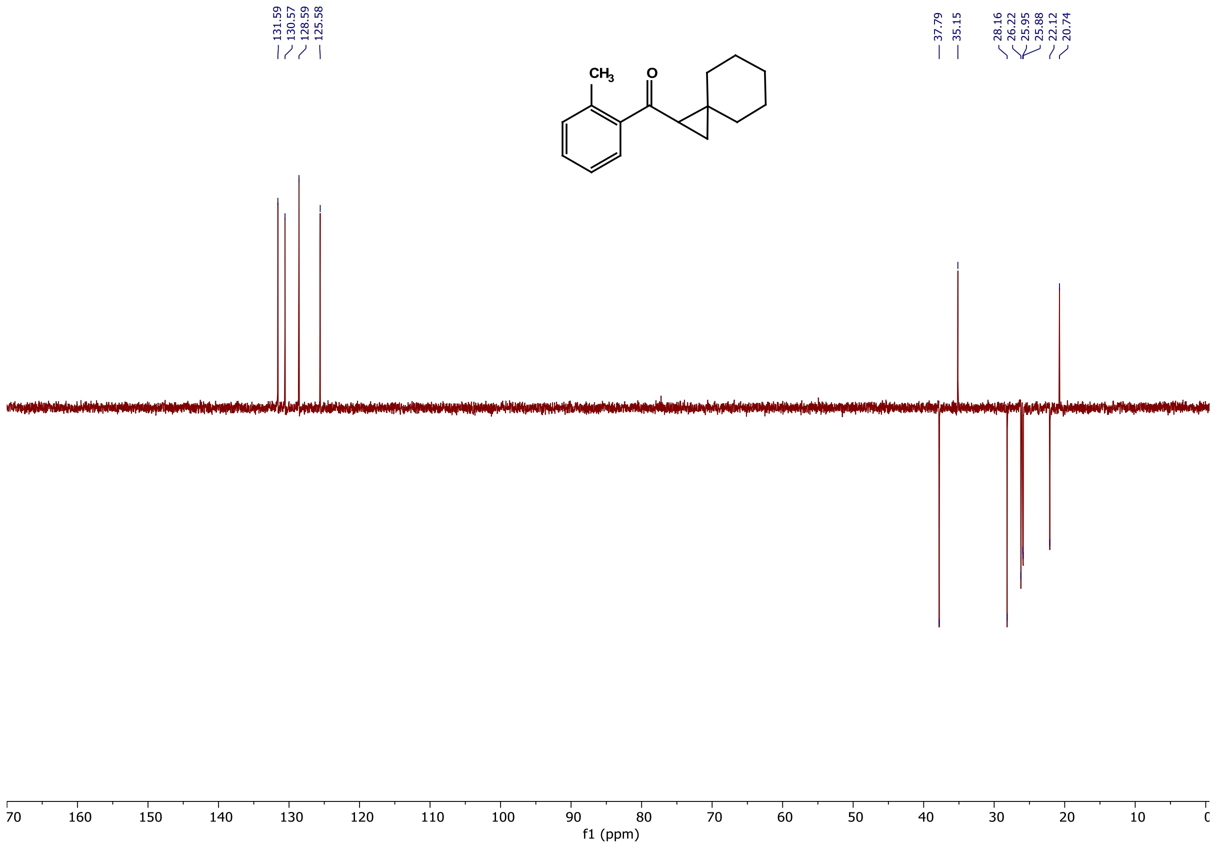

^{1}H NMR (500 MHz, Chloroform-*d*) (**1p**):

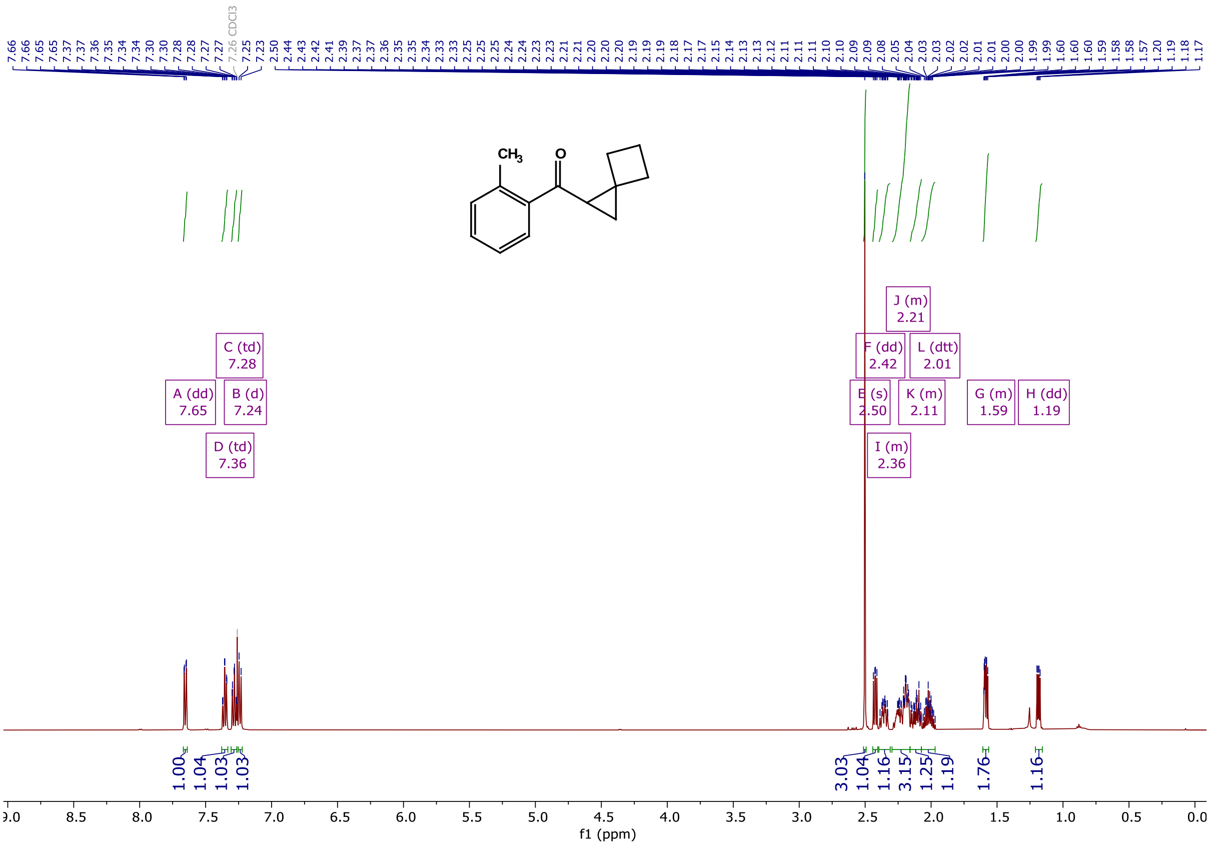

^{13}C NMR (126 MHz, Chloroform-*d*) (**1p**):

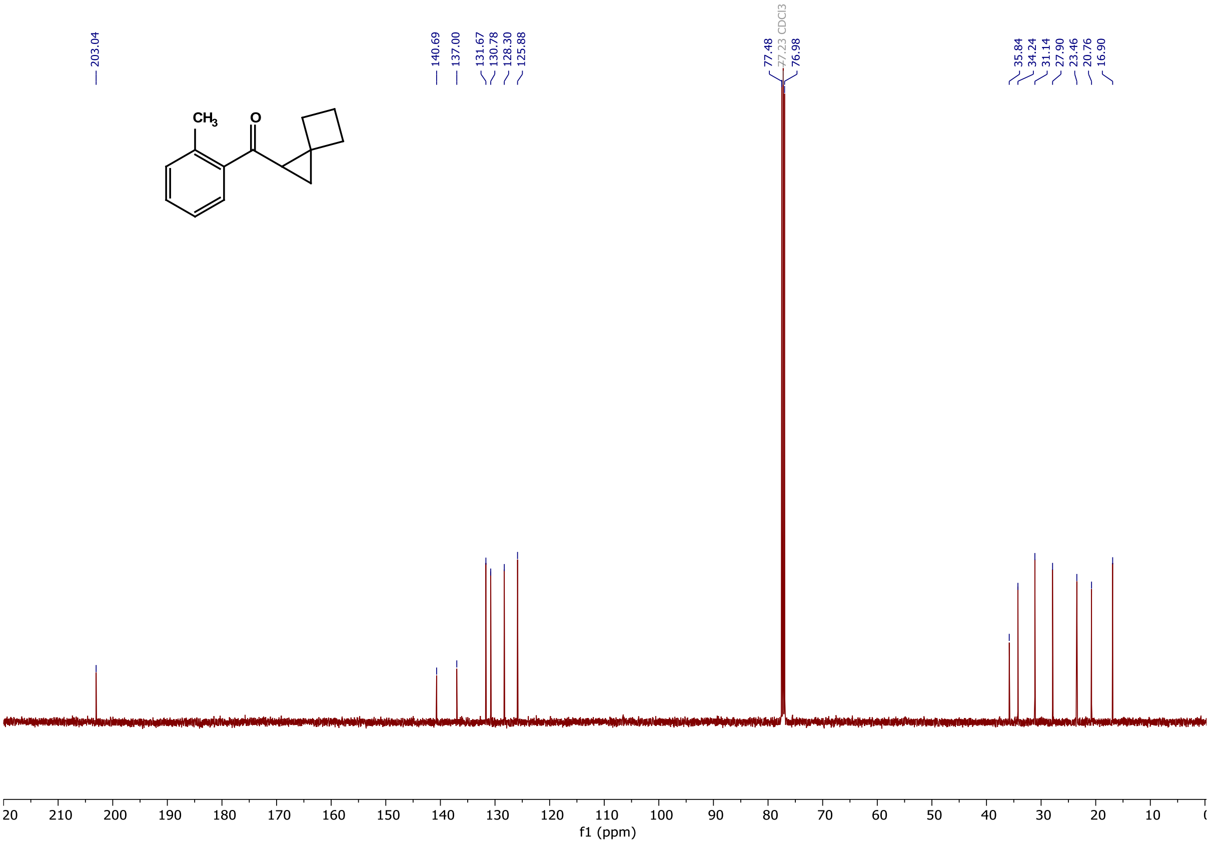

DEPT-135 NMR (126 MHz, Chloroform-*d*) (**1p**):

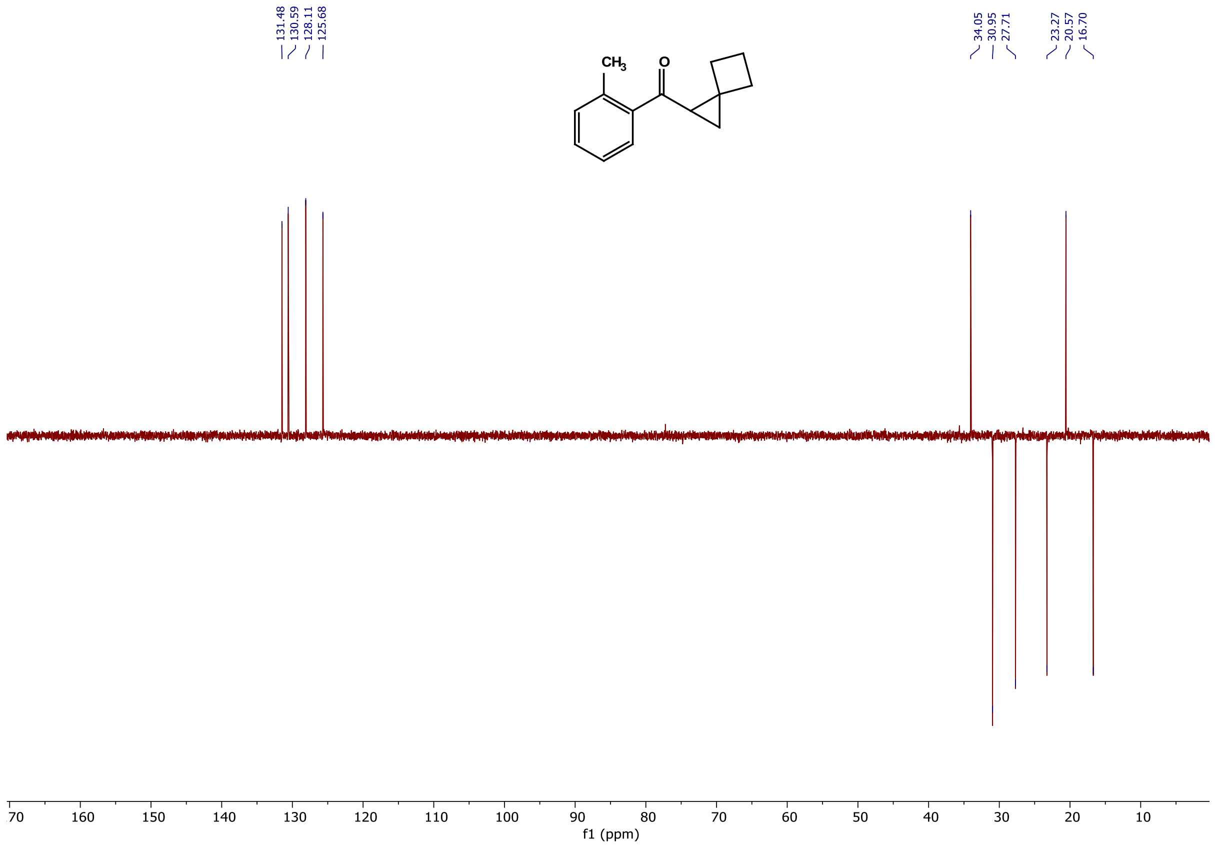

^{1}H NMR (400 MHz, Chloroform-*d*) (**1q**):

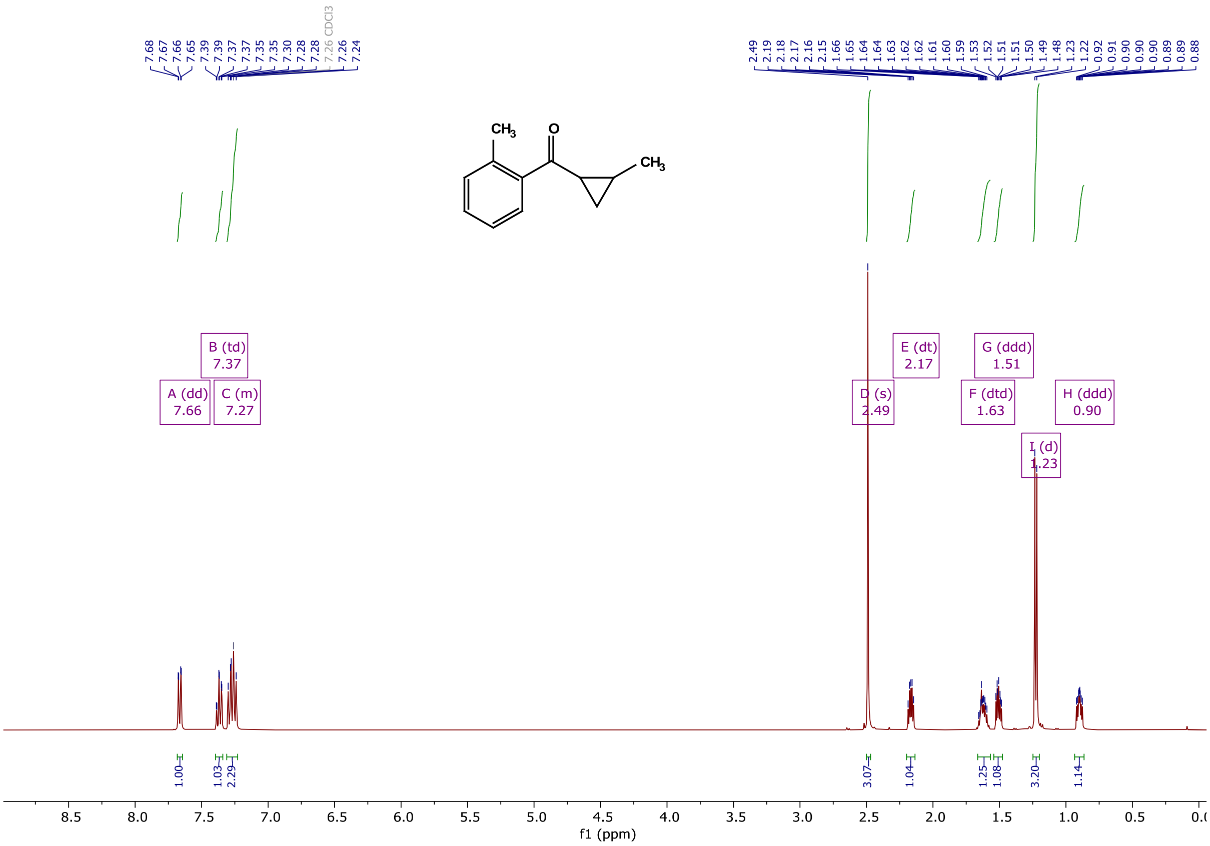

^{13}C NMR (101 MHz, Chloroform-*d*) (**1q**):

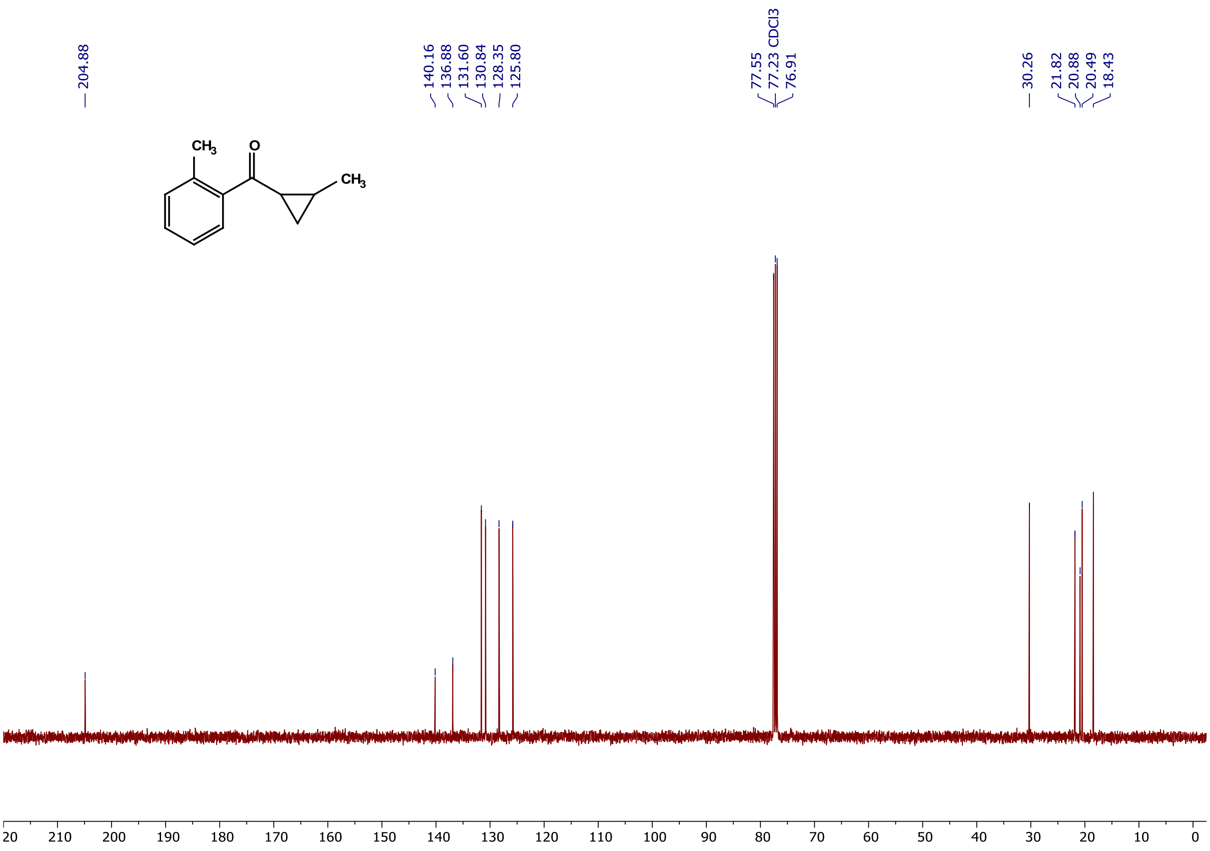

DEPT-135 NMR (101 MHz, Chloroform-*d*) (**1q**):

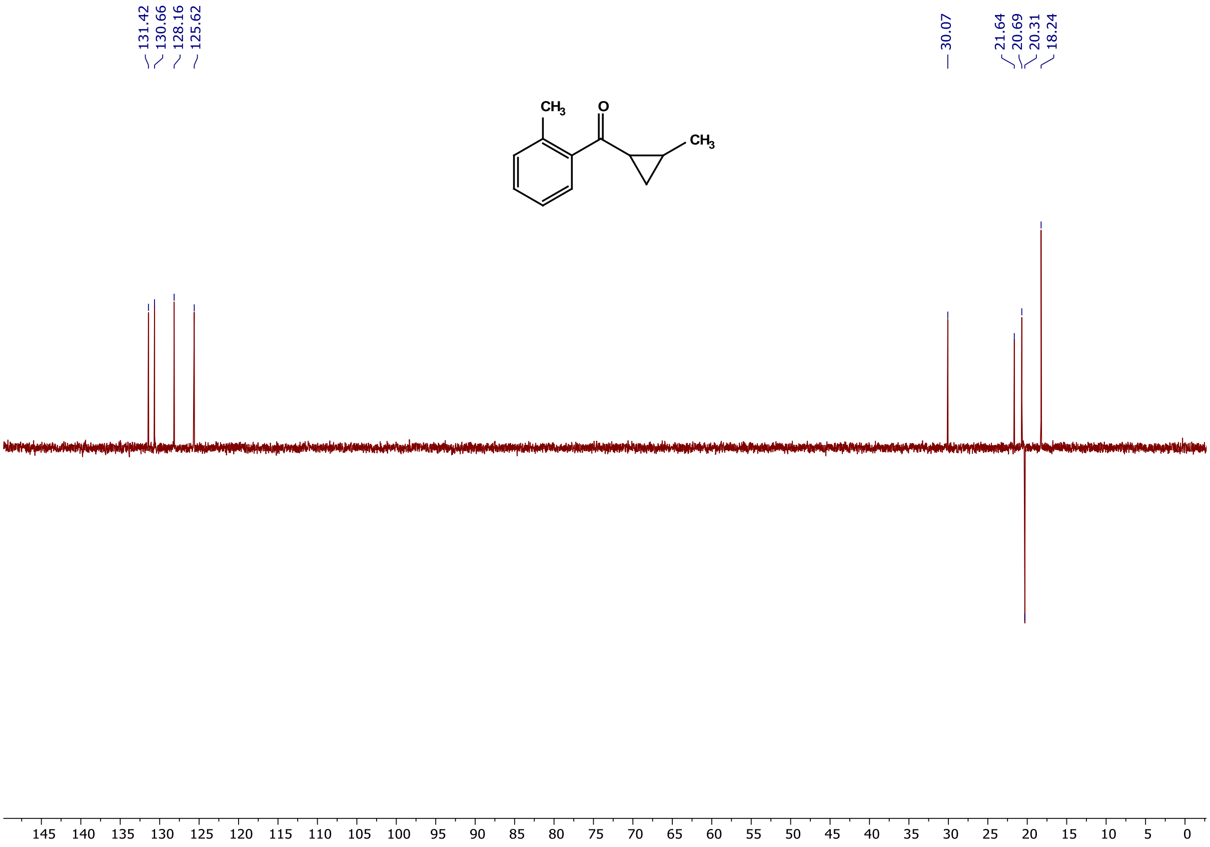

^{1}H NMR (400 MHz, Chloroform-*d*) (**1r**):

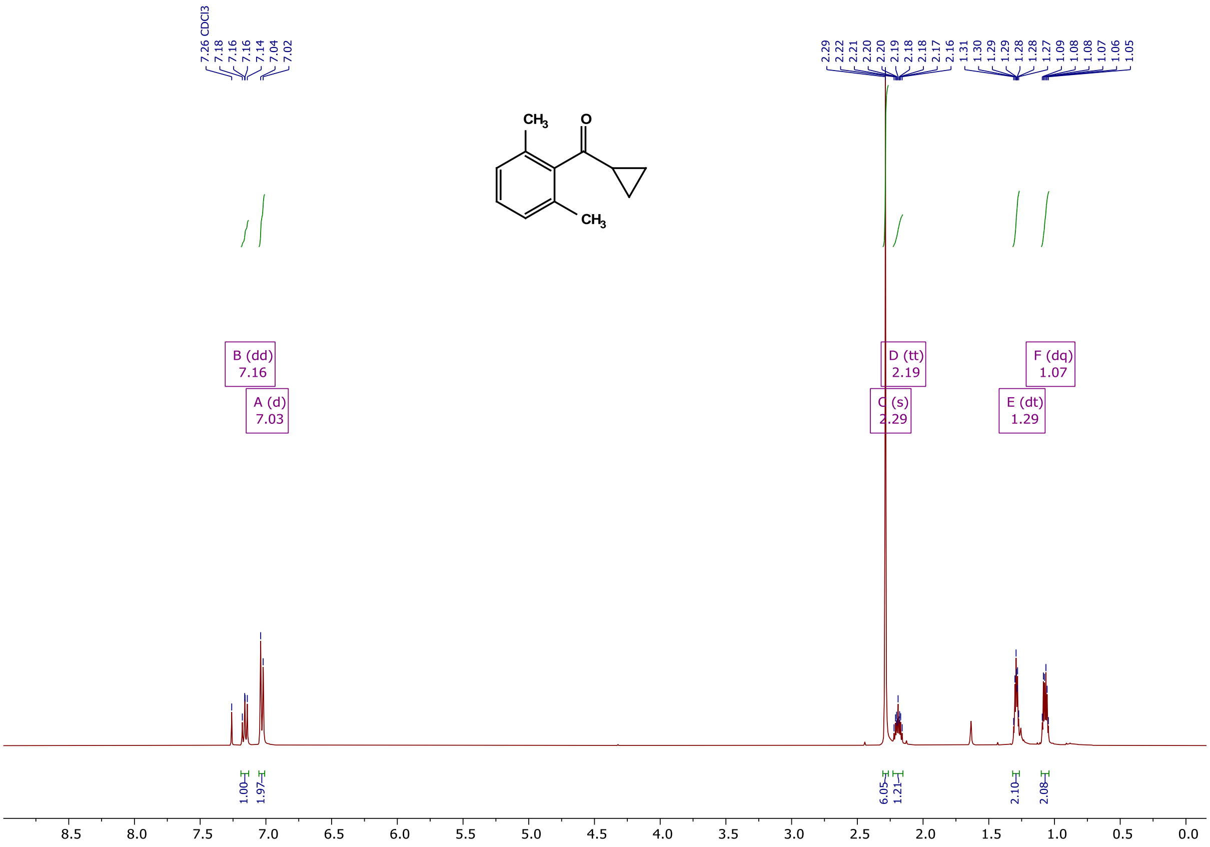

^{13}C NMR (101 MHz, Chloroform-*d*) (**1r**):

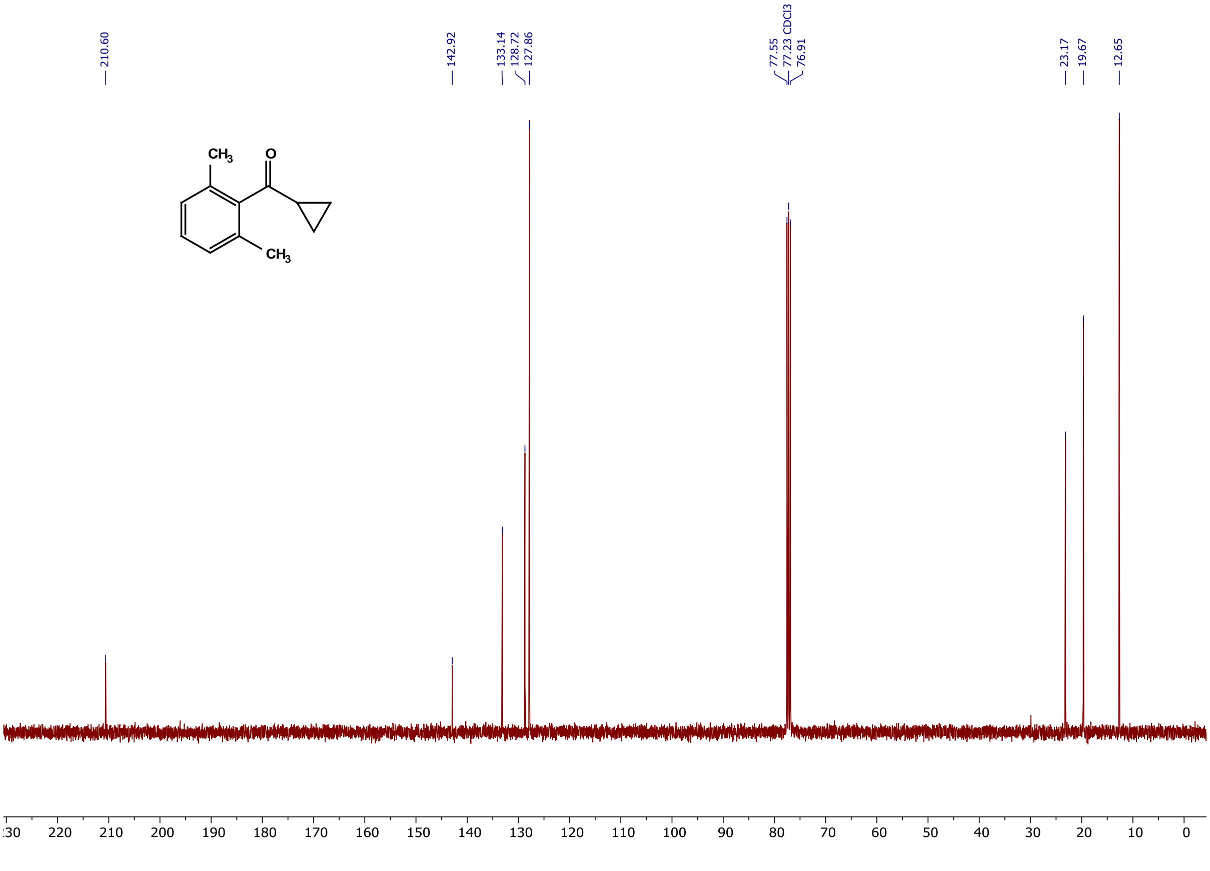

DEPT-135 NMR (101 MHz, Chloroform-*d*) (**1r**):

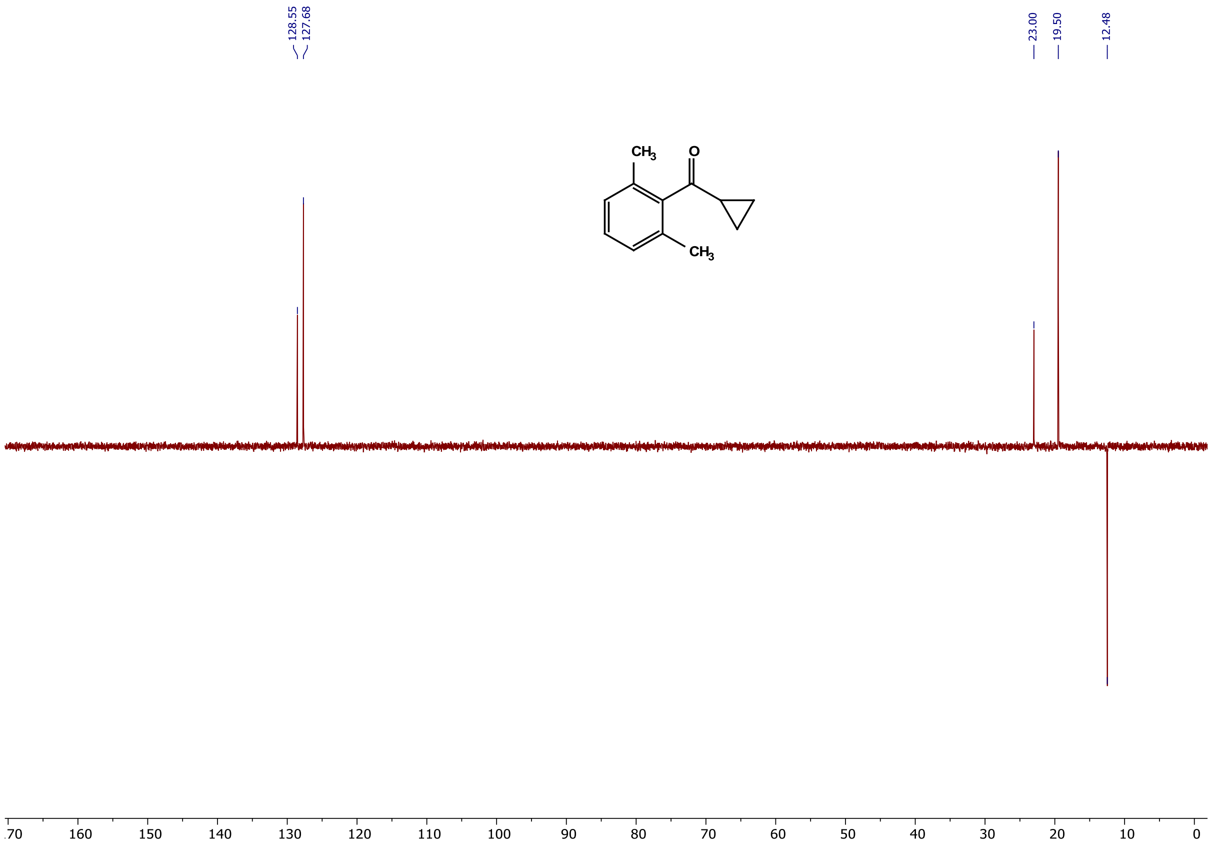

^{1}H NMR (400 MHz, Chloroform-*d*) (**1s**):

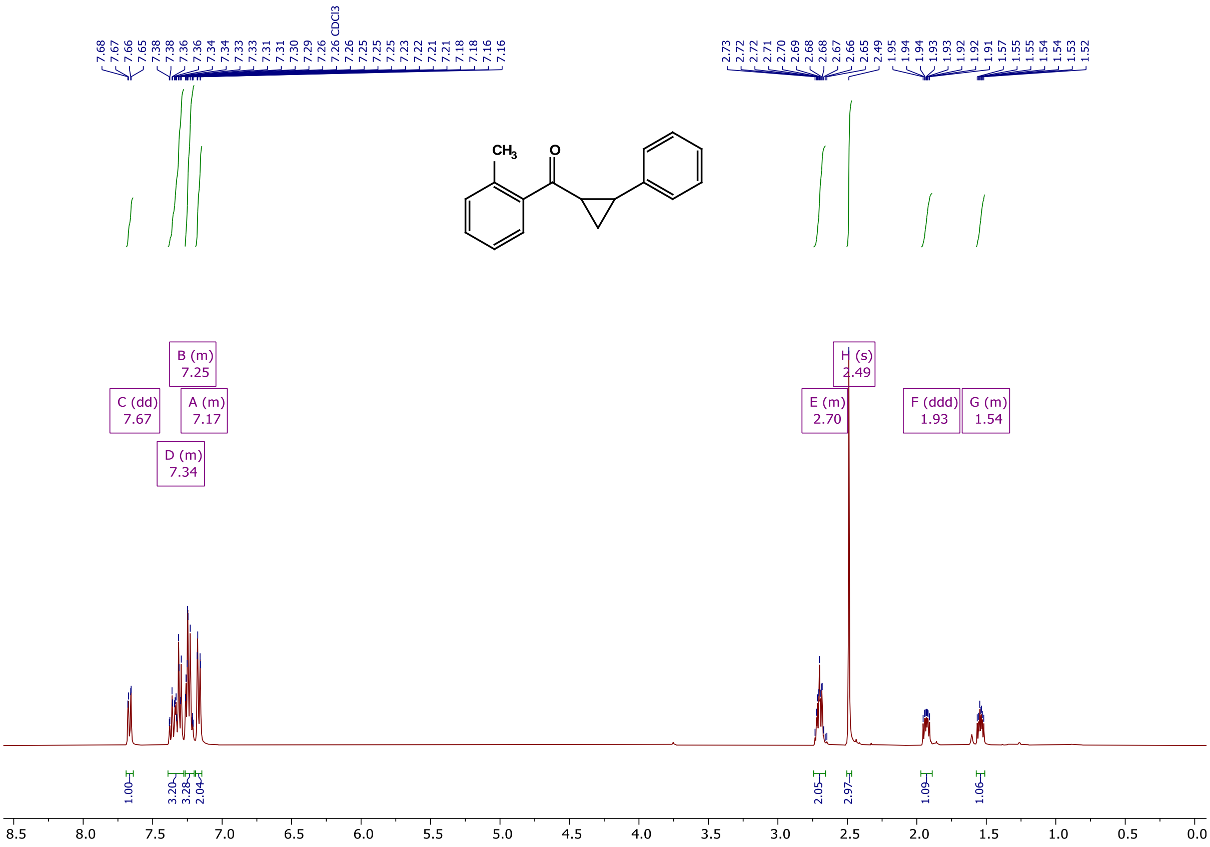

^{13}C NMR (101 MHz, Chloroform-*d*) (**1s**):

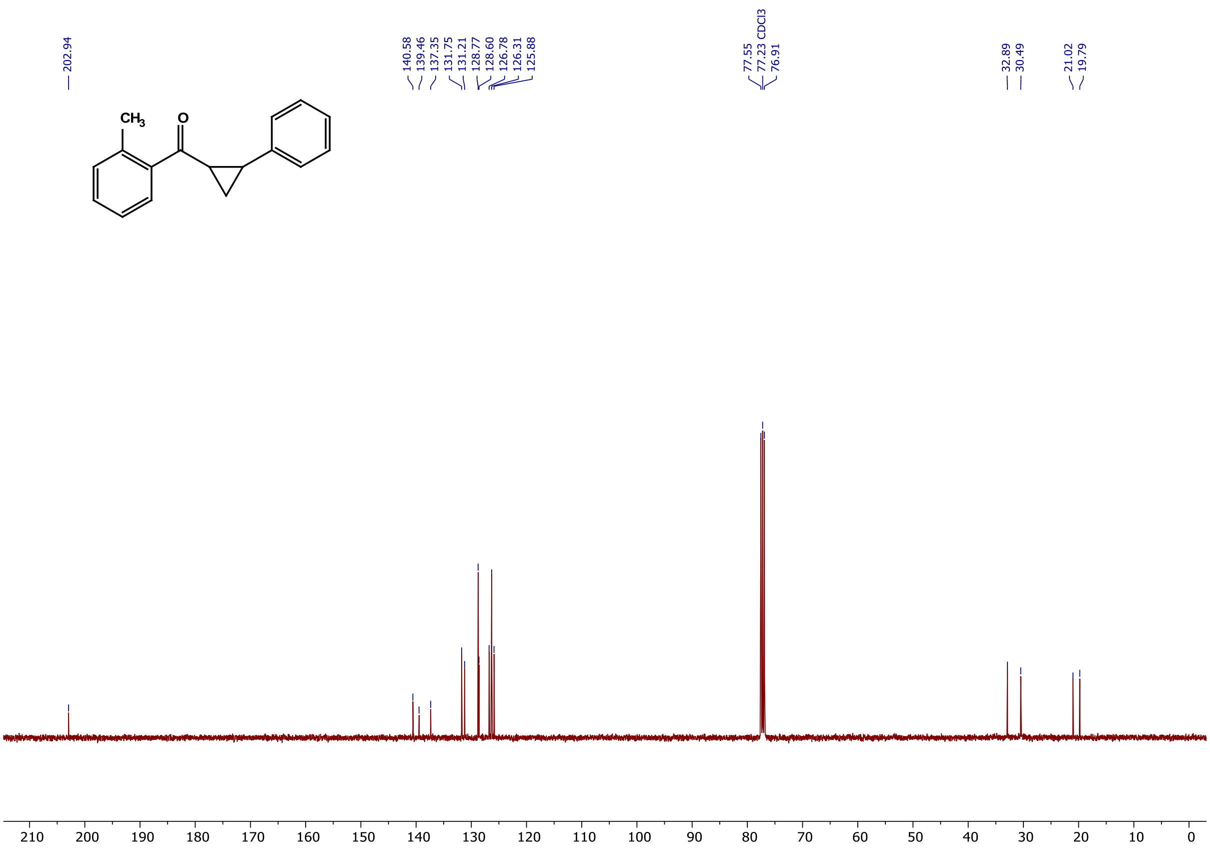

DEPT-135 NMR (101 MHz, Chloroform-*d*) (**1s**):

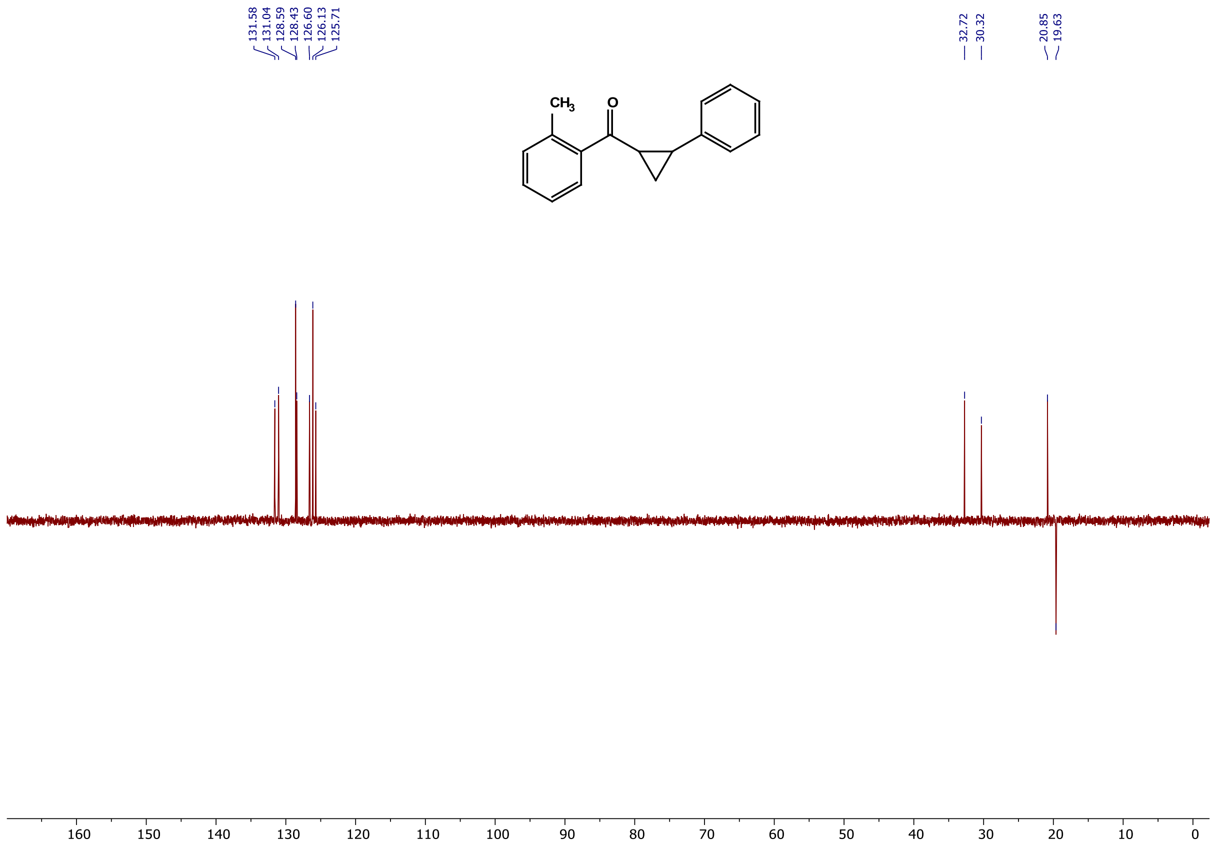

^{1}H NMR (400 MHz, Chloroform-*d*) (**1t**):

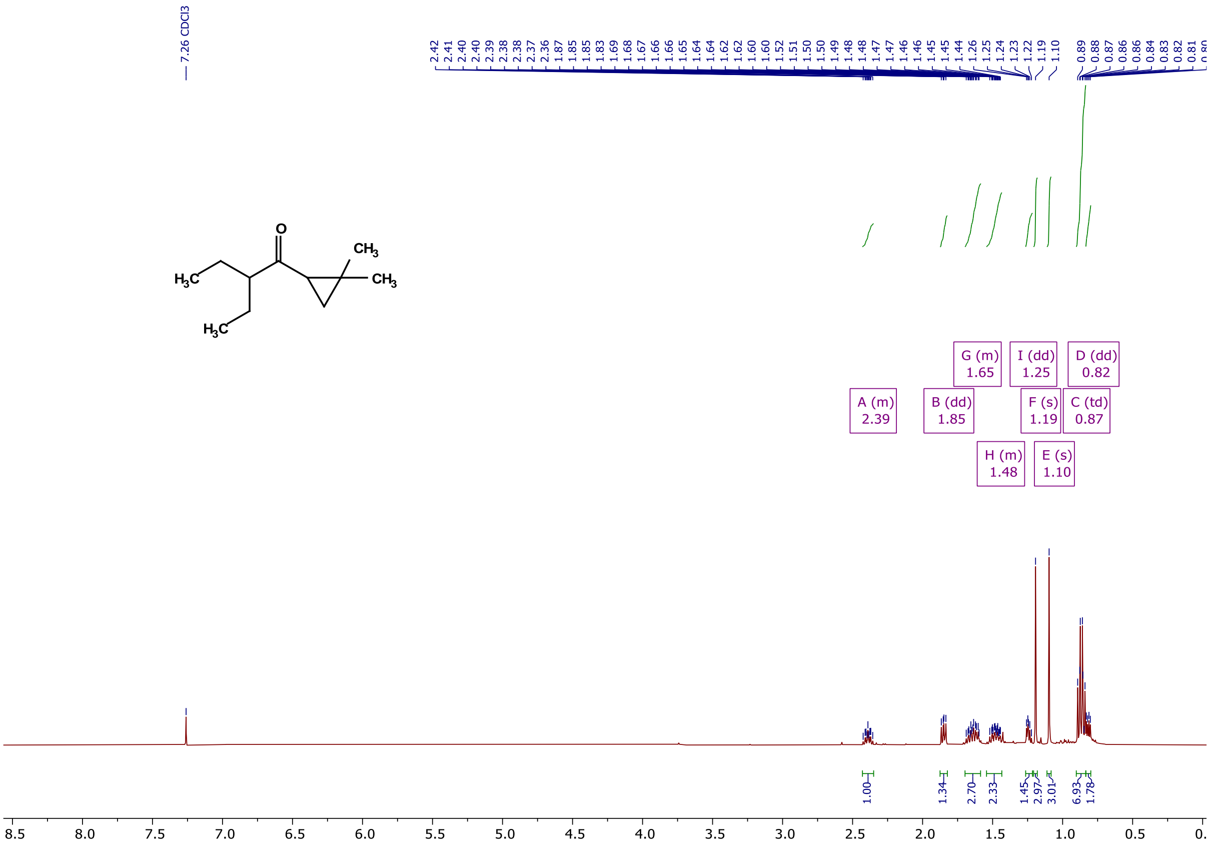

^{13}C NMR (101 MHz, Chloroform-*d*) (**1t**):

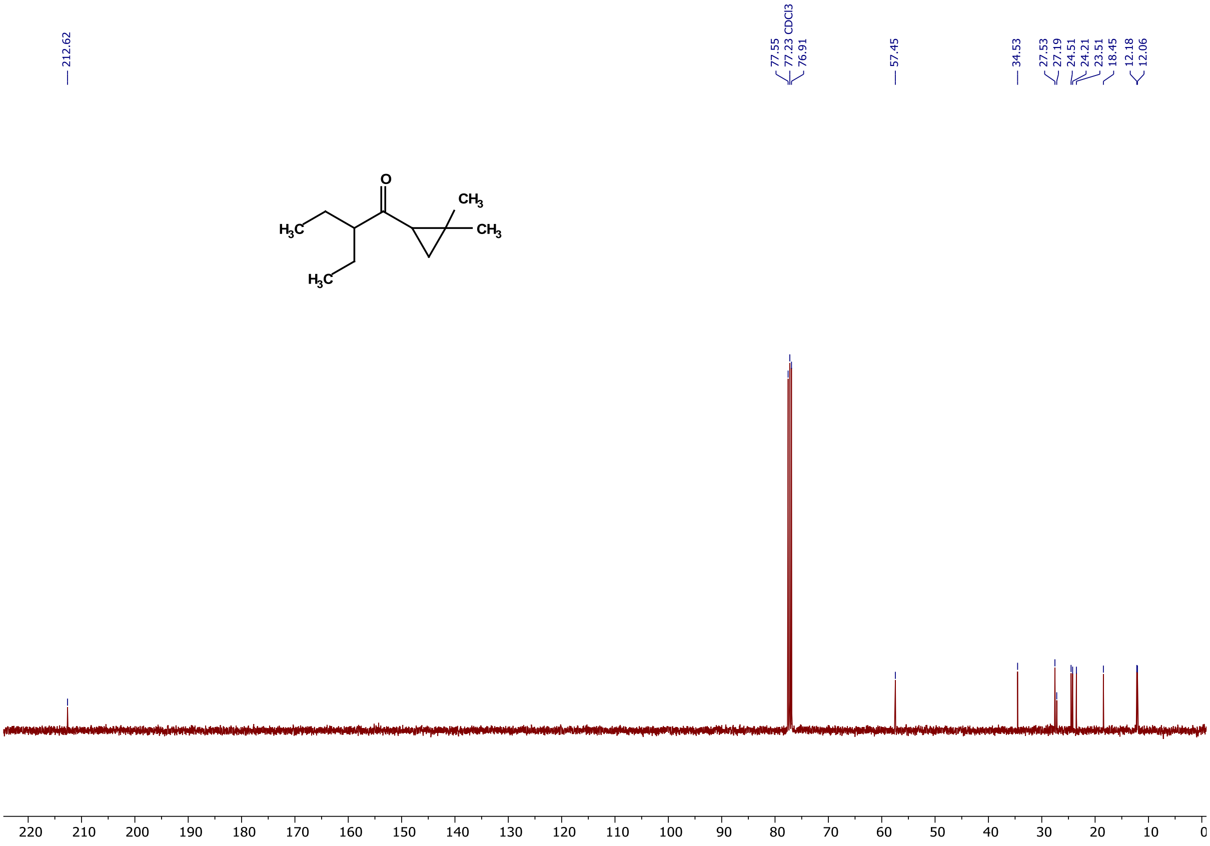

DEPT-135 NMR (101 MHz, Chloroform-*d*) (**1t**):

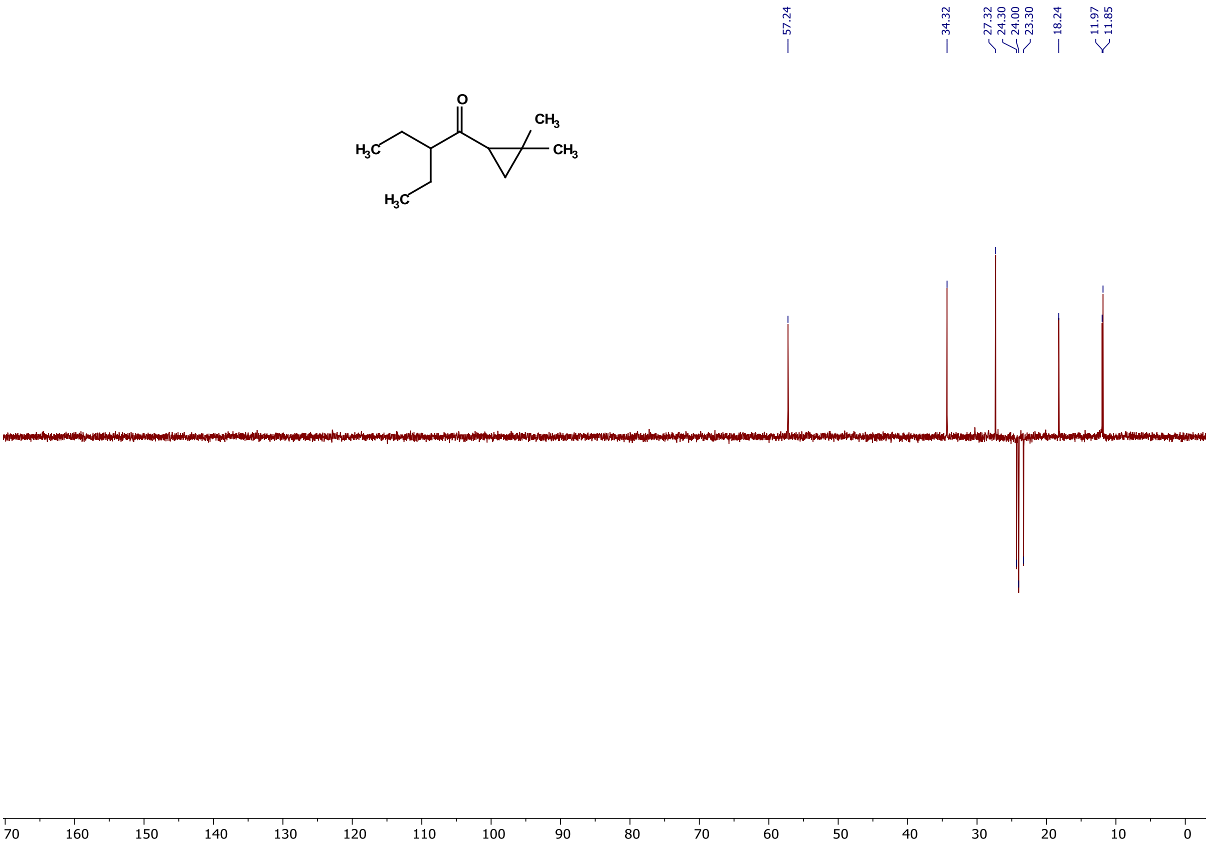

^{1}H NMR (400 MHz, Chloroform-*d*) (**1u**):

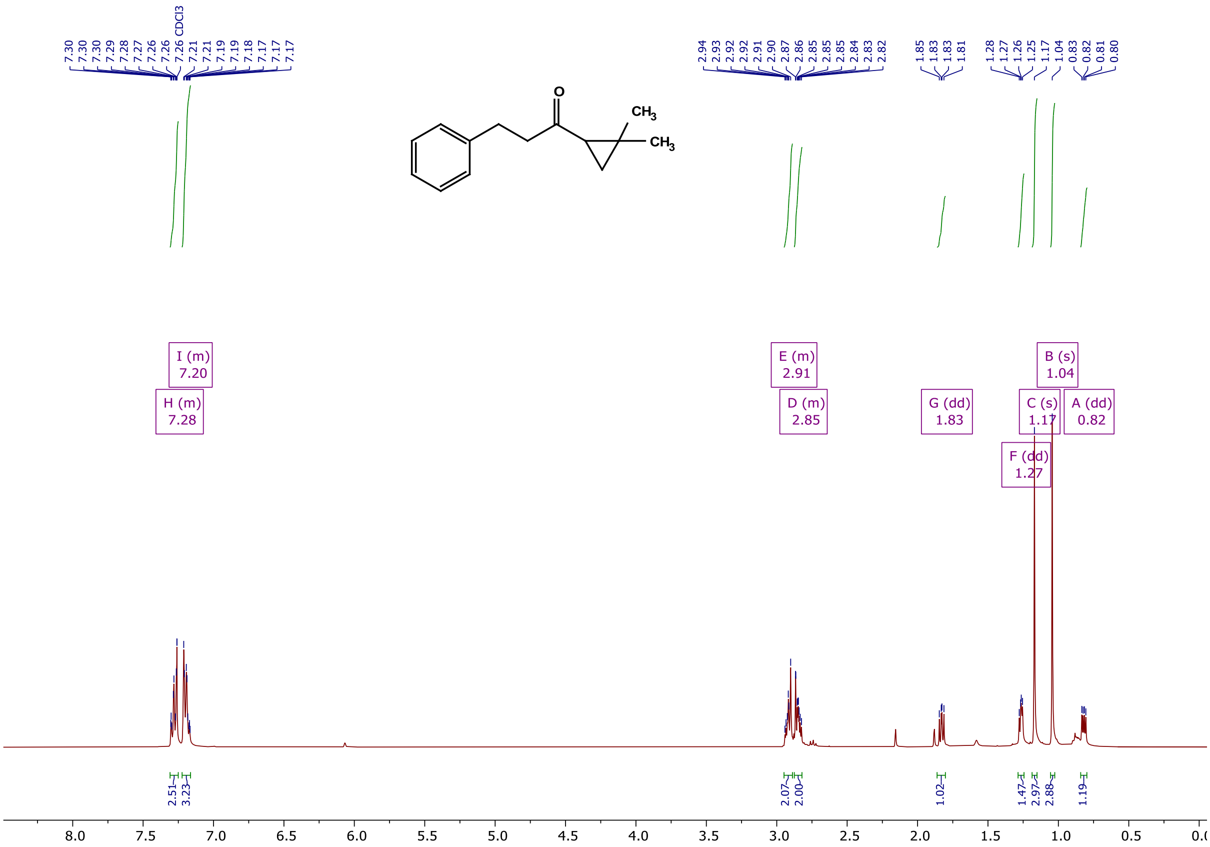

^{13}C NMR (101 MHz, Chloroform-*d*) (**1u**):

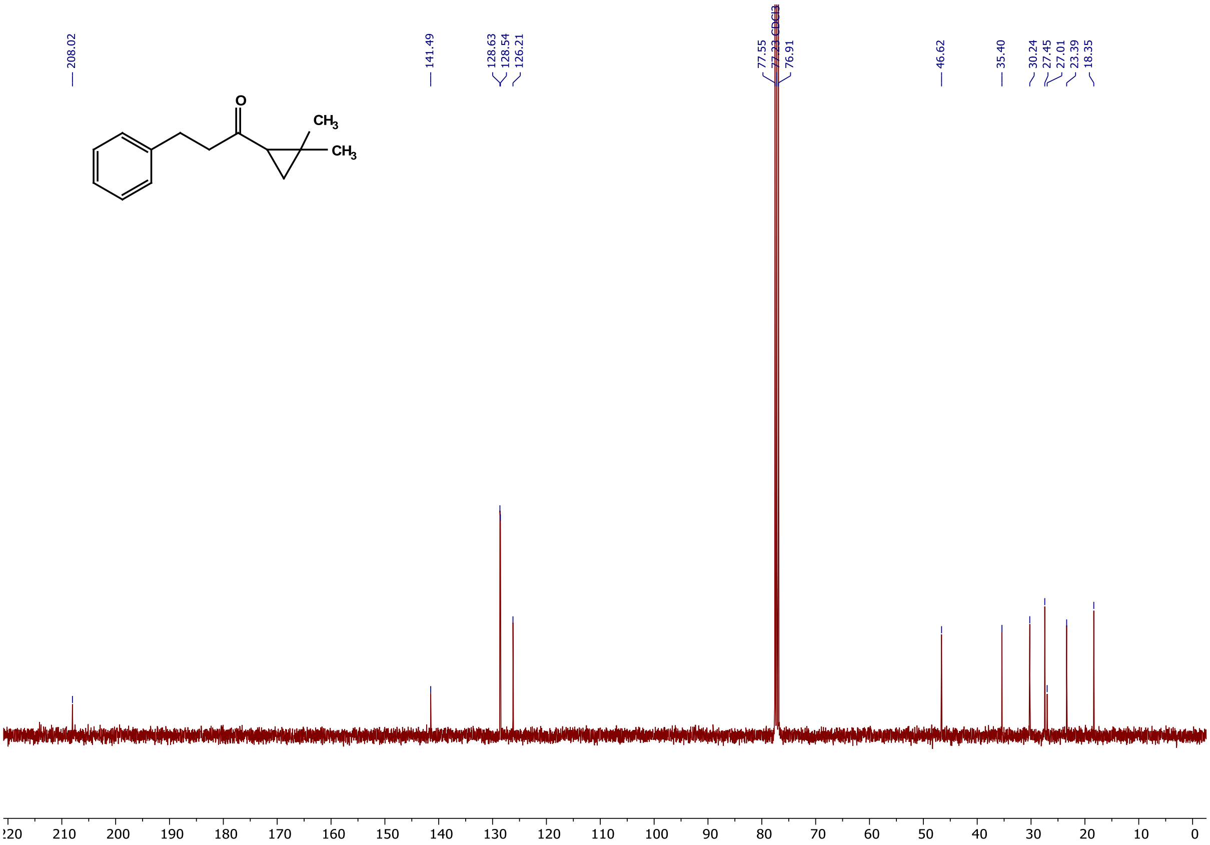

DEPT-135 NMR (101 MHz, Chloroform-*d*) (**1u**):

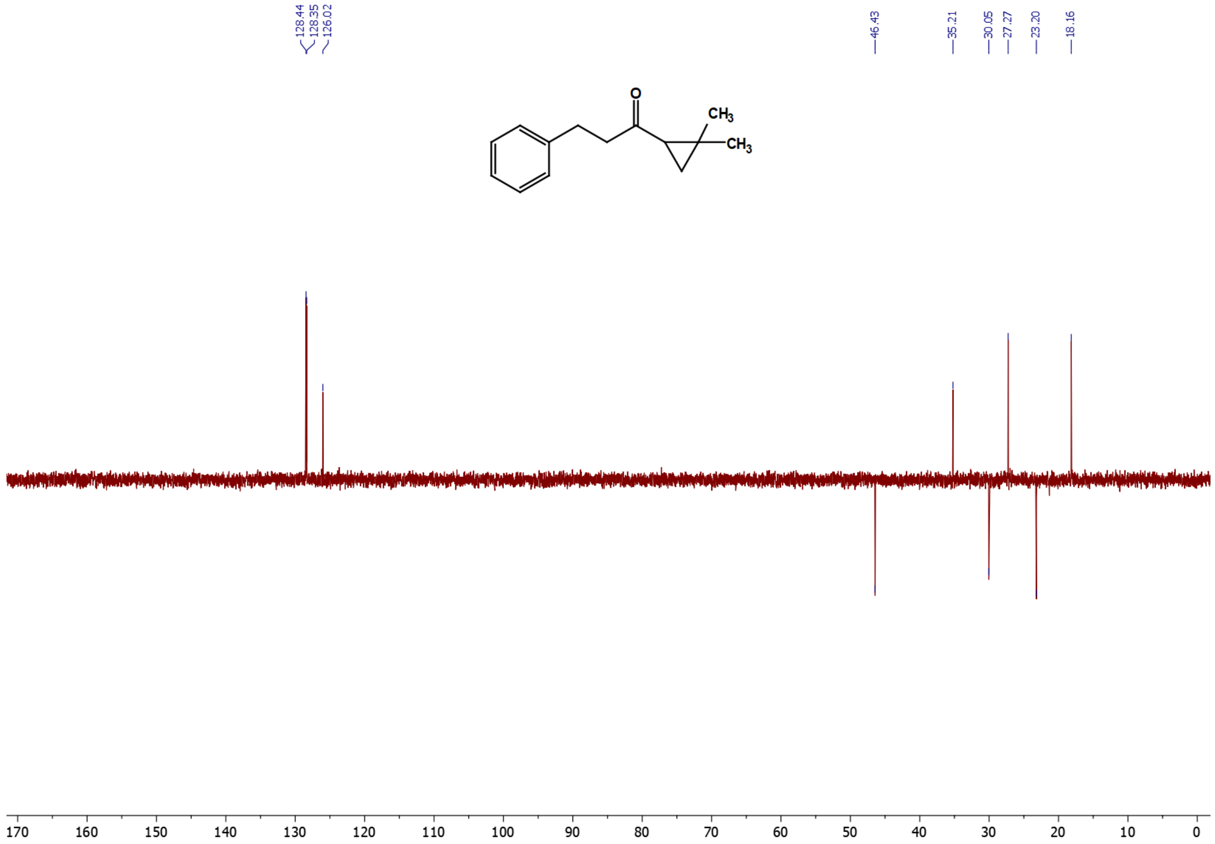

^{1}H NMR (400 MHz, Chloroform-*d*) (**1v**):

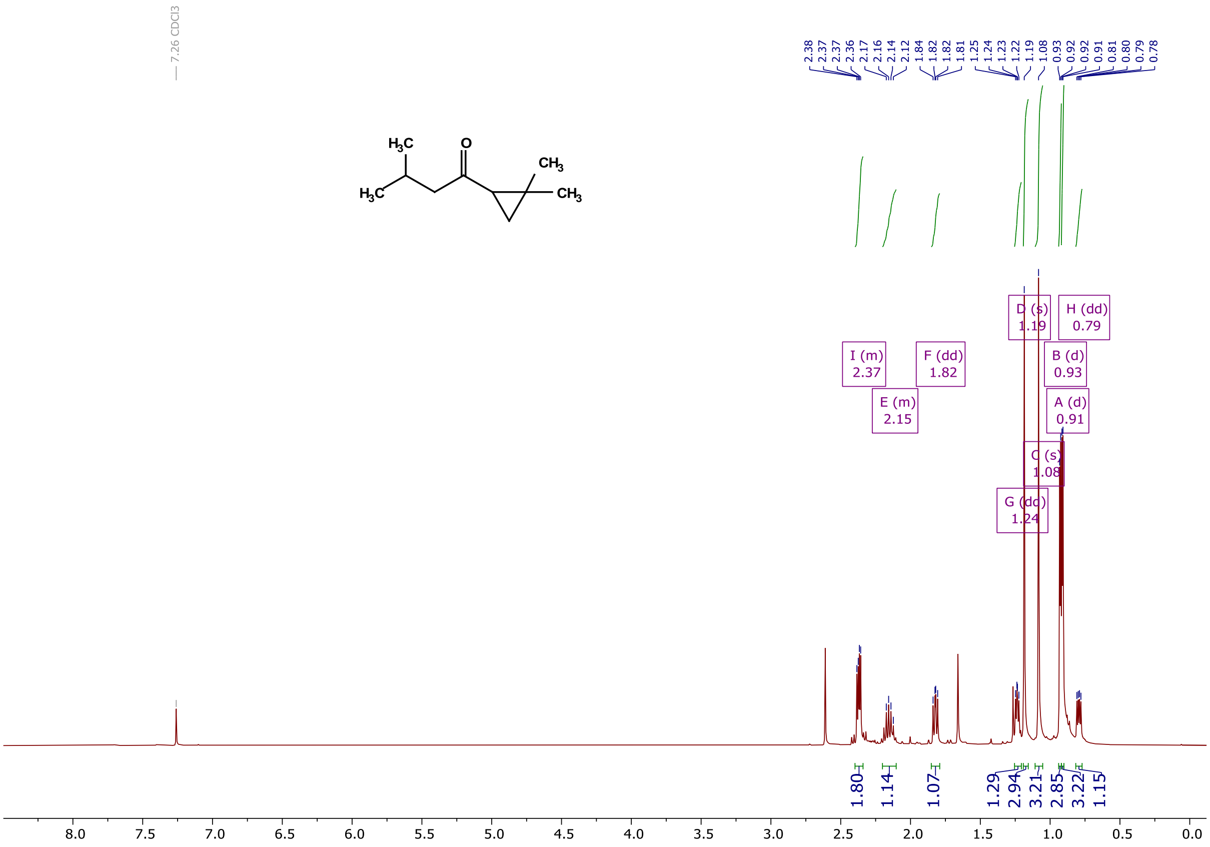

^{13}C NMR (101 MHz, Chloroform-*d*) (**1v**):

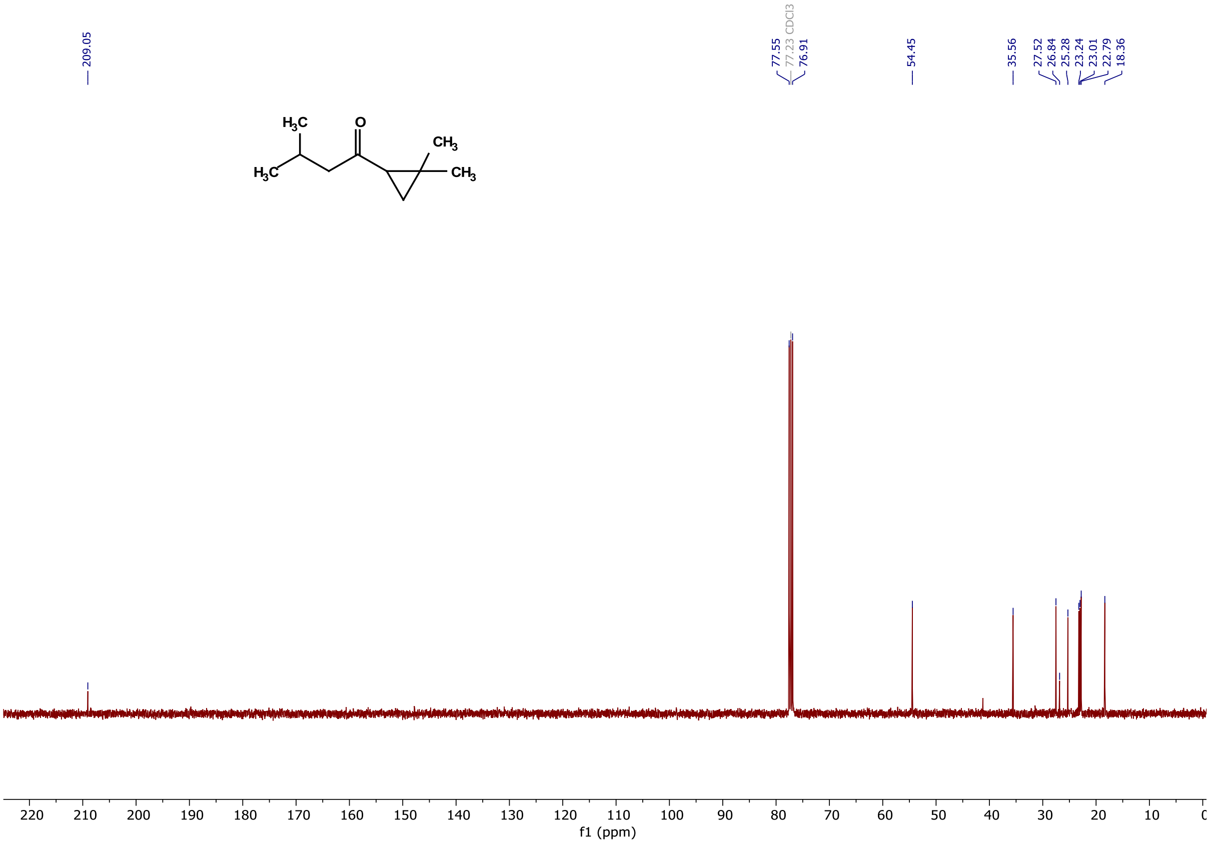

DEPT-135 NMR (101 MHz, Chloroform-*d*) (**1v**):

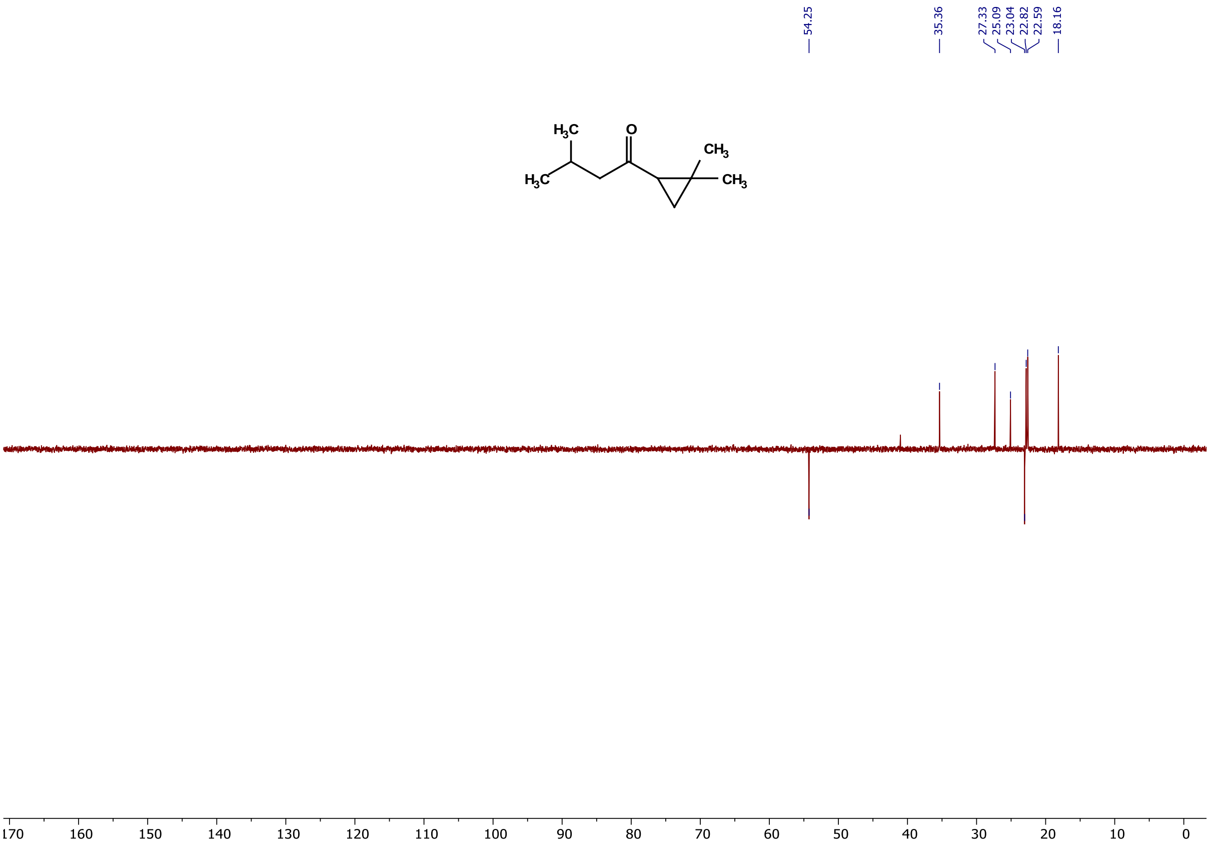

^{1}H NMR (400 MHz, Chloroform-*d*) (**1w**):

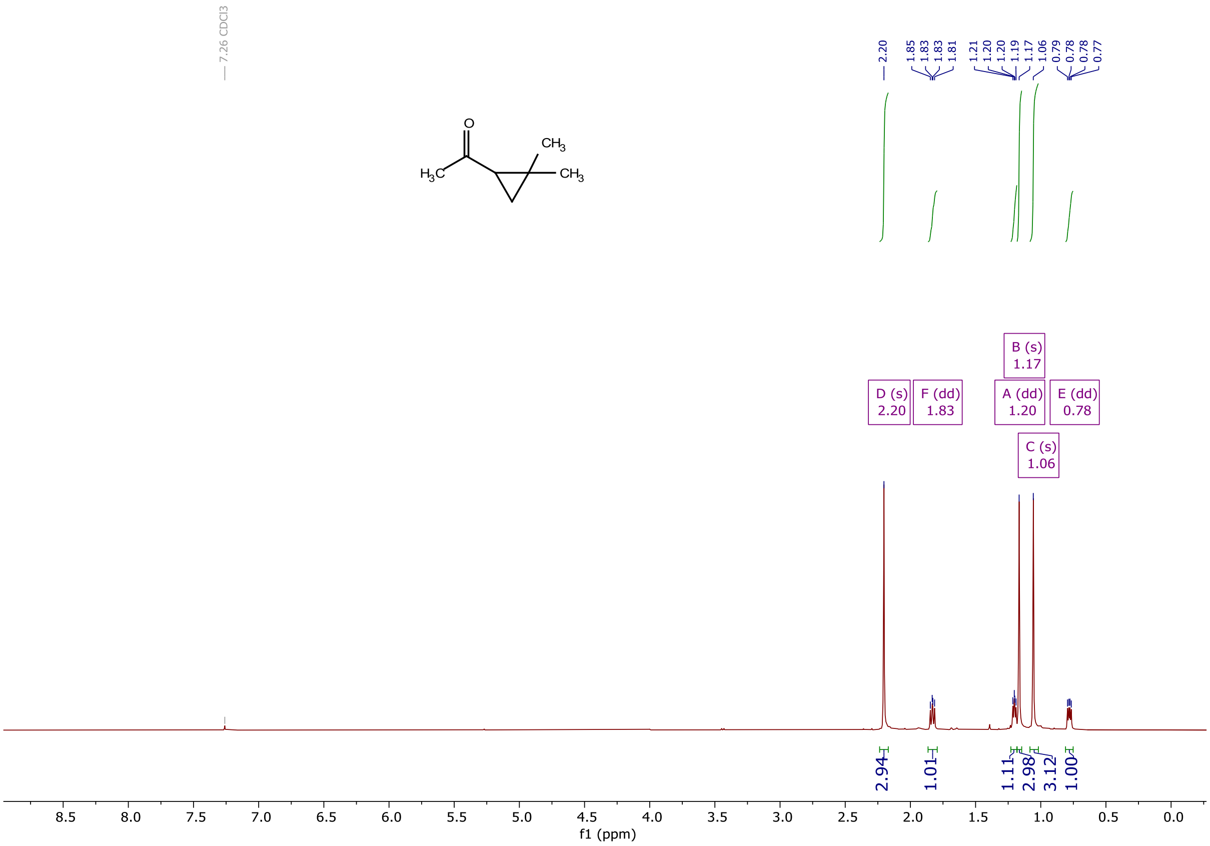

^{13}C NMR (101 MHz, Chloroform-*d*) (**1w**):

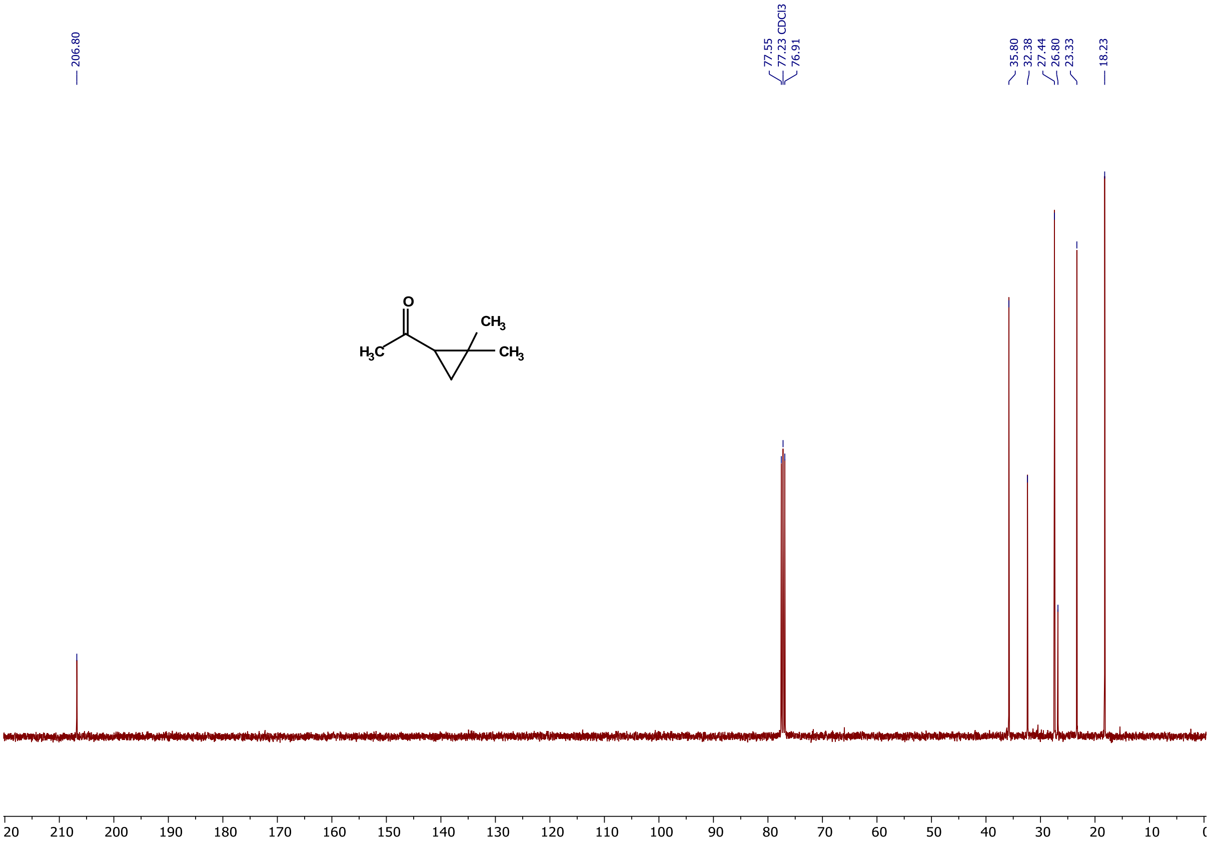

DEPT-135 NMR (101 MHz, Chloroform-*d*) (**1w**):

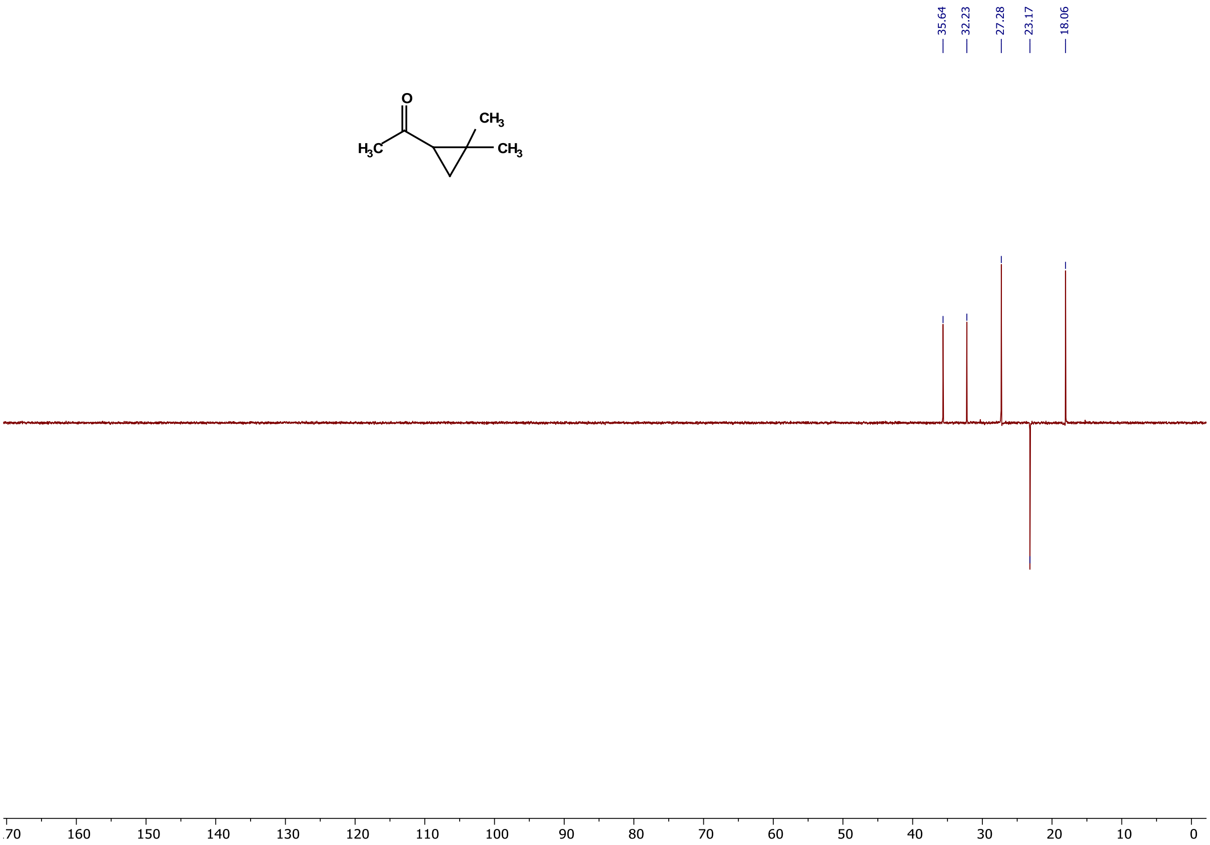

^{1}H NMR (400 MHz, Chloroform-*d*) (**3a**):

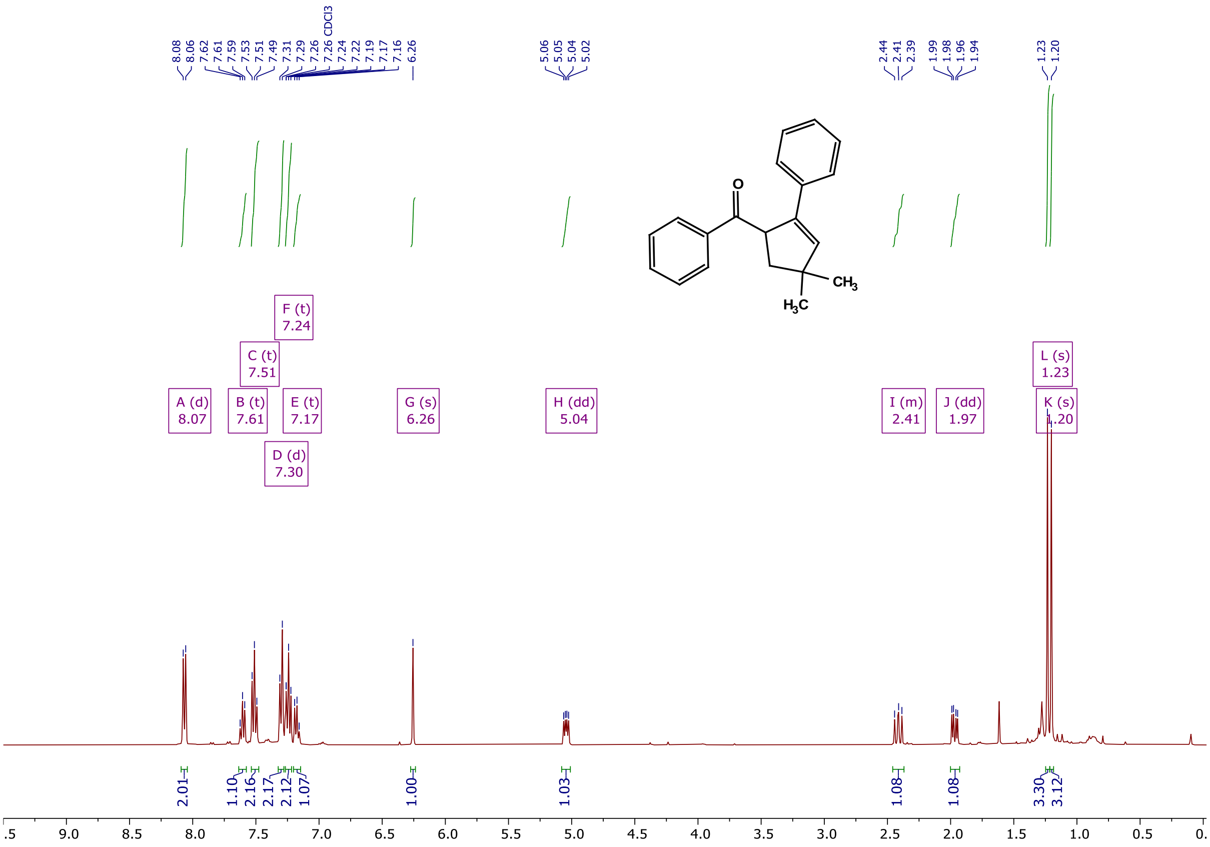

^{13}C NMR (101 MHz, Chloroform-*d*) (**3a**):

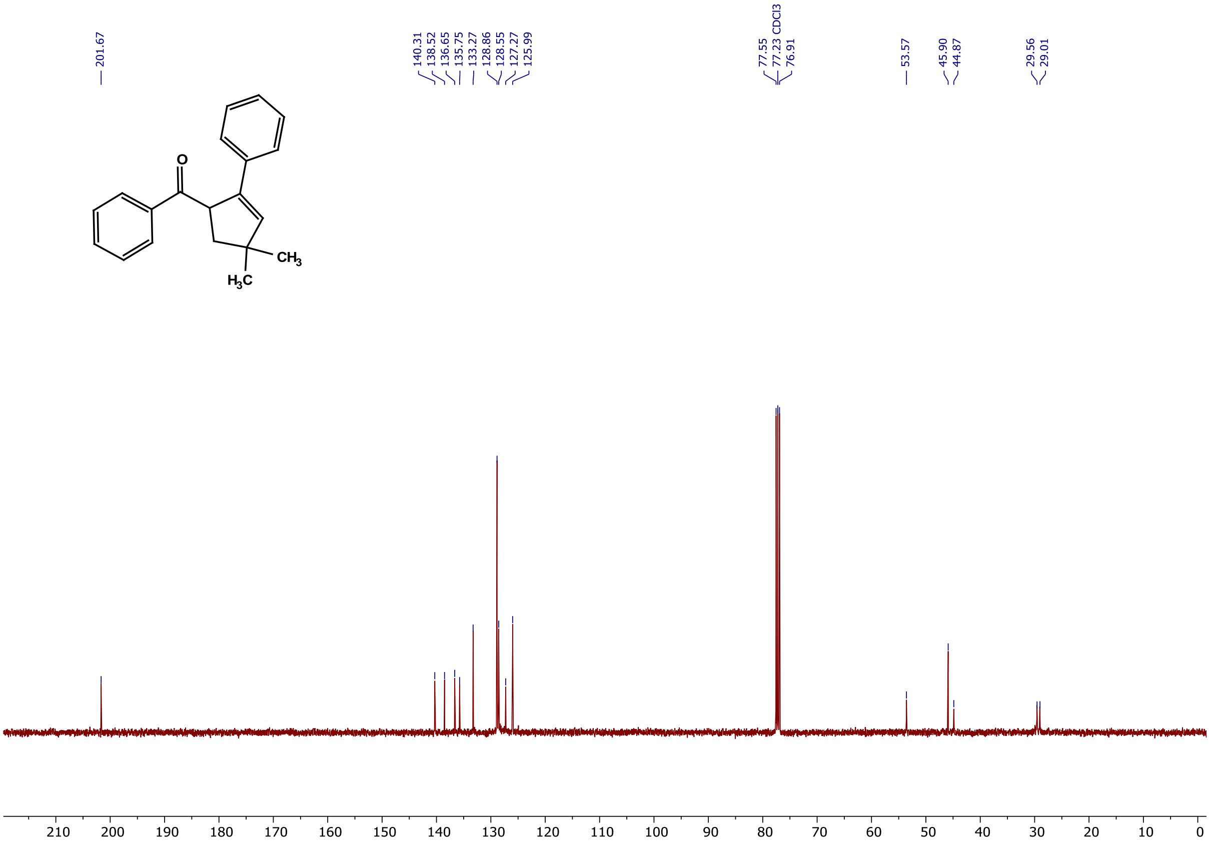

^{1}H NMR (400 MHz, Chloroform-*d*) (**3b**):

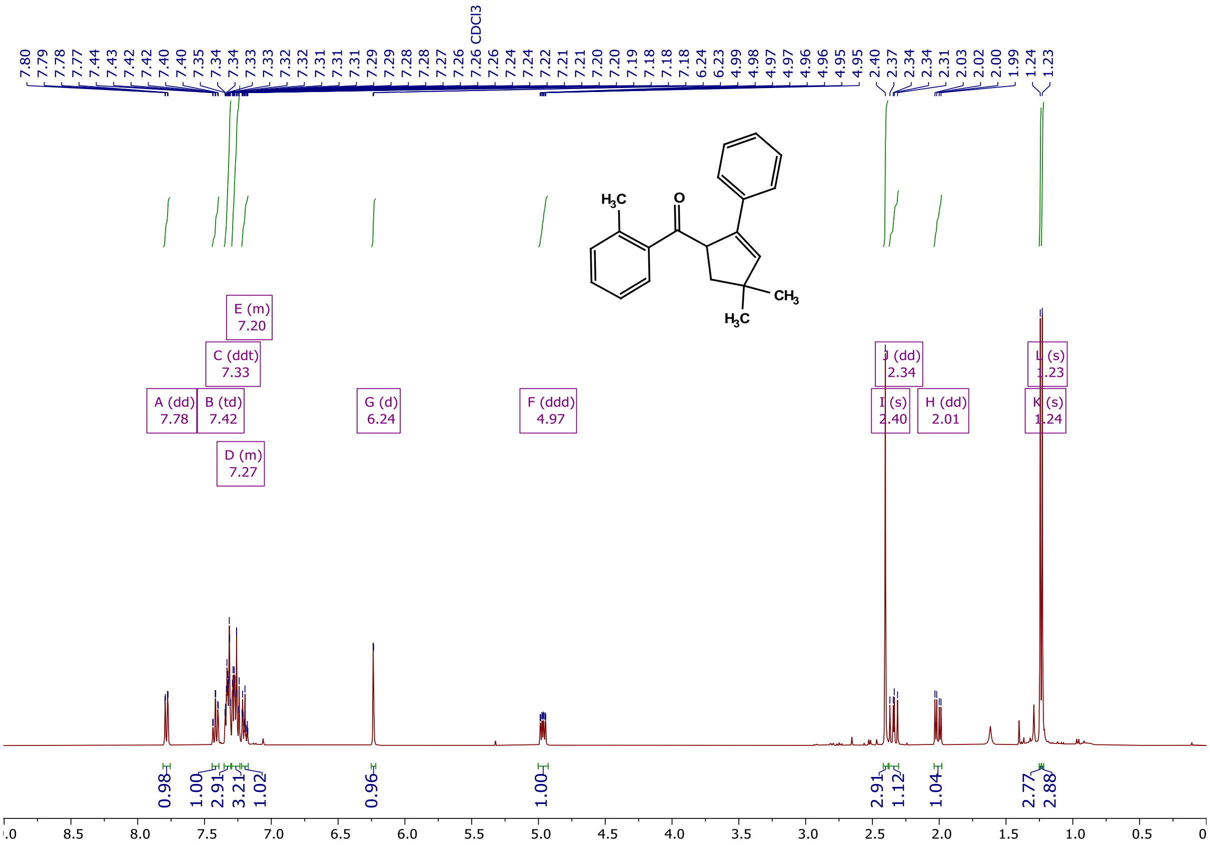

^{13}C NMR (101 MHz, Chloroform-*d*) (**3b**):

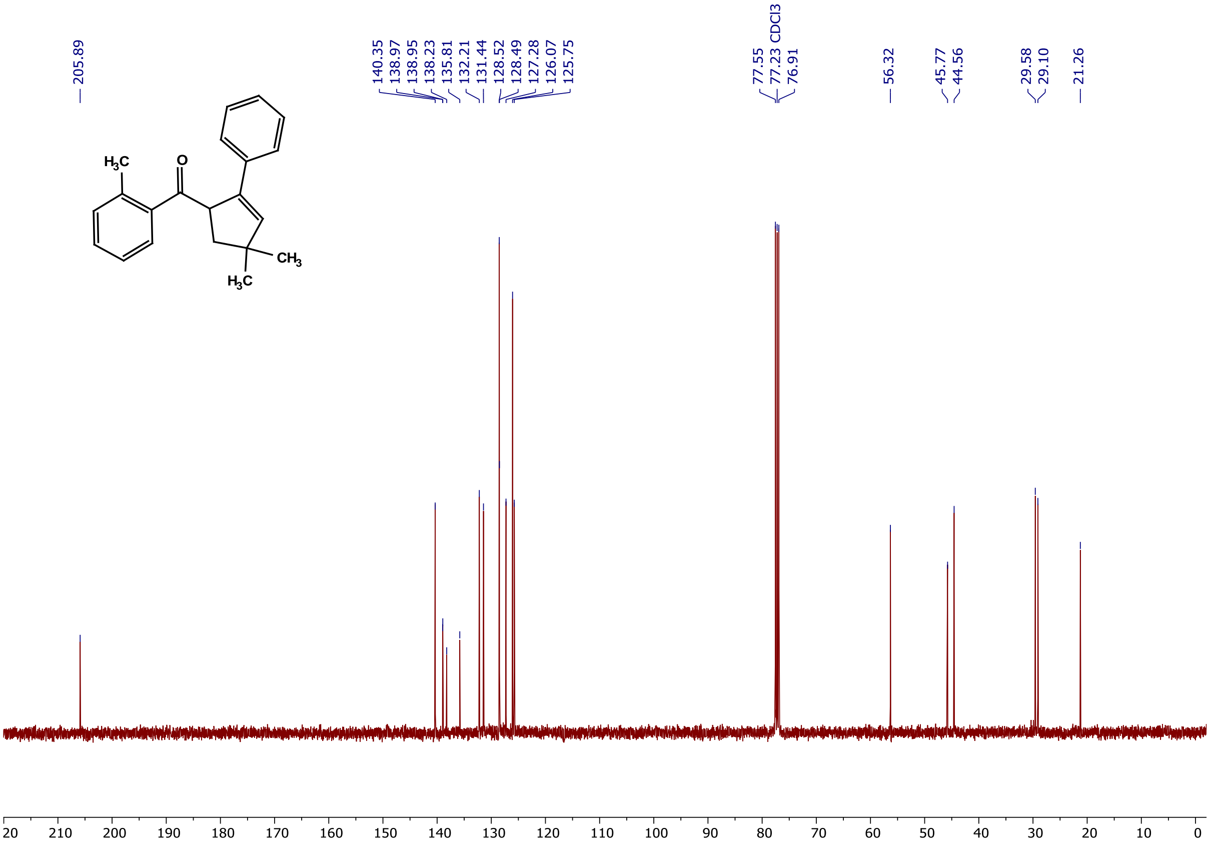

DEPT-135 NMR (101 MHz, Chloroform-*d*) (**3b**):

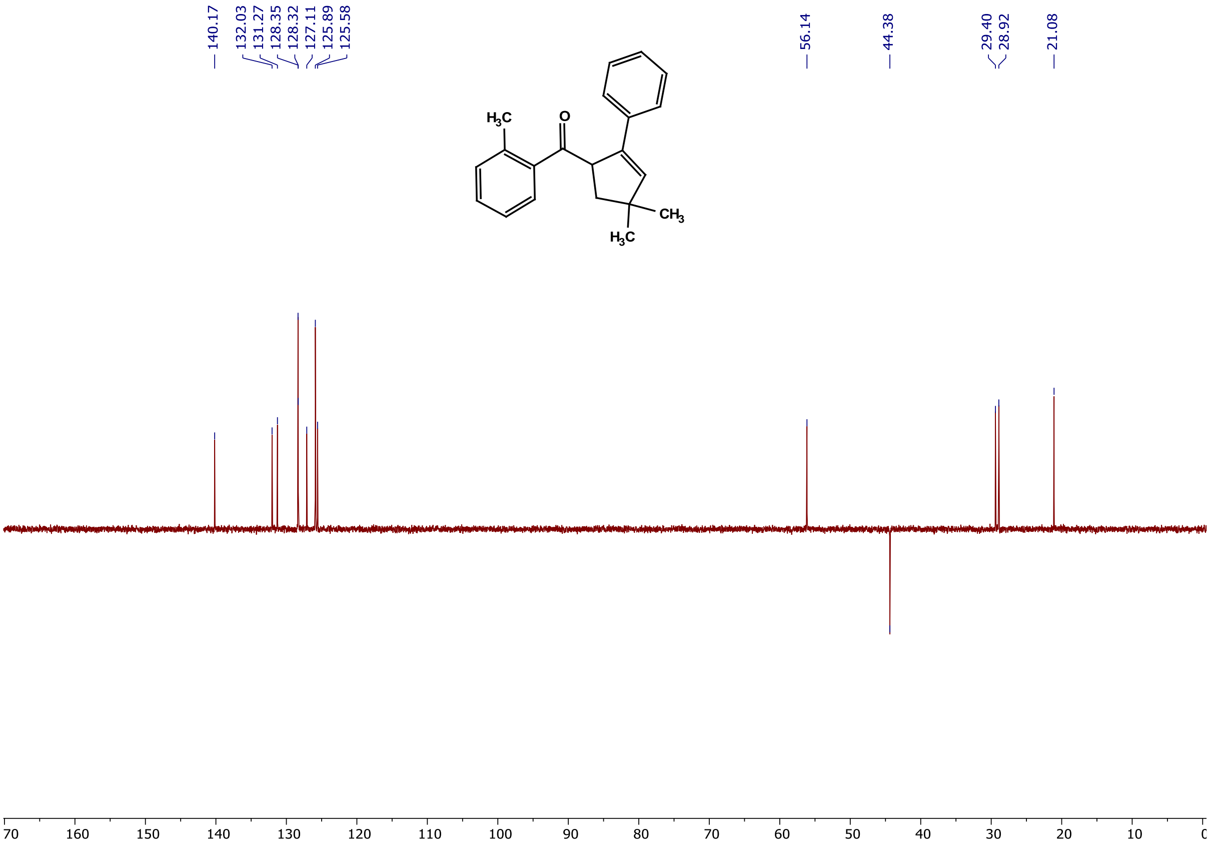

^{1}H NMR (400 MHz, Chloroform-*d*) (**3c**):

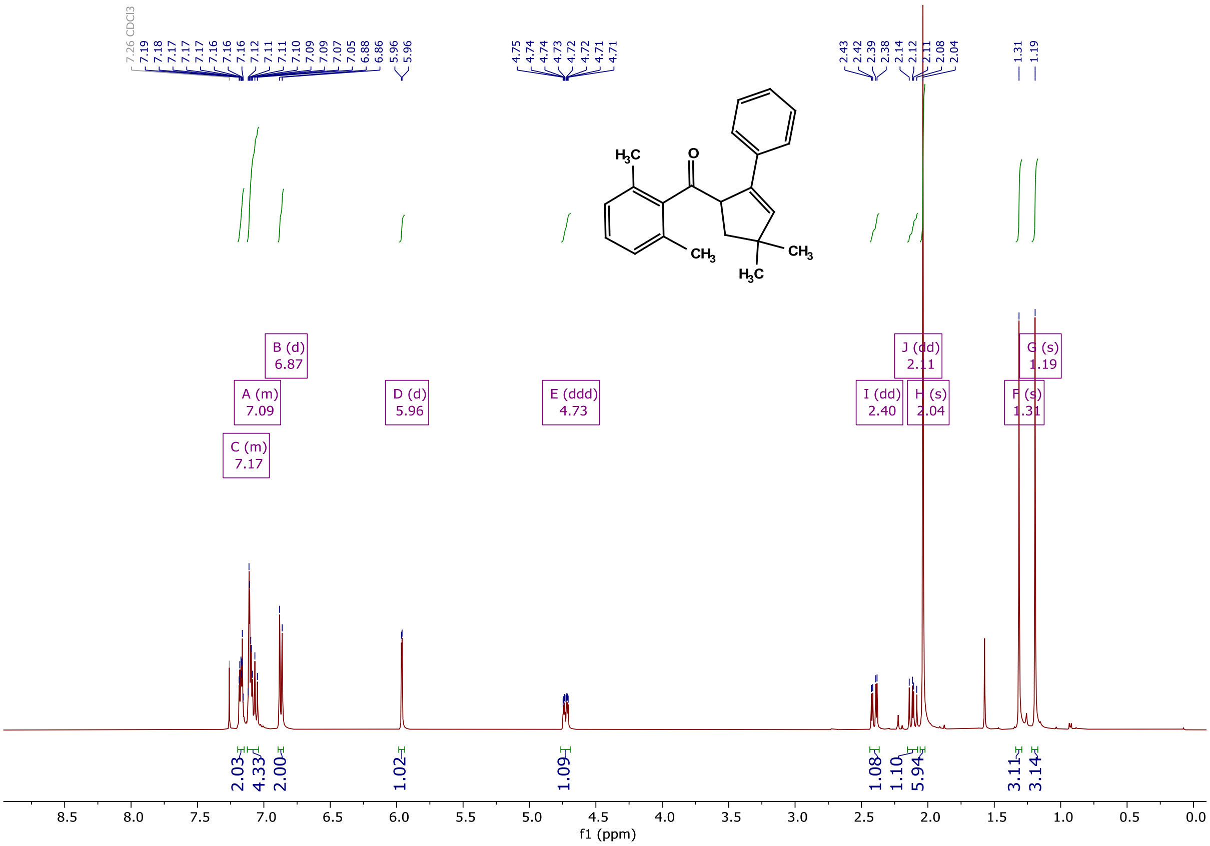

^{13}C NMR (101 MHz, Chloroform-*d*) (**3c**):

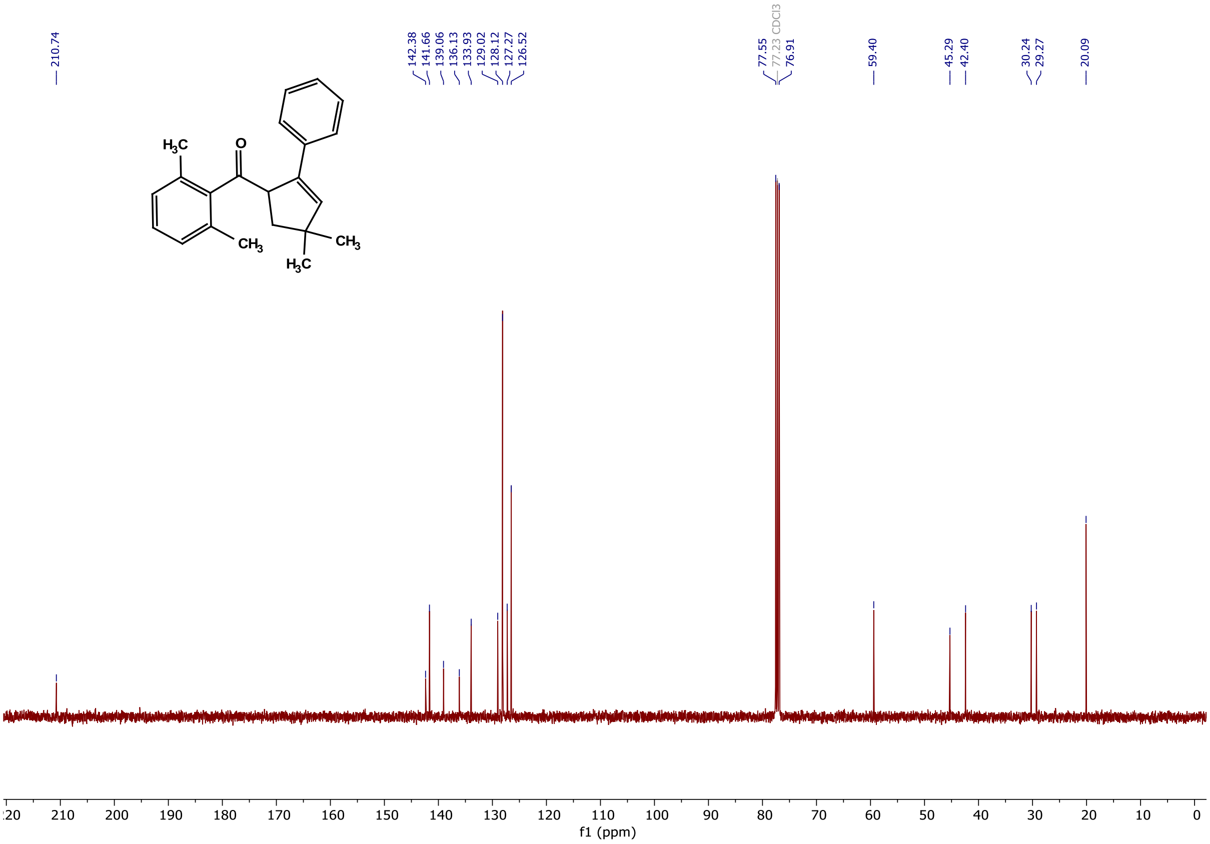

DEPT-135 NMR (101 MHz, Chloroform-*d*) (**3c**):

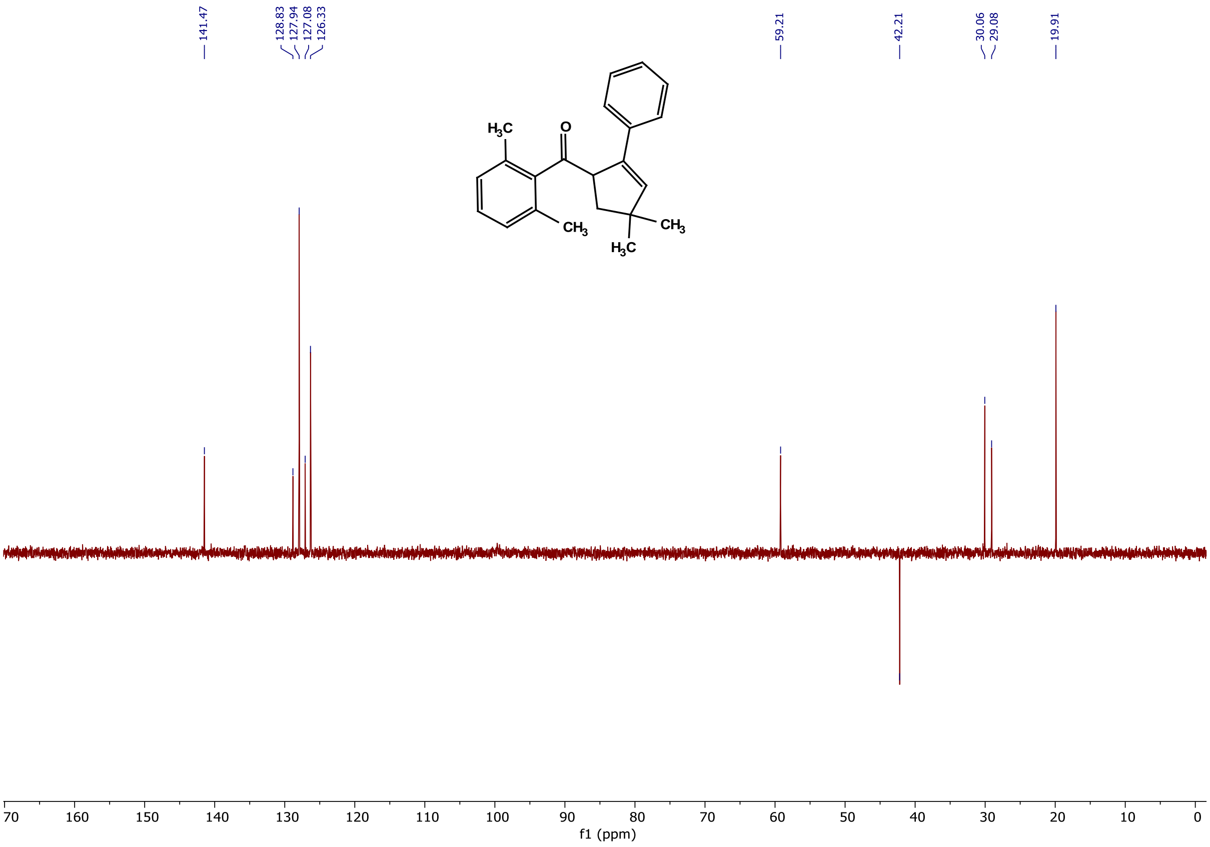

^{1}H NMR (400 MHz, Chloroform-*d*) (**3e**):

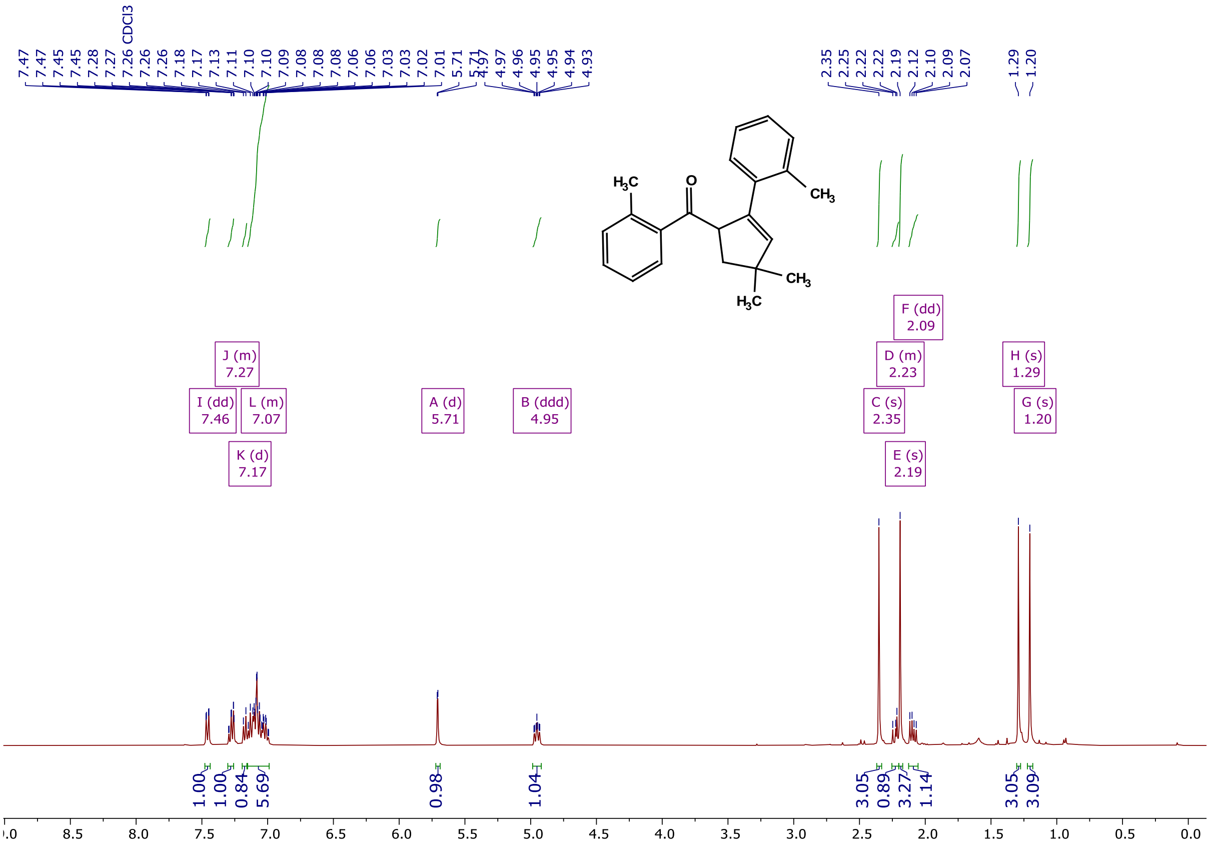

^{13}C NMR (101 MHz, Chloroform-*d*) (**3e**):

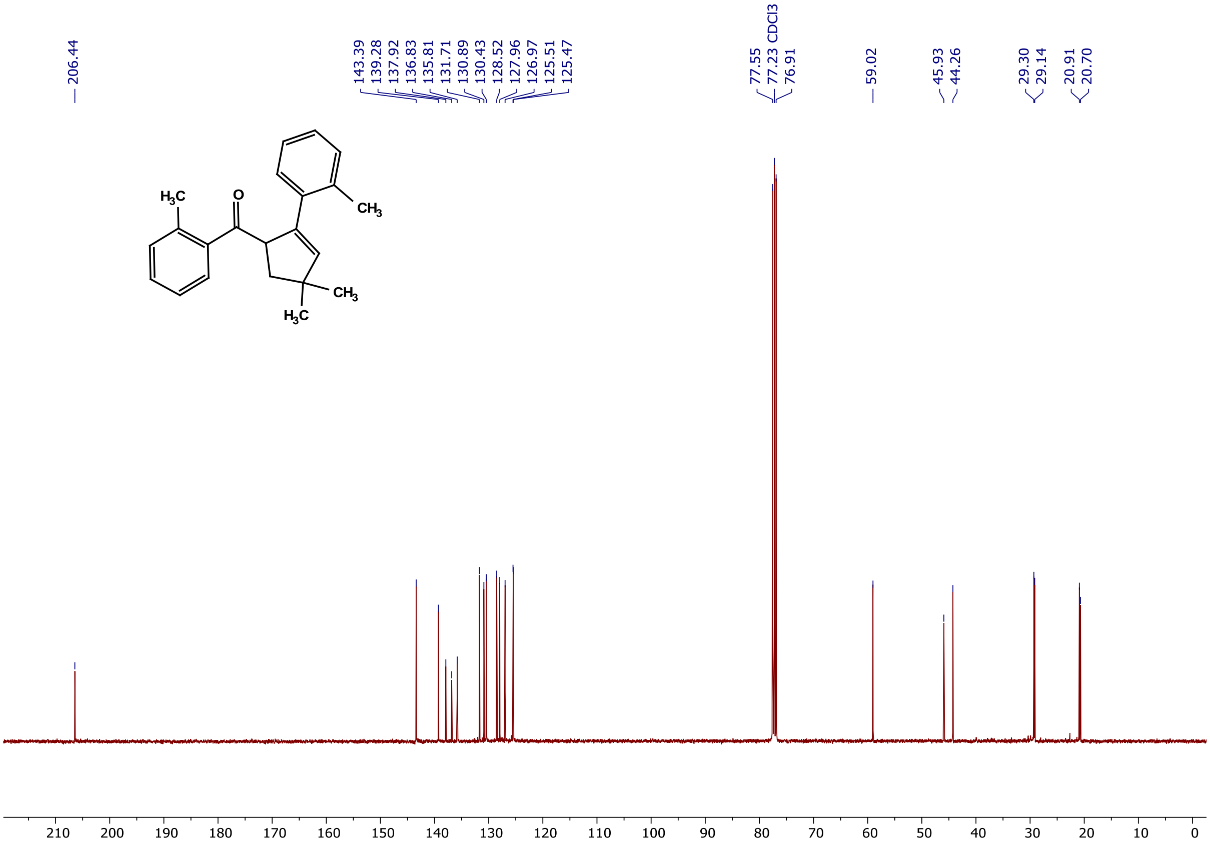

DEPT-135 NMR (101 MHz, Chloroform-*d*) (**3e**):

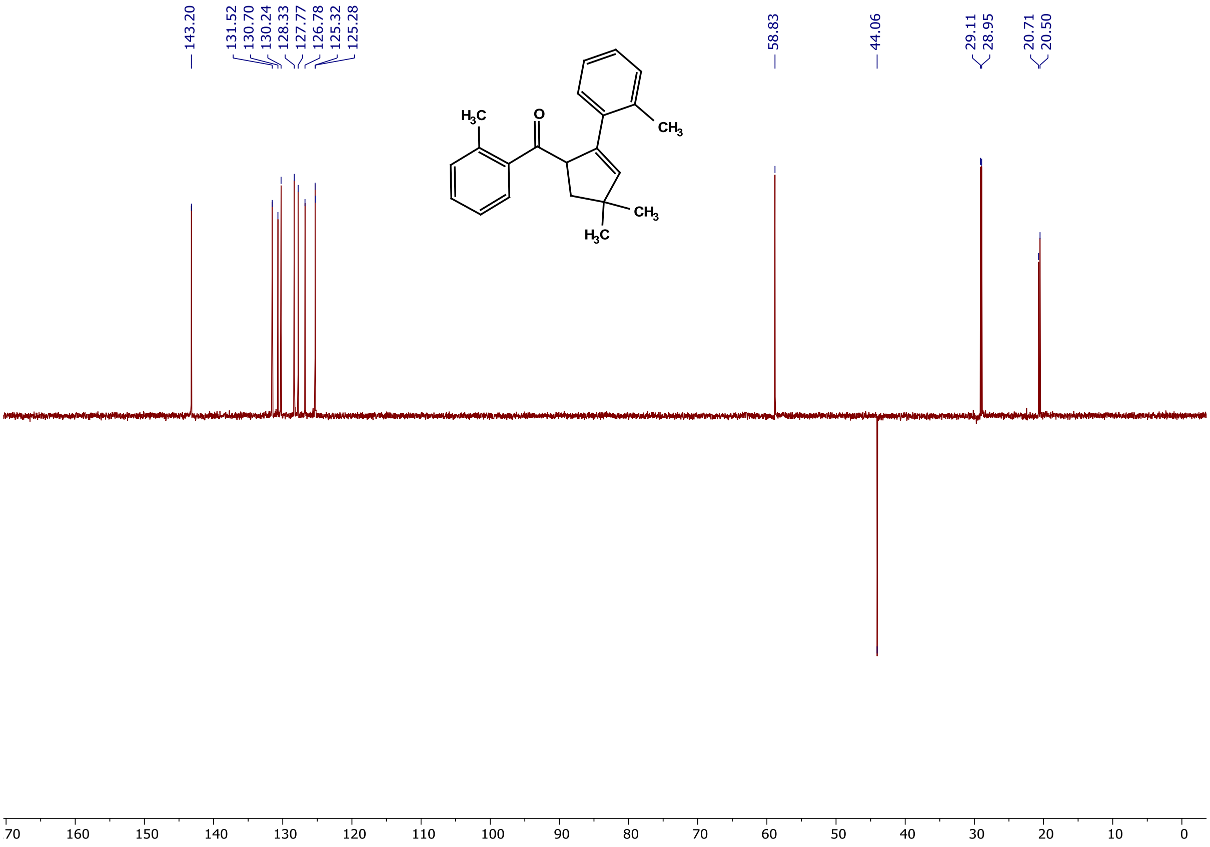

^{1}H NMR (400 MHz, Chloroform-*d*) (**3f**):

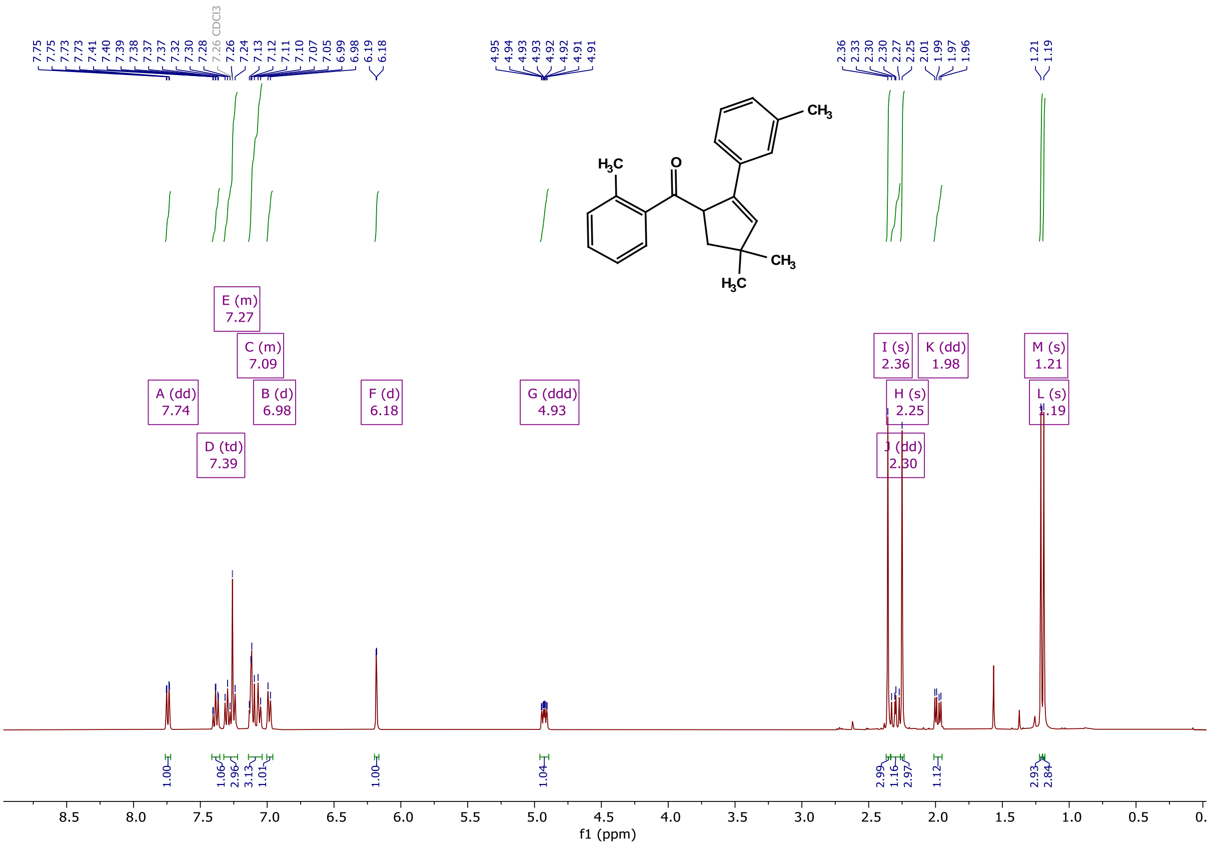

^{13}C NMR (101 MHz, Chloroform-*d*) (**3f**):

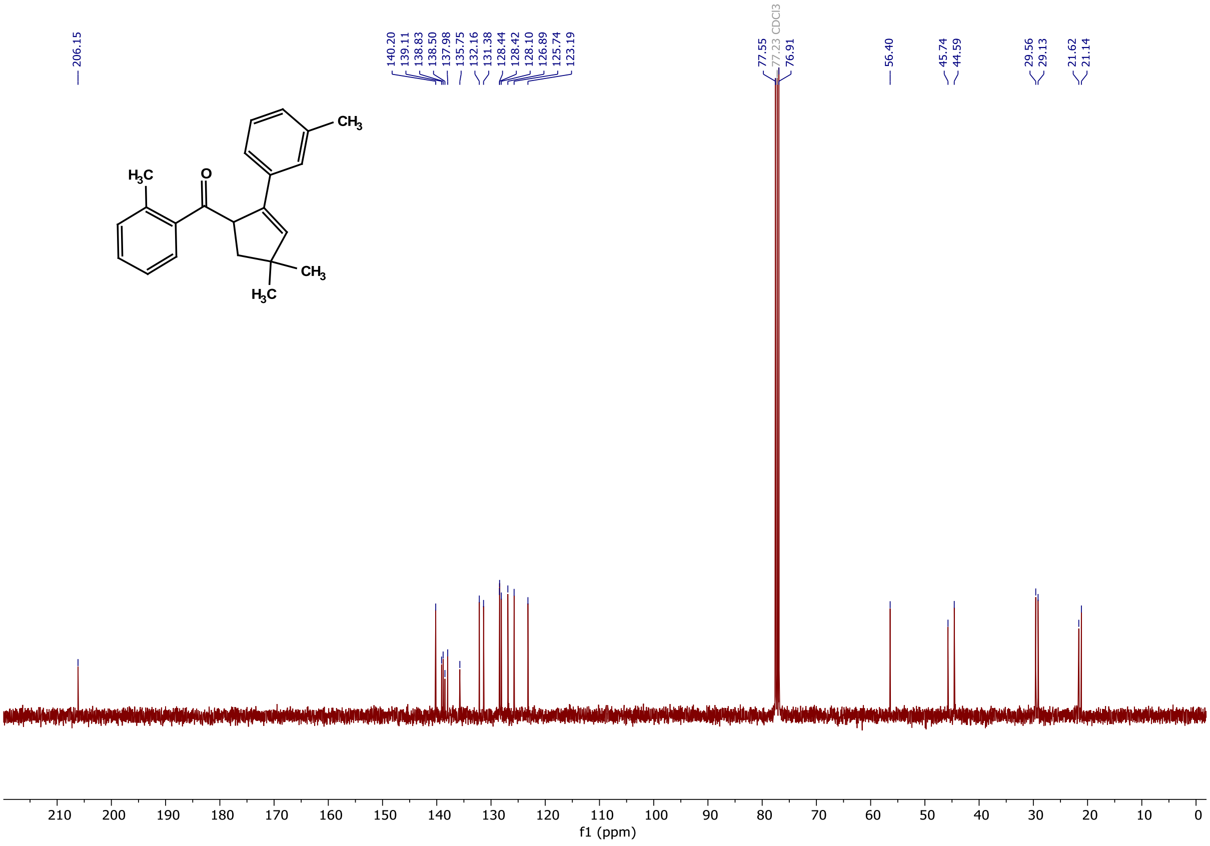

DEPT-135 NMR (101 MHz, Chloroform-*d*) (**3f**):

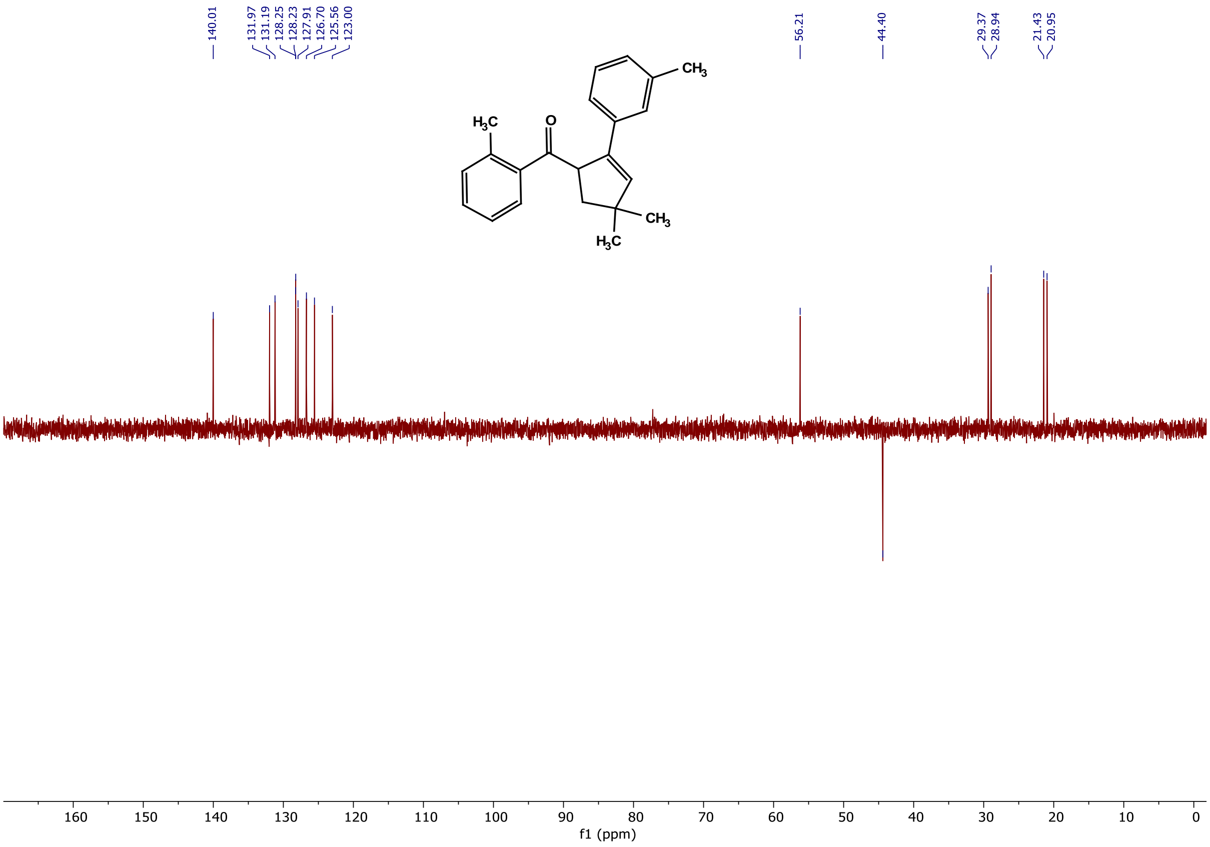

^{1}H NMR (400 MHz, Chloroform-*d*) (**3g**):

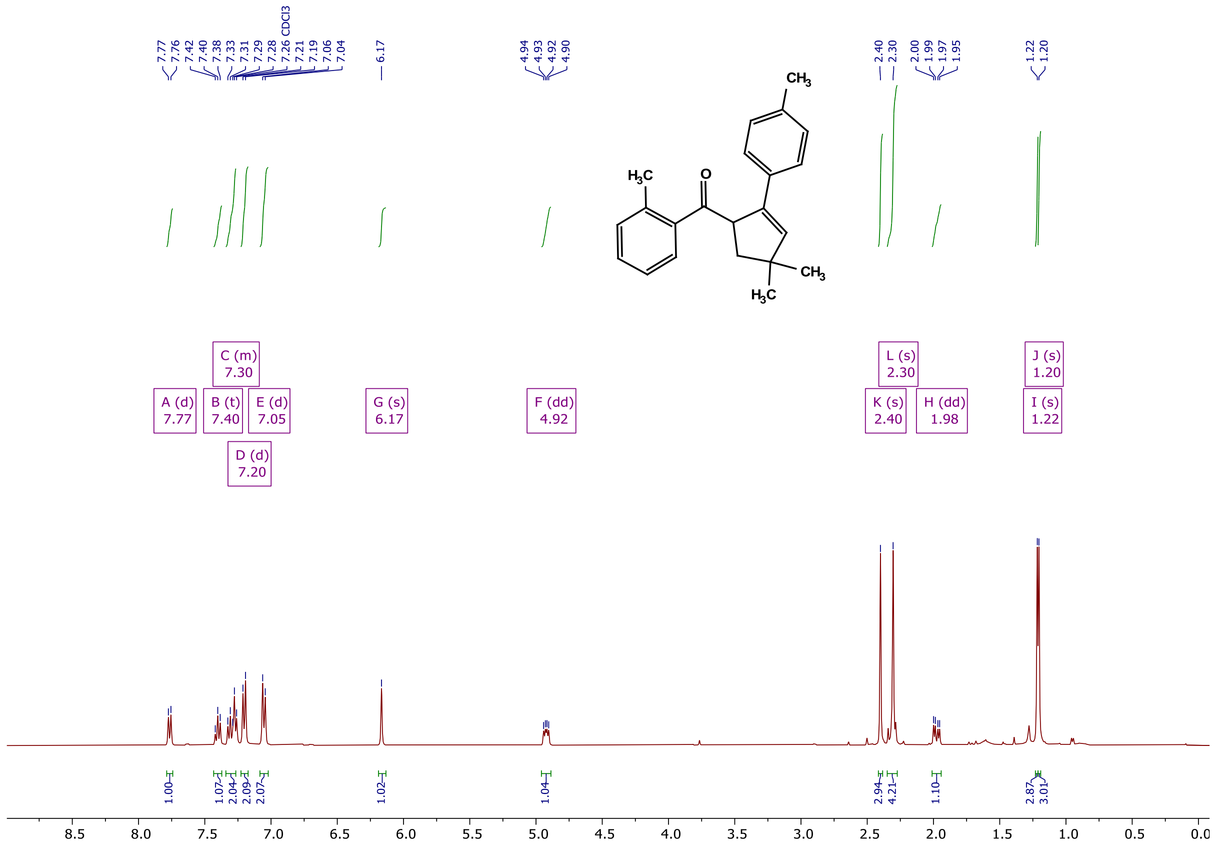

^{13}C NMR (101 MHz, Chloroform-*d*) (**3g**):

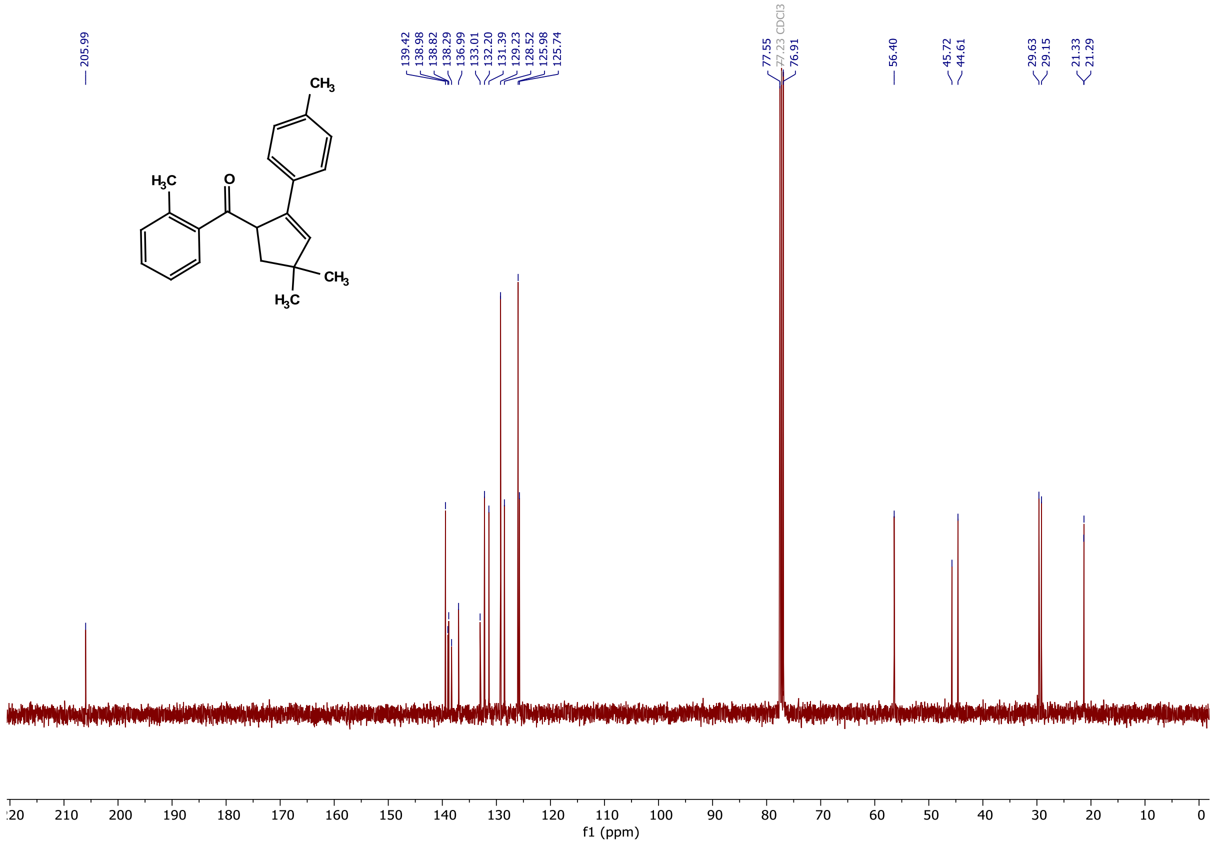

DEPT-135 NMR (101 MHz, Chloroform-*d*) (**3g**):

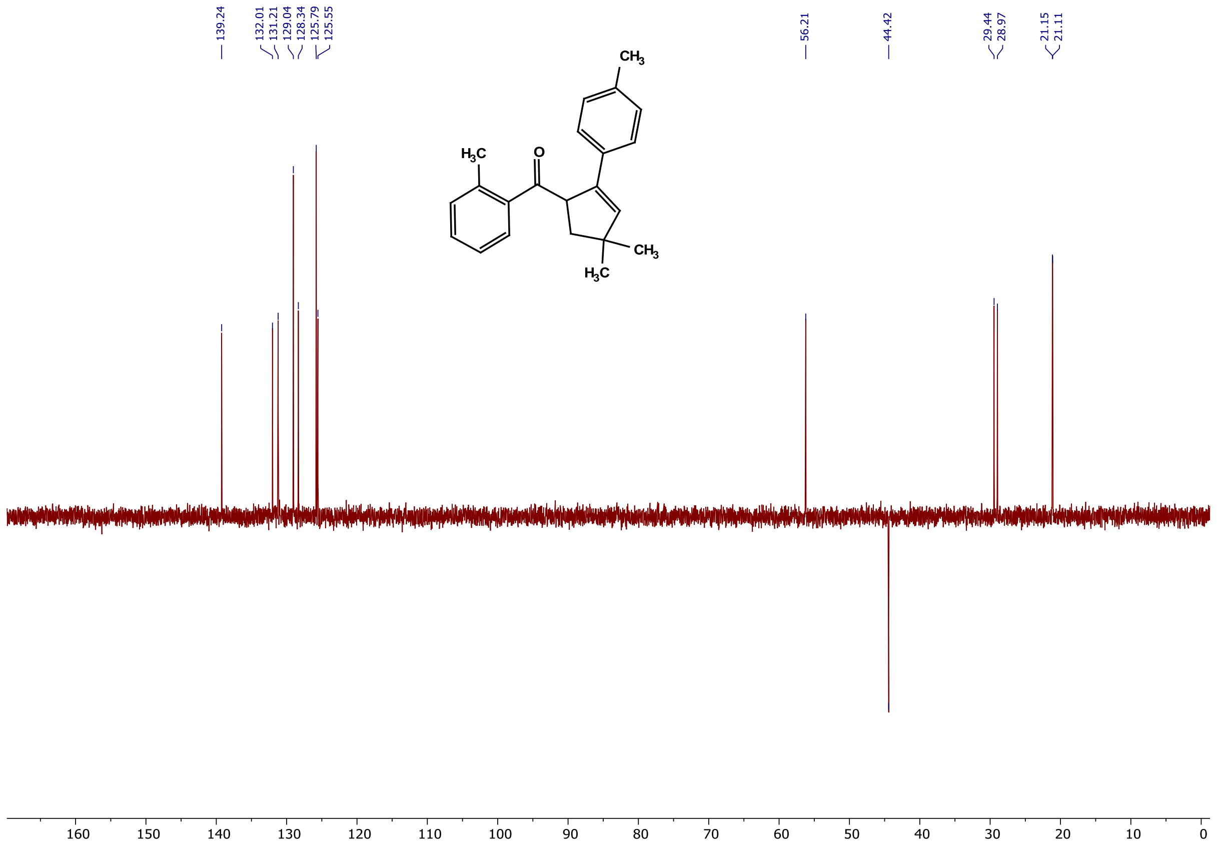

^{1}H NMR (400 MHz, Chloroform-*d*) (**3h**):

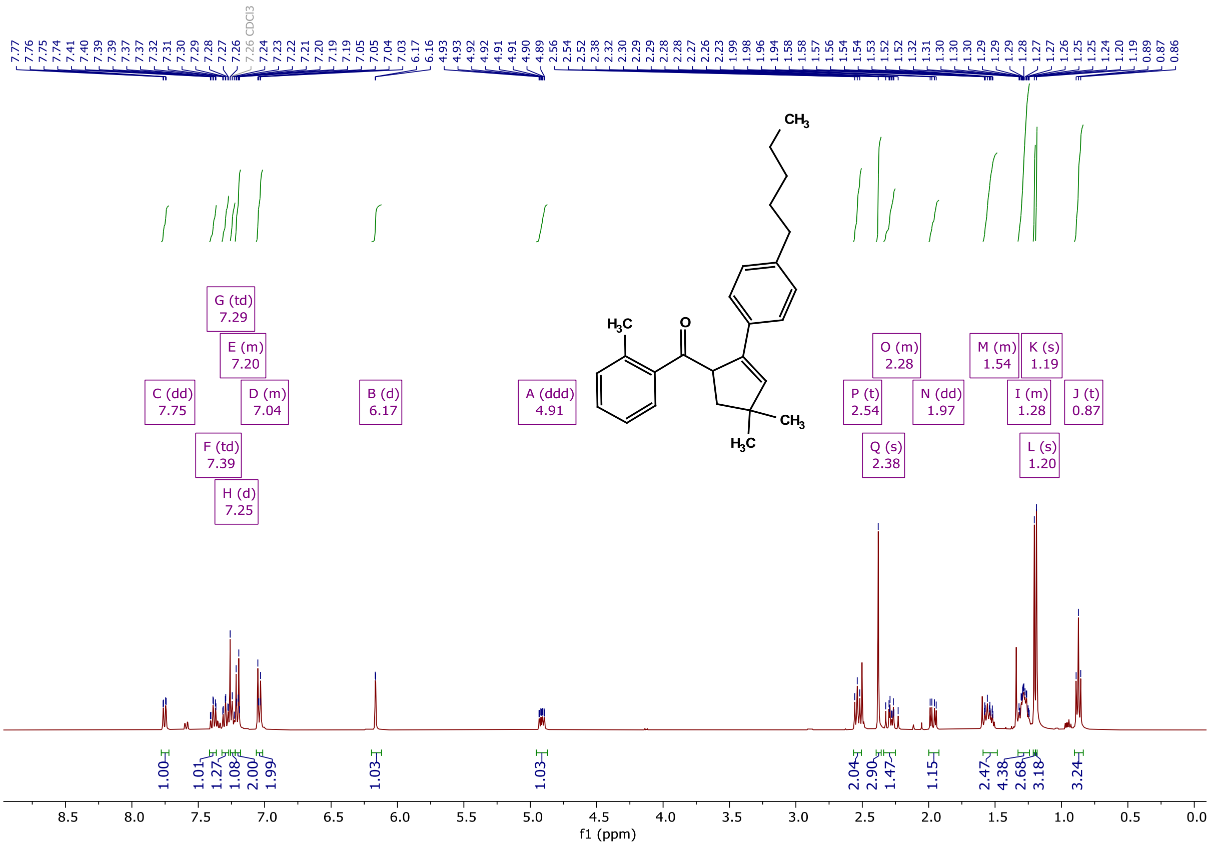

^{13}C NMR (101 MHz, Chloroform-*d*) (**3h**):

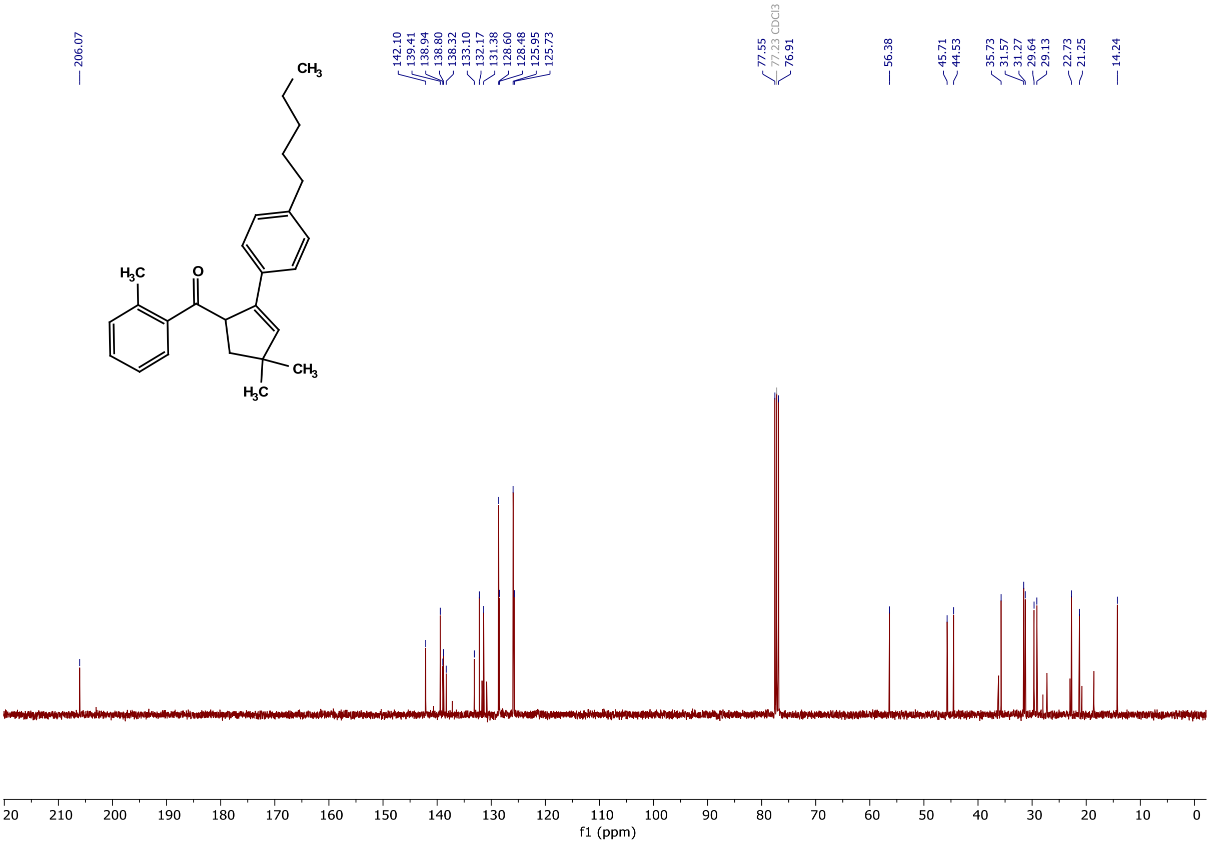

^{1}H NMR (400 MHz, Chloroform-*d*) (**3i**):

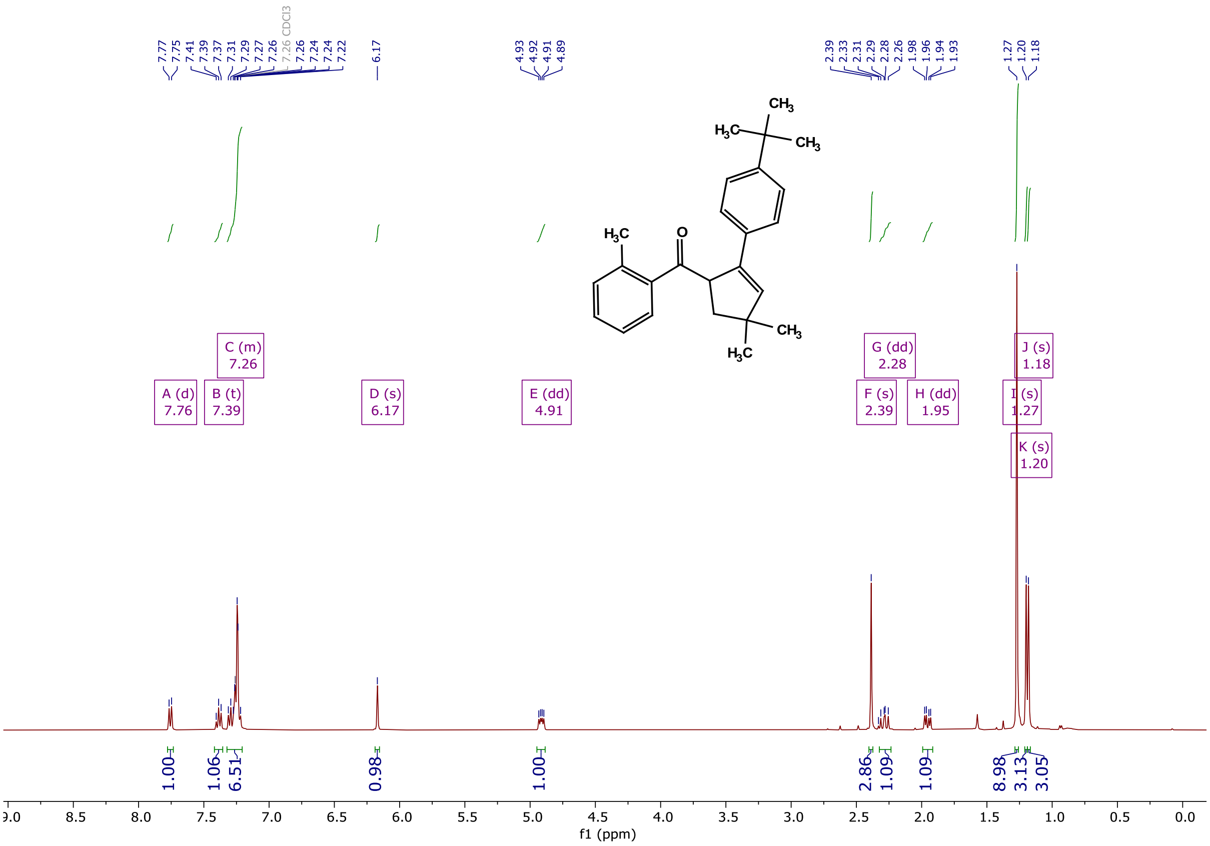

^{13}C NMR (101 MHz, Chloroform-*d*) (**3i**):

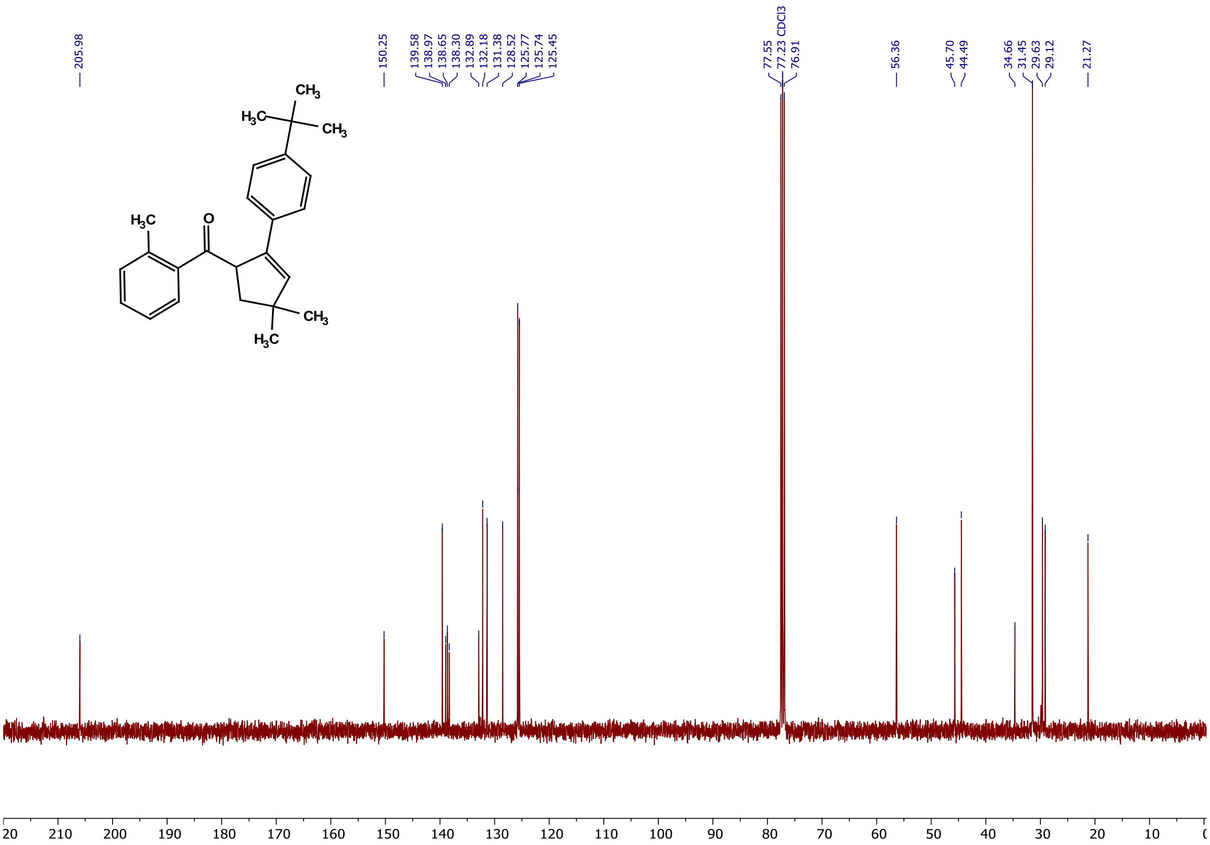

DEPT-135 NMR (101 MHz, Chloroform-*d*) (**3i**):

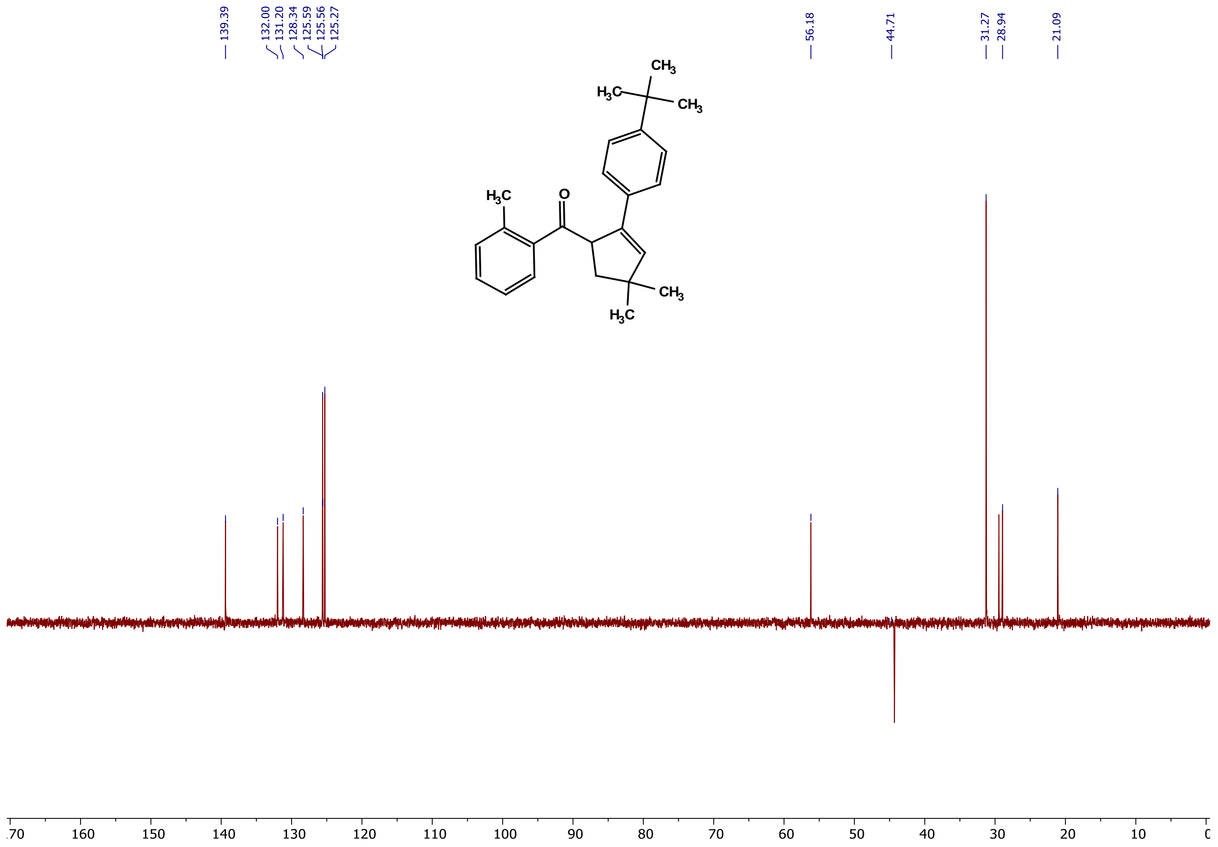

^{1}H NMR (400 MHz, Chloroform-*d*) (**3j**):

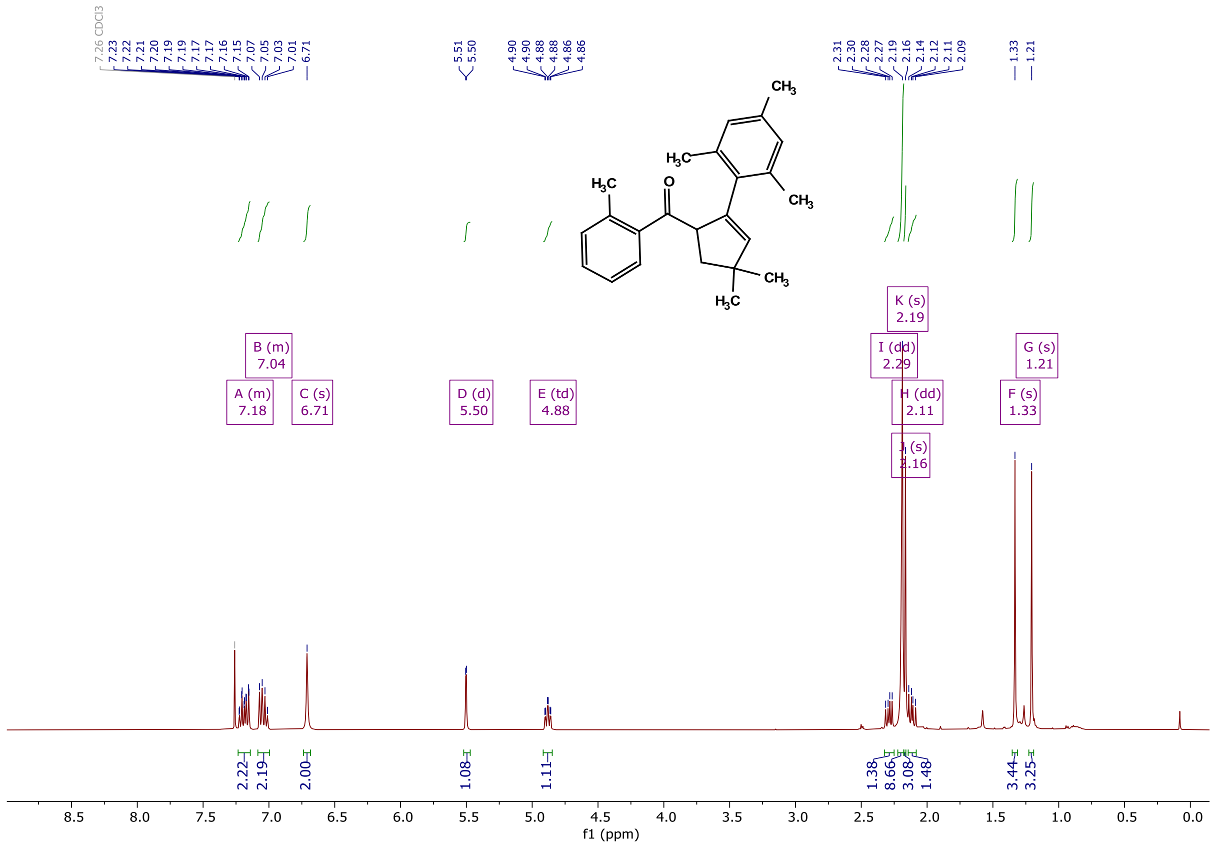

^{13}C NMR (101 MHz, Chloroform-*d*) (**3j**):

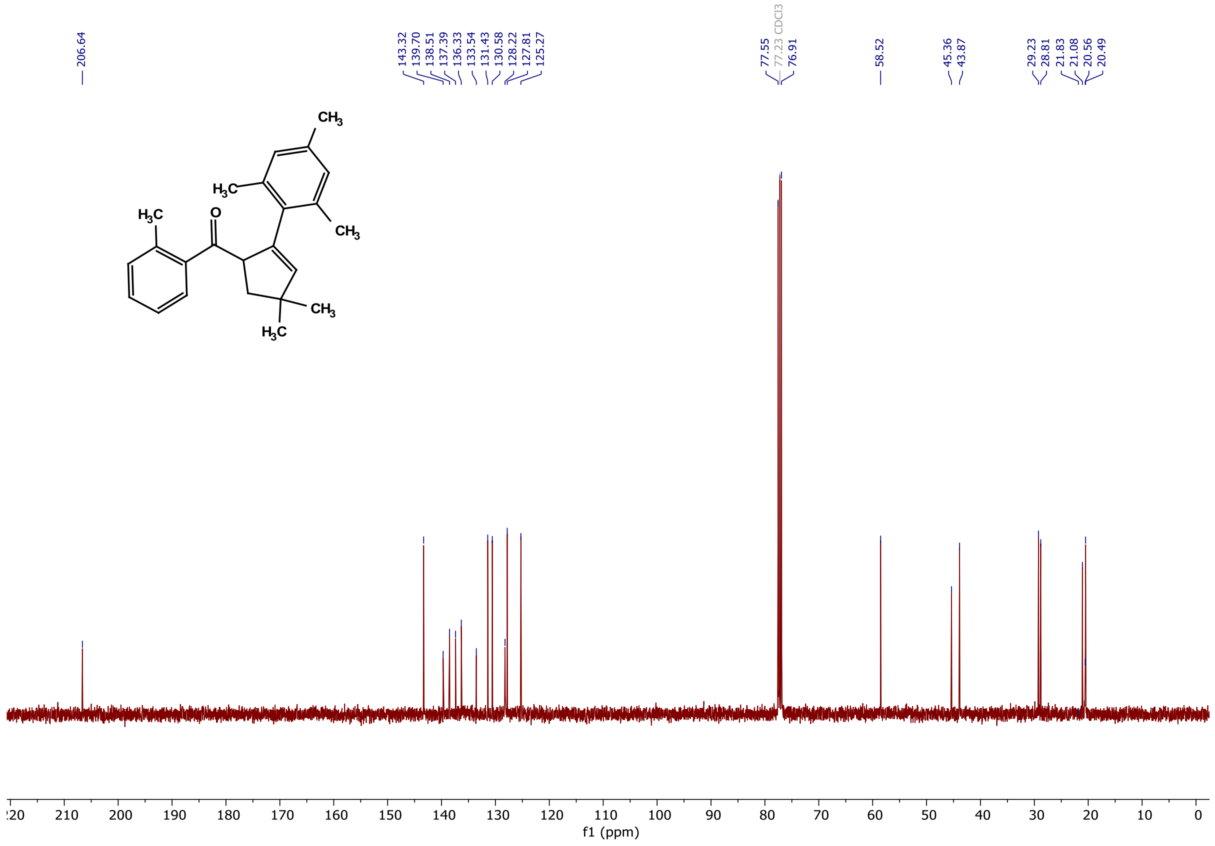

DEPT-135 NMR (101 MHz, Chloroform-*d*) (**3j**):

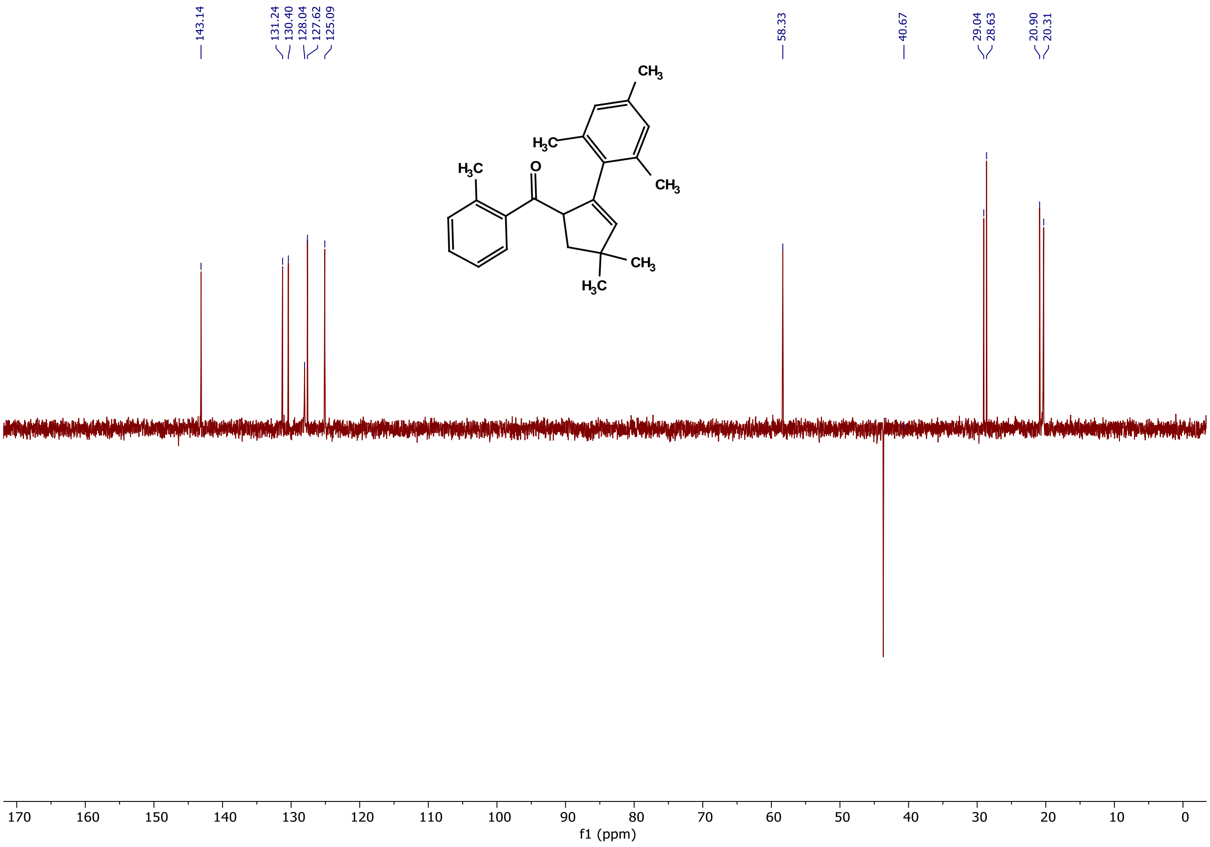

^{1}H NMR (400 MHz, Chloroform-*d*) (**3k**):

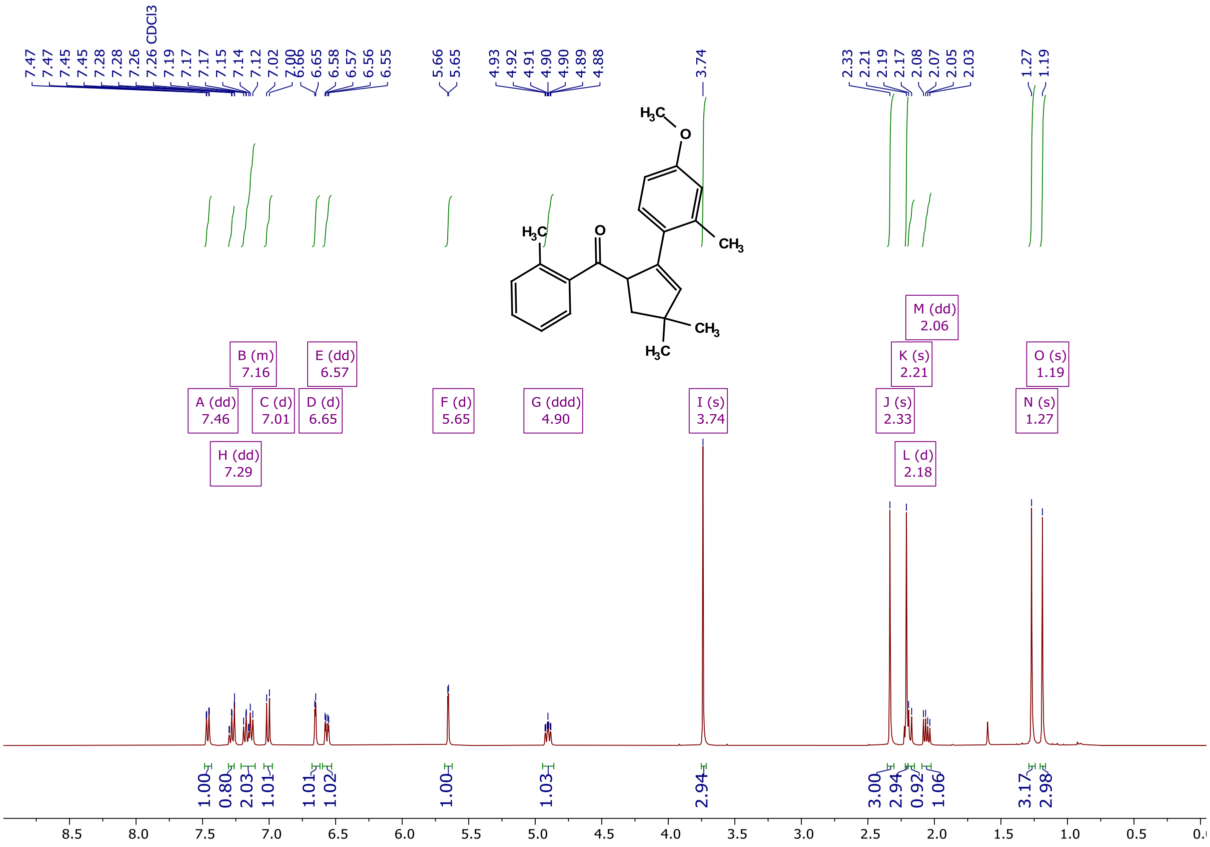

^{13}C NMR (101 MHz, Chloroform-*d*) (**3k**):

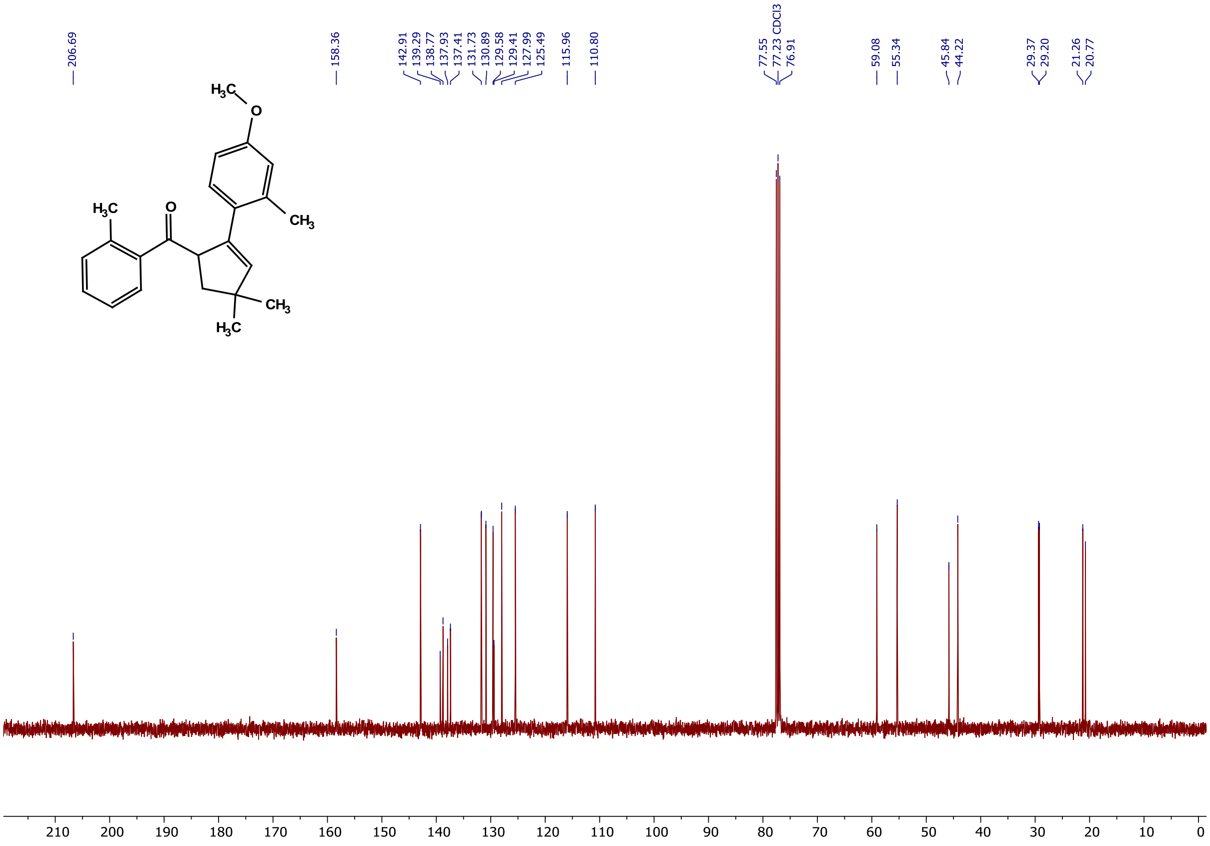

DEPT-135 NMR (101 MHz, Chloroform-*d*) (**3k**):

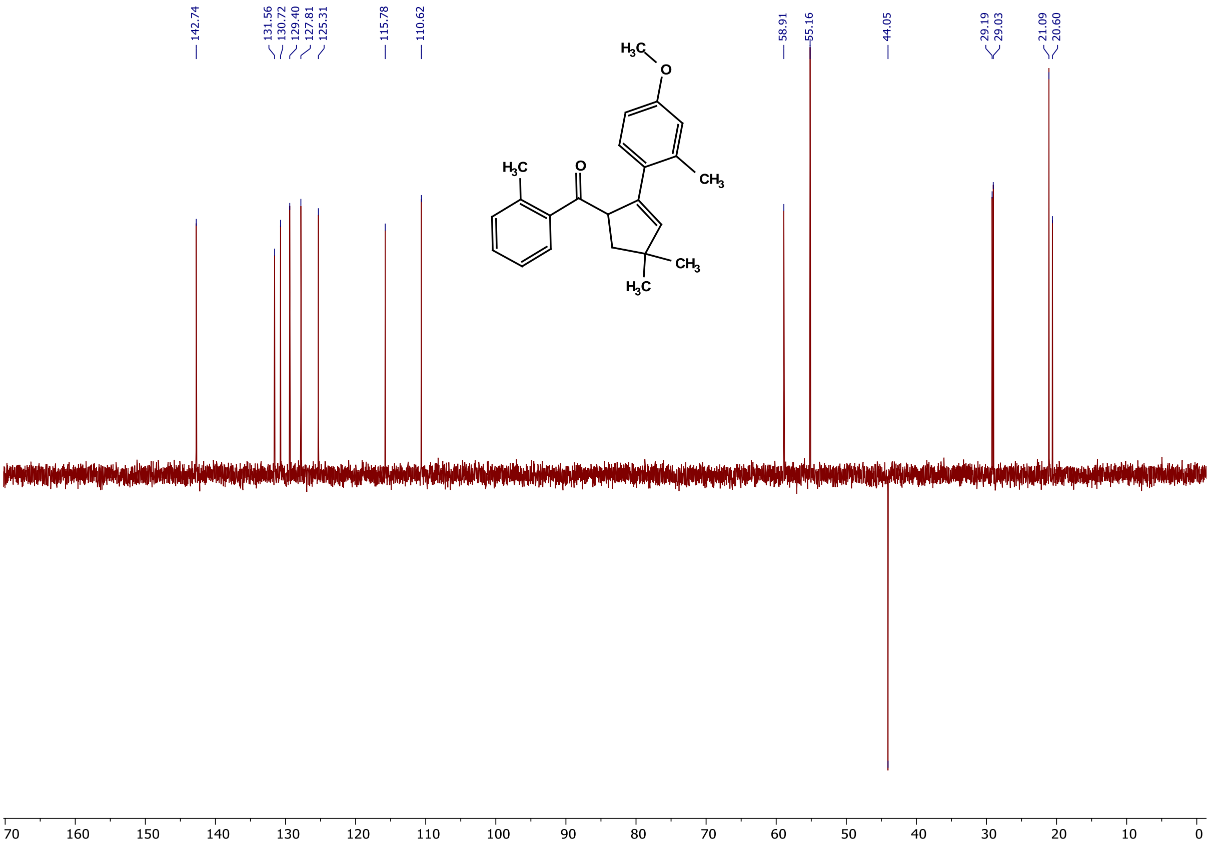

^{1}H NMR (400 MHz, Chloroform-*d*) (**3l**):

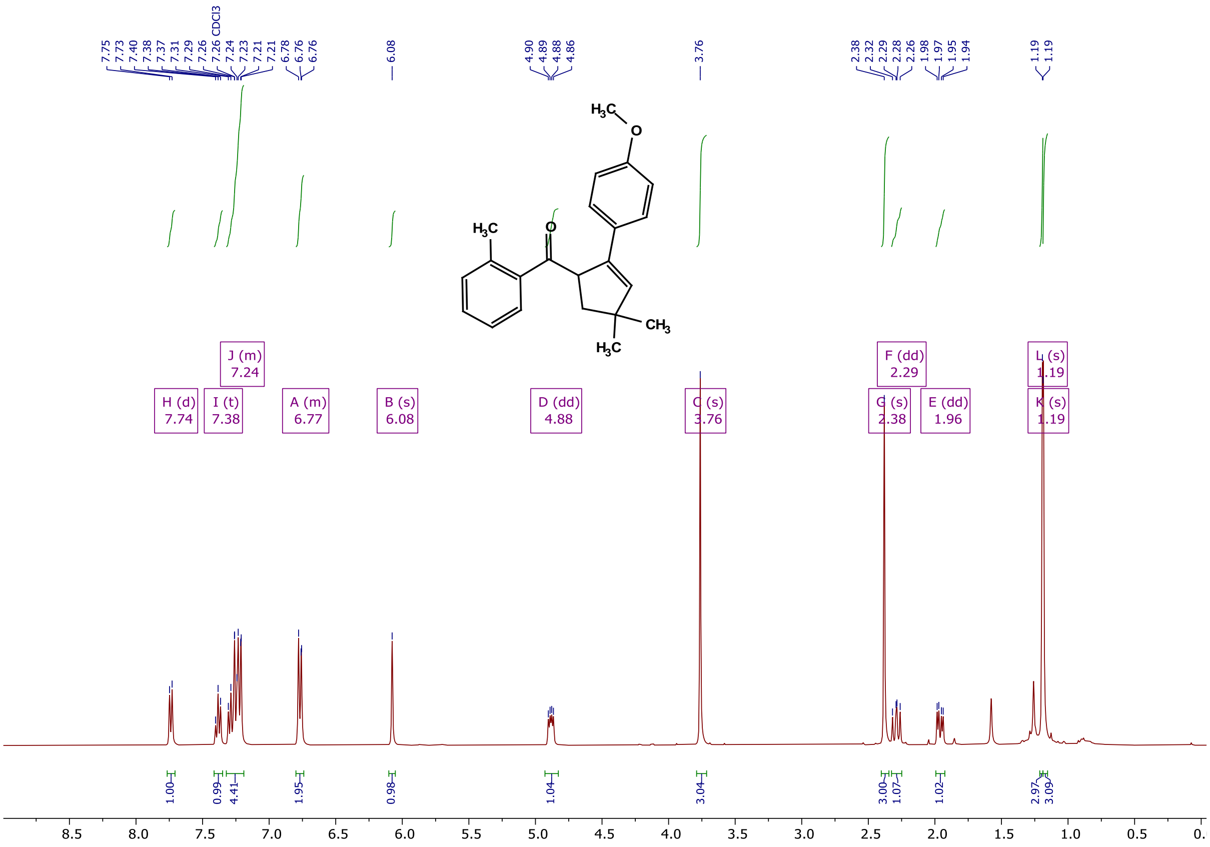

^{13}C NMR (101 MHz, Chloroform-*d*) (**3l**):

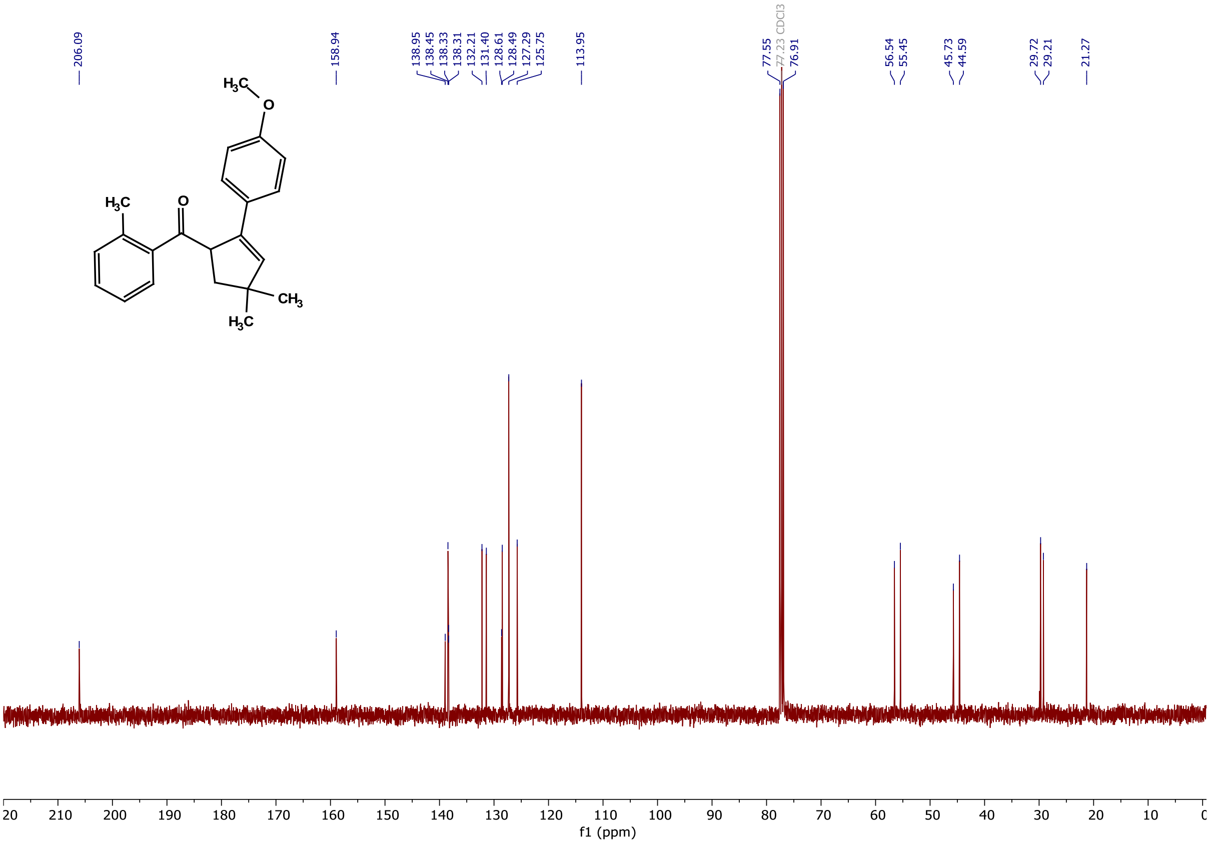

DEPT-135 NMR (101 MHz, Chloroform-*d*) (**3l**):

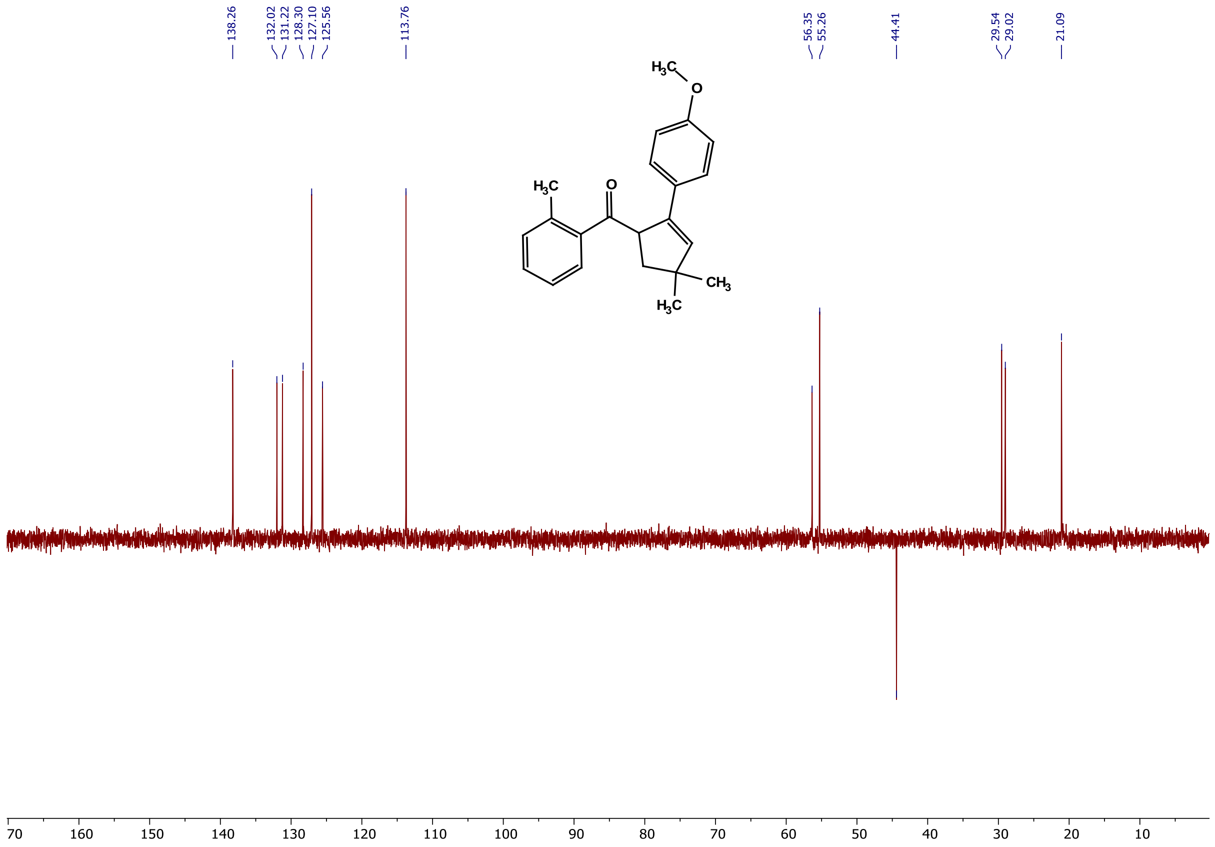

^{1}H NMR (500 MHz, Chloroform-*d*) (**3m**):

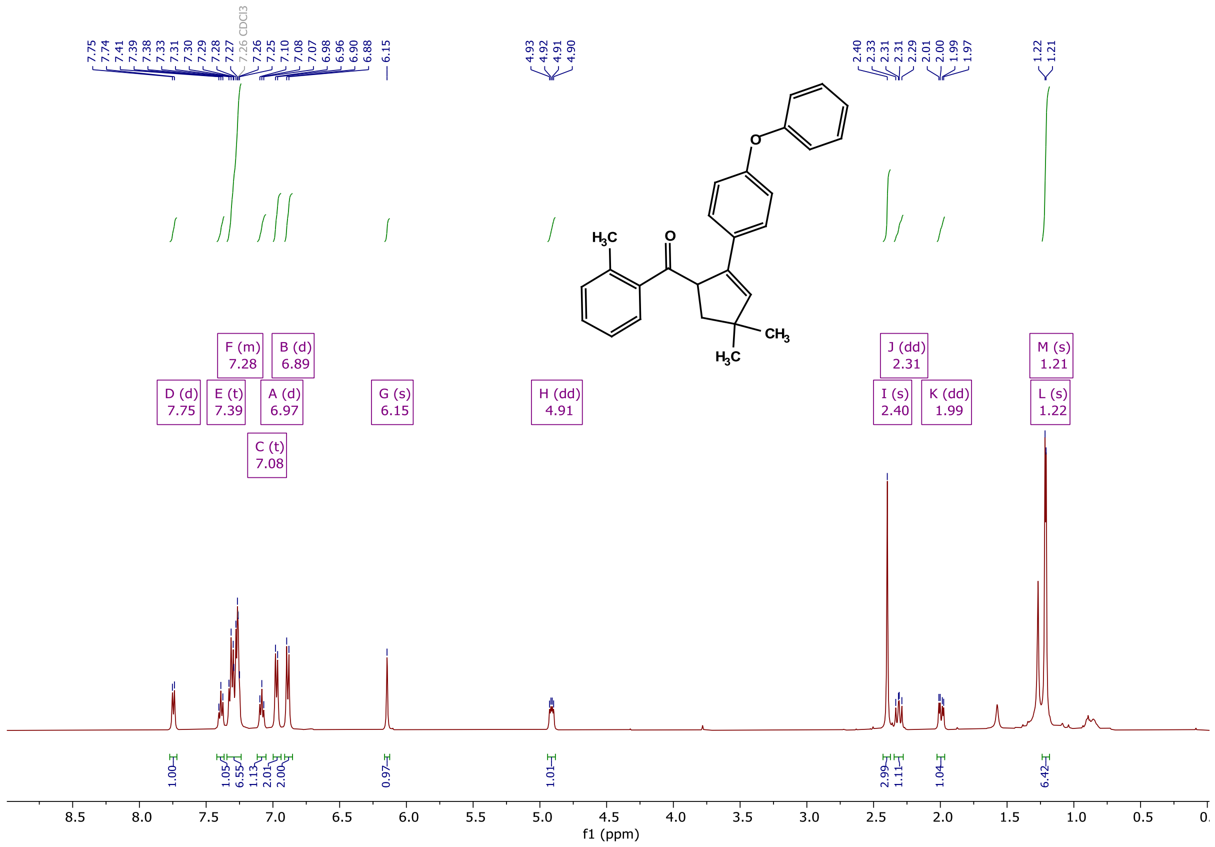

^{13}C NMR (101 MHz, Chloroform-*d*) (**3m**):

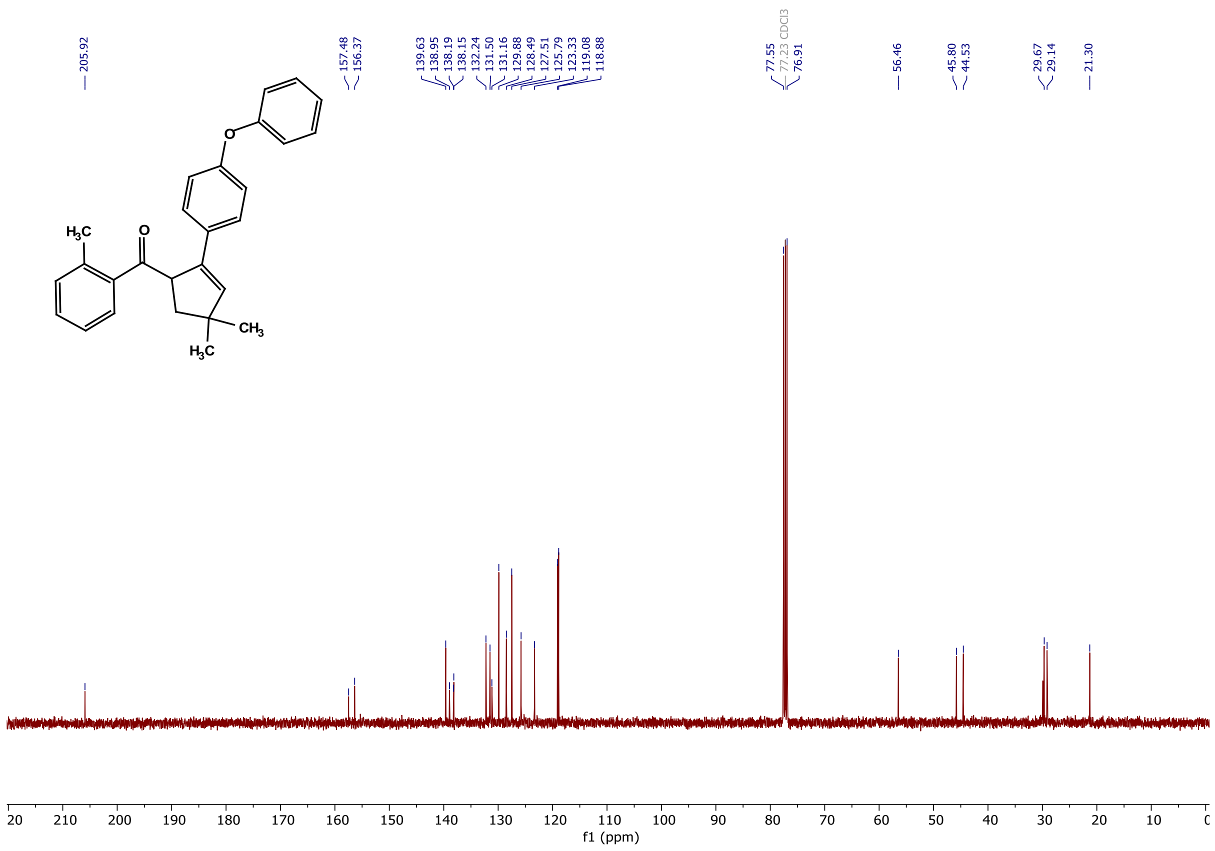

DEPT-135 NMR (101 MHz, Chloroform-*d*) (**3m**):

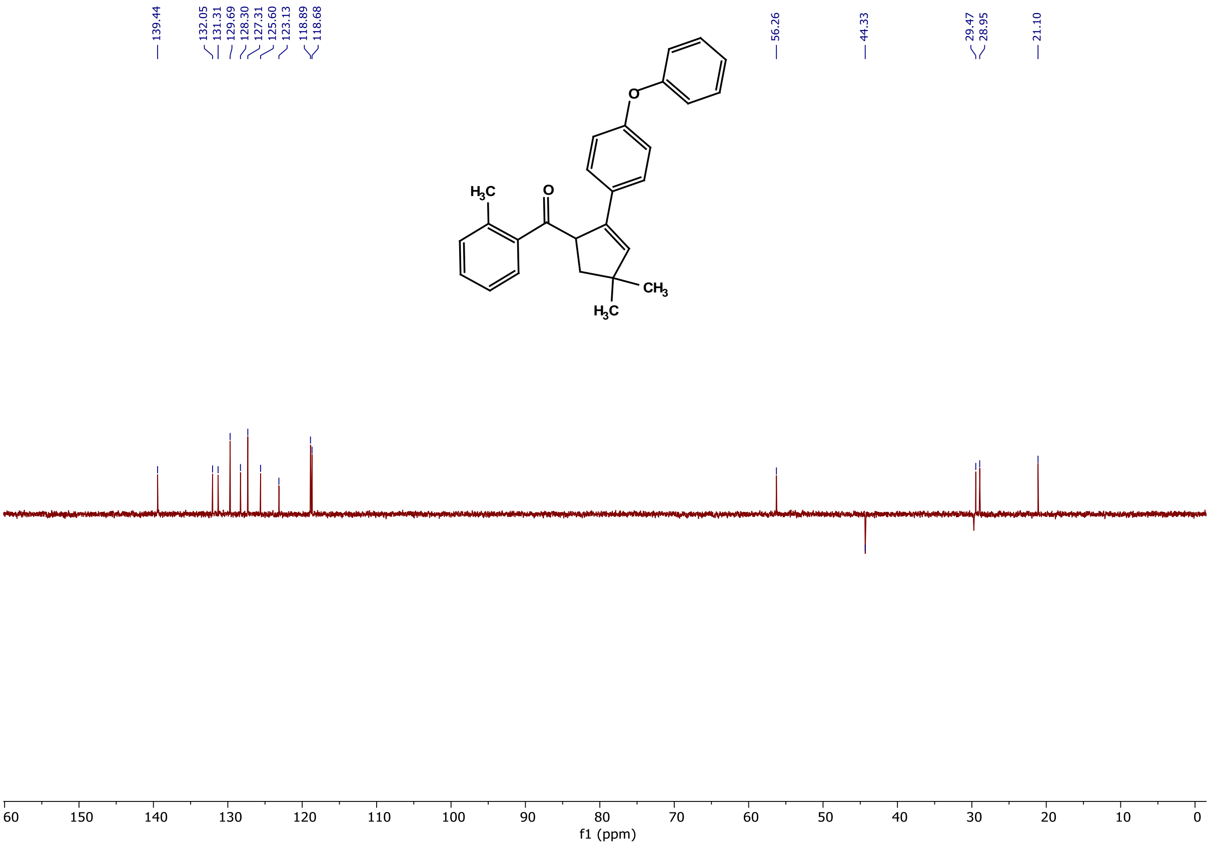

^{1}H NMR (400 MHz, Chloroform-*d*) (**3n**):

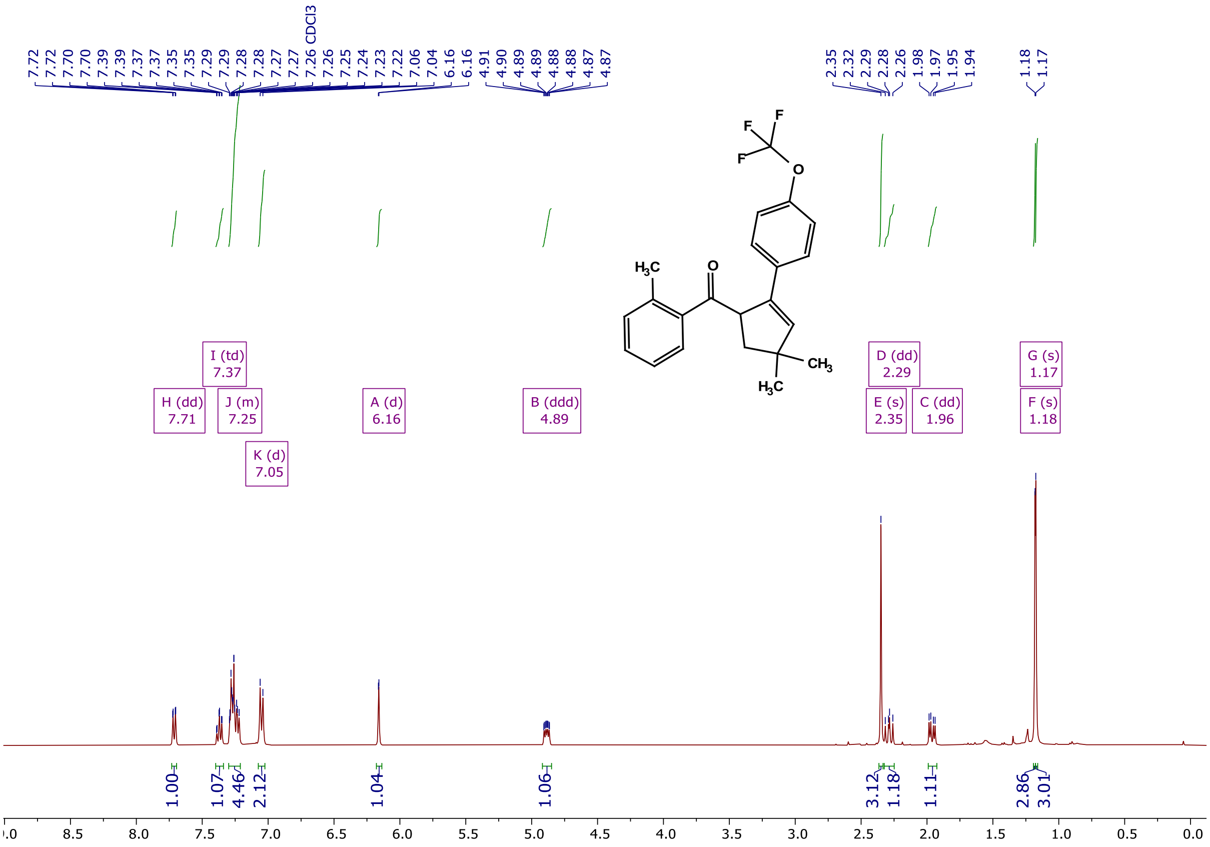

^{13}C NMR (101 MHz, Chloroform-*d*) (**3n**):

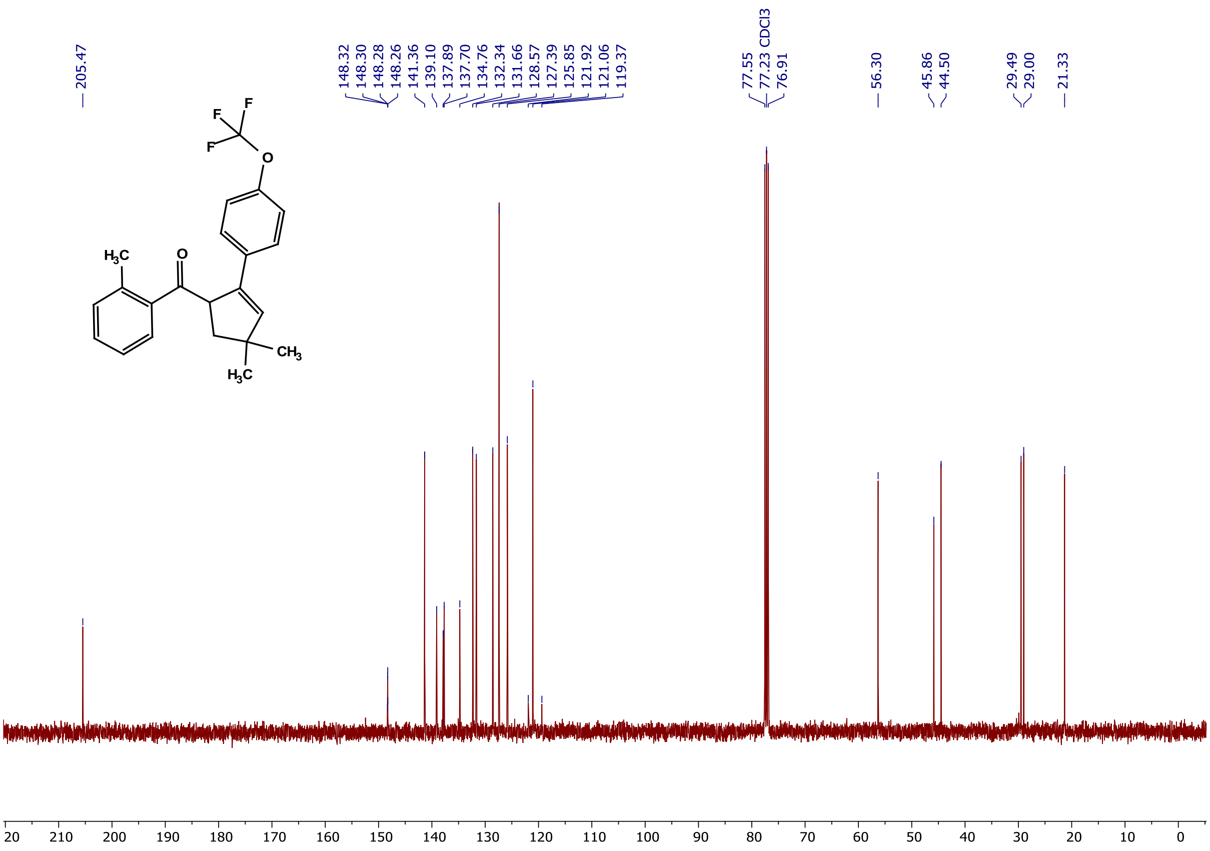

DEPT-135 NMR (101 MHz, Chloroform-*d*) (**3n**):

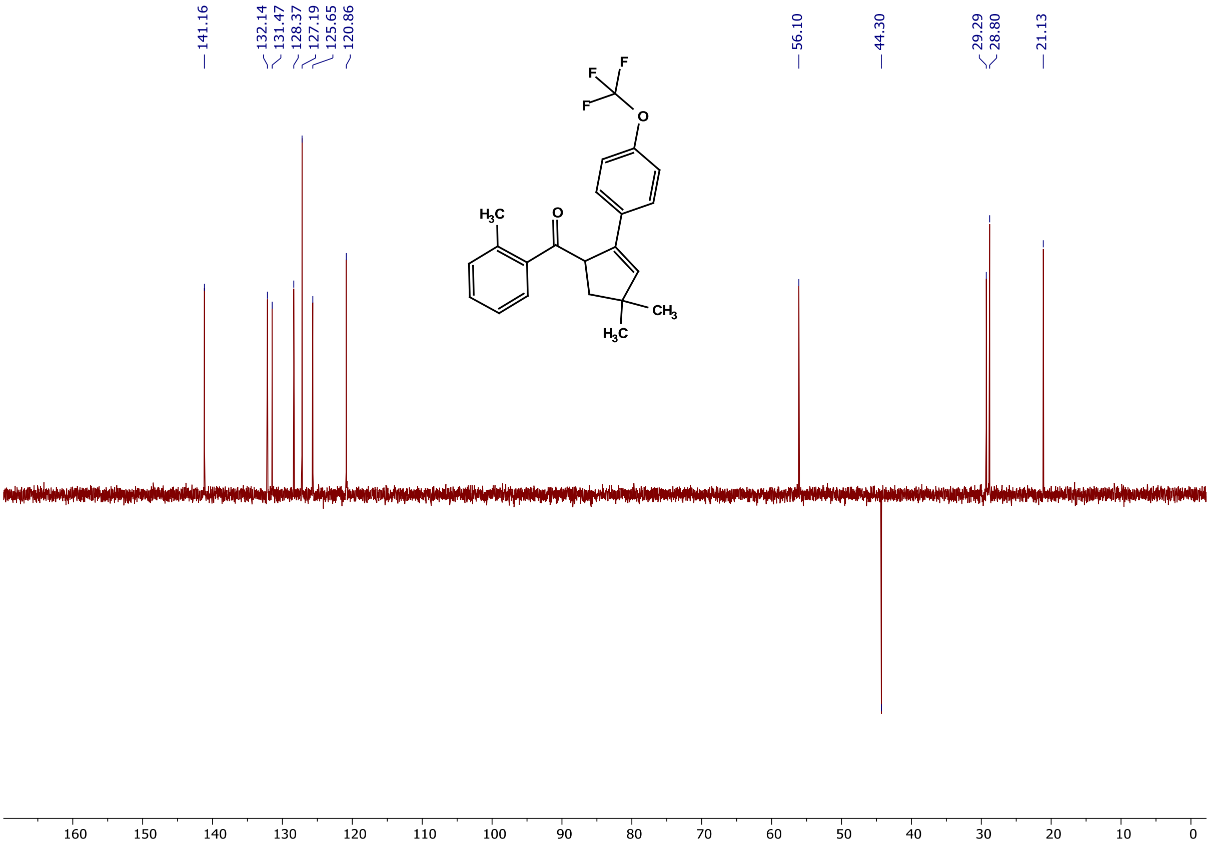

^{19}F NMR (376 MHz, Chloroform-*d*) (**3n**):

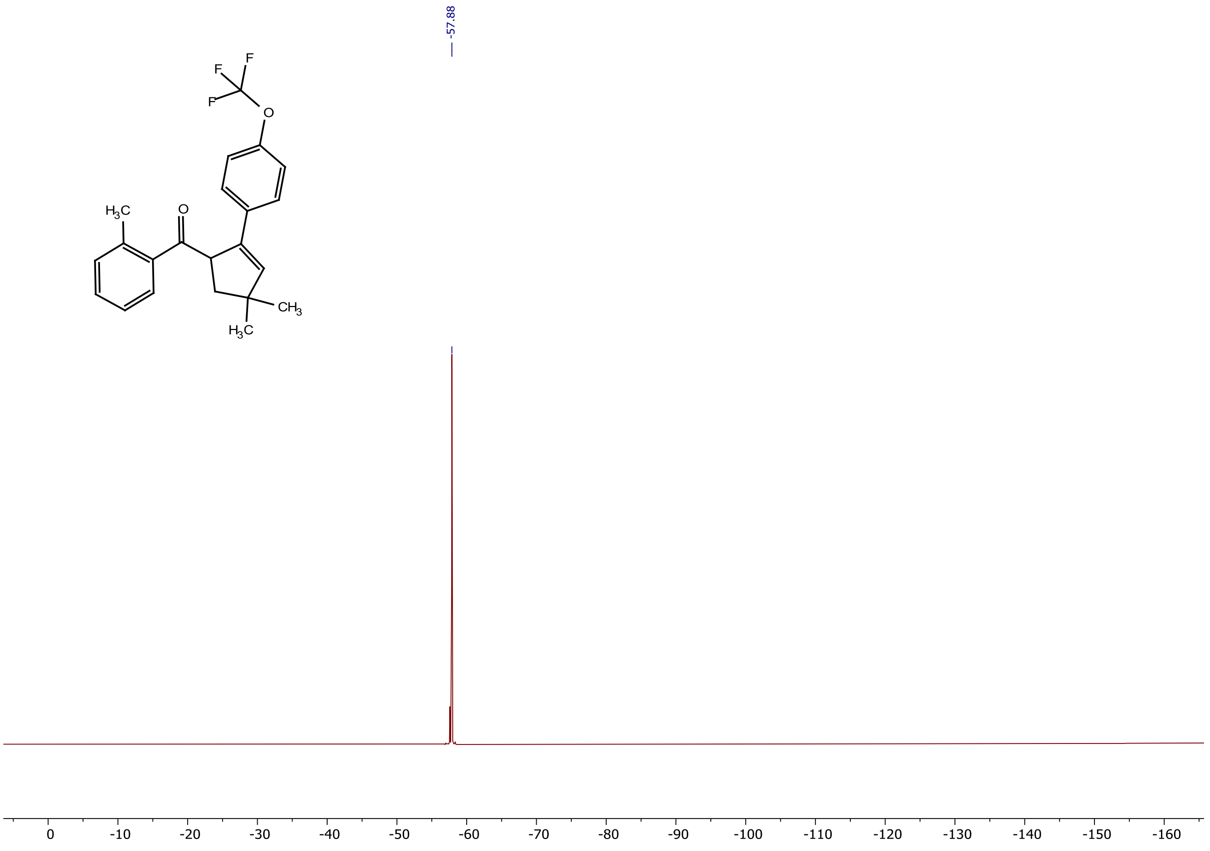

^{1}H NMR (500 MHz, Chloroform-*d*) (**3o**):

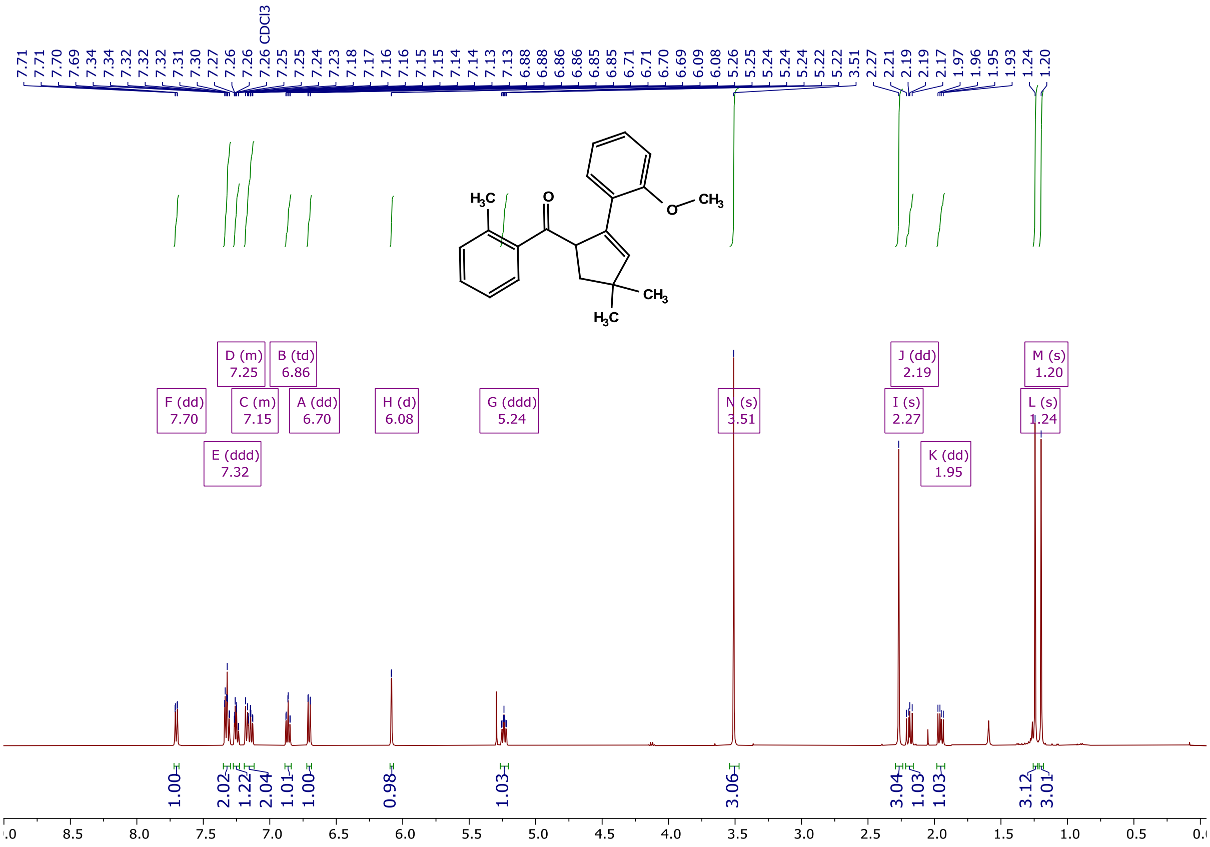

^{13}C NMR (101 MHz, Chloroform-*d*) (**3o**):

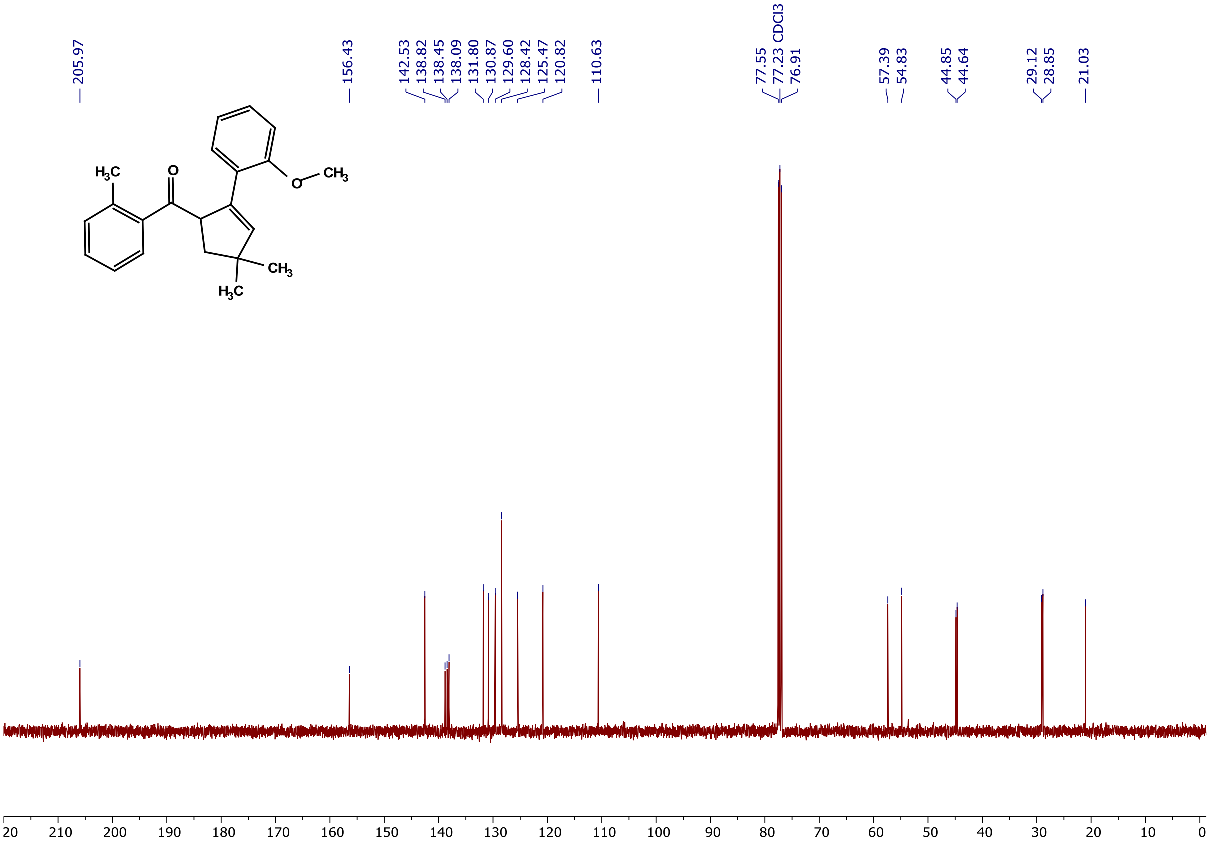

^{1}H NMR (400 MHz, Chloroform-*d*) (**3p**):

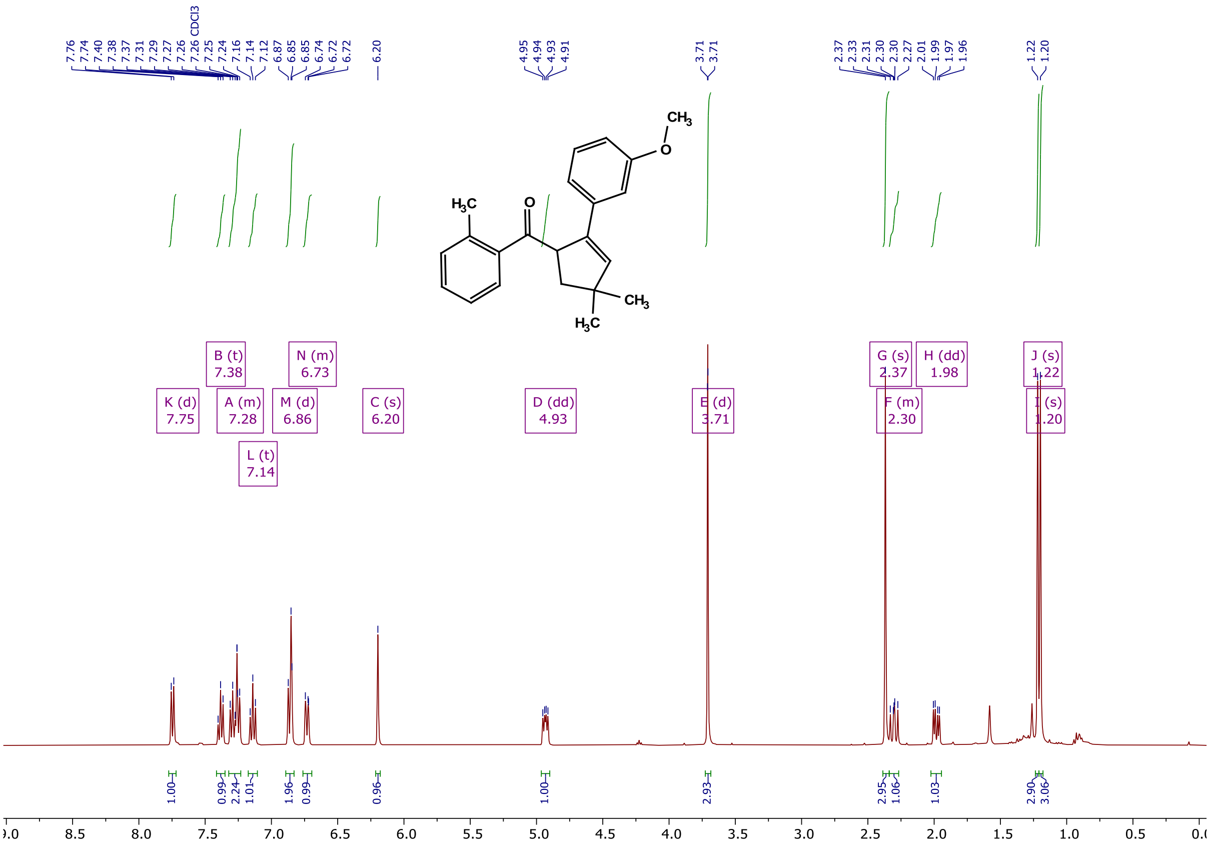

^{13}C NMR (101 MHz, Chloroform-*d*) (**3p**):

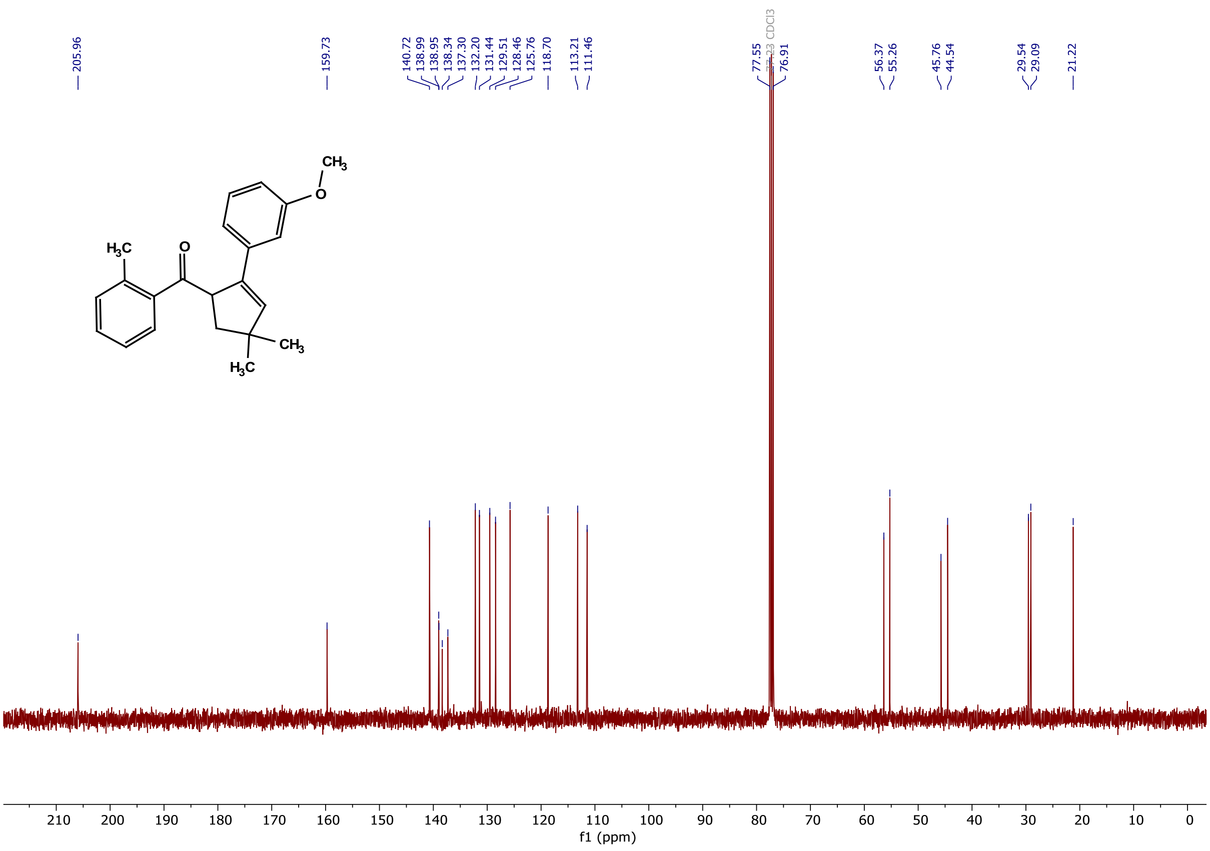

DEPT-135 NMR (101 MHz, Chloroform-*d*) (**3p**):

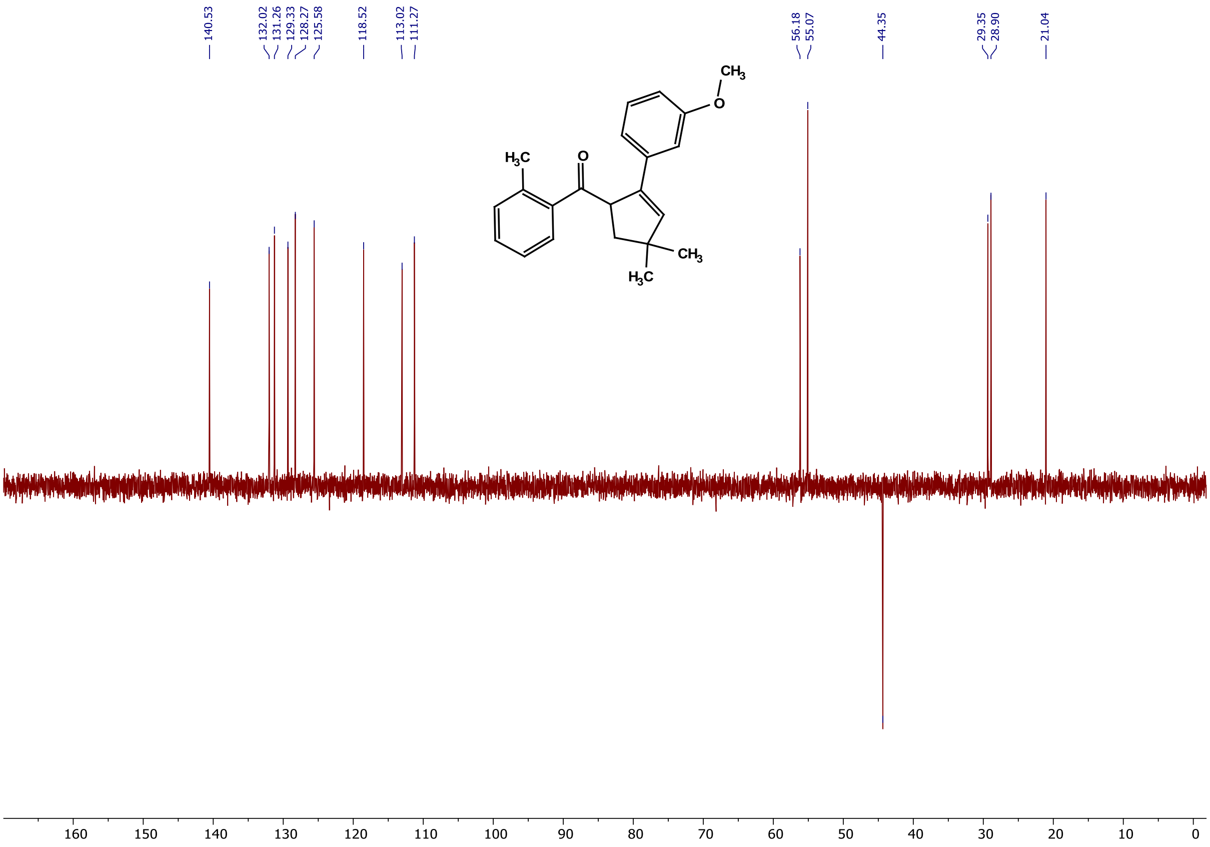

^{1}H NMR (400 MHz, Chloroform-*d*) (**3q**):

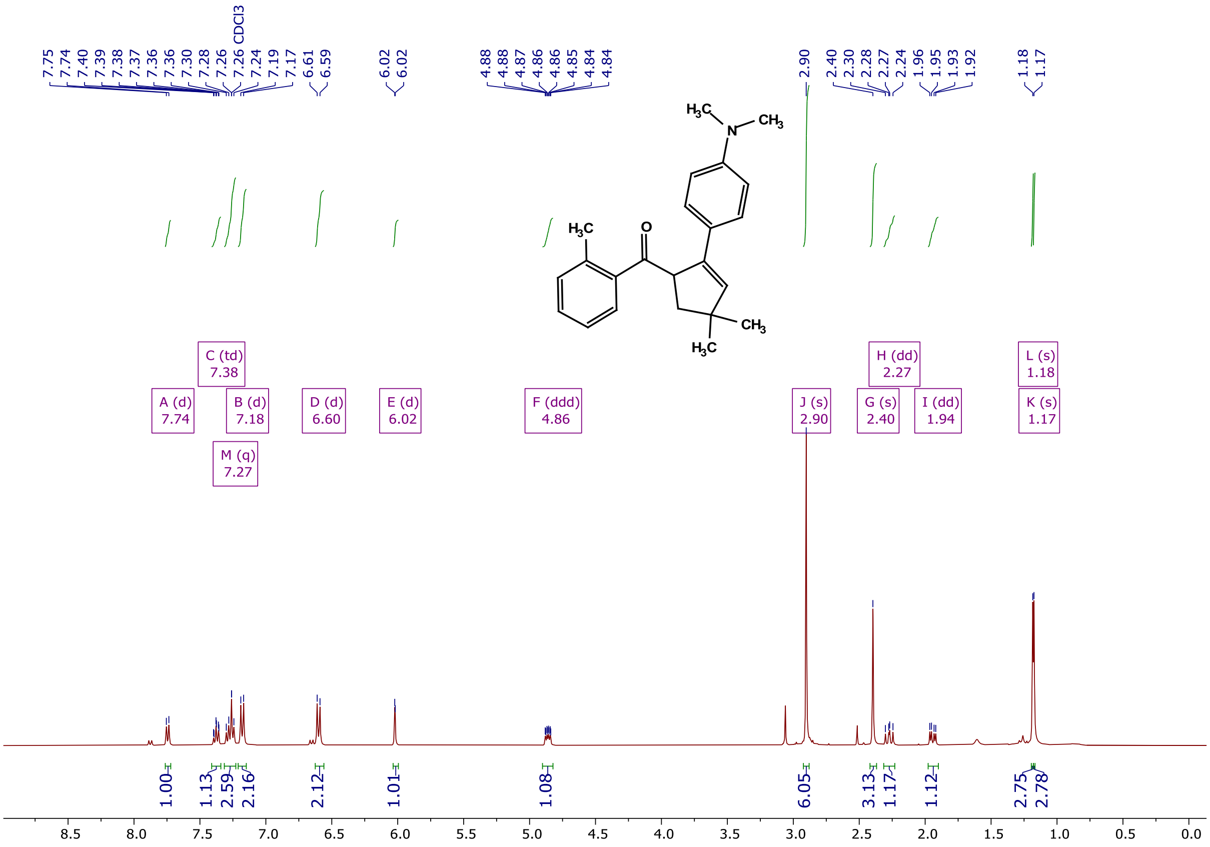

^{13}C NMR (101 MHz, Chloroform-*d*) (**3q**):

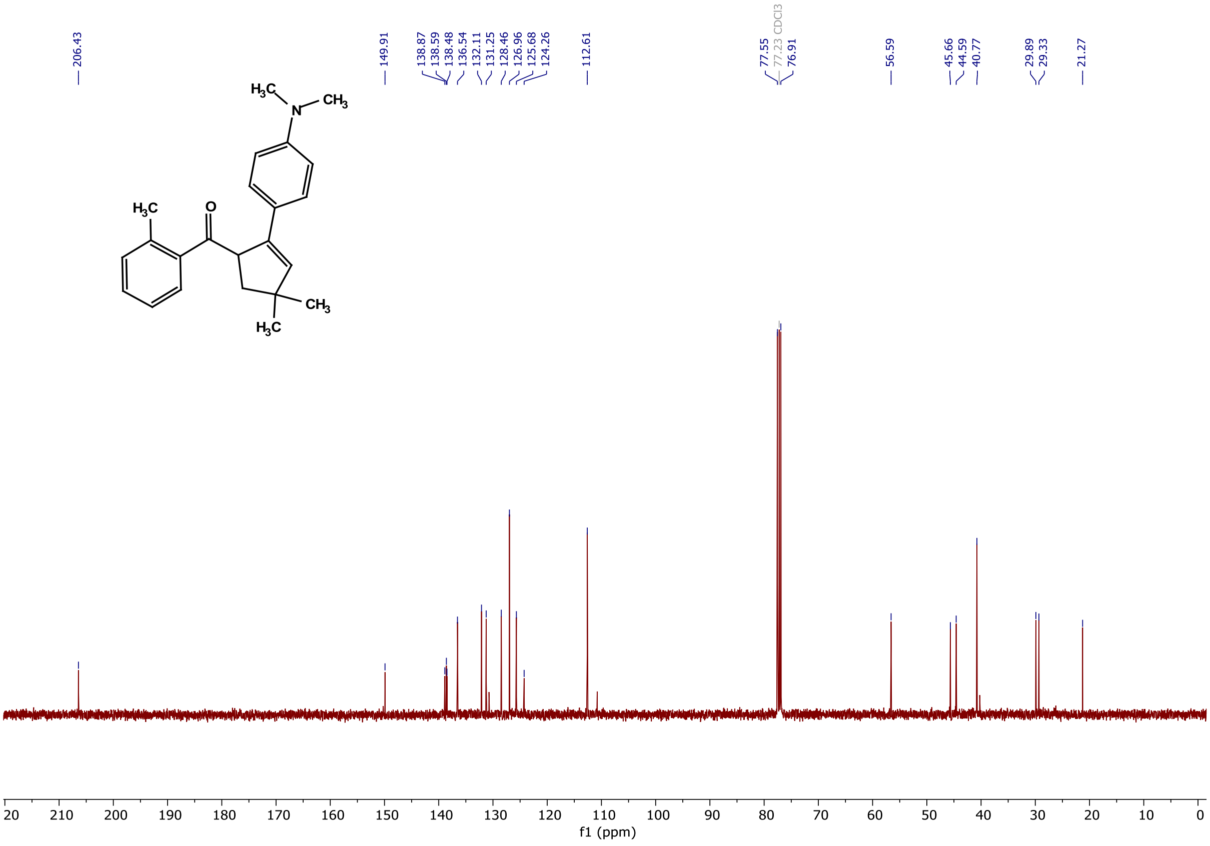

DEPT-135 NMR (101 MHz, Chloroform-*d*) (**3q**):

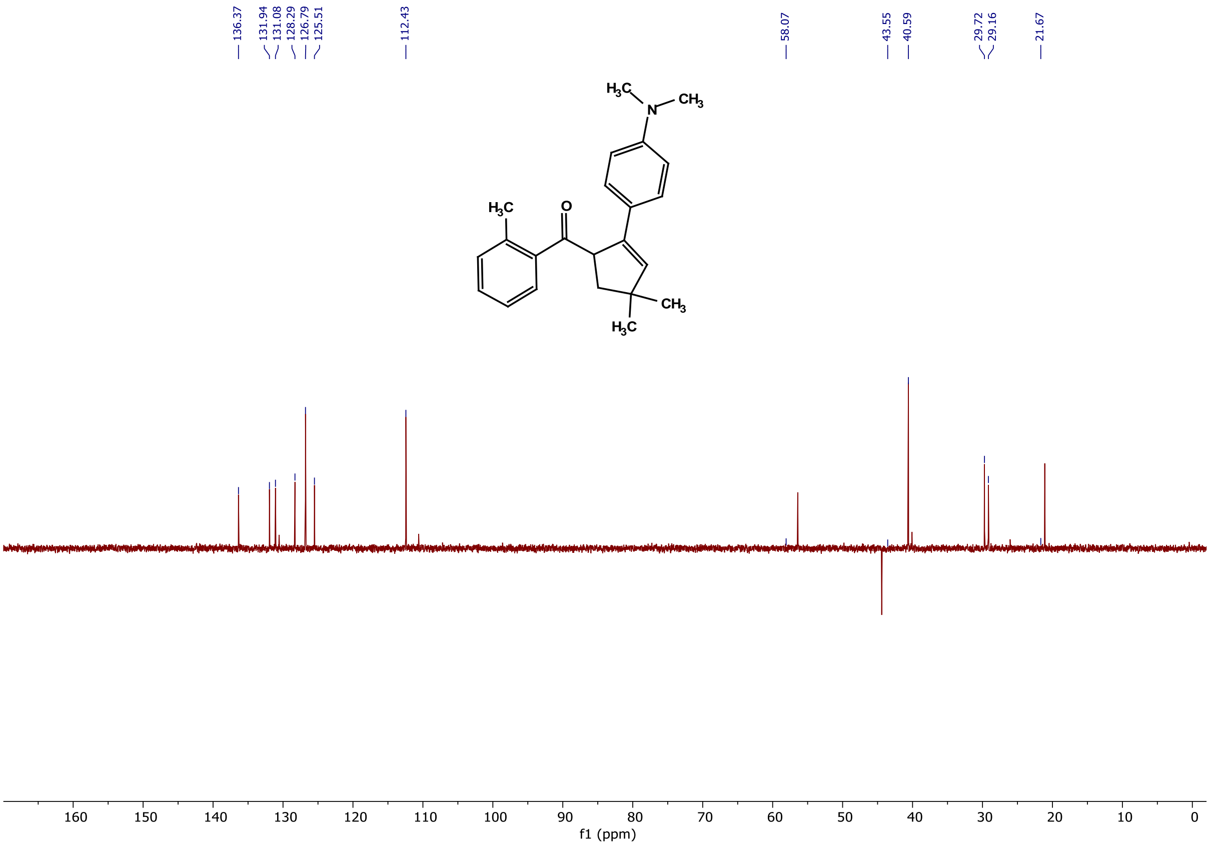

^{1}H NMR (400 MHz, Chloroform-*d*) (**3r**):

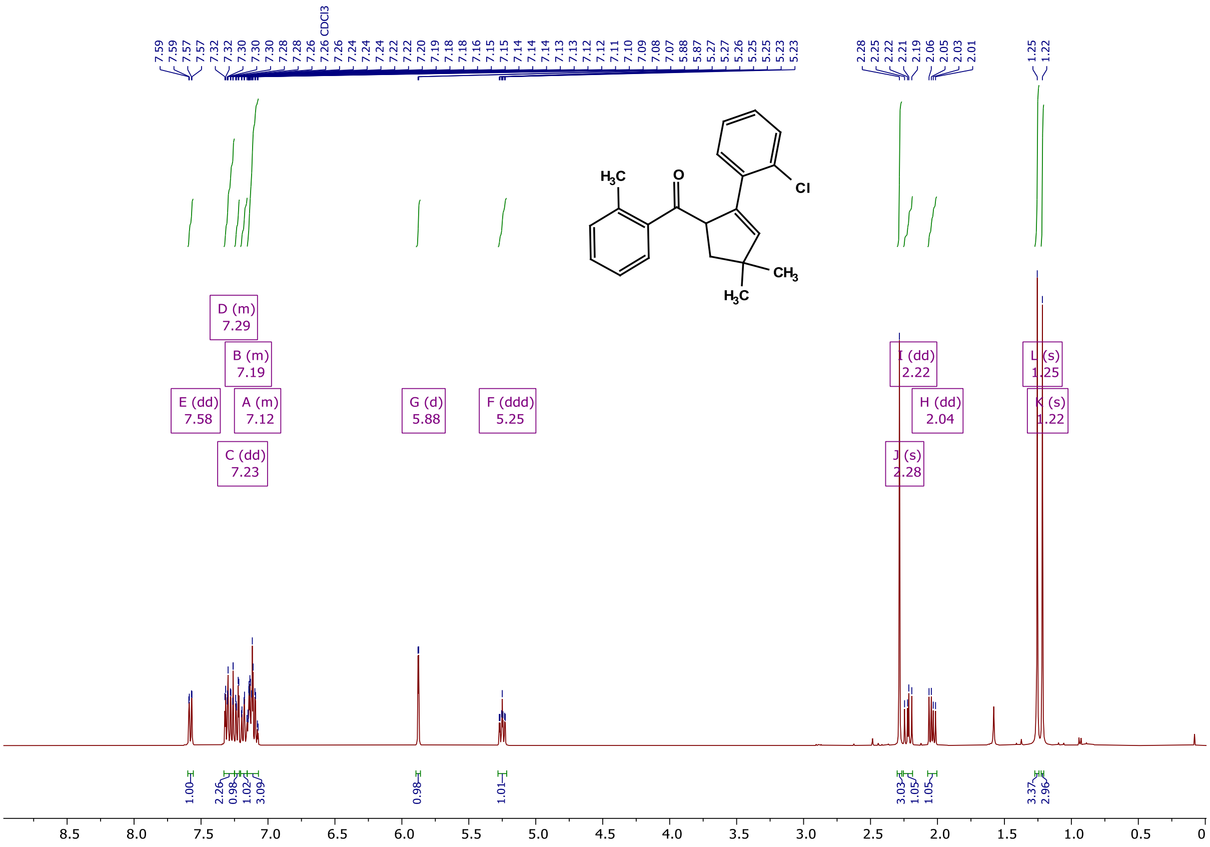

^{13}C NMR (101 MHz, Chloroform-*d*) (**3r**):

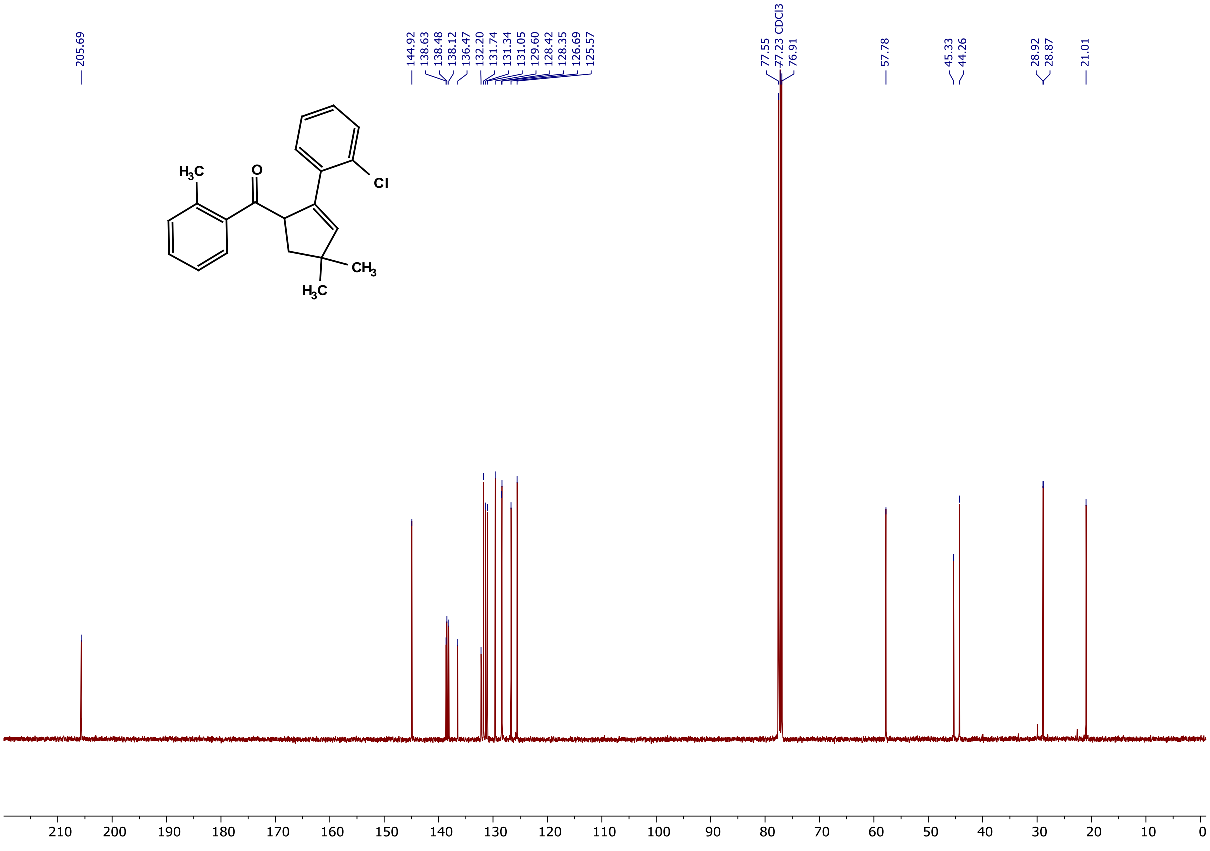

DEPT-135 NMR (101 MHz, Chloroform-*d*) (**3r**):

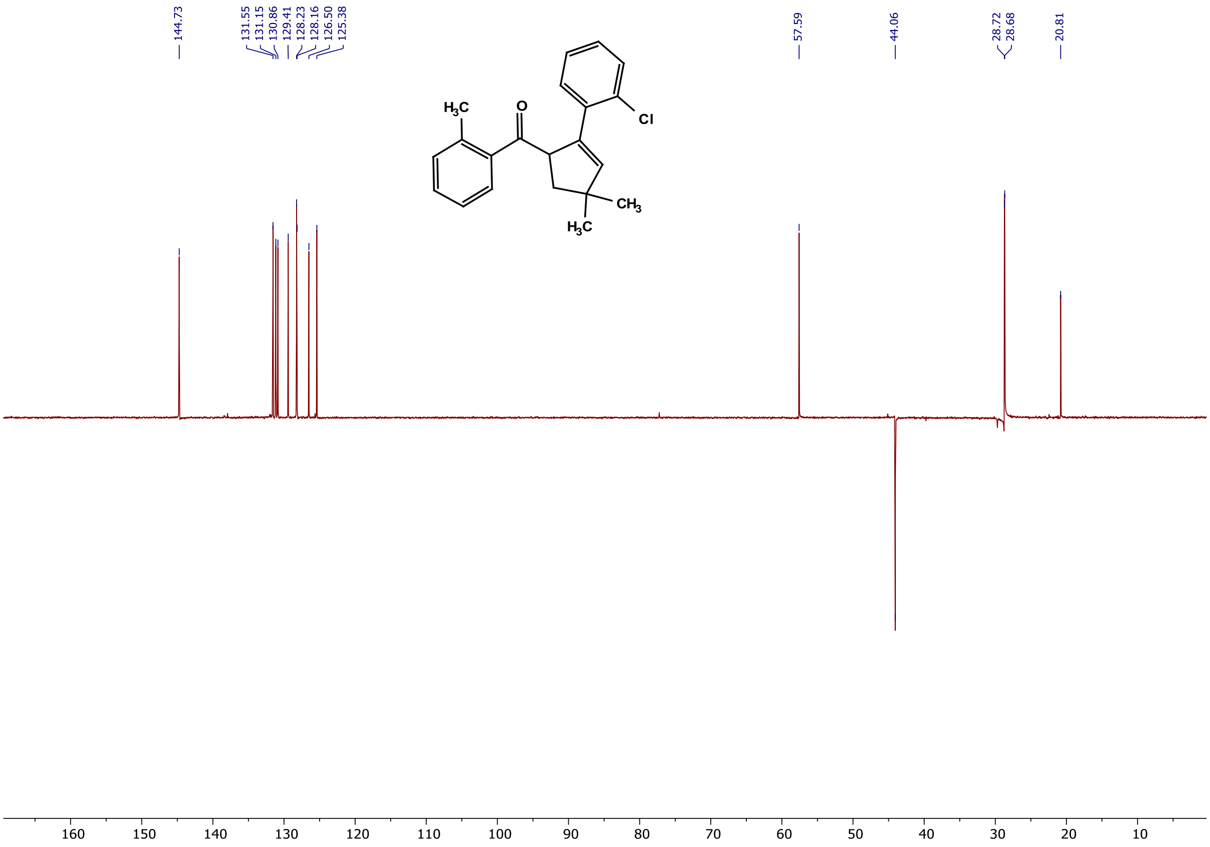

^{1}H NMR (400 MHz, Chloroform-*d*) (**3s**):

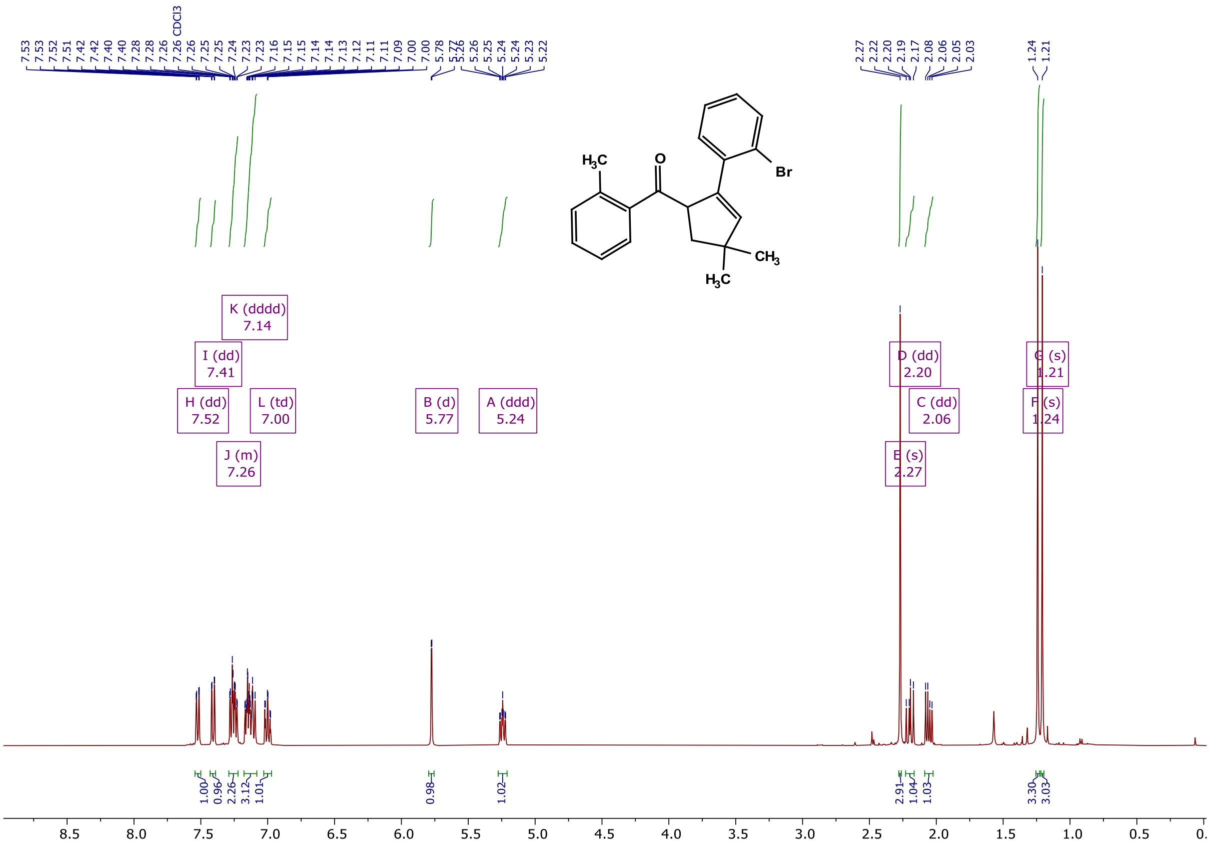

^{13}C NMR (101 MHz, Chloroform-*d*) (**3s**):

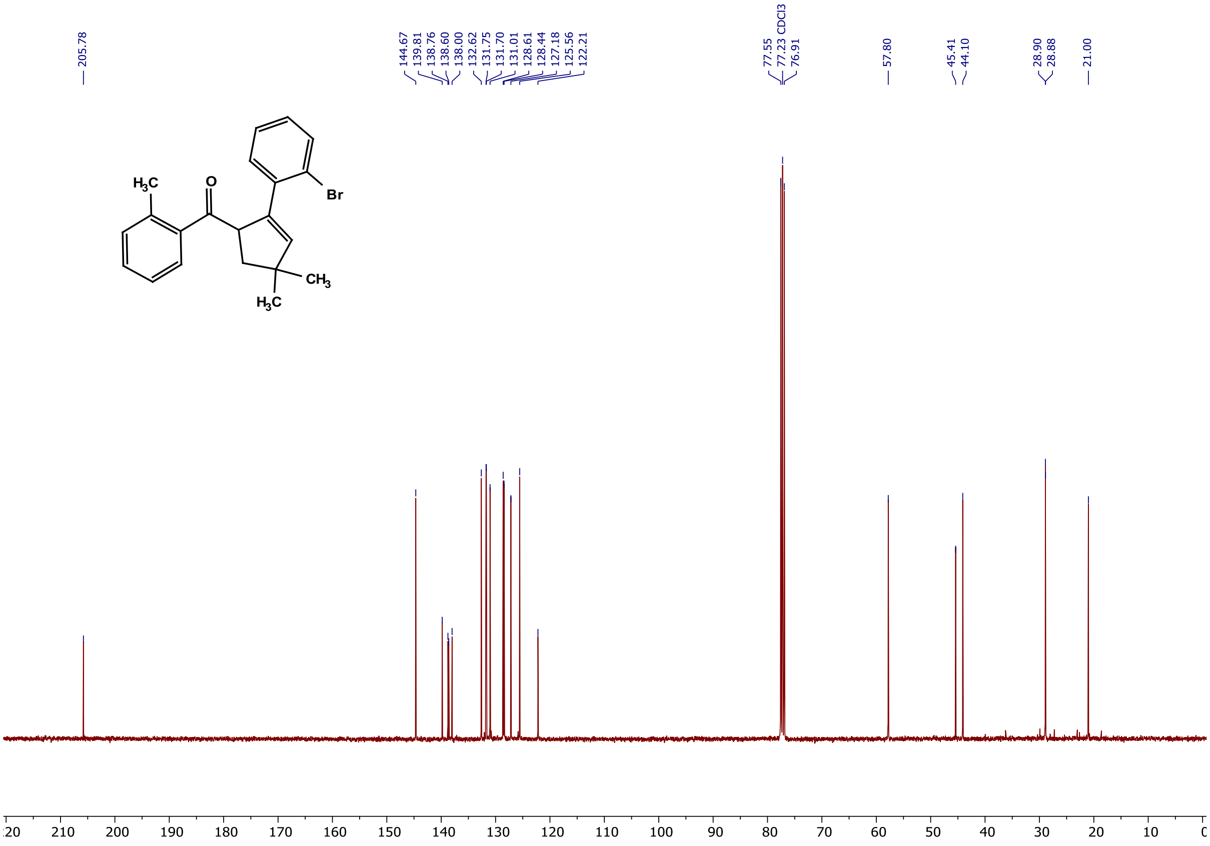

DEPT-135 NMR (101 MHz, Chloroform-*d*) (**3s**):

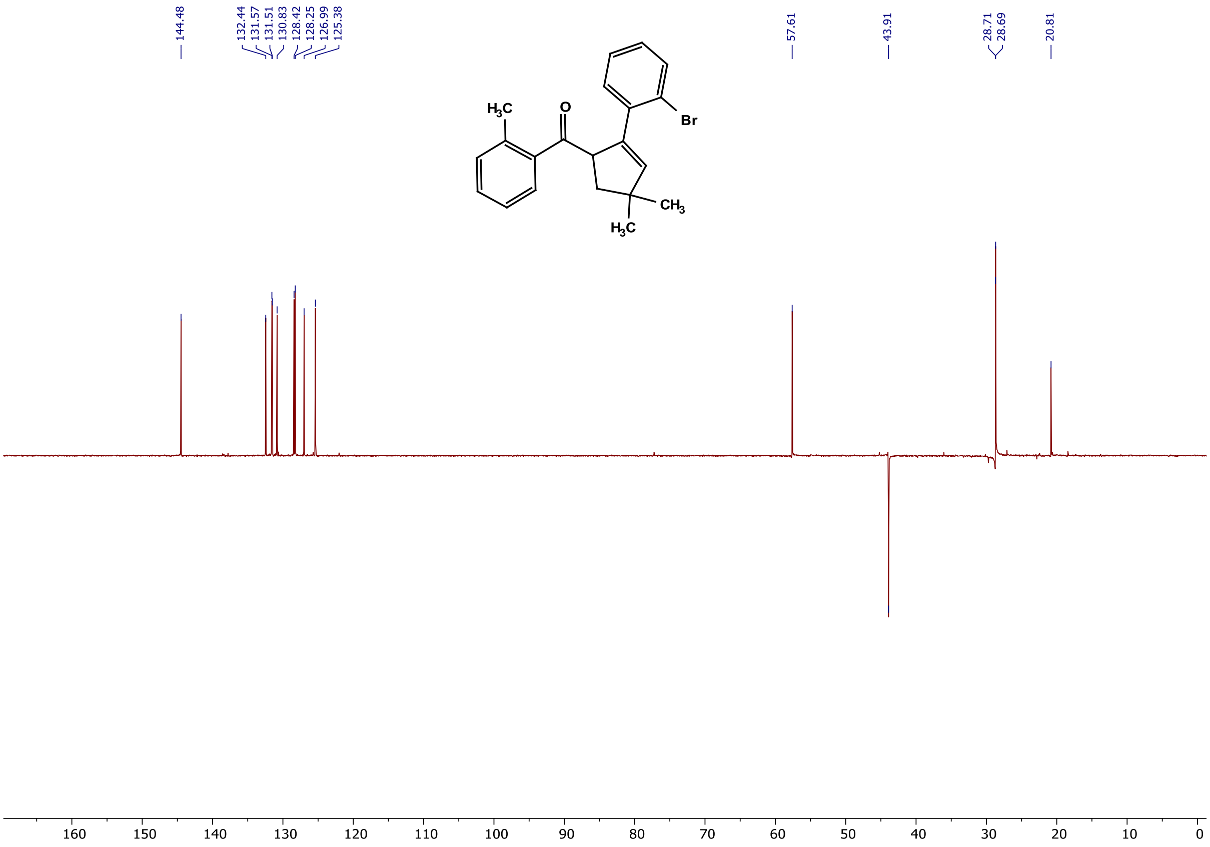

^{1}H NMR (400 MHz, Chloroform-*d*) (**3t**):

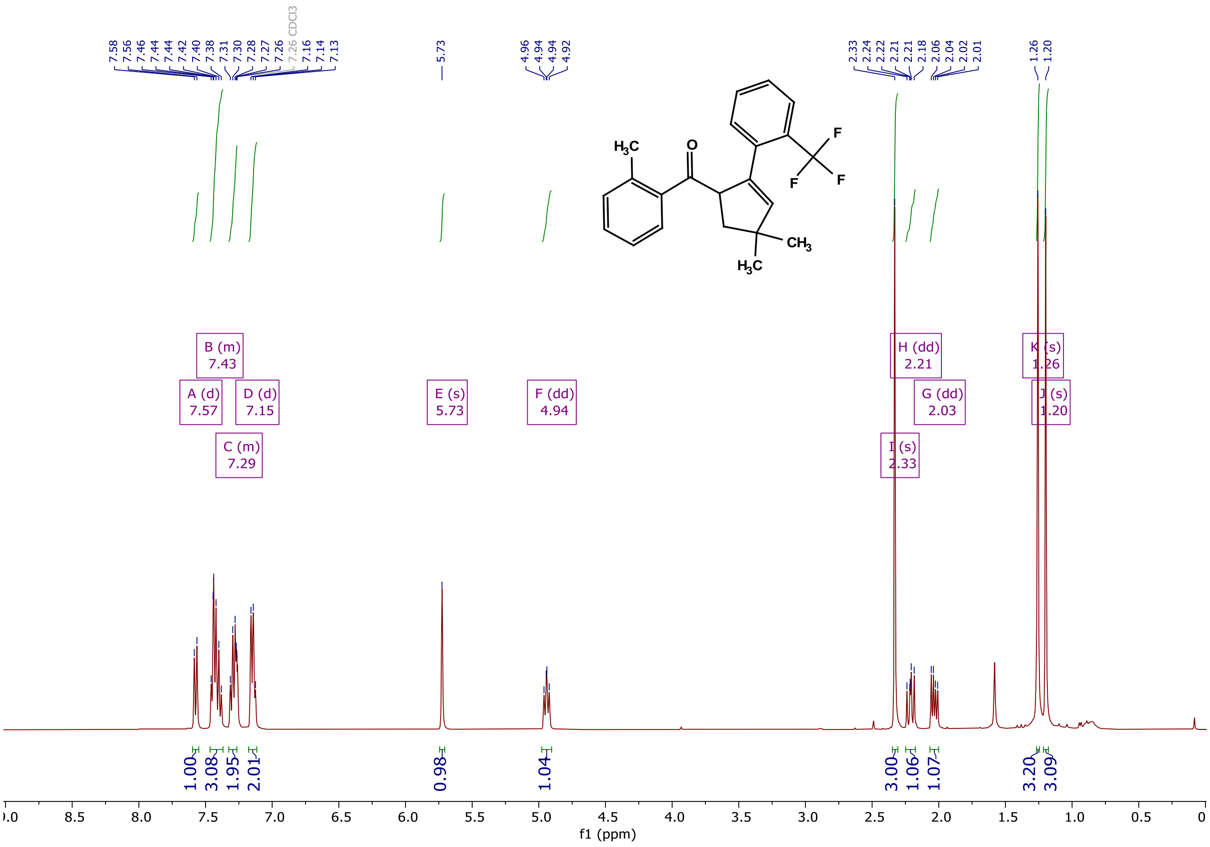

^{13}C NMR (101 MHz, Chloroform-*d*) (**3t**):

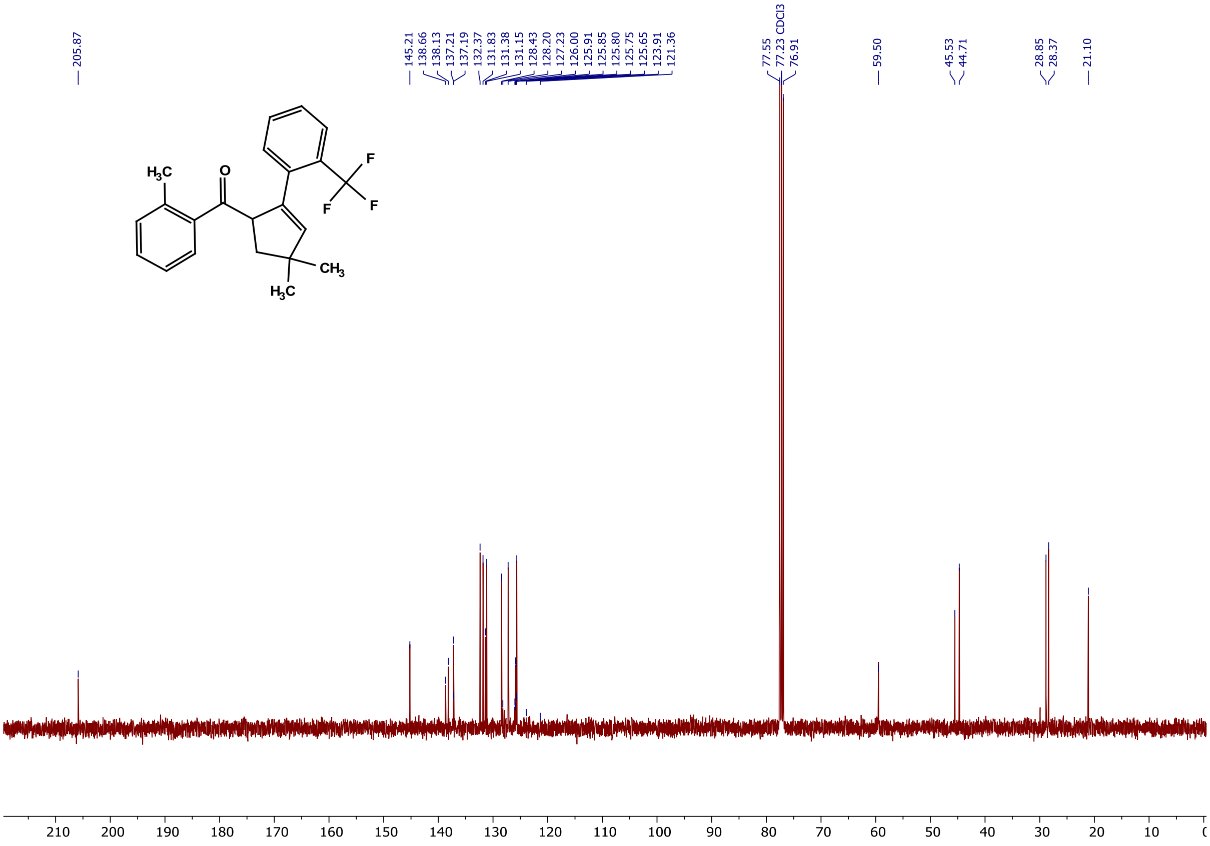

DEPT-135 NMR (101 MHz, Chloroform-*d*) (**3t**):

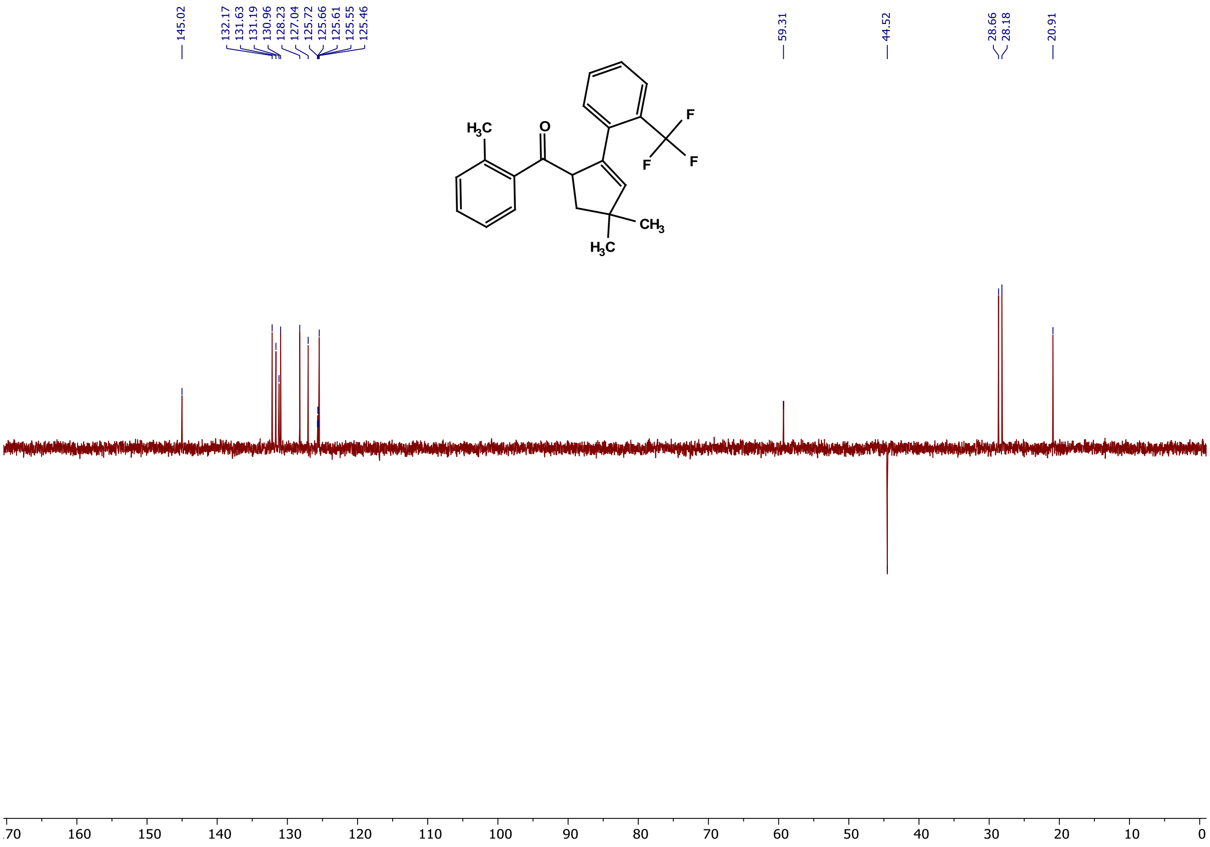

^{19}F NMR (376 MHz, Chloroform-*d*) (**3t**):

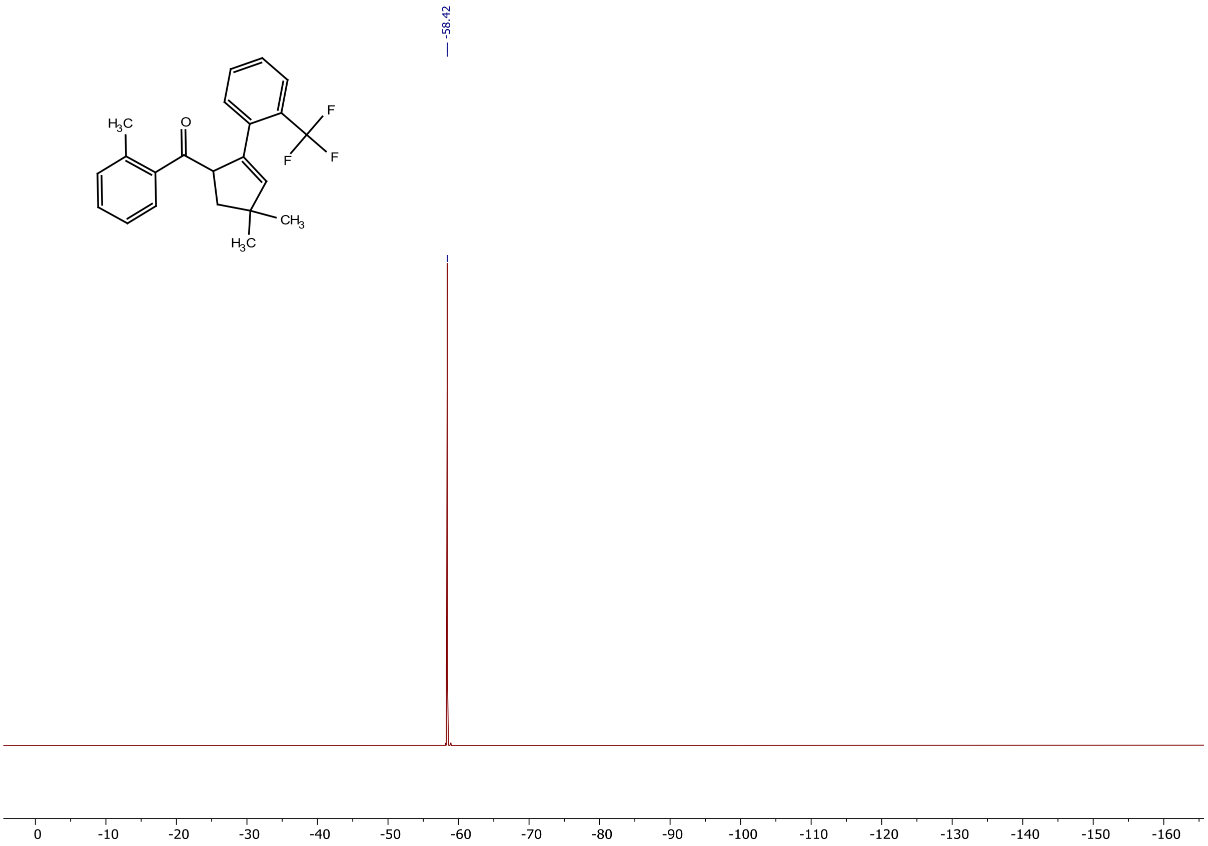

^{1}H NMR (400 MHz, Chloroform-*d*) (**3u**):

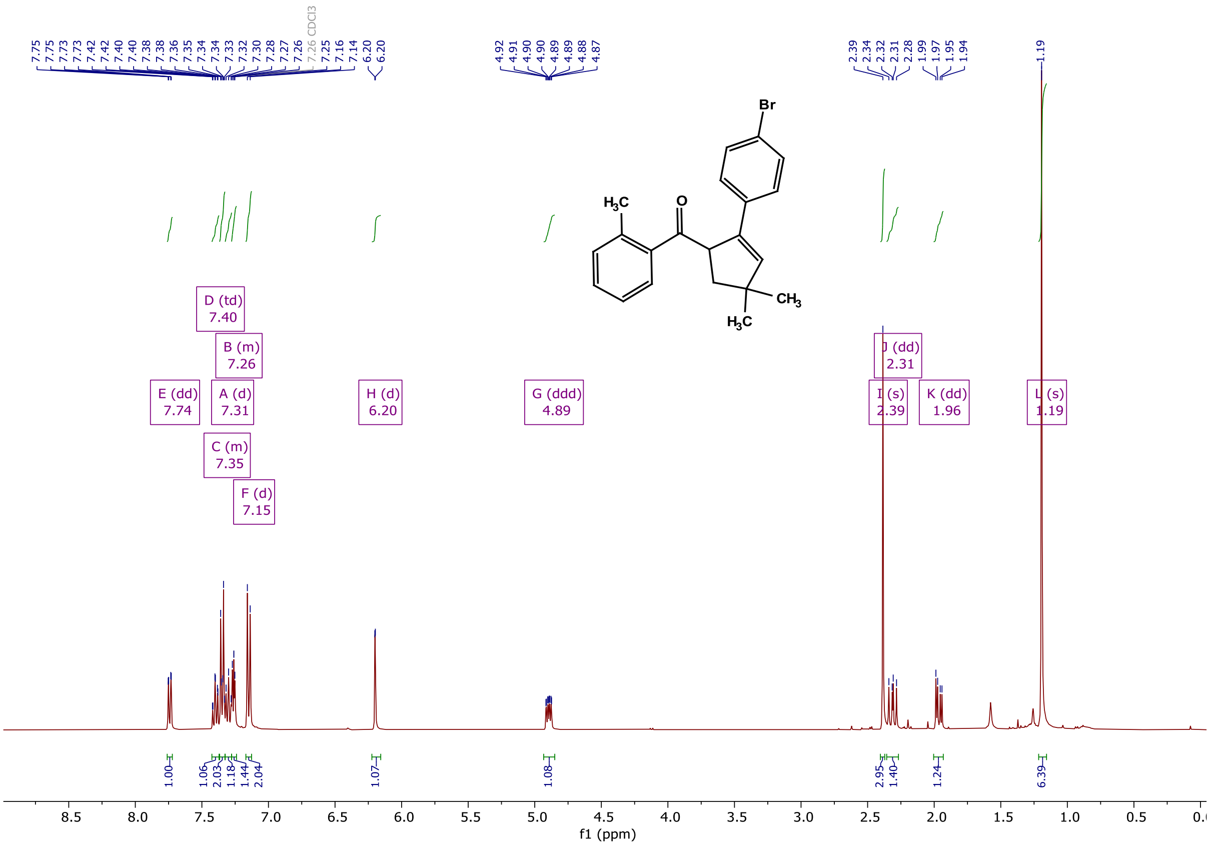

^{13}C NMR (101 MHz, Chloroform-*d*) (**3u**):

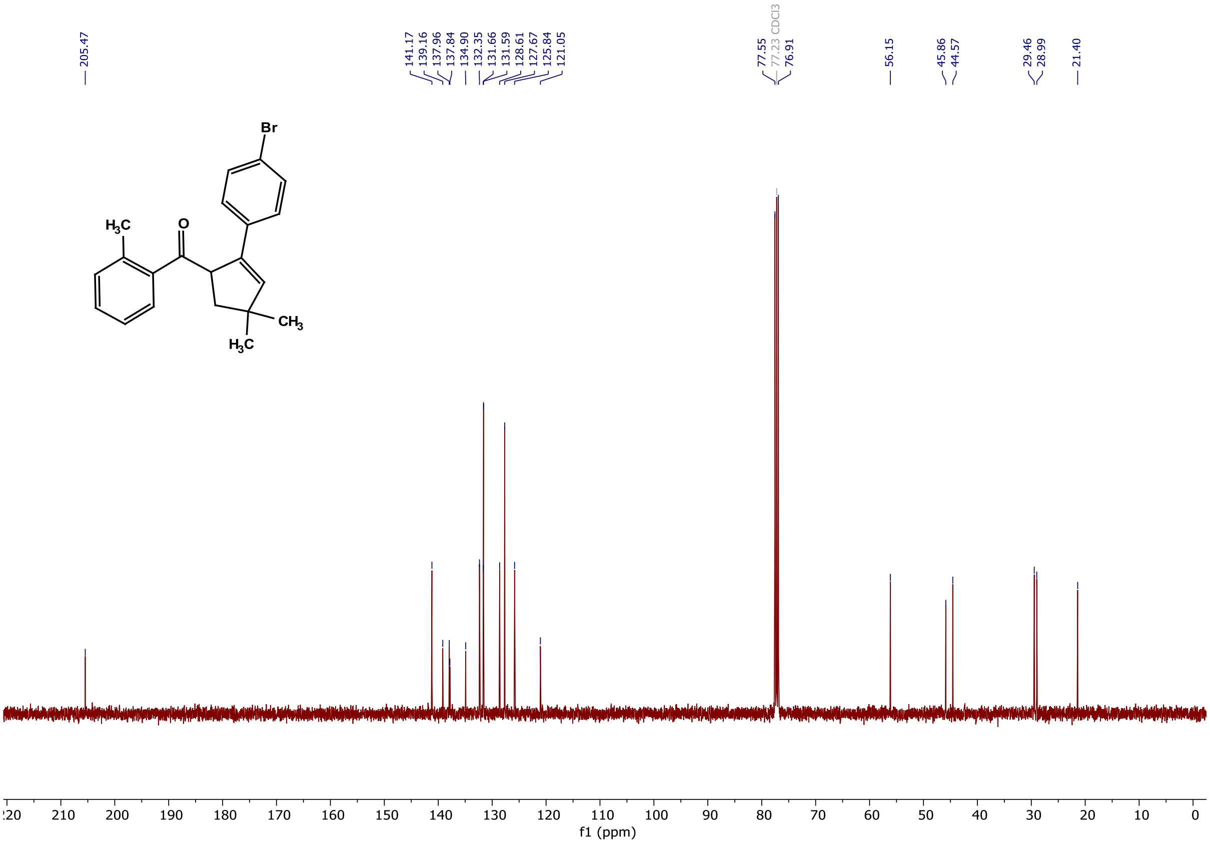

DEPT-135 NMR (101 MHz, Chloroform-*d*) (**3u**):

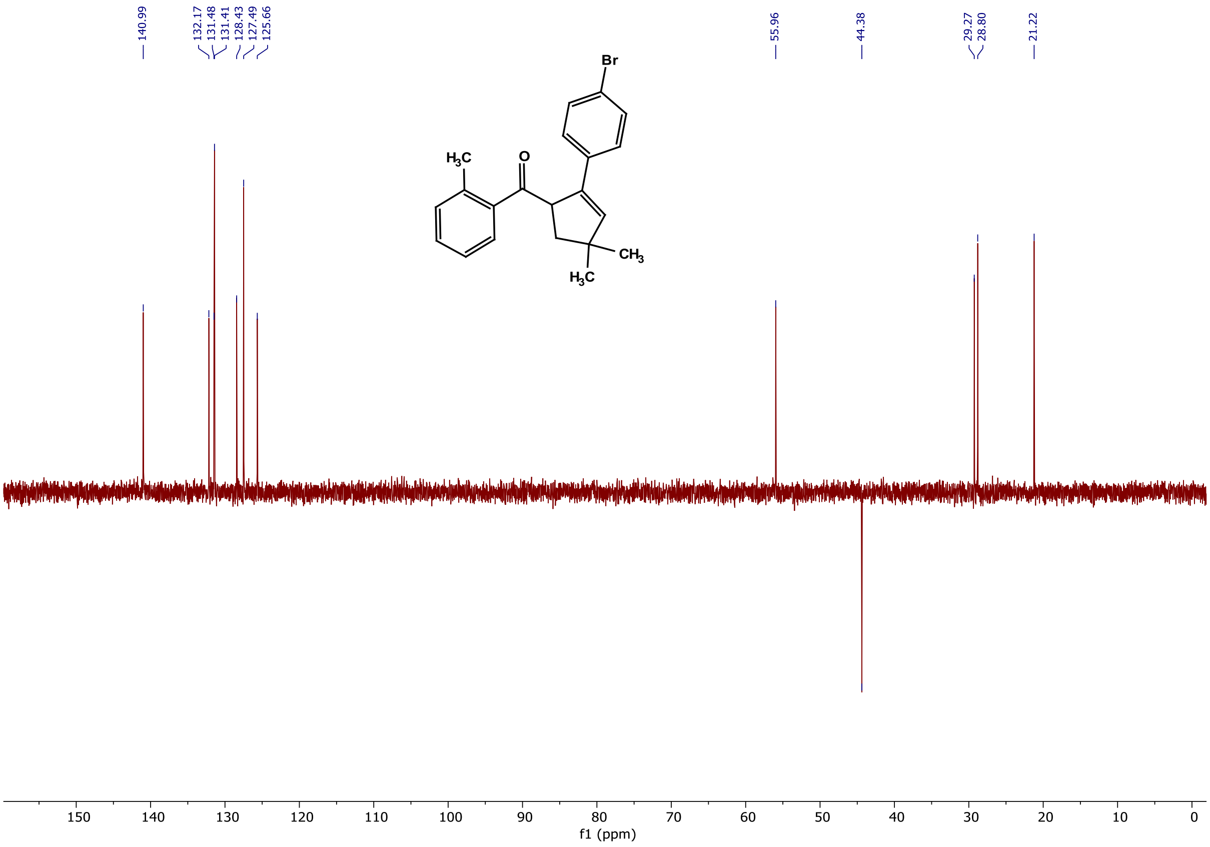

^{1}H NMR (400 MHz, Chloroform-*d*) (**3v**):

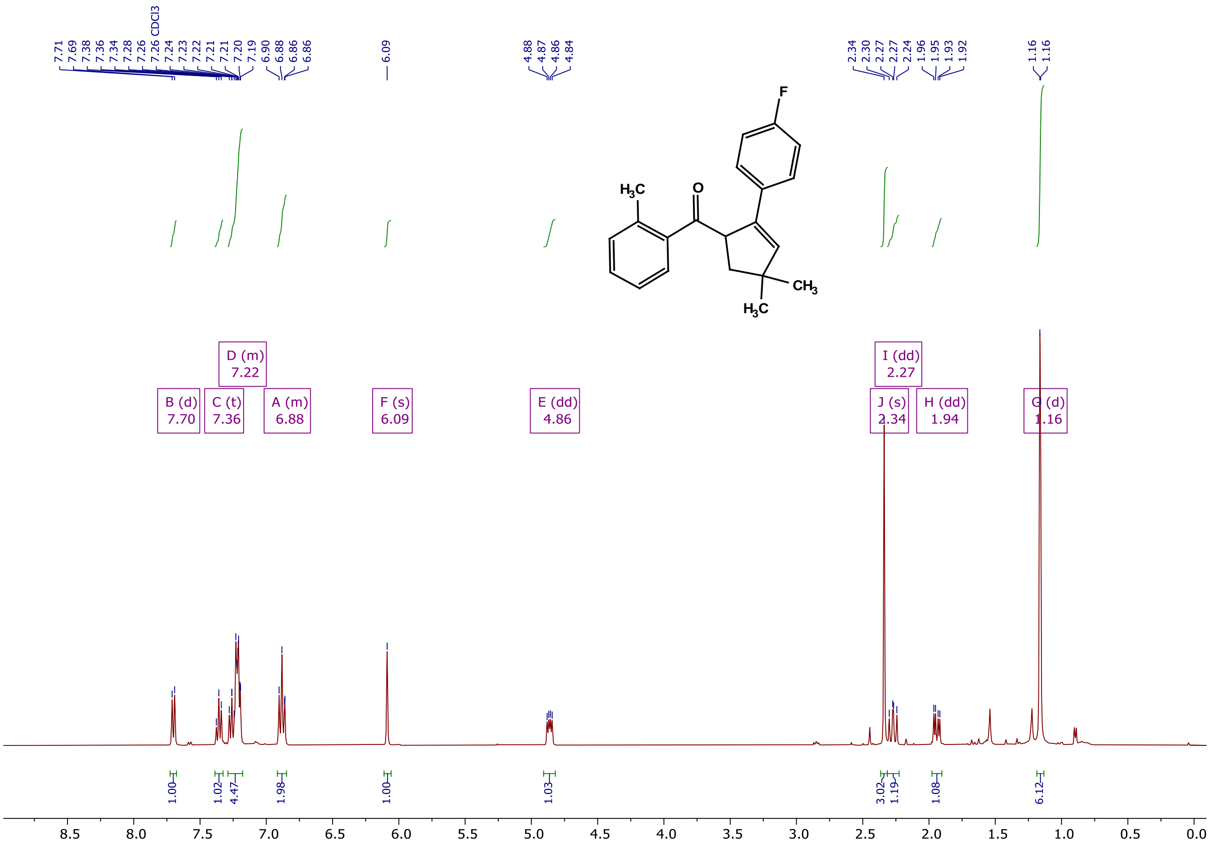

^{13}C NMR (101 MHz, Chloroform-*d*) (**3v**):

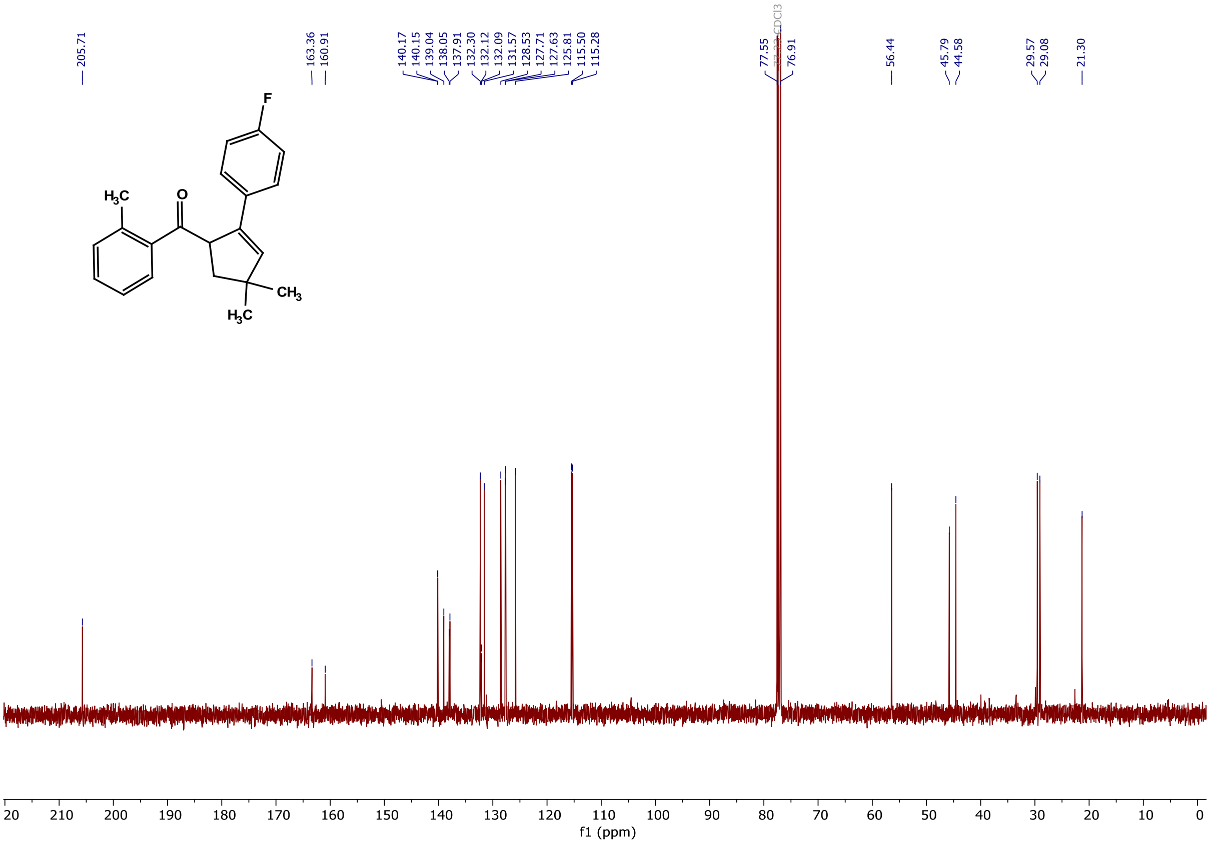

DEPT-135 NMR (101 MHz, Chloroform-*d*) (**3v**):

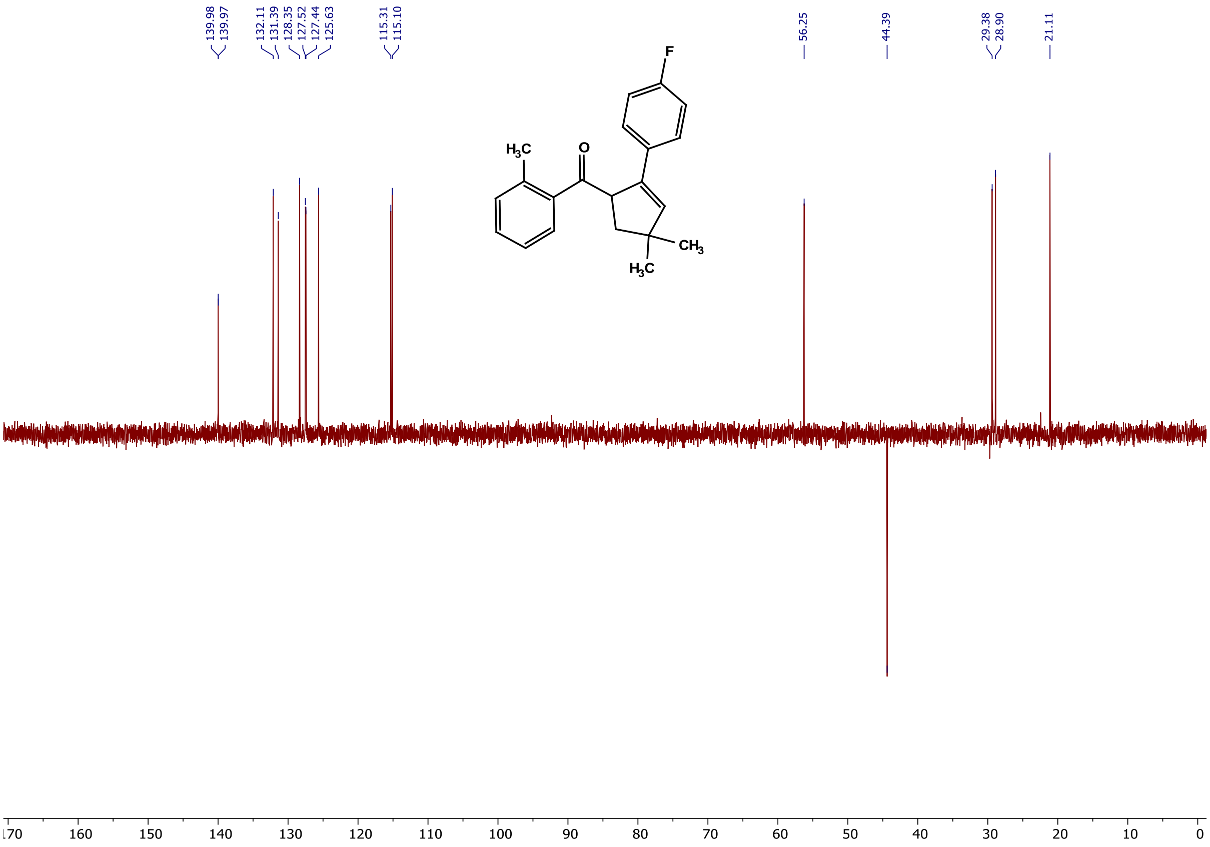

^{19}F NMR (376 MHz, Chloroform-*d*) (**3v**):

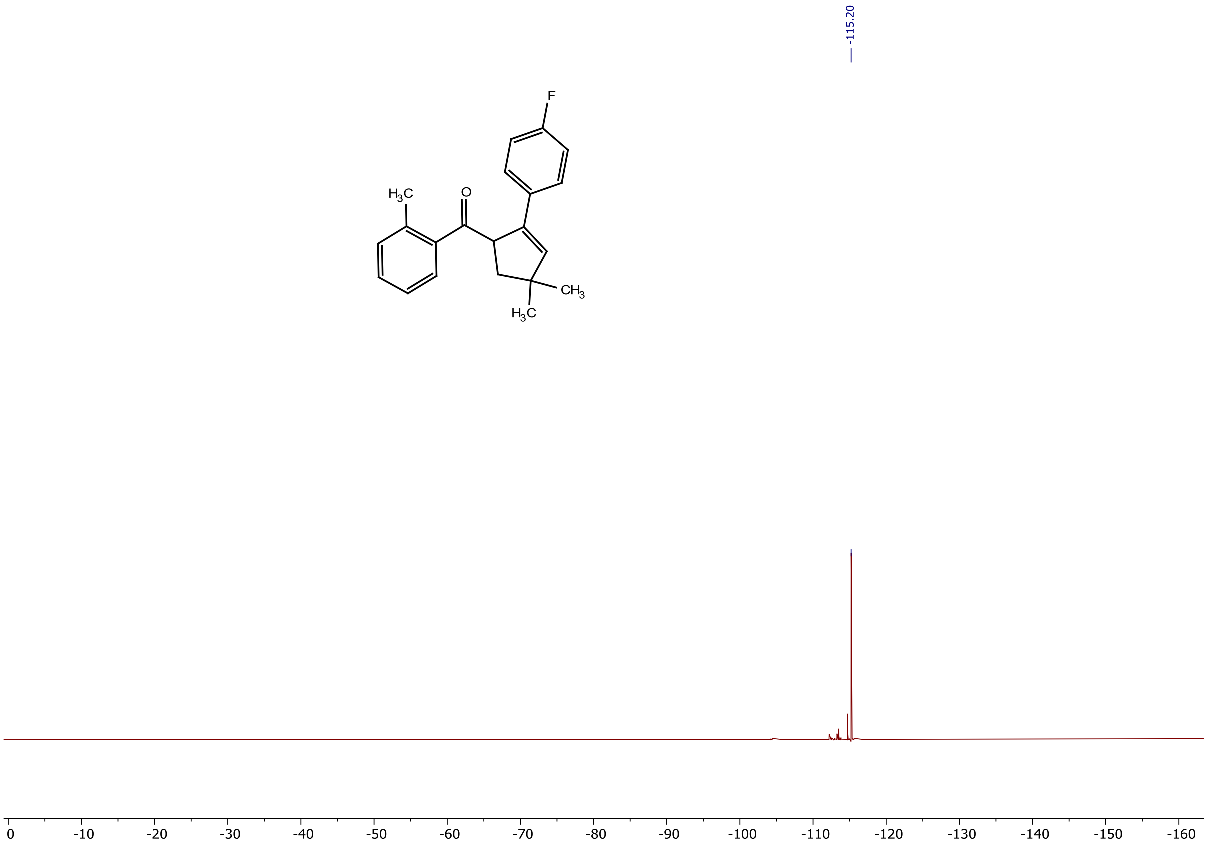

^{1}H NMR (400 MHz, Chloroform-*d*) (**3w**):

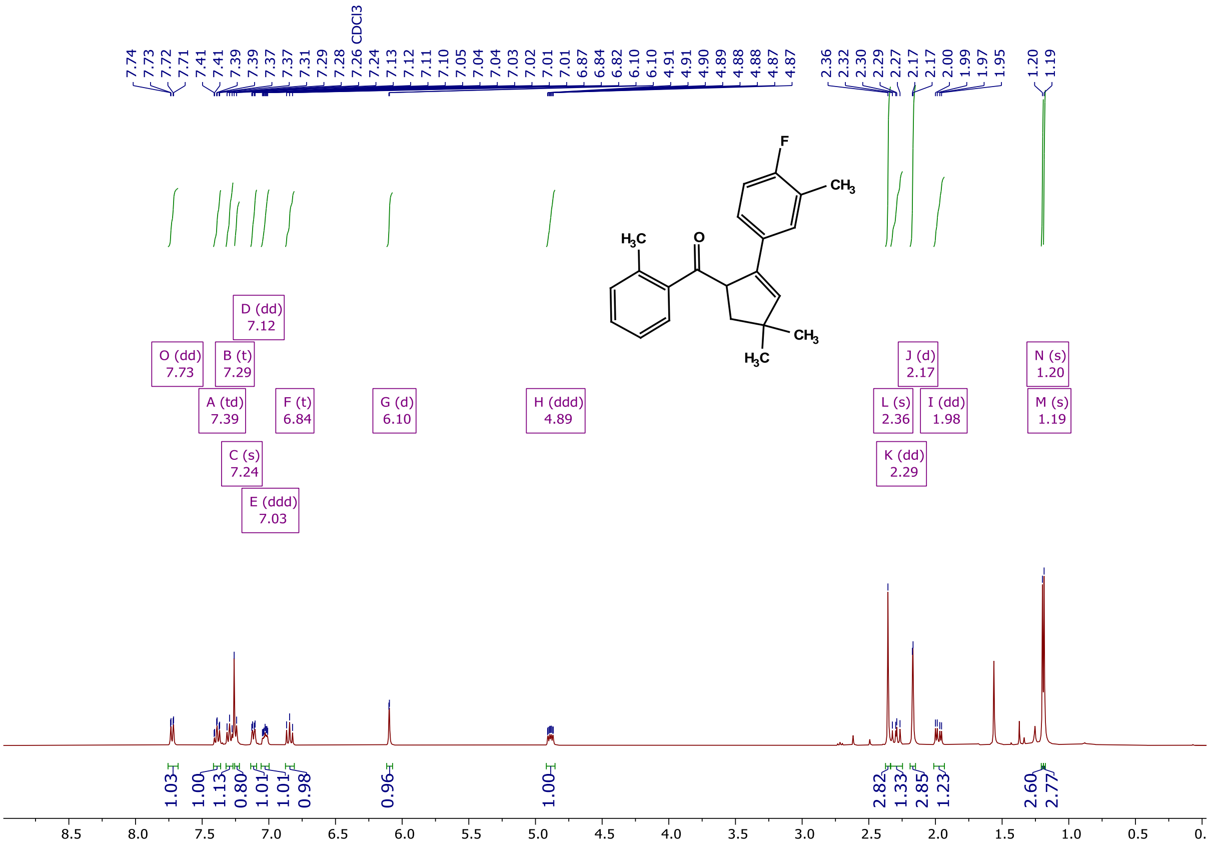

^{13}C NMR (101 MHz, Chloroform-*d*) (**3w**):

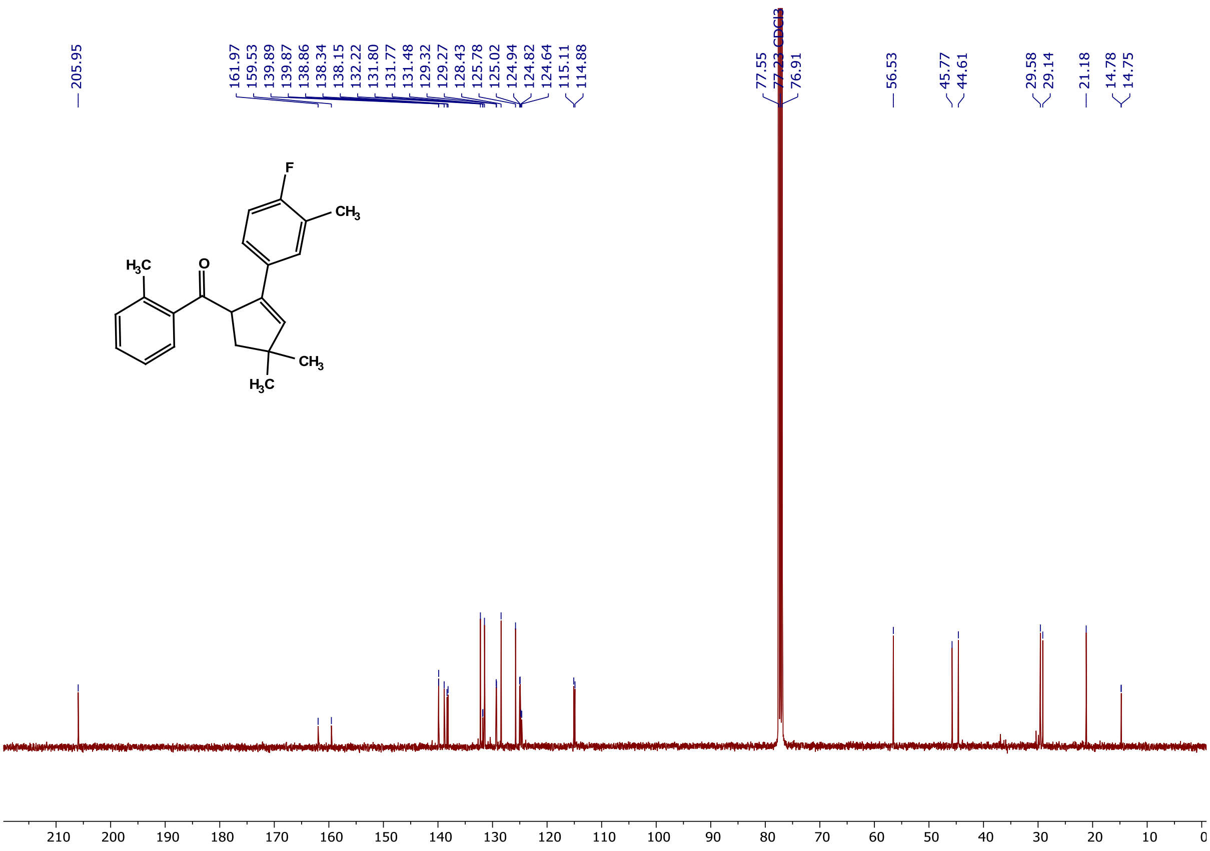

DEPT-135 NMR (101 MHz, Chloroform-*d*) (**3w**):

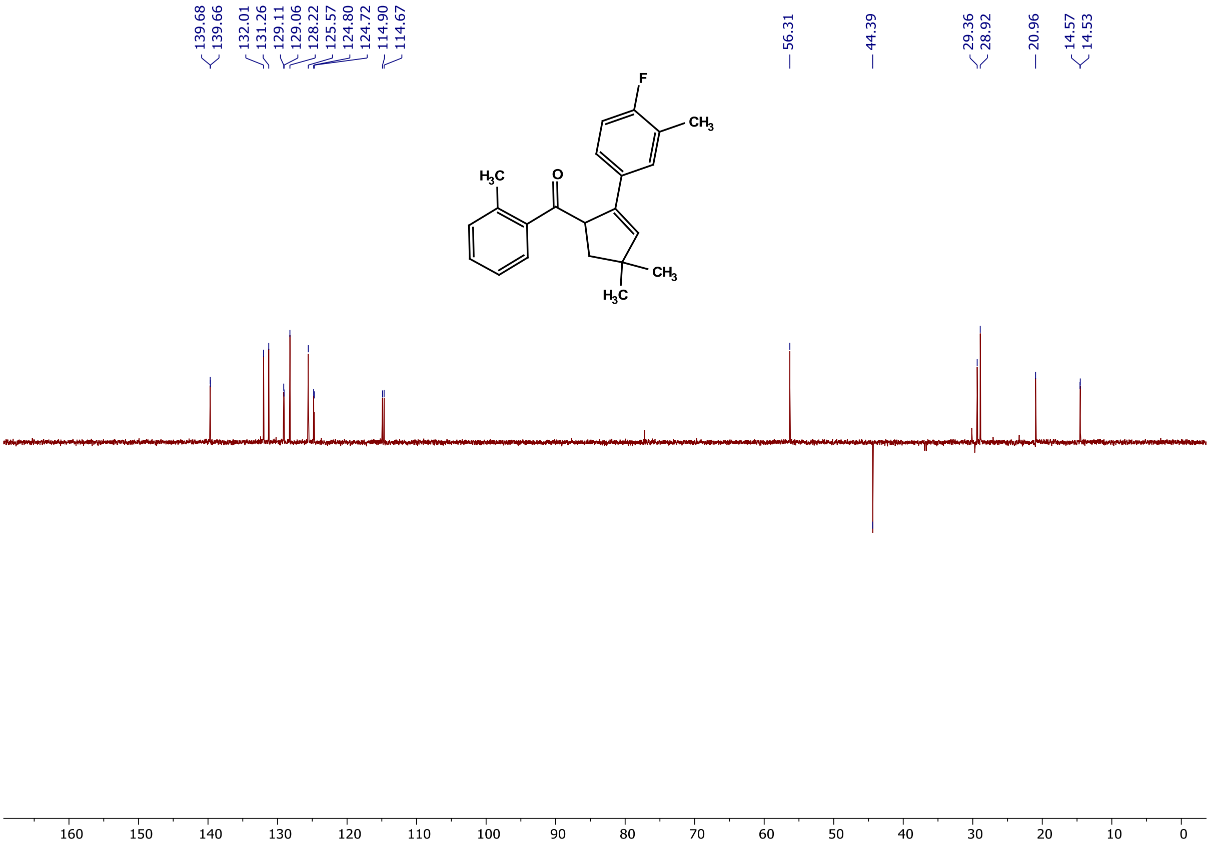

^{19}F NMR (376 MHz, Chloroform-*d*) (**3w**):

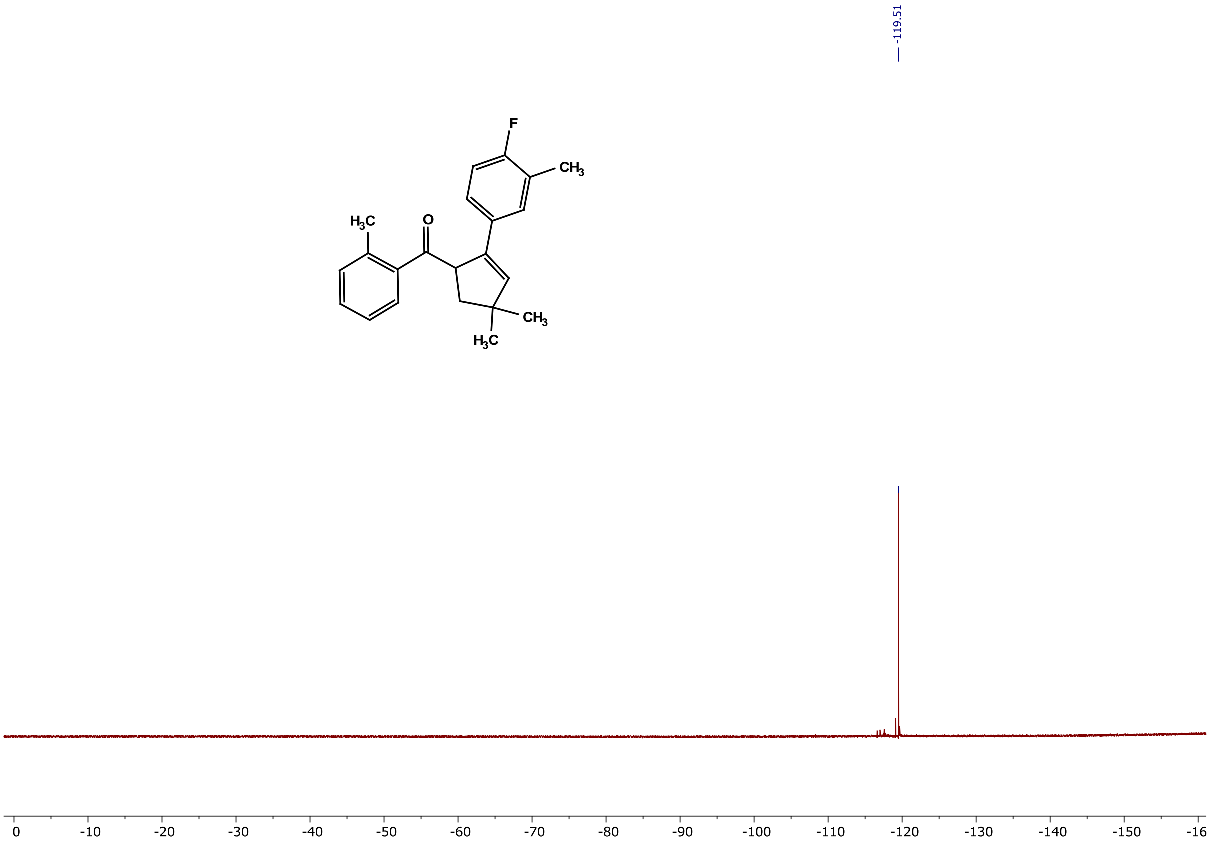

^{1}H NMR (400 MHz, Chloroform-*d*) (**3x**):

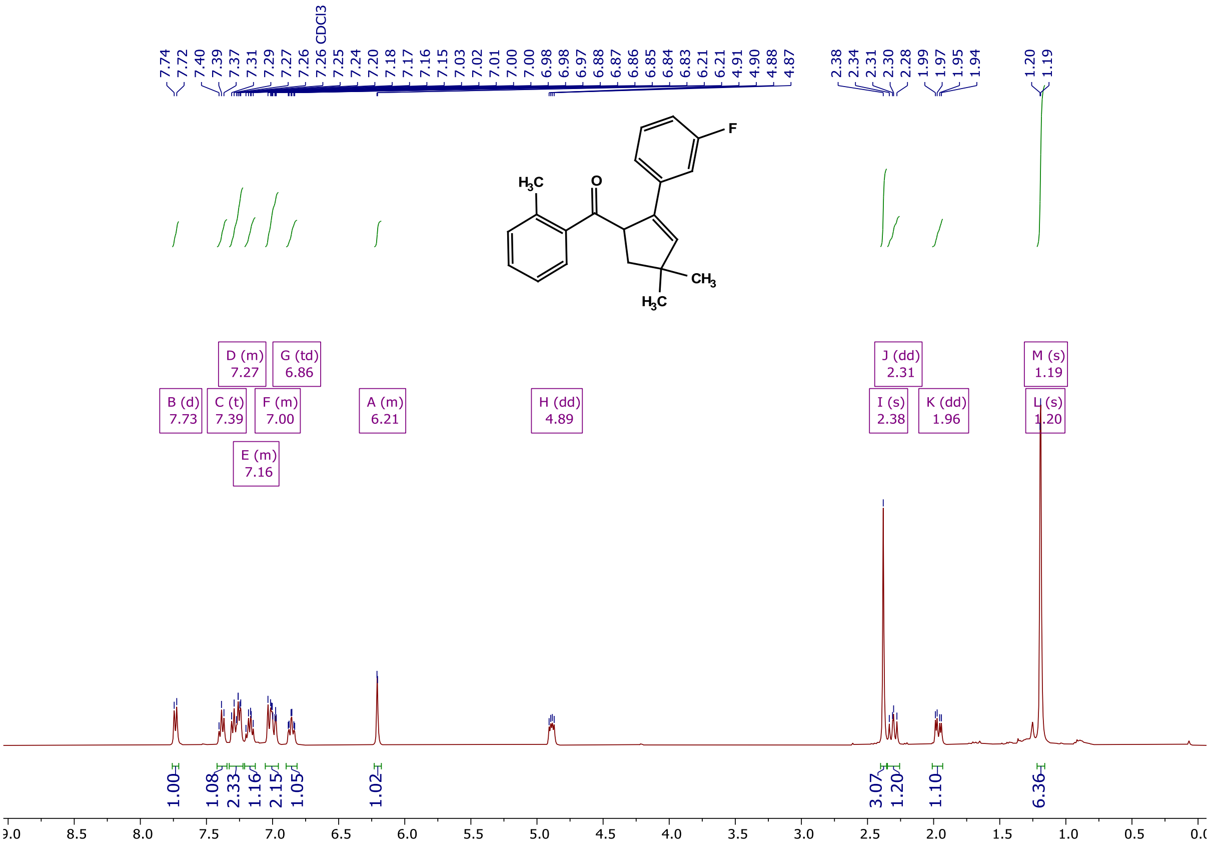

^{13}C NMR (101 MHz, Chloroform-*d*) (**3x**):

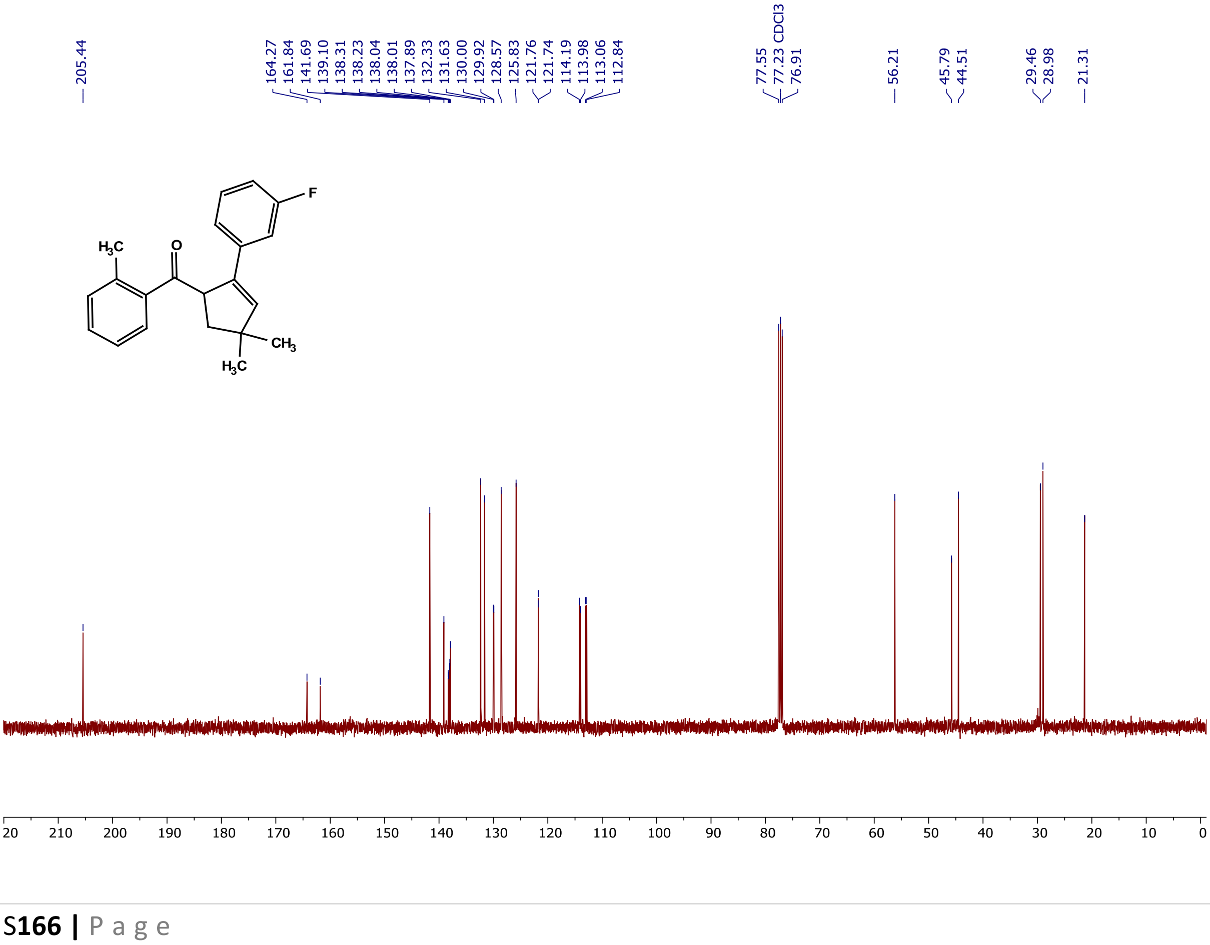

DEPT-135 NMR (101 MHz, Chloroform-*d*) (**3x**):

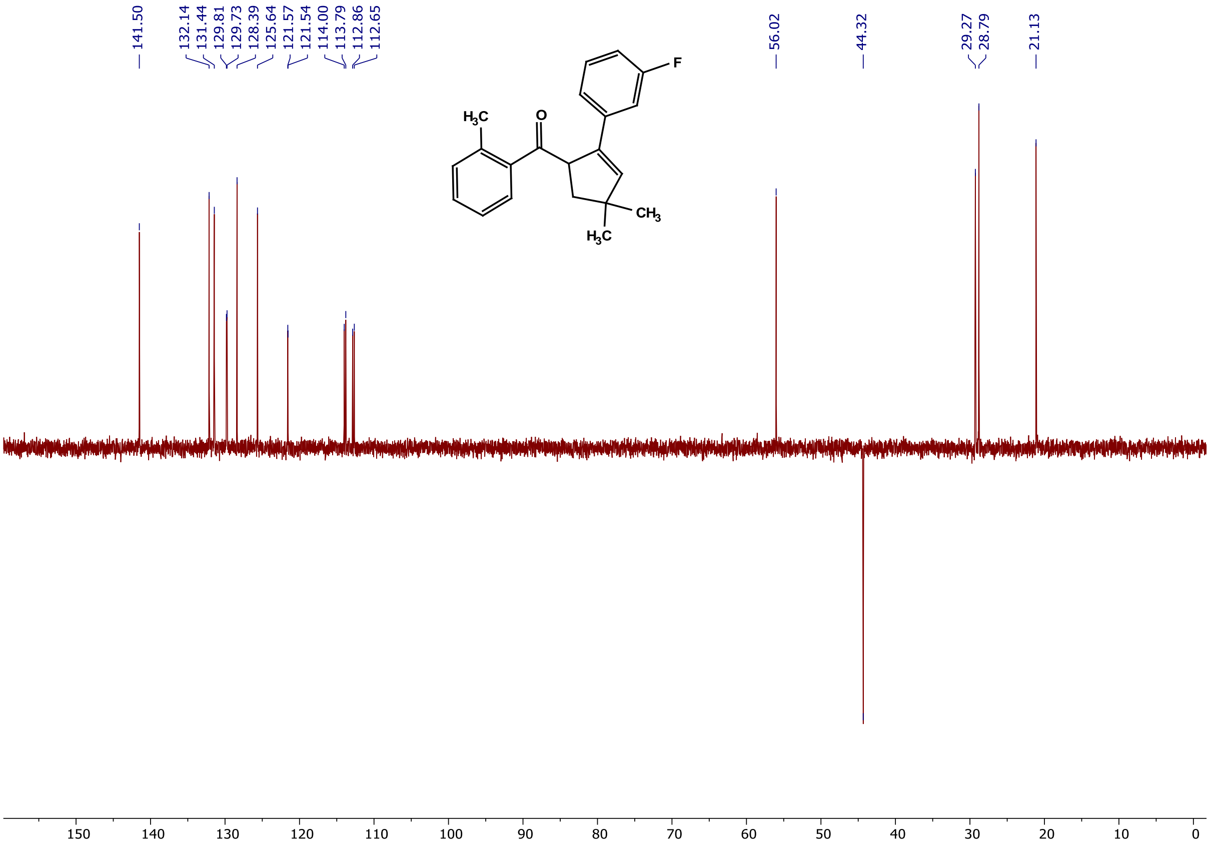

^{19}F NMR (376 MHz, Chloroform-*d*) (**3x**):

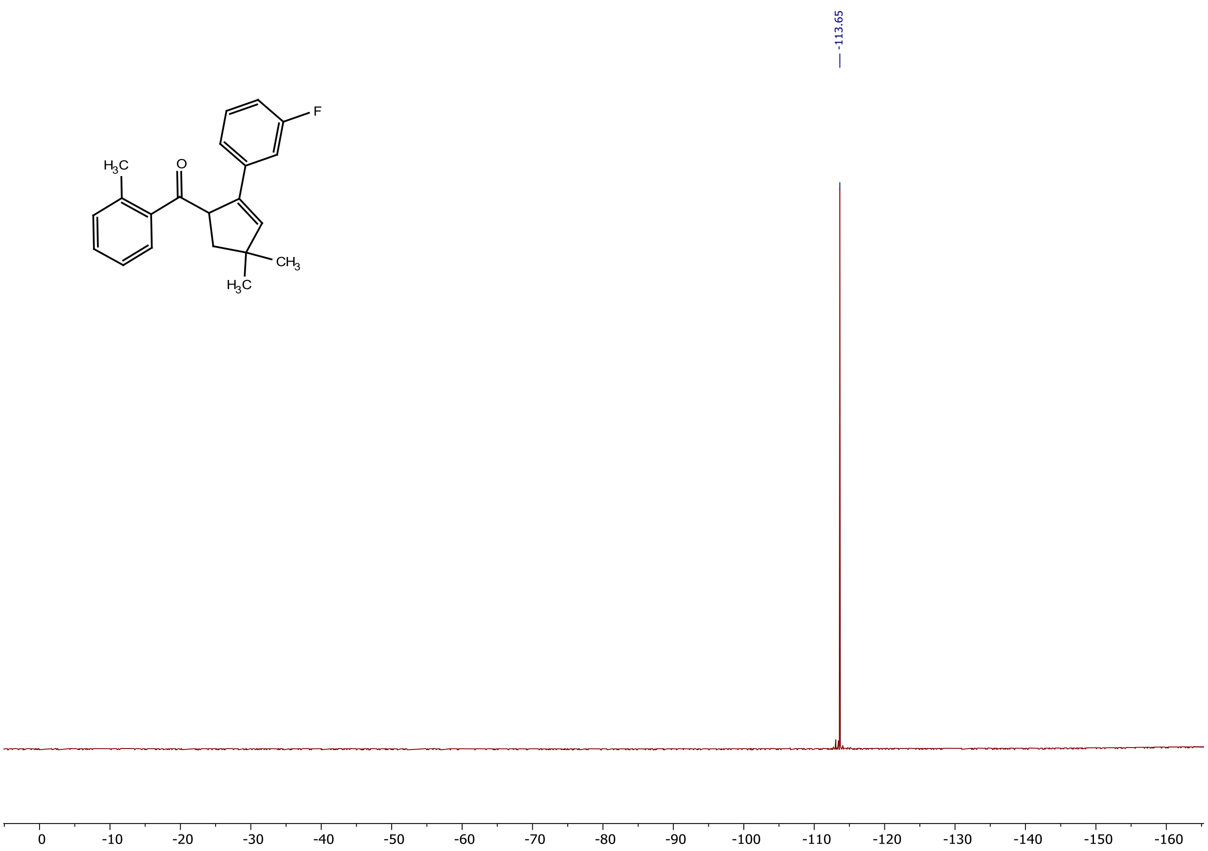

^{1}H NMR (400 MHz, Chloroform-*d*) (**3y**):

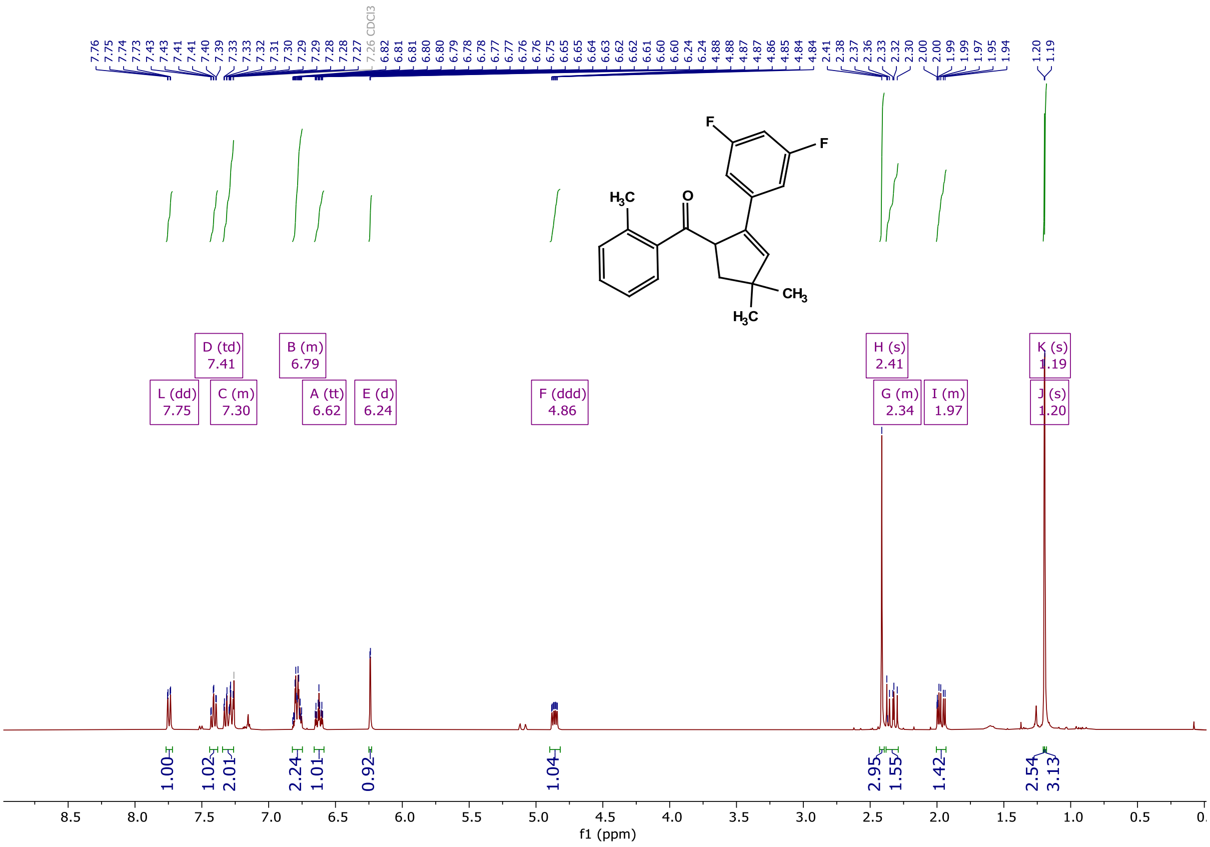

^{13}C NMR (101 MHz, Chloroform-*d*) (**3y**):

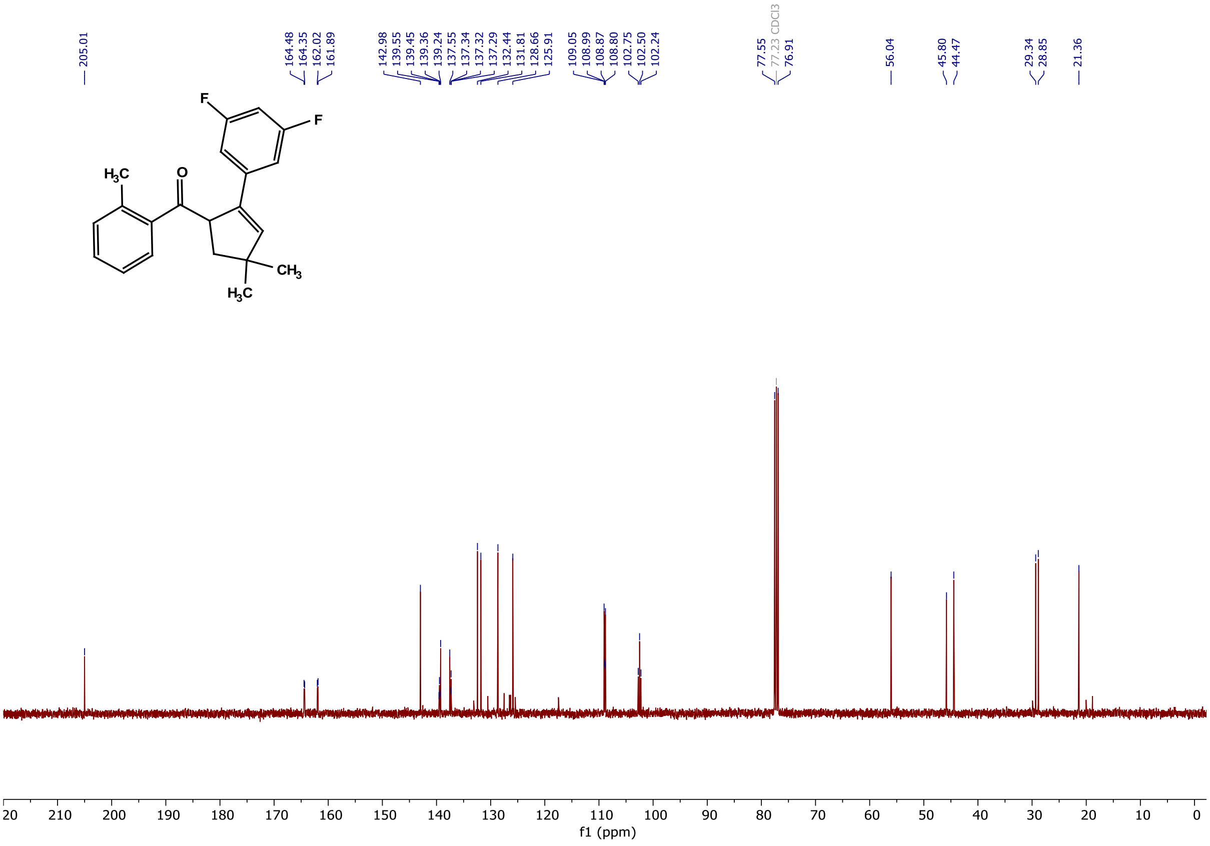

DEPT-135 NMR (101 MHz, Chloroform-*d*) (**3y**):

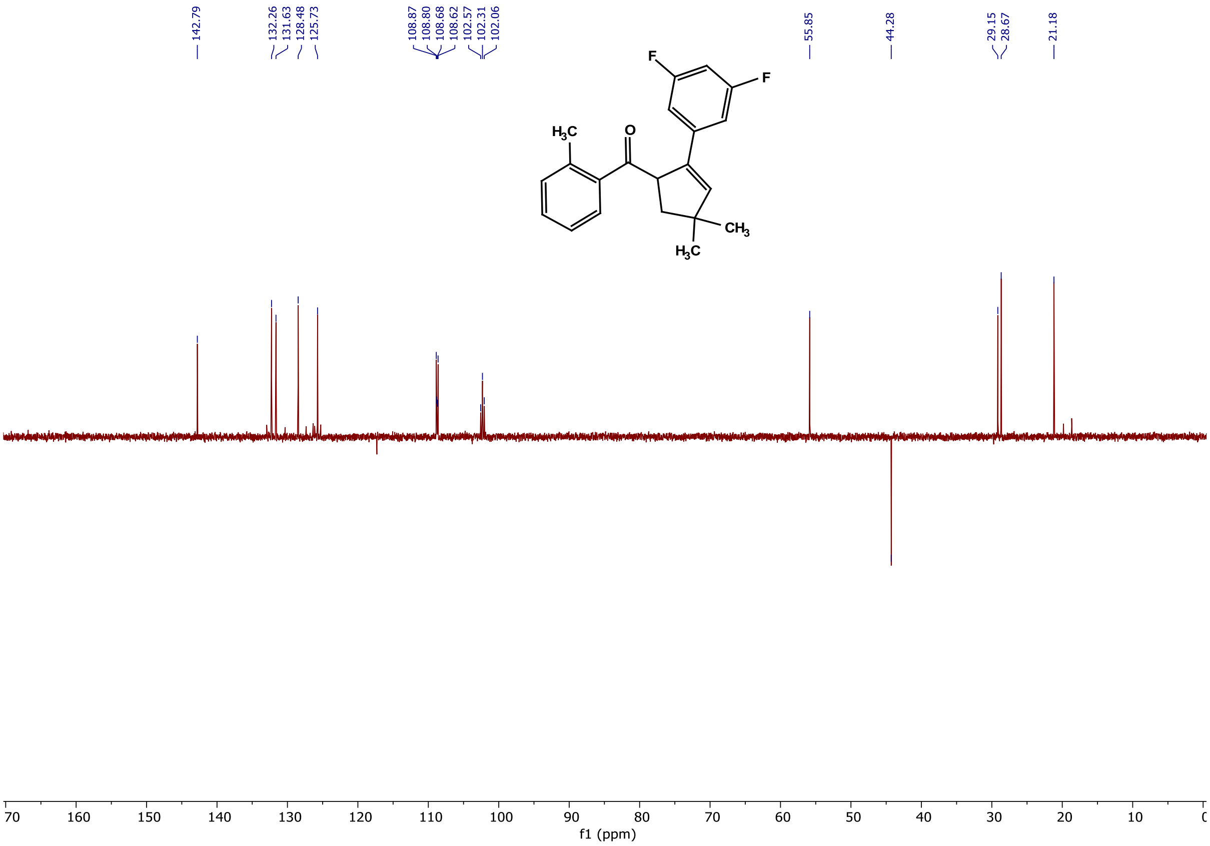

^{19}F NMR (376 MHz, Chloroform-*d*) (**3y**):

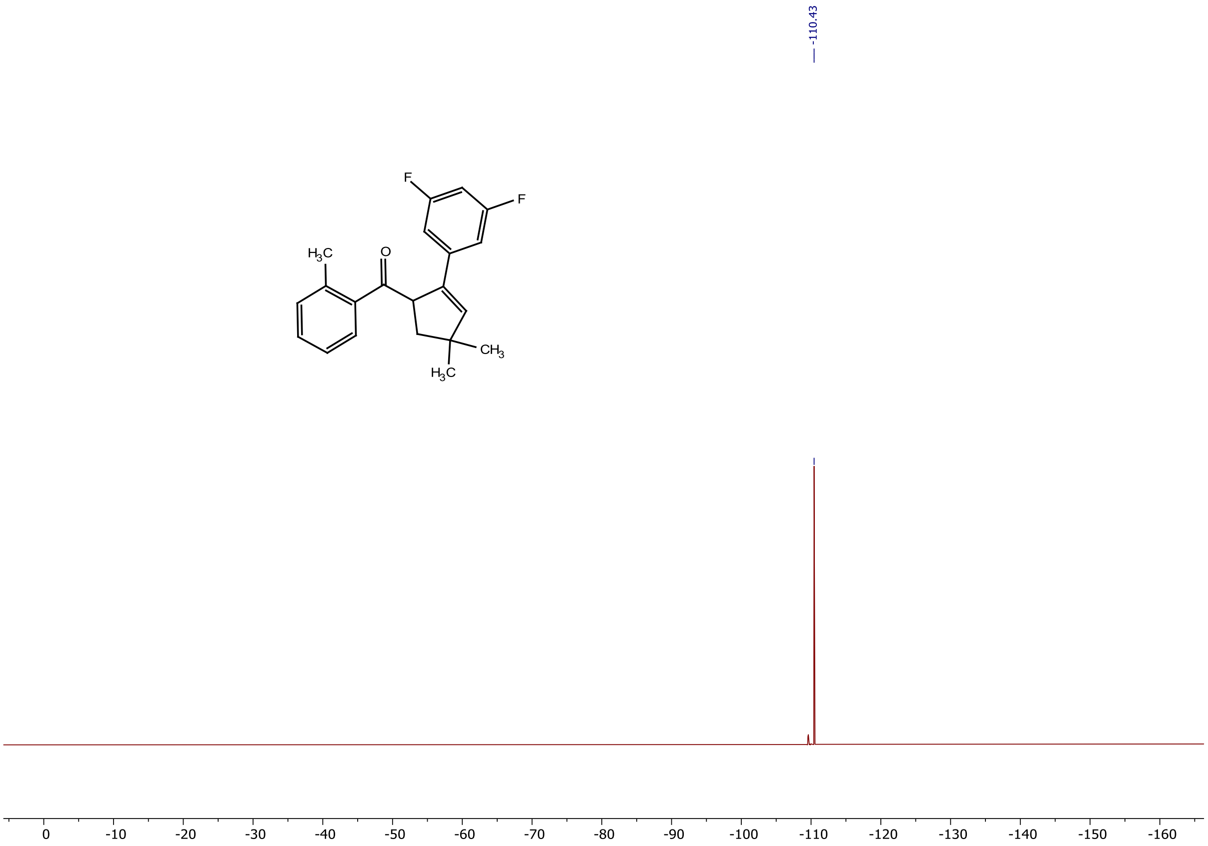

^{1}H NMR (400 MHz, Chloroform-*d*) (**3z**):

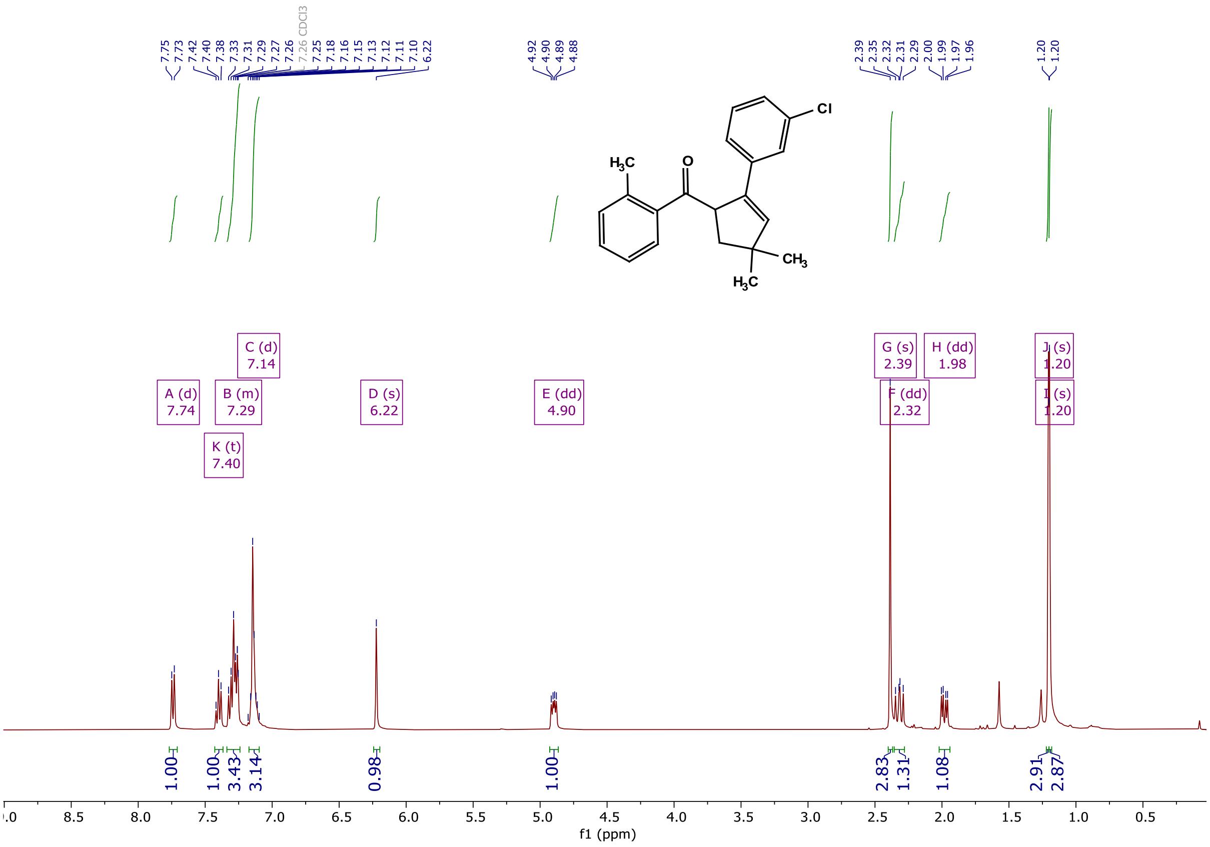

^{13}C NMR (101 MHz, Chloroform-*d*) (**3z**):

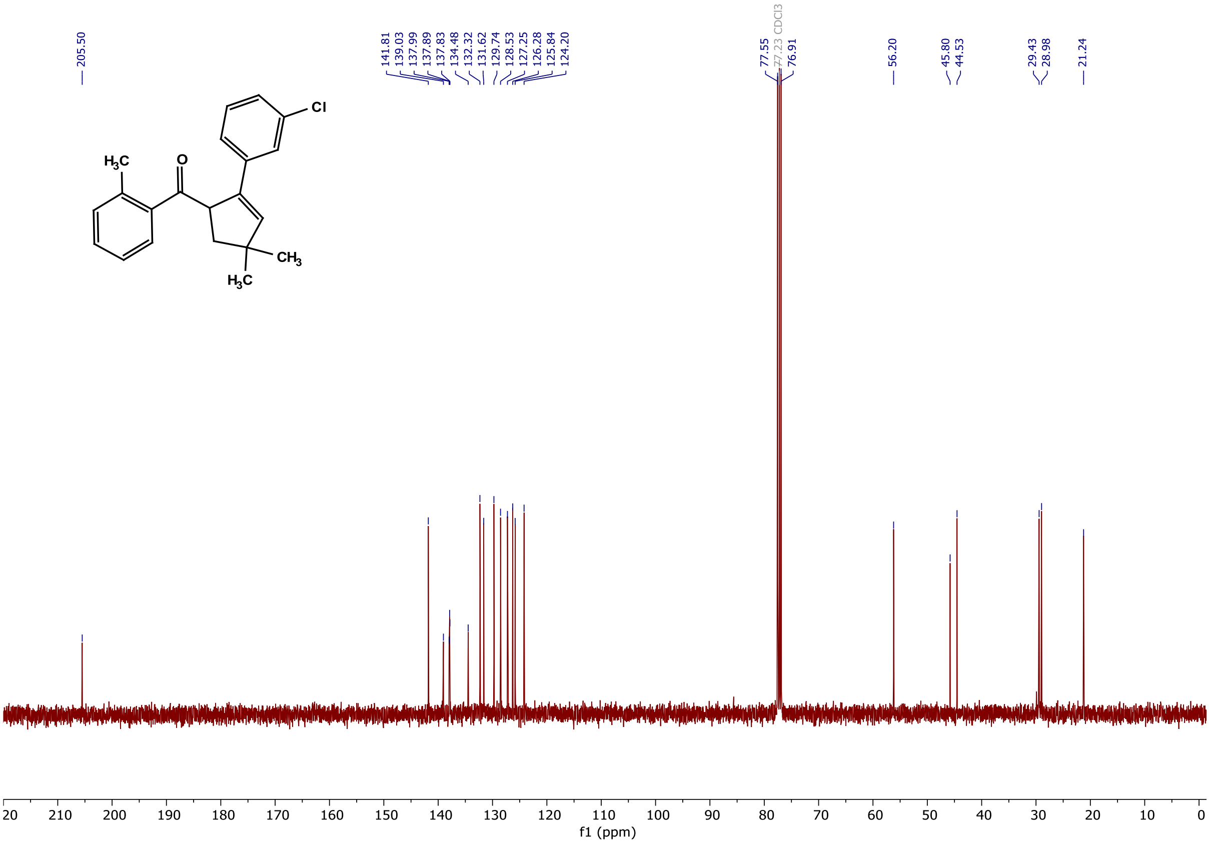

DEPT-135 NMR (101 MHz, Chloroform-*d*) (**3z**):

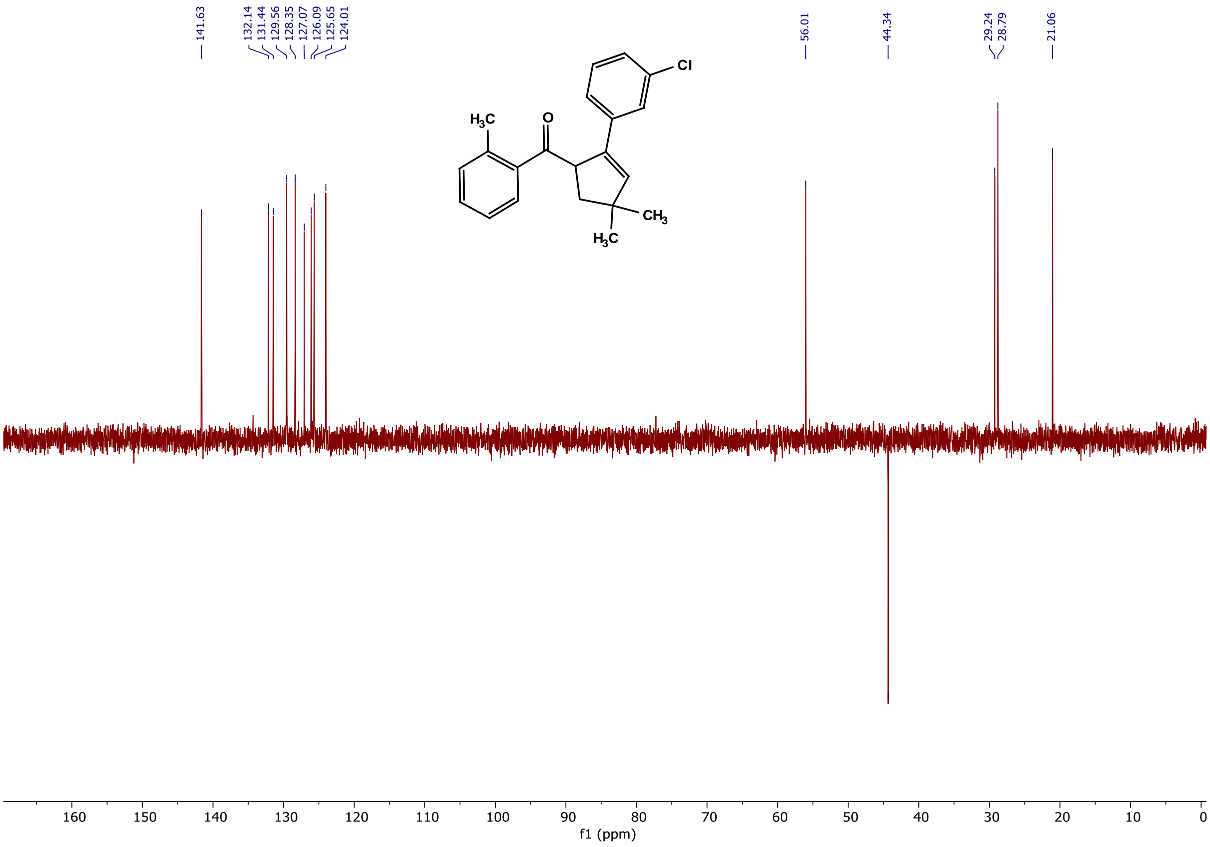

^{1}H NMR (400 MHz, Chloroform-*d*) (**3aa**):

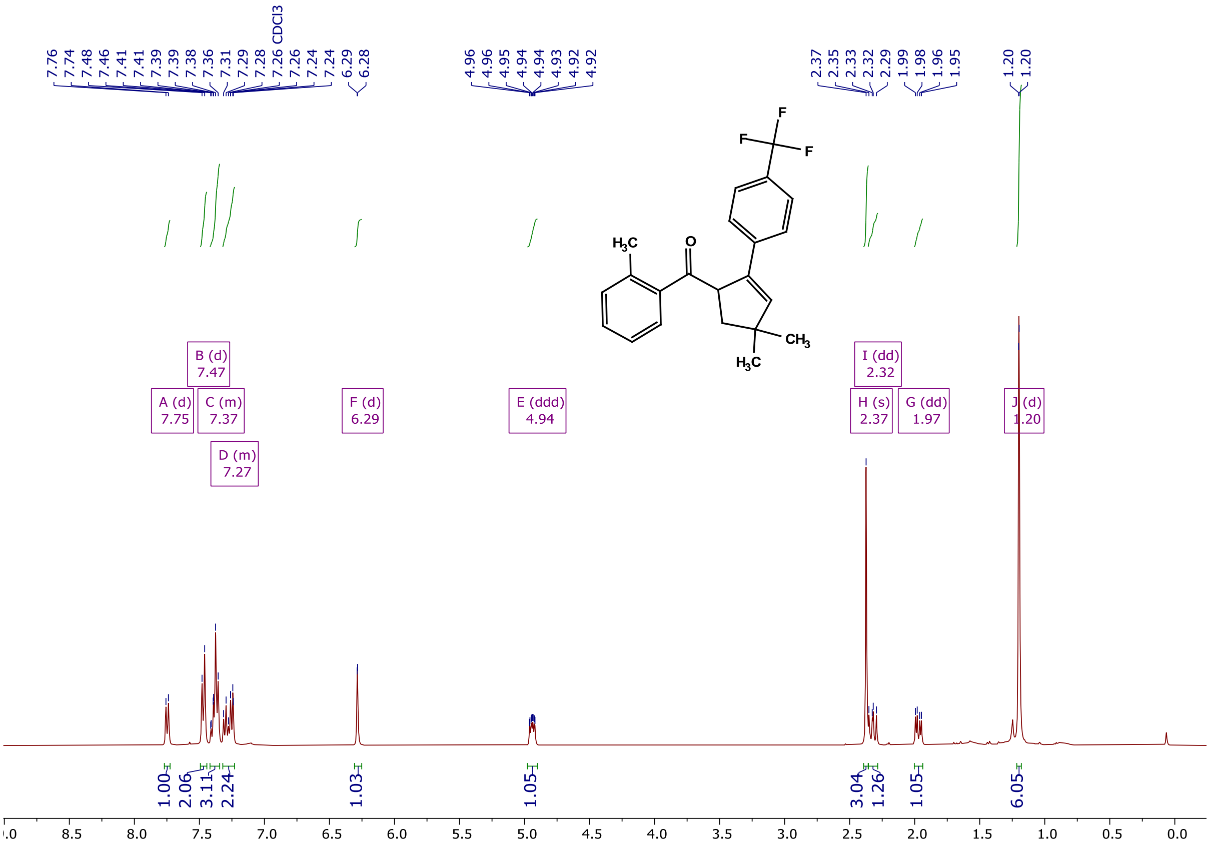

^{13}C NMR (101 MHz, Chloroform-*d*) (**3aa**):

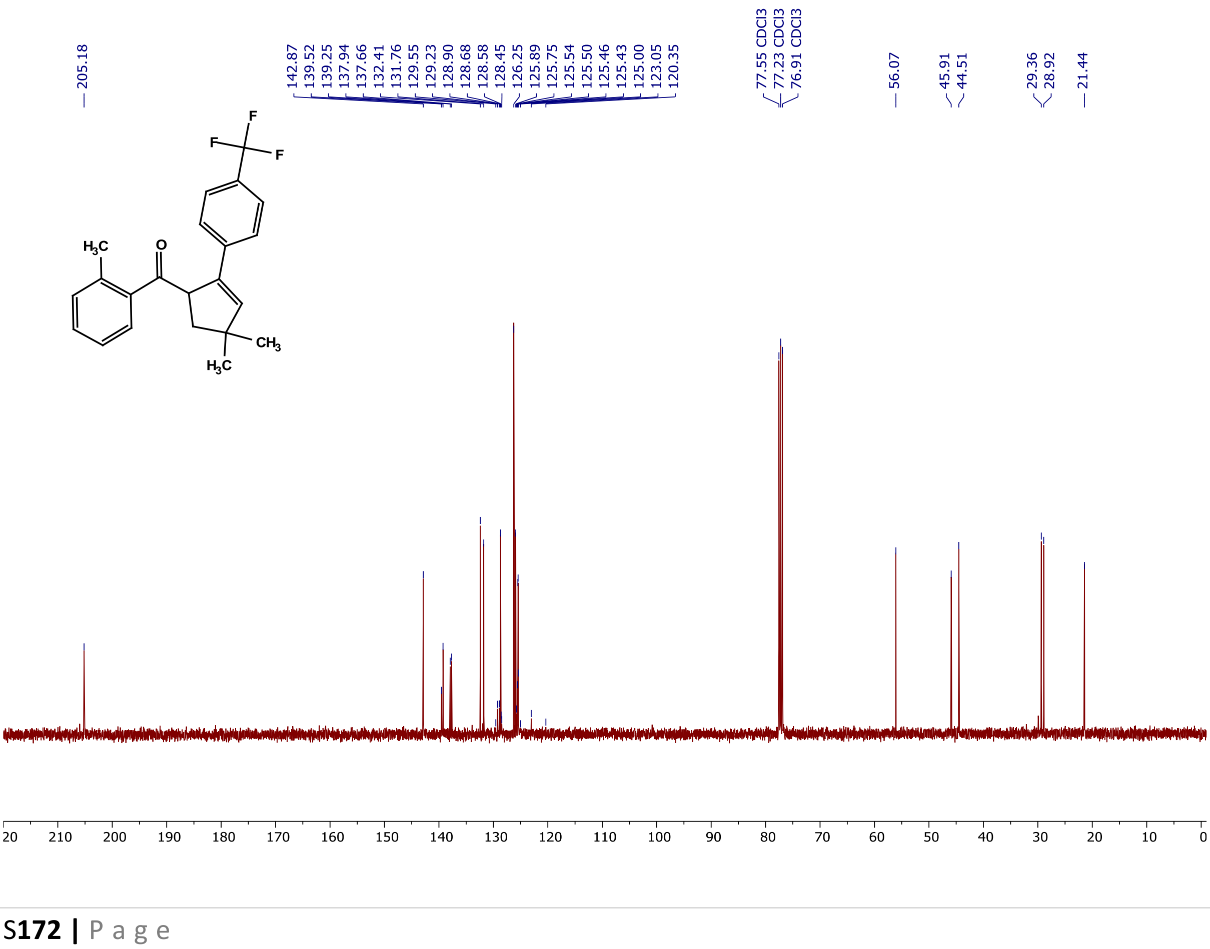

DEPT-135 NMR (101 MHz, Chloroform-*d*) (**3aa**):

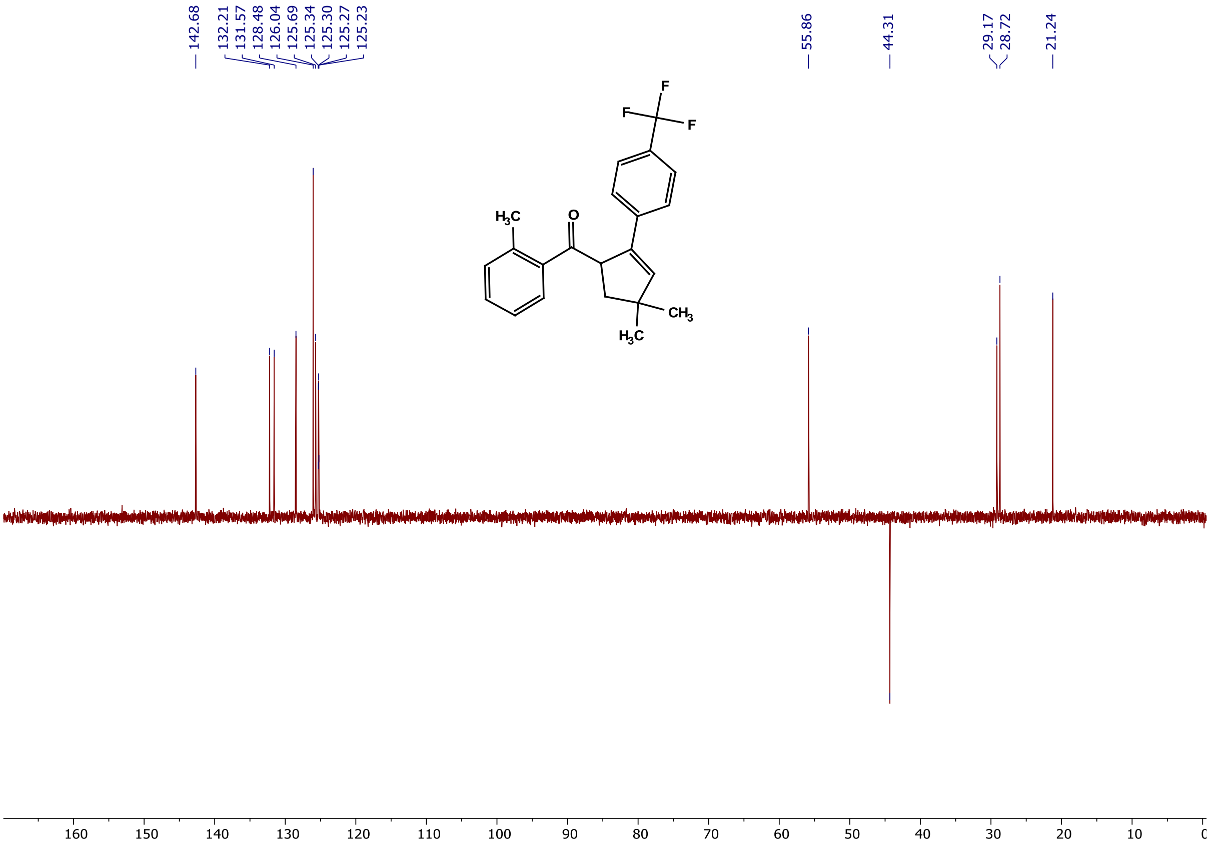

^{19}F NMR (376 MHz, Chloroform-*d*) (**3aa**):

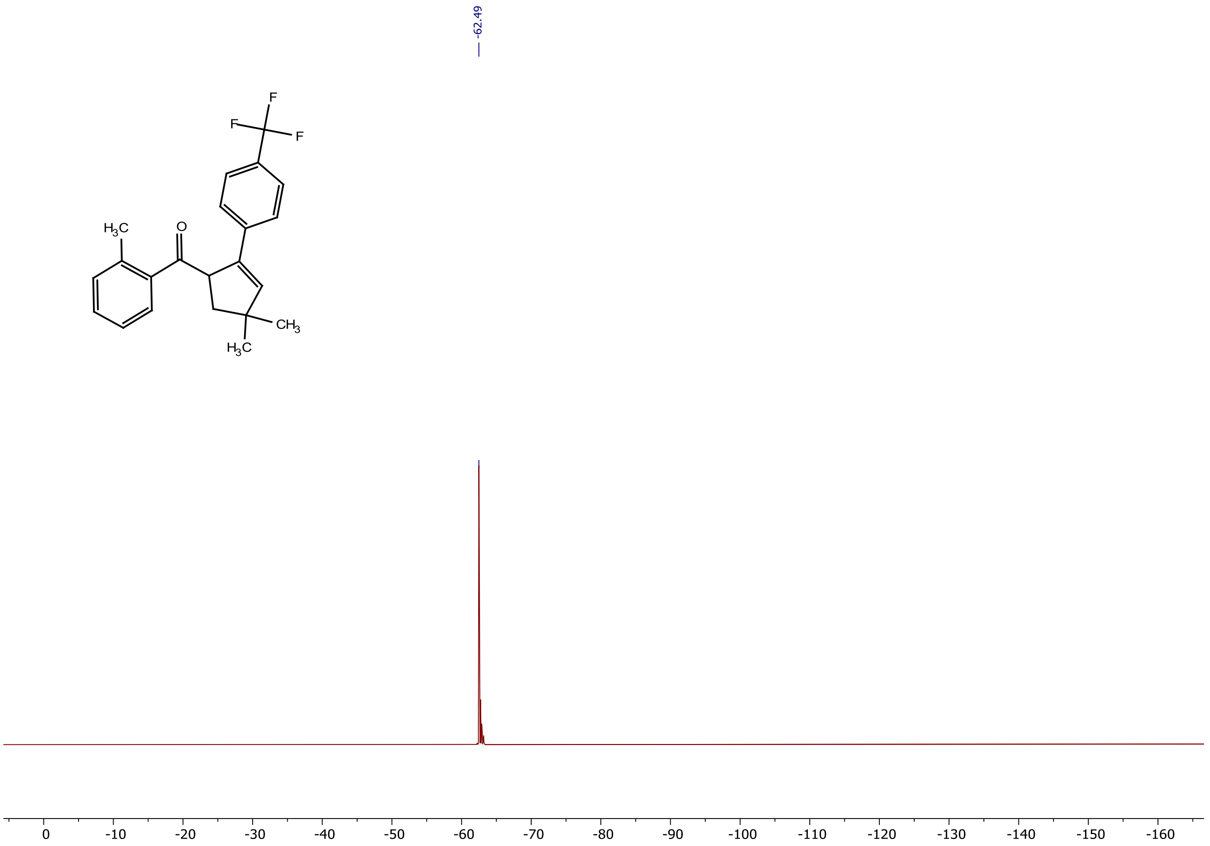

^{1}H NMR (400 MHz, Chloroform-*d*) (**3ab**):

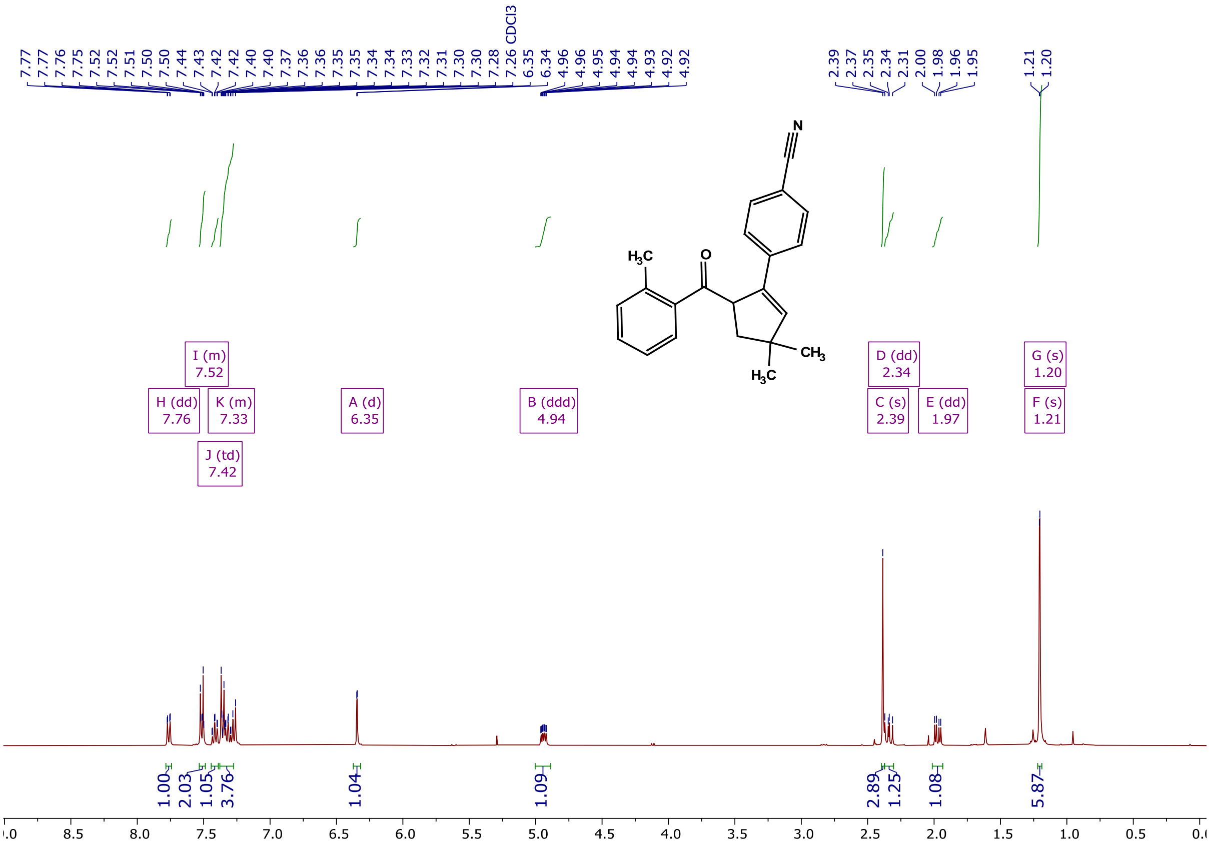

^{13}C NMR (101 MHz, Chloroform-*d*) (**3ab**):

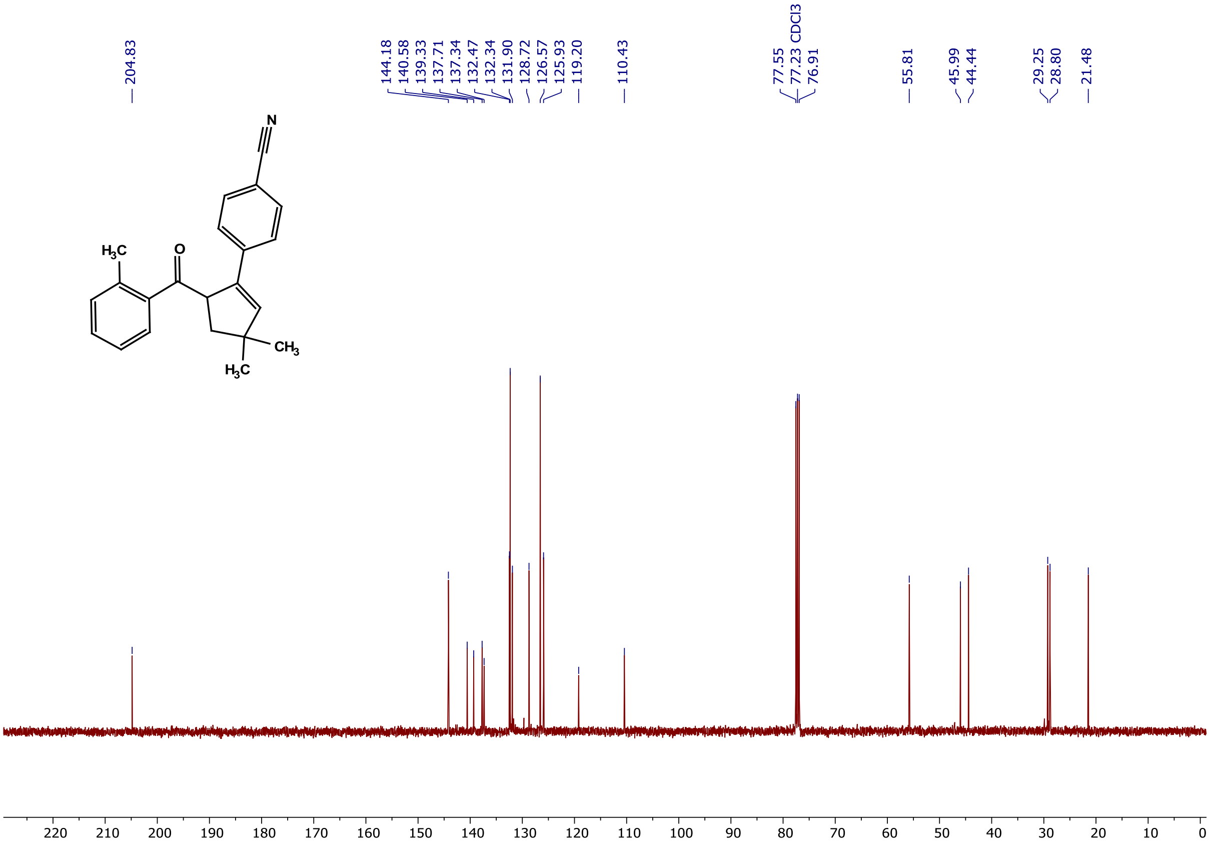

^{1}H NMR (500 MHz, Chloroform-*d*) (**3ac**):

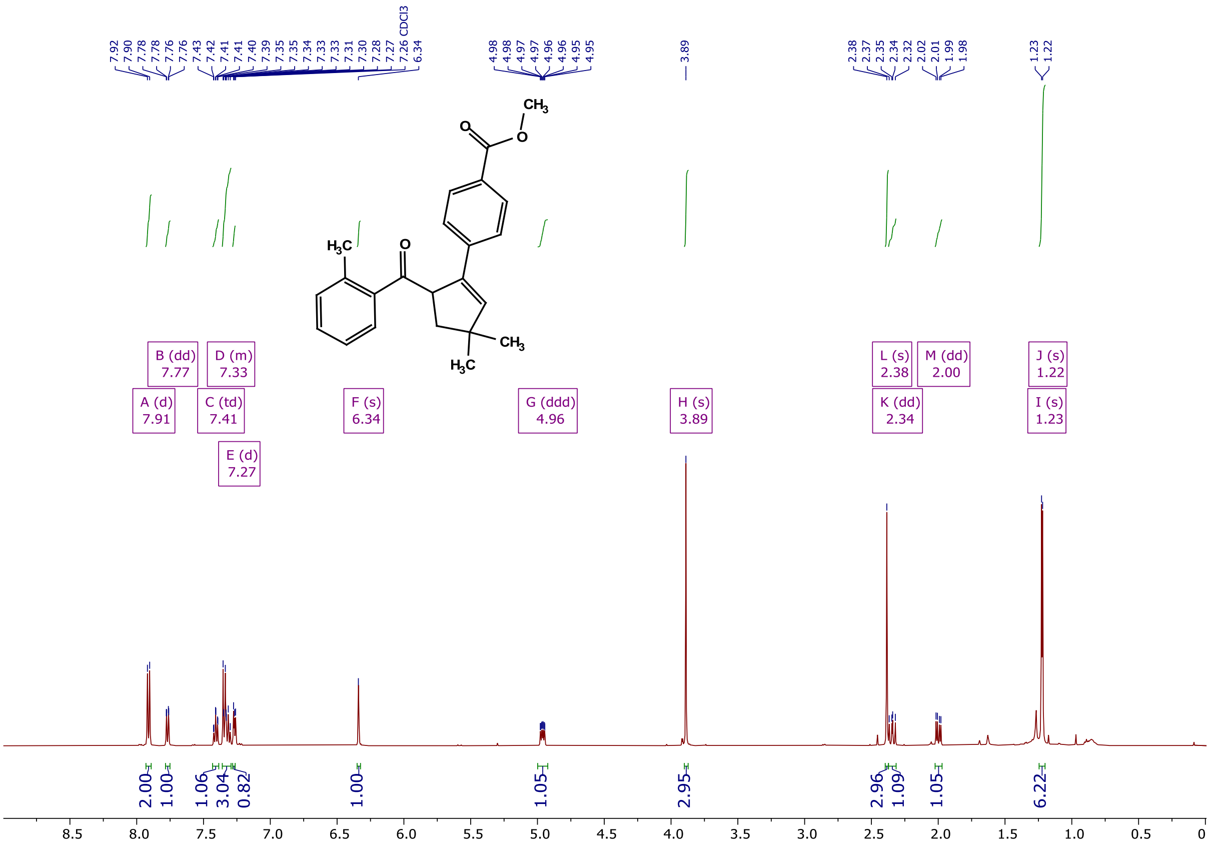

^{13}C NMR (126 MHz, Chloroform-*d*) (**3ac**):

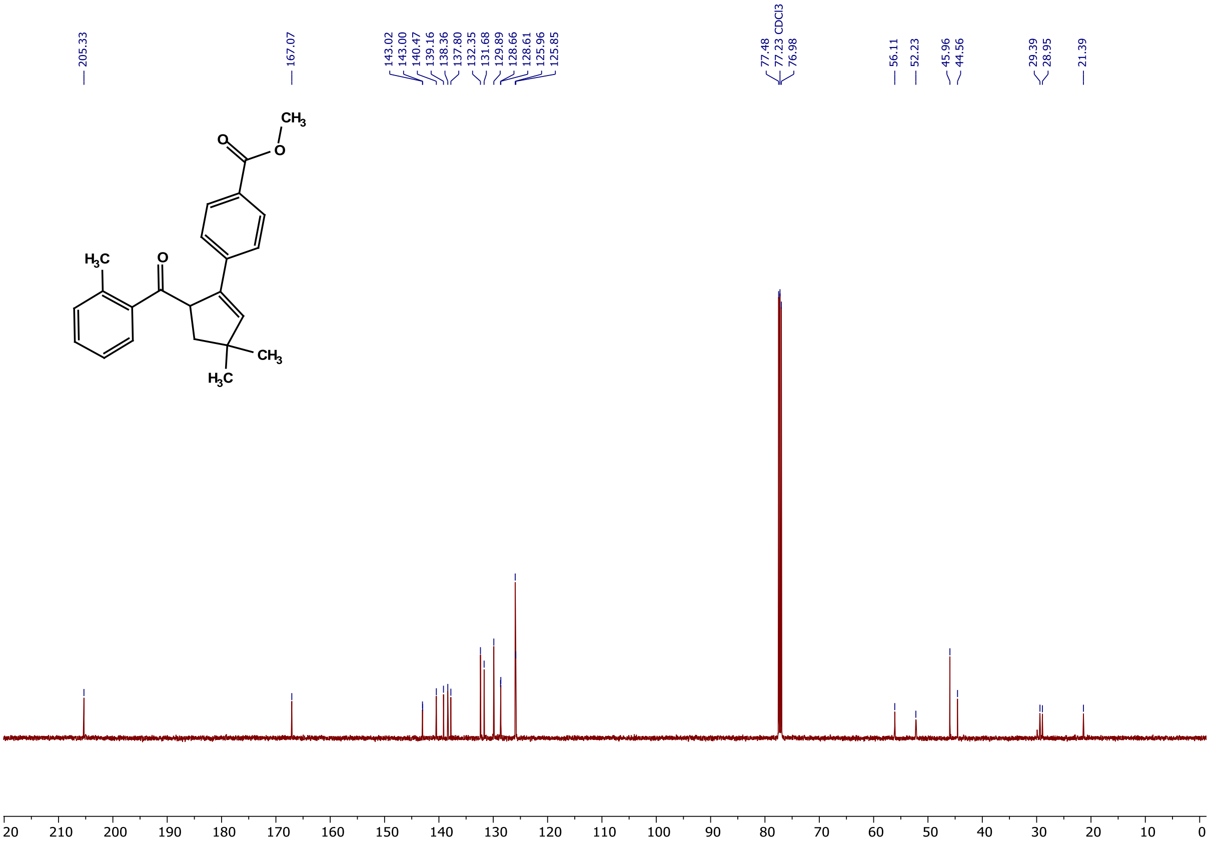

^{1}H NMR (500 MHz, Chloroform-*d*) (**3ad**):

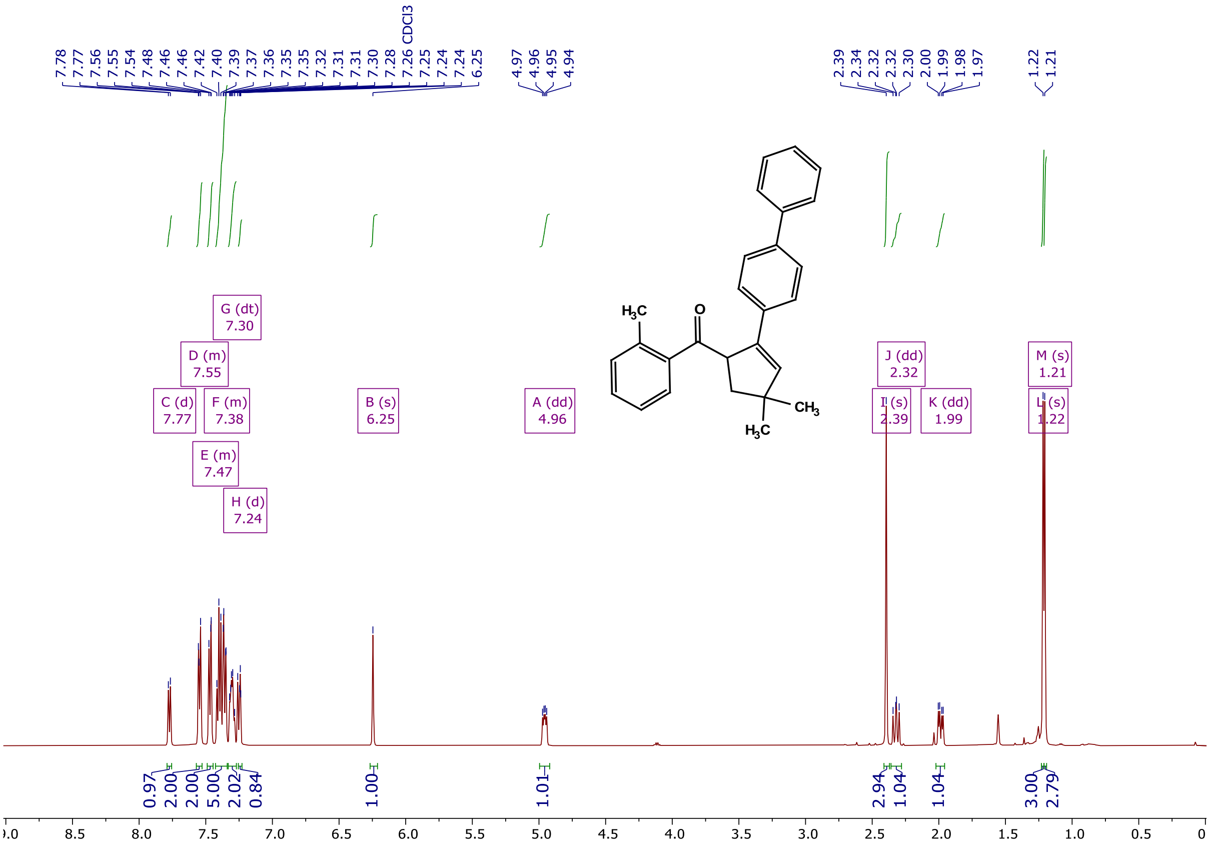

^{13}C NMR (101 MHz, Chloroform-*d*) (**3ad**):

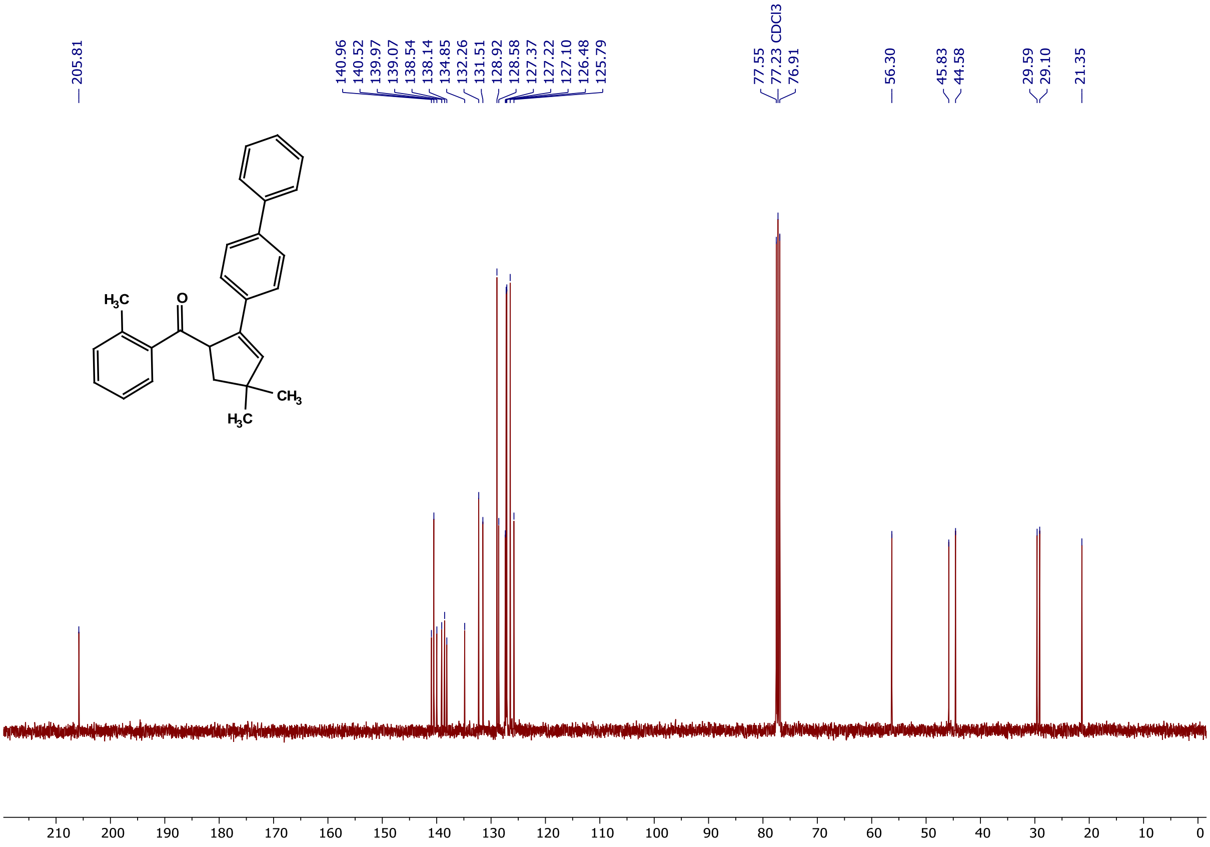

DEPT-135 NMR (101 MHz, Chloroform-*d*) (**3ad**):

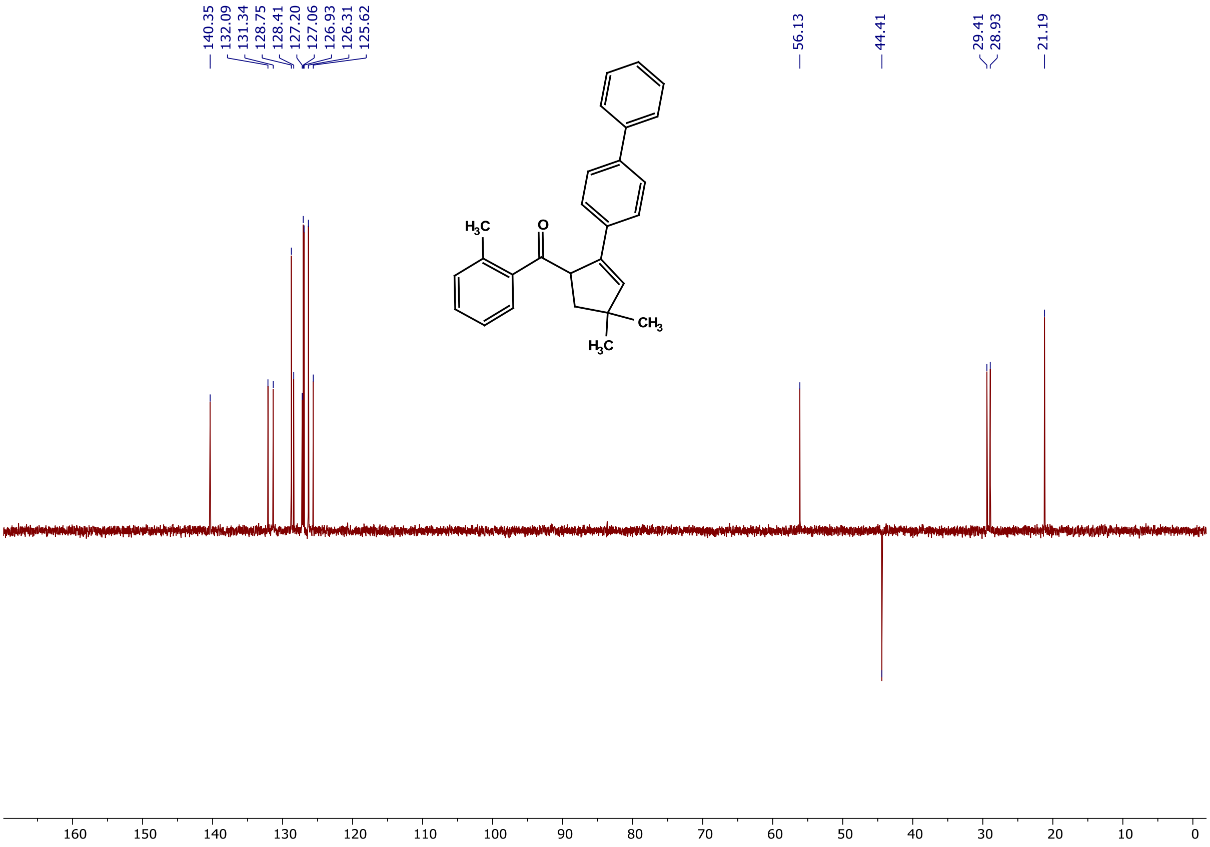

^{1}H NMR (400 MHz, Chloroform-*d*) (**3ae**):

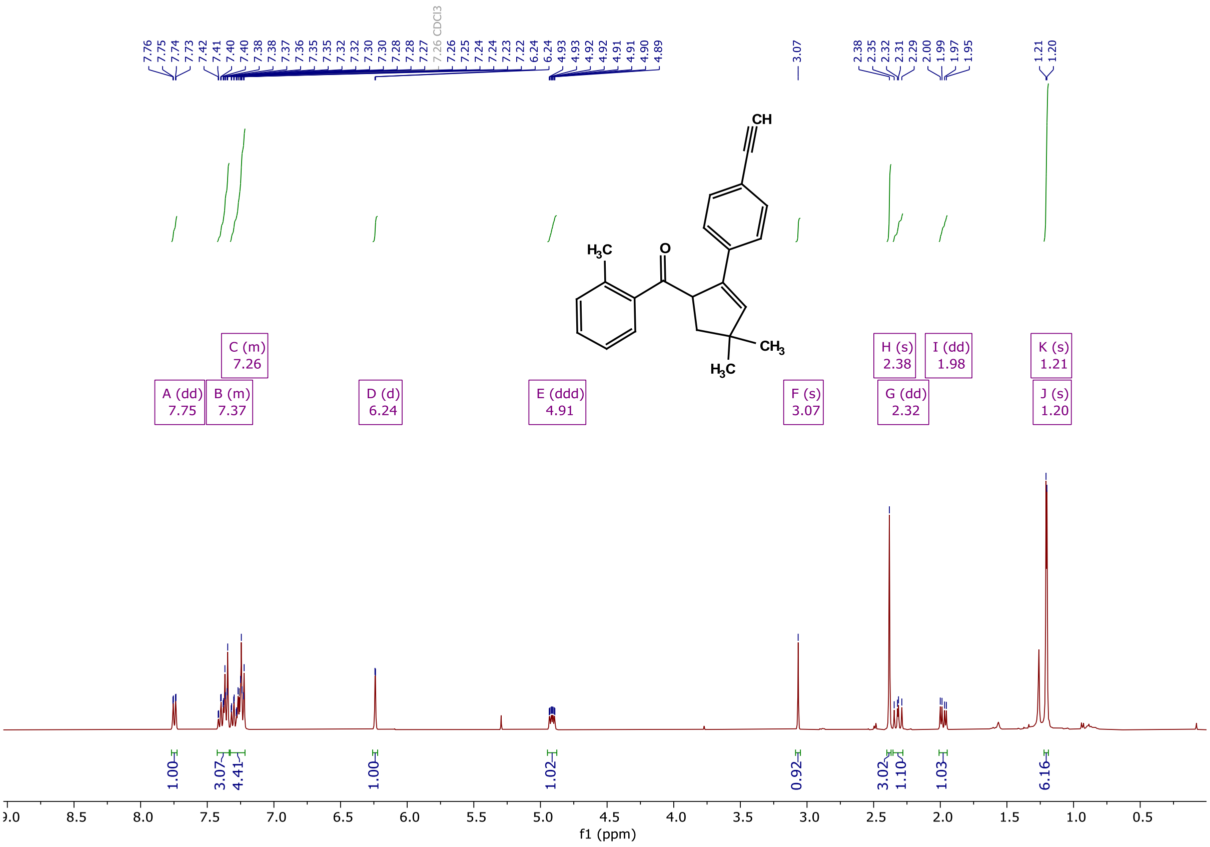

^{13}C NMR (101 MHz, Chloroform-*d*) (**3ae**):

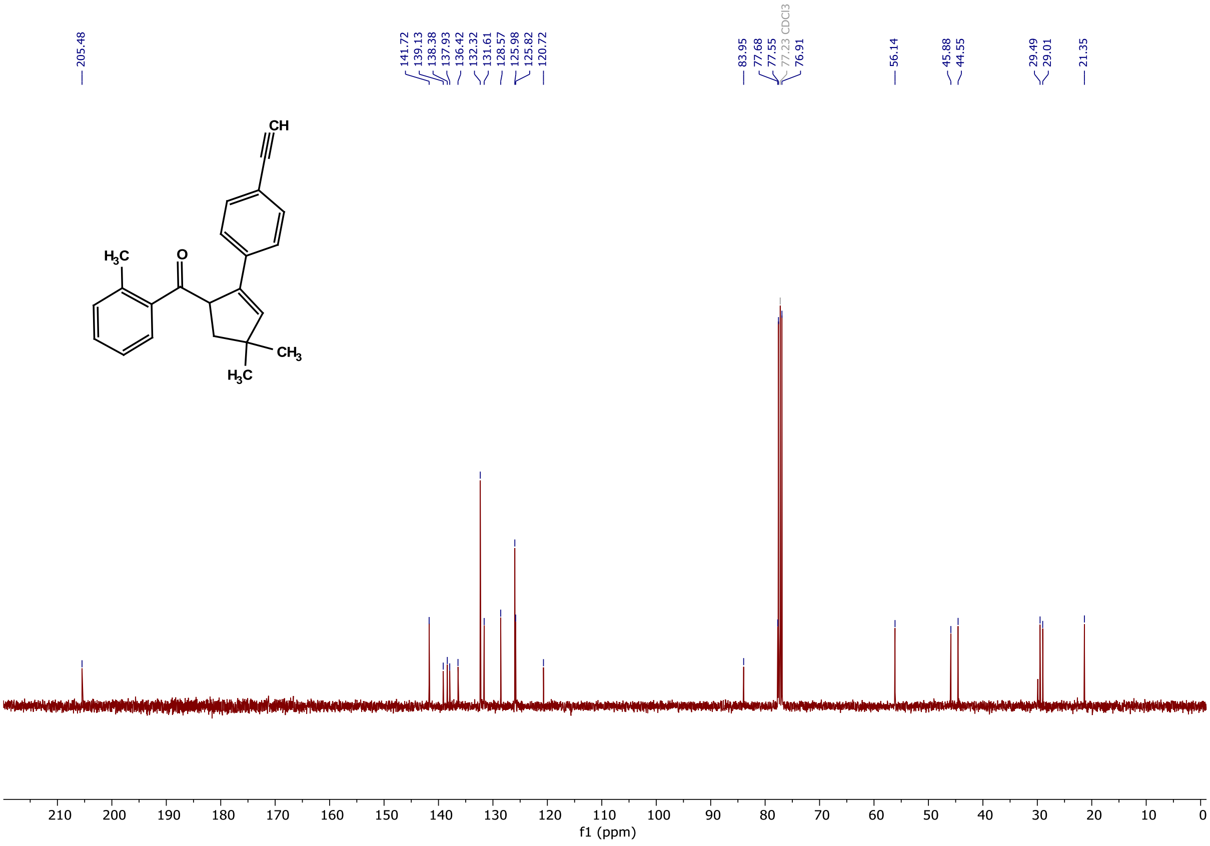

DEPT-135 NMR (101 MHz, Chloroform-*d*) (**3ae**):

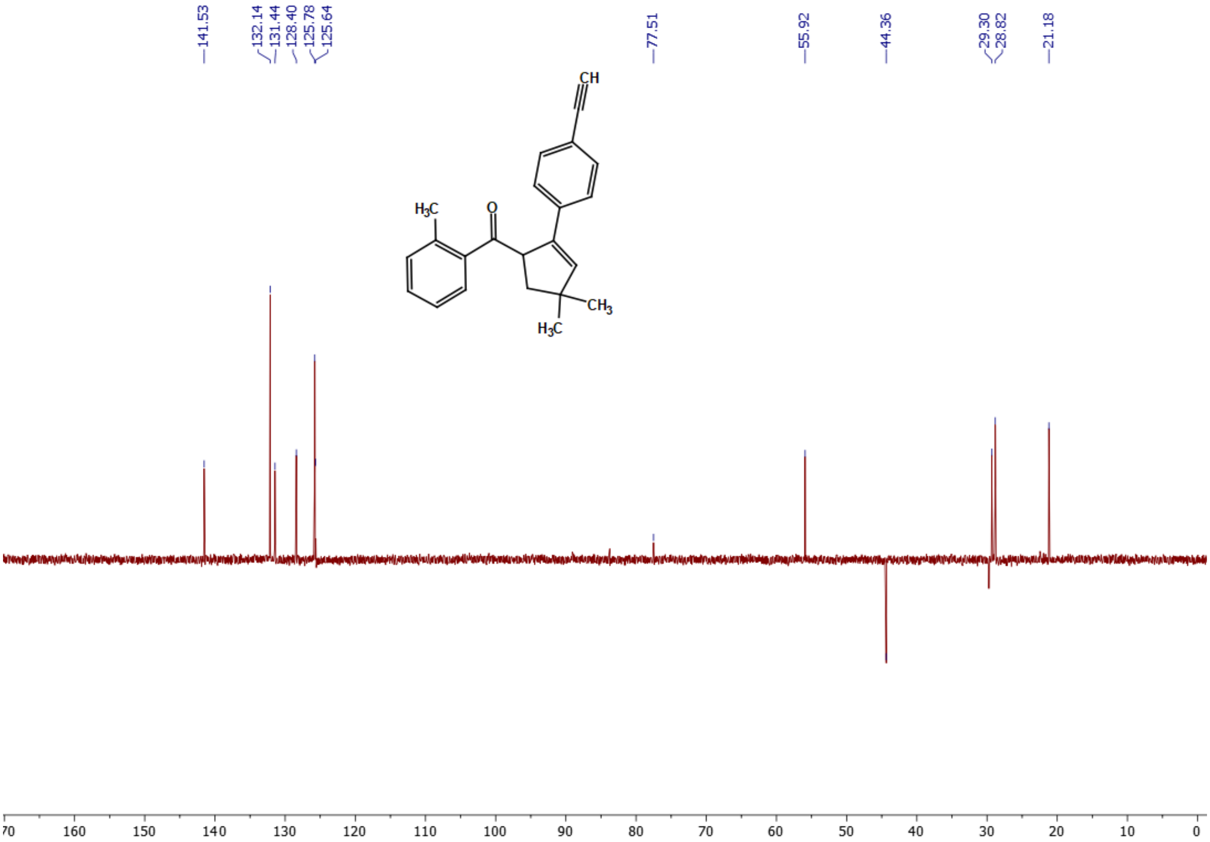

^{1}H NMR (400 MHz, Chloroform-*d*) (**3af**):

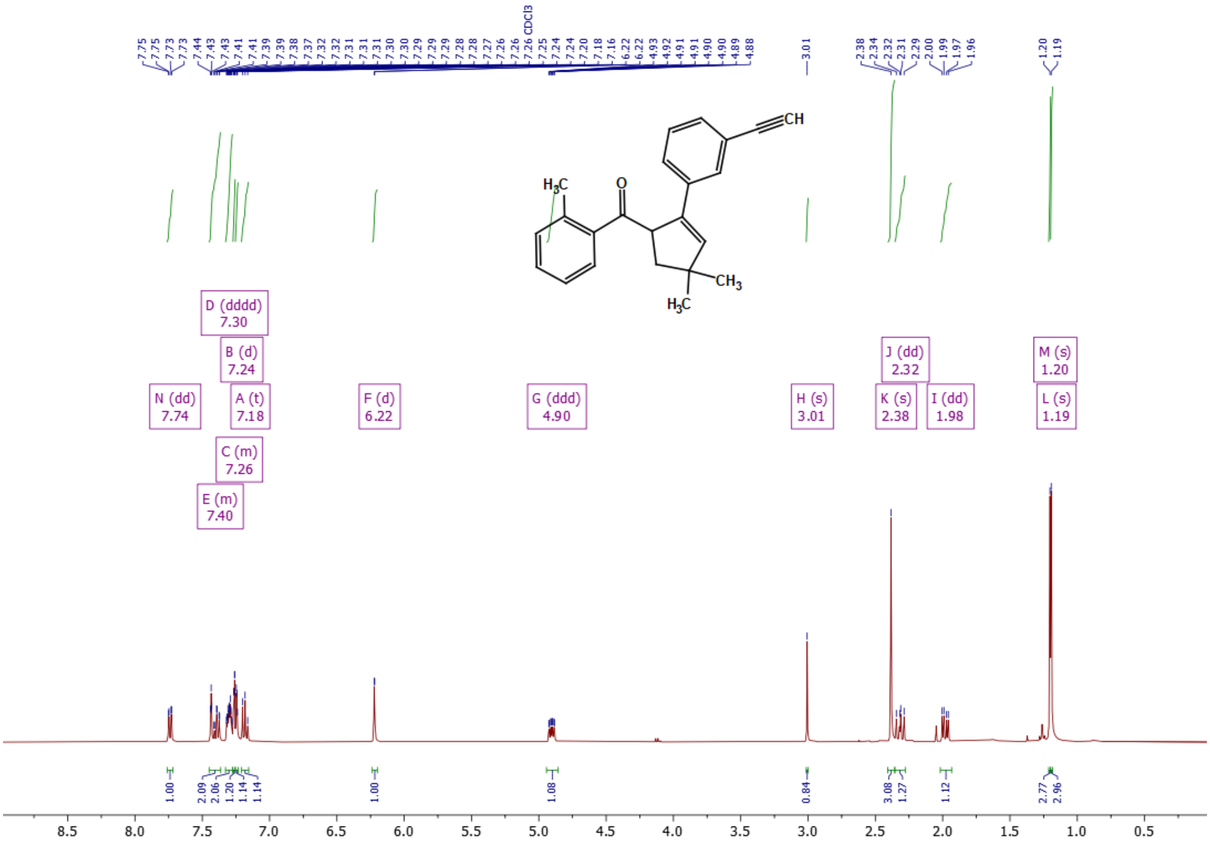

^{13}C NMR (101 MHz, Chloroform-*d*) (**3af**):

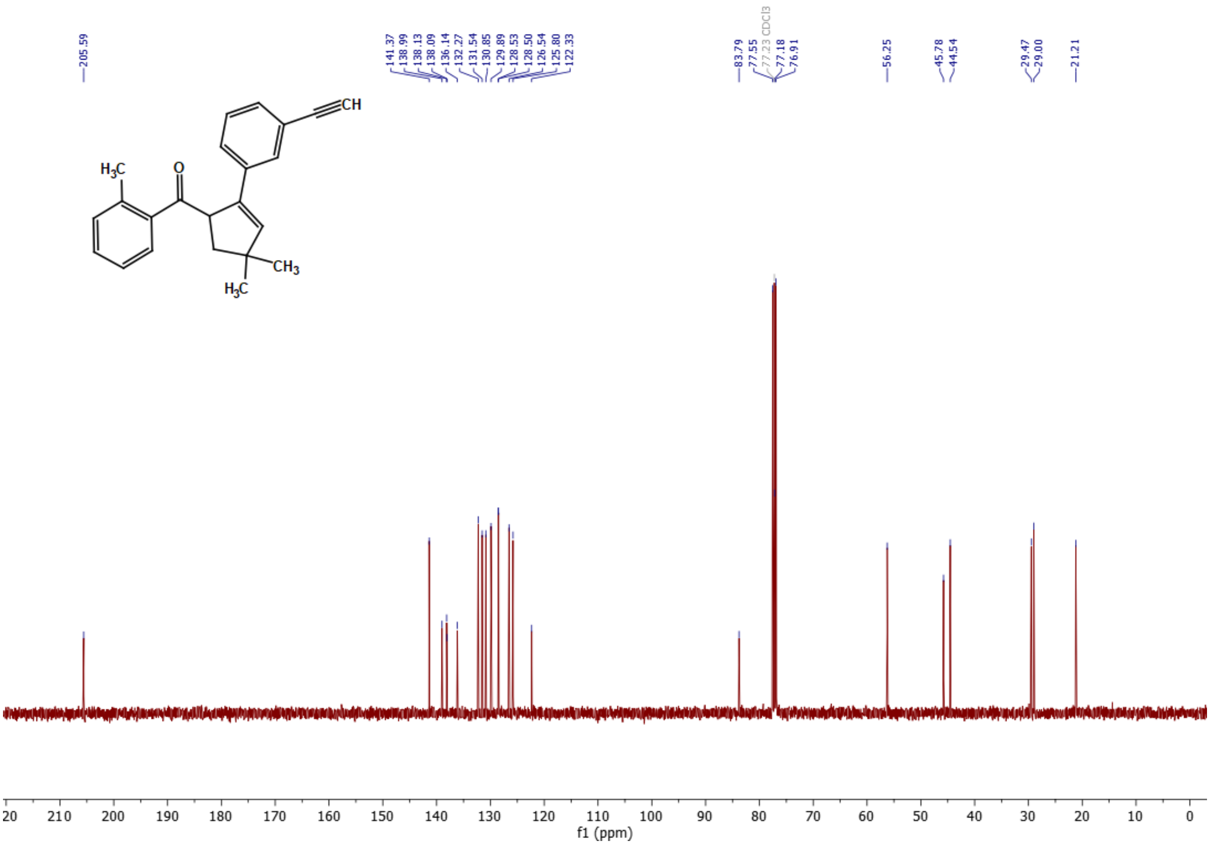

DEPT-135 NMR (101 MHz, Chloroform-*d*) (**3af**):

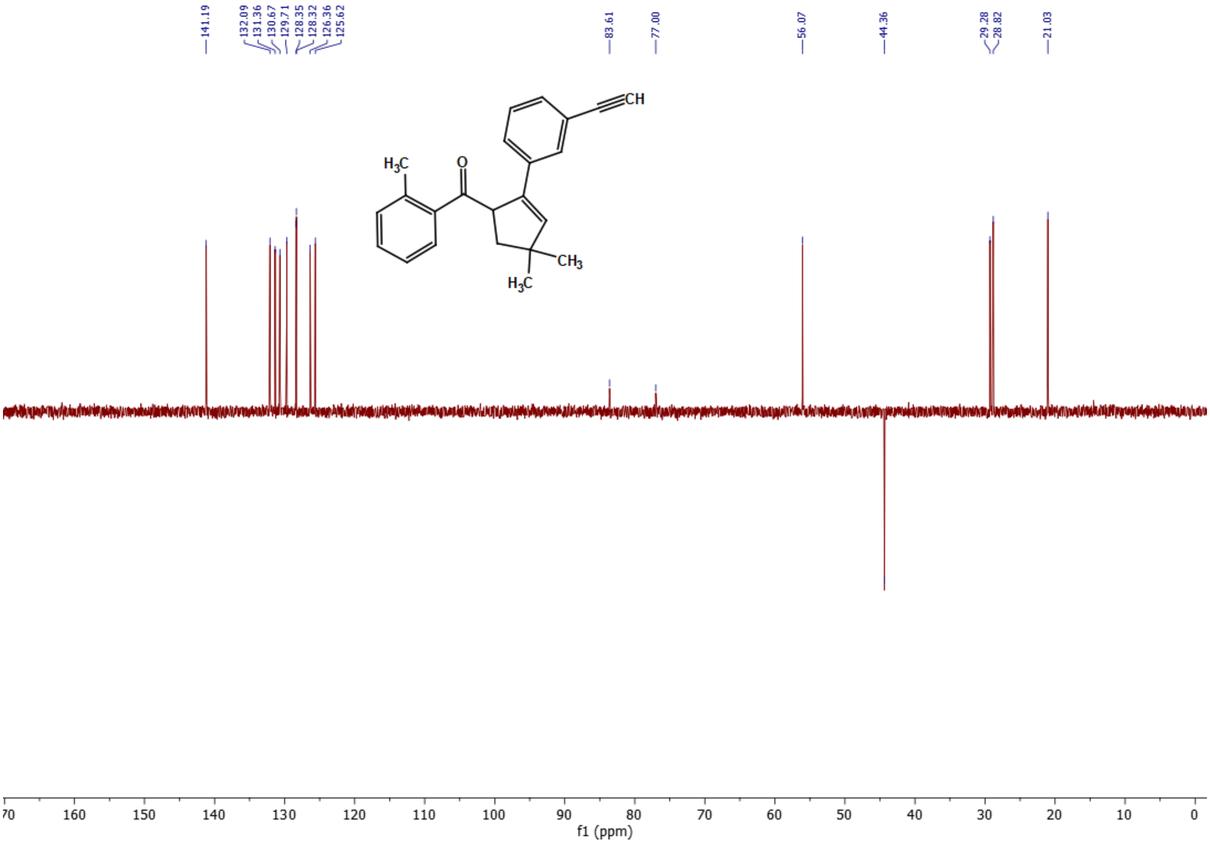

^{1}H NMR (400 MHz, Chloroform-*d*) (**3ag**):

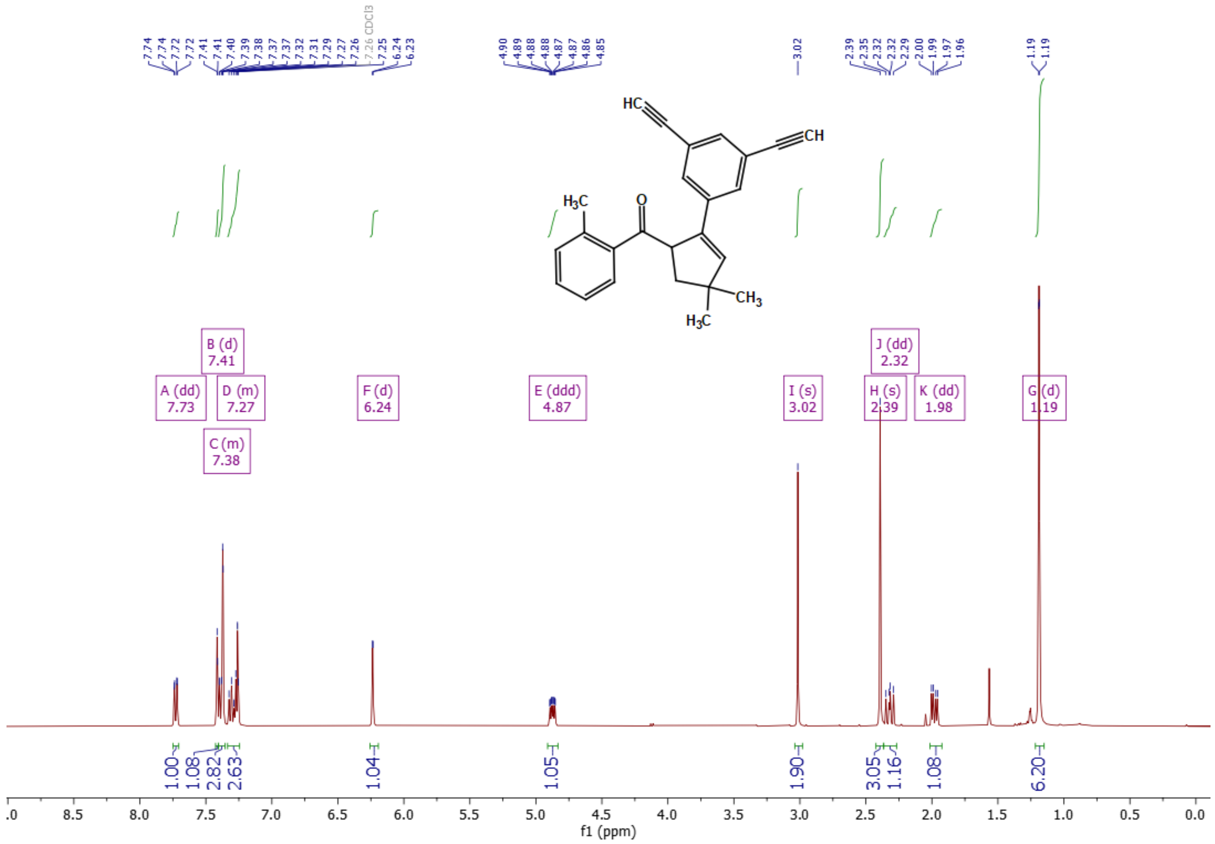

^{13}C NMR (101 MHz, Chloroform-*d*) (**3ag**):

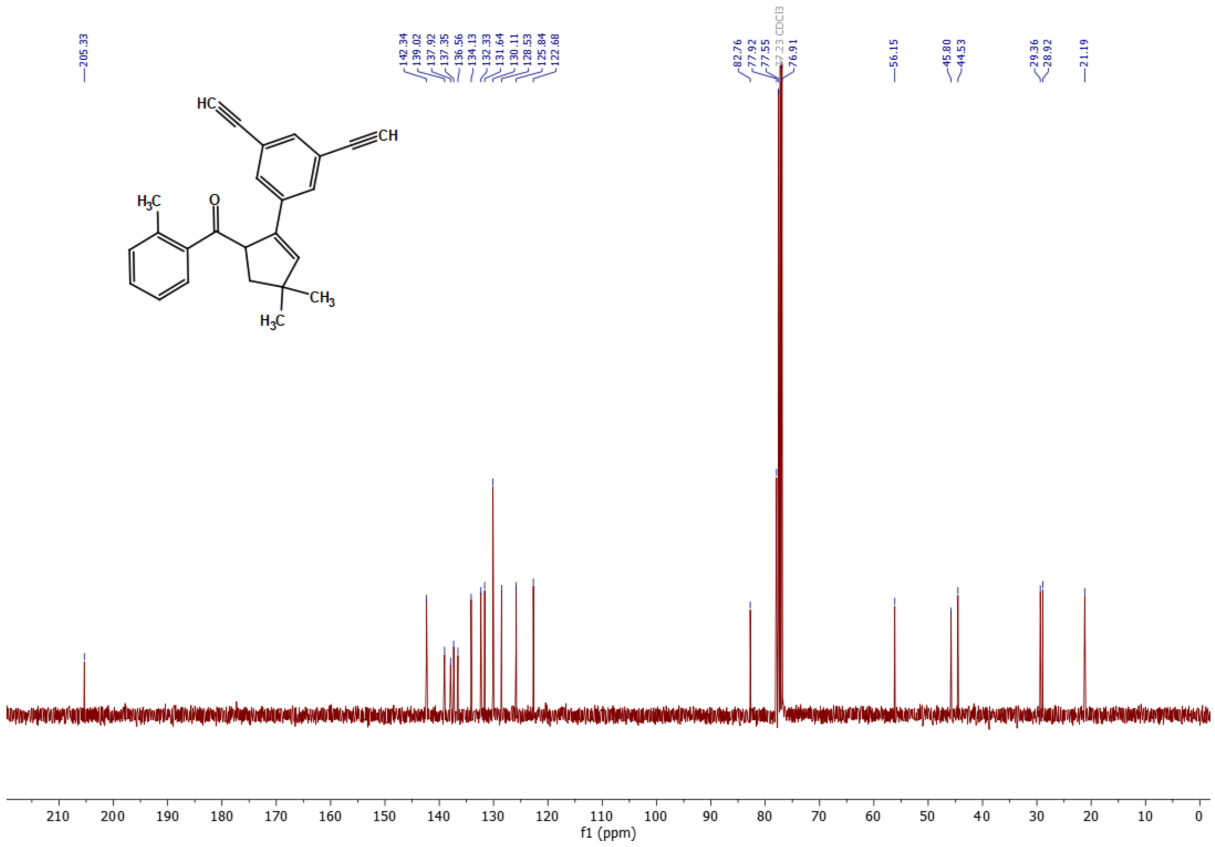

DEPT-135 NMR (101 MHz, Chloroform-*d*) (**3ag**):

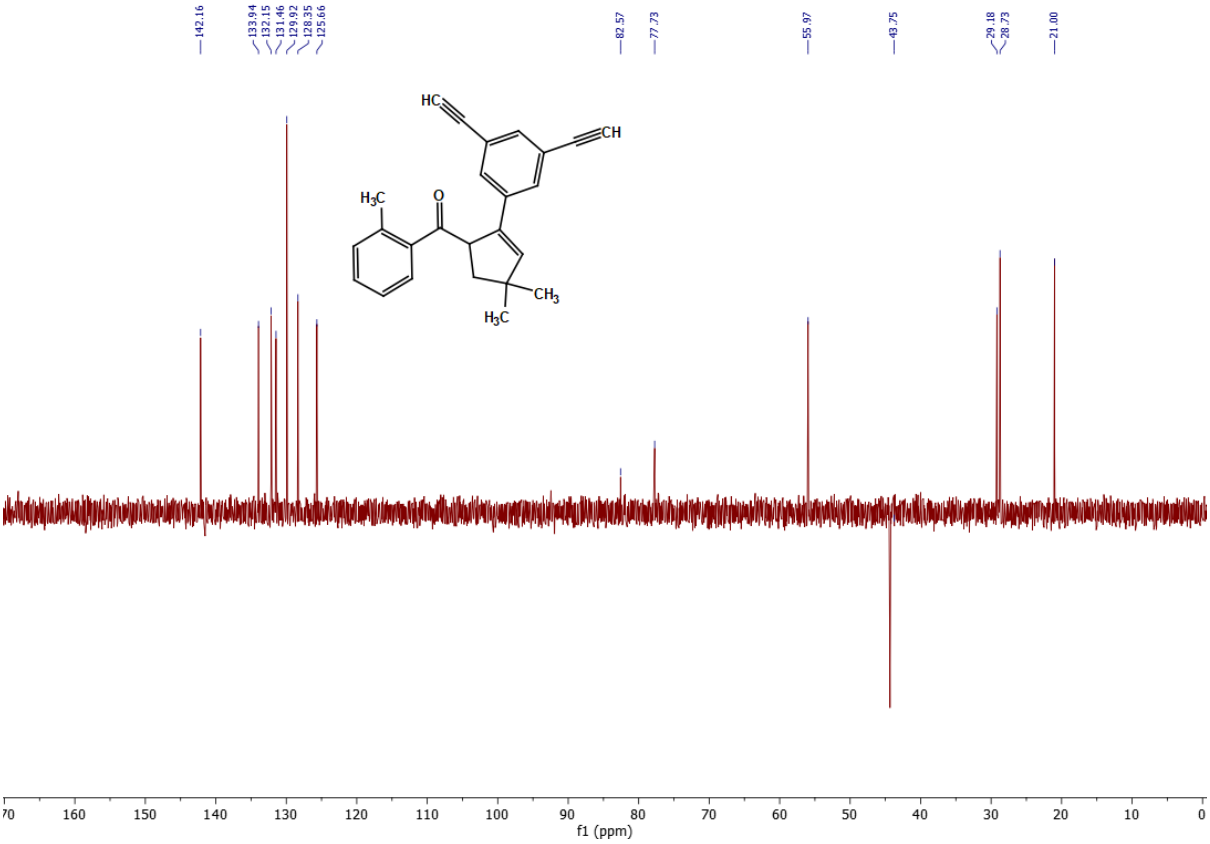

^{1}H NMR (400 MHz, Chloroform-*d*) (**3ah**):

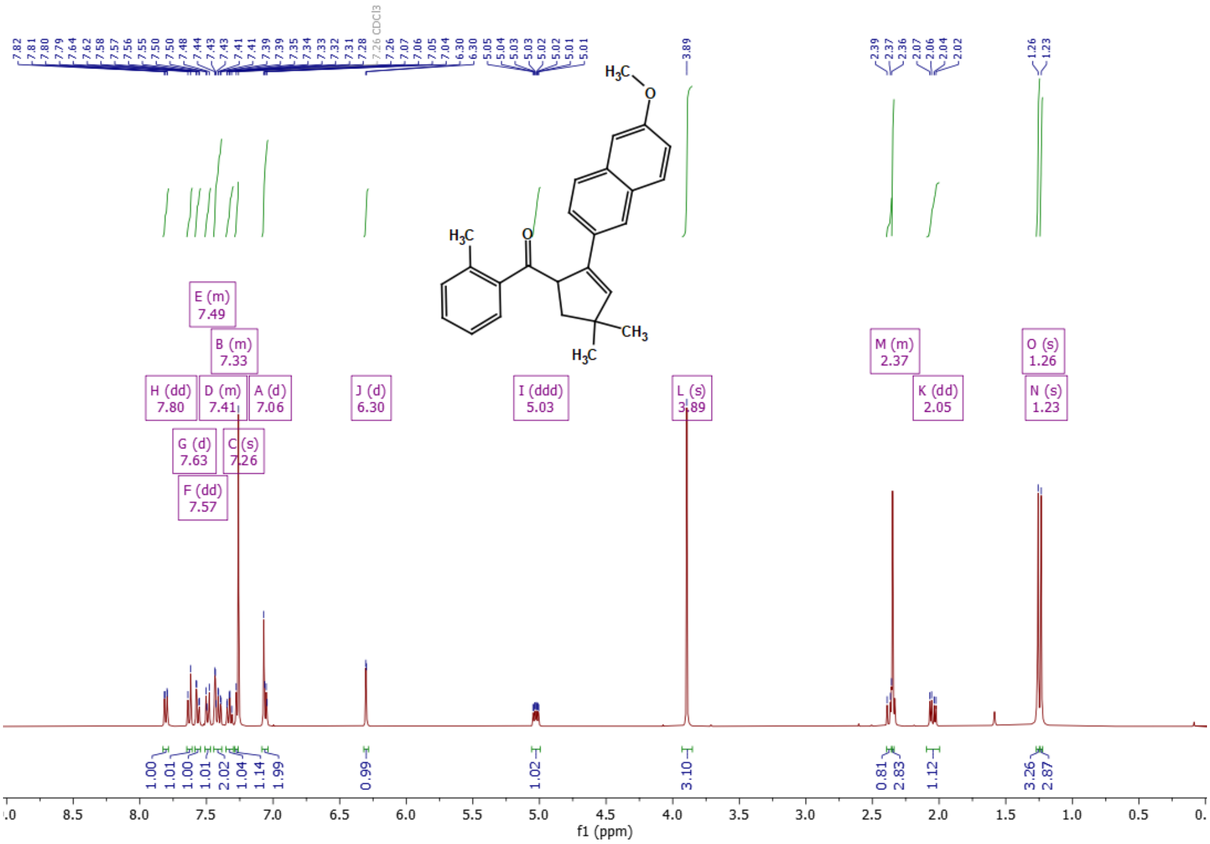

^{13}C NMR (101 MHz, Chloroform-*d*) (**3ah**):

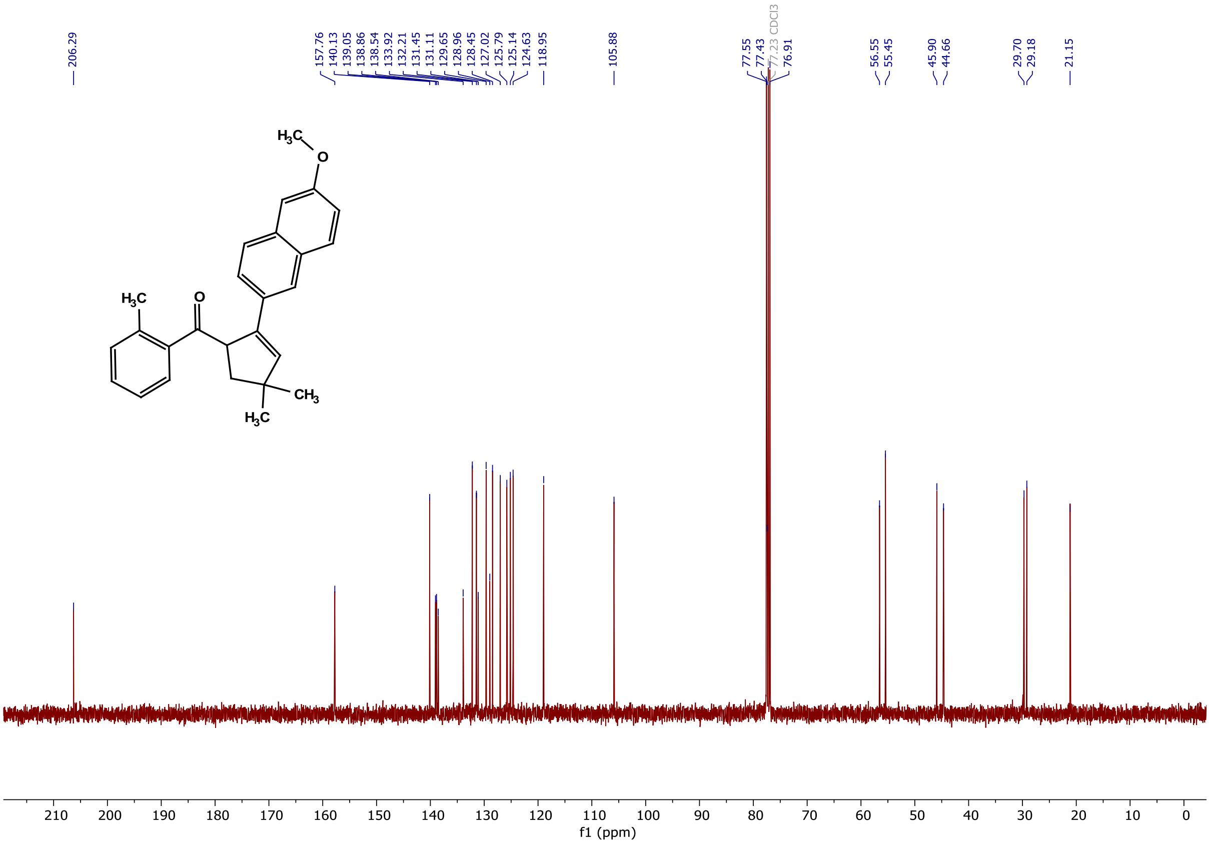

DEPT-135 NMR (101 MHz, Chloroform-*d*) (**3ah**):

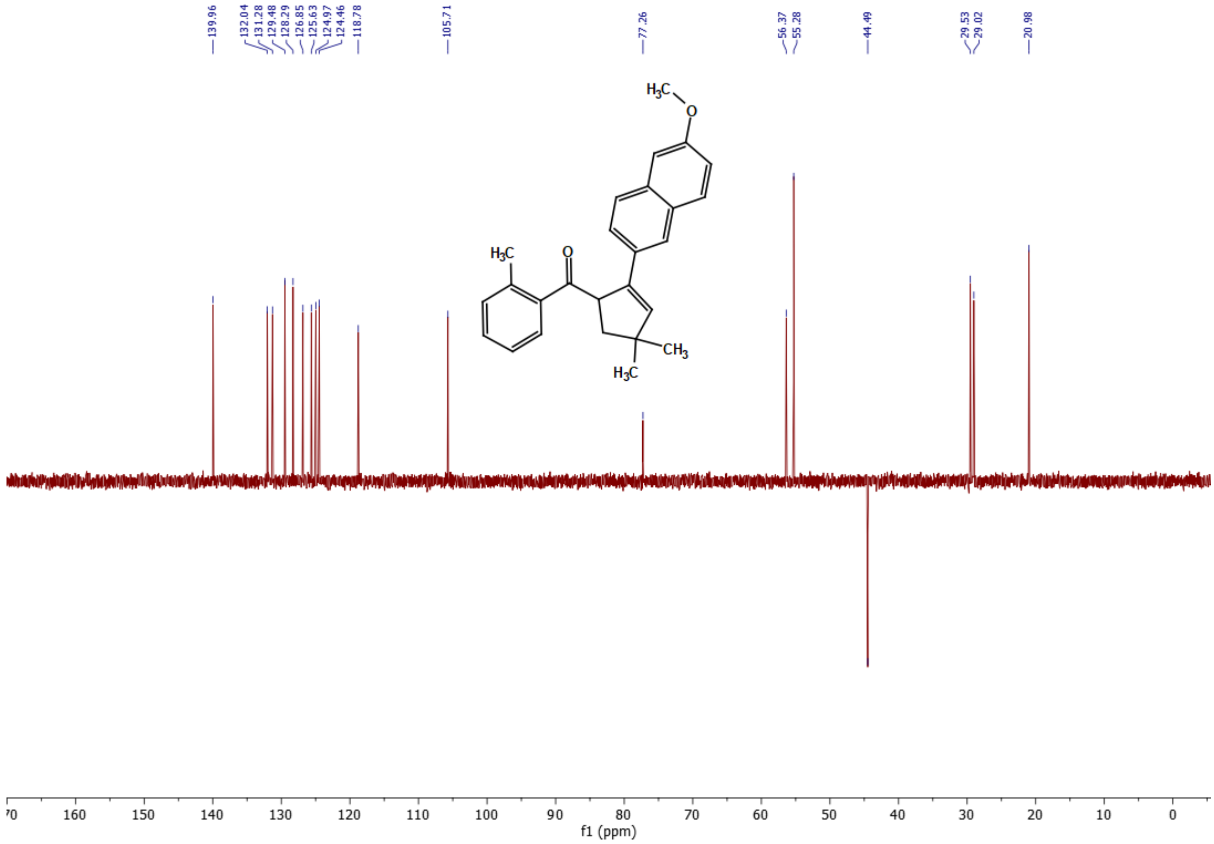

^{1}H NMR (500 MHz, Chloroform-*d*) (**3ai**):

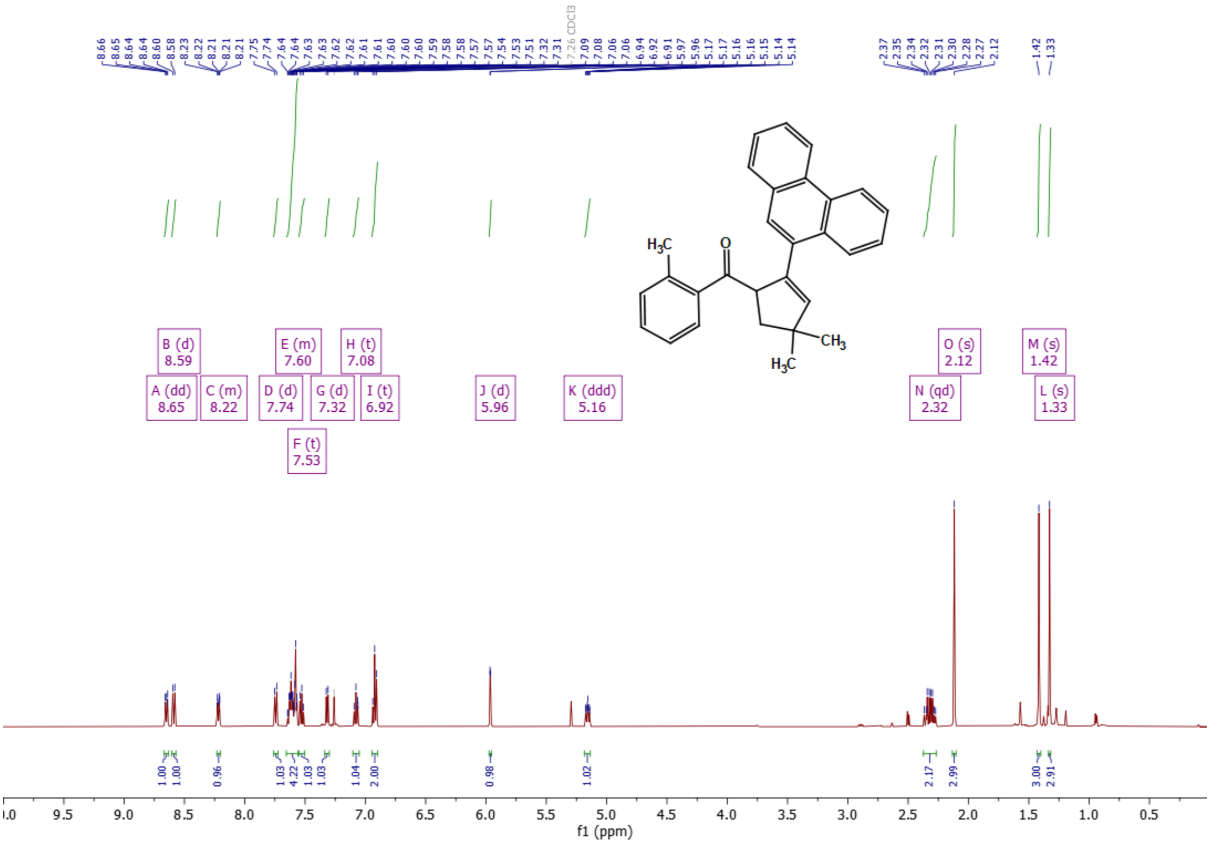

^{13}C NMR (101 MHz, Chloroform-*d*) (**3ai**):

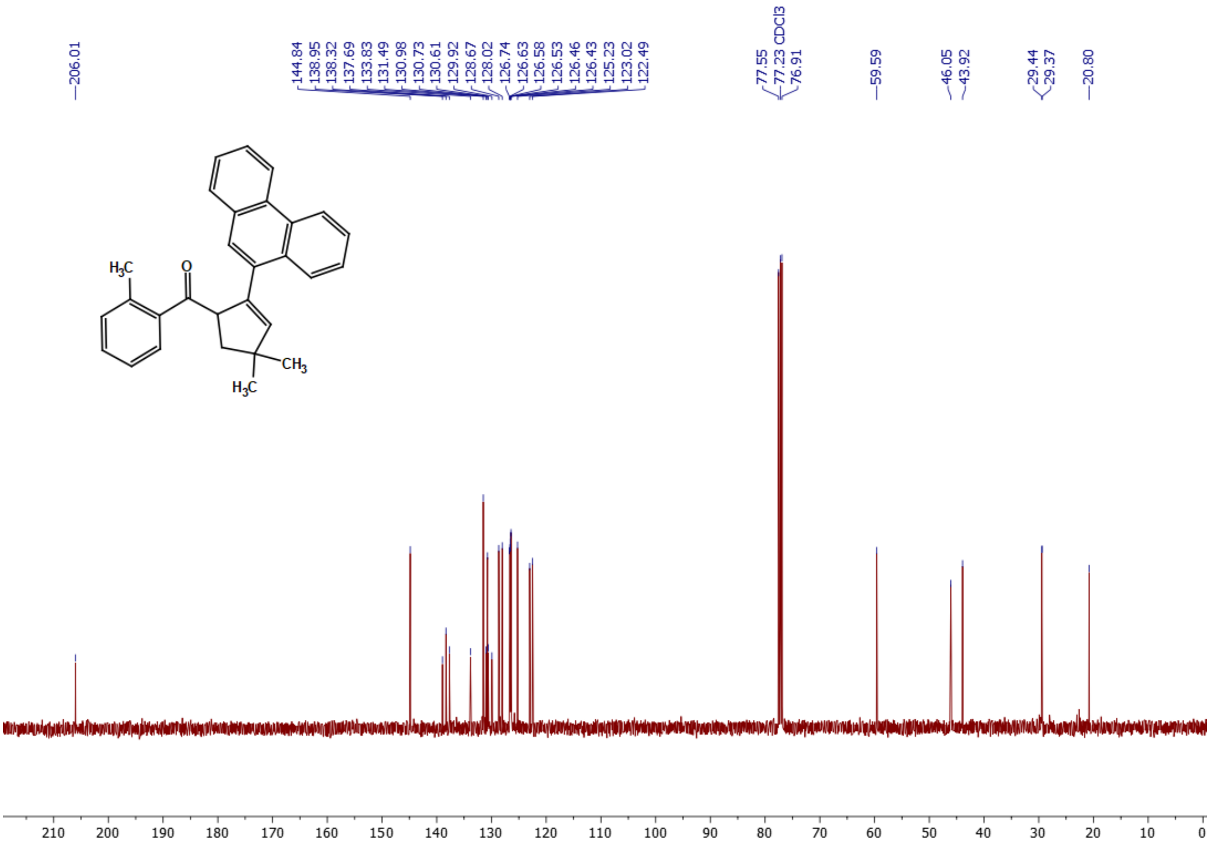

^{1}H NMR (400 MHz, Chloroform-*d*) (**3aj**):

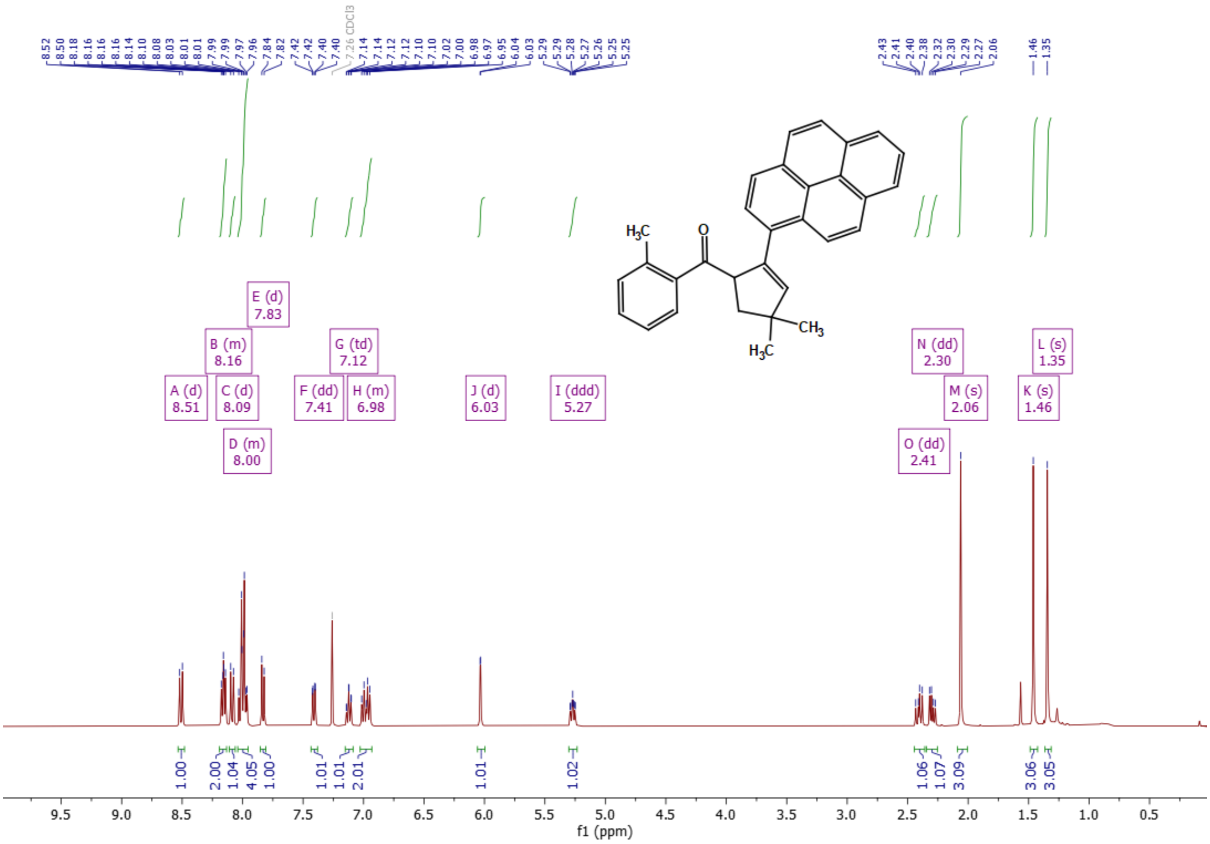

^{13}C NMR (101 MHz, Chloroform-*d*) (**3aj**):

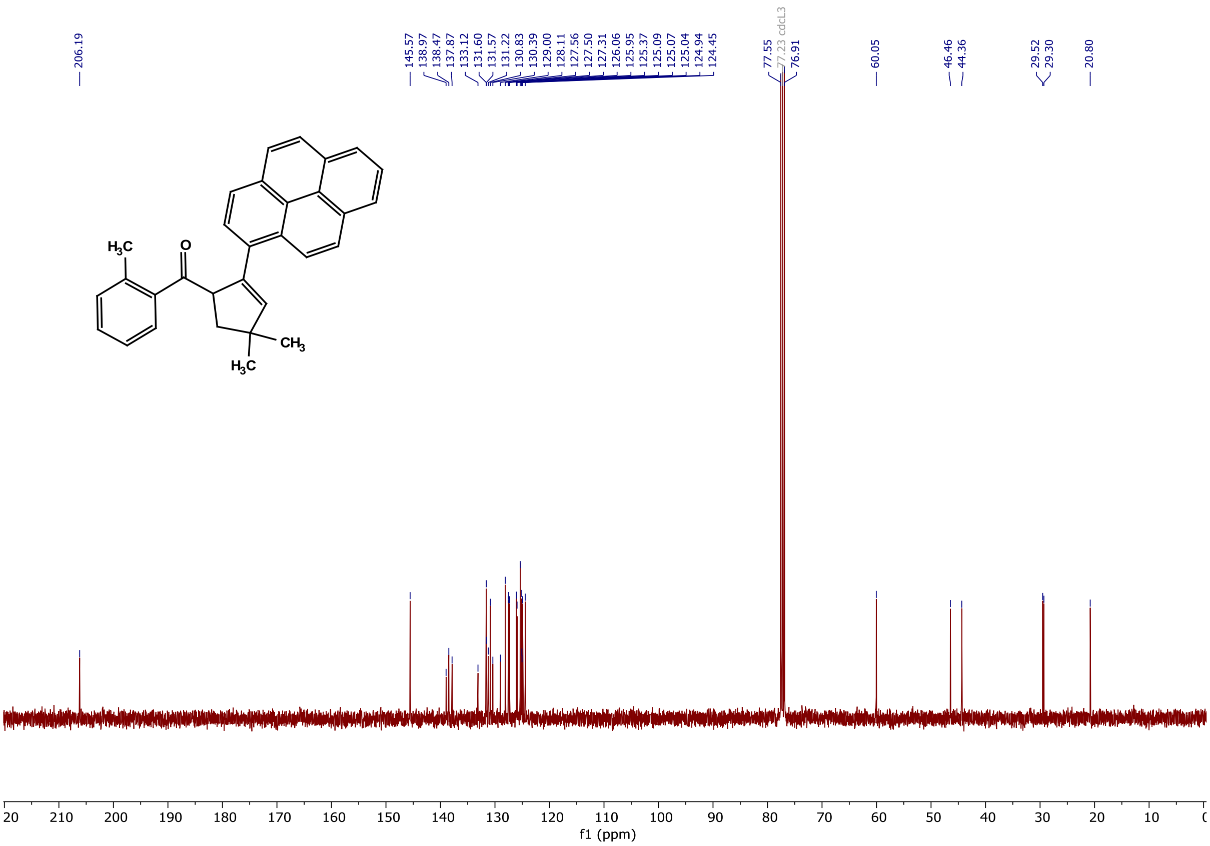

DEPT-135 NMR (101 MHz, Chloroform-*d*) (**3aj**):

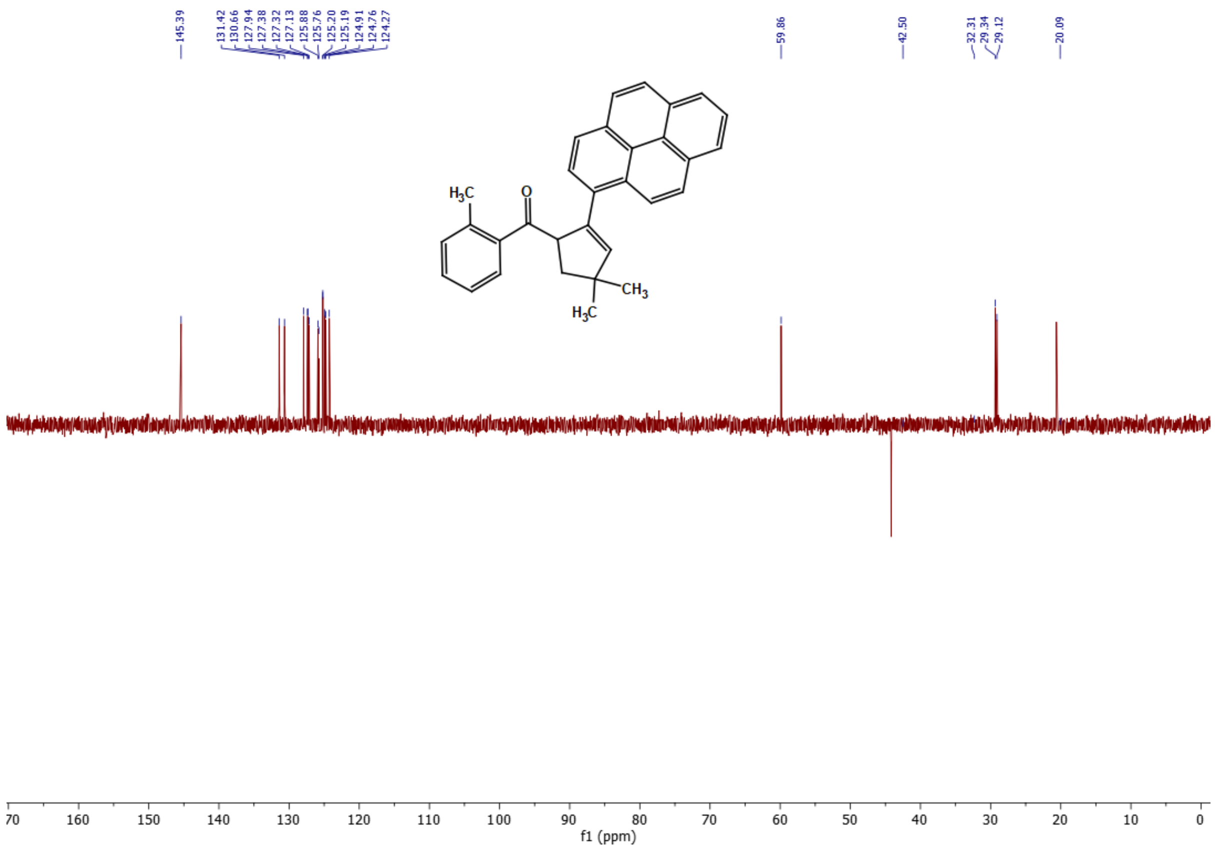

^{1}H NMR (400 MHz, Chloroform-*d*) (**3ak**):

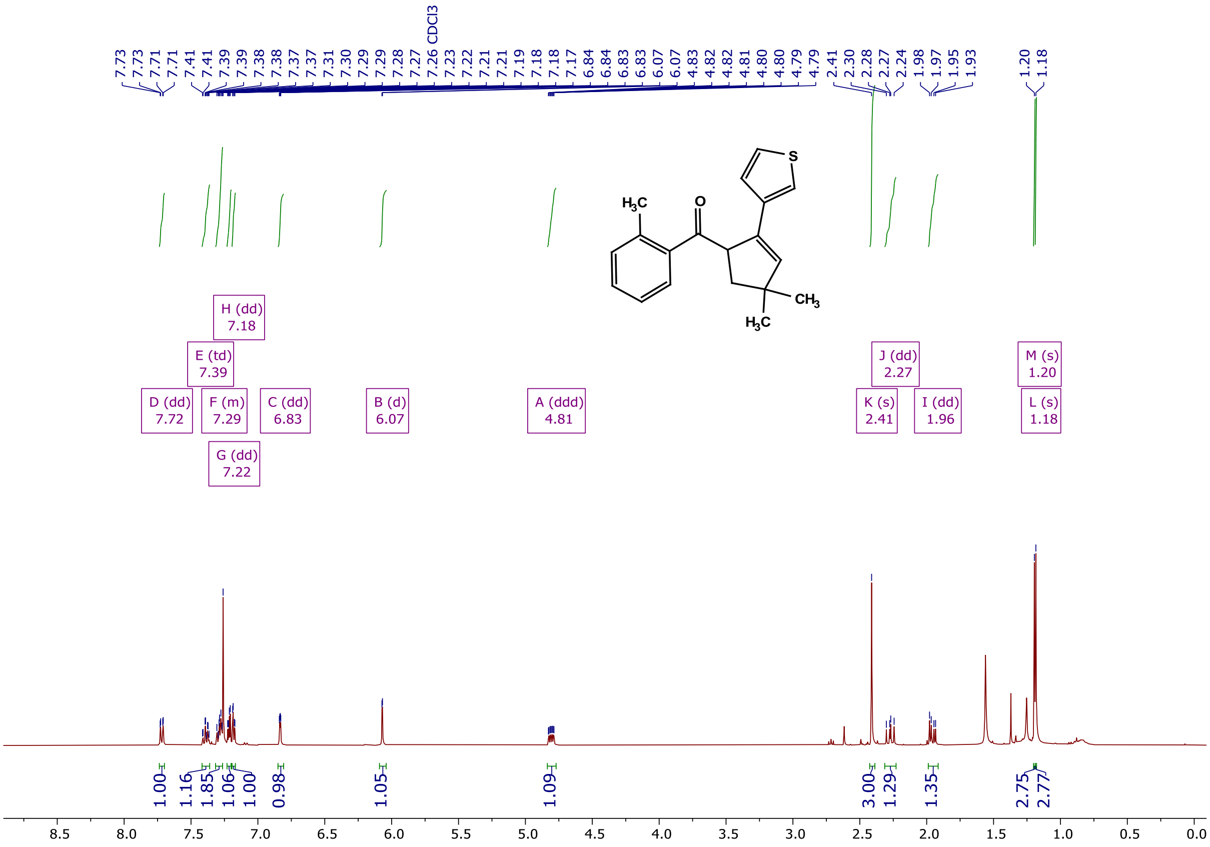

^{13}C NMR (101 MHz, Chloroform-*d*) (**3ak**):

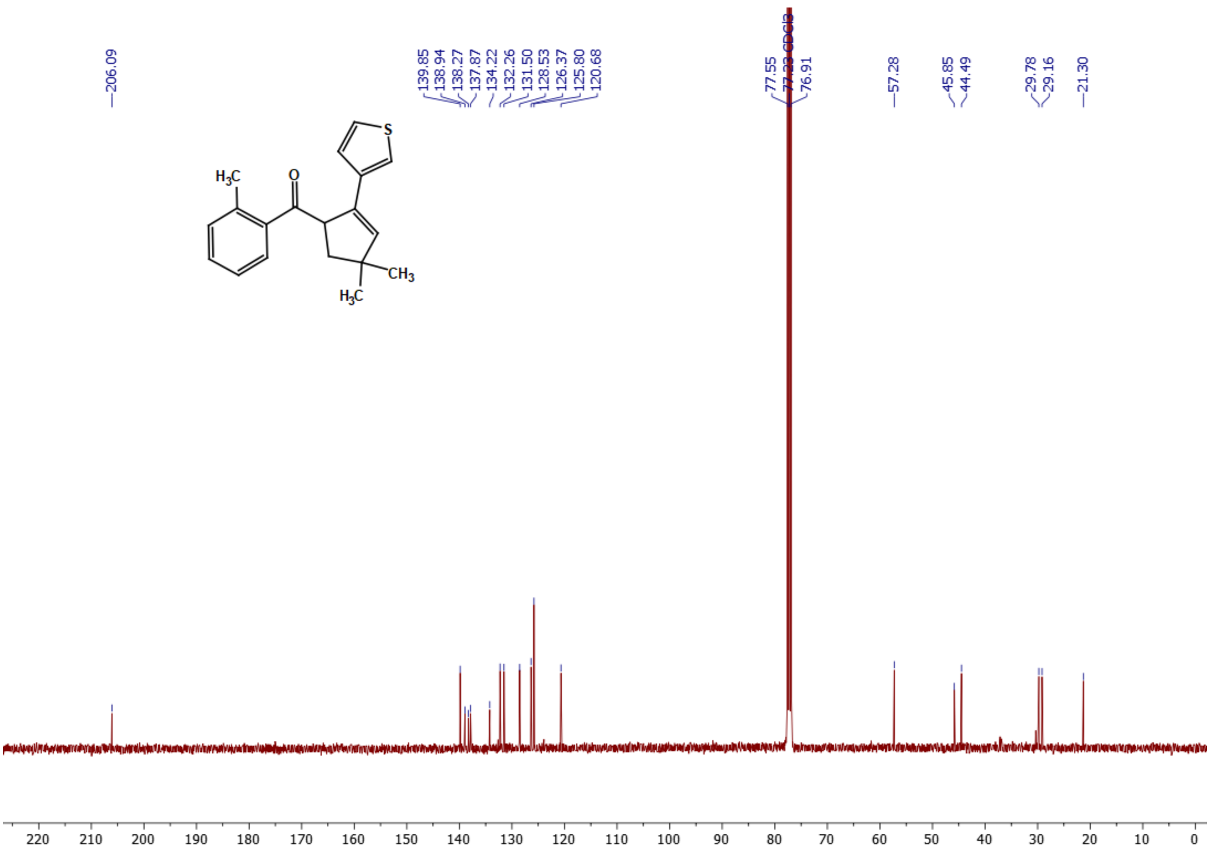

DEPT-135 NMR (101 MHz, Chloroform-*d*) (**3ak**):

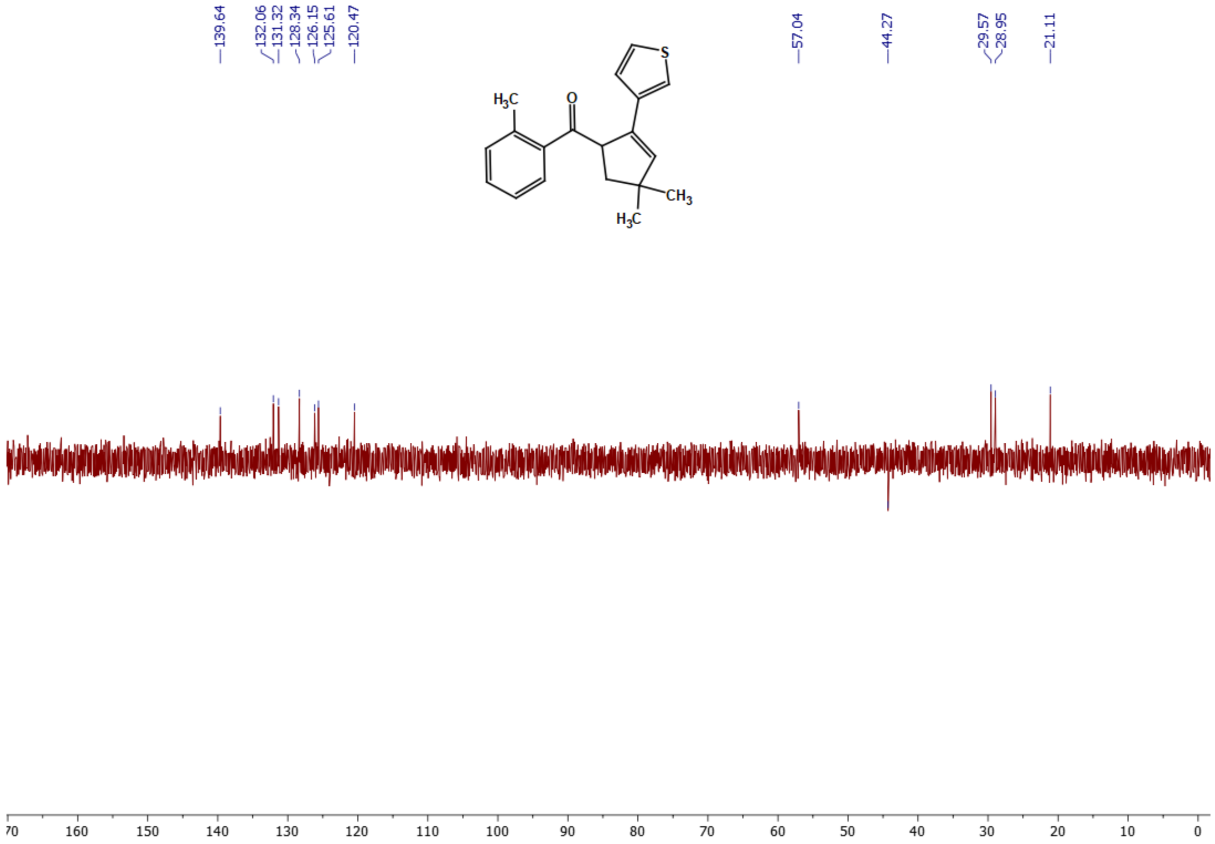

^{1}H NMR (400 MHz, Chloroform-*d*) (**3al**):

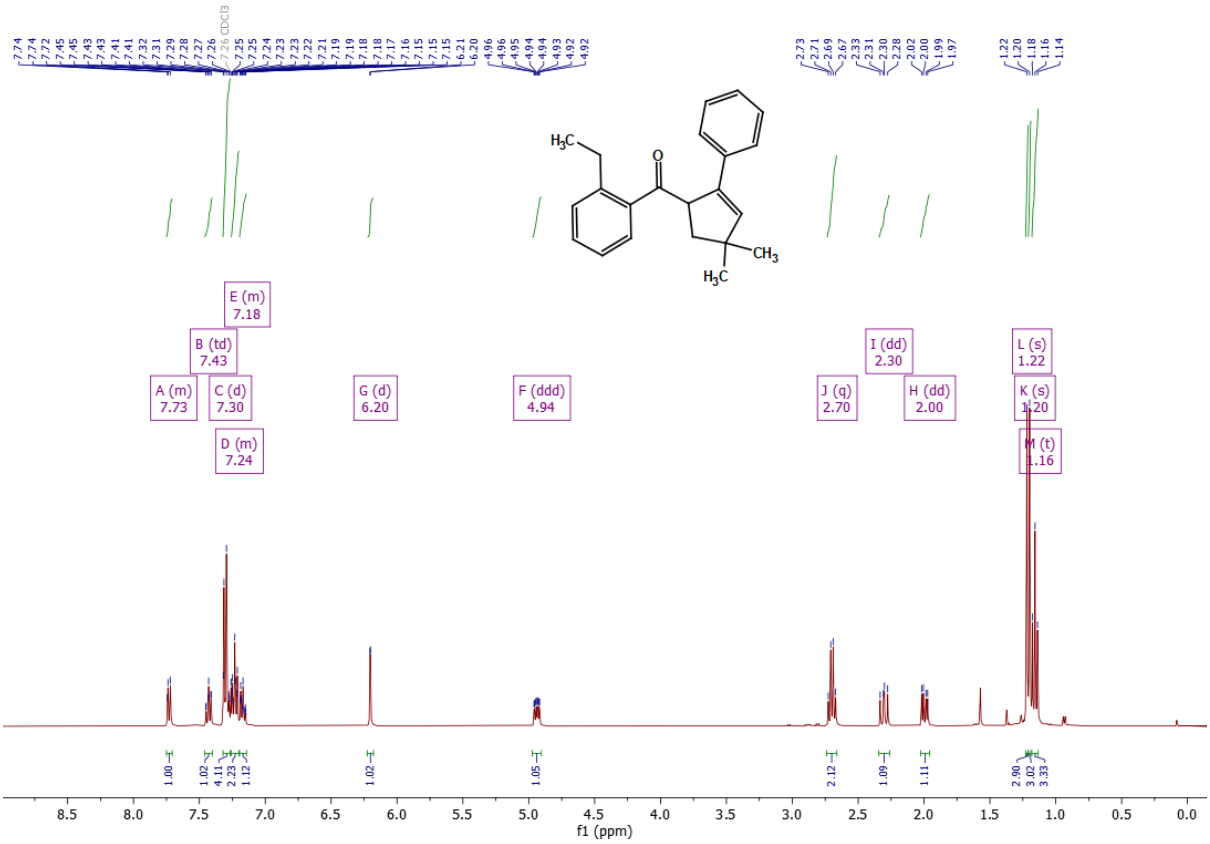

^{13}C NMR (101 MHz, Chloroform-*d*) (**3al**):

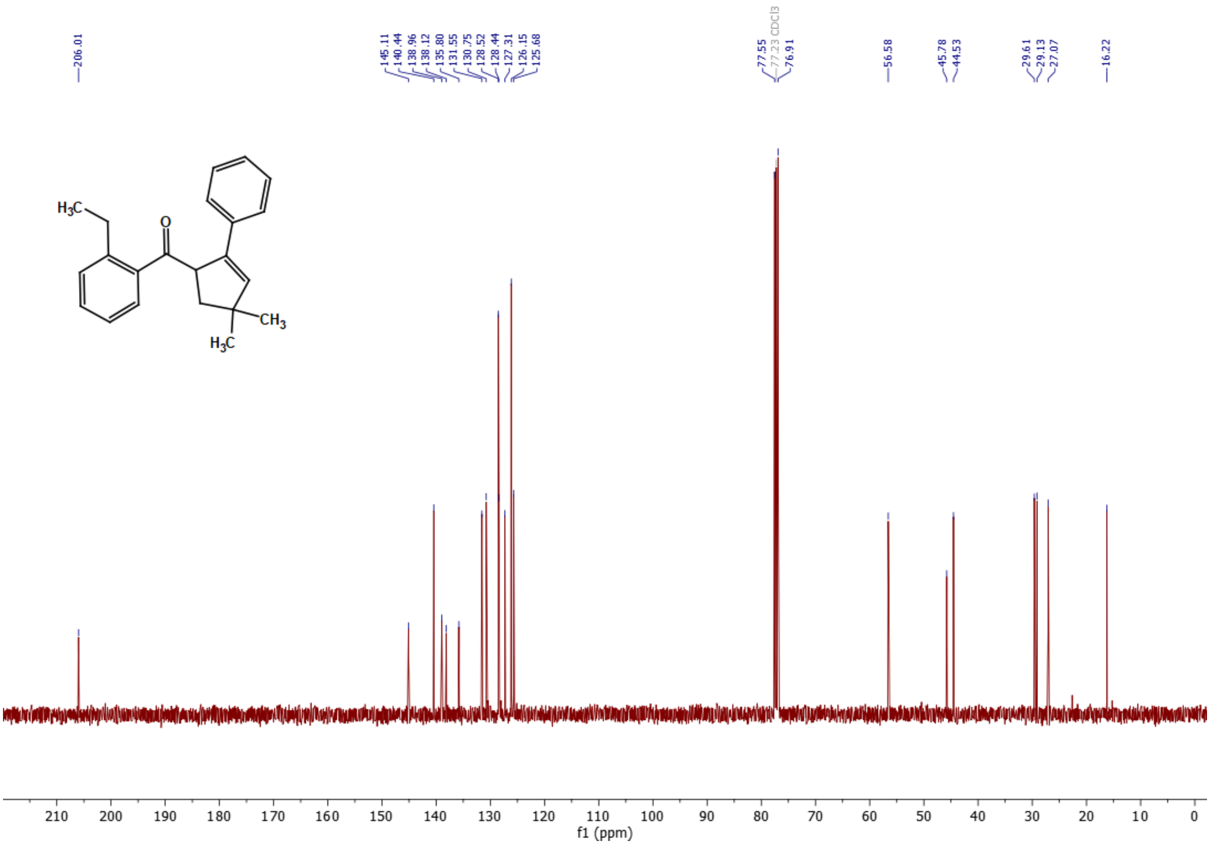

DEPT-135 NMR (101 MHz, Chloroform-*d*) (**3al**):

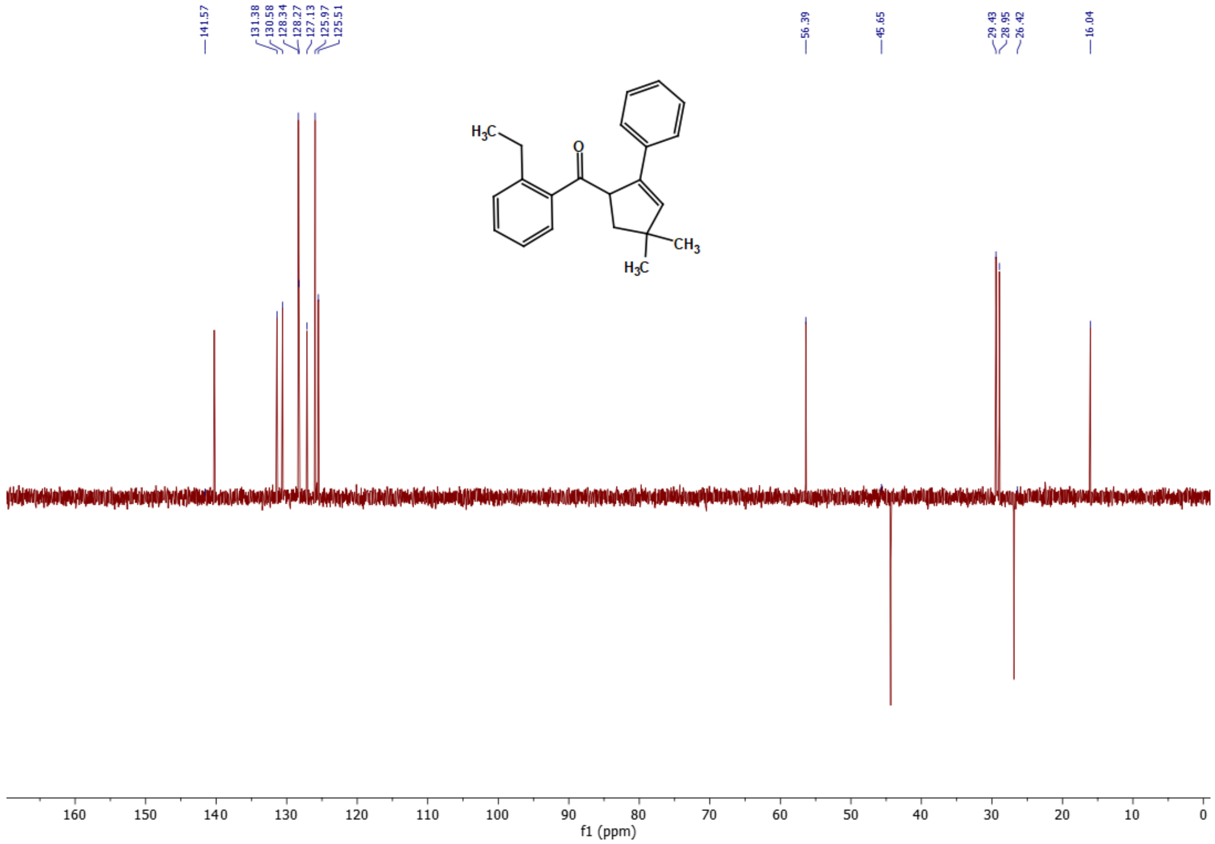

^{1}H NMR (400 MHz, Chloroform-*d*) (**3am**):

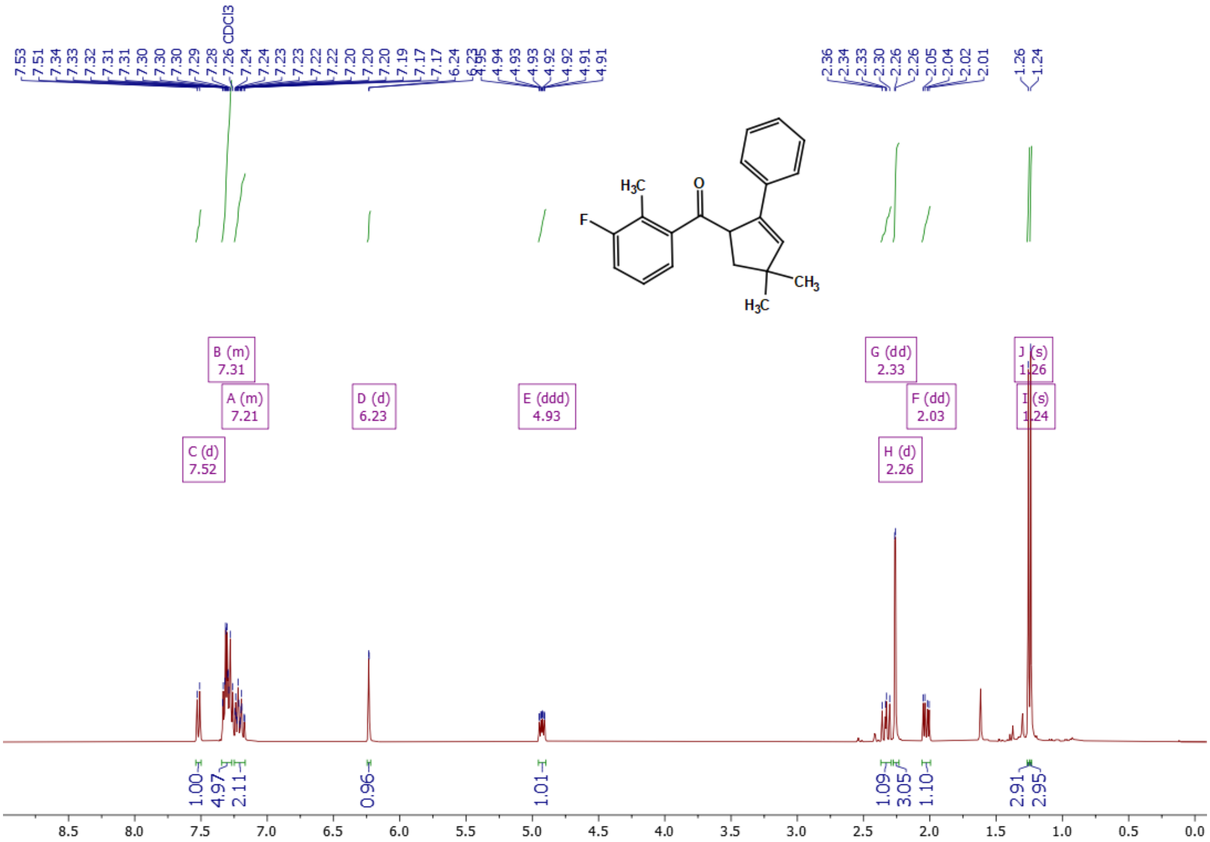

^{13}C NMR (101 MHz, Chloroform-*d*) (**3am**):

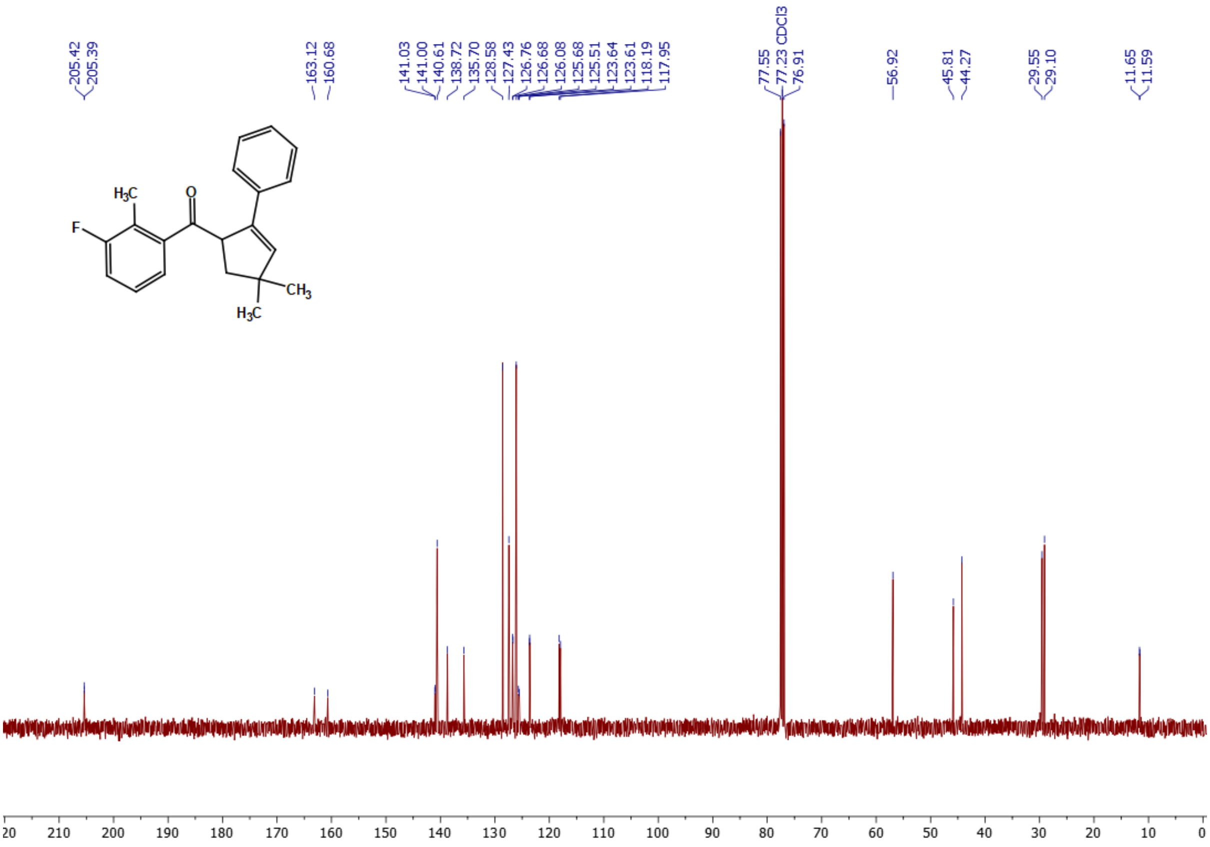

DEPT-135 NMR (101 MHz, Chloroform-*d*) (**3am**):

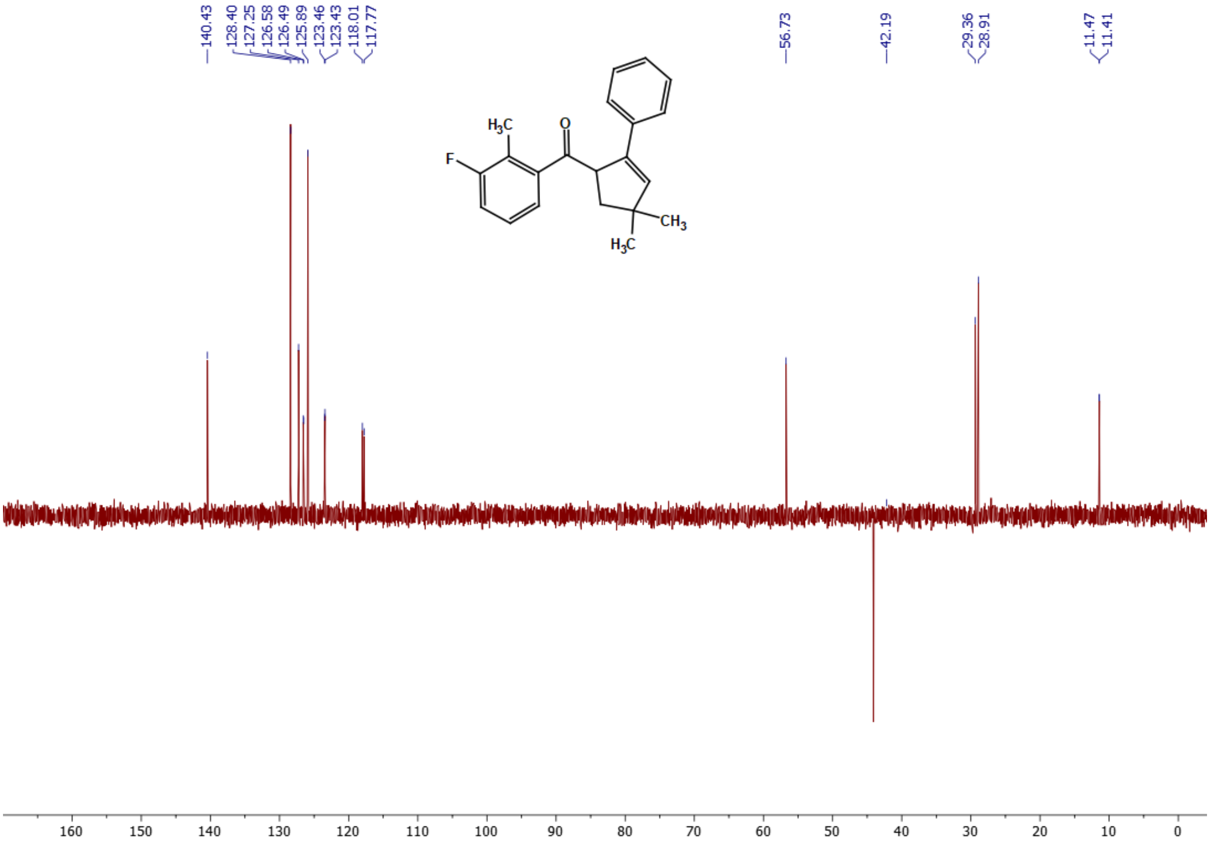

^{19}F NMR (376 MHz, Chloroform-*d*) (**3am**):

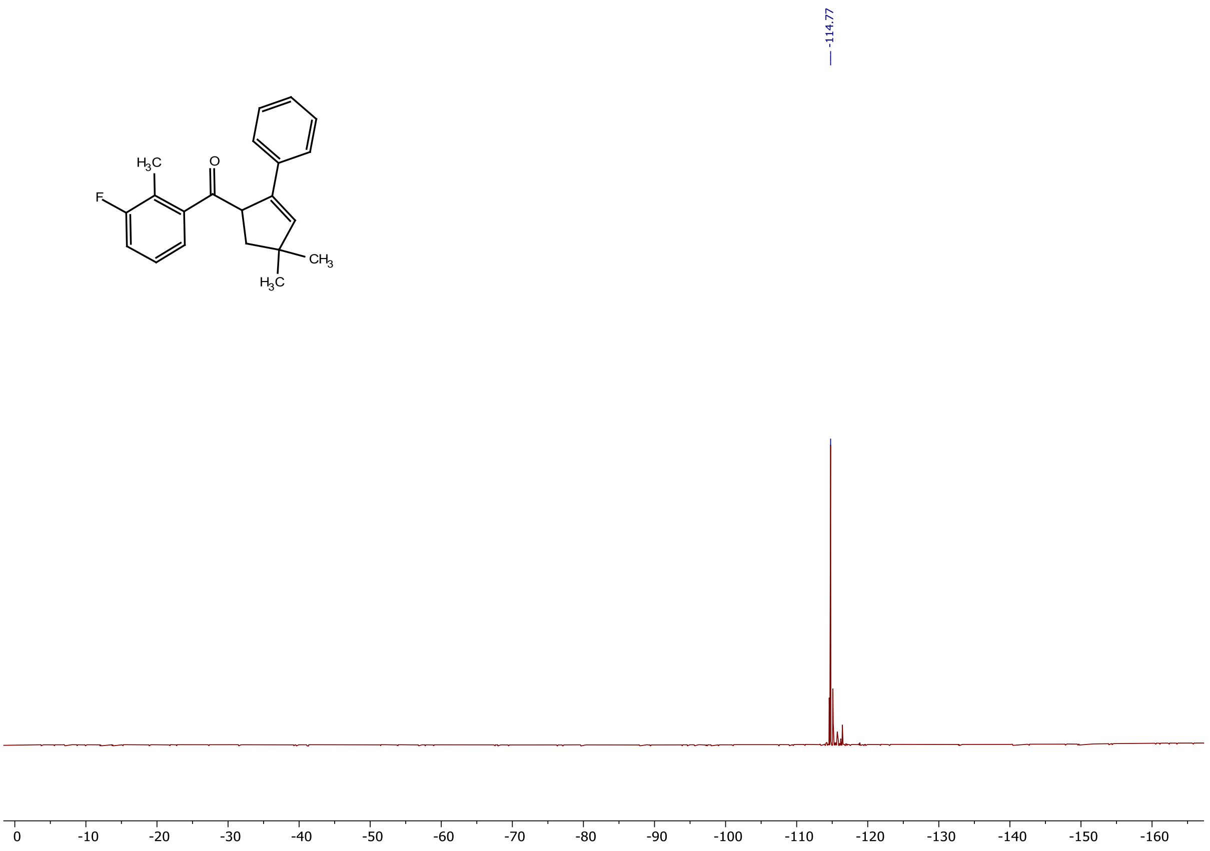

^{1}H NMR (400 MHz, Chloroform-*d*) (**3an**):

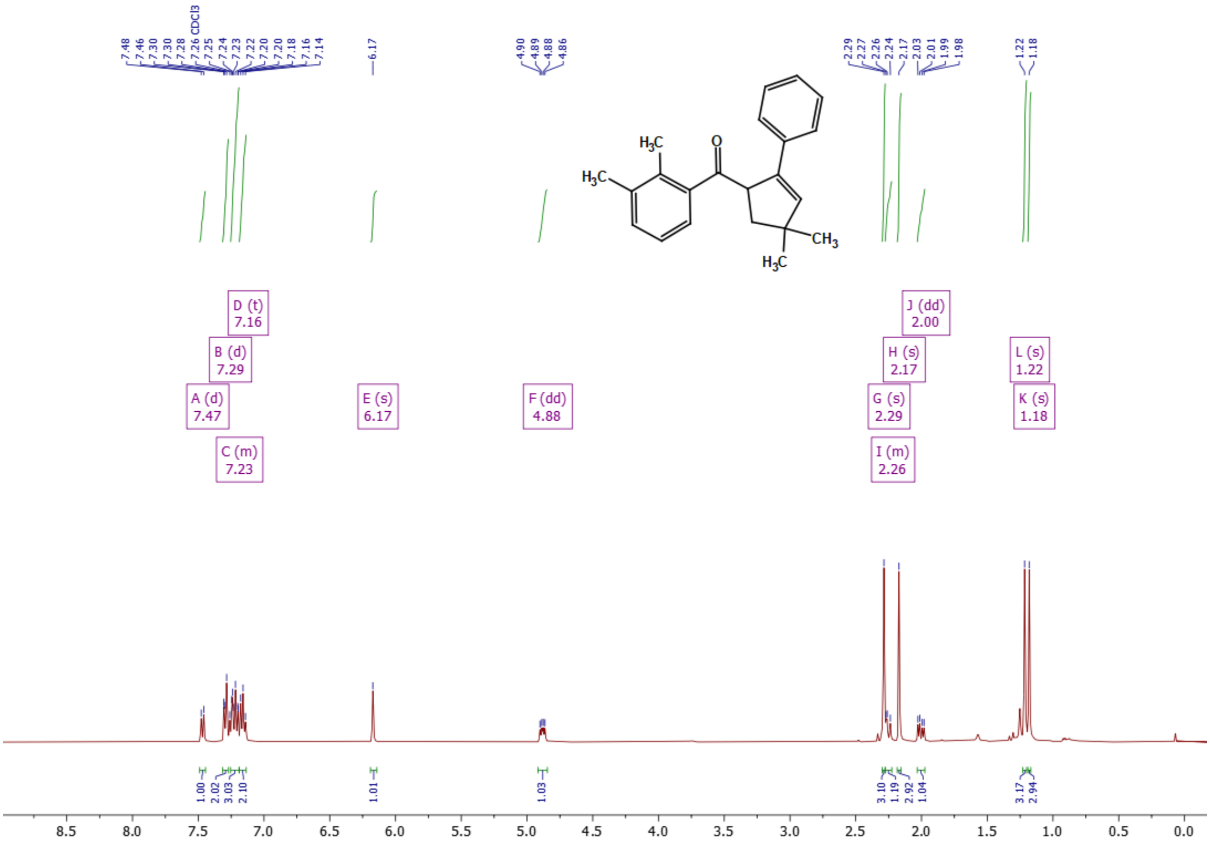

^{13}C NMR (101 MHz, Chloroform-*d*) (**3an**):

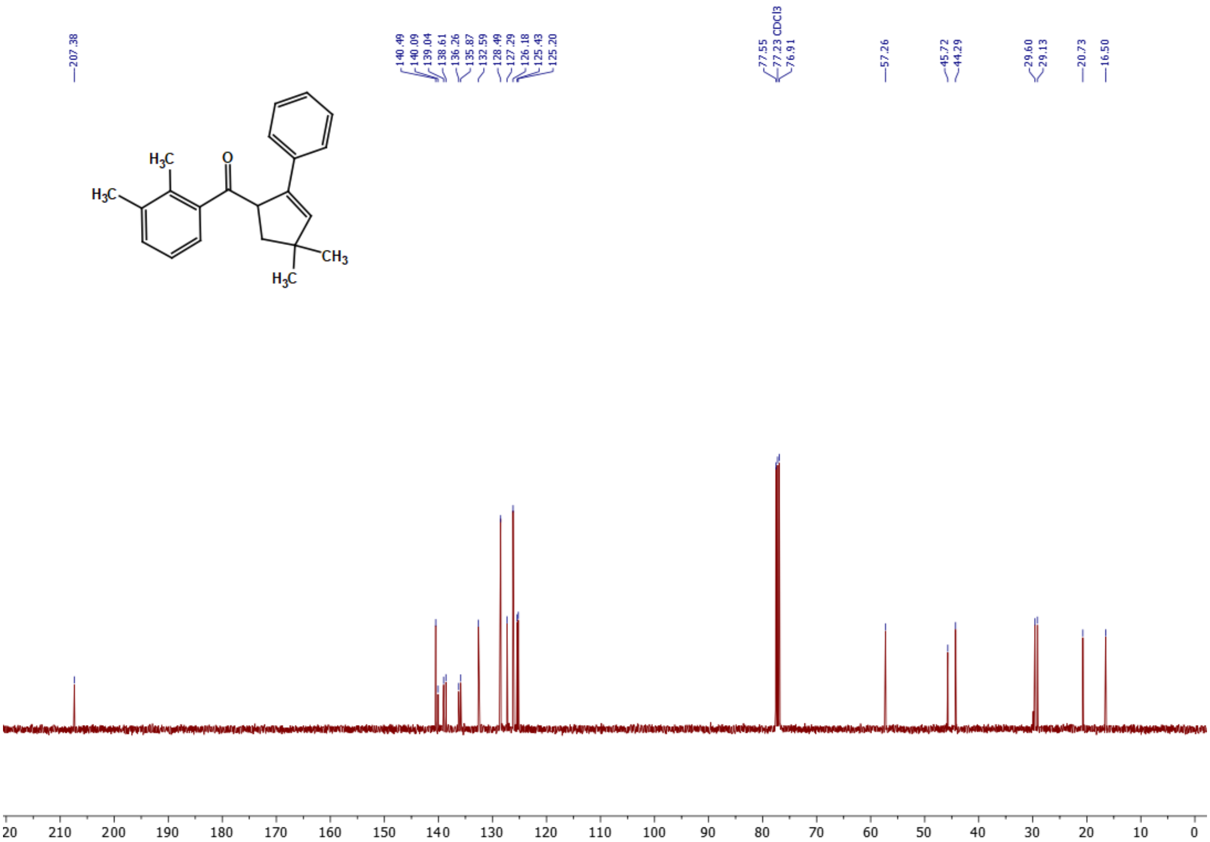

DEPT-135 NMR (101 MHz, Chloroform-*d*) (**3an**):

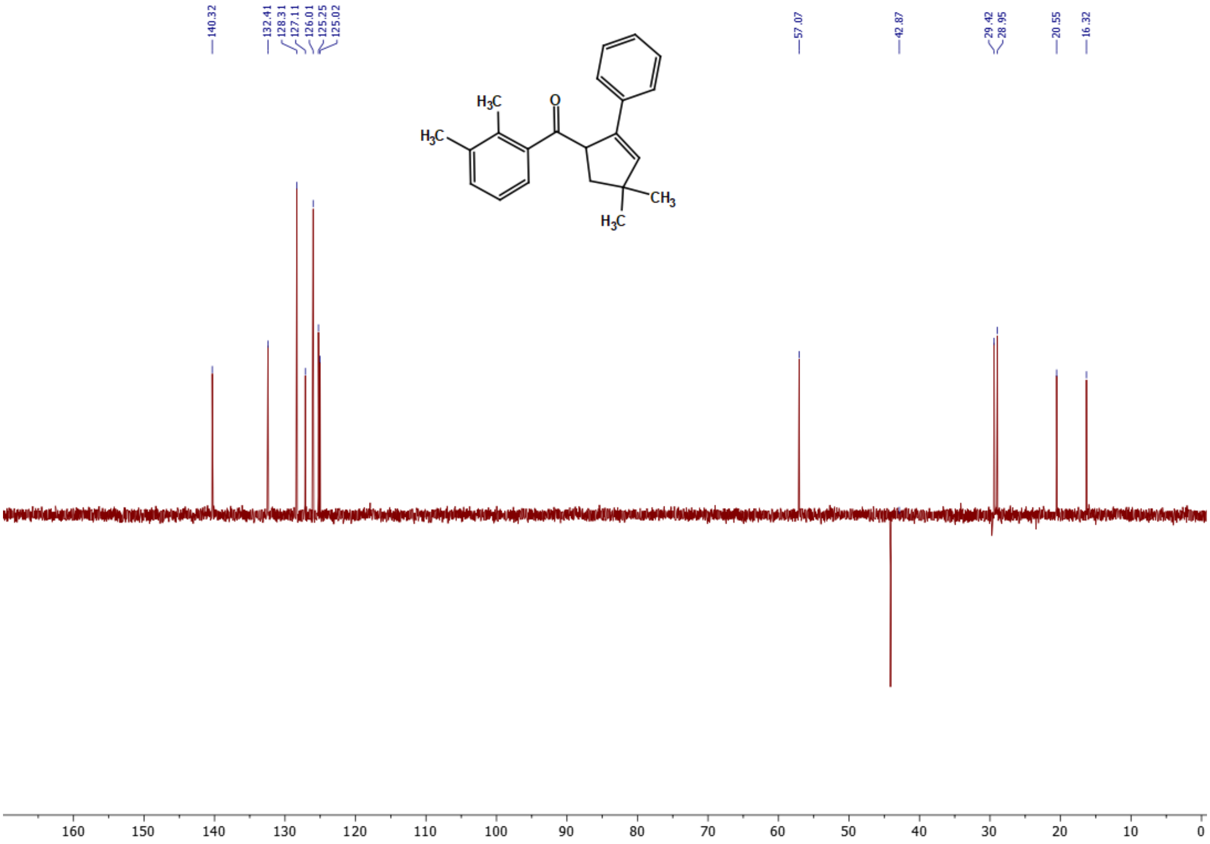

^{1}H NMR (400 MHz, Chloroform-*d*) (**3ao**):

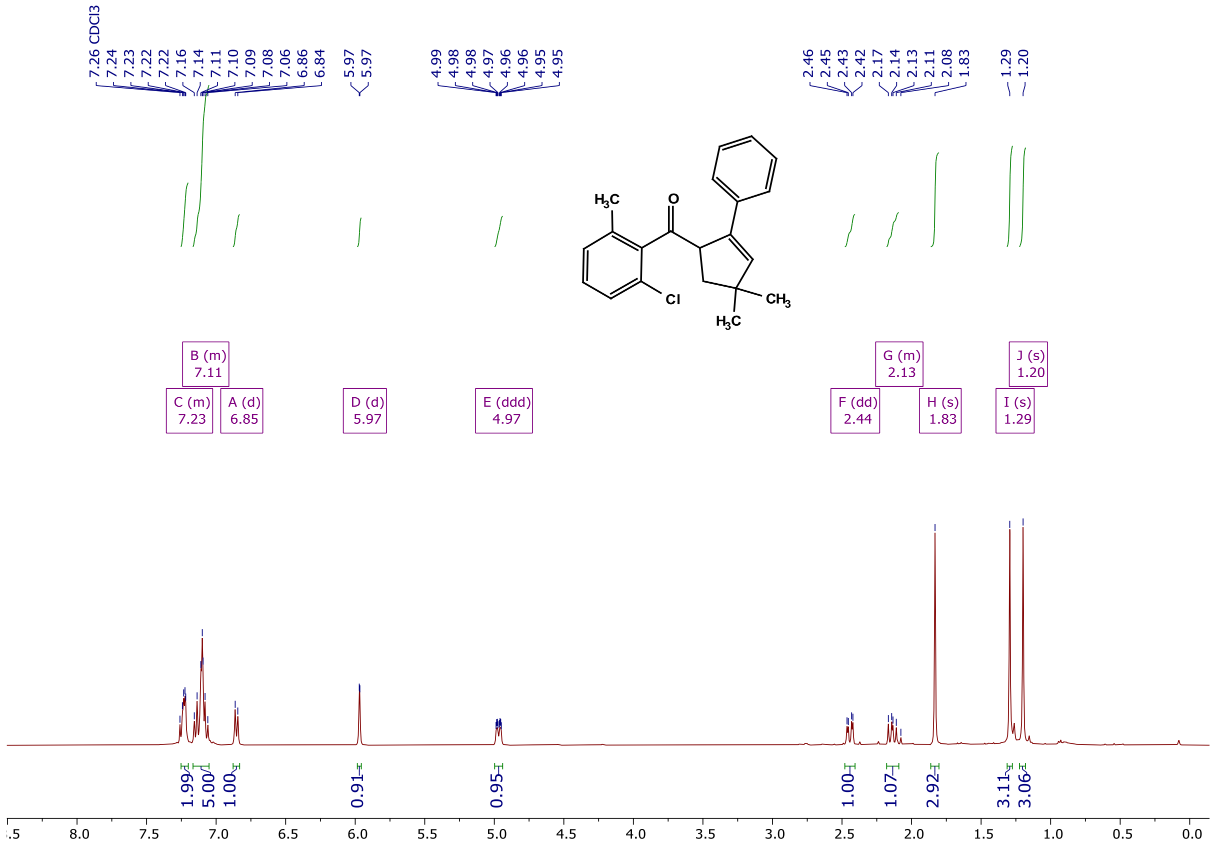

^{13}C NMR (101 MHz, Chloroform-*d*) (**3ao**):

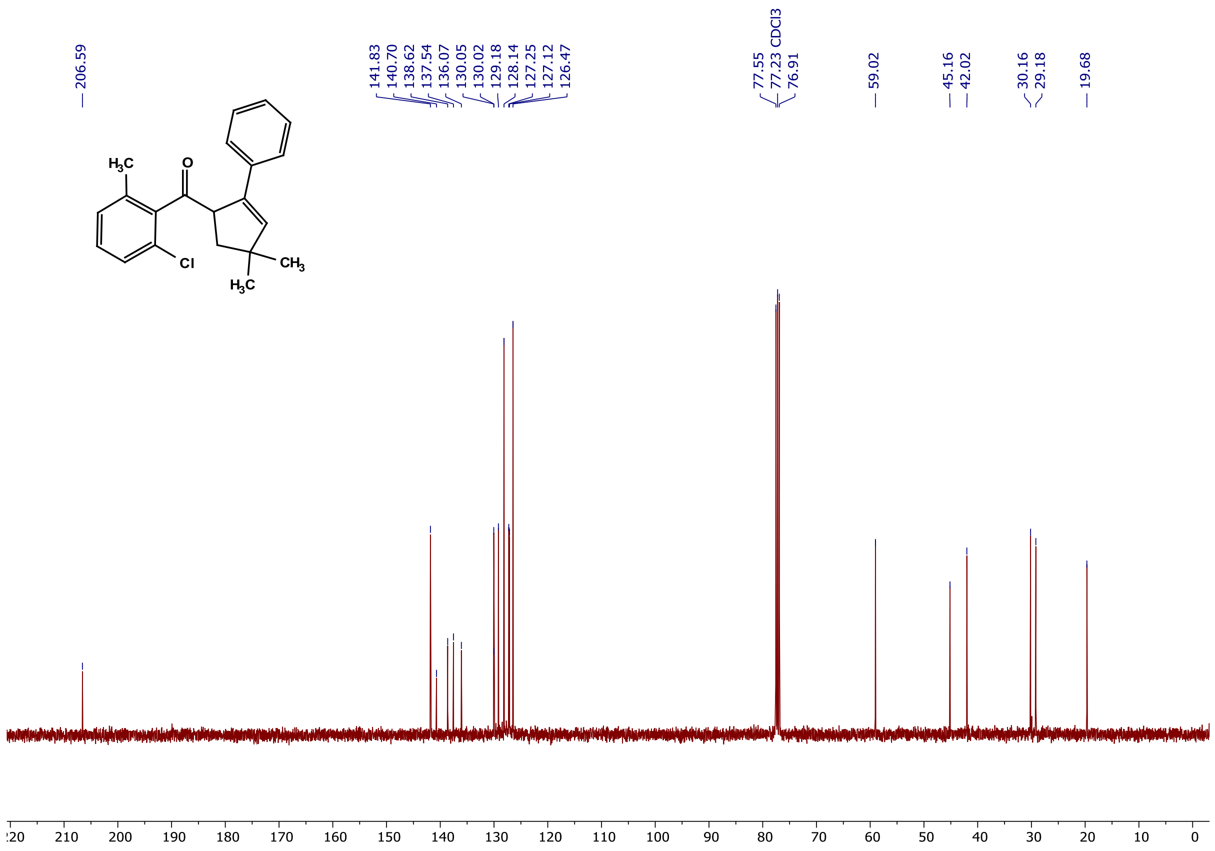

DEPT-135 NMR (101 MHz, Chloroform-*d*) (**3ao**):

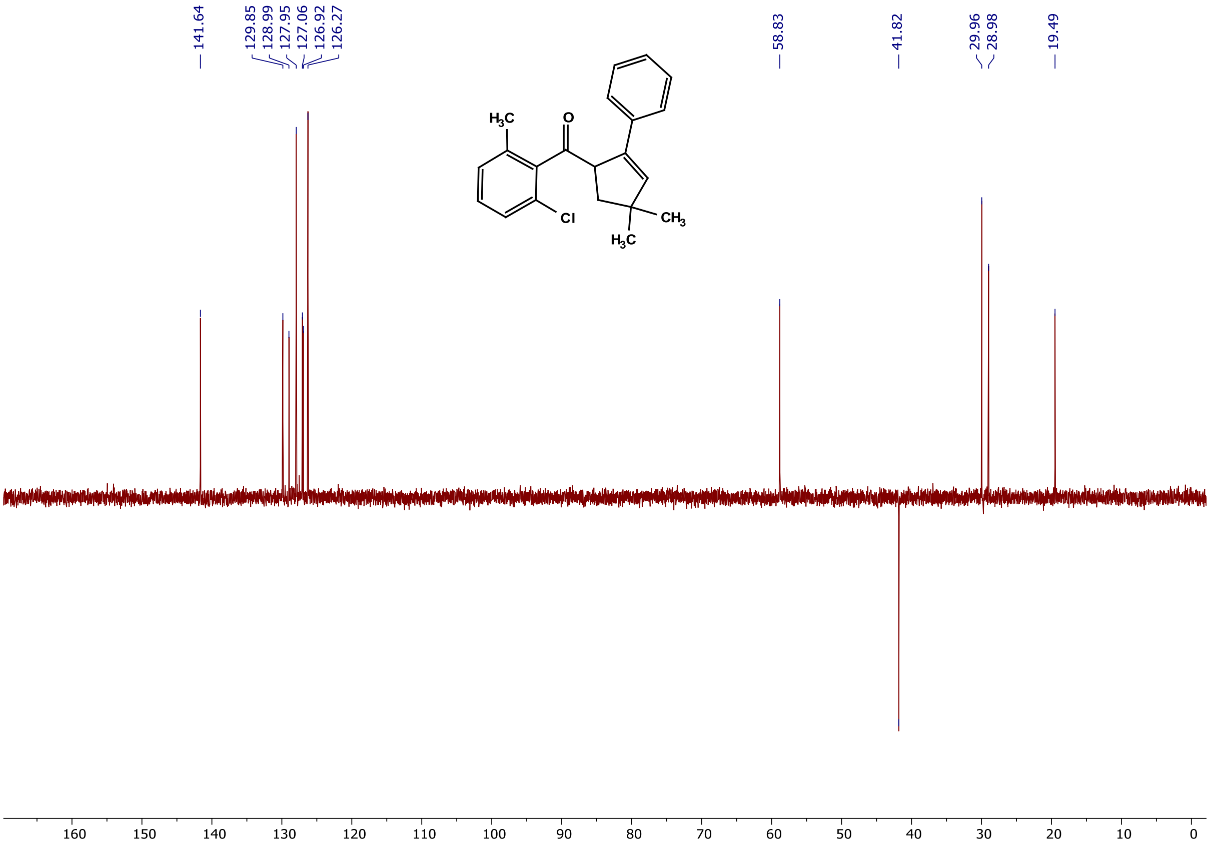

^{1}H NMR (400 MHz, Chloroform-*d*) (**3ap**):

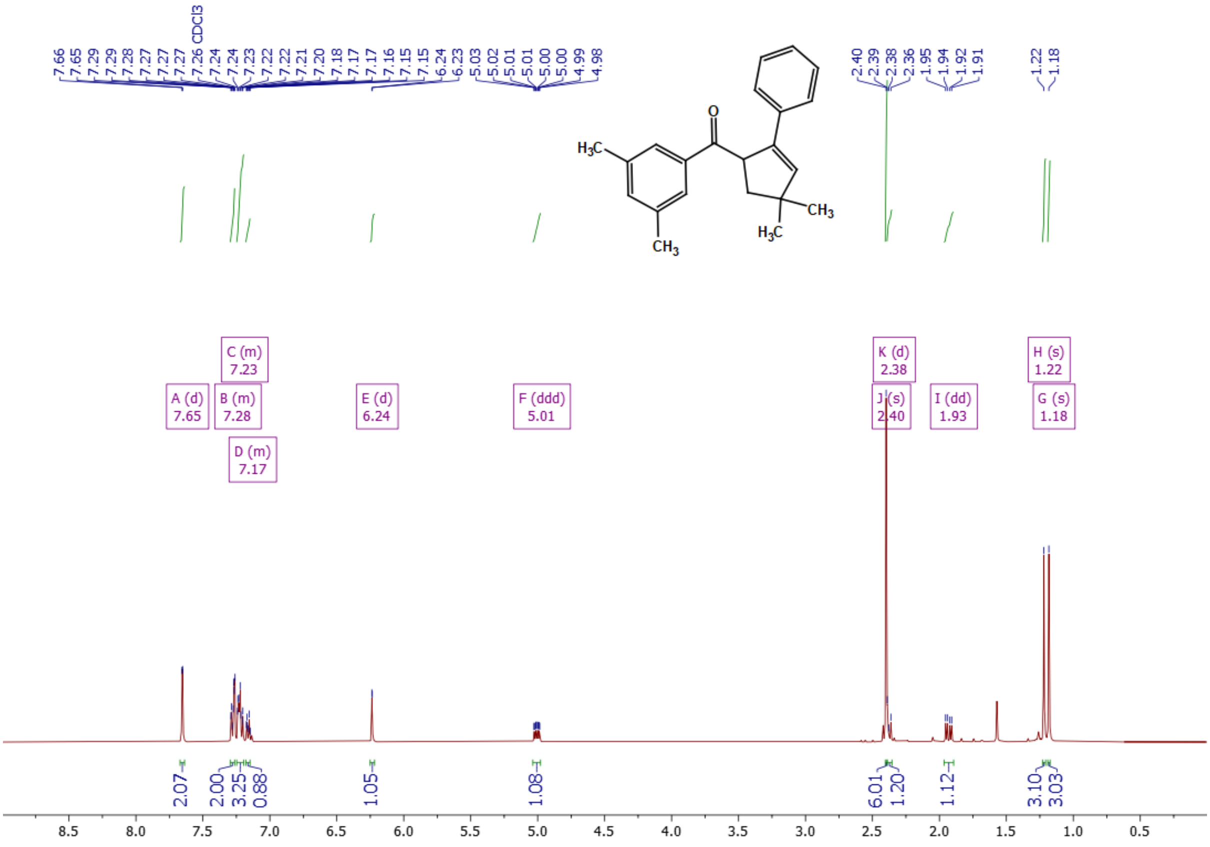

^{13}C NMR (101 MHz, Chloroform-*d*) (**3ap**):

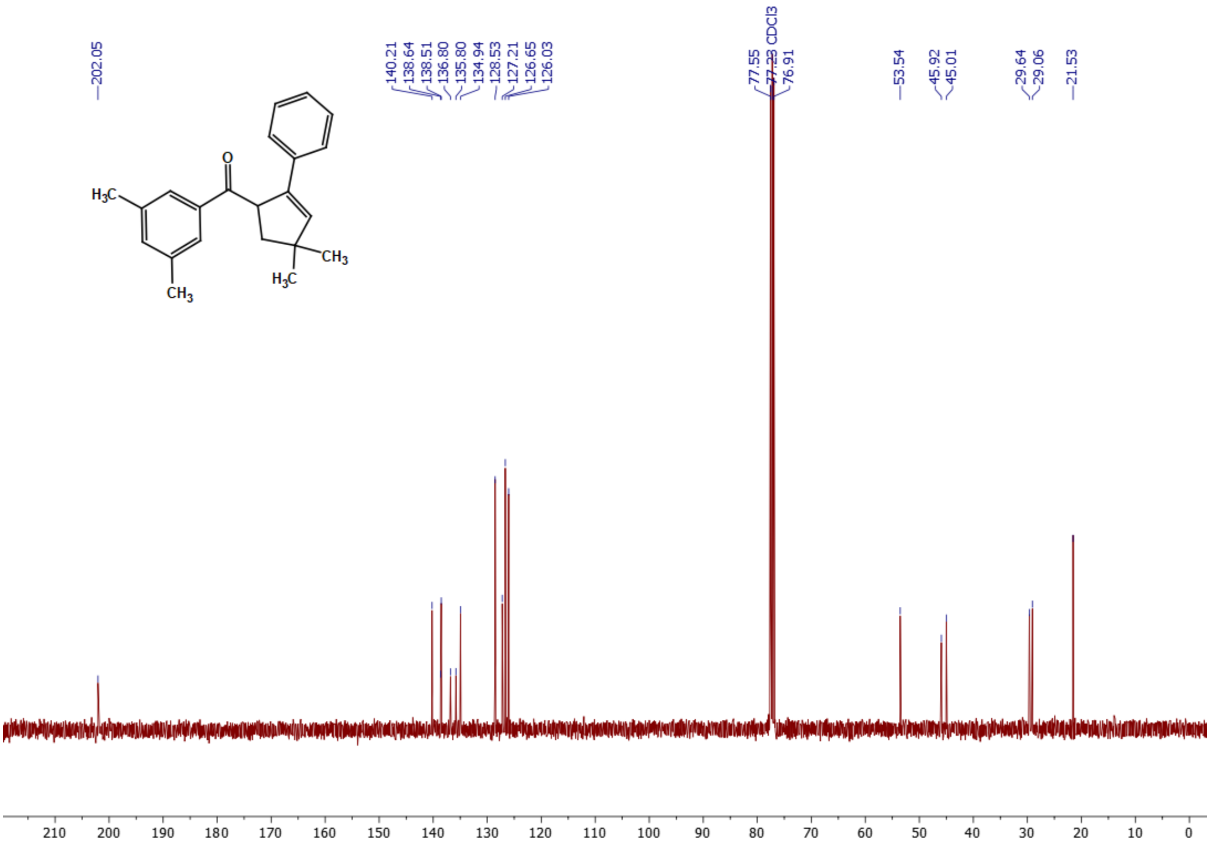

DEPT-135 NMR (101 MHz, Chloroform-*d*) (**3ap**):

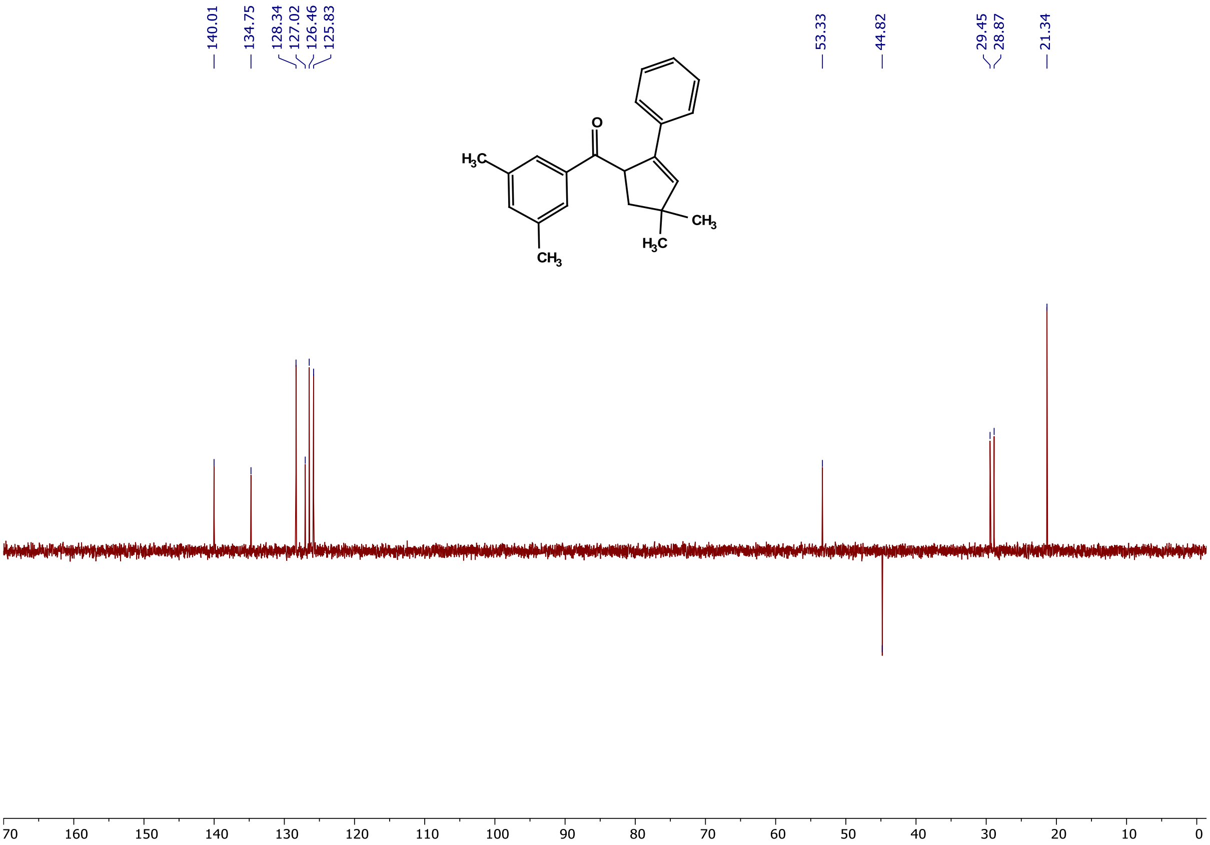

^{1}H NMR (400 MHz, Chloroform-*d*) (**3aq**):

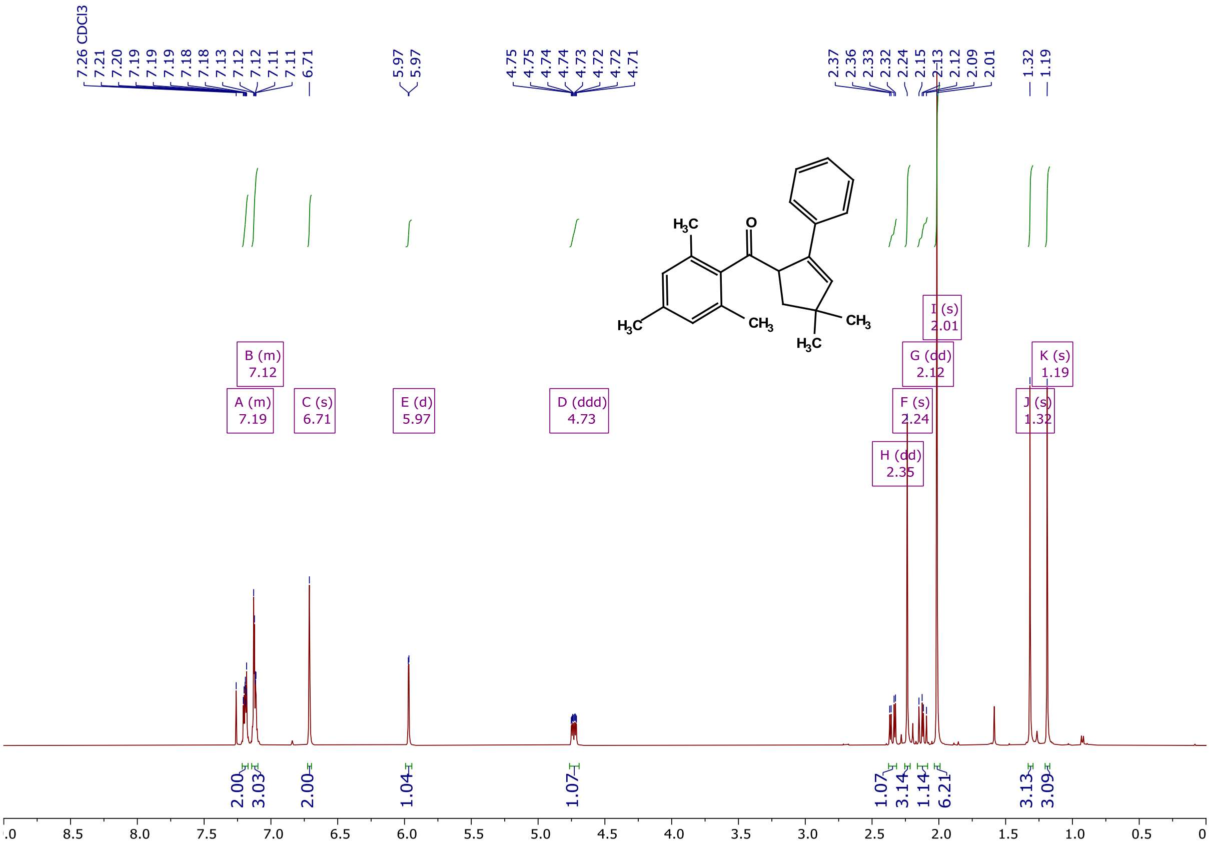

^{13}C NMR (101 MHz, Chloroform-*d*) (**3aq**):

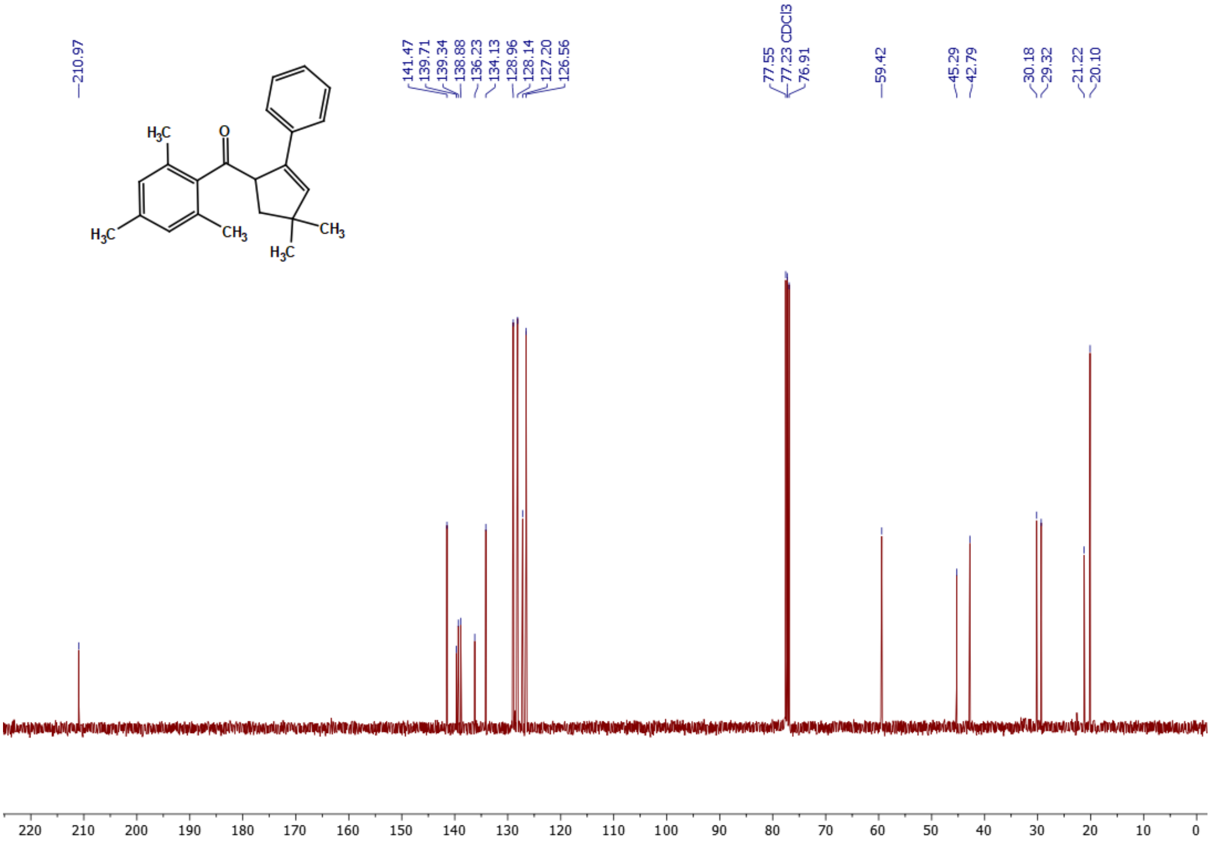

DEPT-135 NMR (101 MHz, Chloroform-*d*) (**3aq**):

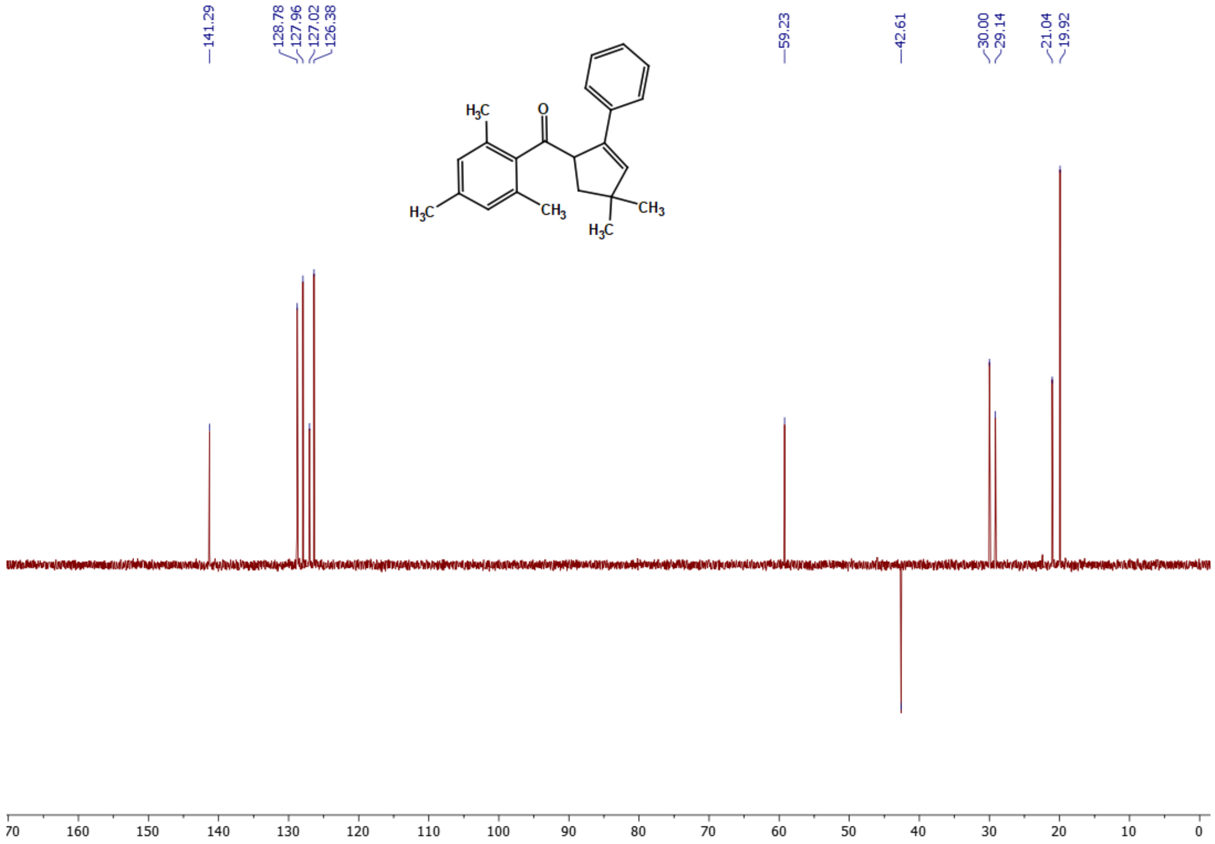

^{1}H NMR (400 MHz, Chloroform-*d*) (**3ar**):

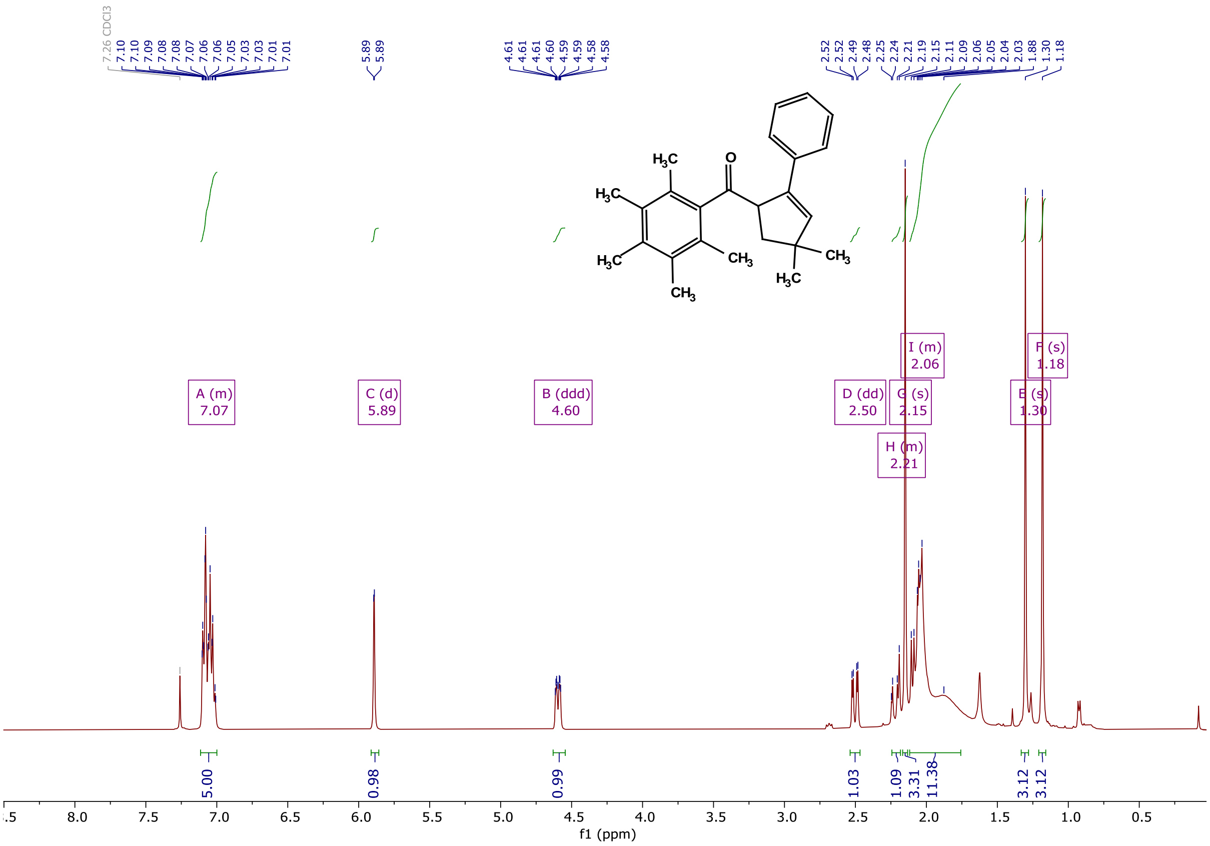

^{13}C NMR (101 MHz, Chloroform-*d*) (**3ar**):

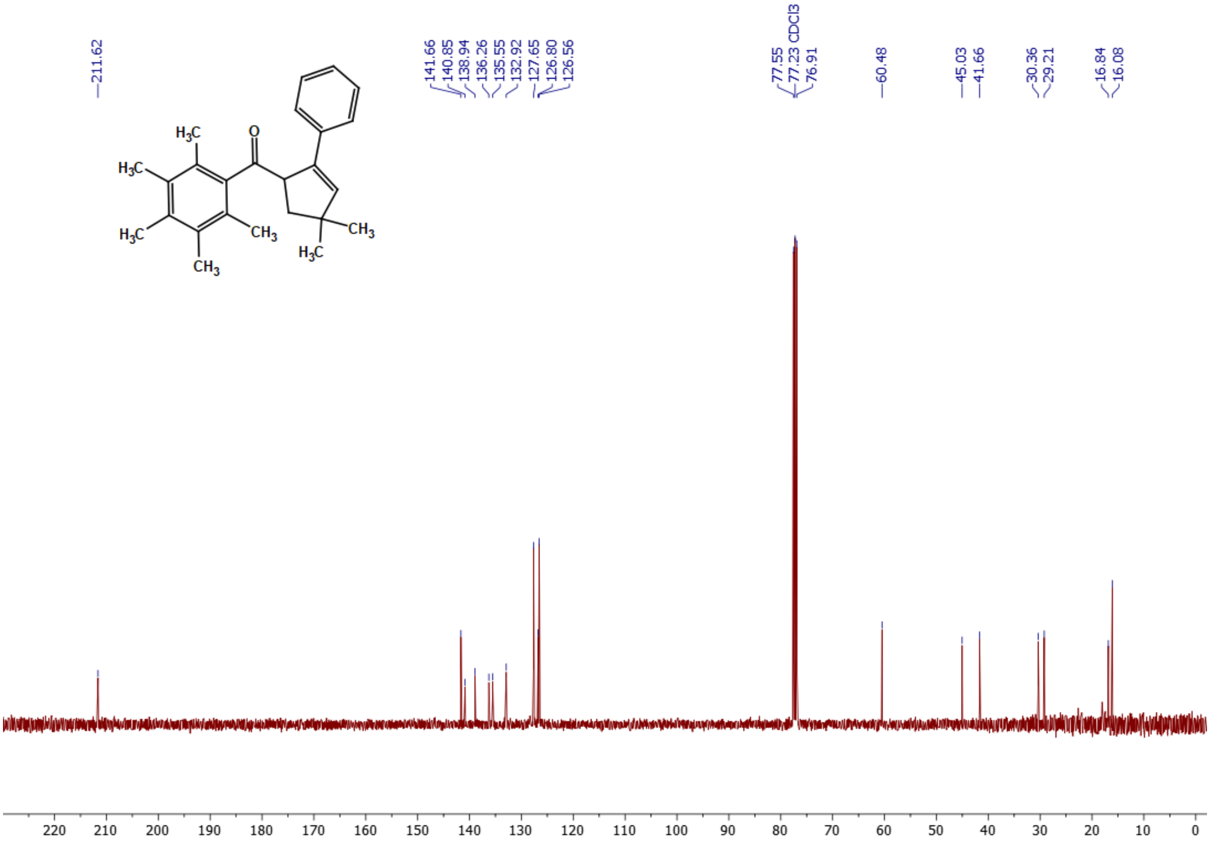

^{1}H NMR (400 MHz, Chloroform-*d*) (**3as**):

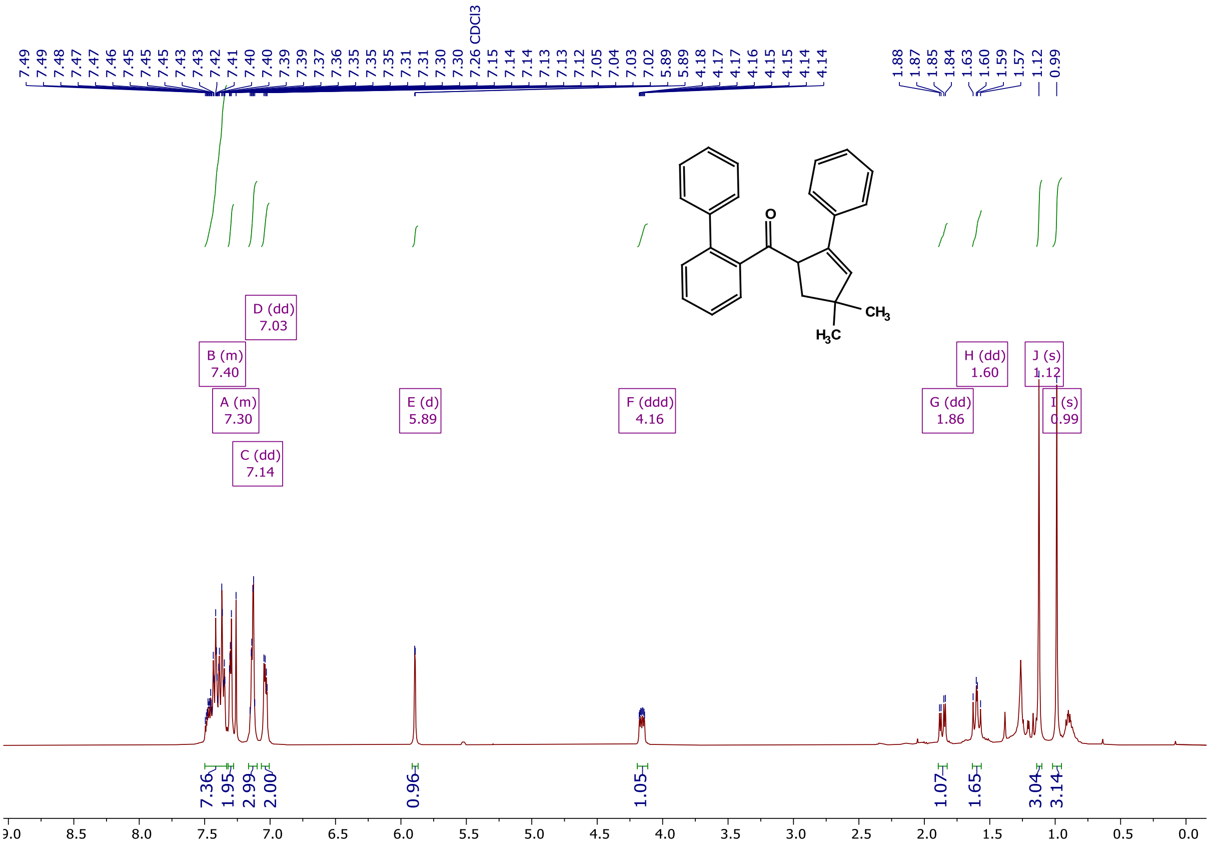

^{13}C NMR (101 MHz, Chloroform-*d*) (**3as**):

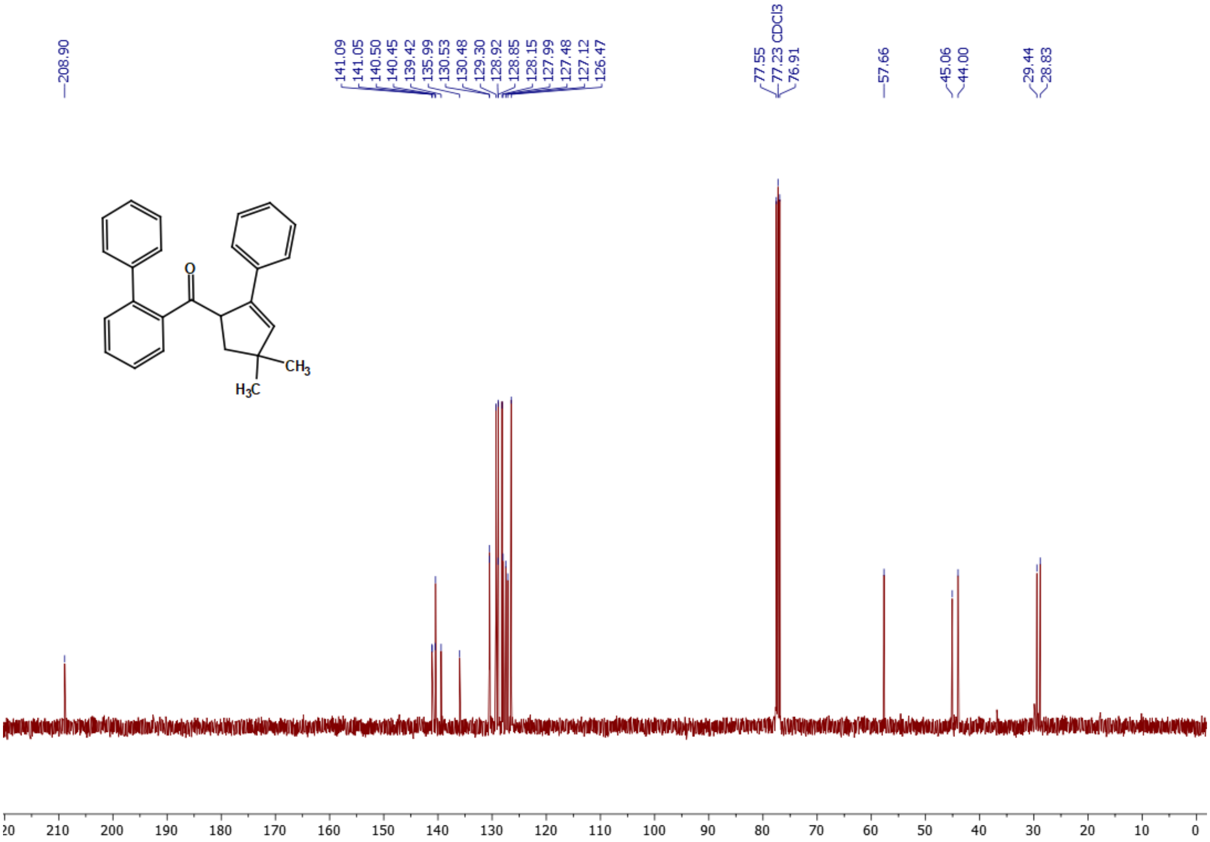

^{1}H NMR (400 MHz, Chloroform-*d*) (**3at**):

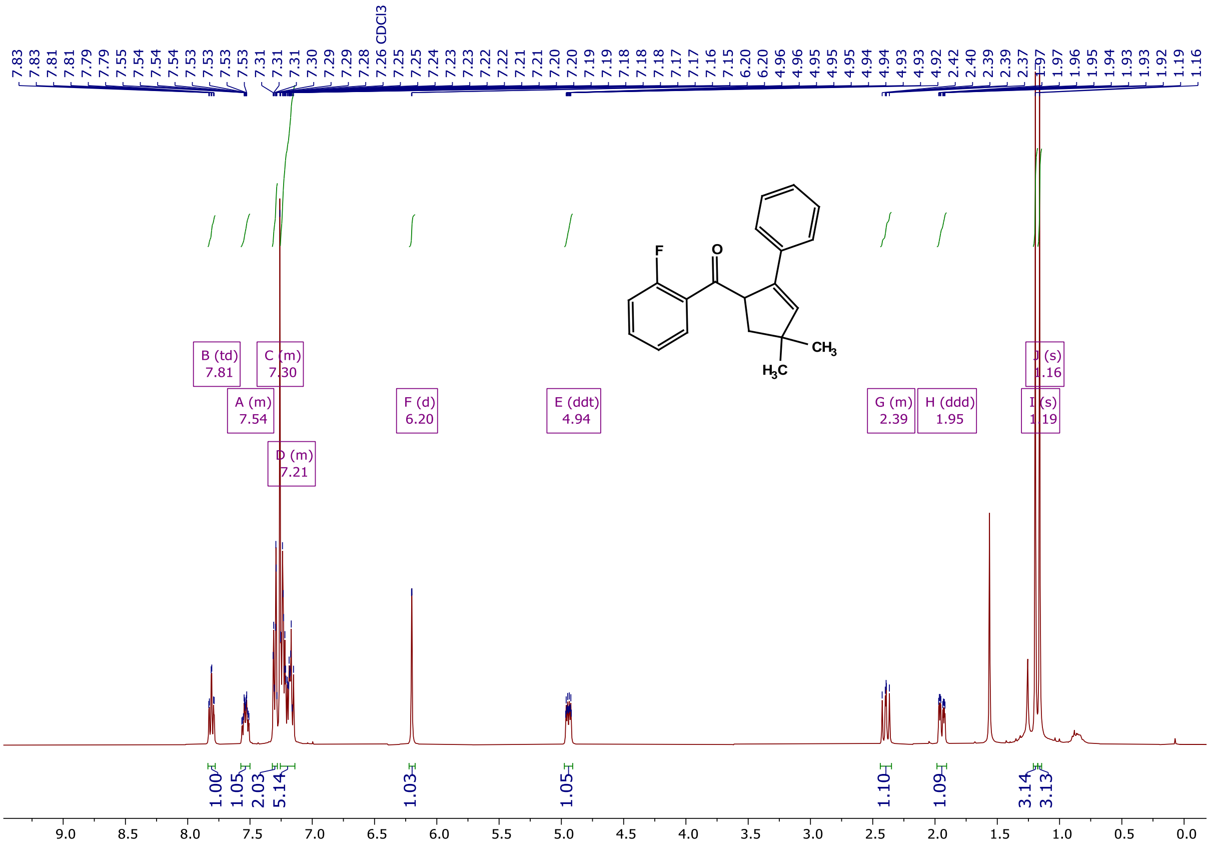

^{13}C NMR (101 MHz, Chloroform-*d*) (**3at**):

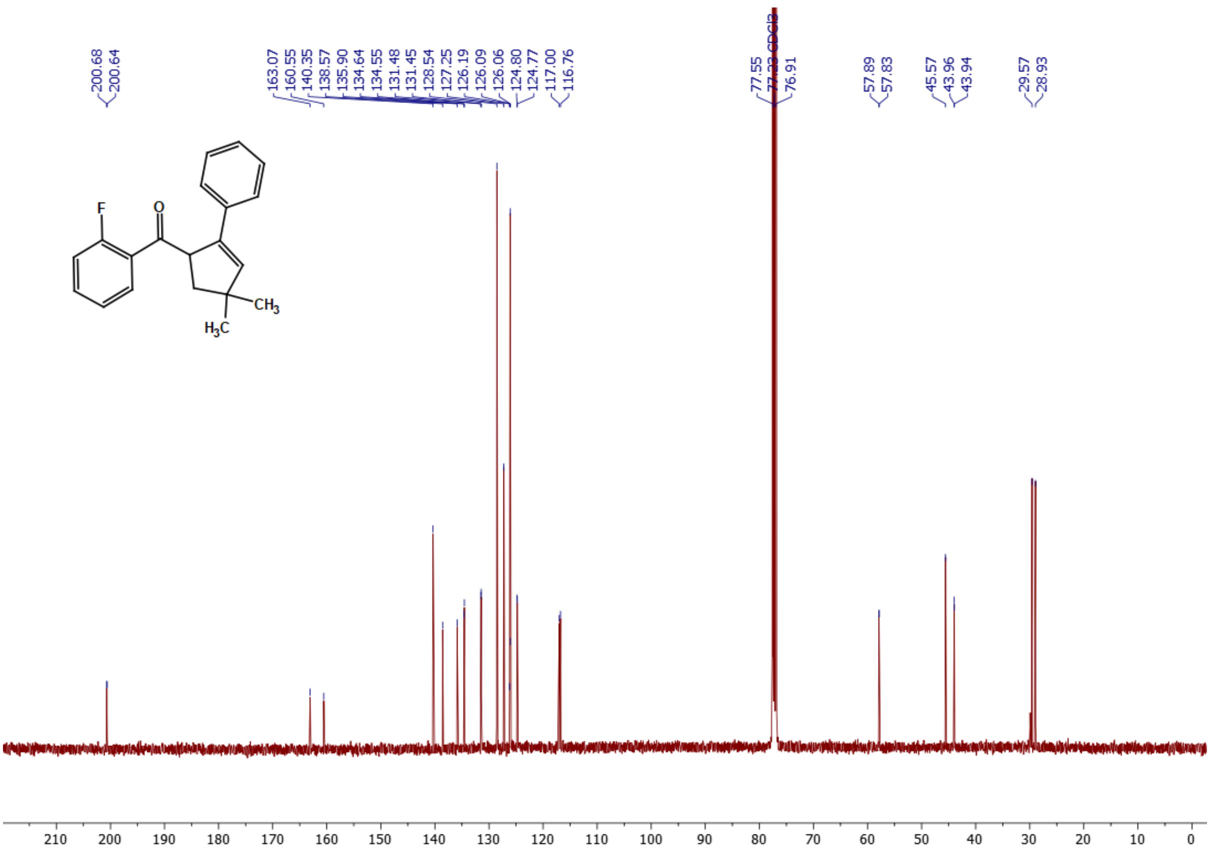

DEPT-135 NMR (101 MHz, Chloroform-*d*) (**3at**):

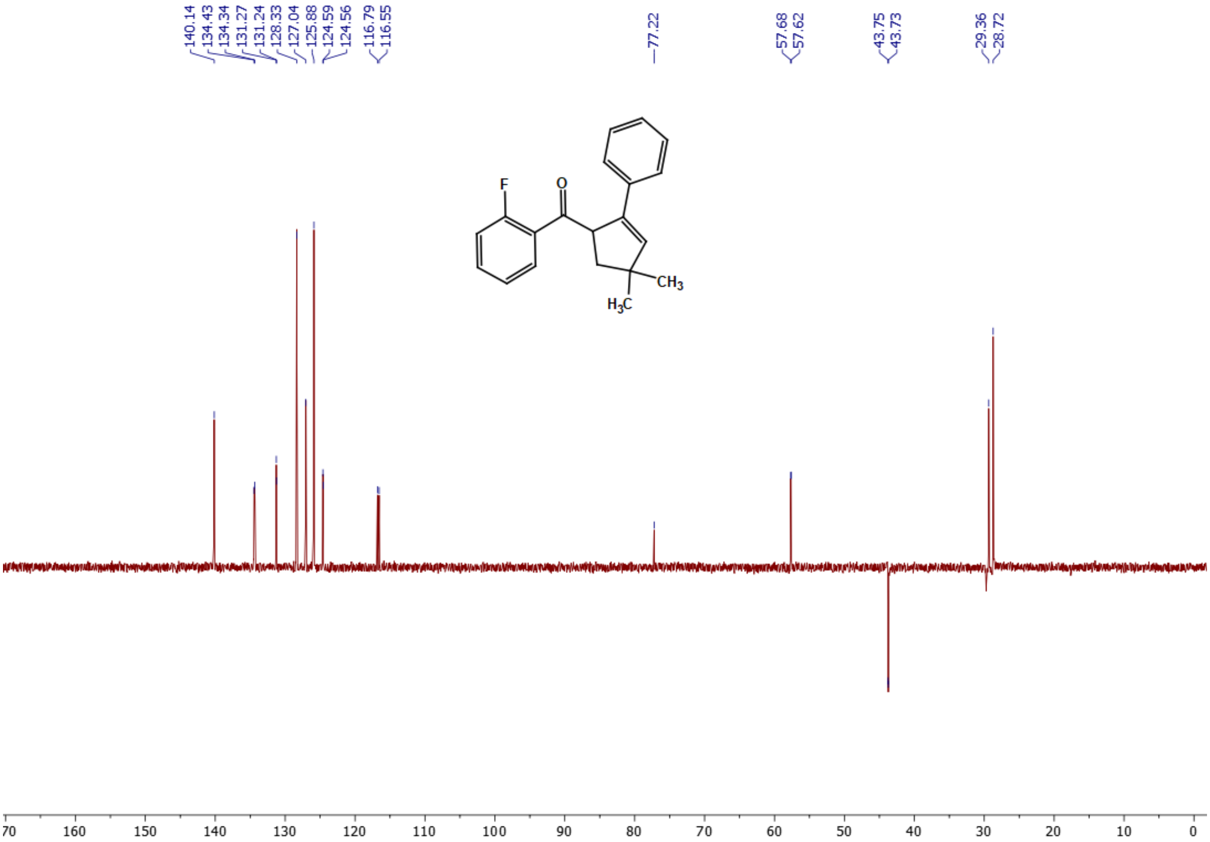

^{19}F NMR (376 MHz, Chloroform-*d*) (**3at**):

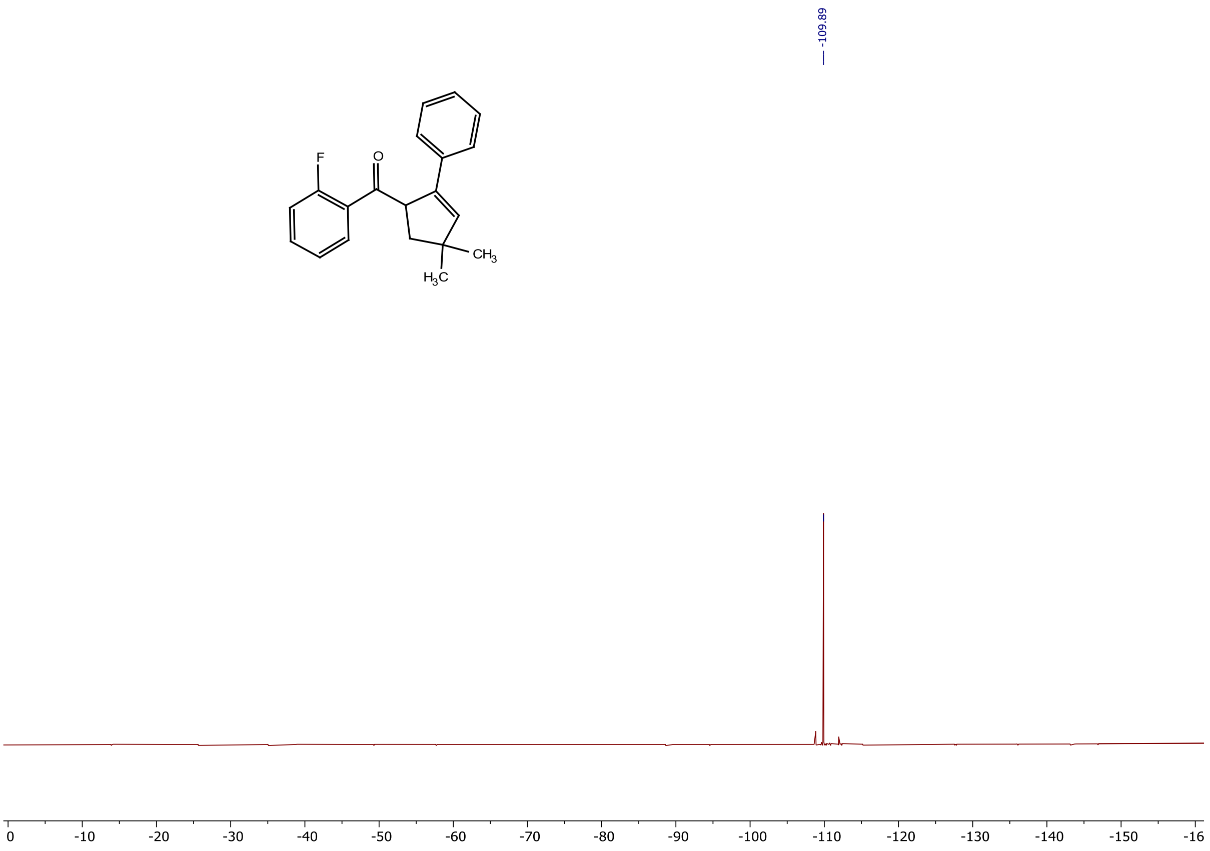

^{1}H NMR (400 MHz, Chloroform-*d*) (**3au**):

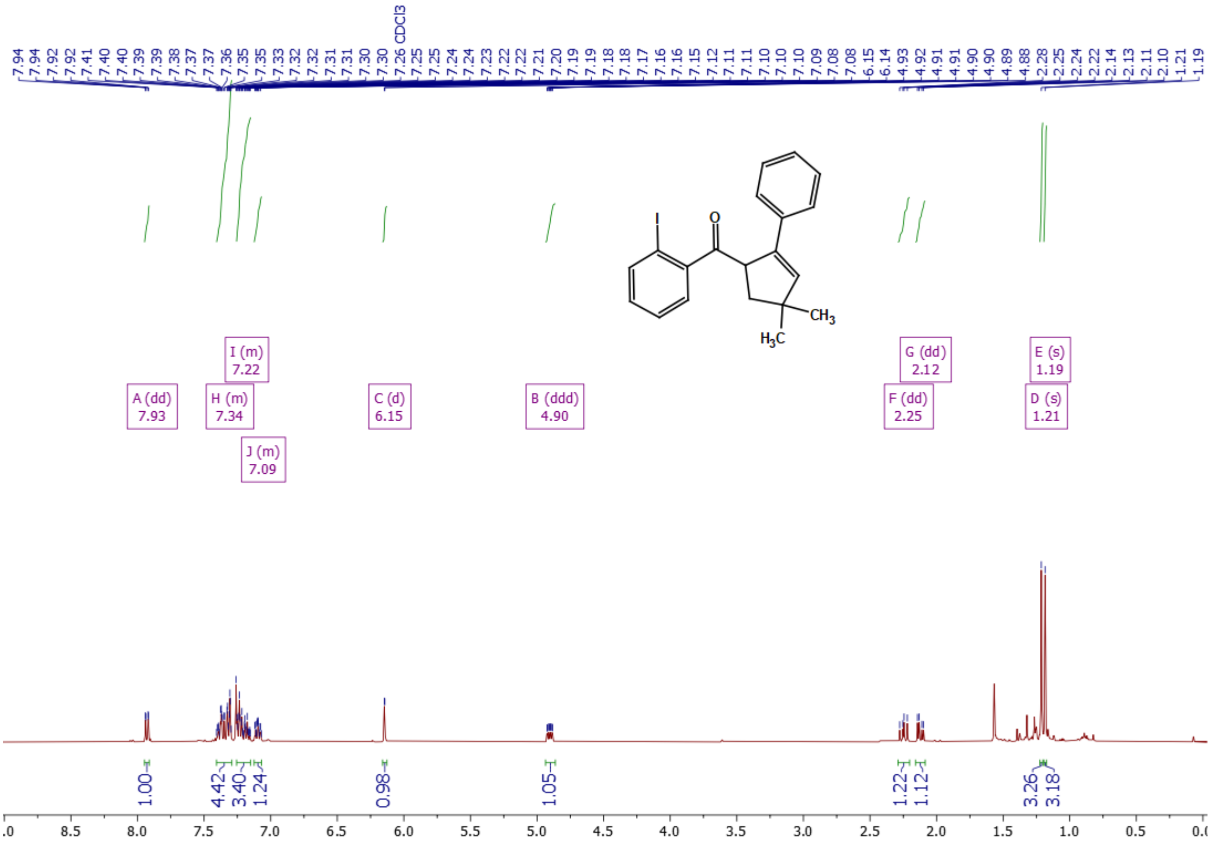

^{13}C NMR (101 MHz, Chloroform-*d*) (**3au**):

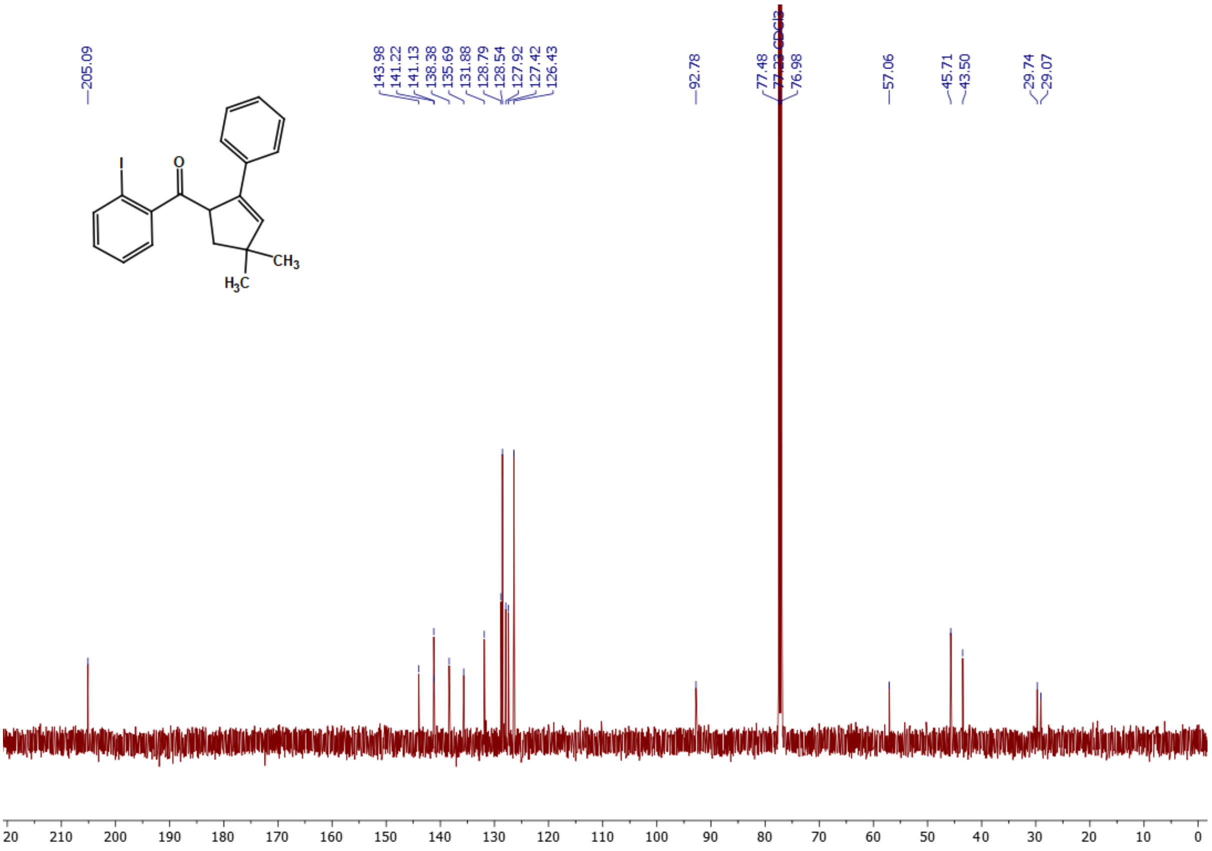

DEPT-135 NMR (101 MHz, Chloroform-*d*) (**3au**):

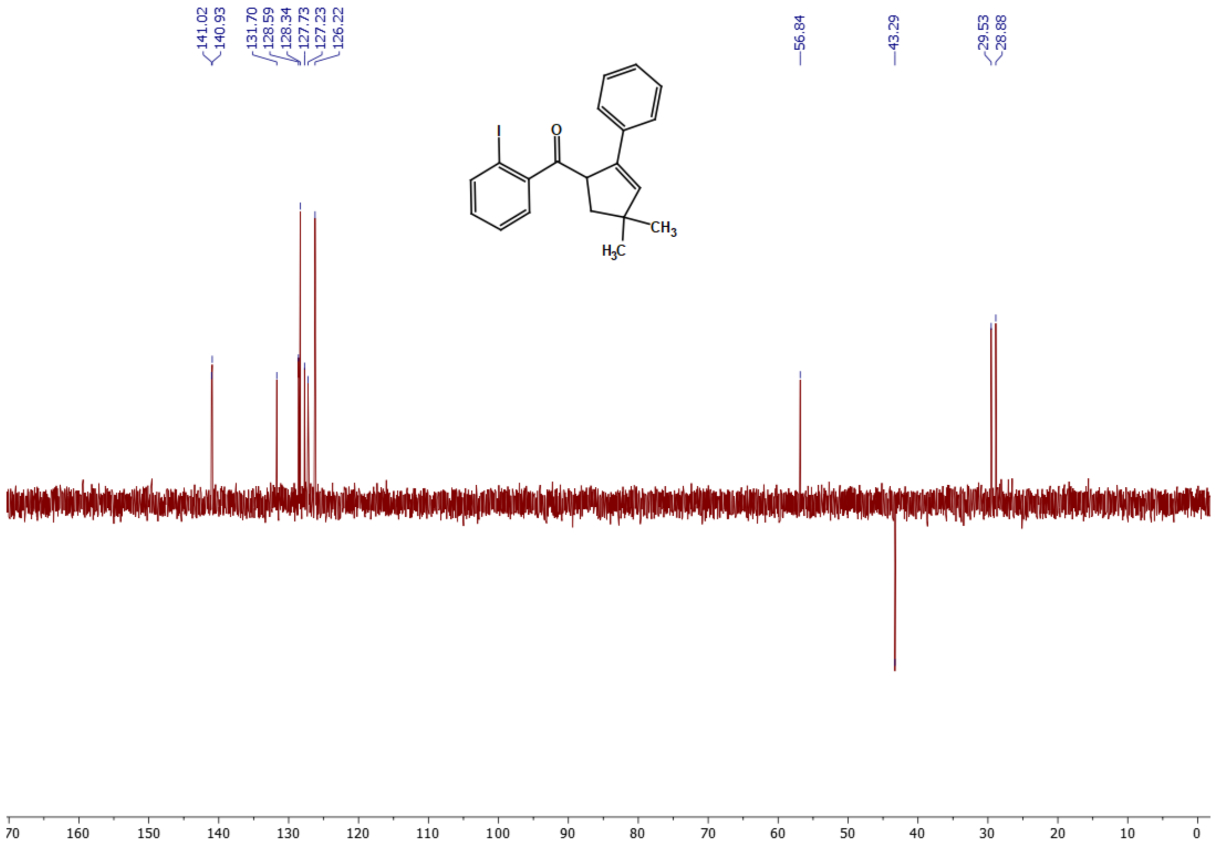

^{1}H NMR (500 MHz, Chloroform-*d*) (**3av**):

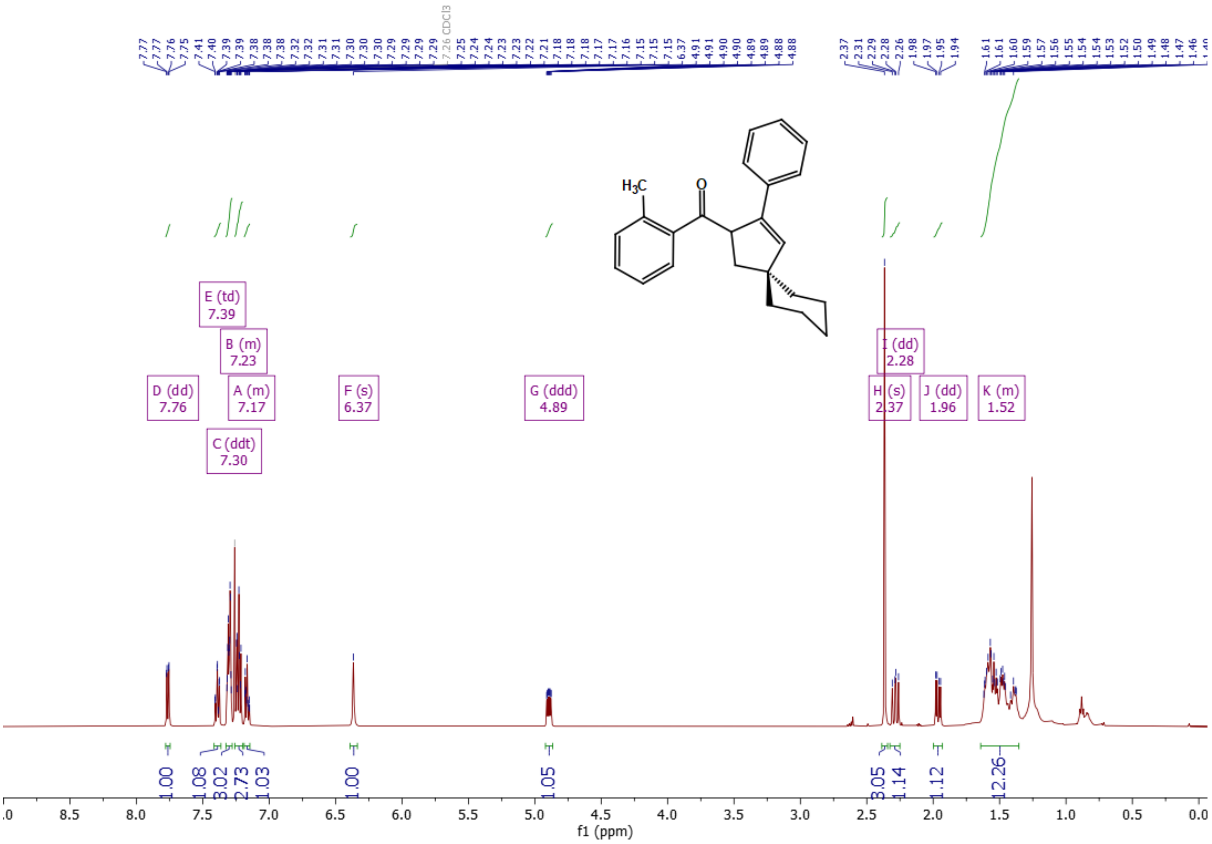

^{13}C NMR (126 MHz, Chloroform-*d*) (**3av**):

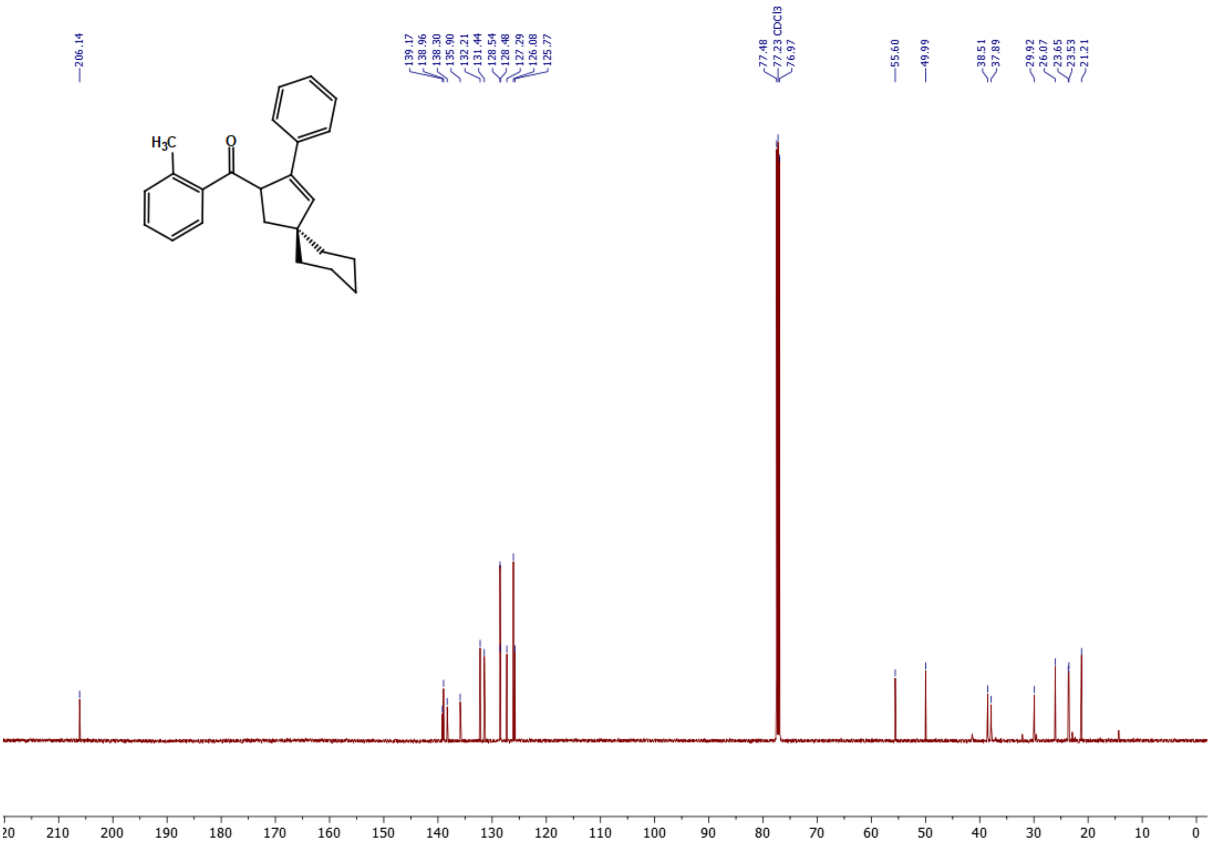

DEPT-135 NMR (126 MHz, Chloroform-*d*) (**3av**):

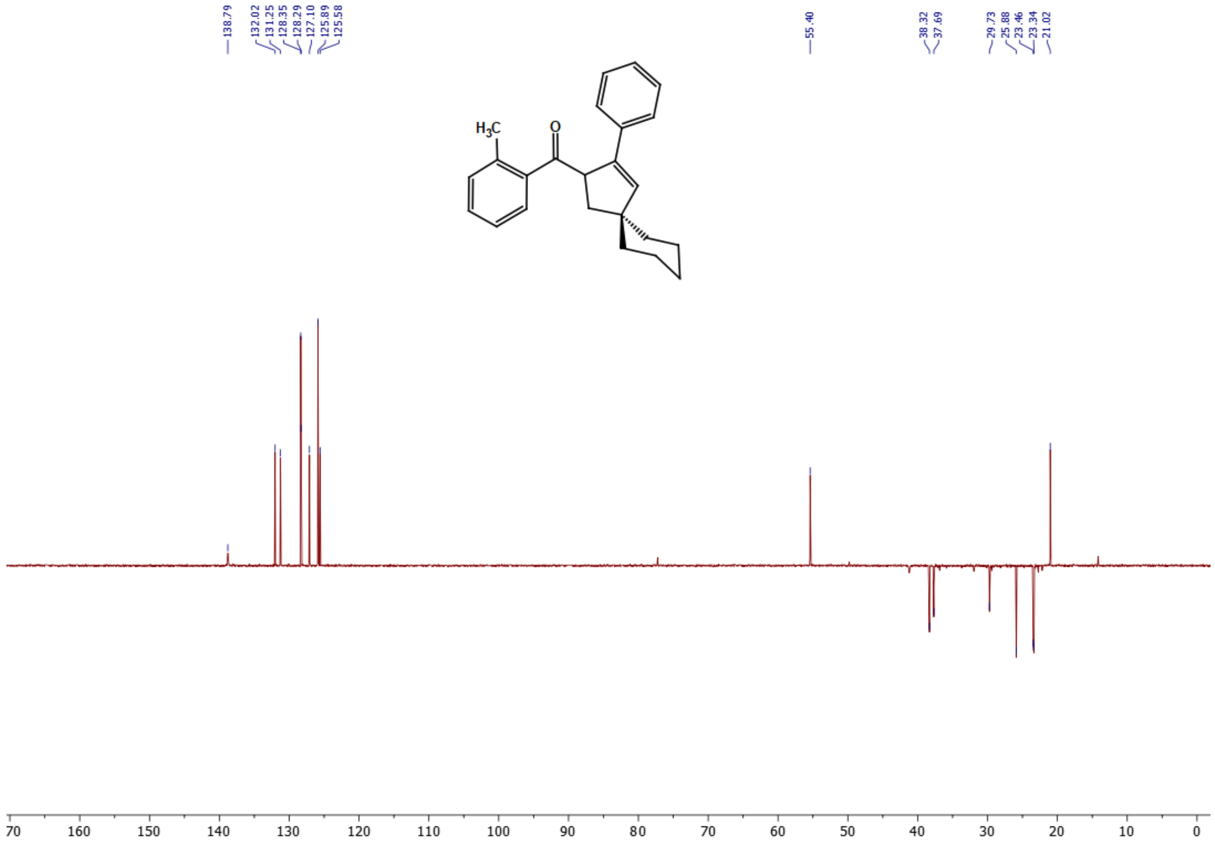

^{1}H NMR (400 MHz, Chloroform-*d*) (**3aw**):

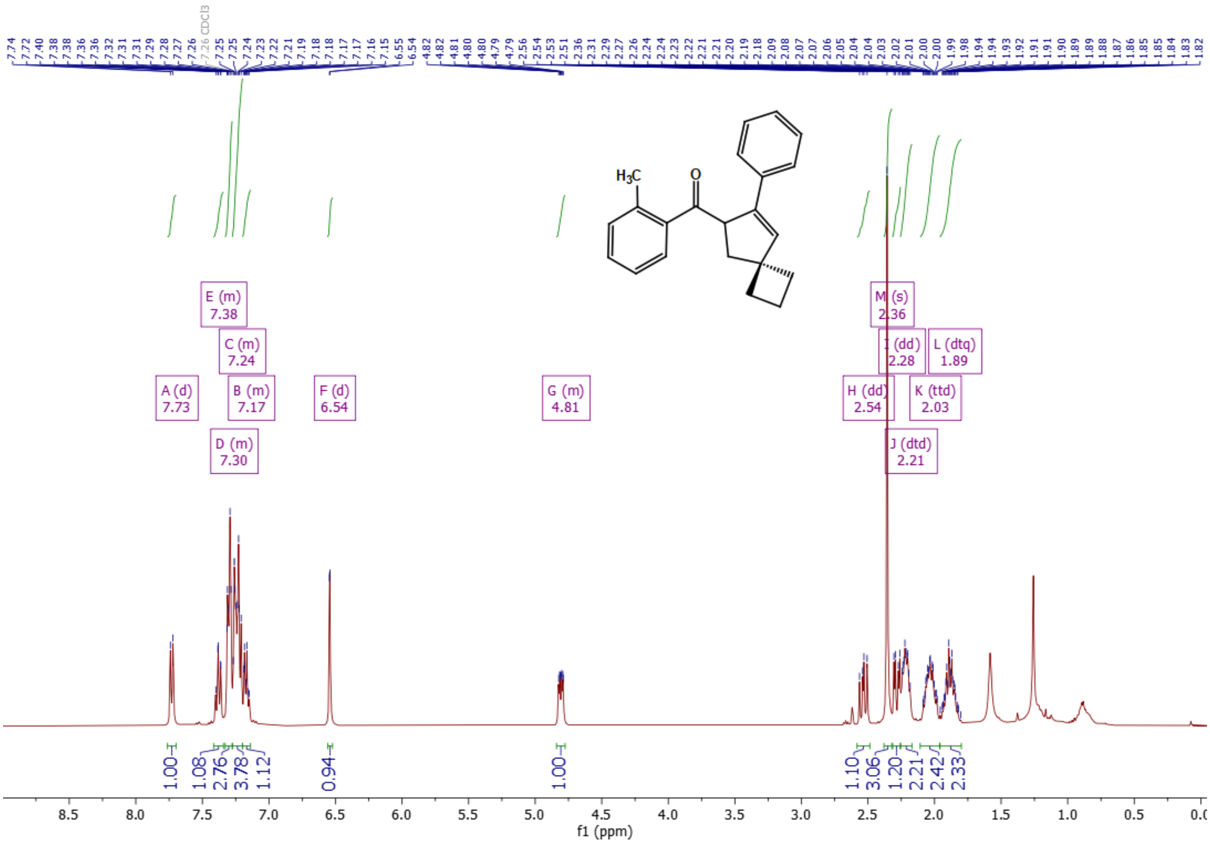

^{13}C NMR (101 MHz, Chloroform-*d*) (**3aw**):

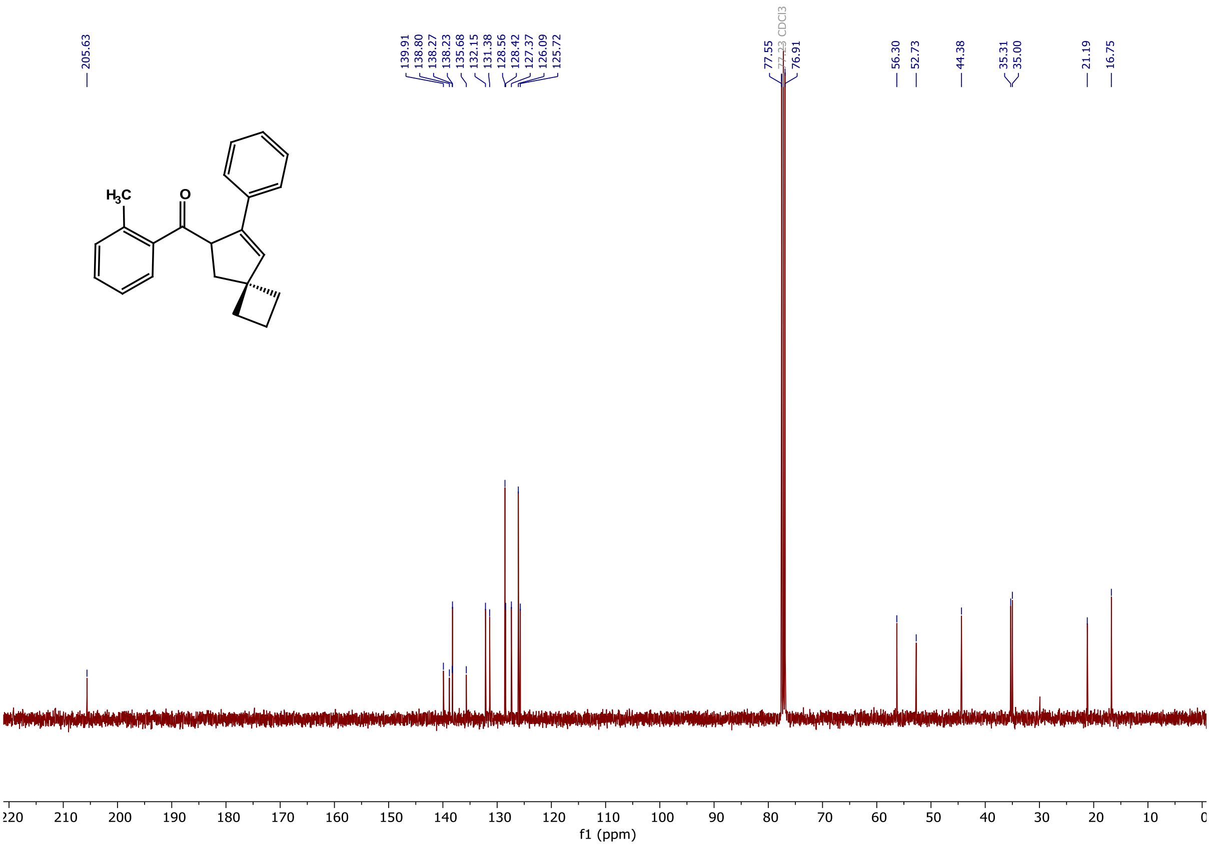

DEPT-135 NMR (101 MHz, Chloroform-*d*) (**3aw**):

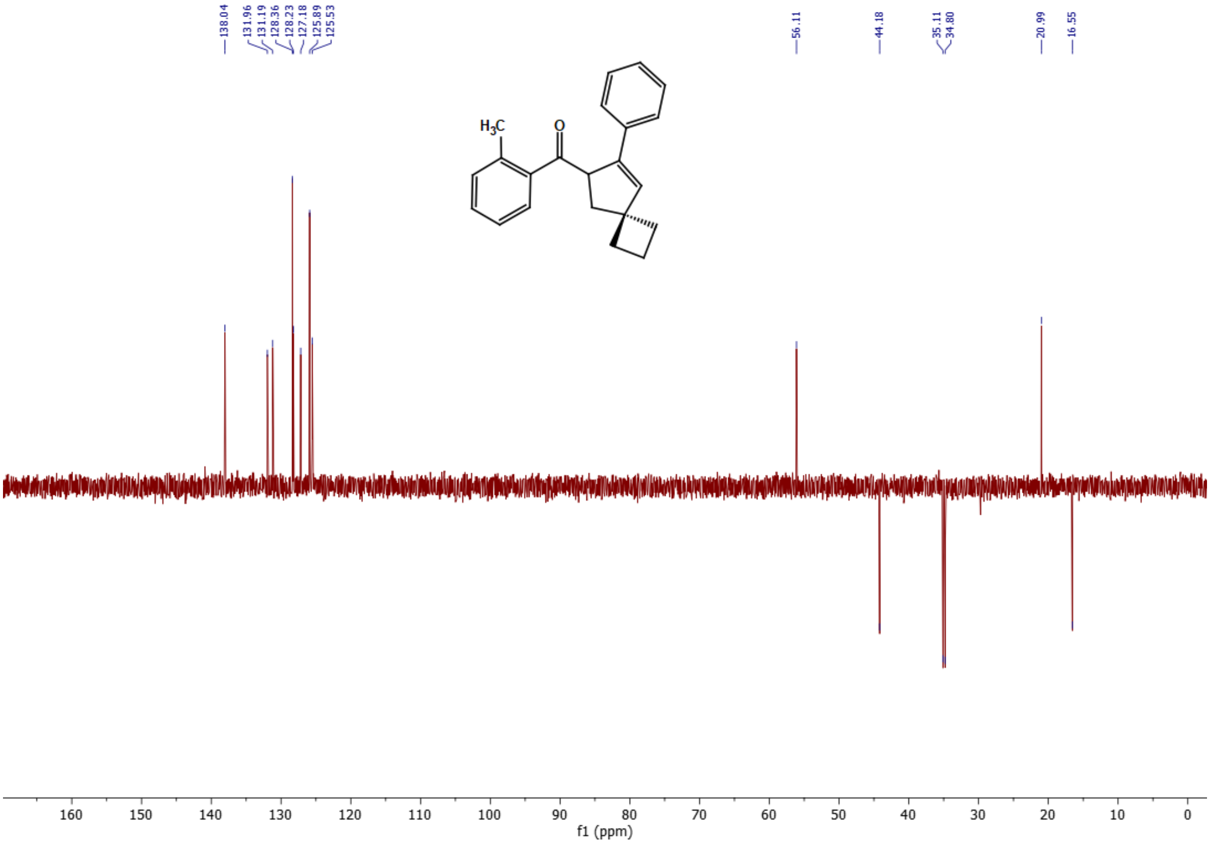

^{1}H NMR (400 MHz, Chloroform-*d*) (**3ax**):

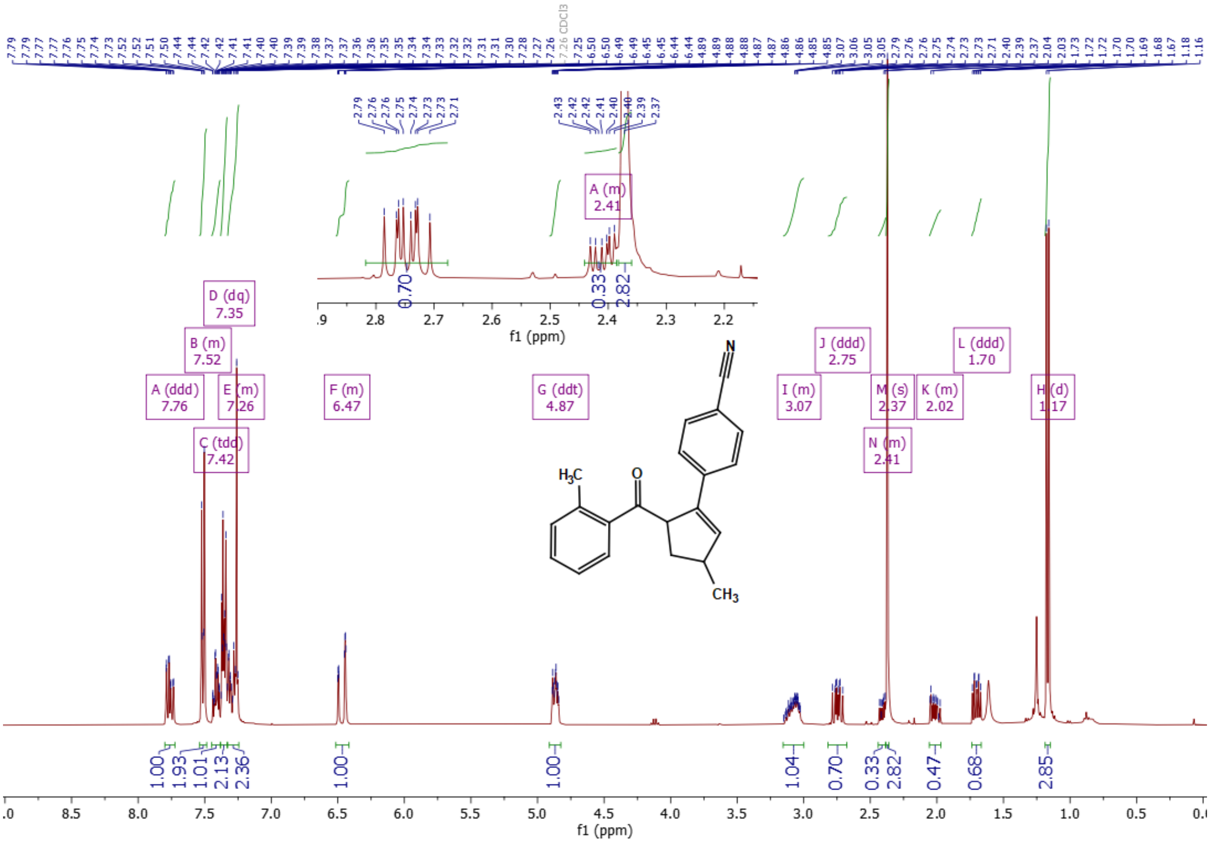

^{13}C NMR (101 MHz, Chloroform-*d*) (**3ax**):

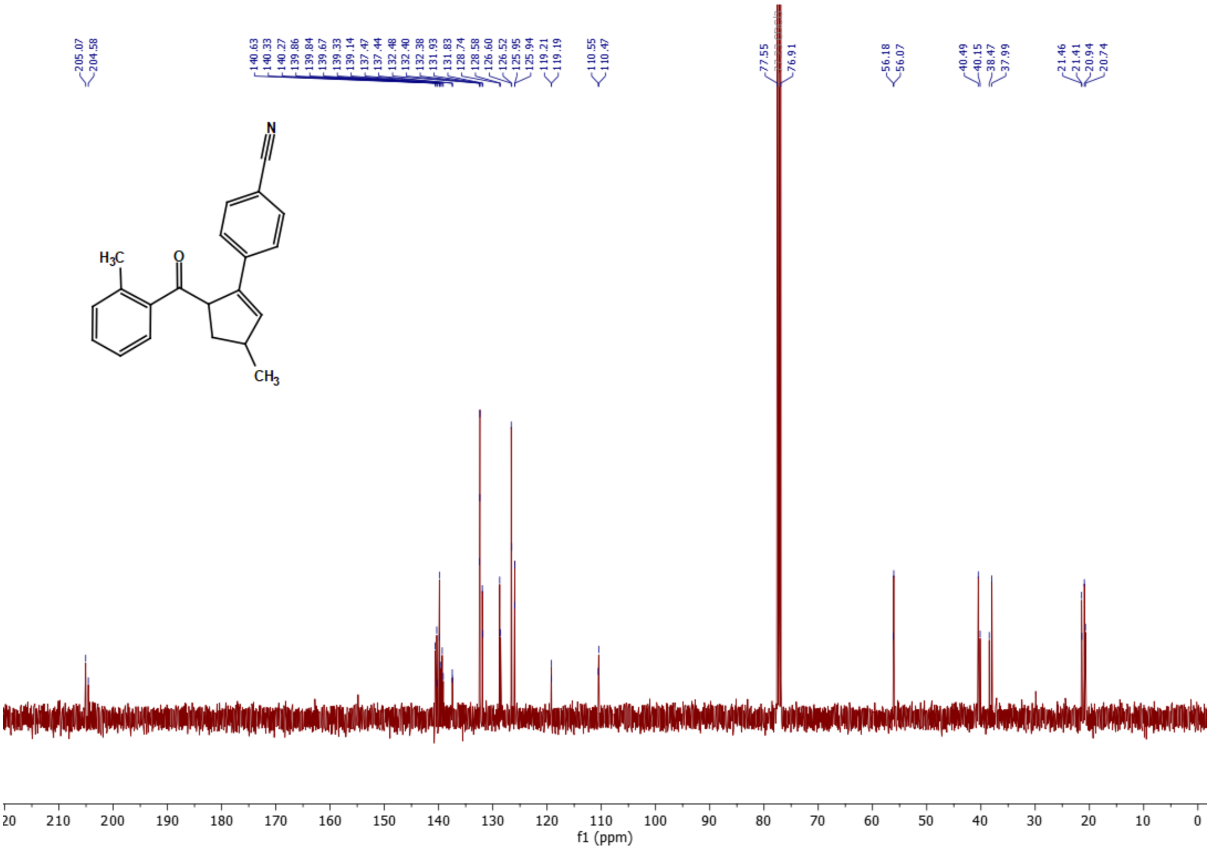

DEPT-135 NMR (101 MHz, Chloroform-*d*) (**3ax**):

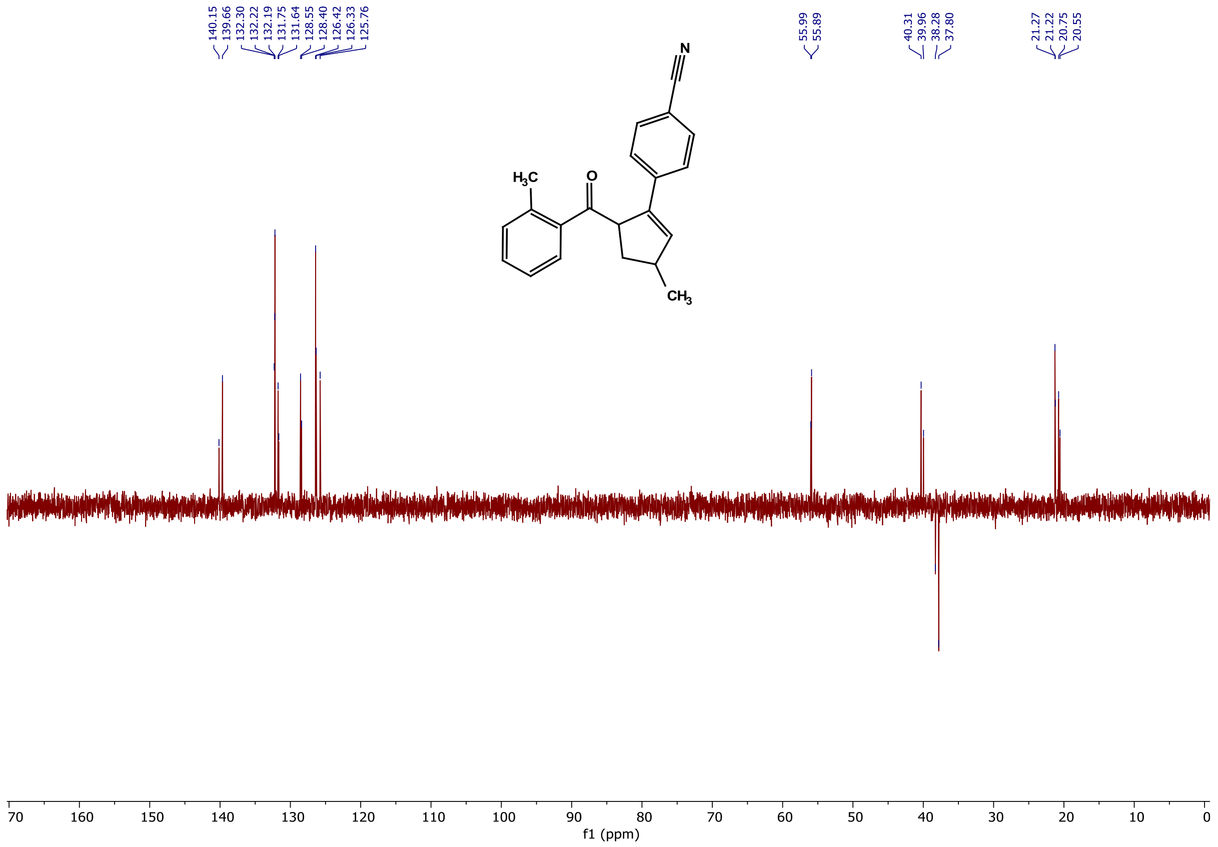

^{1}H NMR (400 MHz, Chloroform-*d*) (**3aab**):

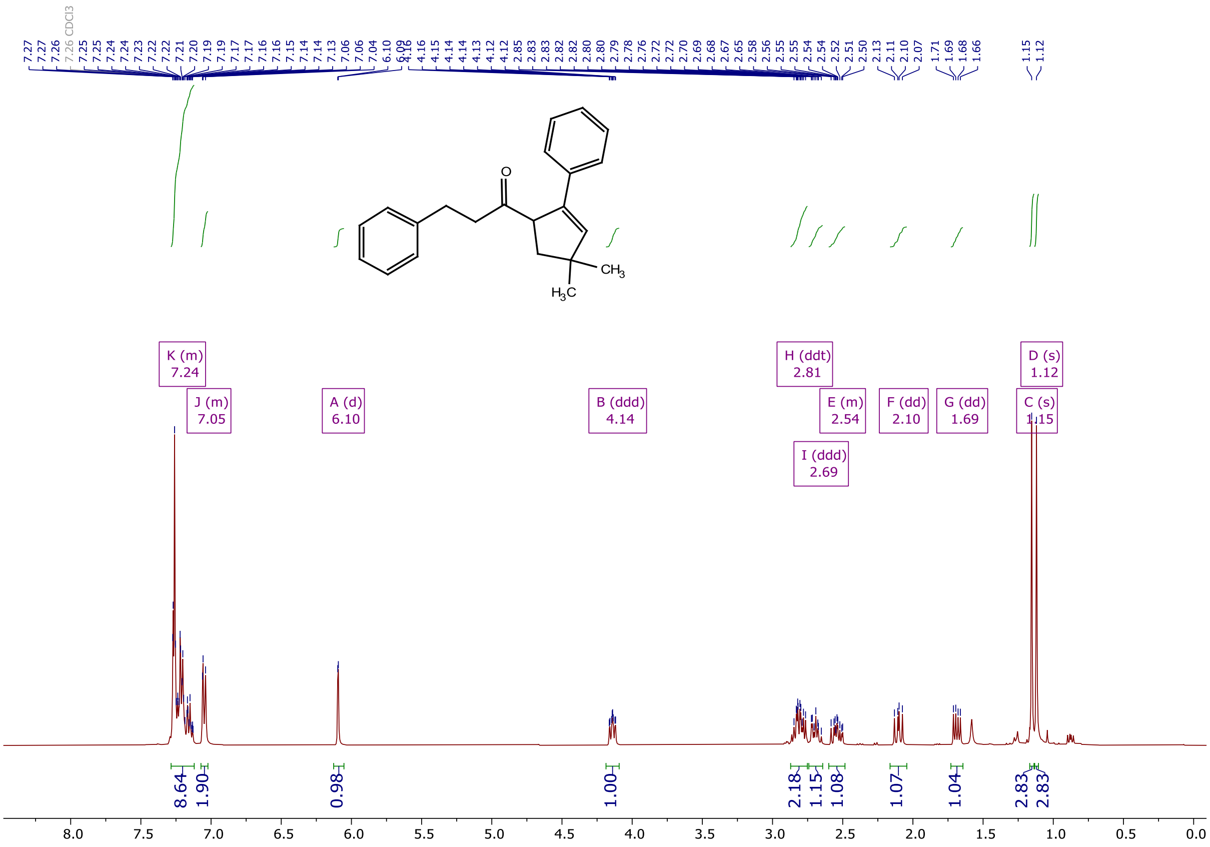

^{13}C NMR (101 MHz, Chloroform-*d*) (**3aab**):

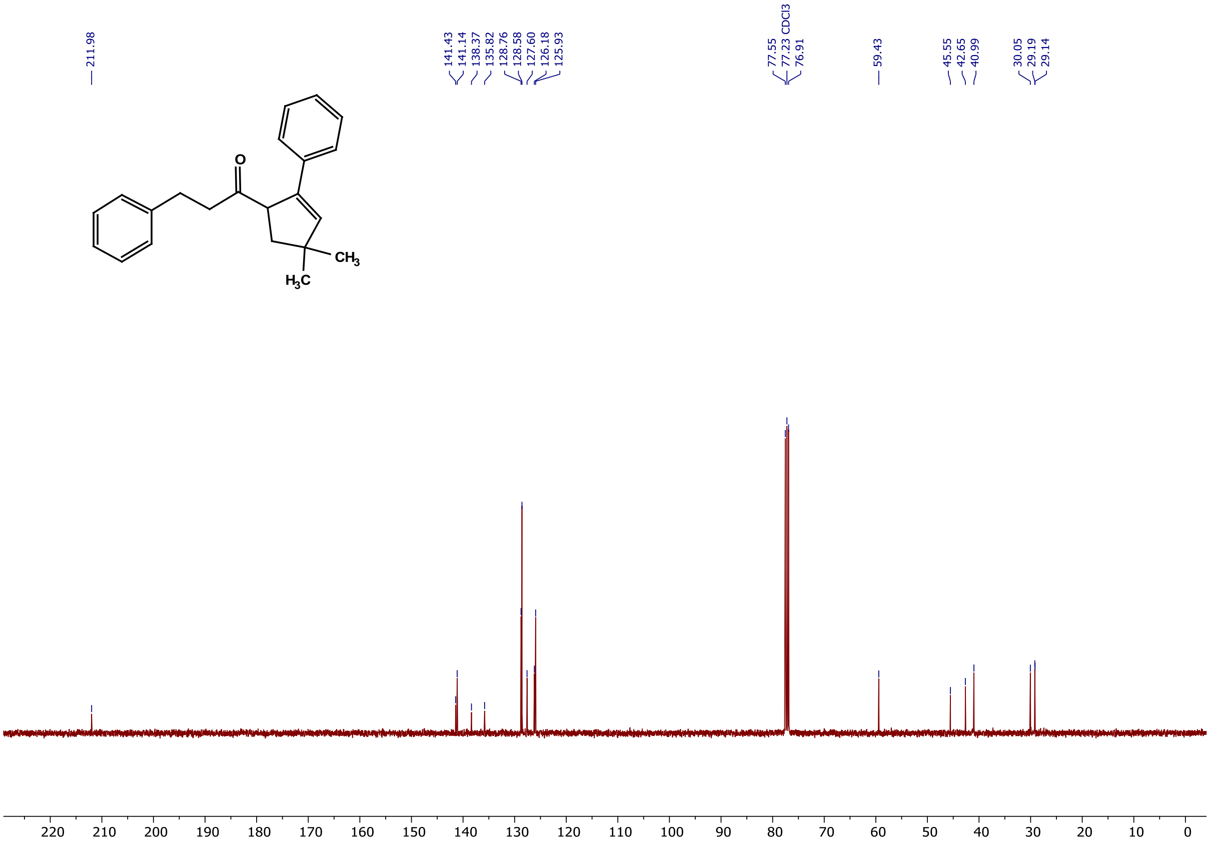

DEPT-135 NMR (101 MHz, Chloroform-*d*) (**3aab**):

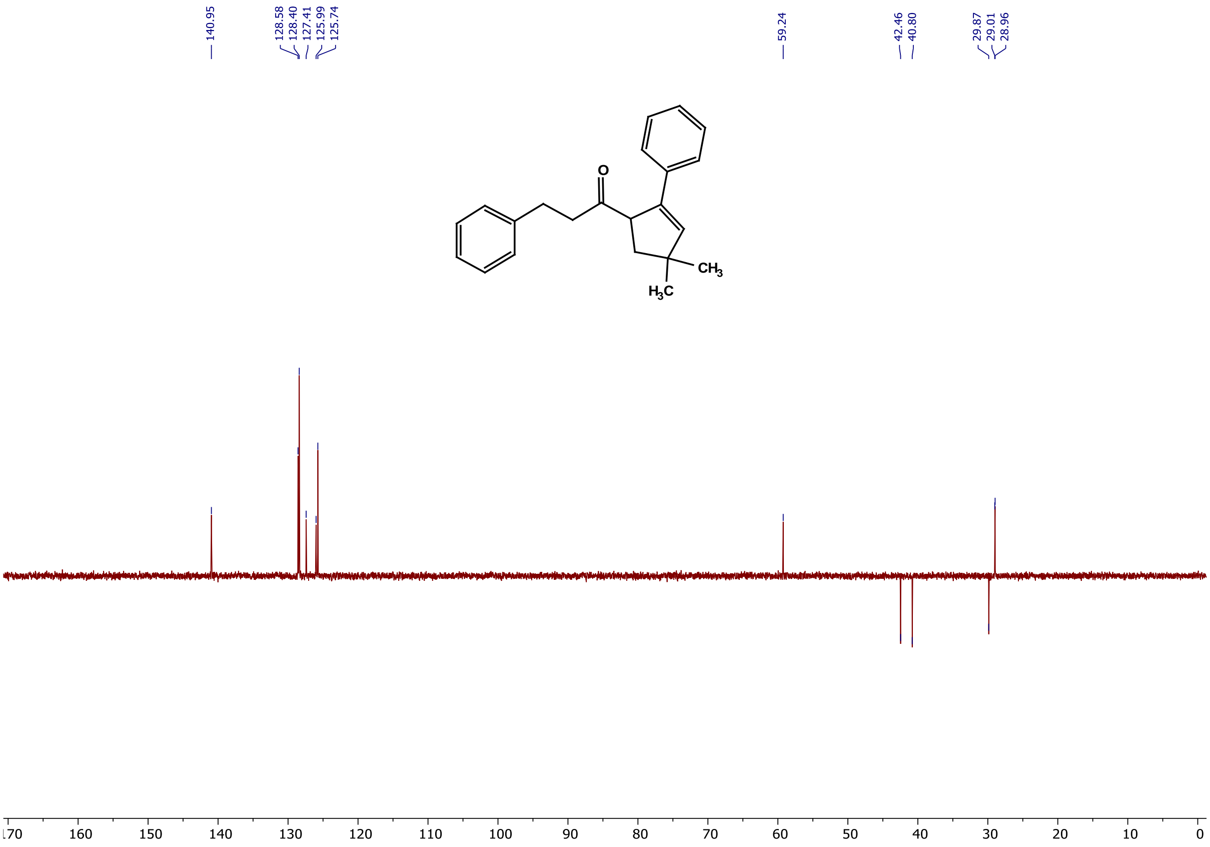

^{1}H NMR (400 MHz, Chloroform-*d*) (**3aac**):

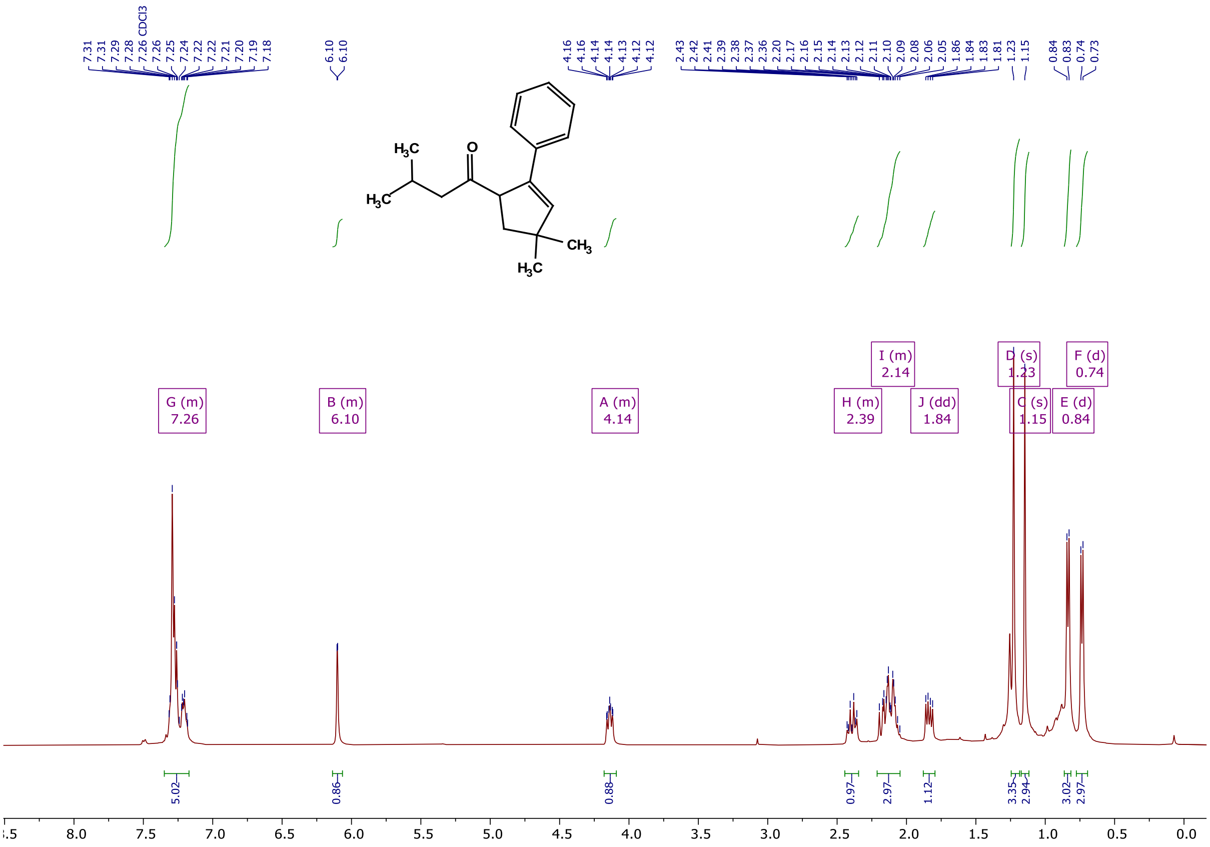

^{13}C NMR (101 MHz, Chloroform-*d*) (**3aac**):

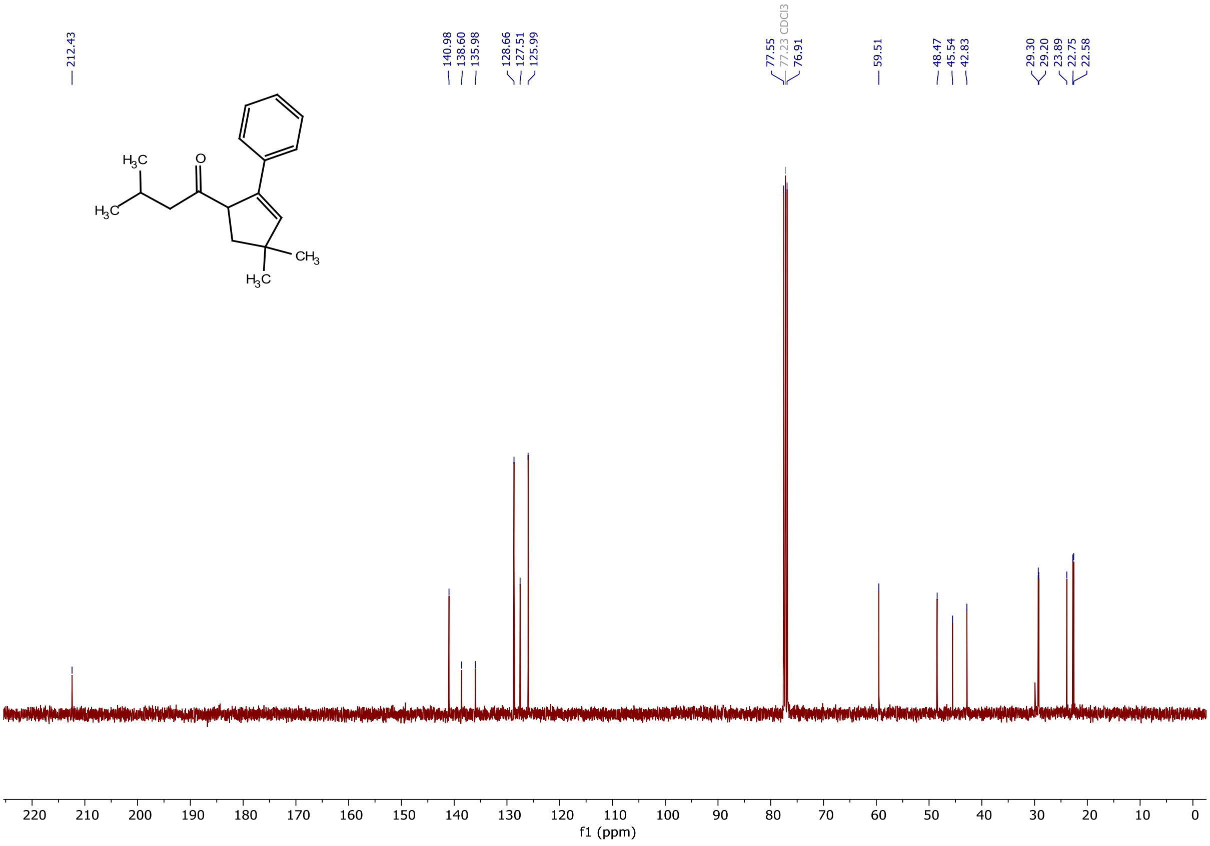

DEPT-135 NMR (101 MHz, Chloroform-*d*) (**3aac**):

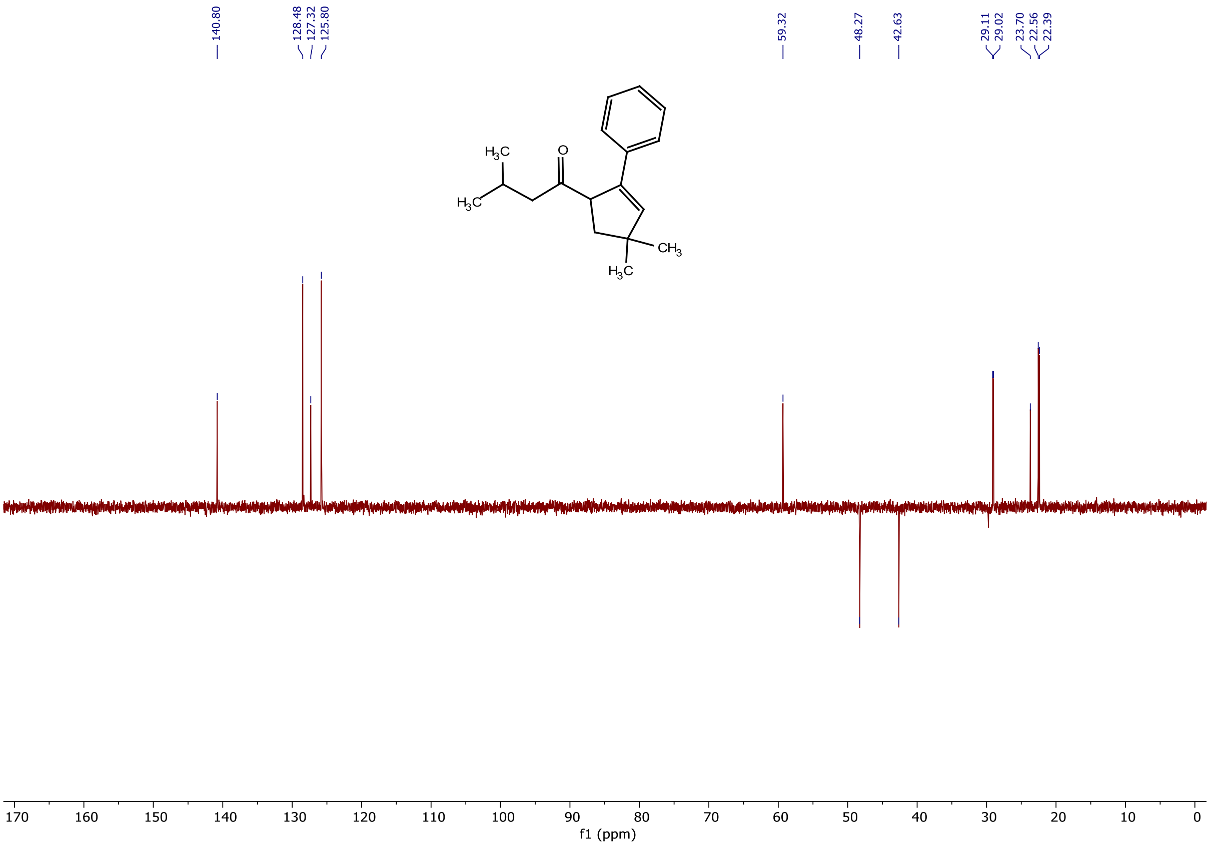

^{1}H NMR (400 MHz, Chloroform-*d*) (**3aad**):

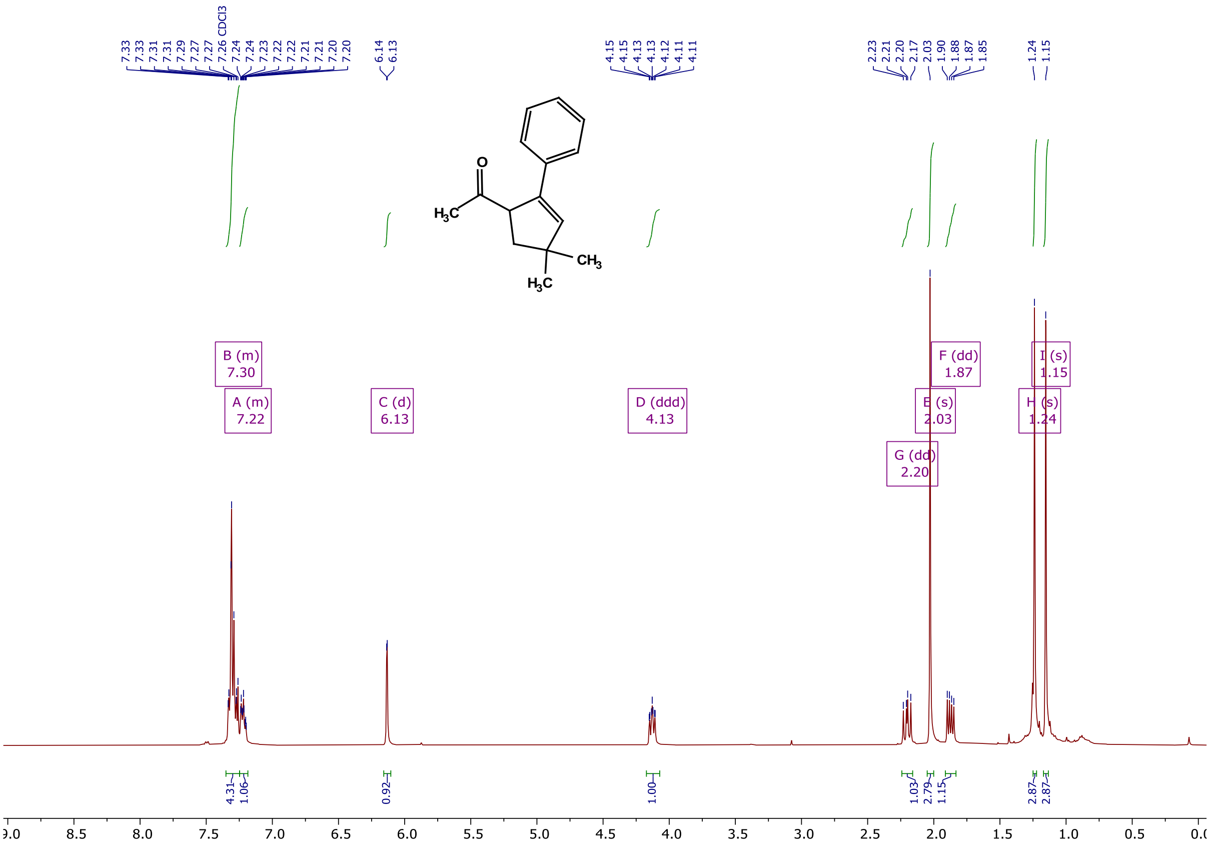

^{13}C NMR (101 MHz, Chloroform-*d*) (**3aad**):

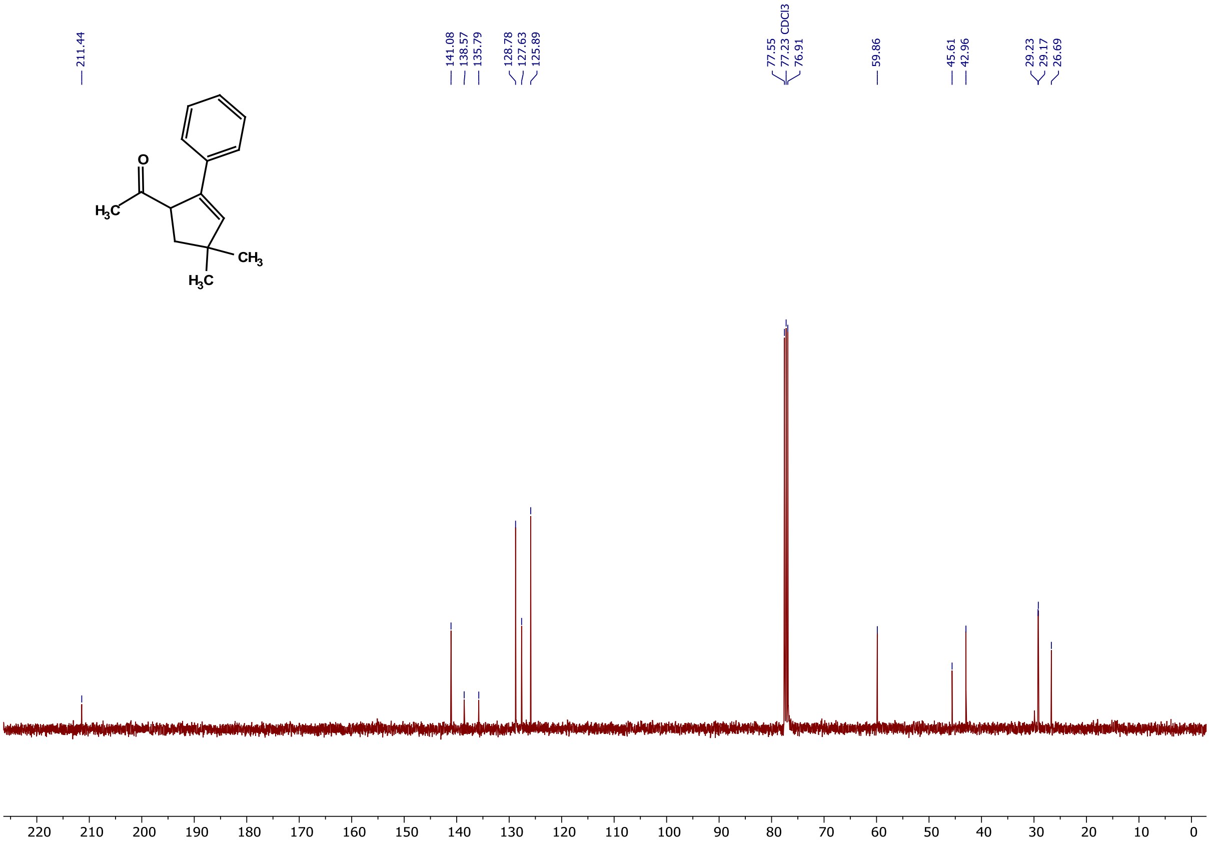

DEPT-135 NMR (101 MHz, Chloroform-*d*) (**3aad**):

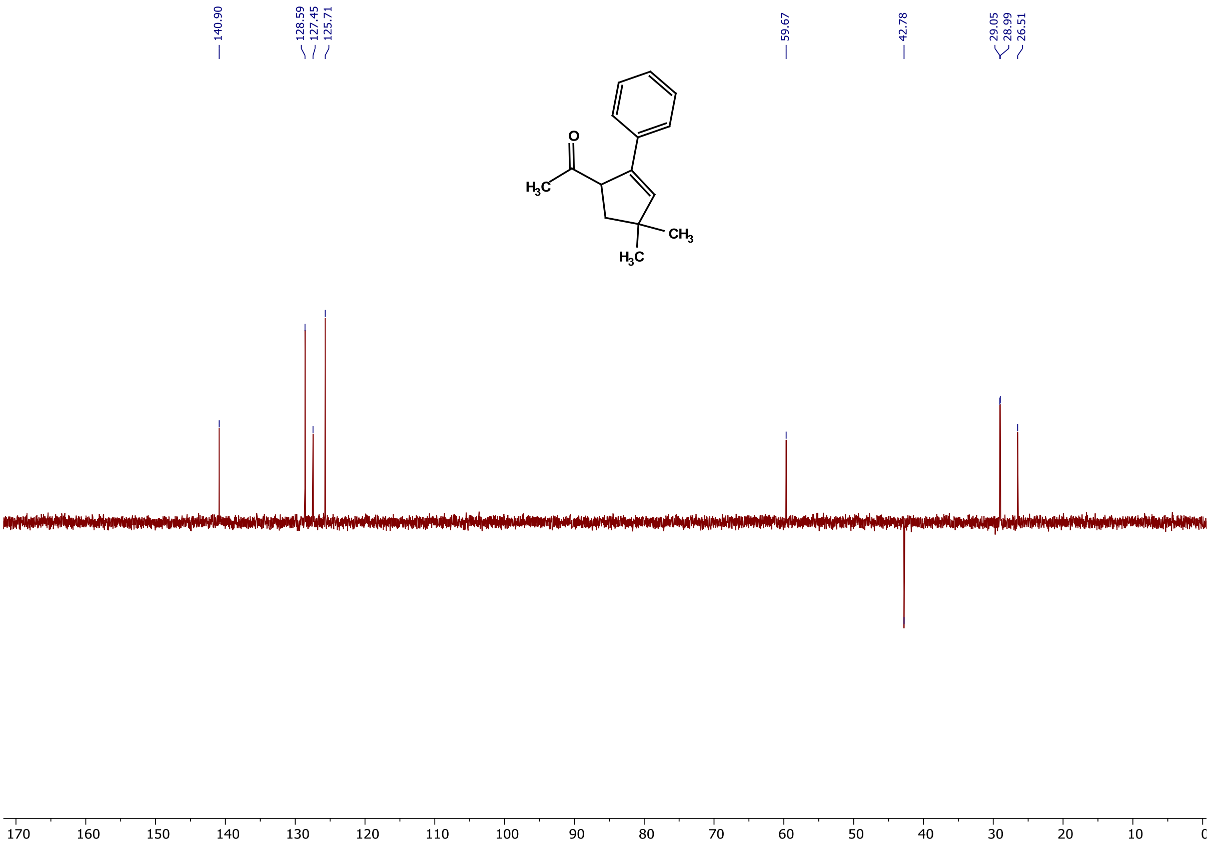

^{1}H NMR (500 MHz, Chloroform-*d*) (**3aaf**):

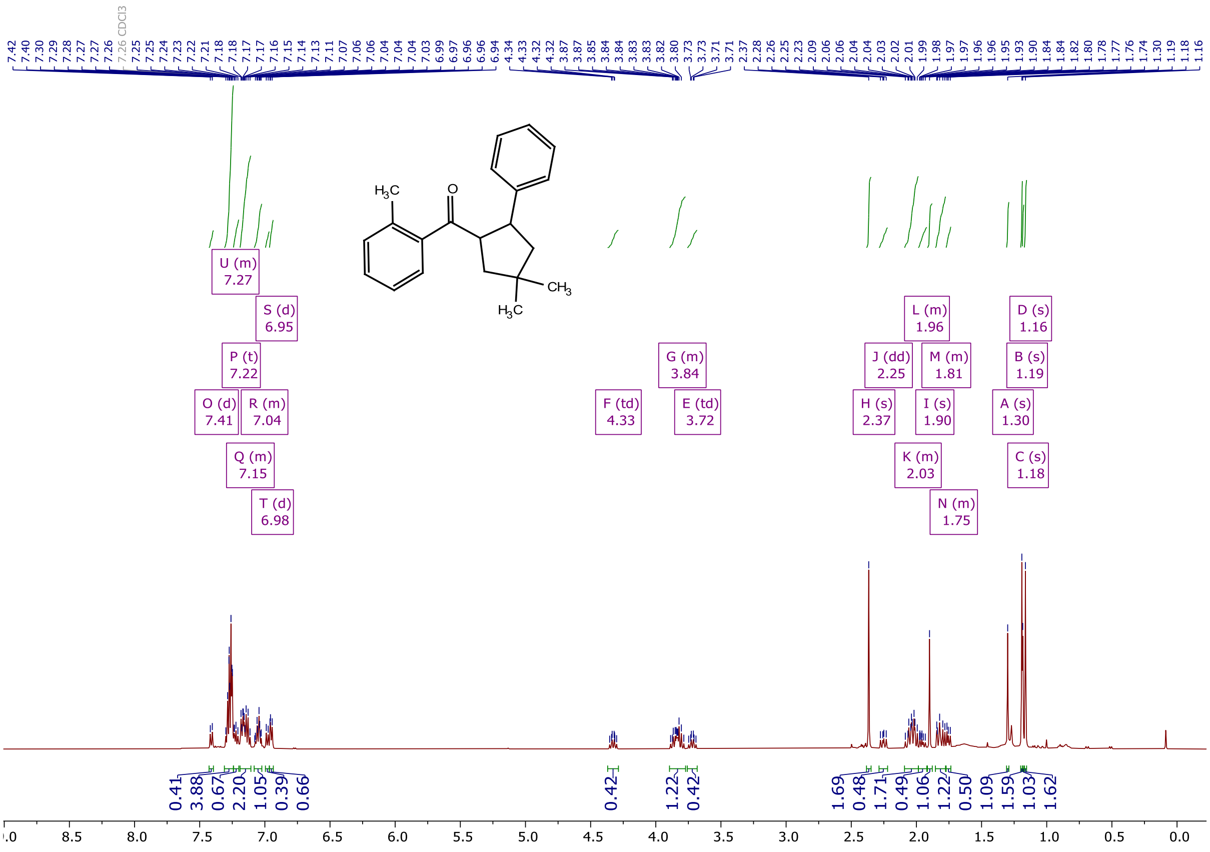

^{13}C NMR (126 MHz, Chloroform-*d*) (**3aaf**):

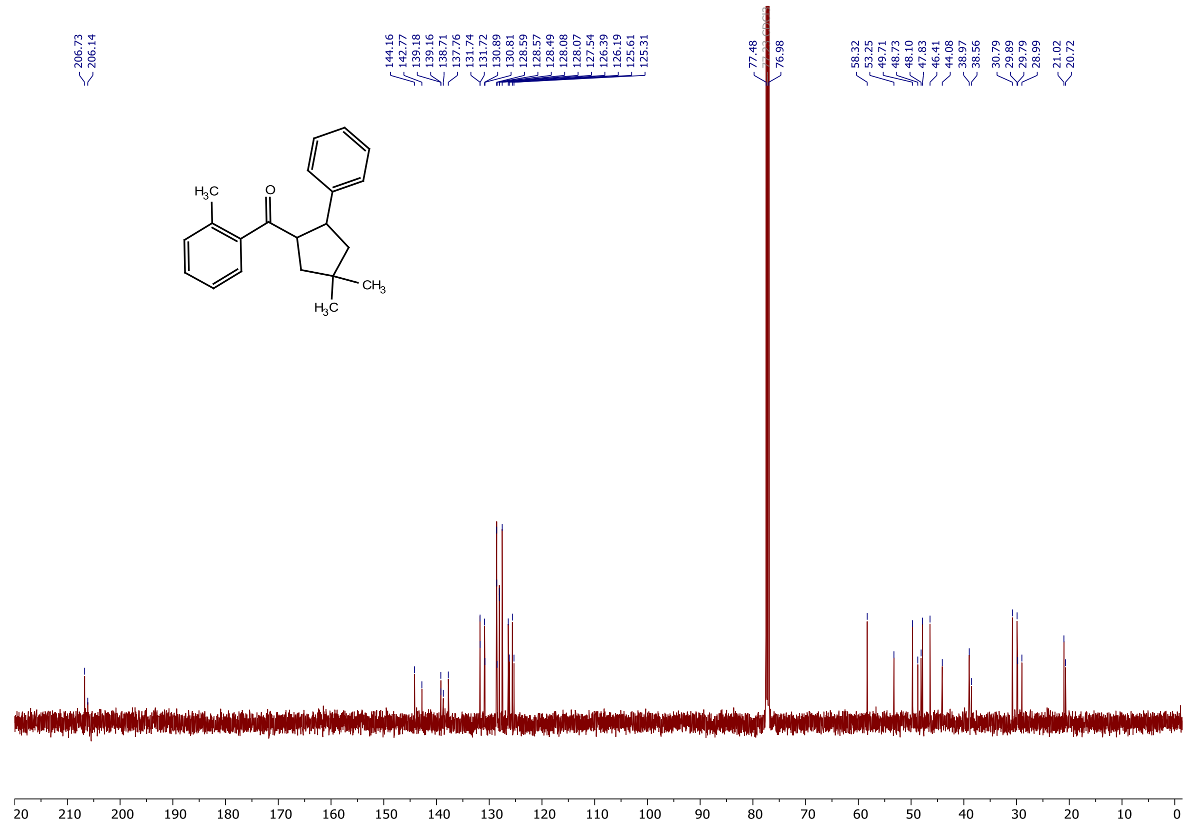

DEPT-135 NMR (126 MHz, Chloroform-*d*) (**3aaf**):

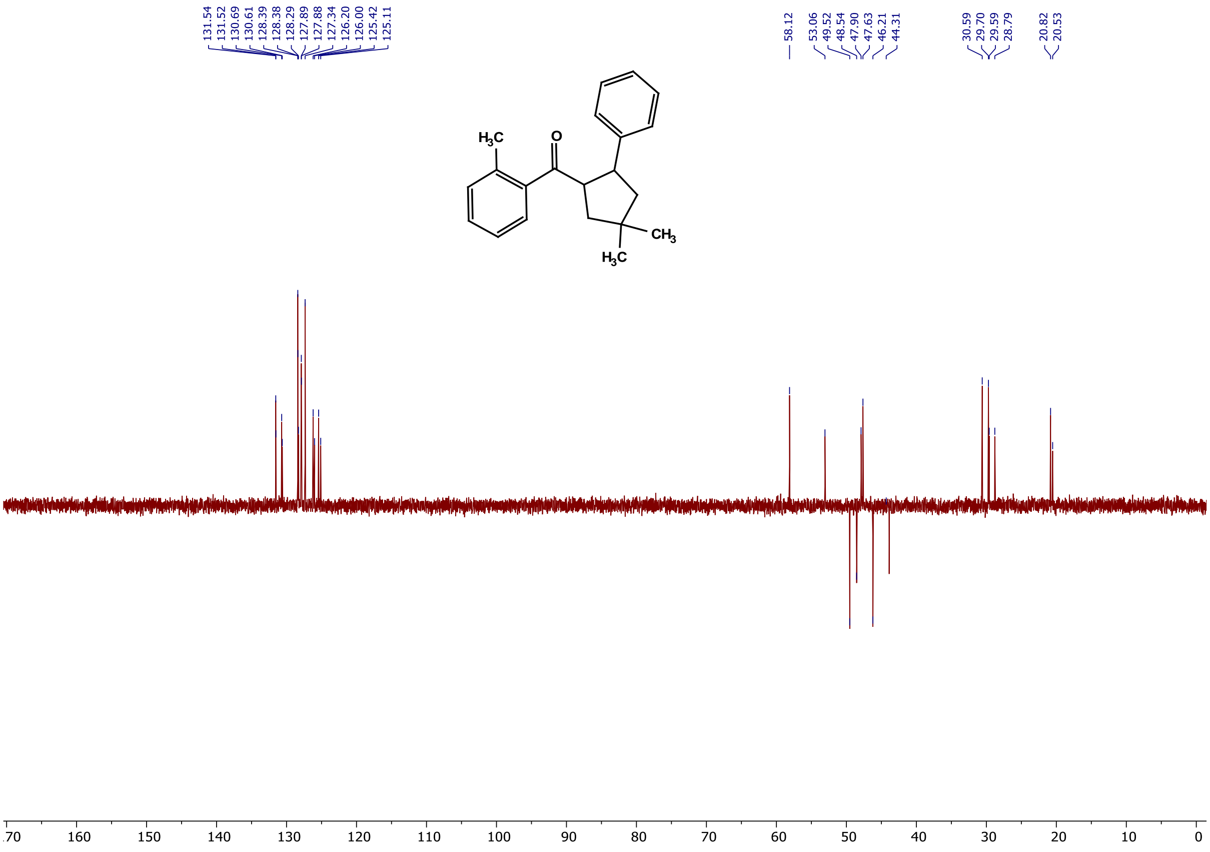

^{1}H NMR (400 MHz, Chloroform-*d*) (Major diastereomer) (**3aah**):

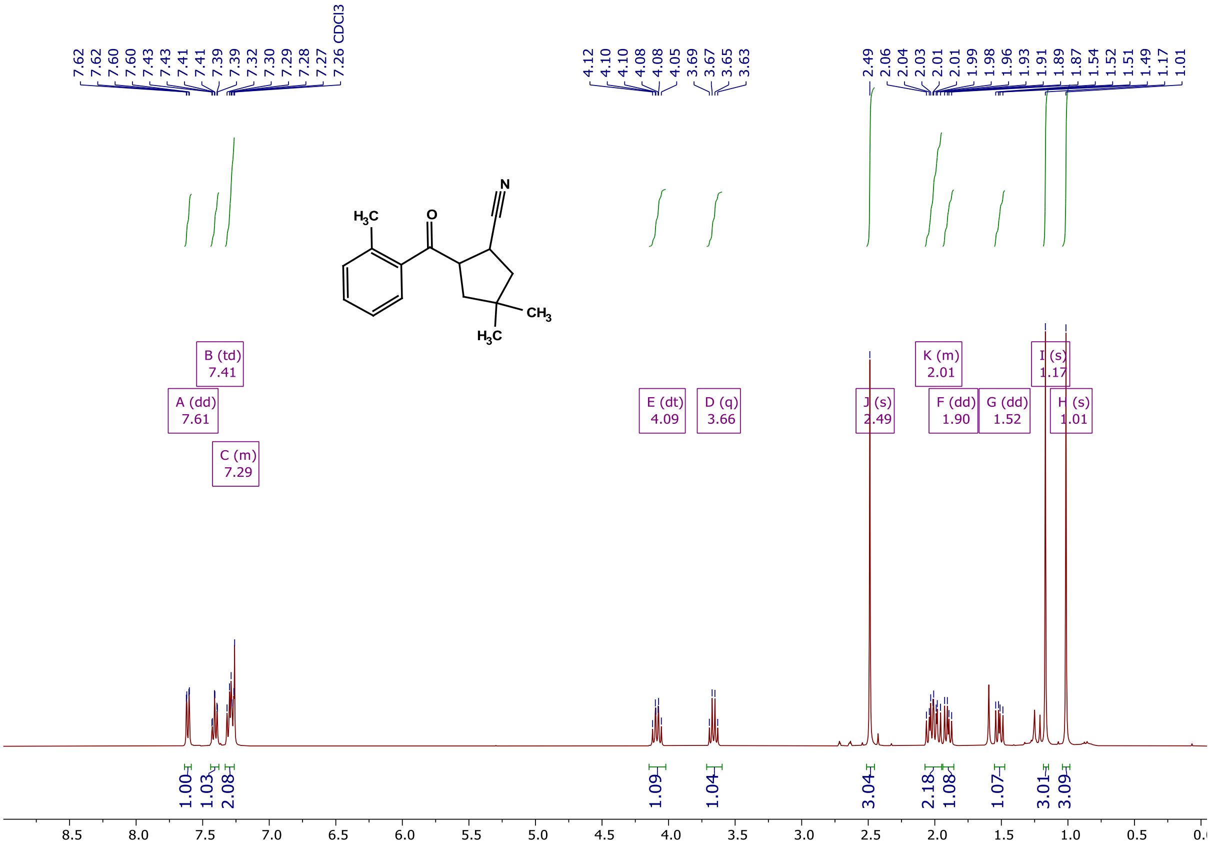

^{13}C NMR (101 MHz, Chloroform-*d*) (Major diastereomer) (**3aah**):

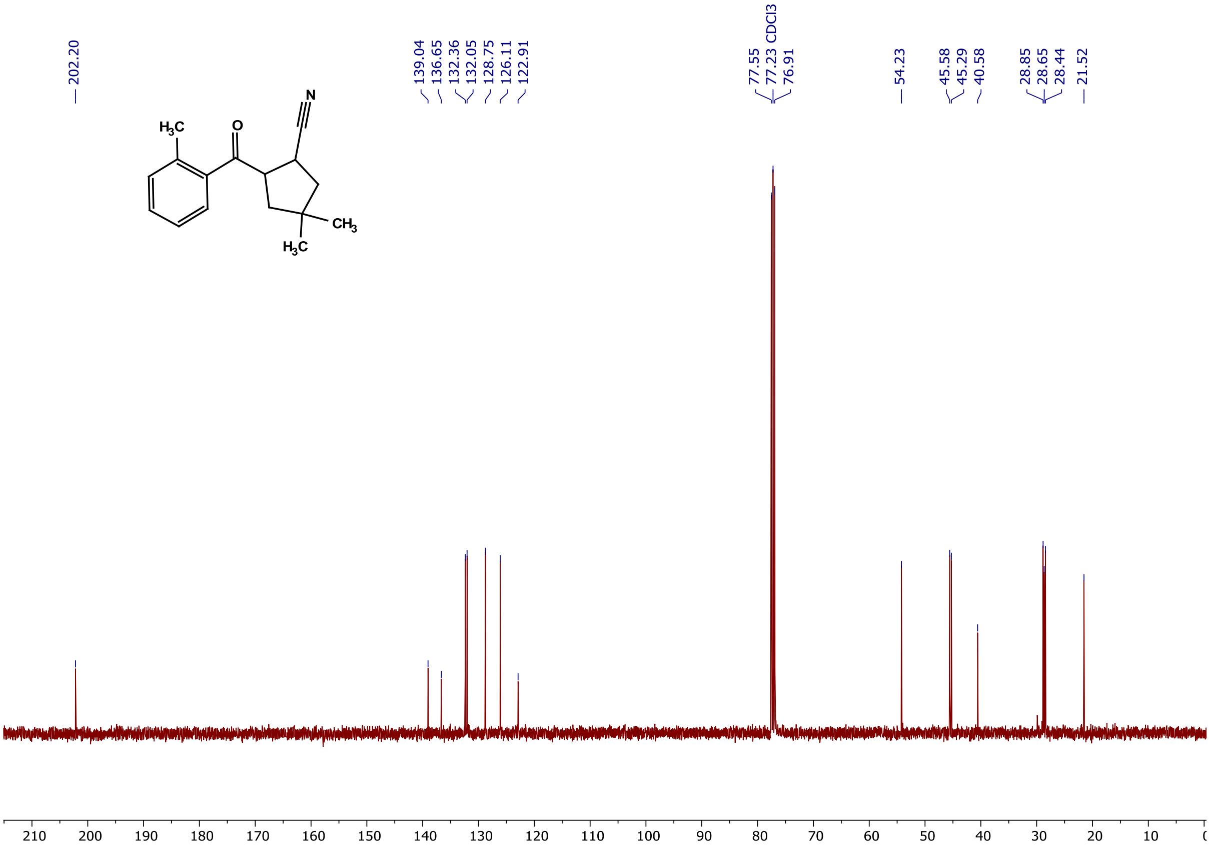

DEPT-135 NMR (101 MHz, Chloroform-*d*) (Major diastereomer) (**3aah**):

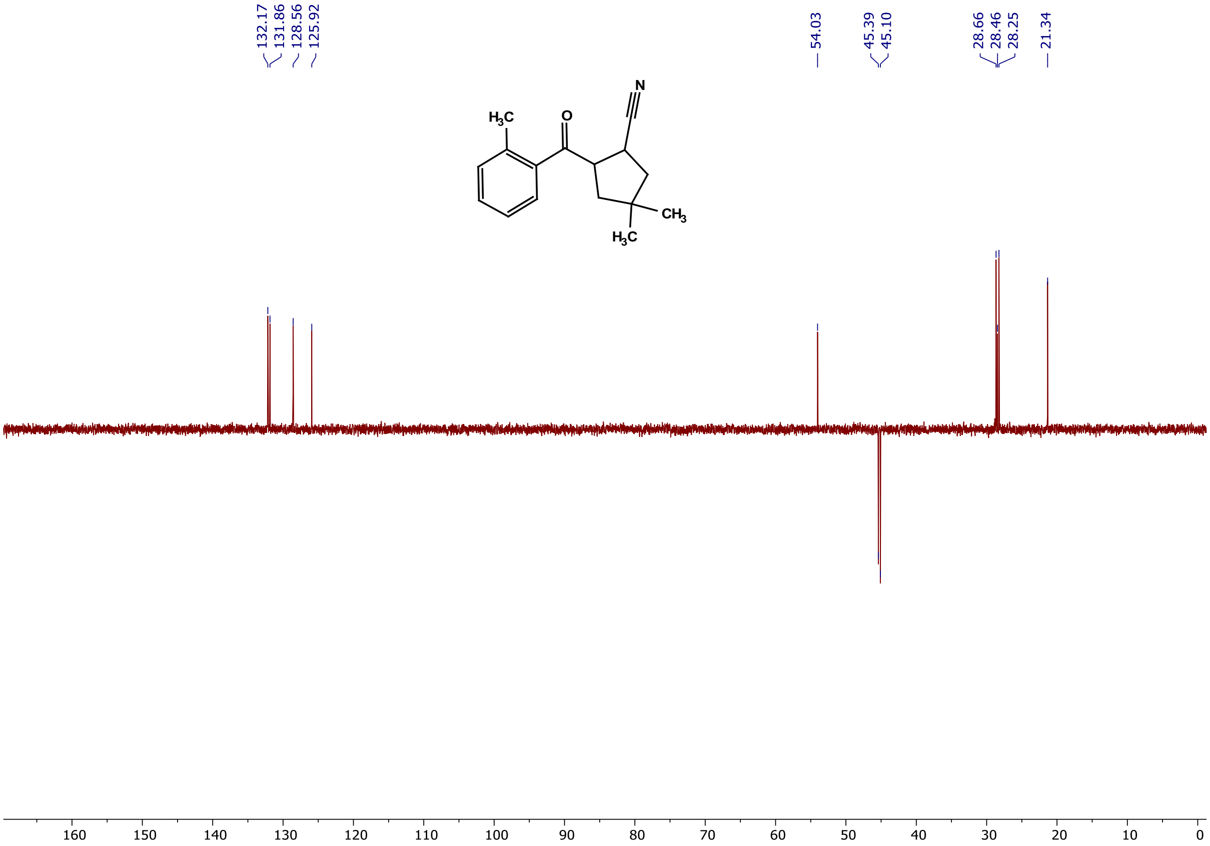

^1H NMR (400 MHz, Chloroform-*d*) (Minor diastereomer) (**3aah**):

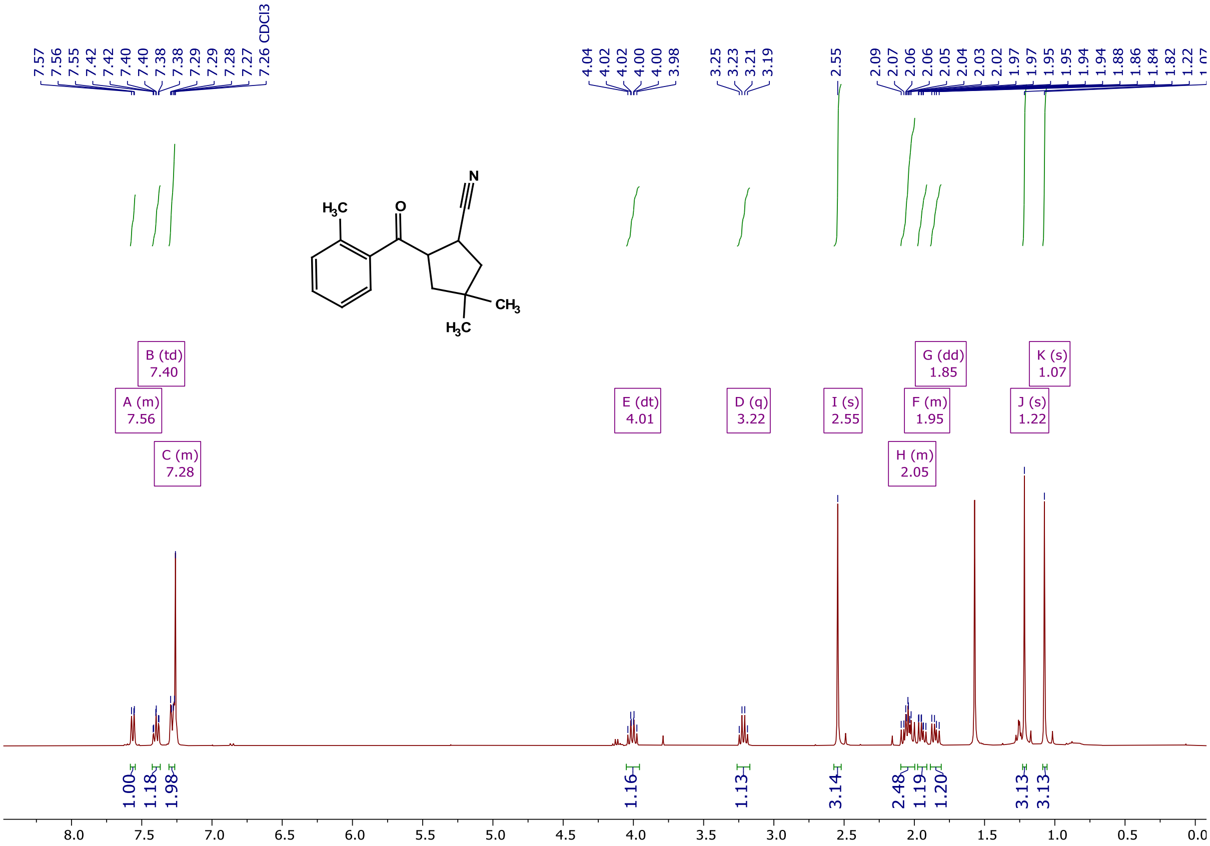

^{13}C NMR (101 MHz, Chloroform-*d*) (Minor diastereomer) (**3aah**):

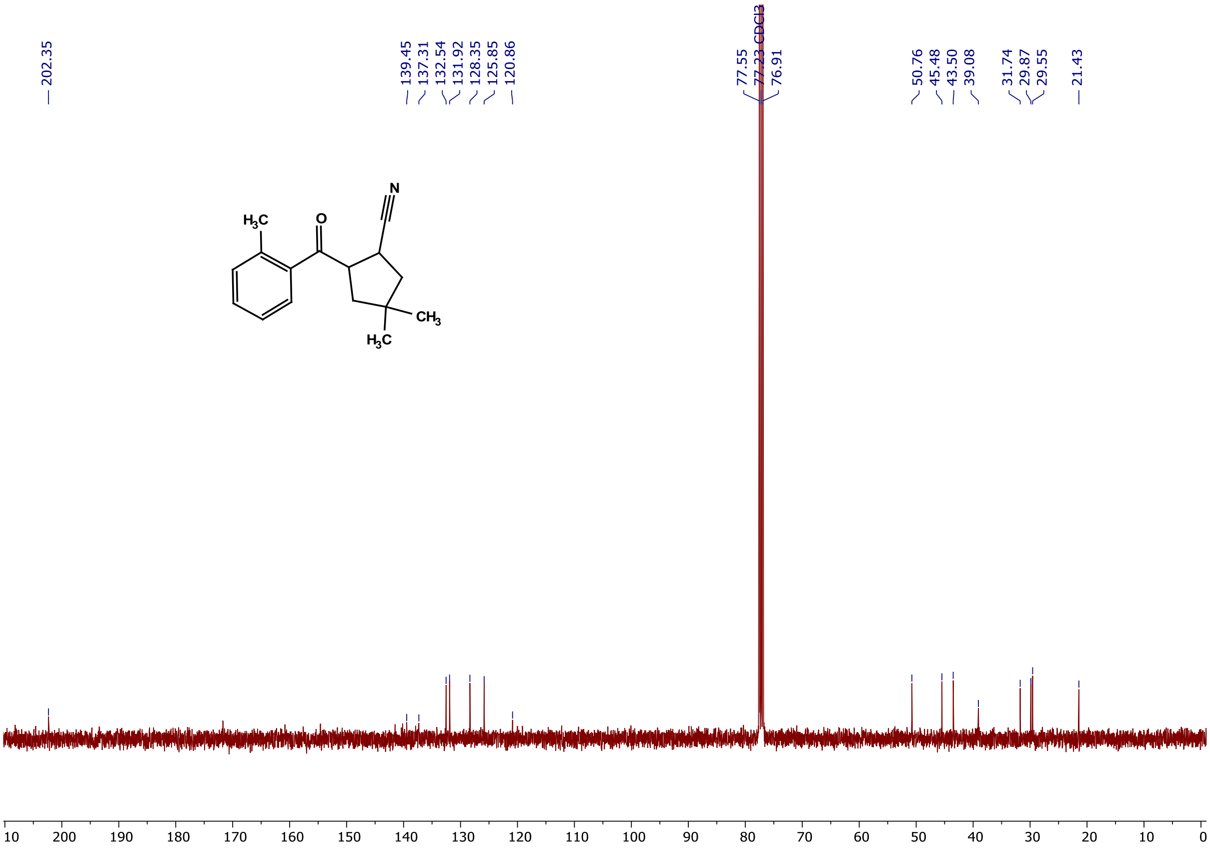

DEPT-135 NMR (101 MHz, Chloroform-*d*) (Minor diastereomer) (**3aah**):

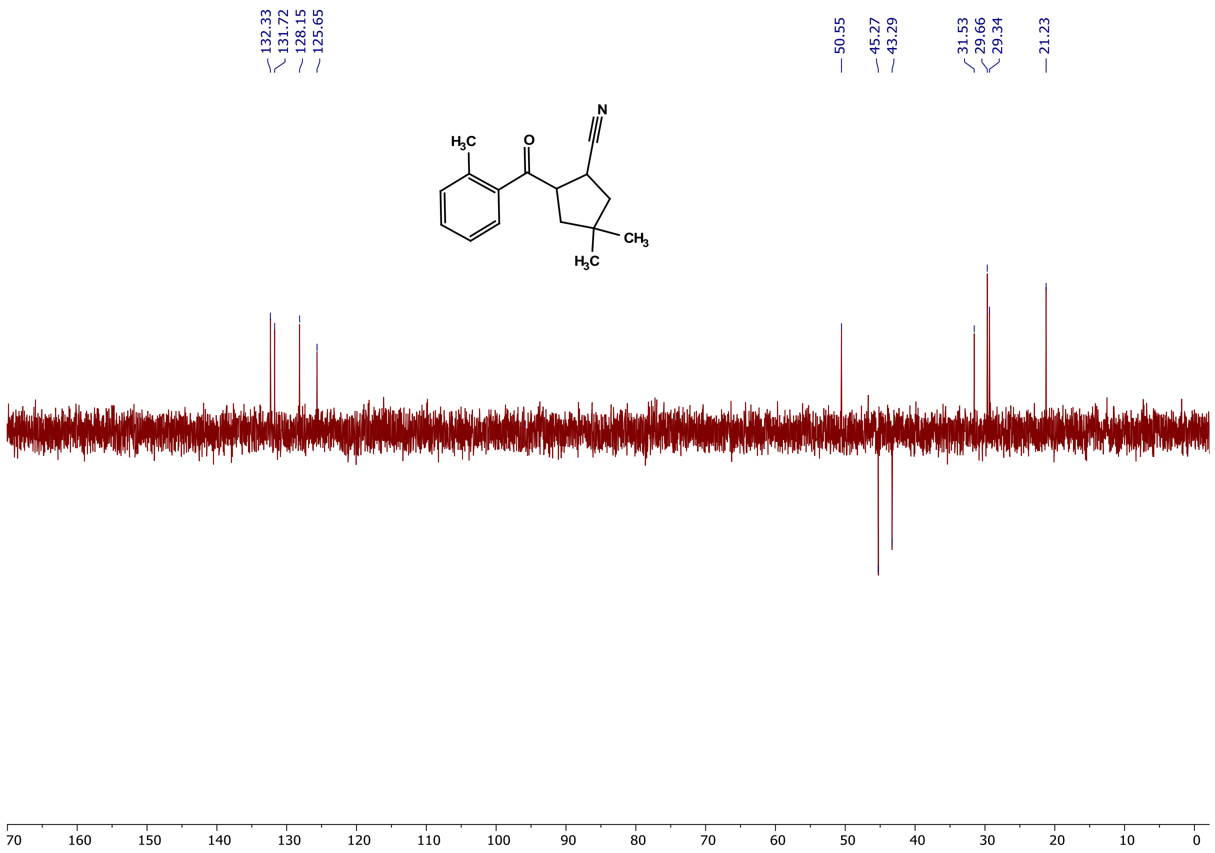

^{1}H NMR (500 MHz, Chloroform-*d*) (**4**):

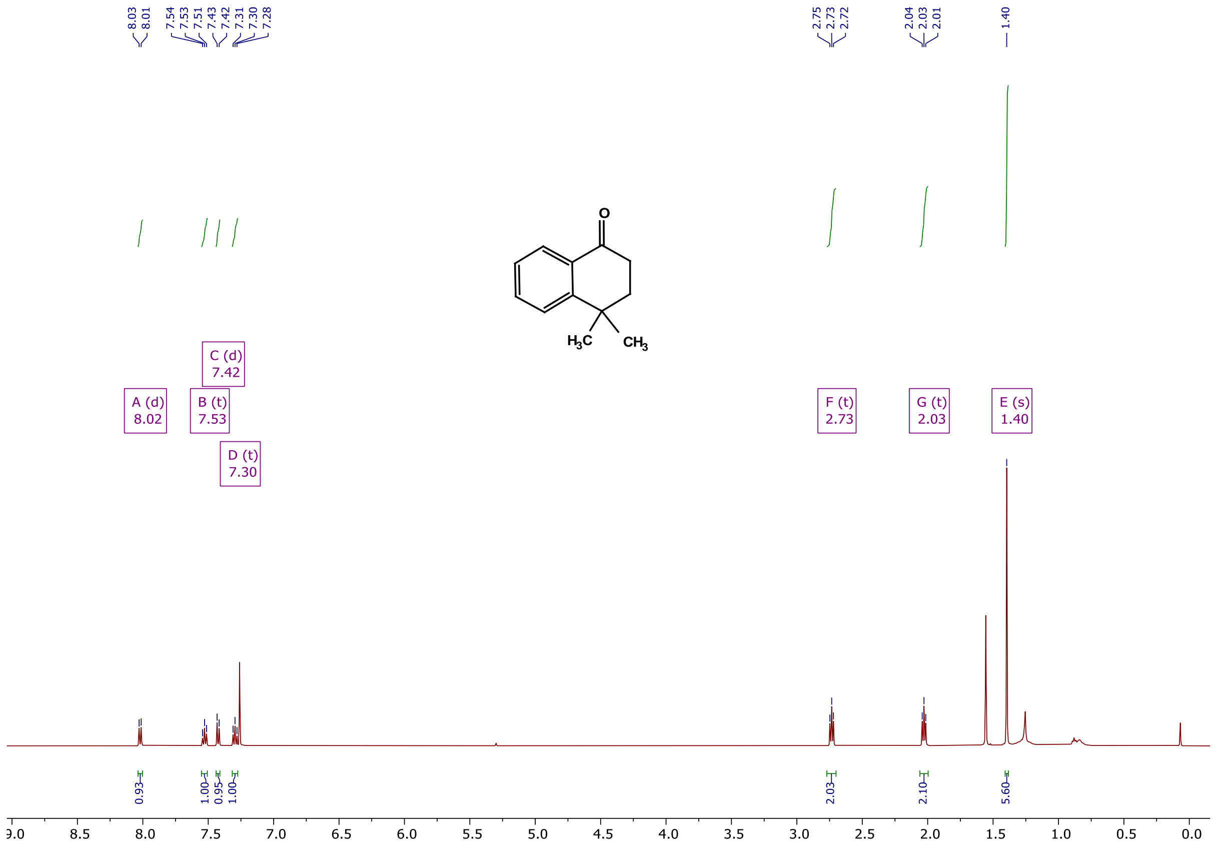

^{13}C NMR (126 MHz, Chloroform-*d*) (**4**):

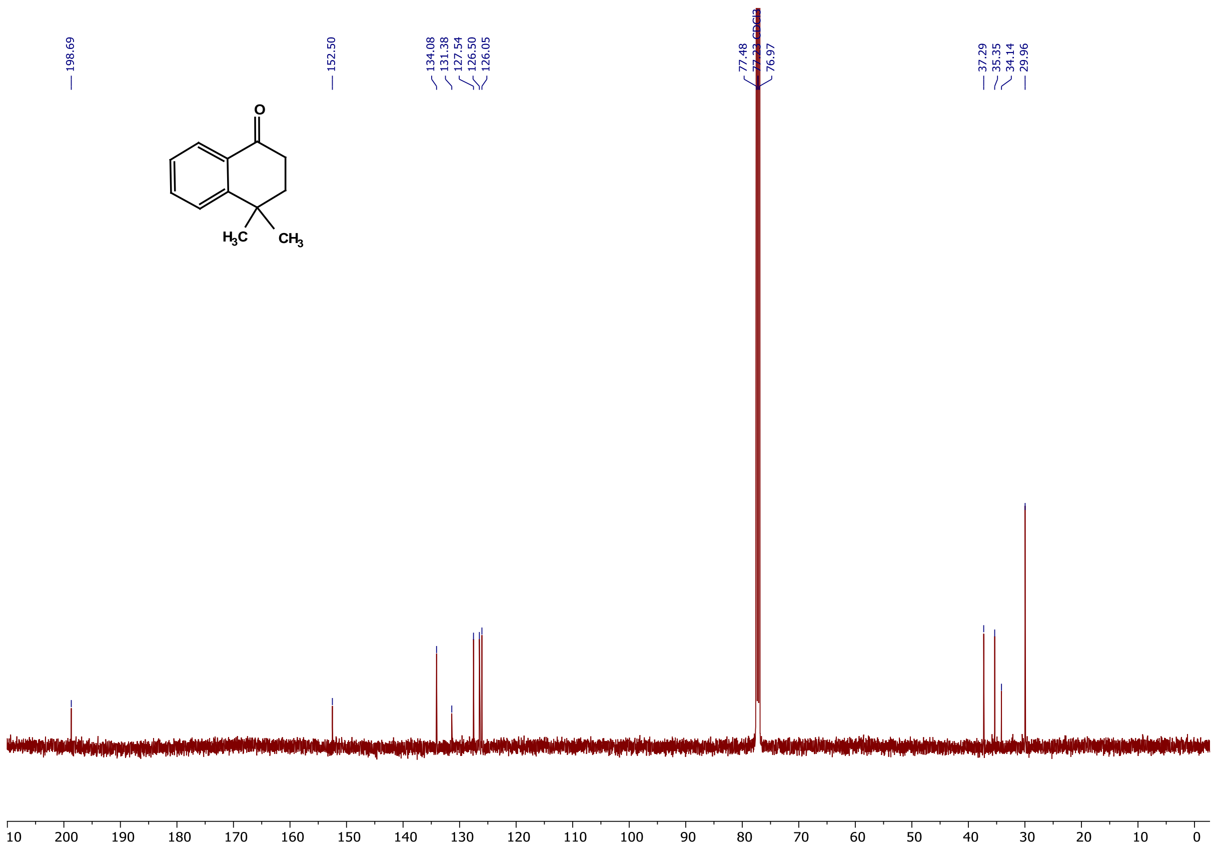

DEPT-135 NMR (126 MHz, Chloroform-*d*) (**4**):

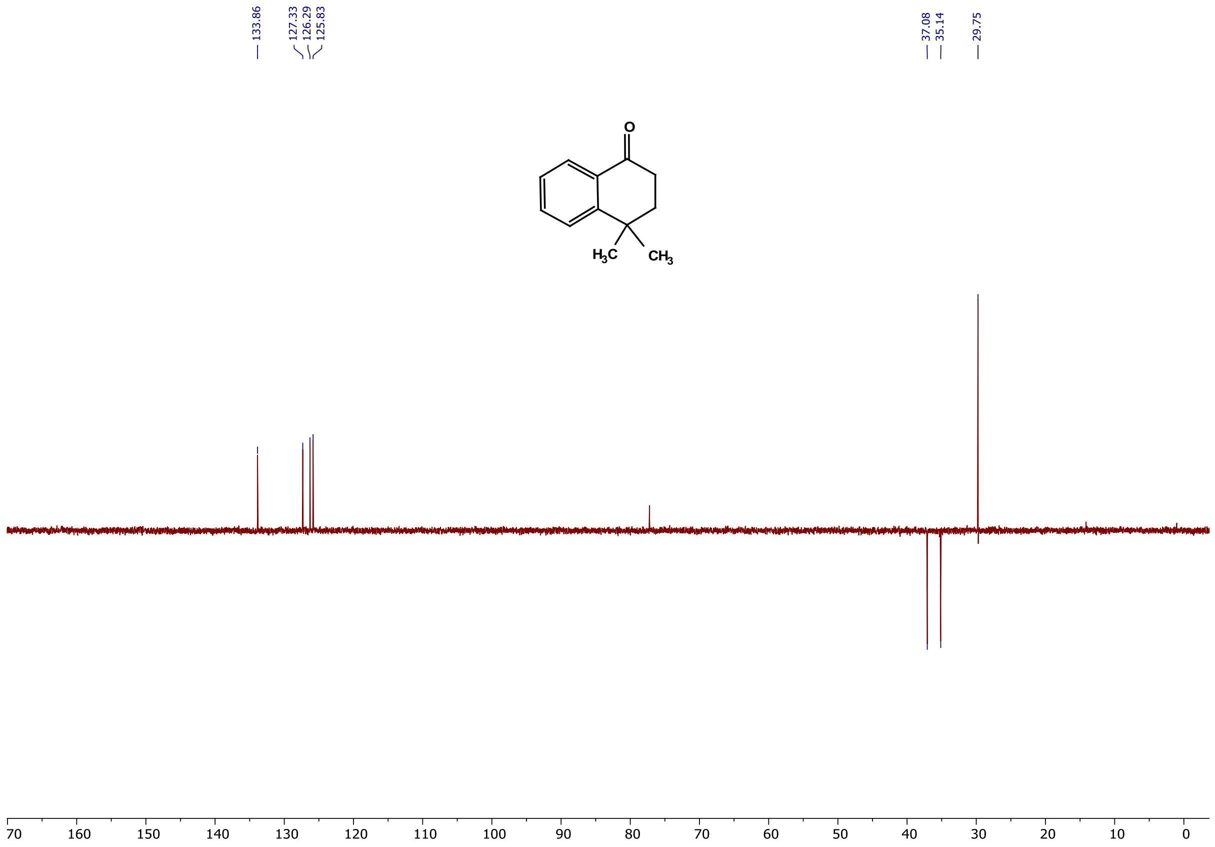

^{1}H NMR (400 MHz, Chloroform-*d*) (**5**):

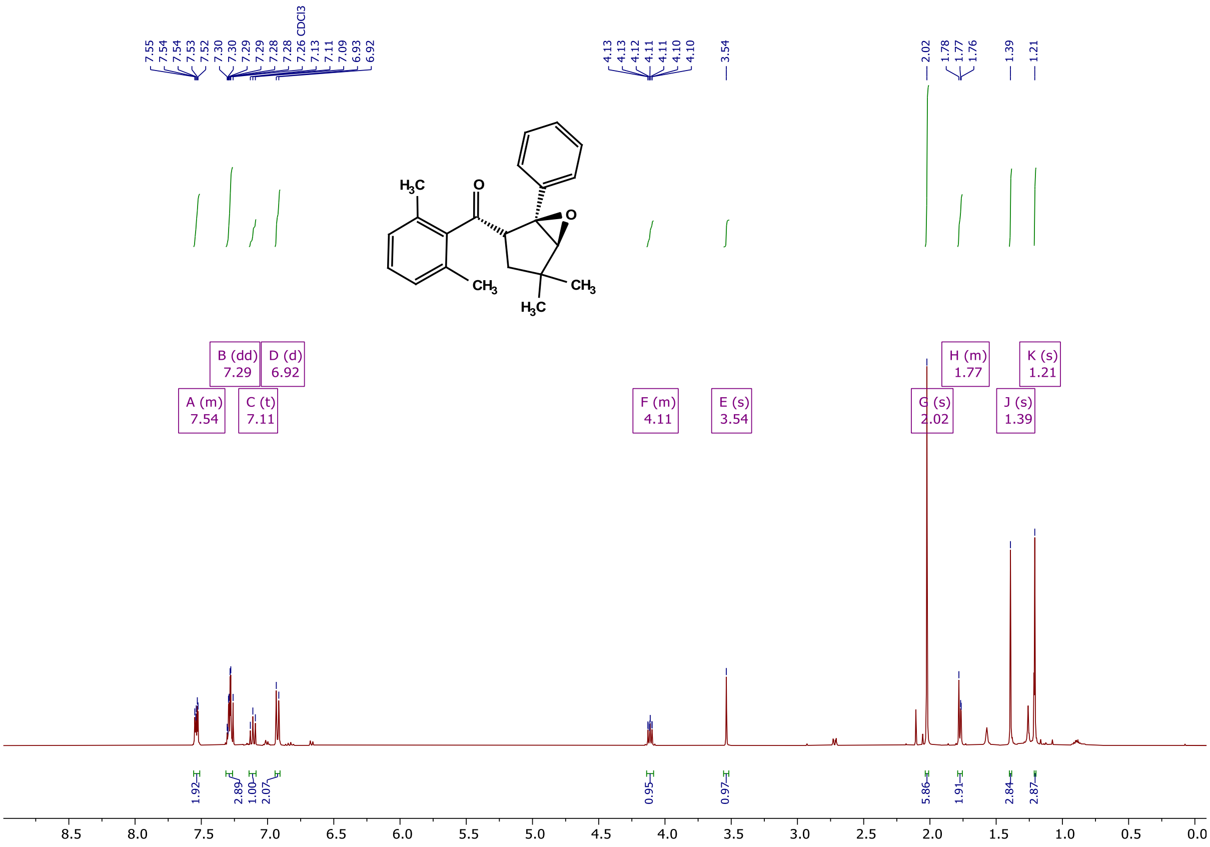

^{13}C NMR (101 MHz, Chloroform-*d*) (**5**):

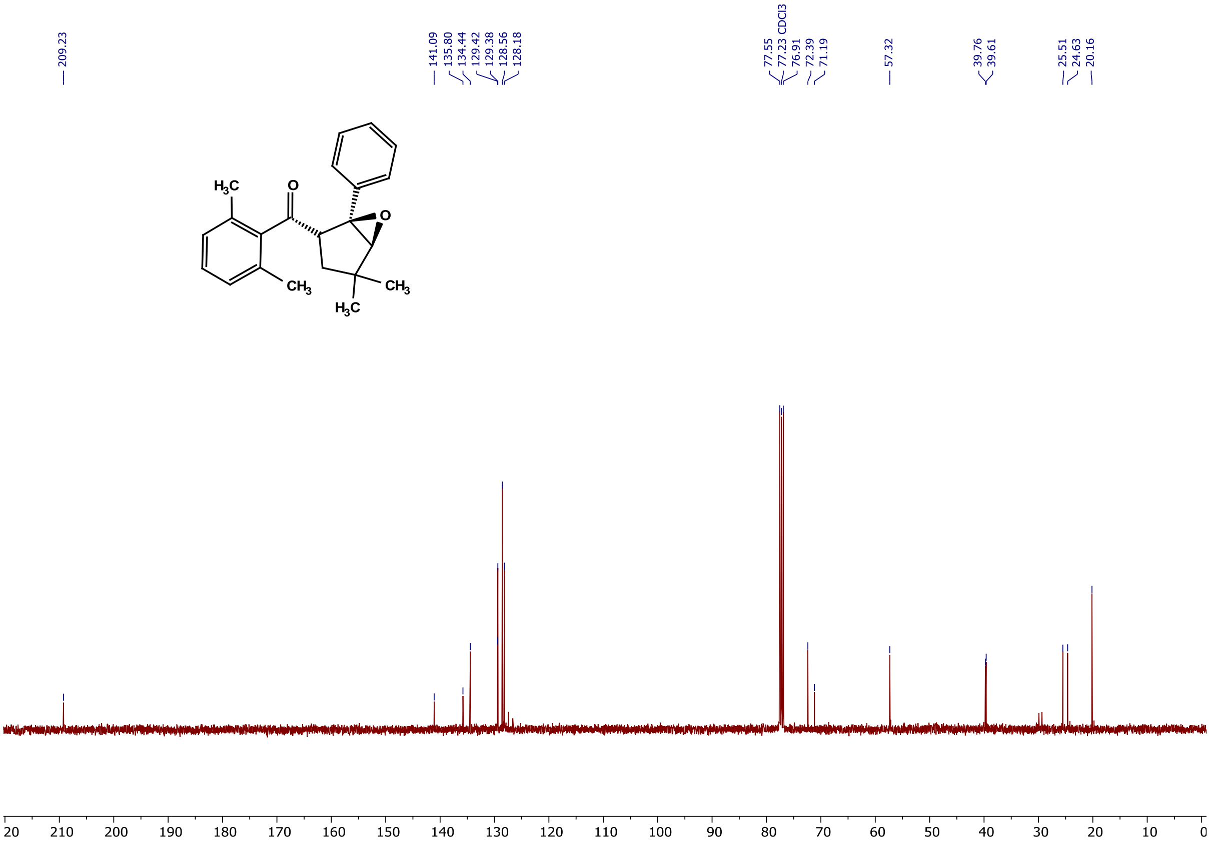

DEPT-135 NMR (101 MHz, Chloroform-*d*) (**5**):

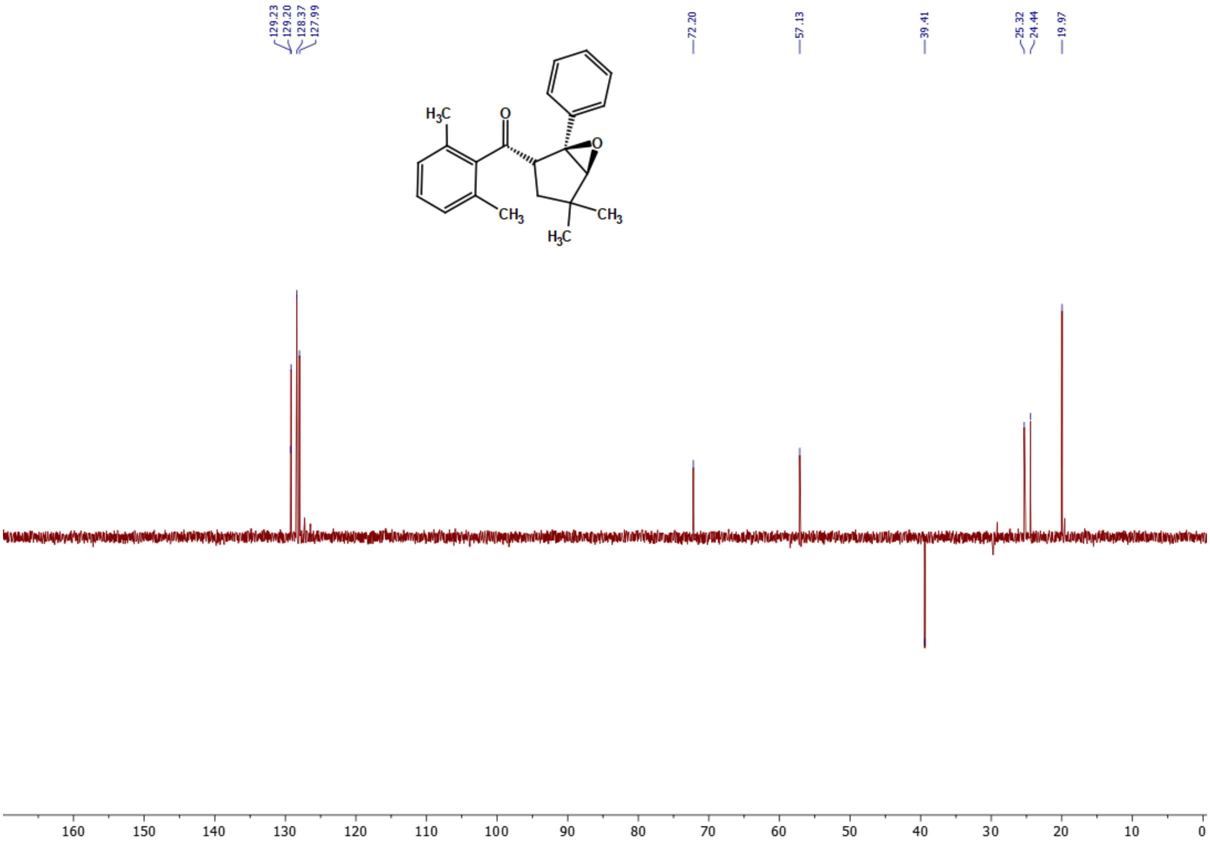

^{1}H NMR (400 MHz, Chloroform-*d*) (**6**):

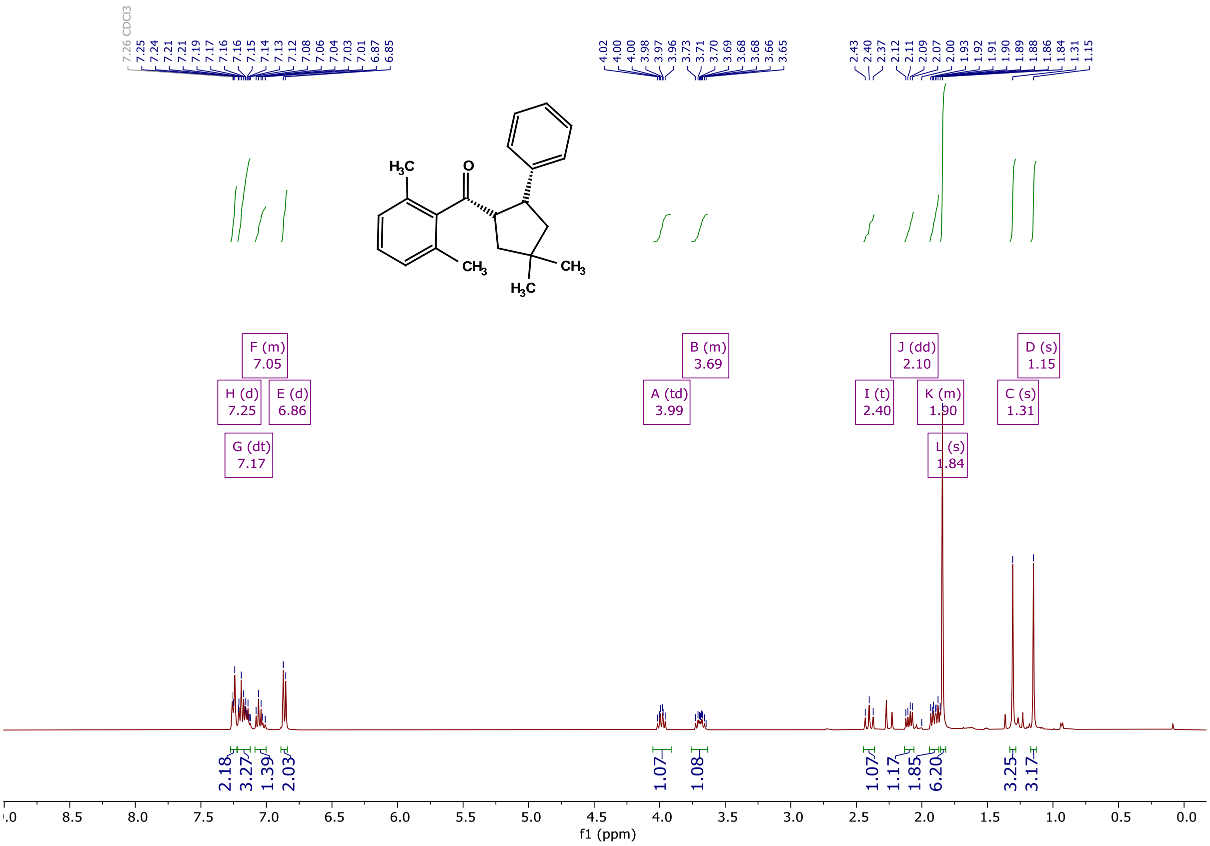

^{13}C NMR (101 MHz, Chloroform-*d*) (**6**):

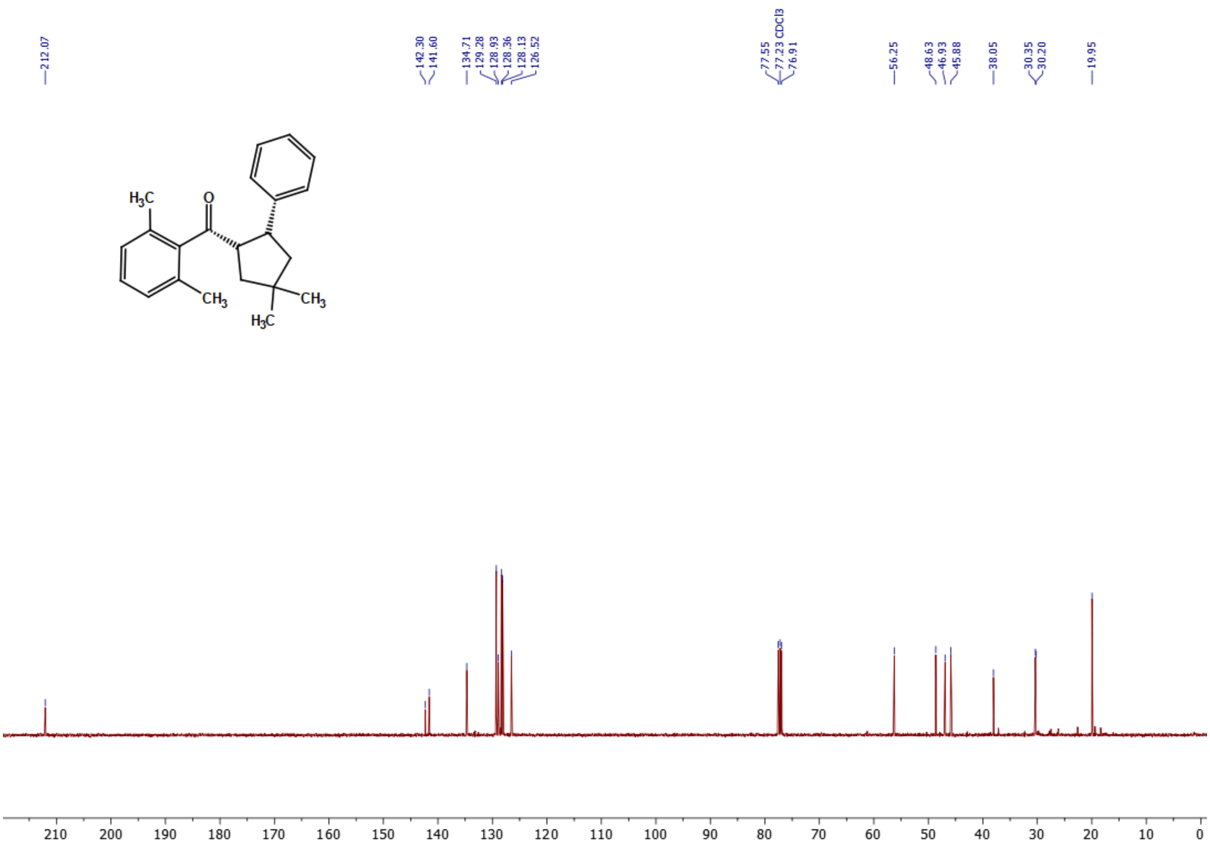

DEPT-135 NMR (101 MHz, Chloroform-*d*) (**6**):

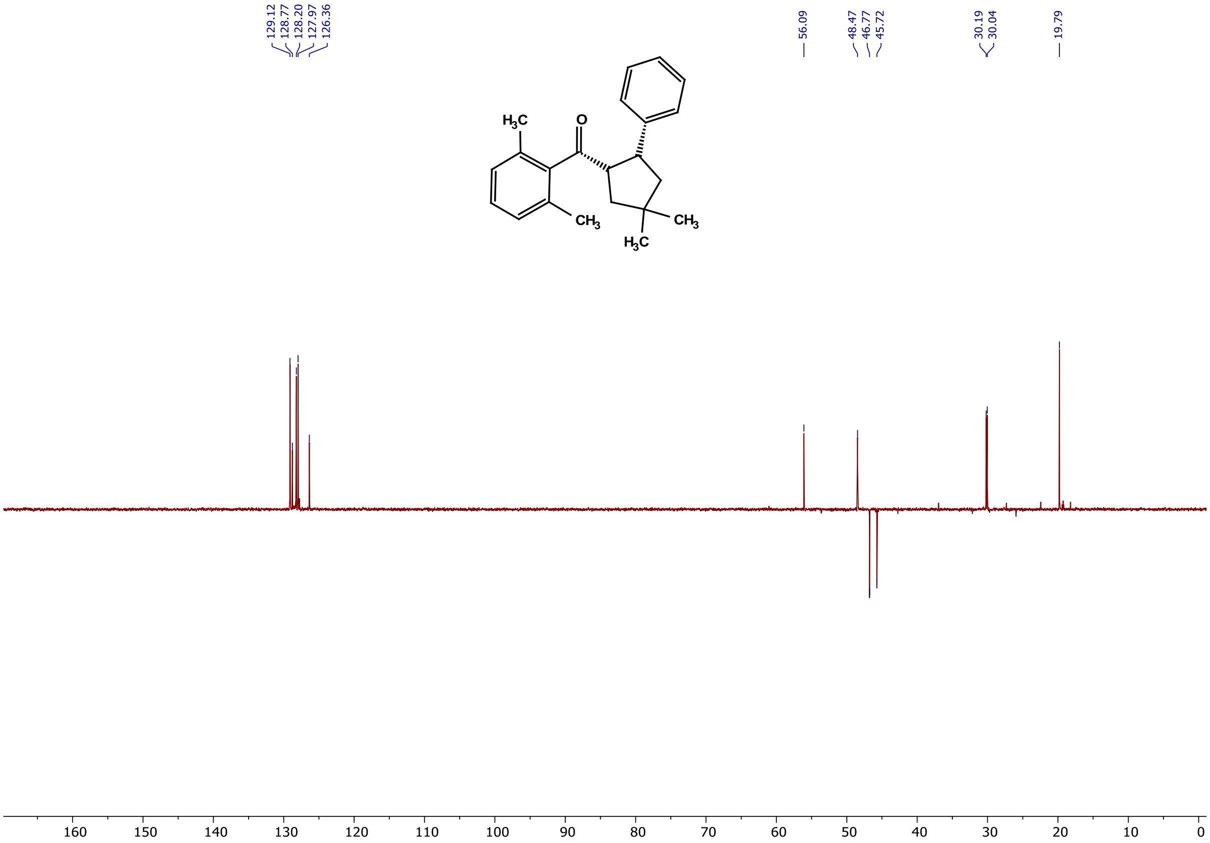

^{1}H NMR (400 MHz, Chloroform-*d*) (**3ar'**):

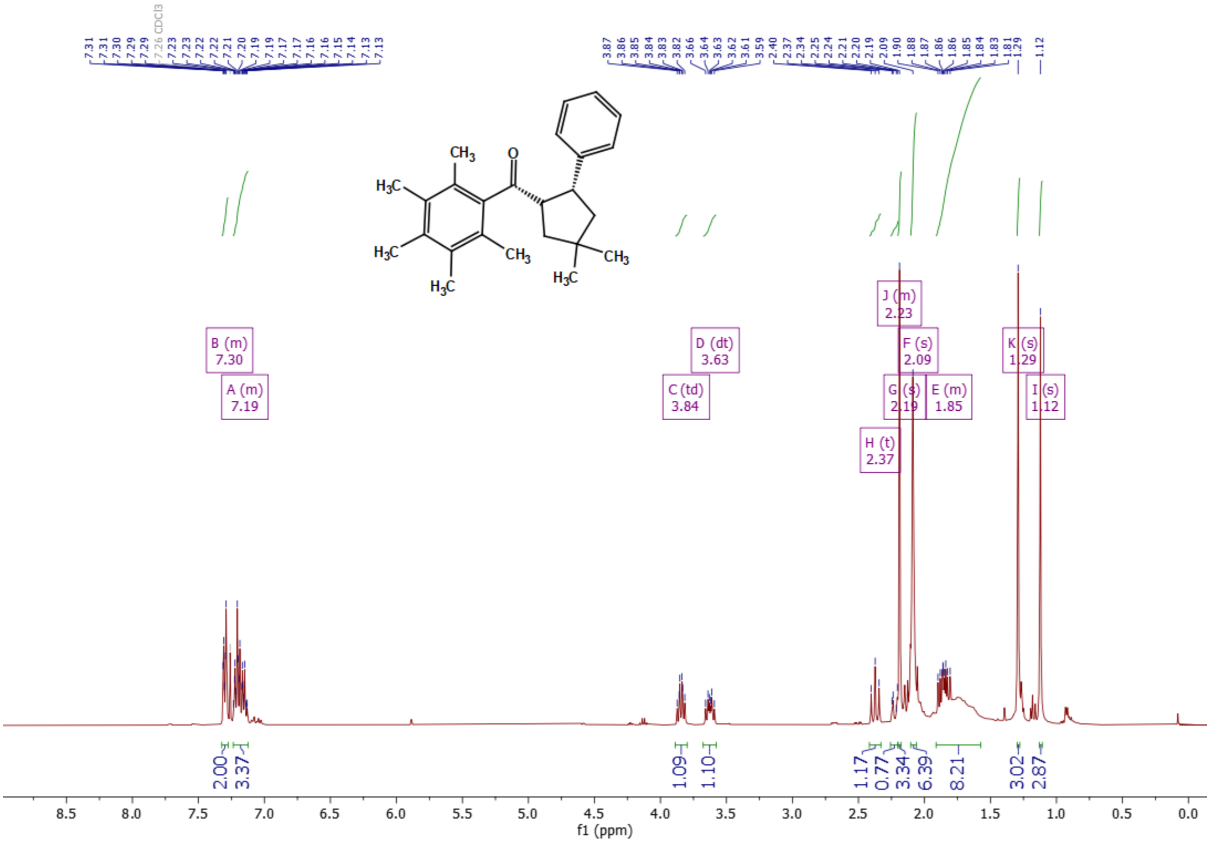

^{13}C NMR (101 MHz, Chloroform-*d*) (**3ar'**):

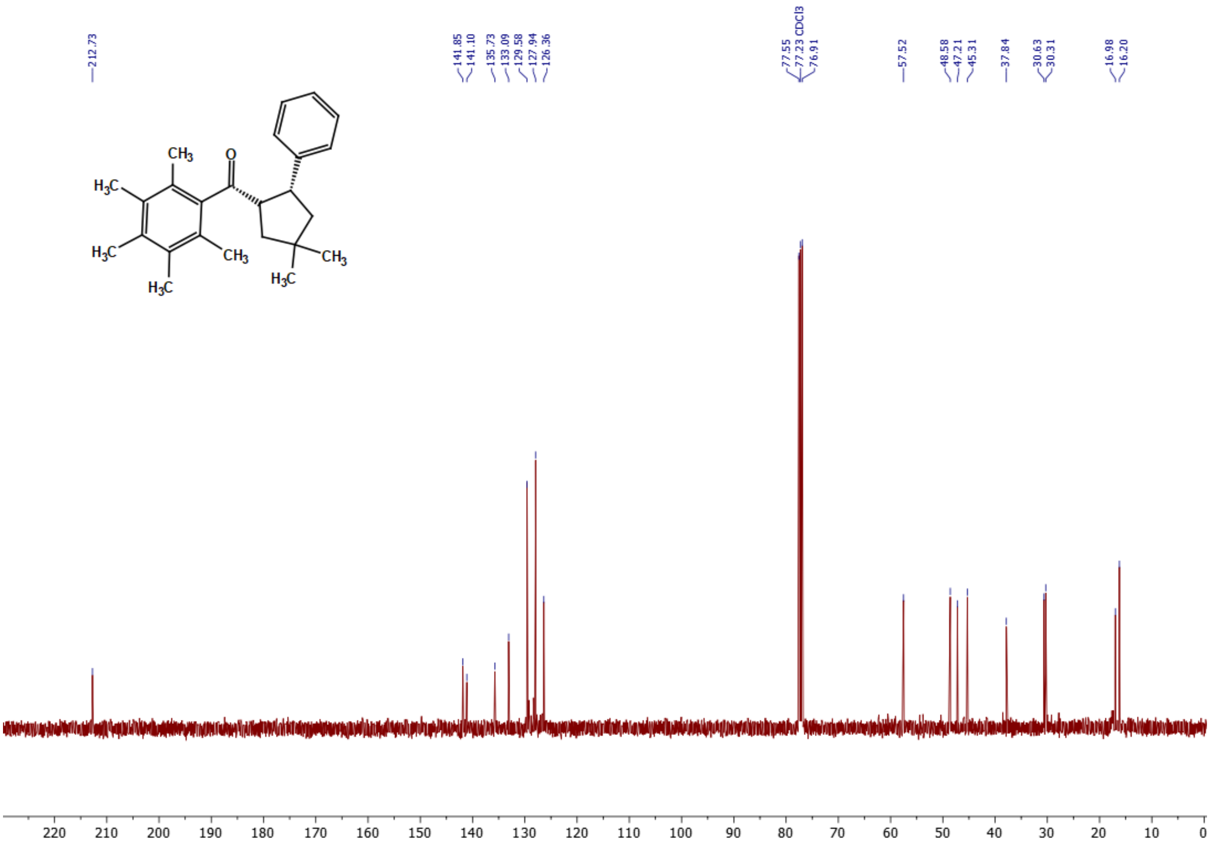

DEPT-135 NMR (101 MHz, Chloroform-*d*) (**3ar’**):

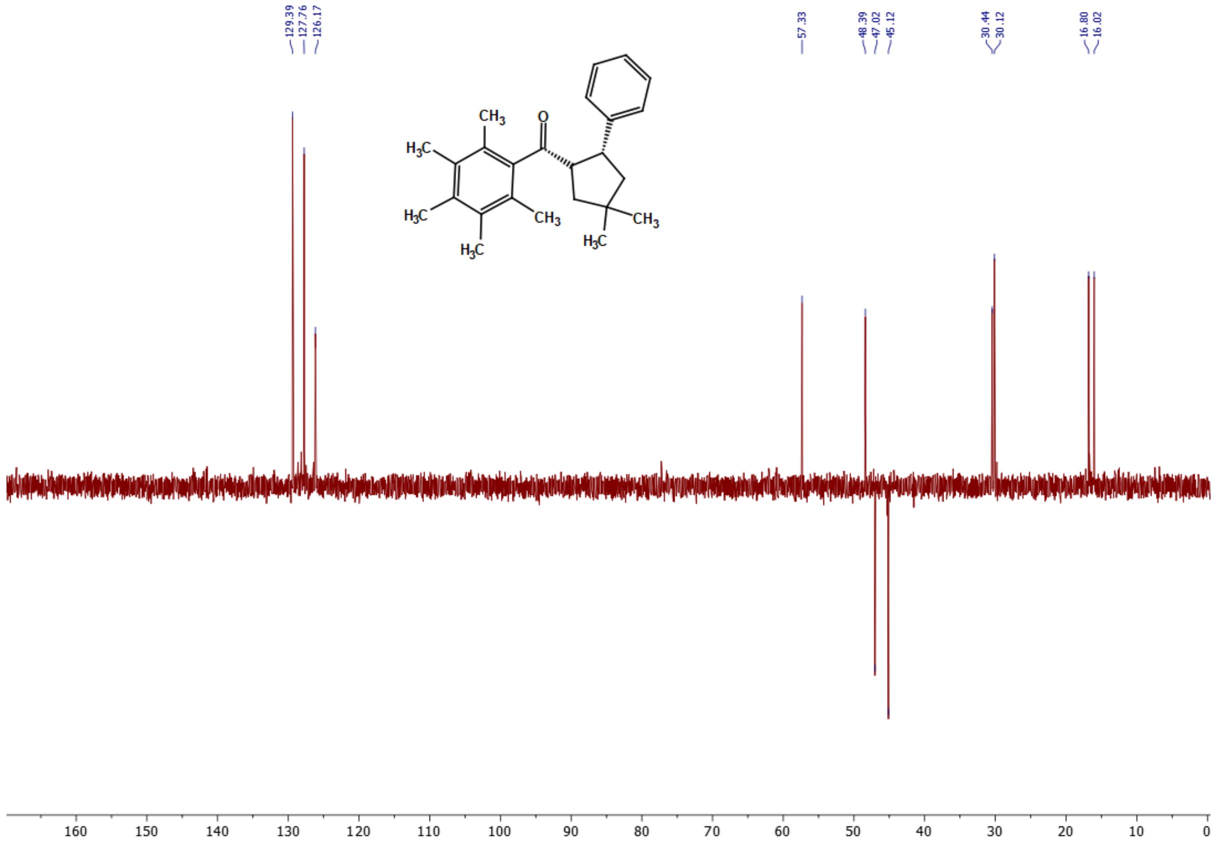

^{1}H NMR (400 MHz, Chloroform-*d*) (**7**):

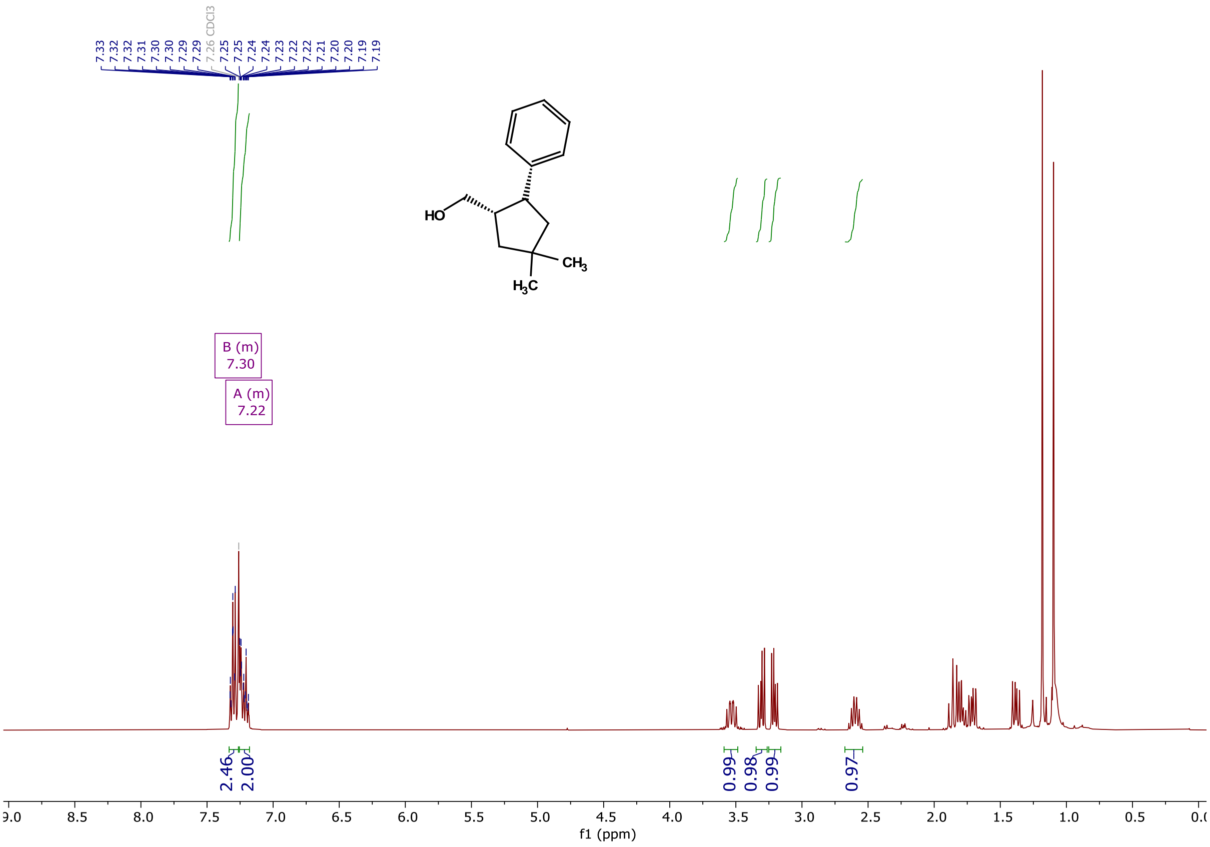

^{13}C NMR (101 MHz, Chloroform-*d*) (**7**):

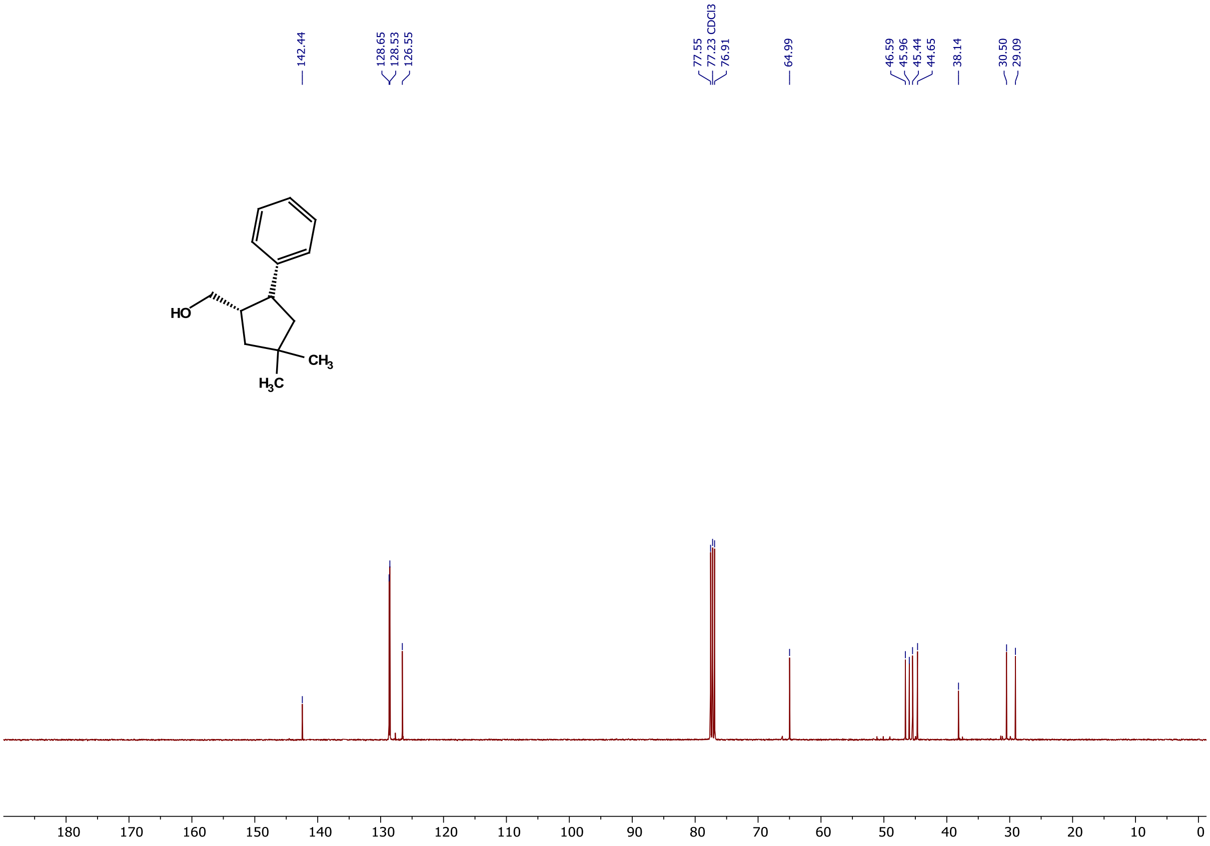

DEPT-135 NMR (101 MHz, Chloroform-*d*) (**7**):

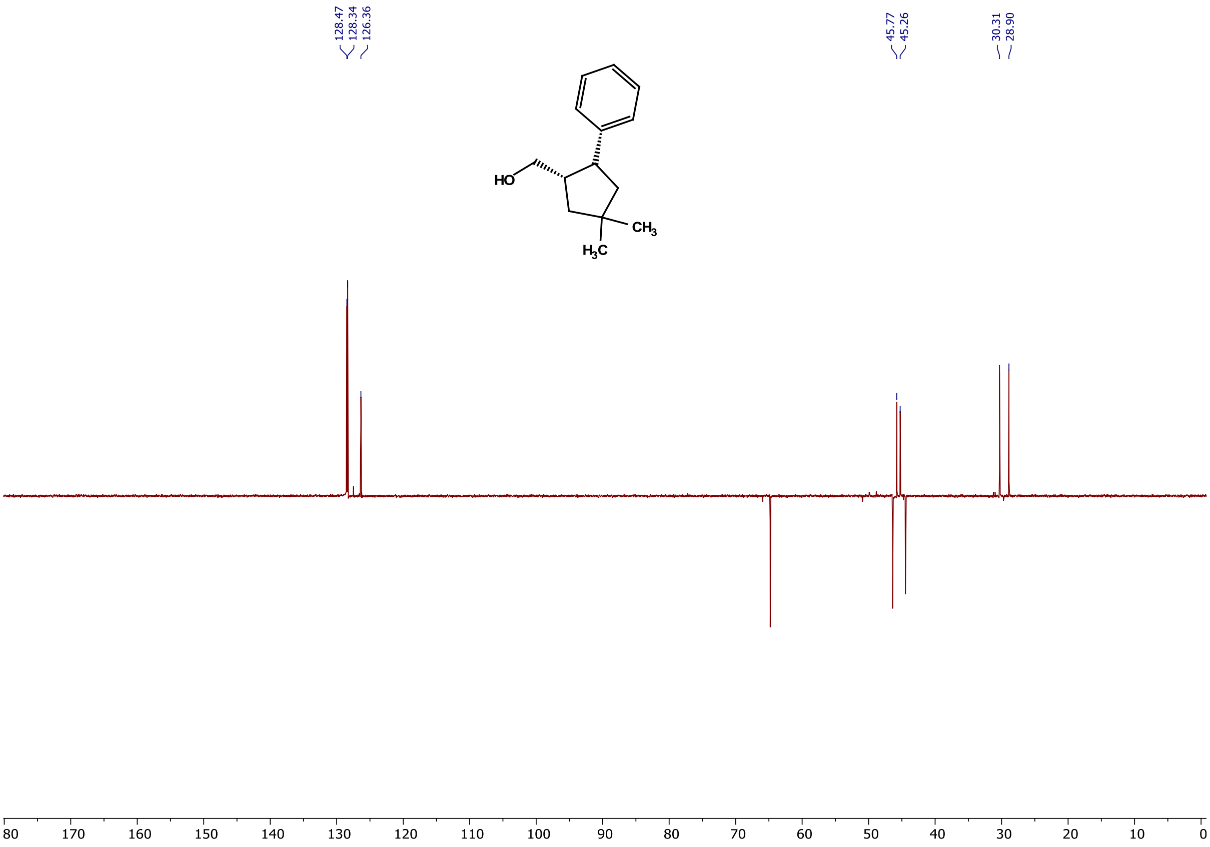

^{1}H NMR (400 MHz, Chloroform-*d*) (**8**):

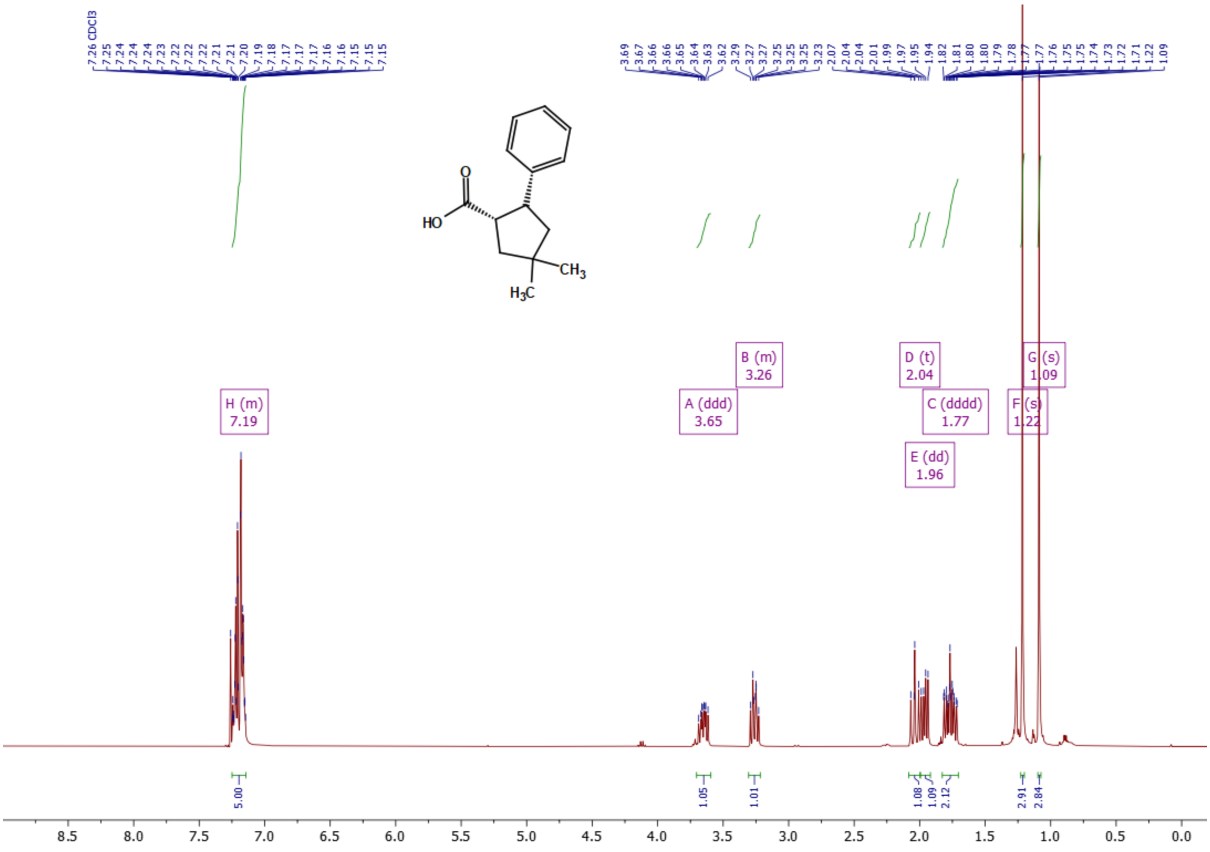

^{13}C NMR (101 MHz, Chloroform-*d*) (**8**):

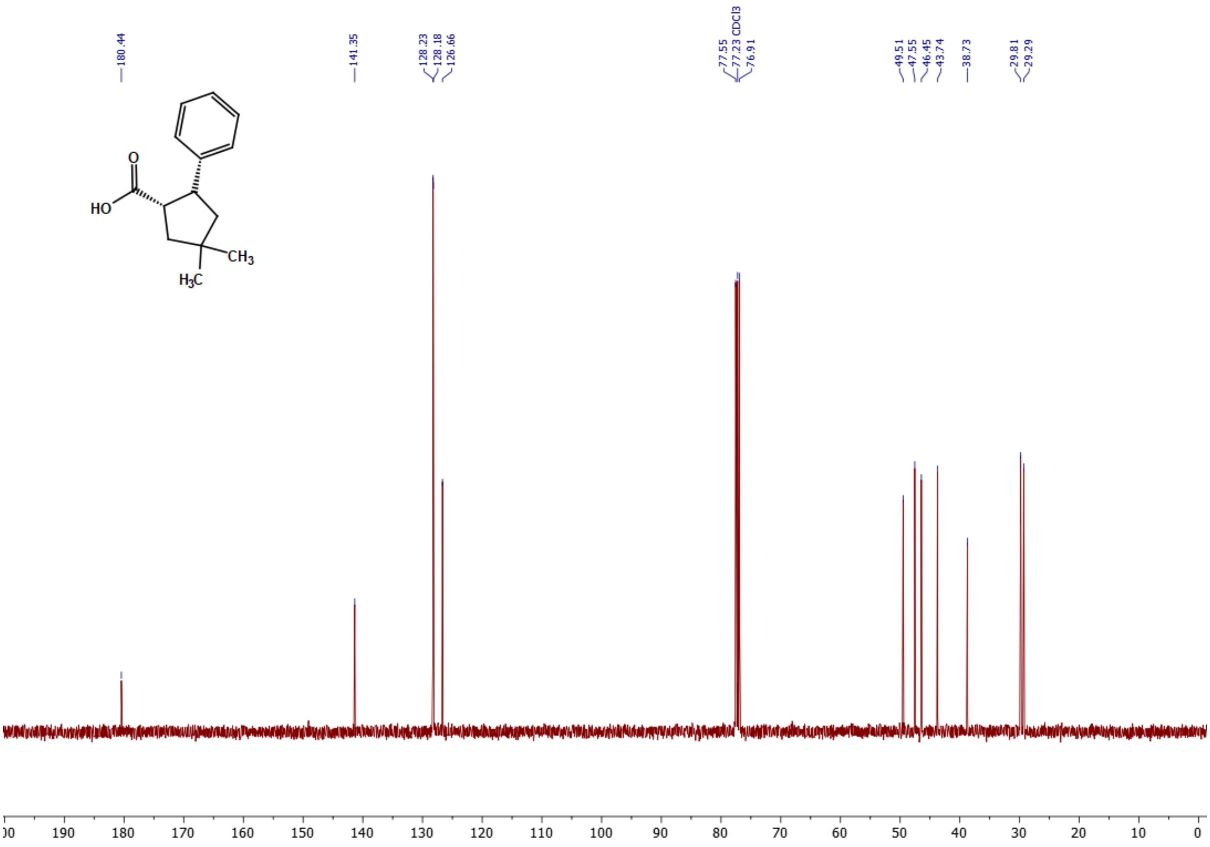

DEPT-135 NMR (101 MHz, Chloroform-*d*) (**8**):

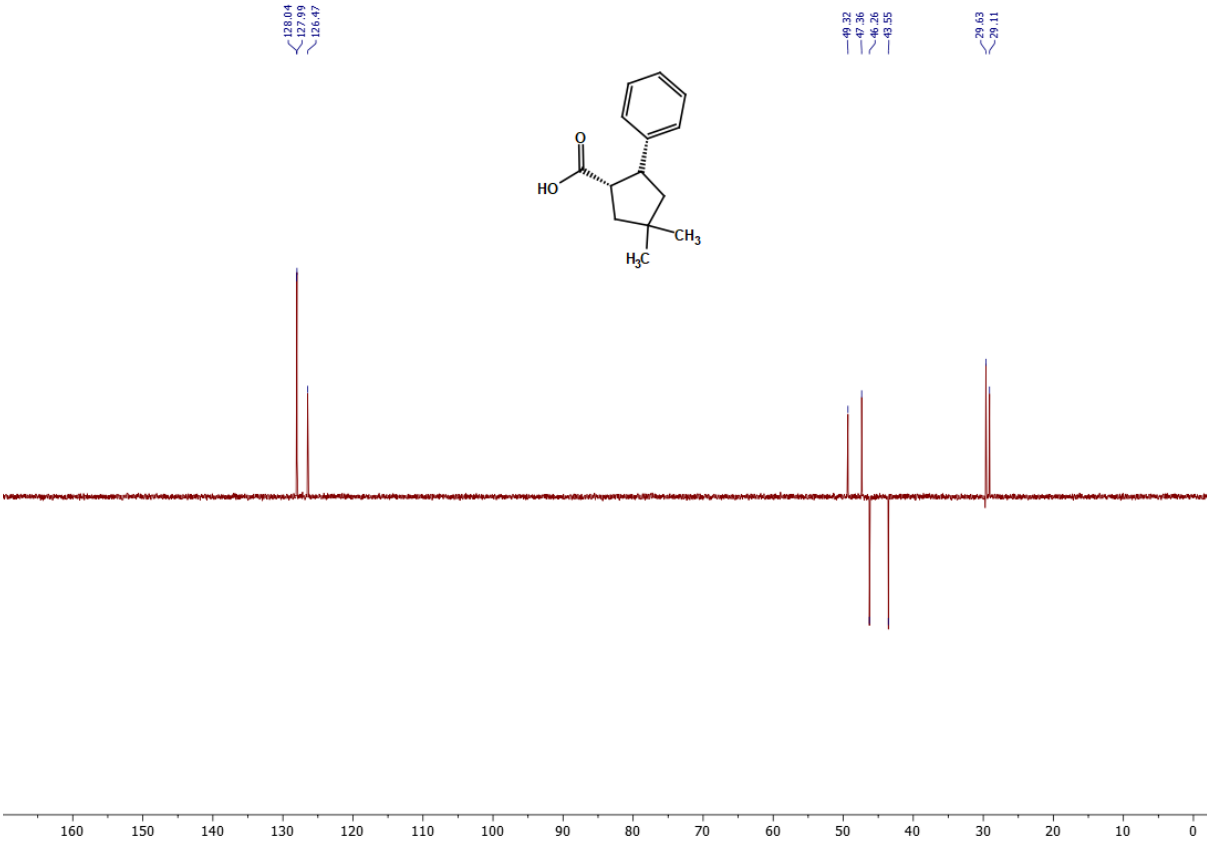

^{1}H NMR (400 MHz, Chloroform-*d*) (**9**):

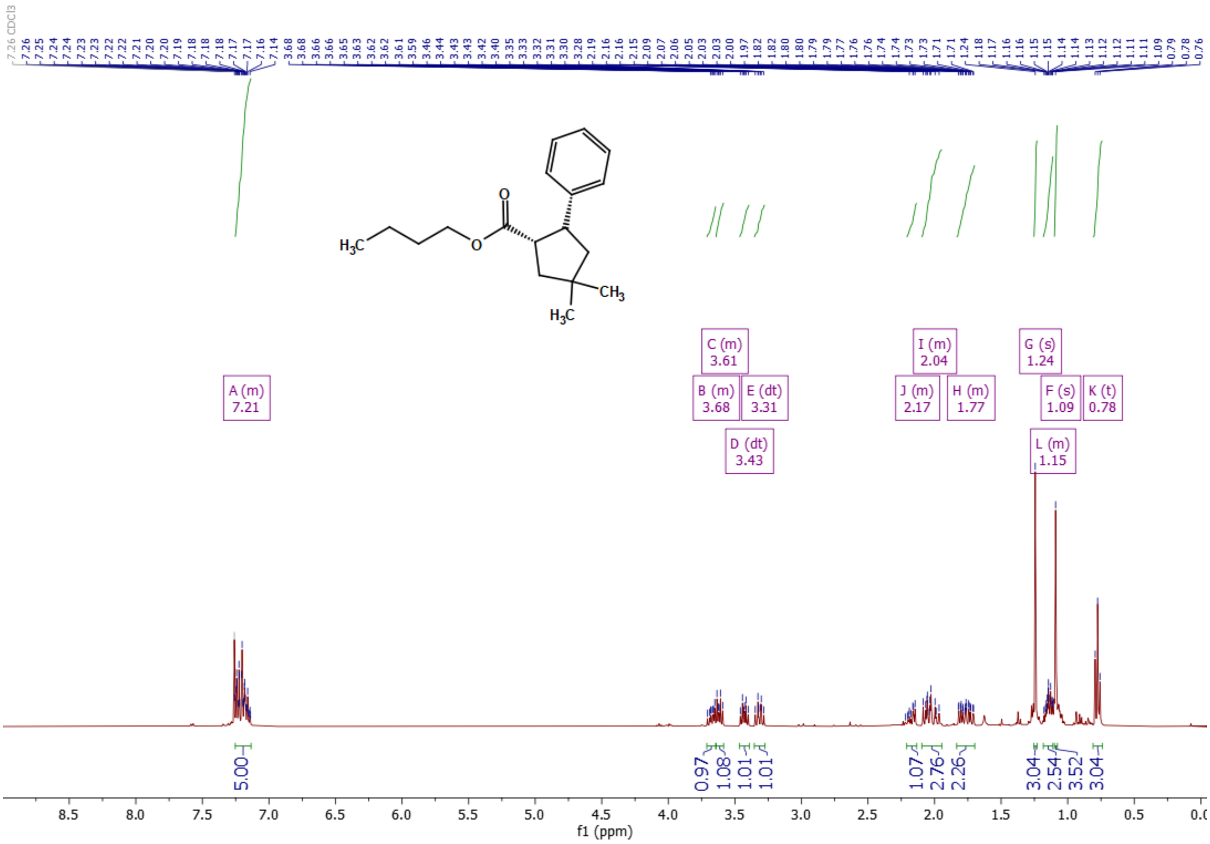

^{13}C NMR (101 MHz, Chloroform-*d*) (**9**):

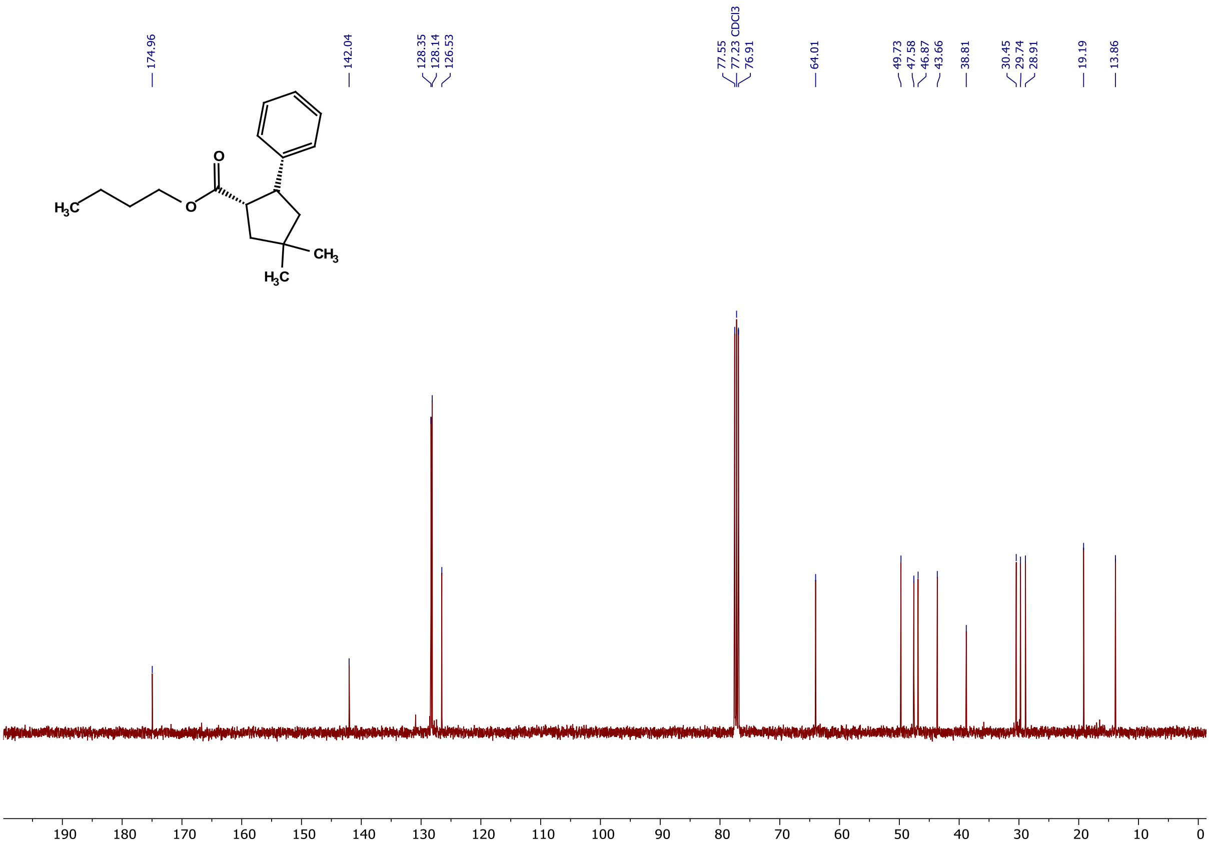

DEPT-135 NMR (101 MHz, Chloroform-*d*) (**9**):

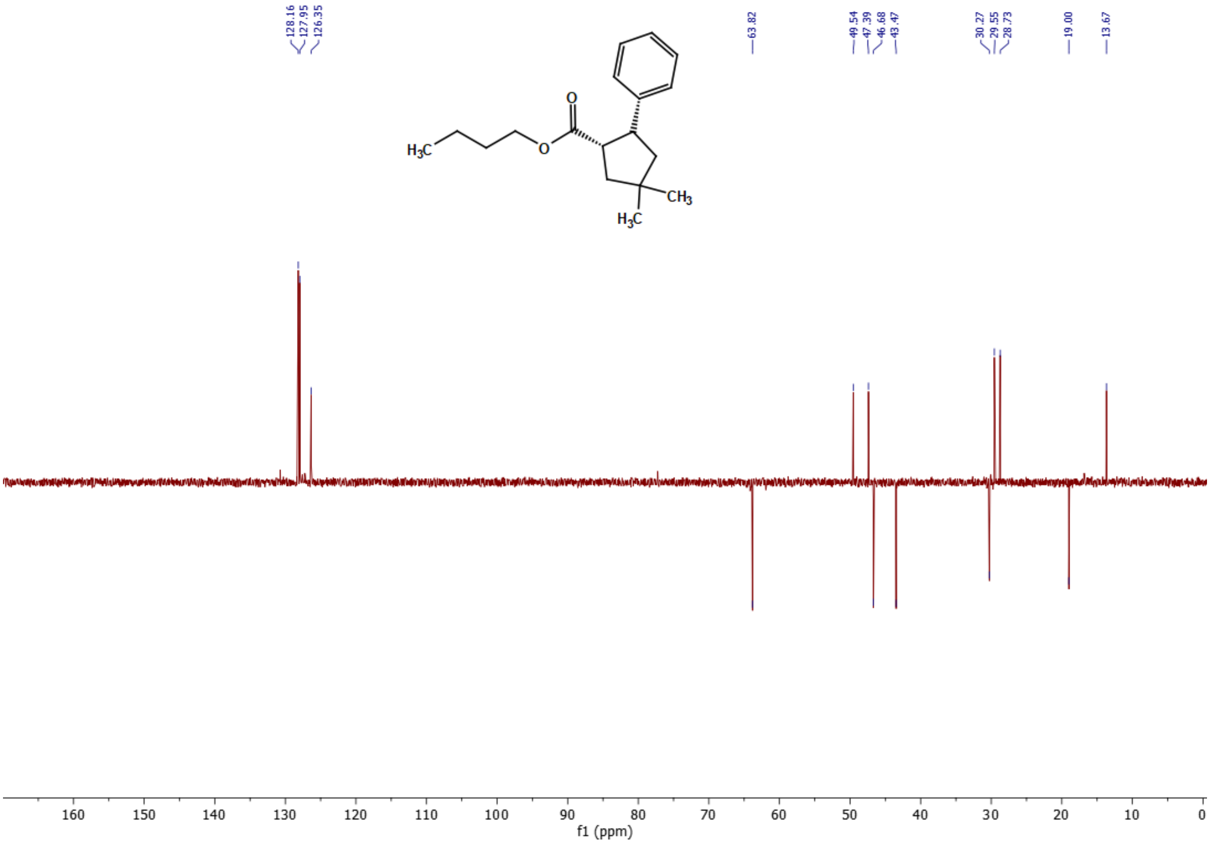

Supplement: Supplementary file 1 — ja1c01356_si_001.pdf [file ja1c01356_si_001.pdf]
